# Iron Deprivation in *Synechocystis*: Inference of Pathways, Non-coding RNAs, and Regulatory Elements from Comprehensive Expression Profiling

**DOI:** 10.1534/g3.112.003863

**Published:** 2012-12-01

**Authors:** Miguel A. Hernández-Prieto, Verena Schön, Jens Georg, Luísa Barreira, João Varela, Wolfgang R. Hess, Matthias E. Futschik

**Affiliations:** * Institute for Biotechnology and Bioengineering, Centre for Molecular; ‡ Centre for Marine Science, University of Algarve, Campus de Gambelas, 8005-139 Faro, Portugal; † Faculty of Biology, University of Freiburg, 79104 Freiburg, Germany

**Keywords:** iron homeostasis, expression profiling, regulation, non-coding RNA, cyanobacteria

## Abstract

Iron is an essential cofactor in many metabolic reactions. Mechanisms controlling iron homeostasis need to respond rapidly to changes in extracellular conditions, but they must also keep the concentration of intracellular iron under strict control to avoid the generation of damaging reactive oxygen species. Due to its role as a redox carrier in photosynthesis, the iron quota in cyanobacteria is about 10 times higher than in model enterobacteria. The molecular details of how such a high quota is regulated are obscure. Here we present experiments that shed light on the iron regulatory system in cyanobacteria. We measured time-resolved changes in gene expression after iron depletion in the cyanobacterium *Synechocystis* sp. PCC 6803 using a comprehensive microarray platform, monitoring both protein-coding and non-coding transcripts. In total, less than a fifth of all protein-coding genes were differentially expressed during the first 72 hr. Many of these proteins are associated with iron transport, photosynthesis, or ATP synthesis. Comparing our data with three previous studies, we identified a core set of 28 genes involved in iron stress response. Among them were genes important for assimilation of inorganic carbon, suggesting a link between the carbon and iron regulatory networks. Nine of the 28 genes have unknown functions and constitute key targets for further functional analysis. Statistical and clustering analyses identified 10 small RNAs, 62 antisense RNAs, four 5′UTRs, and seven intragenic elements as potential novel components of the iron regulatory network in *Synechocystis*. Hence, our genome-wide expression profiling indicates an unprecedented complexity in the iron regulatory network of cyanobacteria.

Iron is a prosthetic component of many enzymes, acting as biocatalyst or electron carrier, and is essential for almost all life forms. Proteins containing iron participate in a wide range of biological processes, including the tricarboxylic acid cycle, photosynthesis, respiration, H_2_ production, N_2_ fixation, gene regulation, and oxygen transport. Although iron is one of the most abundant metals on earth, it is difficult for microbial organisms to acquire due to its poor solubility under aerobic conditions at neutral pH. Thus, microbes have evolved a battery of mechanisms for iron uptake. At the same time, however, free intracellular iron needs to be kept at permissive levels, as it becomes toxic under aerobic conditions by producing reactive oxygen species. For these reasons, complex regulatory networks have evolved to tightly control intracellular iron concentrations, ensuring its essential function yet avoiding cellular damage (Cornelis *et al.* 2011).

In bacteria, iron homeostasis is best understood for *E. coli*. The key regulator sensing intracellular iron levels is the ferric uptake regulator (Fur). Fur contains two metal binding sites: a structural zinc binding site and a regulatory ferrous iron (Fe^2+^) binding site. At elevated iron concentration, Fe^2+^ binds to the regulatory site of the Fur monomer and triggers conformational changes, leading to dimerization and activation of Fur (Pecqueur *et al.* 2006). Activated Fur attaches to specific DNA sequences (termed Fur boxes) in the promoter regions of iron-acquisition genes and represses their transcription (Hantke 2001; McHugh *et al.* 2003). Thus, activation of Fur leads to a reduction in the influx of iron. Conversely, when the intracellular iron concentration decreases, Fur loses its bound Fe^2+^, becomes inactive, and detaches from the promoter regions. The repression of iron-acquisition genes is relieved, and the iron influx increases again. In brief, Fur acts as a negative feedback regulator for iron influx to keep the intracellular iron concentration at a constant level.

For some genes, expression was observed to be upregulated upon Fur activation (Dubrac and Touati 2000). This finding led to the discovery of a *trans*-acting small RNA (sRNA) termed RyhB, which turned out to be an important posttranscriptional regulator of iron homeostasis in *E. coli* (Masse and Gottesman 2002). RyhB binds to regions of partial complementarity in specific mRNAs (such as iron-containing superoxide dismutase) and promotes either their degradation or influences their rates of translation. As RyhB is itself a target for repression by Fur, there is an apparent positive regulation of these genes by Fur. Since its discovery in *E. coli*, functional homologs of RyhB have been found in many heterotrophic bacteria, such as Prrf1 and Prrf2 sRNAs in *Pseudomonas aeruginosa* (Wilderman *et al.* 2004). Although the details of this regulatory layer are not fully revealed, it is apparent that regulatory sRNAs provide specific advantages, which may not be possible to achieve through regulatory proteins (Hao *et al.* 2011; Salvail and Masse 2012).

In cyanobacteria, the regulation of iron homeostasis is expected to be more complex than in nonphotosynthetic bacteria (Shcolnick *et al.* 2009), due to the importance of iron in the photosynthetic electron transport chain. Indeed, the iron quota (atoms per cell) in *Synechocystis* sp. PCC 6803 (hereafter *Synechocystis*), is one order of magnitude higher than in *E. coli* (Finney and O’Halloran 2003; Shcolnick *et al.* 2009). A high regulatory complexity is also indicated by the existence of multiple transcription factors in cyanobacteria belonging to the Fur family of metalloregulators. *Synechocystis* and *Anabaena* PCC 7120 each possess 3 homologs, whereas at least 13 exist in the unicellular marine cyanobacterium *Acaryochloris marina* MBIC1107. Notably, these Fur-like homologs might not all necessarily be involved in the regulation of iron metabolism. One of the three Fur-like proteins of *Synechocystis*, Slr1738, seems to be a functional homolog of the peroxide-sensing PerR (Garcin *et al.* 2012; Li *et al.* 2004). Another, Sll1937, regulates the metabolism of Zn^2+^ and acts as a zinc uptake regulator, Zur (Tottey *et al.* 2012), whereas the third Fur-like protein, Sll0567, is a likely true functional homolog (FurA). Another layer of regulation exists in *Anabaena* PCC 7120, where one of the three Fur homologs is controlled by a *cis*-acting antisense RNA (asRNA) (Hernandez *et al.* 2006). Similar asRNAs complementary to *furA* were also reported for *Microcystis* and *Synechocystis* (Martin-Luna *et al.* 2011; Mitschke *et al.* 2011a; Sevilla *et al.* 2011). Finally, microarray-based RNA profiling indicated complex regulatory responses to changes of iron availability in both the marine cyanobacteria *Synechococcus* sp. WH7803 (Gierga *et al.* 2012) and *Prochlorococcus* (Thompson *et al.* 2011). These findings suggest substantial differences in the architecture of the iron regulatory networks of cyanobacteria compared with *E. coli*.

Iron homeostasis in cyanobacteria has been most intensively studied for *Synechocystis*, in which a complex transcriptional response, impacting several different metabolic pathways and cellular processes, has been reported (Shcolnick *et al.* 2009; Singh *et al.* 2003). One hallmark of the transcriptional response is the expression of the iron stress-induced *isiA* gene (*sll0247)*. Under severe iron starvation, both synthesis and the number of photosystem I complexes decreases, soluble light-harvesting complexes (phycobilisomes) are degraded, and *isiA* becomes highly transcribed (Guikema and Sherman 1983, 1984; Singh *et al.* 2003). The IsiA protein accumulates in the thylakoid membrane, binds chlorophyll, and can form multimeric rings around photosystem I. It is thought that these IsiA rings serve a dual purpose: initially the rings function as an extra light-harvesting complex, compensating for the reduced number of photosystem I complexes, and later they rather play a protective role (Havaux *et al.* 2003). The gene *isiA* appears to be under the control of Fur, although it was shown not to be the main mechanism controlling the iron stress inducibility of the *isiAB* operon (Kunert *et al.* 2003). Notably, an asRNA complementary to the *isiA* mRNA, iron stress-repressed RNA (IsrR), contributes to regulation of *isiA* expression (Dühring *et al.* 2006). Under iron-replete conditions, IsrR accumulates and becomes co-degraded with the *isiA* mRNA upon binding, preventing the synthesis of the IsiA protein (Dühring *et al.* 2006).

Although important insights have been gained in recent years about the molecular players involved in iron homeostasis, the detailed structure of the underlying regulatory network is unknown. For instance, no potential equivalents of the sRNA RyhB have been discovered in cyanobacteria yet. This is remarkable, as a large number of potentially *trans*-acting sRNAs and asRNAs have already been detected (Csiszar *et al.* 1987; Dühring *et al.* 2006; Gierga *et al.* 2009; Mitschke *et al.* 2011a,b; Nakamura *et al.* 2007; Sevilla *et al.* 2011). Their role in regulation, however, has remained largely uncharacterized and demands further study. Their characterization seems particularly important considering that more than 300 putative sRNAs and even more asRNAs have been reported for *Synechocystis* alone (Mitschke *et al.* 2011a).

To gain a more complete picture of iron homeostasis in cyanobacteria, we performed time-resolved genome-wide expression analysis of *Synechocystis* grown under iron-limiting conditions. We used customized oligonucleotide microarrays that detect both protein-coding and non-coding transcripts (ncRNA). The employed platform comprises over 42 000 probes, including the majority of previously detected ncRNA sequences (Georg *et al.* 2009; Mitschke *et al.* 2011a). The use of such a comprehensive platform helps to pinpoint ncRNAs involved in iron homeostasis, as well as identify their dynamic transcriptional response.

## Materials and Methods

### Growth conditions

*Synechocystis* wild type strain was grown at 30° in YBG-11, a modified version of BG-11 medium (Shcolnick *et al.* 2007). Light intensity was adjusted to 50 μmol photons m^−2^·s^−1^ of white light. Growth was monitored by the increase in optical density at 730 nm. Whole-cell spectra were recorded to track the shift in the chlorophyll *a* absorbance peak at 685 nm due to the accumulation of IsiA in the thylakoid membrane. The chelator desferrioxamine B (DFB, Sigma-Aldrich) was added at a final concentration of 100 µM to bubbled liquid cultures to induce iron starvation. The choice of DFB was motivated by a previous study showing its superior effectiveness compared with other chelating agents and with the alternative media exchange method (Shcolnick *et al.* 2009). Samples were taken before induction of iron depletion, as well as at 3, 12, 24, 48, and 72 hr after induction of iron depletion.

### RNA extraction and hybridization to microarray

*Synechocystis* cells (40–50 ml) were collected at different time points by rapid filtration (Pall Supor 800 Filter, 0.8 mm). The filters with the collected cells were transferred to a tube containing 2 ml of PGTX (Pinto *et al.* 2009), immediately frozen in liquid nitrogen, and stored at −80° until extraction. RNA was extracted following the protocol by Pinto and co-workers (Pinto *et al.* 2009). The purity and quality of the extracted RNA was assessed using a NanoDrop ND-1000 spectrophotometer (NanoDrop Technologies) and gel electrophoresis. A sample of 10 µg of RNA was treated with Turbo-DNase (Ambion) following the manufacturer's instructions, and then measured again with the NanoDrop ND-1000 spectrophotometer. Aliquots of the treated RNA (3 µg) were labeled directly with the Kreatech’s ULS labeling kit for Agilent gene expression arrays with Cy3, according to the manufacturer’s protocol. Hybridization was carried out with 1.65 µg RNA per array, according to the Agilent instructions for 4 × 44 k single-color microarrays. For each time point, two replicate hybridizations were carried out.

Customized single-color oligonucleotide microarrays (Agilent) were used for expression profiling. Their general design has been previously described (Georg *et al.* 2009). For our experiment, the set of probes included on the microarray was enlarged to cover newly detected ncRNAs. In total, our oligonucleotide microarray contained 42,303 probes (15,951 duplicates, 321 triplicates; and the rest, with a greater number of replicates), of which 21,022 probes were directed against all 3317 chromosome-located *Synechocystis* genes, as well as 82 of the 408 plasmid-located genes annotated in Cyanobase (http://genome.kazusa.or.jp/cyanobase). The microarray platform also comprised 5028 probes for 1875 intragenic elements referred to as “gene-int” (*e.g.* slr0898-int1), probes for 1939 asRNAs transcribed from the complementary strand of protein-coding genes and referred to as “gene-as” (*e.g.* sll0247-as), as well as probes for 608 sRNAs originating from intergenic regions, referred to as “NC-X” (*e.g.* NC-1). Microarray data were deposited at GEO database (GSE39804) and can be interactively accessed via the CyanoEXpress database at http://cyanoexpress.sysbiolab.eu (Hernandez-Prieto and Futschik 2012).

### Microarray data analysis

Signal intensities for probes were obtained from the scanned microarray image using Agilent Technologies’ Feature Extraction software version 10.5.1.1 (protocol GE1_105_Dec08). The R/Bioconductor platform was used for all preprocessing and statistical analysis. Background correction was carried out using the Limma package (Smyth 2005). To exclude genes that might not be reliably detected, a threshold for signal intensity was defined with reference to a set of 1403 control spots. The controls included empty spots, as well as spots for foreign spike-in RNA (which were not used in our experiment). Since signals from these spots only arise due to nonspecific hybridization, we defined genes as expressed only if their probe signals were higher than the maximal control spot signal in both arrays for at least one time point. Applying this procedure, 16,844 low-intensity probes, including probes for 614 protein-coding and 1286 non-coding transcripts, were excluded. After quantile normalization was performed, normalized values for replicated probes on the arrays were averaged. Differential expression was statistically evaluated by means of the Limma package based on a linear model with the signal intensities at time point 0 hr (before addition of DFB) as reference. *P*-values were converted to false discovery rates (FDR) by the Benjamini-Hochberg approach. Probes with an FDR (*q*-value) < 0.05 and absolute fold change (FC) ≥ 2 were listed as differentially expressed.

For functional enrichment analysis, we used the gene annotation provided by Cyanobase (http://genome.kazusa.or.jp/cyanobase/Synechocystis), where *Synechocystis* genes are associated with 18 main functions and 75 subfunctions. We included asRNA and sRNA genes as two additional classes to both main functions and subfunctions to assess potential overrepresentation of these genes in expression clusters or in the set of differentially expressed genes. Additionally, information from the KEGG pathway database was utilized. Here, we compiled lists of genes associated with 73 pathways with a minimum of five genes annotated for *Synechocystis*. As reference, we used the set of genes that were captured in the microarray platform and defined as expressed. The significance of whether differently expressed genes were enriched in genes associated with a functional category or pathway was calculated using the hypergeometric test (which is equivalent to Fisher's exact test). Derived *p*-values were adjusted for multiple testing, and FDRs were calculated using the Benjamini-Hochberg method. To obtain the functional composition of differentially expressed genes, the significance of enrichment was assessed separately for upregulated and downregulated genes.

Complementary to the standard enrichment analysis for differentially expressed genes, we conducted a gene set enrichment analysis (GSEA). Here, accumulated expression changes in predefined gene sets (as given by the functional categories in Cyanobase or KEGG pathways) were assessed for upregulation or downregulation at a given time point. To this end, the Bioconductor PGSEA package, which is a parametric variant of conventional GSEA, was used. Instead of deriving an enrichment score as performed by GSEA, the PGSEA method calculates a *z*-score for the mean fold change in a gene set. The *z*-score states the magnitude of difference (in units of standard deviations) of the observed mean fold change from the expected mean fold change for a random gene set of equal size. Corresponding *p*-values are then inferred by comparing the *z*-score against the normal distribution. As PGSEA is based on the central limit theorem, only gene sets with a minimum number of 10 genes were included. FDRs were derived from *p*-values using the Benjamini-Hochberg method.

### Clustering of expression data

To hierarchically cluster samples and genes based on differential expression, the software program Cluster was applied. After standardization of the expression values from the different time points, clustering by complete linkage based on centered correlation was carried out. The resulting files were visualized using Java TreeView software (Saldanha 2004).

For soft clustering, the Bioconductor package Mfuzz was utilized (Futschik and Carlisle 2005; Kumar and Futschik 2007). As reference, gene expression at time point 0 hr was taken. To exclude genes that did not show expression changes, a minimum standard deviation of 0.25 for logged fold changes was set. After filtering, the expression changes of 3139 genes were standardized to have a mean value equal to 0 and a standard deviation equal to 1. Genes were clustered based on their log_2_ FC over time using the fuzzy c-means algorithm (FCM). Parameters for the FCM (*i.e.* number of clusters *c* and fuzzifier *m*) were selected using a previously published approach described in the supporting information, *Extended Methods* and Figure S1.

### Northern blots

Northern hybridizations were performed from the separation of 3–6 µg of total RNA by denaturing gel electrophoresis as described by Georg *et al.* (2009). Single-stranded RNA probes were generated by *in vitro* transcription from amplicons containing the T7 promoter as previously described (Georg *et al.* 2009). All oligonucleotide primers used are listed in Table S1.

### Meta-analysis of microarray studies

Data on gene expression from previous *Synechocystis* microarray studies on iron homeostasis were collected from the supplemental material provided by the authors (Shcolnick *et al.* 2009; Singh *et al.* 2003) or from the public repository Gene Expression Omnibus (GEO) (Houot *et al.* 2007). The GEO datasets using two-color microarrays with dye swap were imported into R by the Bioconductor package GEOquery (Davis and Meltzer 2007), adjusted using optimized intensity-dependent normalization (Futschik and Crompton 2005), and statistically evaluated using the Limma package. For comparison between experiments, an absolute log_2_ FC ≥ 0.5 and *p*-value < 0.01 were set as common criteria for differential expression. These criteria were chosen to maximize the number of genes compared between the different studies, since for two studies (Shcolnick *et al.* 2009; Singh *et al.* 2003), only genes differentially expressed with respect to this cut-off were reported. It is important to note that the experimental approaches in the compared studies differed in several aspects, which are outlined in the supporting information.

## Results

### Microarray-based RNA profiling of *Synechocystis* gene expression

Gene expression was measured at different time points over a period of three days. First culture aliquots were already collected shortly after induction (*i.e.* 3 hr) to detect early transcriptional responses. Further measurements were taken at 12, 24, 48, and 72 hr. RNA was extracted, labeled, and directly hybridized onto the microarray without reverse transcription into cDNA. As a reference, gene expression at time point 0 hr (before iron depletion) was taken. In the reference sample (iron replete conditions), the most abundant transcripts were rRNAs and tRNAs (with over 44 times higher signal intensity than the average for all genes). Excluding tRNAs and rRNAs, the 40 most abundant transcriptional units (Table 1) included only six mRNAs and two asRNAs, but 24 sRNAs, *e.g.* SyR5 and the signal recognition particle RNA (SRP). Also, seven clustered regularly interspaced short palindromic repeats (CRISPR)-derived spacer RNAs transcribed from the pSYSA plasmid were abundant. The most strongly expressed protein-coding genes were those encoding the photosystem II core subunit D1 (*psbA3* and *psbA2*), the P700 apoprotein of photosystem I (*psaA*), the phycocyanin alpha subunit (*cpcA*), a homing endonuclease within the tRNAfMet group I intron (*slr0915*), and two proteins of unknown function (*slr1634* and *slr0376*) (Table 1).

**Table 1:** The 40 most abundant transcriptional units (besides rRNAs and tRNAs) at standard conditions judged by microarray probe intensity

Gene ID	Description	Location	Gene/Mean Intensity
SyR5	Small RNA	Chromosome	42.67
SRP	4.5S RNA component of the signal recognition particle	Chromosome	42.67
NC-396	Small RNA	Chromosome	41.98
slr1474-5′UTR	5′ UTR or part of SRP	Chromosome	41.91
NC-268	Small RNA	Chromosome	41.72
Yfr1	Small RNA	Chromosome	41.48
NC-945	Small RNA	Chromosome	41.16
NC-162	Small RNA	Chromosome	40.91
NC-398	Small RNA	Chromosome	40.34
NC-947	Small RNA	Chromosome	39.49
sll7075-as1	Antisense RNA	pSYSA	38.11
NC-946	Small RNA	Chromosome	38.09
NC-666	Small RNA	Chromosome	37.13
Leader-2	CRISPR2 leader	pSYSA	36.37
NC-144	Small RNA	Chromosome	35.95
Spacer9-2	CRISPR2 crRNA	pSYSA	34.90
NC-1783	Small RNA	Chromosome	33.32
NC-182	Small RNA	Chromosome	33.00
NC-247	Small RNA	Chromosome	31.32
sll1867	psbA3 (photosystem II D1 protein)	Chromosome	30.49
slr1311	psbA2 (photosystem II D1 protein)	Chromosome	29.99
Spacer1-2	CRISPR2 crRNA	pSYSA	29.67
Spacer2-2	CRISPR2 crRNA	pSYSA	29.60
slr1353-0-x	Antisense RNA	Chromosome	29.10
NC-289	Small RNA	Chromosome	29.01
NC-423	Small RNA	Chromosome	28.72
us29	Small RNA or 5′UTR of cmpA	Chromosome	26.24
NC-1545	Small RNA	Chromosome	26.08
NC-14	Small RNA	Chromosome	25.62
NC-105	Small RNA	Chromosome	25.07
NC-232	Small RNA	Chromosome	24.98
NC-128	Small RNA	Chromosome	22.98
NC-313	Small RNA	Chromosome	22.08
Spacer5-2	CRISPR2 crRNA	pSYSA	21.95
NC-690	Small RNA	Chromosome	21.83
slr0915	Putative endonuclease	Chromosome	21.69
Spacer1-0	CRISPR2 crRNA	pSYSA	21.47
Spacer14-2	CRISPR2 crRNA	pSYSA	21.07
slr1634	Hypothetical protein	Chromosome	20.60
sll1578	cpcA (phycocyanin alpha subunit)	Chromosome	20.57
slr1834	psaA (P700 apoprotein subunit Ia)	Chromosome	17.93

To obtain a measure for relative abundance of genes, the average probe intensity was divided by the mean intensity of all probes.

**Figure 1:**
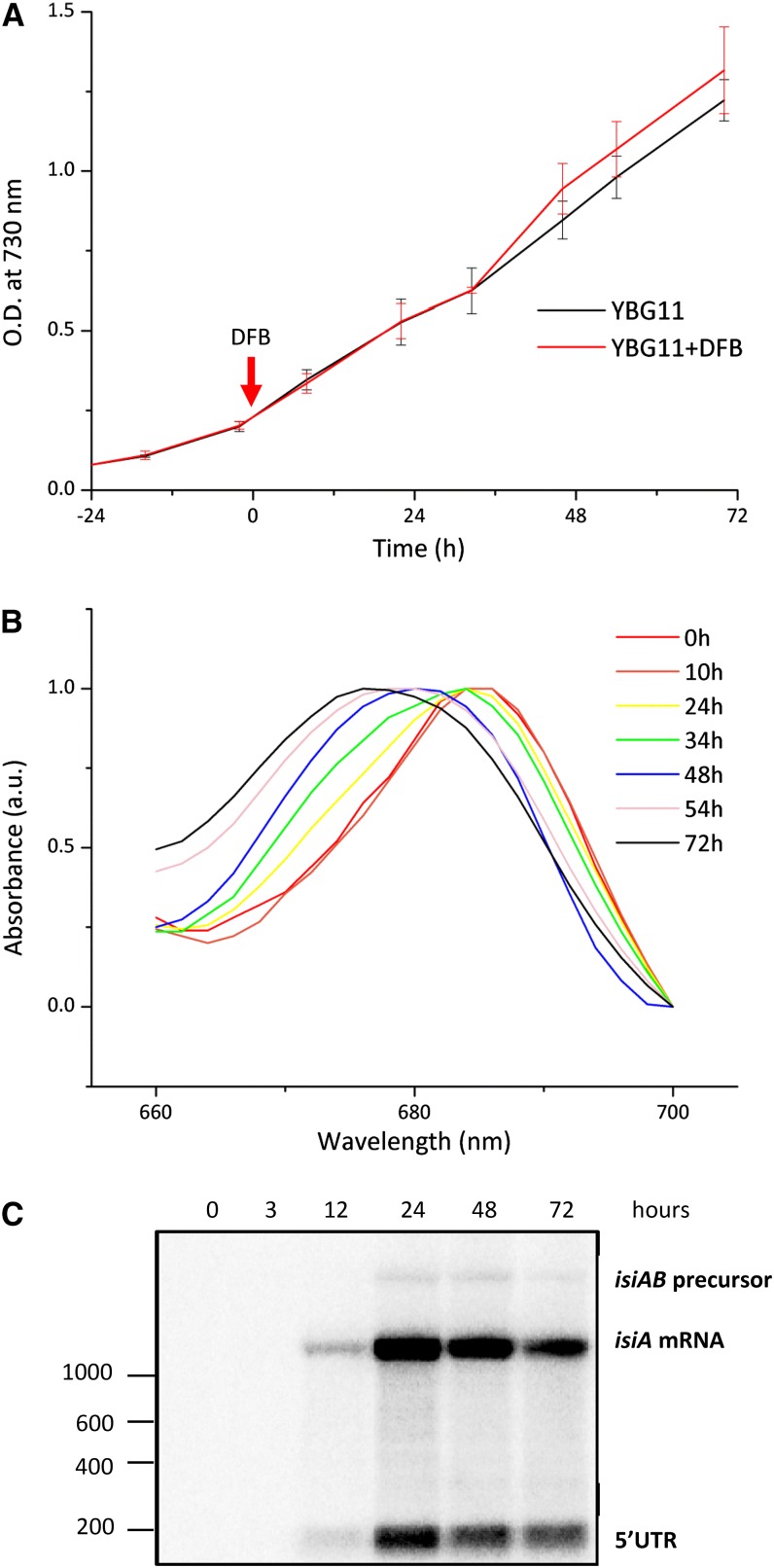
Growth of *Synechocystis* cultures in the presence or absence of DFB. (A) Two sets of cultures were grown in quadruplicate, and samples were taken twice per day over five days. The absorbance values measured at 730 nm from each quadruplicate were averaged and plotted against the sampling time (error bars represent the standard deviation). No statistically significant difference was observed for the two growth curves. (B) Absorption spectra of cultures following the addition of DFB to monitor the characteristic shift in the chlorophyll absorbance peak due to the accumulation of IsiA protein in the thylakoid membrane. Spectra were normalized to 1 at their maximum absorbance value while the absorbance value at 700 nm was taken as 0. (C) Northern blots for the detection of transcripts from the *isiA* locus for *isiA* mRNA, the *isiAB* precursor, and its 5′UTR over a period of 72 hr of iron depletion. A strong increase of monocistronic *isiA* transcript levels within the first 24 hr is apparent. The length of selected marker bands is given to the left.

Addition of desferrioxamine B (DFB) did not affect the growth rate of *Synechocystis* during the time span of the experiment (Figure 1A), but it triggered the iron stress response. As a marker for iron stress response, the characteristic blue shift of the whole-cell absorption spectra due to accumulation of IsiA in the thylakoid membrane became evident 34 hr after the addition of DFB (Figure 1B). A strong induction of *isiA* expression was confirmed by Northern blot analysis (Figure 1C).

### Identification and classification of differentially expressed transcripts

Imposing a threshold for minimum reliable expression, 2746 protein-coding and 1286 non-coding transcriptional units were classified as expressed at least at one time point. Transcripts were considered significantly differentially expressed when the corresponding *q*-value was lower than 0.05 and their absolute log_2_FC value was equal or larger than 1 (*i.e.* a minimum 2-fold upregulation or downregulation was required). In total, 1076 transcription units were detected as differentially expressed for at least one time point after DFB addition. These transcription units consisted of 644 mRNAs and 432 ncRNAs (comprising 307 asRNAs and 125 sRNAs).

We also detected significant expression changes for 321 intragenic elements and 176 UTRs of annotated genes. Most intragenic elements and UTRs displayed expression patterns similar to those of their respective genes upon DFB addition. A few notable exceptions were detected, pointing to additional layers of regulation for these genes in their response to iron limitation (Figure S3 and Figure S4). Four 5′UTR-gene pairs accumulated in a different manner with respect to each other (Figure S3A). These pairs include (i) *ssr2333*, encoding FeoA, a potential activator of iron transport (Su *et al.* 2010); (ii) *slr0040*, encoding the bicarbonate transport system substrate-binding protein CmpA (Koropatkin *et al.* 2007); (iii) *slr1964*, encoding the fluorescence recovery protein (FRP) involved in phycobilisome-dependent non-photochemical quenching (Boulay *et al.* 2010); and (iv) *slr0074*, encoding SufB, involved in assembly of [Fe-S] clusters (Shen *et al.* 2007). Possibly, the distinct expression profiles of these genes and their 5′UTR indicate existence of riboswitches, although further experimental work is required to support such a hypothesis. We also noted that 7 of the 108 analyzed intragenic element/gene pairs accumulated in a different manner (Figure S3B). These genes comprise (i) *sll1020*, encoding a probable glycosyltransferase; (ii) *slr0423*, encoding the rare lipoprotein A; (iii) *sll0450* (*norB*), encoding the cytochrome *b* subunit of the nitric oxide reductase; (iv) *sll1550*, encoding a porin; (v) *slr0338*, encoding a probable oxidoreductase; as well as (vi) *sll0518* and (vii) *slr0913*, encoding proteins of unknown function. Whether the distinct expression of intragenic elements is caused by short sense transcripts, truncated alternative mRNAs, or other transcriptional changes due to iron limitation remains to be determined.

### Identification of main temporal response patterns

**Figure 2:**
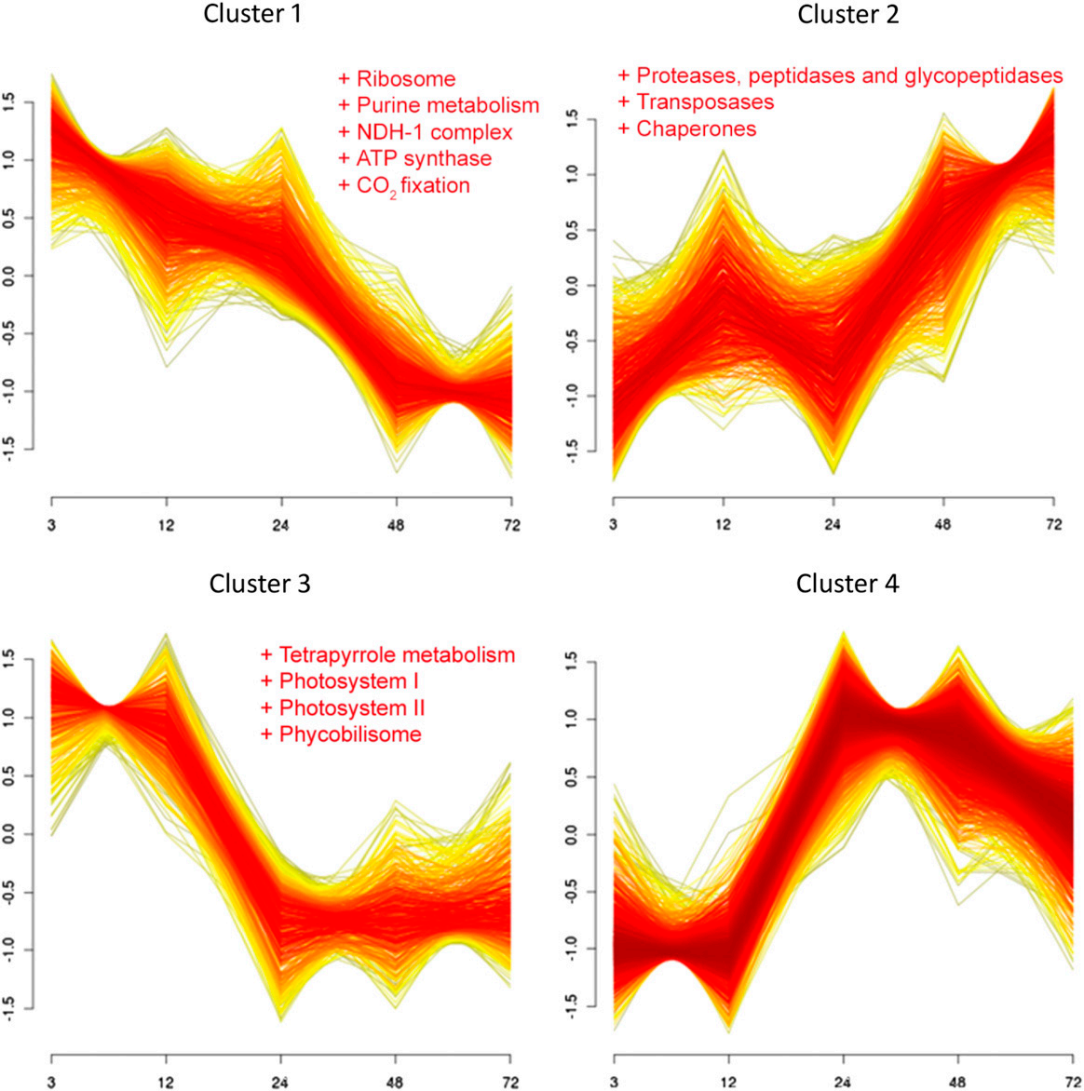
Main temporal patterns of expression response to iron limitation. Expression profiles of four detected clusters with 688, 664, 550, and 1237 genes are shown (only genes with a cluster membership higher than 0.5 were included). Each line represents the standardized expression changes of a gene with mean equal to 0 and standard deviation equal to 1. Thus, the display expression changes represent the temporal trend rather than real expression changes with respect to the non-stressed sample. The color of the line indicates the cluster membership value assigned by soft clustering, with shades of yellow and red symbolizing lower and higher membership, respectively. Functional categories significantly represented (FDR < 0.2) in each cluster are displayed.

To gain an overview of the major transcriptional response patterns, we analyzed log_2_FC corresponding to 3139 genes using soft clustering, a noise-robust method to reveal the major patterns of co-expression (Futschik and Carlisle 2005). Parameters for soft clustering were derived as previously suggested (Schwammle and Jensen 2010). In total, four clusters with distinct profiles were found (Figure 2). Membership values for each gene in the corresponding cluster can be found in Table S2.

Cluster 1 contains genes whose expression decreased gradually during the experiment. Functional enrichment analysis of this set of genes showed an overrepresentation of genes encoding ribosomal proteins (FDR = 5.33 × 10^−7^), enzymes of the purine metabolism (FDR = 7.75 × 10^−3^), subunits of the NDH complex (FDR = 8.35 × 10^−3^), ATP synthase (FDR = 2.26 × 10^−5^) complexes, as well as enzymes involved in carbon fixation (FDR = 2.73 × 10^−5^). In contrast, cluster 2 comprised genes whose expression gradually increased. This cluster is enriched in genes encoding chaperones (four out of five genes in this subcategory, FDR = 0.13) and proteins with a role in the degradation of proteins, peptides, and glycopeptides (five out of eight, FDR = 0.15).

Both clusters 3 and 4 displayed more transient transcriptional response patterns with distinctive changes after 12 hr. For cluster 3, transcripts accumulated during the first 12 hr after the stress induction but displayed noticeably lower levels for the following 60 hr. Genes in this cluster tended to be associated with tetrapyrrole metabolism (FDR = 0.01) or with proteins of photosystem I (FDR = 1.62 × 10^−4^), photosystem II (FDR = 0.01), and the phycobilisome (FDR = 2.65 × 10^−4^). Finally, cluster 4, which was also the largest cluster, contained transcripts whose levels were strongly reduced during the first 12 hr, but increased again after 24 hr. Genes associated with this cluster are quite heterogeneous; no functional category or subcategory was significantly overrepresented in this set.

### Functional composition of the set of differentially expressed genes

**Figure 3:**
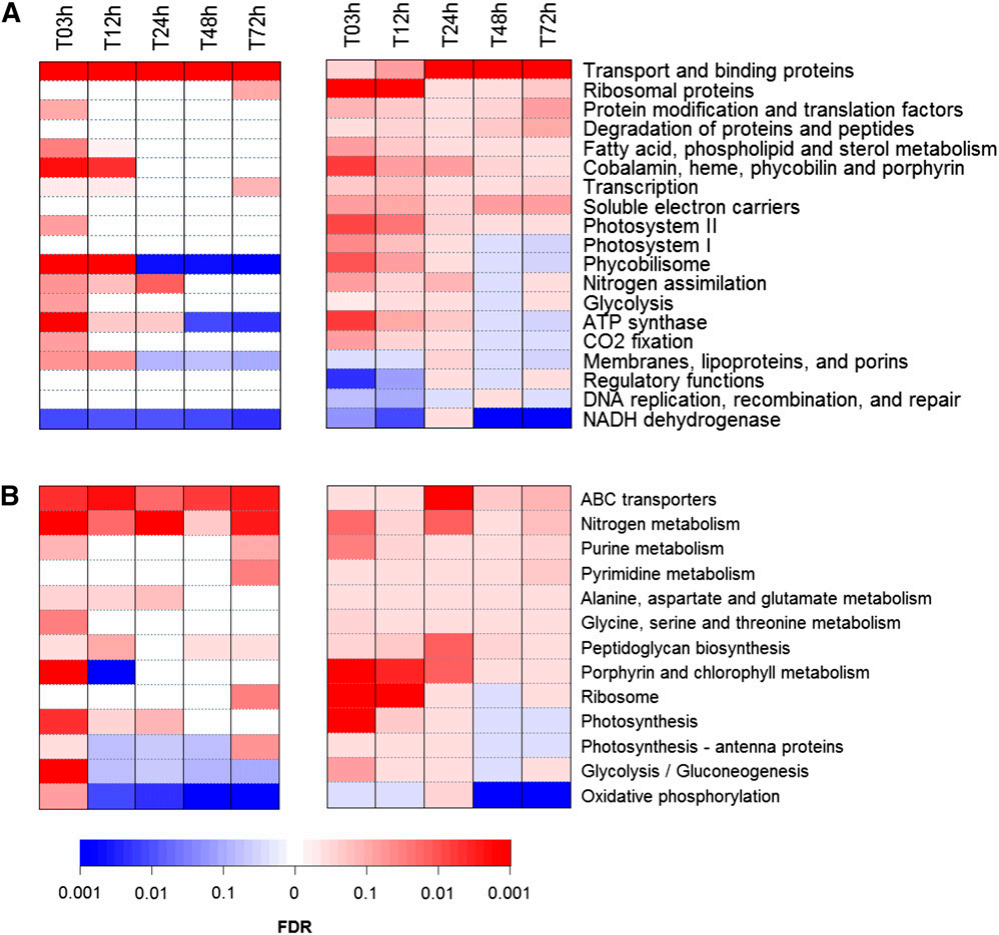
Enrichment analysis of differential gene expression. The heatmaps depict the significance (FDR) for (A) the functional categories from Cyanobase as well as (B) KEGG pathways associated with differential expression upon iron depletion. The results for enrichment analysis of differentially expressed genes (EADEG) are shown on the left side, whereas the results for gene set enrichment analysis (GSEA) are displayed on the right side. Shades of red indicate upregulation; shades of blue indicate downregulation. The bar at the bottom shows the FDR associated with colors. The exact values can be found in Table S2. Functional categories or pathways are displayed if the corresponding FDR is smaller than 0.1 at one time point for EADEG or GSEA.

For a stringent identification of processes and pathways involved in the transcriptional response to iron limitation, we employed two complementary statistical tools: standard enrichment analysis of differentially expressed genes (EADEG) and gene set enrichment analysis (GSEA). Whereas the first method seeks to pick up functional categories, including a larger number of differentially expressed genes than would be expected by chance; the second method compares the average expression of genes within a category and tends to be more sensitive to modest but coordinated changes in expression (see examples in Figure S2). We applied both methods to our expression data using either the gene functions defined in the Cyanobase database or pathways defined in KEGG database as categories (Table S3). The significance for upregulation or downregulation of functions and pathways at different time points is displayed as heatmaps in Figure 3. In general, results of both methods agreed well.

Both upregulation and downregulation of processes and pathways were observed. Some response patterns were highly dynamic and indicated that several processes switched from initial activation to subsequent repression over the course of the experiment. Notably, such a pattern was observed for genes associated with photosynthesis. Transcripts linked to photosystem I and II, phycobilisome, ATP synthase, carbon fixation, as well as porphyrin and chlorophyll metabolism (as defined in Cyanobase) tended to be transiently accumulated during the first 12 hr, but showed lower abundance afterward. Remarkably, a trend to decreased expression at later time points was also observed for genes in the KEGG pathway “oxidative phosphorylation.” This coordination in expression may result from an interconnection on the molecular level. In contrast to higher plants, photosynthesis and oxidative phosphorylation share several components in cyanobacteria (plastoquinone pool, soluble electron carriers, cytochrome *b_6_f*, ATP synthase complex, and terminal oxidases), whereas genes associated with porphyrin and chlorophyll metabolism encode proteins involved in the metabolism of the associated prosthetic groups (heme, chlorophyll *a*, pheophytin, and phycobilins). Thus, a similarity in transcript accumulation might be expected. It should be noted, however, that the reduction in the “oxidative phosphorylation” and “photosystem I” categories tended to be stronger than for the “photosystem II” category (Figure 3). Interestingly, an initial increase of transcripts of ribosomal proteins was also detected by GSEA (Figure 3B, right panel), similar to the pattern described for photosynthetic genes. This finding is consistent with the fact that photosynthetic proteins are quantitatively dominant in the cell and some of them have a fast turnover (Nixon *et al.* 2010). Thus, the observed transient accumulation in transcripts of photosynthetic genes needs to be matched by an increased capacity in protein synthesis.

For other functional categories, we observed more persistent accumulation or depletion in transcripts. Genes encoding for transport and binding proteins tended to be overexpressed throughout the time course. Also, significant enrichment in upregulated genes associated with nitrogen metabolism was detected over the whole time span covered by the experiment. This pattern was contrasted by a strong reduction of transcripts encoding subunits of the NADH dehydrogenase (NDH) complex, which perform several distinct roles in cyanobacteria, like respiration, cyclic electron flow around photosystem I, and CO_2_ uptake (Prommeenate *et al.* 2004). Here, 11 out of 22 genes linked to the NDH complex showed downregulation at all time points. A more detailed assessment of the dynamic expression of the individual genes is given in the following section.

### Differential expression of protein-coding genes involved in the transport and mobilization of iron and the assimilation of nitrogen and inorganic carbon

Although changes in absorbance spectra were only observed at the end of the second day (Figure 1B); the addition of DFB to the cultures induced an immediate response on transcript level. Already three hours after the addition of DFB, 458 protein-coding genes, 280 asRNAs, and 74 sRNAs showed an absolute log_2_FC in their expression intensities greater than 1. Most notably, the *isiAB* operon, as well as genes encoding subunits of iron transporters were highly induced (up to 6-fold at this time point) compared with the reference sample (Table 2 and Table S4). After 12 hr of iron limitation, 408 protein-coding genes were upregulated. In addition to *isiA* and *isiB*, increased transcript levels were observed for bacterioferritin-associated ferredoxin (*bfd*, *ssl2250*), which is involved in the mobilization of ferric iron from bacterioferritin. Higher transcript levels were also found for components of various iron transport systems: ferrichrome (*fhuA*, *sll1406*), ferric ions (*futA*, *slr1295*), ferric citrate (*fecE*, *slr1318*), as well as components of the Ton system (*exbB*, *sll1404*).

**Table 2:** The 60 most significantly differentially expressed protein-coding genes

Funtional Subcategory	Gene ID	Gene Name	3 hr	12 hr	24 hr	48 hr	72 hr	*q*
Glutamate family / nitrogen assimilation	**slr0898**	**Ferredoxin-nitrite reductase**	**2.53**	**1.88**	**2.94**	**1.29**	**2.20**	**2.20∙10^−8^**
	**slr1756**	**Glutamate-ammonia ligase**	**2.53**	**2.20**	**1.73**	**1.19**	**1.85**	**6.69∙10^−8^**
	sll1515	Glutamine synthetase inactivating factor IF17	−2.63	−2.64	−2.66	−1.32	−1.13	7.79∙10^−10^
	ssl1911	Glutamine synthetase inactivating factor IF7	−3.81	−3.29	−3.28	−1.35	−1.72	2.19∙10^−10^
Membranes, lipoproteins, and porins	slr0042	Probable porin; major outer membrane protein	−3.82	−3.84	−4.01	−3.42	−3.77	3.19∙10^−8^
Photosystem I	**sll0247**	**Iron-stress chlorophyll-binding protein, IsiA**	**2.52**	**5.99**	**5.53**	**6.14**	**6.15**	**2.19∙10^−7^**
NADH dehydrogenase	slr1279	NADH dehydrogenase sub 3	−1.17	−1.29	−1.67	−2.29	−2.14	2.16∙10^−5^
	sll0223	NADH dehydrogenase sub 2	−1.15	−1.26	−1.82	−2.43	−2.33	1.42∙10^−6^
	sll1732	NADH dehydrogenase sub 5	−2.64	−2.24	−2.44	−3.49	−3.49	1.47∙10^−6^
	sll1733	NADH dehydrogenase sub 4	−3.02	−4.05	−3.61	−3.8	−3.74	2.24∙10^−9^
Soluble electron carriers	**sll0248**	**Flavodoxin, IsiB**	**2.83**	**6.21**	**5.44**	**6.66**	**6.86**	**5.57∙10^−10^**
Regulatory functions	slr1214	Two-component response regulator PatA subfamily	−2.03	−3.40	−1.46	−2.31	−2.07	2.49∙10^−7^
Nucleoproteins	**sll0517**	**Putative RNA binding protein**	**1.34**	**1.82**	**1.79**	**1.73**	**2.02**	**1.78∙10^−8^**
Transport and binding proteins	**sll1206**	**Ferric aerobactin receptor, FhuA homolog**	**3.08**	**5.18**	**4.74**	**5.56**	**5.73**	**1.98∙10^−10^**
	**sll1404**	**Biopolymer transport∙10xbB protein homolog**	**3.02**	**4.88**	**4.17**	**4.58**	**4.80**	**1.98∙10^−10^**
	**sll1405**	**Biopolymer transport∙10xbD protein homolog**	**2.61**	**4.52**	**3.75**	**4.29**	**4.33**	**1.59∙10^−7^**
	**slr1295**	**Iron transport system substrate-binding protein**	**3.23**	**4.05**	**3.69**	**4.10**	**4.30**	**1.45∙10^−10^**
	**slr0513**	**Iron transport system substrate-binding periplasmic protein**	**3.32**	**4.00**	**3.64**	**4.00**	**3.96**	**1.45∙10^−10^**
	**sll1450**	**Nitrate/nitrite transport system substrate-binding protein**	**2.97**	**1.90**	**3.64**	**1.99**	**2.45**	**5.80∙10^−9^**
	**sll1406**	**Ferrichrome-iron receptor**	**1.54**	**3.51**	**2.43**	**2.75**	**2.52**	**8.86∙10^−8^**
	**slr1392**	**Ferrous iron transport protein B**	**1.12**	**2.80**	**2.12**	**2.74**	**2.96**	**9.97∙10^−9^**
	**sll1878**	**Iron(III)-transport ATP-binding protein**	**1.53**	**2.15**	**1.98**	**2.29**	**2.60**	**1.42∙10^−7^**
	**slr1488**	**Multidrug resistance family ABC transporter**	**0.59**	**2.33**	**1.82**	**2.55**	**2.89**	**1.60∙10^−9^**
	**sll0834**	**Low-affinity sulfate transporter**	**2.13**	**2.38**	**1.45**	**1.57**	**1.50**	**3.85∙10^−8^**
	**slr0074**	**ABC transporter subunit**	**0.92**	**1.91**	**1.23**	**1.75**	**2.08**	**2.13∙10^−8^**
	slr0044	Bicarbonate transport system ATP-binding protein	−3.29	−3.35	−3.16	−2.89	−3.00	1.30∙10^−7^
	slr0040	Bicarbonate transport system substrate-binding protein	−3.88	−4.11	−3.00	−3.34	−3.64	5.29∙10^−8^
	slr0043	Bicarbonate transport system ATP-binding protein	−3.70	−3.84	−4.05	−3.91	−3.91	4.80∙10^−10^
	slr0041	Bicarbonate transport system permease protein	−5.22	−5.33	−5.07	−4.68	−5.00	4.91∙10^−9^
Other categories	**ssl2250**	**Bacterioferritin-associated ferredoxin**	**1.66**	**2.54**	**2.22**	**2.97**	**3.18**	**2.78∙10^−8^**
	**sll1407**	**Probable methyltransferase**	**0.53**	**2.35**	**1.46**	**2.10**	**1.95**	**1.91∙10^−8^**
	sll0217	Flavoprotein	−6.26	−5.97	−5.21	−6.21	−6.10	2.38∙10^−8^
	sll0219	Flavoprotein	−5.89	−6.27	−5.79	−5.75	−6.09	6.29∙10^−11^
Unknown	**sll0249**	**Hypothetical protein**	**1.39**	**4.74**	**3.64**	**5.34**	**5.33**	**4.80∙10^−10^**
	**sll1549**	**Salt-enhanced periplasmic protein**	**1.74**	**3.93**	**3.12**	**3.70**	**3.87**	**5.57∙10^−10^**
	**ssl0461**	**Hypothetical protein**	**0.89**	**2.83**	**1.87**	**3.37**	**3.42**	**9.79∙10^−10^**
	**slr1964**	**Hypothetical protein**	**1.97**	**1.74**	**1.54**	**2.06**	**1.81**	**1.67∙10^−4^**
	sll1004	Hypothetical protein	−1.67	−2.16	−1.48	−1.79	−1.47	4.03∙10^−5^
	sll0788	Hypothetical protein	−2.32	−2.16	−2.26	−1.70	−1.35	9.66∙10^−8^
	ssr1528	Hypothetical protein	−1.83	−2.11	−2.32	−2.32	−2.06	8.32∙10^−8^
	sll1735	Hypothetical protein	−2.48	−2.87	−1.96	−2.95	−2.84	3.04∙10^−9^
	sll1734	Protein involved in low CO2-inducible	−3.20	−4.05	−2.99	−3.62	−3.52	2.55∙10^−9^
	slr1513	Periplasmic protein, function unknown	−3.46	−4.49	−3.41	−5.16	−4.90	1.26∙10^−9^
	slr1512	Sodium-dependent bicarbonate transporter	−3.67	−4.30	−3.46	−5.54	−5.28	1.42∙10^−9^
	sll0218	Hypothetical protein	−6.35	−6.51	−5.84	−6.32	−6.47	2.70∙10^−11^
	**slr1485**	**Phosphatidylinositol phosphate kinase**	**2.09**	**3.92**	**3.46**	**3.86**	**4.47**	**9.48∙10^−9^**
	**ssr2333**	**Unknown protein**	**2.14**	**3.78**	**3.19**	**3.86**	**4.13**	**2.38∙10^−8^**
	**slr1484**	**Unknown protein**	**1.73**	**3.60**	**2.89**	**3.47**	**3.77**	**6.72∙10^−8^**
	**sll0327**	**Unknown protein**	**2.26**	**2.48**	**1.69**	**0.99**	**1.87**	**1.28∙10^−6^**
	**sll1862**	**Unknown protein**	**-0.28**	**0.24**	**0.80**	**3.58**	**4.36**	**3.45∙10^−10^**
	**slr0514**	**Unknown protein**	**1.36**	**1.77**	**1.46**	**1.59**	**1.76**	**4.26∙10^−8^**
	**sll1863**	**Unknown protein**	**-0.92**	**-0.26**	**0.59**	**3.53**	**4.51**	**4.75∙10^−10^**
	ssr1038	Unknown protein	−2.73	−1.70	−2.34	−0.98	−1.08	5.81∙10^−7^
	slr0476	Unknown protein	−1.23	−1.15	−1.78	−2.66	−2.38	3.99∙10^−8^
	sll0266	Unknown protein	−3.05	−2.99	−1.37	−0.58	−1.23	9.92∙10^−7^
	slr1667	Target gene of sycrp1	−5.75	−2.55	−2.69	−0.08	−0.21	5.74∙10^−6^
	slr0616	Unknown protein	−3.57	−3.56	−3.15	−3.51	−3.40	3.53∙10^−6^

Genes whose transcripts accumulated after the addition of DFB are in bold. Genes are ordered based on their functional category classification in Cyanobase.

Among the most strongly downregulated genes were those encoding the iron-containing flavodiiron proteins Flv4 (*sll0217*) and Flv2 (*sll0219*), as well as the co-transcribed *sll0218* encoding a protein of unknown function. Other downregulated genes can be linked to the transport and accumulation of inorganic carbon, such as subunits of the bicarbonate transport system CmpABCD (*slr0040–slr0044*) and subunits of the NDH complex involved in CO_2_ uptake (Table 2).

Several genes associated with nitrogen assimilation and metabolism were differentially expressed. Genes for transport (*nrtABC*, *sll1450-sll1452*) and reduction of nitrate (*nirA*, *slr0898*), as well as two genes encoding glutamine synthetase (*glnN*, *slr0288*, and *glnA*, *slr1756*), were upregulated. Notably, NtcA (*sll1423*), the global nitrogen regulator, was also upregulated after addition of DFB. In contrast, genes encoding the glutamine synthetase inactivating factors IF17 (*gifB*, *sll1515*) and IF7 (*gifA*, *ssl1911*) were downregulated.

Among the 22 differentially expressed genes that are associated in Cyanobase with regulatory functions, we found upregulated the *ndhR* repressor (*sll1594*), consistent with the downregulation of its target genes *sll1734* and *slr1512*; *nusG* (*sll1742*) encoding the transcription antitermination protein; members of two-component regulatory systems (*e.g. rre23/sll1879*, *hik5/sll1888*); the cyanobacterial phytochrome 1 (*cph1*, *slr0474*); and the co-transcribed gene *rcp1* (*slr0473*) encoding the phytochrome 1 response regulator (Table S4).

### Comparison with previous microarray studies reveals a core set of iron response genes

The results obtained from the gene expression profiling were compared with those of three previously published microarray studies (Houot *et al.* 2007; Shcolnick *et al.* 2009; Singh *et al.* 2003). As common threshold criteria for differential expression, absolute log_2_FC ≥ 0.5 and *p*-value < 0.01 were set (see supporting information and Figure S5 for a detailed comparison of the studies).

**Figure 4:**
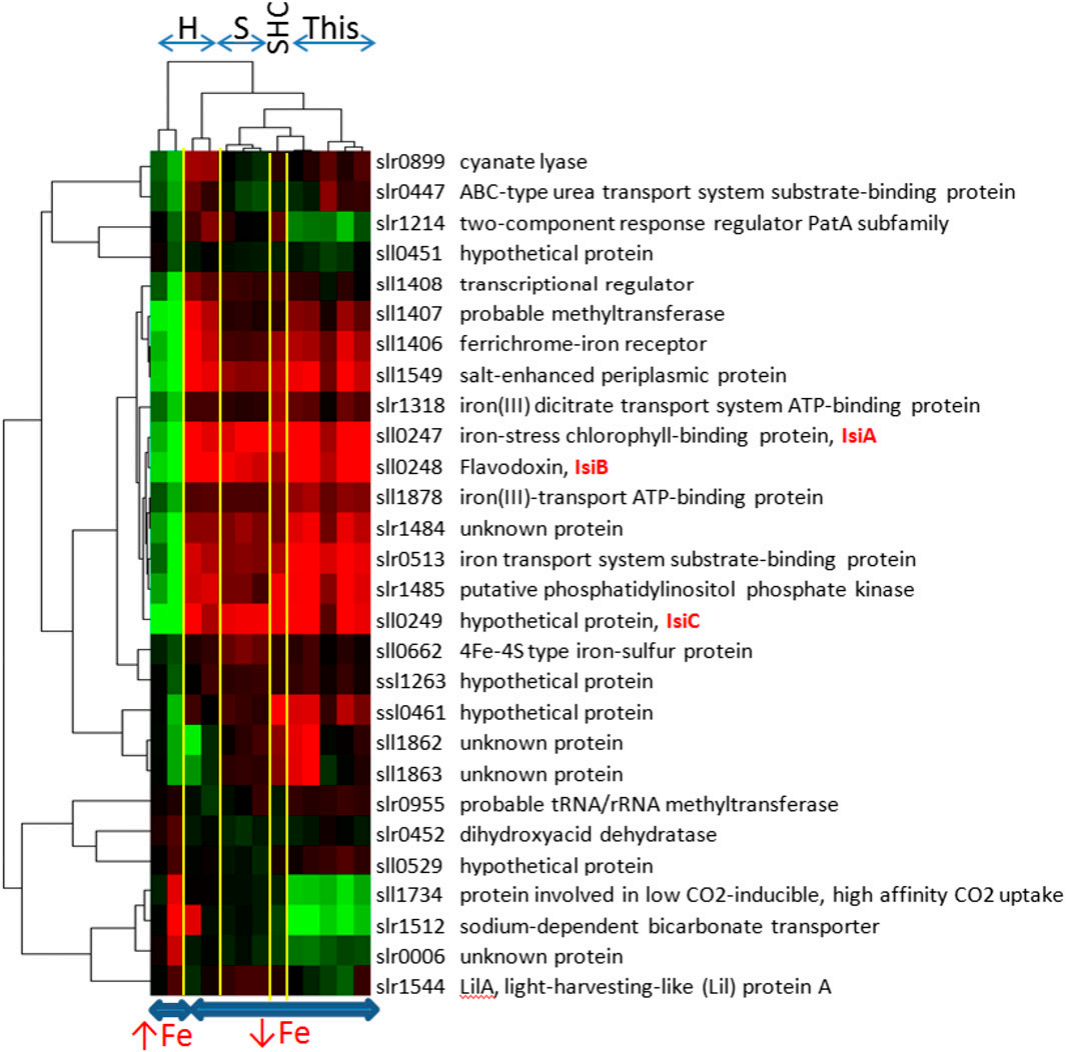
Heatmap of expression changes for the core set of iron response genes. The 28 genes detected as differentially expressed (absolute log_2_FC ≥ 0.5 and *p* < 0.01) in all the experiments analyzed here were hierarchically clustered based on normalized expression values. As reference, expression for the sample grown under normal conditions was taken. Columns represent the different experiments under comparison with following abbreviations: H (Houot *et al.* 2007), S (Singh *et al.* 2003), SHC (Shcolnick *et al.* 2009), and This (the study presented here). The first two columns correspond to expression analysis performed on cultures grown under excess iron while the rest was subjected to iron-limiting conditions (see supporting information, *Extended Material and Methods*, for details). The level of differential expression is represented by a green to red color gradient, corresponding to genes negatively (green) or positively (red) expressed in relation to the control sample.

In total, 375 genes (27.4% of the total genes) were differentially expressed in at least two of the four studies analyzed. A much smaller number (28 genes; 2% of the total genes) was detected consistently as differentially expressed in all the studies (Table S5). The highly robust regulatory response of these genes was further examined by hierarchical clustering across the experiments (Figure 4). Of these 28 genes, 21 were upregulated and only 3 genes were downregulated under iron-limiting conditions, whereas 4 genes showed different expression trends between the compared experiments. The upregulated genes are the co-transcribed *isiA*, *isiB*, *sll0249* (Kojima *et al.* 2006), and the adjacent gene *ssl0461*, as well as the co-localized genes *sll1406*, *sll1407*, and *sll1408* involved in the transport of ferric siderophores (Katoh *et al.* 2001). Other genes such as *sll1878* (FutC protein), *slr0513* (FutA2 protein), and *slr1318* (FecE protein) with demonstrated roles in iron transport (Katoh *et al.* 2001) also belong to this core set of upregulated genes. Nine of the genes differentially expressed in all experiments encode proteins of unknown function. The set of consistently downregulated genes comprised *sll1734* (*cupA*), encoding the CO_2_ uptake subunit of the NDH-1 complex; *slr1512* (*sbtA*), encoding the high affinity Na-dependent bicarbonate uptake system; and *slr0006*, encoding a protein of unknown function that is upregulated under CO_2_-limiting conditions (Carmel *et al.* 2011).

### Comparative clustering of differentially expressed protein-coding and non-coding genes

**Figure 5:**
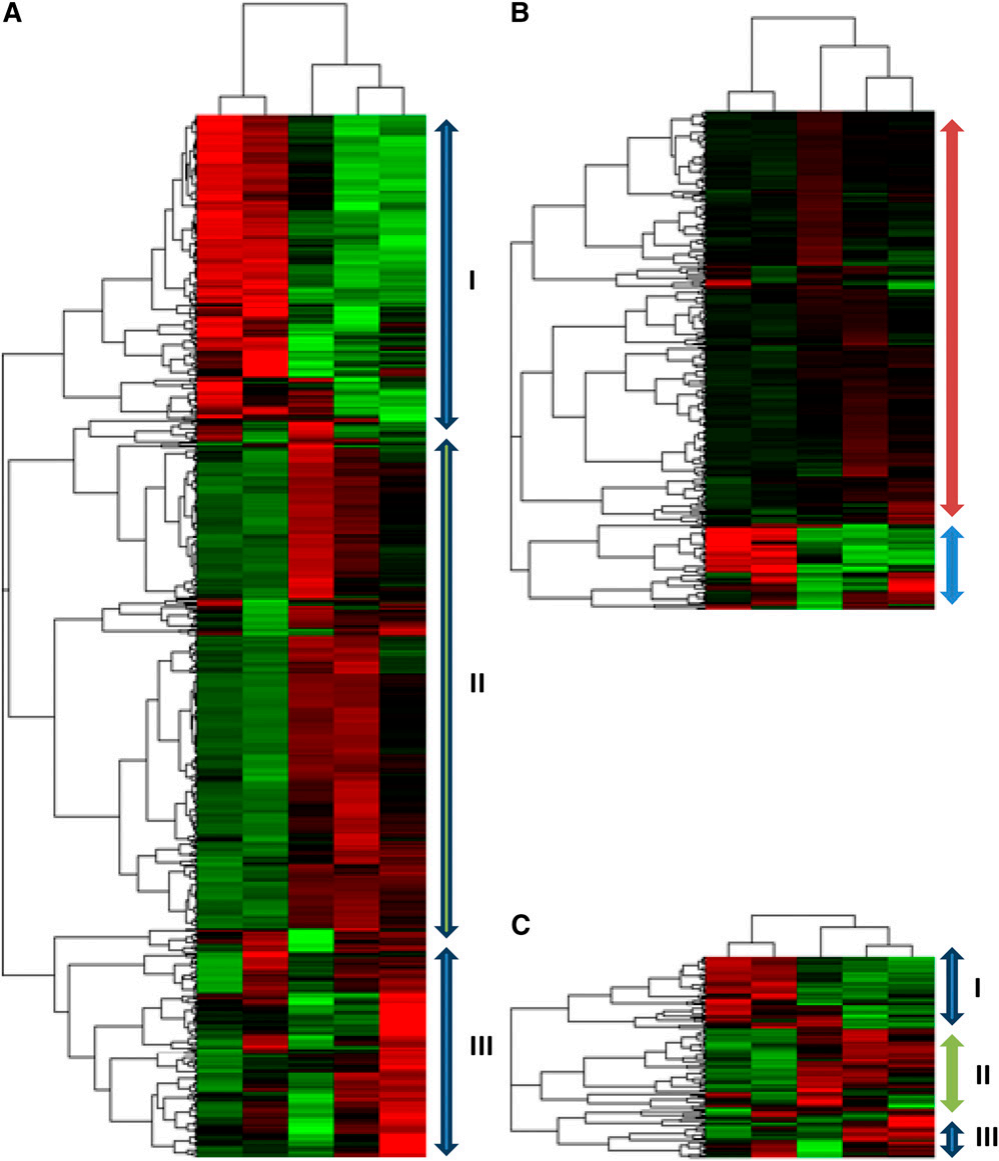
Heatmap of expression changes. Differentially expressed transcripts (|log_2_(FC)| ≥ 1.0 and q < 0.05 in at least one of the time points) were hierarchically clustered: (A) Protein-coding genes, (B) asRNAs, and (C) sRNAs. Rows represent genes, and columns represent the different time points. Red represents an increase in transcript levels, and green represents a decrease in transcript levels upon depletion of iron. No differences in expression correspond to black squares. The associated dendogram shows the relationships between the genes based on their expression, with the length of the branches representing the degree of similarity in expression.

An important feature of the microarray platform utilized in our study is the coverage of non-coding *Synechocystis* genes. To compare their overall transcriptional response to iron deprivation, differentially expressed protein-coding genes, asRNAs, and sRNAs were hierarchically clustered based on their expression changes. The expression profiles of protein-coding genes can be divided into three main clusters (Figure 5A). Cluster I includes mainly genes whose expression increased upon the addition of DFB and slowly decreased during the experimental time. Cluster II contains genes whose expression initially decreased and gradually increased after the addition of DFB. Cluster III had no clear expression profile. Cluster II is the largest of the three clusters, containing approximately the same number of genes as the other two clusters combined. For the 307 differentially expressed asRNAs, the overall clustering structure is somewhat different. A large group of asRNAs displayed small expression changes, whereas the expression of 30 asRNAs increased strongly during the first 12 hr and quickly decreased thereafter (Figure 5B and Table S6). This latter group included (i) IsrR, the asRNA to *isiA*; (ii) *sll0027-as1*, the asRNA to *ndhD4* encoding the NDH subunit D4; (iii) *sll1867-as1*, the asRNA to *psbA3*; (iv) slr1878-as1, the asRNA to *cpcE*, encoding the phycocyanin alpha-subunit phycocyanobilin lyase; and (v) slr2143-as1, the asRNA to *cefD*, encoding L-cysteine/cystine lyase. Finally, the clustering obtained for the 125 putative sRNAs resembled the clustering of the protein-coding genes (Figure 5C). Three main clusters were also observed in this group, two of which (II and III) included upregulated sRNAs at different time points after the addition of DFB, whereas the 45 sRNAs in cluster I decreased in abundance along the measured time (Table S7).

### Antisense RNAs can be grouped in distinct classes based on their co-expression with complementary genes

Of the 1939 probes detecting asRNA, 307 (15.8%) showed differential expression under iron-limiting conditions. One fifth of them (N = 62) were complementary to genes, which were also differentially expressed (Table 3). For the vast majority of these asRNA (59 out of 62), the median expression decreased during the experiment. In contrast, their complementary sense genes displayed a more balanced distribution of expression changes with 30 upregulated genes and 32 downregulated genes. The 3 asRNAs with a positive differential expression are complementary to genes downregulated during iron-limiting conditions; *i.e. sll0217* encoding the flavodiiron protein Flv4; *slr0144*, the first gene of Pap operon that includes 9 genes related to the assembly of photosystem II (Wegener *et al.* 2008); and *sll1723* encoding a group I glycosyltransferase homolog.

**Table 3:** asRNA-mRNAs pairs in which both asRNA and sense gene are differentially expressed

		Log_2_(FC)						
Gene ID	Description	3 hr	12 hr	24 hr	48 hr	72 hr	*q*	*r*
sll7029	Hypothetical protein	−1.13	−1.34	0.63	0.16	−0.40	3.20∙10^−7^	0.99
sll7029-as1		−1.38	−1.52	−0.56	−0.85	−1.13	2.42∙10^−6^	
sll0174	Hypothetical protein	−0.84	−1.06	−0.09	−0.06	−0.52	1.04∙10^−4^	0.99
sll0174-as1		−1.28	−1.63	−0.10	−0.01	−0.58	9.62∙10^−5^	
sll1864	Probable chloride channel protein	−2.02	−1.48	−1.50	0.17	−0.23	1.24∙10^−5^	0.99
NC5-0-x		−1.43	−0.79	−1.09	0.87	0.31	6.51∙10^−5^	
slr0304	Hypothetical protein	−1.44	−1.57	−0.53	−0.38	−0.68	4.90∙10^−7^	0.99
slr0304-as1		−1.46	−1.53	−0.62	−0.60	−0.90	4.25∙10^−6^	
slr0593	cAMP binding membrane protein	−1.32	−1.35	−0.48	−0.02	−0.60	1.76∙10^−6^	0.99
slr0593-as1		−2.09	−1.95	−0.94	0.04	−1.09	9.62∙10^−4^	
sll0640	Probable sodium/sulfate symporter	−1.20	−1.24	−0.28	−0.08	−0.84	1.18∙10^−7^	0.99
sll0640-as4		−1.91	−1.99	−0.32	−0.09	−0.98	4.21∙10^−5^	
slr1293	Similar to phytoene dehydrogenase	−1.27	−1.65	0.16	0.36	−0.16	2.78∙10^−8^	0.98
slr1293-as1		−1.21	−1.27	−0.40	−0.22	−0.70	8.39∙10^−6^	
sll0095	Hypothetical protein	−1.70	−1.60	0.12	0.17	−0.60	5.88∙10^−5^	0.98
sll0095-as1		−1.34	−1.28	0.19	−0.17	−0.46	2.31∙10^−6^	
slr0415	Na^+^/H+ antiporter	−1.02	−1.15	−0.04	0.10	−0.46	1.65∙10^−4^	0.97
slr0415-as3		−1.49	−1.35	−0.63	−0.62	−1.07	5.37∙10^−7^	
slr0727	Unknown protein	−1.32	−1.37	0.34	0.01	−0.42	1.13∙10^−5^	0.97
slr0727-as1		−1.38	−1.50	−0.05	0.17	−0.62	2.60∙10^−4^	
sll1276	ATP-binding protein of ABC transporter	−0.68	−0.79	0.98	1.09	0.65	1.74∙10^−4^	0.97
sll1276-as2		−1.04	−1.08	0.43	0.38	−0.34	1.21∙10^−5^	
slr1944	Periplasmic protein, function unknown	−1.10	−1.20	0.62	0.18	−0.41	3.68∙10^−7^	0.94
slr1944-as2		−1.57	−1.69	−0.35	−0.10	−0.81	3.45∙10^−5^	
slr0488	Virulence factor MviN homolog	−1.64	−1.67	−0.43	−0.29	−1.37	8.68∙10^−7^	0.93
slr0488-as1		−1.21	−1.14	0.38	0.25	−0.27	3.16∙10^−4^	
slr1462	Hypothetical protein	−1.27	−0.99	0.10	−0.09	−0.30	4.23∙10^−6^	0.93
slr1462-as2		−1.36	−1.38	−0.43	−0.08	−0.54	7.26∙10^−6^	
slr1529	Nitrogen assimilation regulatory protein	−0.74	−1.17	−0.07	−0.35	−0.67	3.29∙10^−6^	0.92
slr1529-as1		−1.73	−1.73	0.22	−0.18	−0.89	1.16∙10^−4^	
slr1691	gln-Dependent NAD(+) synthetase	−1.25	−1.29	−0.21	0.17	−0.60	4.71∙10^−8^	0.92
slr1691-as2		−1.08	−0.94	−0.51	−0.18	−0.94	3.00∙10^−5^	
slr1968	Unknown protein	−2.23	−2.23	−0.66	−0.50	−1.43	4.75∙10^−7^	0.89
slr1968-as3		−1.21	−1.07	−0.06	−0.17	−0.11	4.35∙10^−6^	
slr1403	Unknown protein	−1.09	−1.18	0.37	0.04	−0.36	2.08∙10^−6^	0.87
slr1403-as6		−1.55	−1.52	−0.62	−0.01	−0.78	1.06∙10^−4^	
slr1254	Phytoene dehydrogenase	0.25	−0.62	1.21	0.71	0.46	1.61∙10^−4^	0.86
slr1254-as1		−1.16	−1.07	0.28	−0.04	−0.51	1.38∙10^−5^	
slr0359	Hypothetical protein	−1.36	−1.05	−0.50	−0.03	−0.37	6.91∙10^−6^	0.81
slr0359-as2		−1.09	−1.11	0.07	0.03	−0.72	6.65∙10^−4^	
sll1200	Hypothetical protein	−1.17	−1.24	−0.54	−0.51	−0.93	1.77∙10^−5^	0.74
sll1200-as2		−1.20	−1.12	−0.34	−0.67	−0.36	5.43∙10^−5^	
sll1612	Folylpolyglutamate synthase	−1.31	−0.38	−0.02	−0.34	−0.67	0.007	0.74
sll1612-as1		−2.23	−1.92	−0.75	−0.52	−1.25	4.41∙10^−4^	
ssr1558	Hypothetical protein	−1.00	−0.70	0.21	0.31	0.08	0.003	0.66
ssr1558-as1		−0.24	−0.35	1.05	0.31	−0.20	1.19∙10^−4^	
sll1515	gln Synthetase inactivating factor IF17	−2.63	−2.64	−2.66	−1.32	−1.13	7.79∙10^−10^	0.54
sll1515-as6		−1.19	−1.02	−0.07	−0.14	−0.31	0.004	
sll0898	Hypothetical protein	0.44	0.67	0.65	0.49	1.12	2.84∙10^−5^	0.51
sll0898-as1		−2.31	−1.35	−1.70	−0.71	−0.79	9.93∙10^−8^	
slr1704	Hypothetical protein	−0.69	−0.76	−1.08	0.20	0.06	1.06∙10^−5^	0.49
slr1704-as2		−2.29	−2.27	−0.90	−0.29	−1.29	1.87∙10^−6^	
sll1319	Hypothetical protein	−2.12	−2.19	−1.11	−0.32	−0.91	9.84∙10^−6^	0.43
sll1319-as1		−1.19	−0.84	−1.21	−0.72	−0.09	2.45∙10^−7^	
sll0830	Elongation factor EF-G	−1.02	−0.44	−0.49	−0.10	−0.23	0.001	0.42
sll0830-as1		−1.14	−1.13	0.47	0.01	−0.82	1.53∙10^−7^	
slr2132	Phosphotransacetylase	−2.35	−2.14	0.00	−0.23	−0.85	2.58∙10^−6^	0.38
slr2132-as8		−1.04	−0.84	−1.02	−0.56	−0.71	1.70∙10^−4^	
sll1878	Iron(III)-transport ATP-binding protein	1.53	2.15	1.98	2.29	2.60	1.42∙10^−7^	0.37
sll1878-as2		−0.90	−1.10	0.66	0.73	−0.07	1.37∙10^−4^	
slr1392	Ferrous iron transport protein B	1.12	2.80	2.12	2.74	2.96	9.97∙10^−9^	0.33
slr1392-as1		−1.82	−1.96	−0.46	−0.17	−0.95	4.24∙10^−5^	
sll1206	Ferric aerobactin receptor, FhuA homolog	3.08	5.18	4.74	5.56	5.73	1.98∙10^−10^	0.25
sll1206-as1		−1.52	−1.76	−0.53	−0.86	−1.21	1.30∙10^−5^	
ssl0294	Hypothetical protein	0.39	0.73	0.33	0.84	1.16	5.59∙10^−6^	0.24
ssl0294-as1		−1.11	−1.00	−0.33	−0.25	−0.53	7.66∙10^−4^	
slr1929	Type 4 pilin-like protein	−0.02	0.71	−0.04	1.17	1.50	9.25∙10^−5^	0.01
slr1929-as1		−1.31	−1.36	0.22	−0.31	−0.60	3.16∙10^−7^	
slr0144	Hypothetical protein	−0.71	−0.19	−1.59	0.18	0.53	3.37∙10^−4^	0.001
slr0144-as1		1.50	1.29	0.90	0.96	1.02	9.61∙10^−5^	
slr0898	Ferredoxin-nitrite reductase	2.53	1.88	2.94	1.29	2.20	2.20∙10^−8^	−0.21
slr0898-as1		−1.28	−1.40	−0.23	0.12	−0.57	6.49∙10^−6^	
slr1512	Sodium-dependent bicarbonate transporter	−3.67	−4.30	−3.46	−5.54	−5.28	1.42∙10^−9^	−0.22
slr1512-as1		−1.41	−1.51	0.10	0.01	−0.74	3.60∙10^−7^	
slr0007	Probable sugar-phosphate nucleotidyltransferase	−1.07	−0.92	−0.97	−1.57	−1.62	6.41∙10^−7^	−0.31
slr0007-as1		−1.01	−1.11	−0.35	−0.48	−0.76	5.60∙10^−7^	
sll1744	50S ribosomal protein L1	0.71	0.85	0.35	0.33	1.06	3.76∙10^−7^	−0.32
rpl1-1-x		0.07	−0.16	−0.06	−1.09	−1.62	4.20∙10^−7^	
sll0518	Unknown protein	1.12	0.64	0.65	0.06	0.18	1.57∙10^−5^	−0.33
sll0518-as1		−0.82	−1.25	−0.25	−0.35	−0.76	4.61∙10^−5^	
slr0474	Regulator for phytochrome 1 (Cph1)	0.45	0.90	−0.66	1.46	1.57	3.19∙10^−8^	−0.35
slr0474-as1		−0.85	−1.32	−0.13	−0.12	−1.00	5.24∙10^−6^	
slr0993	Putative peptidase	1.21	1.02	0.83	−0.38	−0.26	3.73∙10^−5^	−0.44
slr0993-as4		−0.99	−1.04	0.30	−0.12	−0.43	3.29∙10^−7^	
slr1908	Probable porin; major outer membrane protein	1.13	1.21	0.89	0.64	0.34	2.65∙10^−7^	−0.46
slr1908-as1		−1.44	−1.26	0.28	−0.24	−0.69	1.01∙10^−6^	
slr0559	ABC transporter for natural amino acids	1.05	0.47	0.57	0.04	0.07	6.07∙10^−6^	−0.49
slr0559-as1		−1.62	−1.70	−1.09	−1.18	−1.33	2.12∙10^−6^
sll1043	Polyribonucleotide nucleotidyltransferase	0.93	1.12	0.80	0.95	1.29	4.94∙10^−7^	−0.53
sll1043-as3		−1.31	−1.30	0.54	−0.06	−0.70	4.18∙10^−7^	
sll0217	Flavoprotein	−6.26	−5.97	−5.21	−6.21	−6.10	2.38∙10^−8^	−0.56
sll0217-as2-0		0.98	1.17	0.86	1.06	1.29	4.44∙10^−7^	
slr0848	Hypothetical protein	1.09	0.96	0.59	0.26	0.27	2.99∙10^−7^	−0.58
slr0848-0-x		−0.99	−1.13	−0.30	−0.46	−0.87	1.11∙10^−5^	
slr1318	Iron(III) dicitrate transport system ATP-binding protein	0.32	1.70	1.18	1.55	1.39	5.44∙10^−8^	−0.63
slr1318-as1		−0.57	−1.11	−0.52	−0.67	−0.94	8.30∙10^−6^	
sll0374	Urea transport system ATP-binding protein	1.11	0.90	0.75	0.10	0.09	7.28∙10^−7^	−0.64
sll0374-as2		−1.05	−0.89	−0.19	−0.41	−0.45	2.11∙10^−4^	
sll1119	Hypothetical protein	1.50	0.84	0.62	0.11	0.68	3.64∙10^−7^	−0.69
sll1119-as4		−1.23	−1.36	0.17	−0.19	−0.63	7.02∙10^−7^	
slr0585	Argininosuccinate synthetase	1.65	1.39	1.03	0.44	0.76	2.25∙10^−6^	−0.74
slr0585-as3		−1.71	−1.76	0.34	−0.10	−0.78	1.11∙10^−5^	
sll0083	Phosphoheptose isomerase	1.07	0.84	0.56	−0.07	−0.02	2.09∙10^−5^	−0.78
sll0083-as1		−1.13	−1.03	−0.13	−0.20	−0.42	1.37∙10^−6^	
ssl3177	Hypothetical protein	1.05	0.93	0.76	0.14	0.55	3.01∙10^−6^	−0.78
ssl3177-as1		−1.09	−1.04	−0.01	0.02	−0.49	2.58∙10^−5^	
sll1070	Transketolase	1.59	1.22	0.92	0.67	0.76	1.00∙10^−5^	−0.81
tktA-as2-0-x		−1.36	−1.21	−0.33	−0.43	−0.89	5.16∙10^−5^	
slr1020	Sulfolipid biosynthesis protein SqdB	1.09	0.81	0.48	0.30	0.29	9.50∙10^−6^	−0.82
slr1020-as1		−1.75	−1.64	−0.46	−0.51	−1.08	1.67∙10^−4^	
sll1723	Probable glycosyltransferase	−1.80	−1.84	−0.31	0.03	−0.86	3.32∙10^−7^	−0.83
sll1723-as1		1.01	0.64	0.25	−0.23	−0.17	1.58∙10^−6^
sll1130	Unknown protein	1.22	1.06	0.59	0.31	0.48	9.54∙10^−7^	−0.89
sll1130-as1		−1.15	−1.03	−0.27	−0.06	−0.72	8.91∙10^−4^	
sll1330	Two-component system response regulator OmpR subfamily	1.09	1.20	0.42	−0.18	0.42	3.37∙10^−7^	−0.89
sll1330-as1		−1.34	−1.35	−0.41	−0.42	−0.40	1.09∙10^−4^	
ssr1375	Hypothetical protein	0.45	0.89	−1.13	−0.47	−0.07	7.81∙10^−7^	−0.92
ssr1375-as1		−0.96	−1.23	−0.42	−0.43	−0.53	5.63∙10^−6^	
slr1280	NADH dehydrogenase subunit NdhK	−0.90	−1.03	−1.27	−1.64	−1.54	2.35∙10^−5^	−0.94
slr1280-as1		−1.16	−1.17	−0.41	0.02	−0.41	1.71∙10^−5^	
slr0534	Probable transglycosylase	1.02	1.04	0.56	0.59	0.55	6.57∙10^−4^	−0.97
slr0534-as5		−1.80	−1.90	−0.52	−0.48	−0.83	2.59∙10^−7^	
sll0247	Iron-stress induced protein A, IsiA	2.52	5.99	5.53	6.14	6.15	2.19∙10^−7^	−1.00
sll0247-as2		−1.06	−7.04	−5.93	−6.61	−6.89	1.98∙10^−10^	

Log_2_ fold changes with respect to time 0 hr and the significance of differential expression (*q*-value) are shown. For each pair, the Pearson correlation coefficient *r* for the expression is also listed.

**Figure 6:**
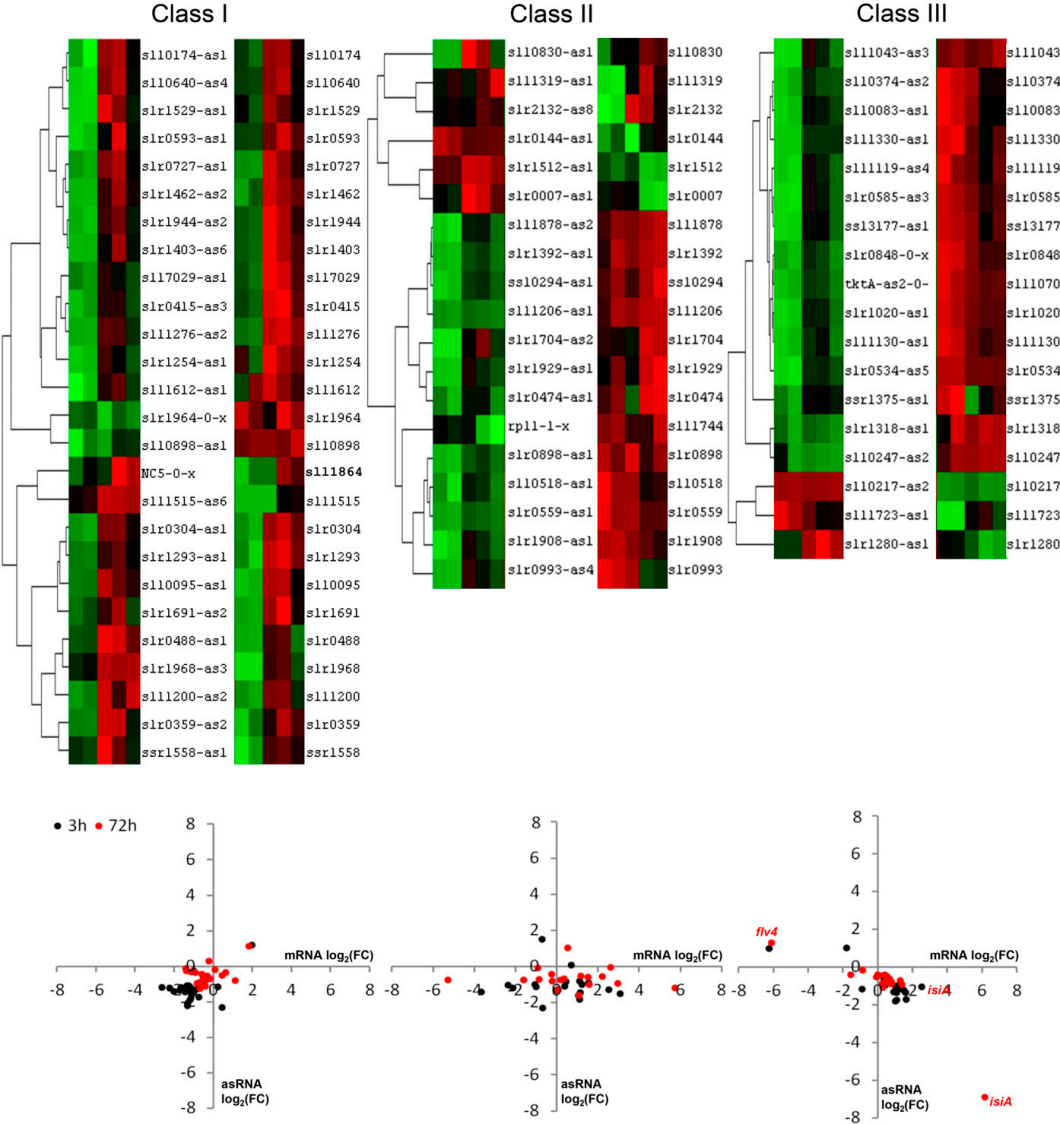
Classification of asRNA-mRNA pairs based on their expression correlation. (Top) Heatmaps obtained as result of hierarchically clustering the asRNA-mRNA pairs. Class I includes pairs with a correlation coefficient *r* > 0.5, class II includes pairs with 0.5 > *r* > −0.5, and class III includes pairs with *r* < −0.5. (Bottom) Scatter plots depicting the log_2_(FC) for the mRNA on the x-axis and the complementary asRNA on the y-axis at 3 hr (black dots) and 72 hr (red dots).

To elucidate the dynamics of antisense transcription with respect to their sense transcript for the 62 pairs, we assigned the pairs to different classes based on correlation of the expression of sense and antisense transcript (Table 3). Such tentative classification was motivated by previous findings that asRNA can modify the stability of target RNA (Georg and Hess 2011). Formation of duplex RNA resulting from mRNA-asRNA hybridization can increase RNA stability by masking cleavage sites of endoribonucleases as well as decrease RNA stability by promoting RNA degradation. Thus, changes in abundance of sense and antisense transcripts may be coupled through such mechanisms. In total, three classes were defined: Class I includes all pairs, for which sense and antisense transcript abundance were strongly correlated (*i.e.* with a Spearman correlation coefficient *r*_S_ > 0.5). Class II includes pairs with modest or no (anti)correlation between sense and antisense (−0.5 ≤ *r*_S_ ≤ 0.5), whereas class III comprises pairs for which the sense-antisense pairs are strongly anticorrelated (*r*_S_ < −0.5). Figure 6 compares the characteristic expression changes of sense and antisense transcript during the course of the experiment for the three classes. Interestingly, we found that class I pairs generally comprised asRNAs whose expression (based on microarray signal intensity) was higher than those of their complementary mRNAs. This was in striking contrast to sense-antisense pairs from class III for which the mRNA transcripts displayed higher levels than their corresponding asRNA transcripts (*p*-value = 0.0014).

In class I, the 26 pairs include *gifB*/*as-gifB*, *folC* (*sll1612*)/*as-folC*, *nadE* (*slr1691*)/*as-nadE*, and *nrtX* (*slr1529*)/*as-nrtX*, all of them related to nitrogen metabolism. Here, expression of asRNA might promote stability of their target RNA. Class II comprises 19 pairs, *e.g. fhuA* (*sll1206*)*/as-fhuA*, *feoB (slr1392)/as-feoB*, and *futC/as-futC*, whose sense genes code for components of iron transporters that become highly expressed under iron-limiting conditions. As only modest correlation with asRNA existed, asRNA might rather serve to fine-tune the base levels of mRNA abundance. Finally, class III comprises 18 asRNAs, including IsrR and As1_flv4. Upon iron deprivation, IsrR levels were reduced while the *isiA* transcript accumulated in a highly anticorrelated fashion (*r*_S_ = −0.9, *p*-value = 2.49 × 10^−5^). An opposite behavior was found for the pair *flv4/as1_flv4*. Here, As1_flv4 accumulates over time, whereas a decrease in the transcript levels for flavodiiron protein Flv4 was found. The relevance of the observed reciprocal expression has already been demonstrated for these two asRNAs in previous work, indicating their crucial role in fine-tuning of target RNA expression (Dühring *et al.* 2006; Eisenhut *et al.* 2012). More precisely, the asRNA As1_flv4 establishes a transient threshold for *flv4* expression in the early phase after a change in inorganic carbon supply, preventing premature synthesis of the proteins from the *flv4-2* operon. The expression of As1_flv4 itself is tightly regulated at transcriptional level by the AbrB-like transcription factor Sll0822 (Eisenhut *et al.* 2012).

Besides their co-expression, we also investigated the locations of asRNAs with respect to the corresponding full-length mRNA. Interestingly, we detected that asRNAs in class I tended to be located toward the 3′UTR of the complementary transcript, with 17 of the 25 asRNAs (68%) closer to the 3′UTR than to the 5′UTR. For the asRNA in classes II and III, no such tendency was observed.

### Potential *trans*-acting sRNAs involved in iron response and their predicted targets

**Figure 7:**
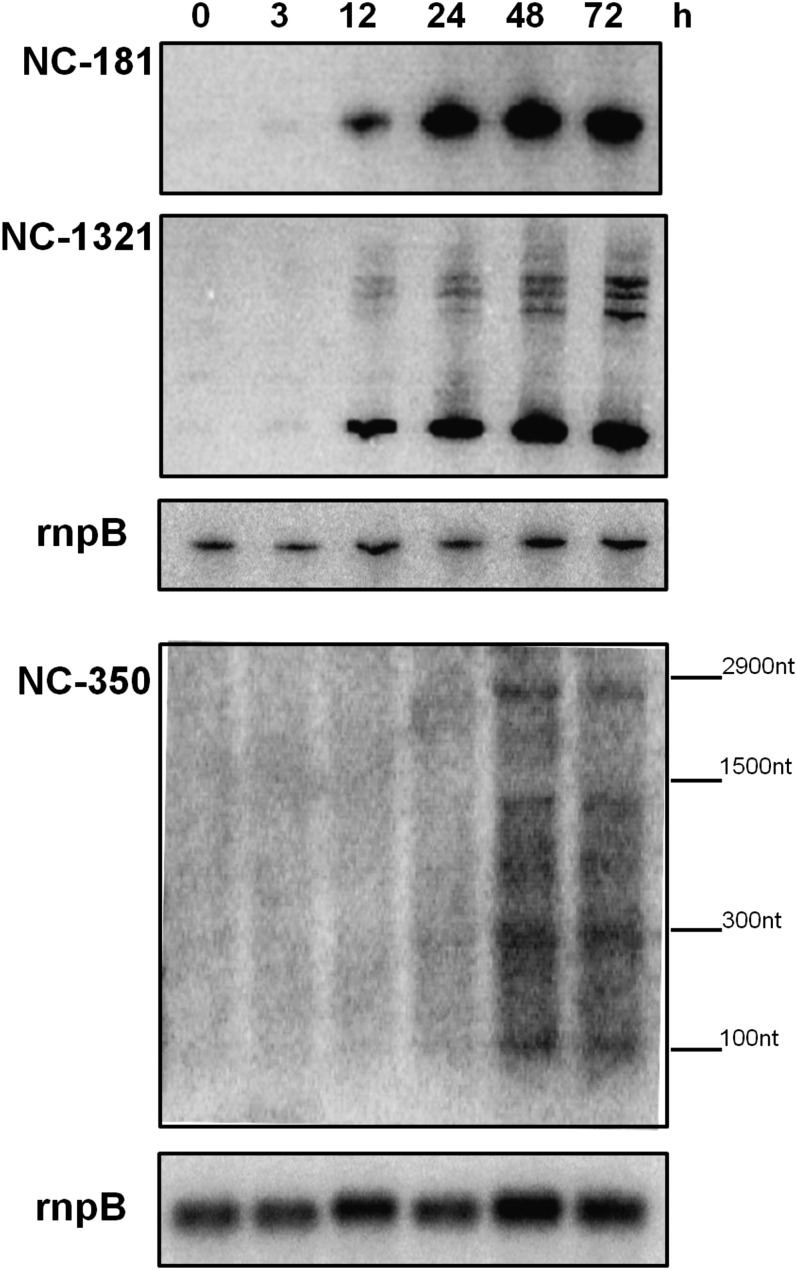
Northern blots confirming sRNAs inducibility by iron depletion over a period of 72 hr. Equal loading was verified by a control hybridization for the RNase P RNA (rnpB). For NC-350, the length of the selected marker bands is given to the right.

Small RNAs were among the most highly expressed transcripts in the control condition (Table 1). Remarkably, the sRNAs were also significantly enriched among differentially expressed genes (N = 125; FDR = 6.4 × 10^−11^). The majority were downregulated upon the addition of DFB (Table S7). Our interest was to identify sRNAs with a potential role in iron homeostasis. For this, we utilized *isiA* as a marker gene for the transcriptional response to iron limitation and calculated the correlation coefficient between *isiA* and differentially expressed sRNAs. Setting a correlation coefficient of 0.7 as a minimum threshold, we identified four ncRNAs whose expression over time correlated strongly with *isiA* (Table 4). NC-181 showed the highest correlation (*r*_S_ = 0.98), followed by NC-1321 (*r*_S_ = 0.90), NC-265 (*r*_S_ = 0.74), and NC-350 (*r*_S_ = 0.70). Of these four sRNAs, NC-181 was the most upregulated during iron-limiting conditions (log_2_FC = 3.71 at 48 h). The inducibility of several of these sRNAs by iron stress was verified by Northern analysis (Figure 7). The blots showed that NC-181 and NC-1321 accumulated as distinct sRNAs, about 70 and 80 nucleotides in length, with virtually identical kinetics. A distinct signal was obtained for NC-350, originating from the *slr0550-slr0551* intergenic spacer. Whereas the two low-molecular weight signals confirmed the presence of short transcripts, about 100 and 300 nucleotides in length, the longer signals suggest read-through and co-transcription with gene *slr0551* over its full length (Figure 7).

**Table 4:** sRNAs whose expressions are highly correlated with isiA expression over the time series

Upregulated, Log_2_(FC)						
Gene ID	3 hr	12 hr	24 hr	48 hr	72 hr	*q*
NC-181	1.52	3.31	3.52	3.72	3.74	5.09∙10^−8^
NC-1321	0.40	1.35	1.02	1.53	1.66	5.42∙10^−6^
NC-265	0.27	0.48	0.48	1.39	1.51	0.002
NC-350	0.36	1.04	0.41	0.82	1.49	5.13∙10^−7^
Downregulated, Log_2_(FC)						
Gene ID	3 hr	12 hr	24 hr	48 hr	72 hr	*q*
NC-117	−0.07	−0.90	−1.14	−0.50	−0.95	5.56∙10^−4^
NC-1606	−0.13	−1.05	−0.43	−1.06	−1.10	1.03∙10^−5^
NC-981	−0.40	−1.10	−0.48	−0.75	−0.57	6.84∙10^−6^
NC-1637	0.20	−0.59	−0.54	−1.12	−1.13	6.60∙10^−7^
NC-29	−0.69	−1.10	−1.79	−0.65	−1.04	7.26∙10^−7^
NC-954	−0.22	−0.74	−0.37	−1.37	−1.20	4.43∙10^−4^

Spearman correlation coefficient larger than 0.7 in absolute value.

Because most bacterial sRNAs act through sequence-specific binding to regions close to the ribosome binding site of mRNAs, we sought to predict potential RNA targets using a window of 250 nucleotides around the respective start codons (150 upstream and 100 downstream) and employing the IntaRNA algorithm (Busch *et al.* 2008). Setting a free-energy cut-off of −10 kcal/mol, the number of predicted targets was 724 for NC-181, 100 for NC-1321, 772 for NC-265, and 539 for NC-350, indicating many potential false-positives. To reduce these numbers, the observed expression data were utilized. We reasoned that potential targets should show inverse correlation with sRNA levels, as most bacterial sRNAs studied so far are negative regulators of gene expression. Defining a threshold of *r*_S_ < −0.5 for inverse correlation, the list of candidates was drastically reduced to 45 potential targets for NC-181, 6 for NC-1321, 29 for NC-265, and 23 for NC-350 (Table S8). To assess whether sRNAs target specific functions, we carried out functional enrichment analyses. No significantly enriched function was detected for predicted targets of NC-1321 or NC-265. However, several functional categories were found to be enriched in potential targets of NC-181 and NC-350. The 45 potential targets of NC-181 tended to be overrepresented in the set of genes associated with “photosynthesis and respiration” (FDR = 3.01 × 10^−6^), mainly due to the presence of genes encoding subunits of ATP synthase (3 genes, FDR = 0.02) and NADH dehydrogenase (3 genes, FDR = 0.10). Equally, the 23 predicted targets for NC-350 were significantly enriched in genes linked to “energy metabolism” (FDR = 0.03); “fatty acid, phospholipid, and sterol metabolism” (FDR = 0.03); “photosynthesis and respiration” (FDR = 0.06); and “transport and binding proteins” (FDR = 0.05).

We also searched for sRNAs, whose expression was anticorrelated with *isiA*. In this case, we detected six transcripts (NC-117, NC-1606, NC-981, NC-1637, NC-29, and NC-954) that had a correlation coefficient of lower than −0.7 with *isiA*. All of the detected sRNAs were significantly downregulated with respect to the control sample and, consequently, correlated with genes such as *Flv2*, *Flv4* and *petF* (*ssl0020*, encoding ferredoxin I), which were also downregulated during iron-limiting conditions. Such strong correlation with key players in iron homeostasis might be indicative for a functional role for this set of sRNAs.

## Discussion

Iron is an essential element for all organisms. In particular, cyanobacteria require sufficient iron to synthesize the photosynthetic machinery. However, the intracellular iron concentration has to be tightly regulated, as an excess of Fe^2+^ is toxic. To study the details of the underlying regulatory mechanism, we measured the expression upon iron limitation using a comprehensive microarray platform. Following the addition of DFB and the concomitant reduction of available iron, the expression of many protein-coding genes, asRNAs, and sRNAs was significantly affected. Complementary types of enrichment analysis pointed to the activation or repression of various processes and pathways. As expected, genes encoding proteins involved in iron transport, as well as other genes involved in the adaptation to iron depletion, such as the *isiAB* operon, were highly induced during the whole experiment. The observed transient accumulation of several transcripts encoding photosynthetic proteins was somewhat surprising. In particular, eight genes (including *isiA*) encoding subunits of the two photosystems were detected as differentially expressed (Table S4 and Figure S4). Among them, two genes, *psaL* and *psaK1*, associated with photosystem I accumulated during the first 12 hr (Table S4). Both corresponding proteins, PsaK1 and PsaL, are incorporated in the late steps of photosystem I assembly (Dühring *et al.* 2007). Notably, PsaL has been shown to be essential in the formation of trimeric photosystem I complexes and also to facilitate the correct assembly of IsiA to the trimeric photosystem I (Kouřil *et al.* 2005). A similar induction during the first 12 hr was observed for *psaB* encoding one of the photosystem I core subunits. At later time points, *psaB* transcripts levels were strongly reduced, in agreement with previous reports (Table S4). Components of photosystem II (*psbO*, *psbZ*, *psbI*, and *psb28*) also showed initial upregulation during the first 12 hr. PsbI and Psb28 both seem to perform a role in the assembly of photosystem II (Nixon *et al.* 2010). Interestingly, a mutant lacking *psb28* had impaired accumulation of both CP47 and the photosystem I core subunits, PsaA and PsaB. This limitation appeared to be linked to the synthesis and incorporation rate of chlorophyll into these proteins during assembly of both photosystems (Dobáková *et al.* 2009). Because the N-terminal domains of PsaA and PsaB, CP47, CP43, as well as IsiA, belong to the CP43-like family of chlorophyll-binding proteins, it is tempting to speculate that the observed upregulation of *psb28* during the first 12 hr might be due to its participation in the assembly of IsiA.

### Coordinated adaption of molecular processes and pathways to iron-limiting conditions

**Figure 8:**
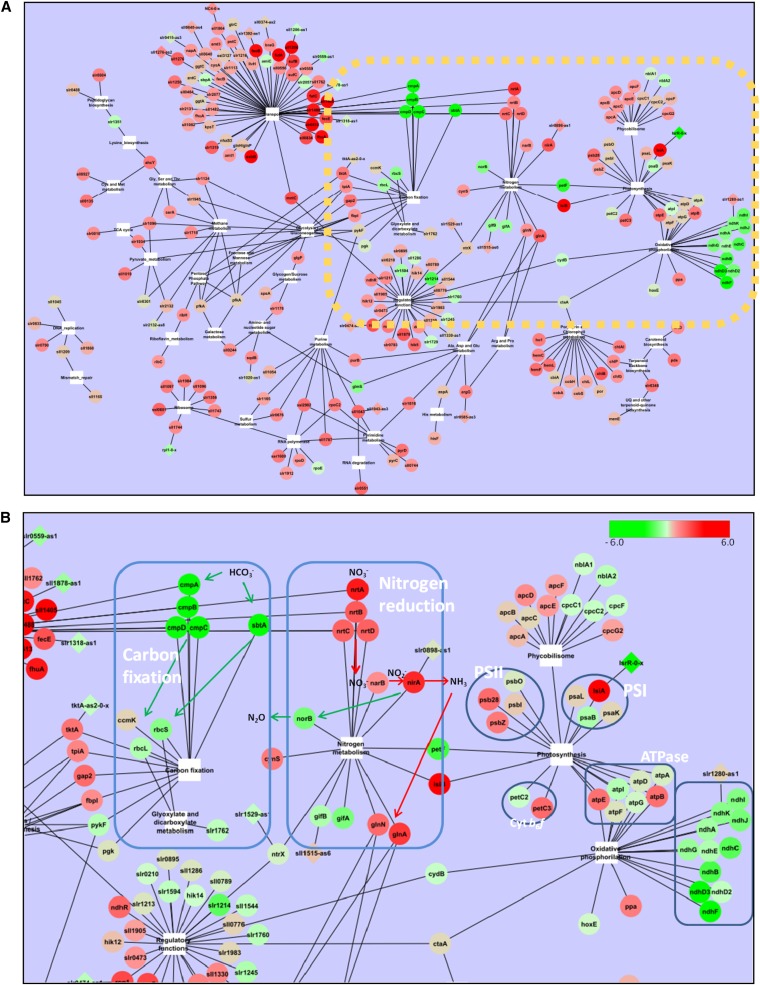
Expression data mapped onto the KEGG pathway network. Protein-coding genes (circular nodes) differentially expressed under iron-limiting conditions were linked to their associated KEGG pathways. Genes are colored based on the expression level after 48 hr from the addition of DFB according the displayed color bar. Antisense RNAs (diamond shapes) differentially expressed were linked to the corresponding complementary gene. The whole network is displayed in (A). The yellow rectangle marks the part that is enlarged in (B) and includes carbon fixation, nitrogen reduction, and the photosynthesis systems. HCO_3_^−^, NO_3_^−^, NO_2_^−^, N_2_O, and NH_3_ metabolites are shown in (B) to highlight pathways discussed in the main text.

As iron-containing proteins serve diverse functions, any limitation of iron will affect various processes and pathways. For cyanobacteria, it is crucial to rapidly adapt the different molecular mechanisms to changes in external conditions. Because many of these processes are inherently linked, a coordinated response is required to balance potentially opposing demands and ensure maintenance of essential functions. Indeed, we observed such coordination between different pathways when we graphically visualized the functional dependencies of differentially regulated genes. Figure 8A shows the network of protein-coding genes based on their association with the KEGG pathways. Strikingly, many genes connecting distinct pathway are differentially regulated, indicating an adaptive change in the network of processes at systems levels.

Of particular interest here is the intriguing interplay of carbon fixation, nitrogen reduction, and the photosynthesis systems (Figure 8B). Genes encoding proteins involved in nitrogen transport and assimilation were positively regulated during all measured times (Figure S6), despite their functional iron dependency (Flores *et al.* 2005). It is important to note that even when these genes were significantly induced, the magnitude of the induction decreased over the course of the experiment (Table 2). This decrease may indicate that the system was settling into a new equilibrium. In contrast to nitrogen assimilation, genes encoding proteins involved in the uptake of inorganic carbon, such as CmpABCD, SbtAB, or subunits of the NDH complex 3 (Table 2), were negatively regulated (Figure 8B). Notably, these genes, which displayed a downregulation in our study, also belong to the core set of only 28 genes that were differentially expressed in all independent microarray experiments monitoring iron homeostasis in *Synechocystis* (Table S5). Similar downregulation of genes involved in carbon fixation was reported by Shcolnick *et al.* (2009) following the induction of iron stress by DFB, and it can also be observed in the data by Houot *et al.* (2007), which had triggered iron stress without the use of a chelator.

A coordinated inverse regulation between genes involved in the metabolism of carbon and nitrogen has been reported before (Muro-Pastor *et al.* 2001; Osanai *et al.* 2006). Since both processes, nitrogen reduction and carbon fixation, depend on iron-containing enzymes, a coordinated regulation might reflect the prioritization of one pathway over the other depending on the environmental conditions. The inference of such coordinated regulation is supported by several other observations. For instance, a mutant lacking the metal-regulated gene A (*mrgA*), encoding a protein that exerts a role in the mobilization of iron from bacterioferritins (Shcolnick *et al.* 2007), showed a similar downregulation of genes involved in carbon fixation and upregulation of nitrite/nitrate transporters compared with wild-type strain even under normal conditions. The levels of expression of these genes is probably due to the impaired mobilization of iron from the cell reservoirs and thus explains why the *mrgA* knockout presents transcript levels resembling those of iron-stressed cells (Shcolnick *et al.* 2009). A further link between carbon fixation and iron limitation is likely provided by the *sll0217-sll0218-sll0219* operon, which is involved in acclimation to low-carbon, high-light stress and whose expression under such stress conditions reduced photosystem II damage (Hihara *et al.* 2001; Zhang *et al.* 2009). In iron-limiting conditions, photosystem II protective mechanisms can be expected to be especially important due to the observed reduction in the number of photosystem I complexes. Surprisingly, however, it was downregulated in our study. Thus, the repression of these genes might be caused either because of their iron content or because of their association to carbon fixation. This latter affirmation is supported by the detected upregulation of *ndhR*, a negative regulator of genes involved the CO_2_ uptake system of *Synechocystis* (Wang *et al.* 2004), including the *sll0217-sll0218-sll0219* operon (Eisenhut *et al.* 2012), as well as recent findings indicating that Flv2 and Flv4 might function as an alternative photosystem II electron acceptor to plastoquinones, directing electrons to enzymes involved in carbon fixation (Zhang *et al.* 2012). In summary, several independent lines of investigation suggest a direct effect of iron availability on iron-dependent pathways dedicated to carbon and nitrogen fixation.

### Inference of RNA regulators and remodeling of the transcriptome

Knowledge about the abundance of ncRNAs and the different ways in which they can modify the expression of their regulatory targets has increased dramatically in recent years for all kingdoms of life (Barrandon *et al.* 2008; Storz 2002) and has altered our view of the composition of the genome for bacteria (Georg and Hess 2011). The function of the vast majority of the more than 300 recently described sRNAs originating from intergenic regions in *Synechocystis* and of the asRNAs against more than 800 different genes is still unknown (Mitschke *et al.* 2011a). In the context of iron homeostasis, recent studies discovered asRNAs against key genes such as *fur*A and *isi*A, and they revealed their role as regulatory elements in *Anabaena* and *Synechocystis*. A prime example for the action of asRNA is given by IsrR, which contributes to the posttranscriptional control of chlorophyll-binding protein IsiA (Dühring *et al.* 2006). This regulation was also clearly reflected in our experiment, in which a strong negative correlation between both transcripts was observed. Strikingly, the expression of several other asRNAs and their corresponding sense-RNAs were also affected by the lack of iron (Table 3). The use of asRNAs as regulatory factors might offer multiple advantages, such as fast responses to environmental stimuli and the efficient silencing of gene expression from leaky promoters. The three classes of asRNAs/mRNAs pairs displayed in Figure 6 appear to be characterized by different relative levels of expression. In class I, asRNAs tend to be more expressed than the protein-coding genes, whereas in class III, the reversed relation was observed. How these patterns influence protein abundance remains to be determined in follow-up studies.

We also detected differential expression for various sRNAs (Table S6). Regulatory sRNAs involved in the regulation of iron homeostasis are known from several different groups of bacteria. The best-studied representative is the enterobacterial sRNA RyhB (Wassarman *et al.* 2001), which regulates more than 20 different proteins when iron becomes limiting (Masse and Gottesman 2002; Salvail and Masse 2012). By analogy to the regulation of *ryhB* expression by Fur in *E. coli*, we might expect that a functional RyhB homolog in *Synechocystis* will cluster with genes such as *isiA* or with genes encoding iron transporters. Three such co-regulated sRNAs, NC-181, NC-1321, and NC-350, whose expression is highly correlated with that of *isiA*, were identified, and their iron-dependent regulation was confirmed by Northern analyses. For NC-350, Northern analyses suggested that two of the four NC-350 transcripts extend the coding sequence of gene *slr0551* and therefore might constitute its 5′-UTR rather than be a *bona fide* sRNA (Figure 7). With a length of 456 nucleotides, this is one of the longest 5′-UTRs in *Synechocystis*, providing an extensive target for regulatory factors. Interestingly, the protein encoded by *slr0551* is an RNase J homolog. RNase J was originally discovered in *Bacillus subtilis* (Even *et al.* 2005). Although it is absent in *E. coli*, it is typically present in cyanobacteria and Archaea (Clouet-d’Orval *et al.* 2010; Hasenohrl *et al.* 2011). There is no information on the function of RNase J in cyanobacteria. However, it does play a major role in the evolutionarily related plant chloroplasts, in which it compensates for inefficient transcription termination by removal of antisense RNA (Sharwood *et al.* 2011). Since sense-antisense RNA interactions are at the heart of the iron stress response in *Synechocystis*, the observed activation of RNase J expression suggests this enzyme plays a functional role in remodeling the cyanobacterial transcriptome under iron stress. The very long 5′UTR may play a regulatory or autoregulatory function, analogous to the 361-nucleotide 5′ UTR of the RNase E mRNA in *E. coli* (Diwa *et al.* 2000). It should be stressed that at present we cannot exclude a separate sRNA function for the two shorter NC-350 transcripts.

A clearer picture was found for NC-181 and NC-1321, for which we detected accumulation of distinct sRNAs, 70 and 80 nucleotides in length. Thus, both upregulated sRNAs and possibly also NC-265 are prime candidates for regulators of gene expression under iron stress on a posttranscriptional level. A putative regulatory role is further supported by the analysis of predicted target genes. For NC-181, superoxide dismutase B (*sodB*) ranks relatively high on the list of targets (predicted energy -11.42 kcal/mol; Table S8). Notably, this gene is also regulated by RyhB in *E.coli* and by PrrF1 and Prrf2 in *Pseudomonas aeruginosa* (Afonyushkin *et al.* 2005; Masse *et al.* 2005; Wilderman *et al.* 2004). Moreover, we noticed that, based on sequence similarity, possible homologs of NC-181 might exist in widely different groups of cyanobacteria, including several other unicellular as well as filamentous species (data not shown).

### Conclusions

Most previous work on bacterial sRNA regulators involved in the iron stress regulatory networks has focused on human pathogens. However, for cyanobacteria as photosynthetic organisms equipped with versatile iron-dependent proteins, the regulation of iron homeostasis might be more complex. Our study provides the first time-resolved in-depth analysis of possible RNA regulators of iron homeostasis in a cyanobacterial model organism. It suggests a very complex regulatory network involving many more components at posttranscriptional level than is so far known from other bacteria. We have identified 10 sRNAs, 62 asRNAs, four 5′ UTRs, and seven intragenic elements likely to be involved in the iron regulatory network. In particular, our finding of 10 strongly iron-regulated sRNAs indicates extensive posttranscriptional regulation. Among the predicted targets of some of these sRNAs are several electron transfer chain components and proteins requiring iron as a cofactor. Future work is needed to address the specific functions of these sRNAs *in vivo*. Our identification of a small set of regulated sRNAs is an essential precondition for such experiments. As *Synechocystis* can be easily manipulated and as a large number of functional genomics and genetics tools are available, their functions can now be efficiently characterized. To facilitate follow-up studies, we included the microarray data in the CyanoEXpress database (http://cyanoexpress.sysbiolab.eu), where it can be interactively displayed and analyzed by other researchers. Furthermore, the identification of 28 protein-coding genes, detected in this and three previous microarray studies as differentially expressed, defined a core set of proteins involved in the iron-stress response. The fact that several genes involved in the assimilation of inorganic carbon are included in this core set suggests a previously unknown link between carbon and iron regulatory networks that is worth studying further. In addition, 9 of these 28 proteins are of unknown function and present promising targets for detailed functional analysis.

## Supplementary Material

Supporting Information
