# Supplementary material for: Iron Deprivation in Synechocystis: Inference of Pathways, Non-coding RNAs, and Regulatory Elements from Comprehensive Expression Profiling
Source: G3 (Bethesda). 2012 Dec 1;2(12):1475–95. doi: 10.1534/g3.112.003863 (PMC3516471; doi:10.1534/g3.112.003863)
Supplement: Supporting Information [file supp_2_12_1475__index.html]

Supporting Information 

# Iron Deprivation in *Synechocystis*: Inference of Pathways, Non-coding RNAs, and Regulatory Elements from Comprehensive Expression Profiling

## Supporting Information for Hernandez-Prieto *et al.*, 2012

**Files in this Data Supplement:**

- Supporting Information - Figures S1-S6, File S1, and Tables S1-S8 (PDF, 2.4 MB)
- Figure S1 - Minimum centroid distance for variation of FCM parameter c (PDF, 85 KB)
- Figure S2 - Example of two differentially regulated gene sets detected by PGSEA (PDF, 96 KB)
- Figure S3 - Expression of protein-coding genes with respect to the corresponding 5' UTRs and intragenic elements (PDF, 190 KB)
- Figure S4 - Genome-wide overview combining 454 reads sequencing data for each nucleotide from the (+) and (-) cDNA populations from Mitschke et al. 2010 with the microarray data (PDF, 12 MB)
- Figure S5 - Comparison of microarray experiments (PDF, 163 KB)
- Figure S6 - Functional network based on KEGG pathways (PDF, 1.8 MB)
- File S1 - Extended methods (PDF, 81 KB)
- Table S1 - List of primers used to generate single-stranded RNA probes to test gene expression under iron limiting conditions (PDF, 87 KB)
- Table S5 - Core set of genes detected as differentially expressed in all iron-stress studies compared in this article (PDF, 97 KB)
- Table S6 - List of Antisense RNAs differentially expressed (PDF, 227 KB)
- Table S7 - List of Small RNAs differentially expressed (PDF, 121 KB)
- Table S8 - List of predicted targets for the sRNAs induced during iron starvation (PDF, 136 KB)
- Table S2 - Soft clustering membership values (.xls, 539 KB)
- Table S3 - Results from EADEG (S3A-sheet 1) and GSEA (S3B-sheet 2) analysis (.xls, 58 KB)
- Table S4 - List of protein-coding genes differentially expressed (.xls, 179 KB)
