# Supplementary material for: Iron Deprivation in Synechocystis: Inference of Pathways, Non-coding RNAs, and Regulatory Elements from Comprehensive Expression Profiling
Source: G3 (Bethesda). 2012 Dec 1;2(12):1475–95. doi: 10.1534/g3.112.003863 (PMC3516471; doi:10.1534/g3.112.003863)
Supplement: Supporting Information [file supp_2.12.1475_003863SI.pdf]

SUPPORTING INFORMATION

for the article:

Iron deprivation in Synechocystis: Inference of pathways, non-coding RNAs and regulatory elements from comprehensive expression profiling

Content

**1**	**Extended Methods**	**2 SI**
	a. Estimation of optimal parameters for soft clustering	
	b. Previous *Synechocystis* microarray studies included in meta-analysis	
**2**	**Figure S1** – Minimum centroid distance for variation of FCM parameter c	**5 SI**
**3**	**Figure S2** – Example of two differentially regulated gene sets detected by PGSEA	**6 SI**
**4**	**Figure S3** – Expression of protein-coding genes with respect to the corresponding 5'UTRs and intragenic elements	**7 SI**
**5**	**Figure S4** – Genome-wide overview combining 454 reads sequencing data for each nucleotide from the (+) and (-) cDNA populations from Mitschke *et al.* 2010 with the microarray data (Figure_S4.pdf)	**9 SI**
**6**	**Figure S5** – Comparison of microarray experiments	**10 SI**
**7**	**Figure S6** – Functional network based on KEGG pathways	**11 SI**
**8**	**Table S1** – List of primers used to generate single-stranded RNA probes to test gene expression under iron limiting conditions	**14 SI**
**9**	**Table S2** – Soft clustering membership values (Table_S2.xlsx)	**15 SI**
**10**	**Table S3** – Results from EADEG (S3A-sheet 1) and GSEA (S3B-sheet 2) analysis (Table_S3.xlsx)	**16 SI**
**11**	**Table S4** – List of protein-coding genes differentially expressed (Table_S4.xlsx)	**17 SI**
**12**	**Table S5** – Core set of genes detected as differentially expressed in all iron-stress studies compared in this article	**18 SI**
**13**	**Table S6** – List of Antisense RNAs differentially expressed	**20 SI**
**14**	**Table S7** – List of Small RNAs differentially expressed	**25 SI**
**15**	**Table S8** – List of predicted targets for the sRNAs induced during iron starvation	**28 SI**

File S1 – Extended Methods

1. Estimation of optimal parameters for soft clustering

The parameters for the fuzzy c-means (FCM) algorithm (i.e., the number of clusters *c* and the fuzzifier *m*) were obtained following the approach proposed by Schwämmle and Jensen (2010). An optimal value of 2.02 for the fuzzifier *m* was estimated from the number of clustered genes (N= 3218) and the number of time points (D=5) using their heuristically derived formula. This setting of parameter *m* ensures that no clusters are found for randomized data with the same number of genes and time points.

The minimum centroid distance (D_{min}) was calculated for repeated clustering, with c ranging from 2 to 20 (Figure S1). In general, D_{min} decreases monotonically. For values larger than c=4, however, D_{min} decreases notably less, indicting *c*=4 as an optimal solution (Schwämmle and Jensen 2010). The complete set of expressed genes resulting from filtering was utilized for this calculation.

2. Previous *Synechocystis* microarray studies included in meta-analysis

Microarray data obtained in our study were compared to three previously published microarray experiments that monitored the transcriptional response in *Synechocystis* to perturbations of extra-cellular iron concentration (Singh, McIntyre et al. 2003; Shcolnick, Summerfield et al. 2009; Houot, Floutier et al. 2007).

Singh et al. measured the gene expression during the recovery from stress produced by lack of iron. Firstly, cells were grown for at least 6 days in iron-free media at 30°C with a light intensity of 20-30 μmol photons $m^{-2} \cdot s^{-1}$. Subsequently, iron was added directly to the media and samples were taken at 0, 3, 12 and 24 hours after reconstitution.

Houot et al. induced iron starvation in *Synechocystis* cells by growing them in media containing 1 or 2 mM of ferric ammonium citrate for 2 days at 30°C with a light intensity around 30 μmol photons $m^{-2} \cdot s^{-1}$. Cells were washed and re-suspended in iron-free media for another 2 days before RNA was extracted. Houot et al. also measured gene expression in cells grown under iron excess. Here, cells were exposed to an iron concentration 1000 times higher (17mM) than that of standard BG11 (17 μM). Samples were taken 4 and 6 hours after the addition of the extra iron.

Shcolnick et al. extracted RNA from iron starved cells using desferrioxamine B (DFB) as a chelator (Shcolnick, Shaked et al. 2007). The cells were grown at 30°C with a light intensity of 60 μmol photons $m^{-2} \cdot s^{-1}$ in the modified BG11 media, YBG11, in the presence of 50 μM DFB.

Besides the differences introduced due to experimental set-up and sampling time, experiments also differed in the microarrays used: both Singh et al. and Shcolnick et al. applied the platform described by Postier et al. (Postier, Wang et al. 2003), which is based on a two stage polymerase chain reaction (PCR) method to create probes against 3165 genes in the *Synechocystis* chromosome; whereas Houot et al. used the commercially available CyanoCHIP (http://www.takara-bio.com), which employs PCR fragments of C-terminal coding regions from 3079 genes in the Synechocystis chromosome as probes.

As result, the four independent studies partially differed in the set of genes detected as differentially expressed. Such divergence is a common feature when comparing gene expression data sets derived from different platforms. More importantly, however, the gene sets derived from the four studies show significant overlap. The results from our study are most similar to those by Houot et al. with 165 shared genes (40.5 % of differentially expressed genes in Houot et al.) (Figure S5). Less overlap was detected with the studies of Shcolnick et al. (112 genes; 33.7 %) and Singh et al. (72 genes; 23.8 %). In general, the measured expression levels tend to be concurrent, as shown by the calculated Pearson's correlation coefficients of $\log_2$FC for differentially expressed genes between our and previous studies. The highest correlation was obtained for the study by Houot et al. (R2 = 0.55) (Figure 4D), followed by Shcolnick et al. (R2 = 0.47) (Figure 4C) and Singh et al. (R2 = 0.46) (Figure 4B). Based on the corresponding contingency tables for differential expression, the observed

similarity with previous studies was highly significant (Houot et al.: $p = 2.2x10^{-16}$, Shcolnick et al.: $p = 4.0x10^{-12}$, Singh et al.: $p = 0.02$).

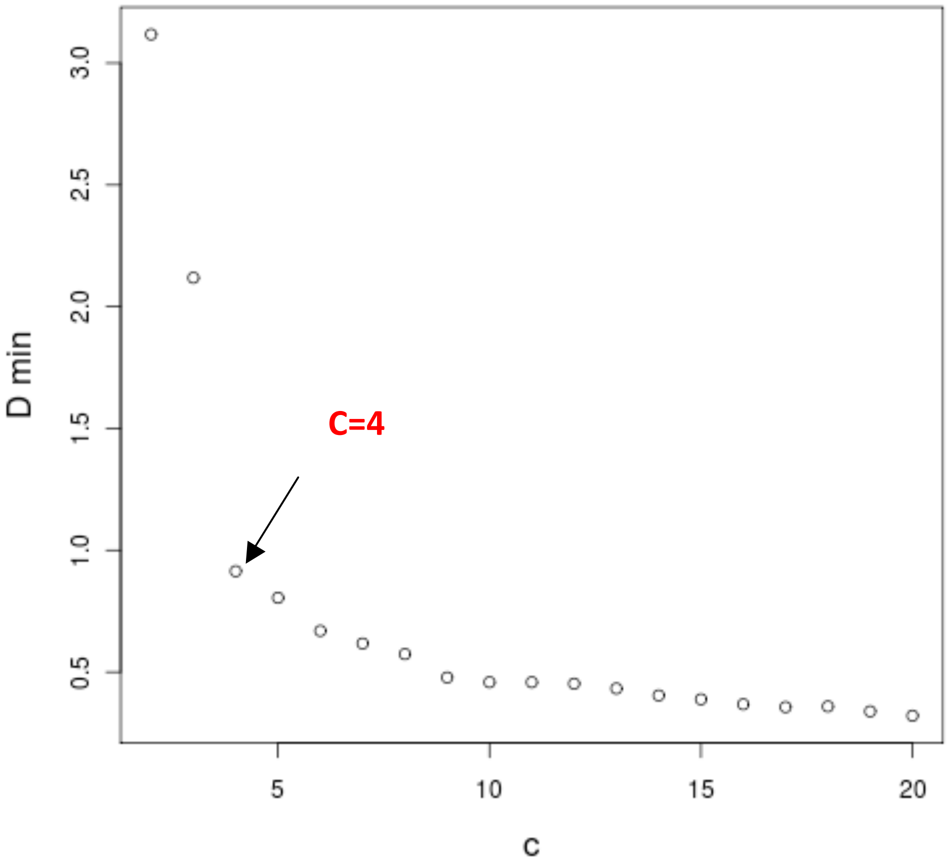

Figure S1 Minimum centroid distance for the variation of FCM parameter c. The expression data set was repeatedly clustered (N=10) for integer values of c from 2 to 20. For FCM clustering, the Bioconductor package *Mfuzz* was employed. After each clustering, the minimum centroid distance was calculated. The plot shows average minimum centroid distance with respect to parameter c. The average minimum centroid distance decreases notably less for c > 4, indicting c=4 as the optimal value.

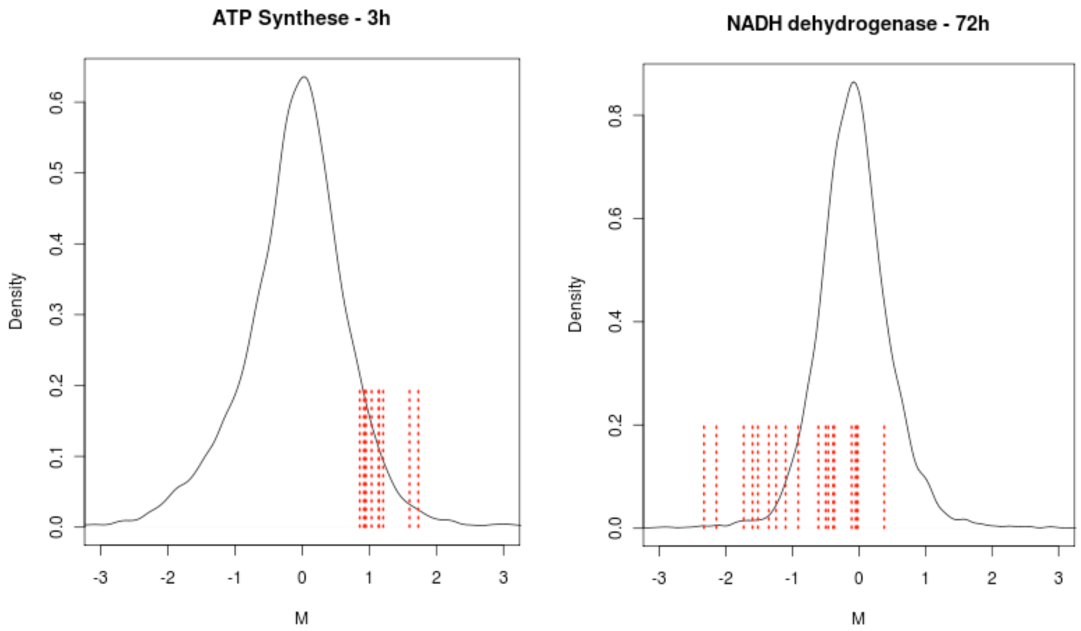

Figure S2 Differentially regulated gene sets detected by PGSEA. The plots show two examples of gene sets that were significant differentially regulated by parametric gene set enrichment analysis (PGSEA). The black line represents the distribution of logged fold changes (M) at time point 3h (left side) and 72h. The dashed red lines indicate the expression changes for genes associated with ATP synthase (left side) and NADH dehydrogenase (right side) in Cyanobase. Genes associated with ATP synthase tend to be up-regulated at time point 3h, whereas genes associated with NADH dehydrogenase tend to be down-regulated at time point 72h.

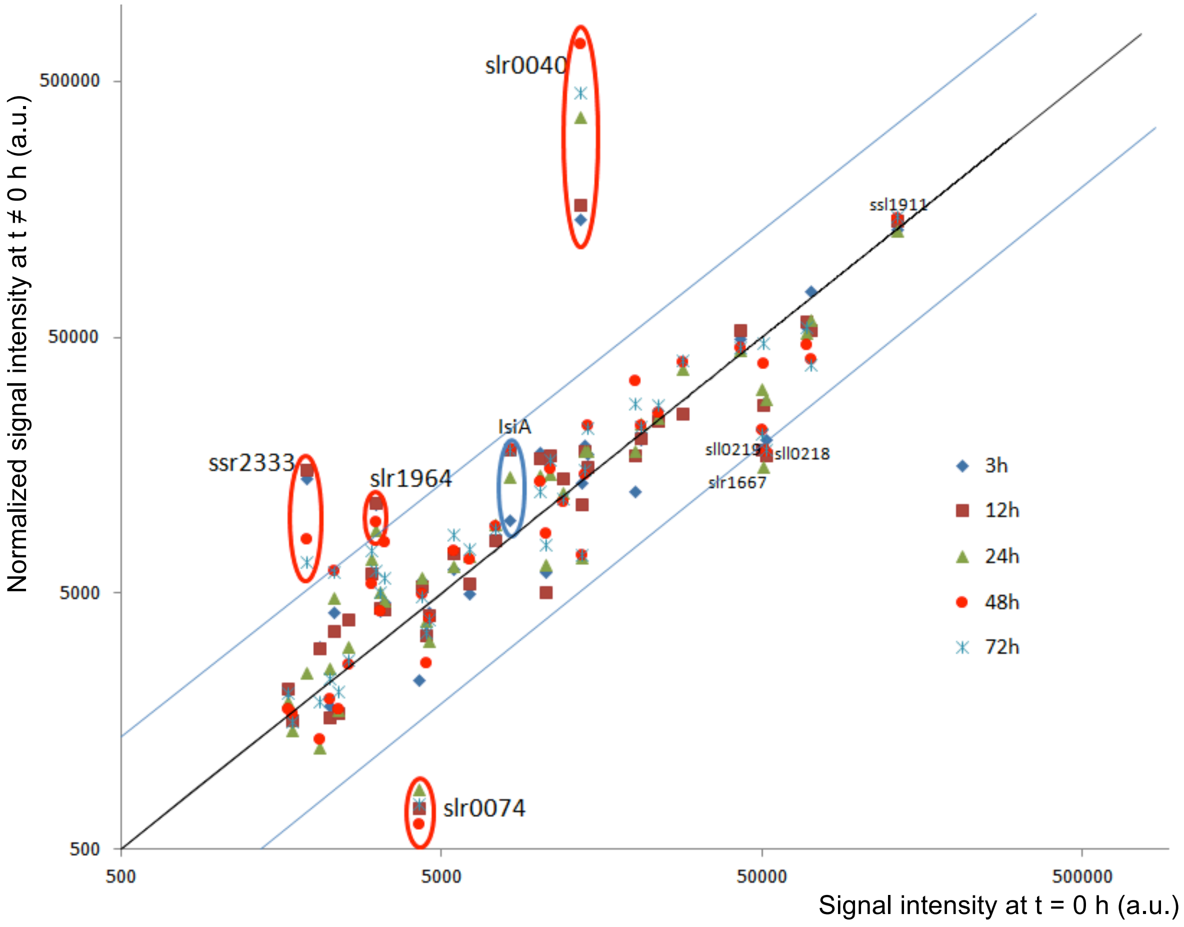

Normalized signal intensity at t ≠ 0 h (a.u.)
Signal intensity at t = 0 h (a.u.)
500000
50000
5000
500
500
5000
50000
500000
slr0040
ssl1911
IsiA
ssr2333
slr1964
sll0219
sll0218
slr1667
slr0074
3h
12h
24h
48h
72h

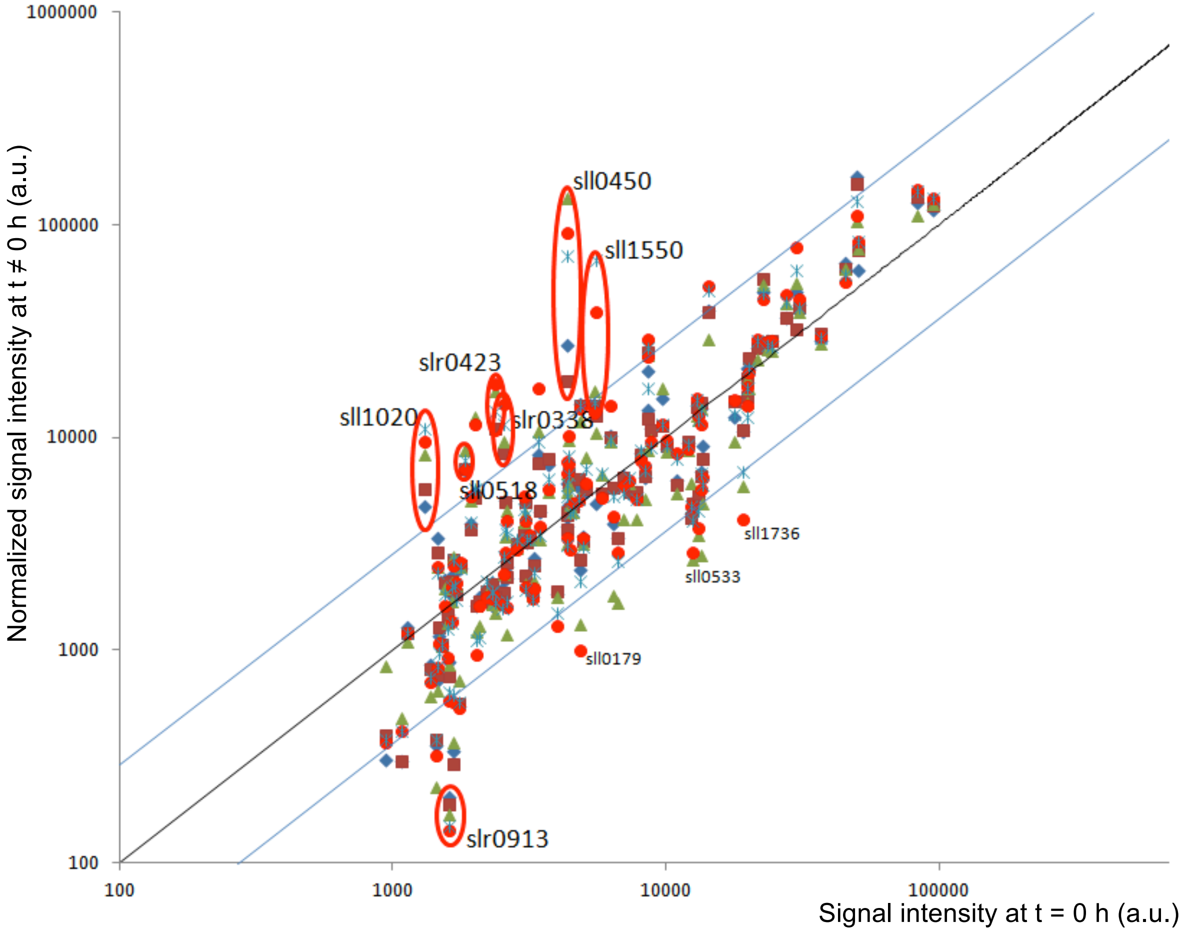

Normalized signal intensity at t ≠ 0 h (a.u.)
Signal intensity at t = 0 h (a.u.)
1000000
100000
10000
1000
100
100
1000
10000
100000
sll0450
sll1550
slr0423
sll1020
slr0338
sll0518
sll1736
sll0533
sll0179
slr0913

Figure S3 Expression of protein-coding genes with respect to the corresponding 5'UTRs and intragenic elements. To determine whether the differences in signal intensity detected between 5'UTR or intragenic elements with respect to the main gene were due to differences in transcript levels or were simply artifacts caused by different probe affinity, the signals were normalized as follows: We used the ratio obtained from dividing the intensity of (A) the 5'UTR or (B) intragenic element at each time point (Xt) by their intensity at time point 0h (X0h) to normalize the gene intensity at each sampled time (Yt). As a result, when plotting the gene intensity at time 0h (Y0h in the X axis) against its normalized signal intensity at each time point (e.g. [(X0h/X3h)*Y3h]), pairs where transcripts vary in a similar manner will fall around a X=Y central line, while pairs in which the 5'UTR or intragenic element is regulated differently to the gene will fall over or under it (red-circled genes). We have marked only those pairs for which all time points were over the set threshold as differently regulated.

Figure S4 is available for download at http://www.g3journal.org/lookup/suppl/doi:10.1534/g3.112.003863/-/DC1 or http://cyano.sysbiolab.eu/Publications/IronDeprivation.FigureS4.pdf (11.5MB)

Figure S4 Genome-wide overview combining 454 reads sequencing data for each nucleotide from the (+) and (-) cDNA populations from Mitschke *et al*. 2011 with the microarray data. The sequenced cDNA libraries were derived from untreated RNA (-), and from RNA enriched for primary transcripts by terminator exonuclease treatment (+). The reads of the (-) library (light grey) are accumulated on the reads of the (+) library (dark grey), reads are log2 transformed. The mean normalized log2 expression values (scale on left) of the 6 different microarray experiments with different time points of iron depletion are plotted for each probe as short horizontal bars that span the corresponding hybridization region. All probes of a single RNA feature are connected by lines. The control condition (0h –Fe) is plotted in green and the different time points are given in different shades of orange. A legend for the color code is given in the upper left region of the plot. Open reading frames are shown in dark blue, internal transcripts in light blue, UTRs in white, antisense RNAs in red and intergenic encoded ncRNAs in yellow.

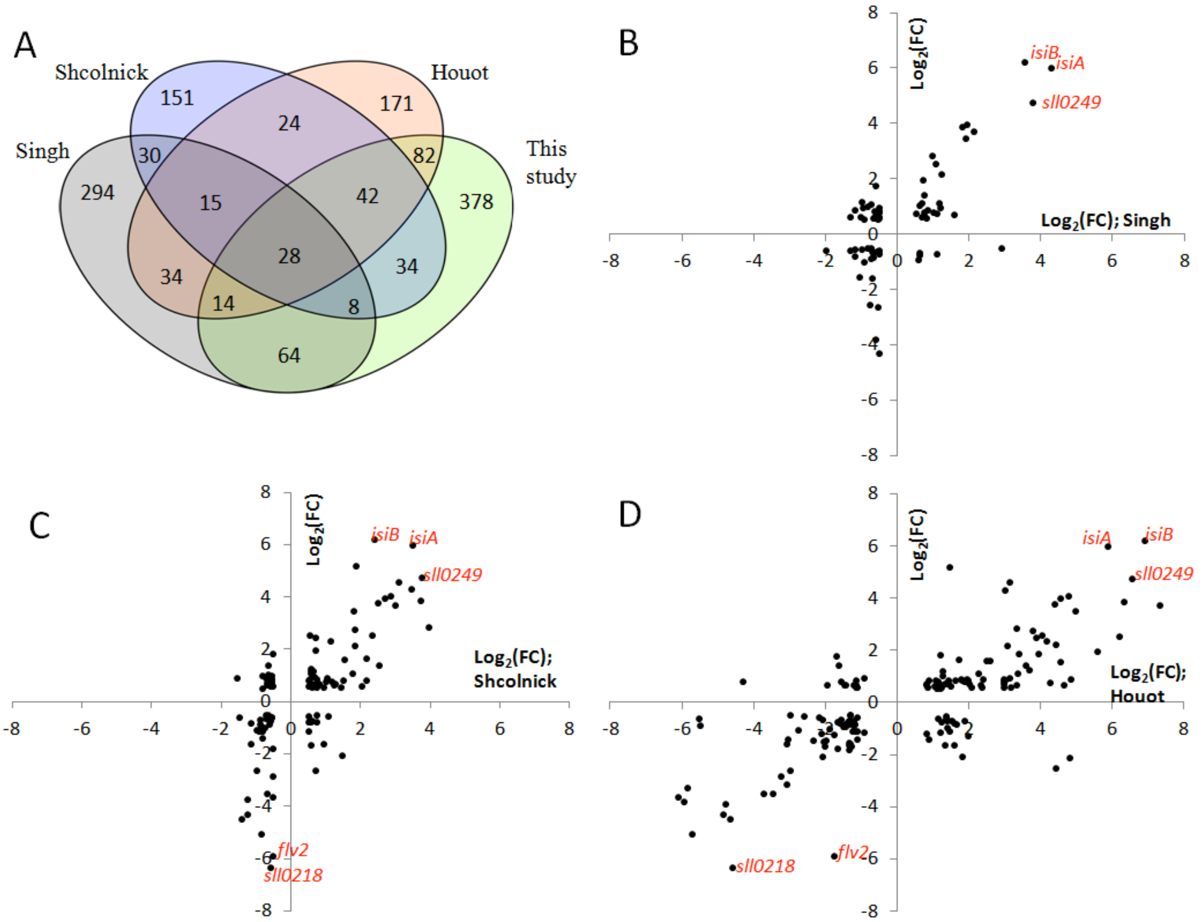

Figure S5 Comparision of microarray experiments. (A) Venn diagram that display the number of protein-coding genes differentially expressed (absolute log2(FC) ≥ 0.5, p value < 0.05) in the compared experiments. Numbers in the overlapping areas indicate genes that exhibited differential expression in either two, three or four of the studies compared. (B-D) The expression changes between samples grown in iron repleted and depleted conditions obtained in each of the compared experiments were plotted in the form of log2(FC) against the median log2(FC) obtained in our analysis. Key genes in the iron stress response, such as isiA and isiB, and genes encoding the flavodiiron protein (flv2) and the cotranscribed sll0218, which undergo down regulation upon DFB addition, are indicated in red.

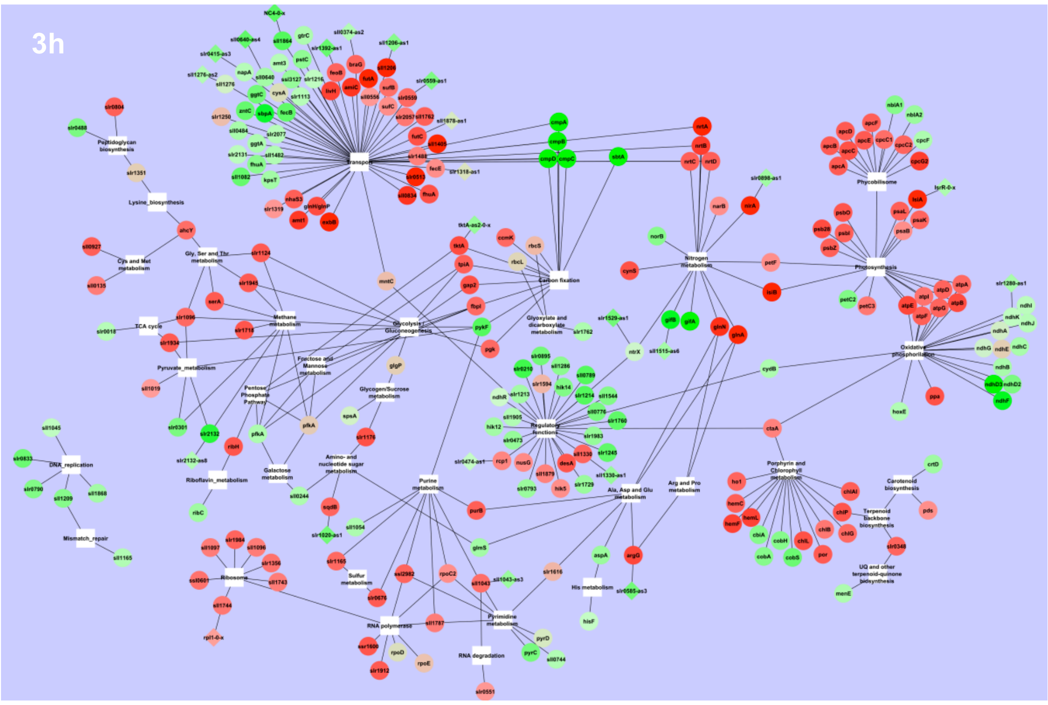
3h

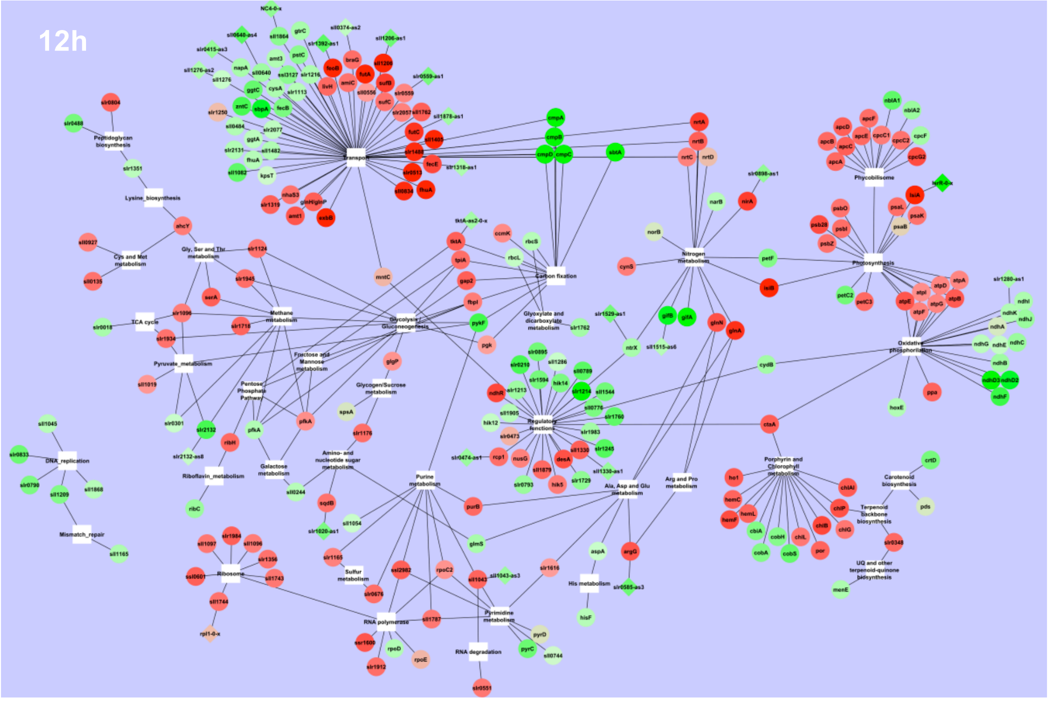
12h

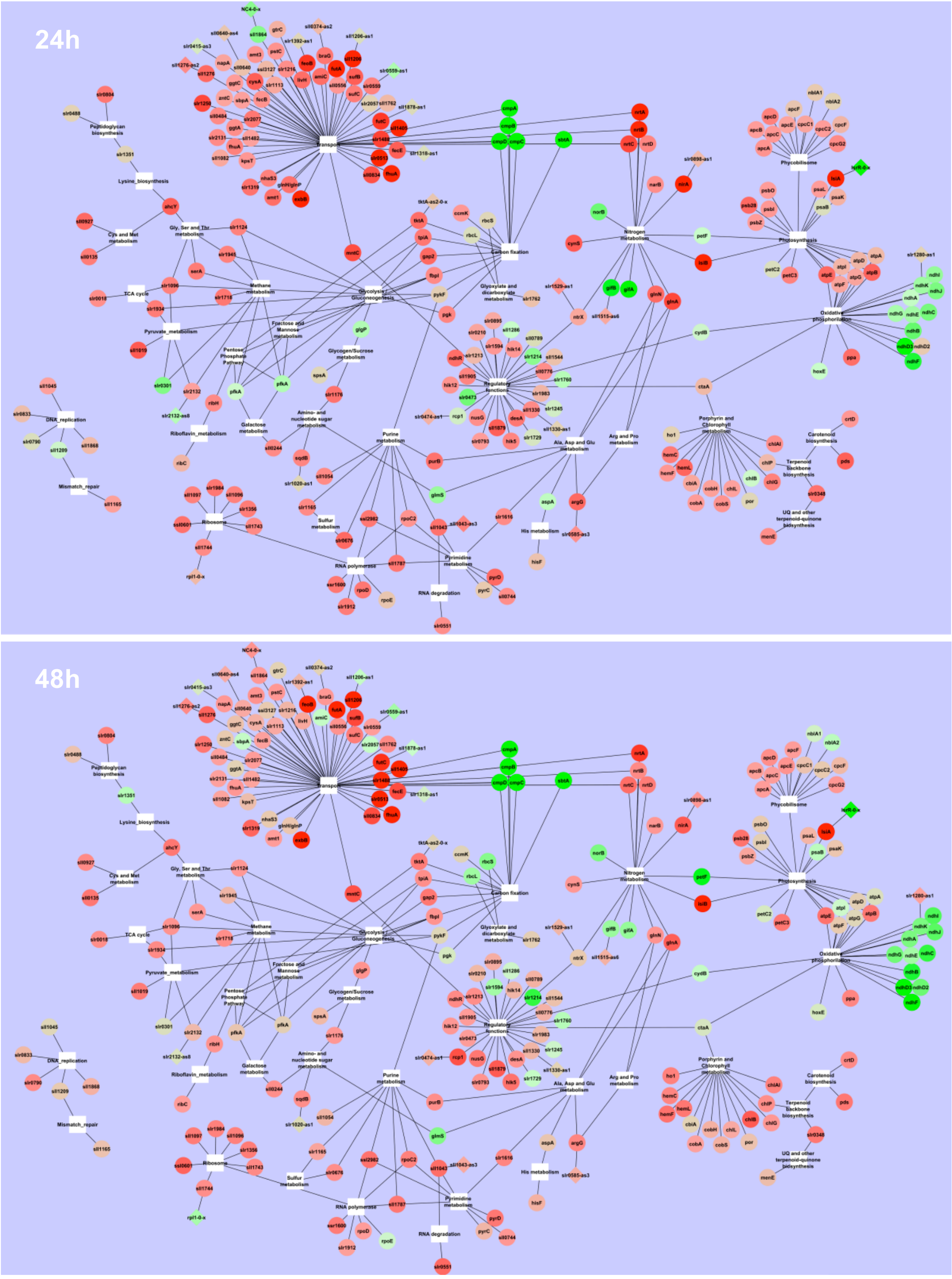
24h
48h

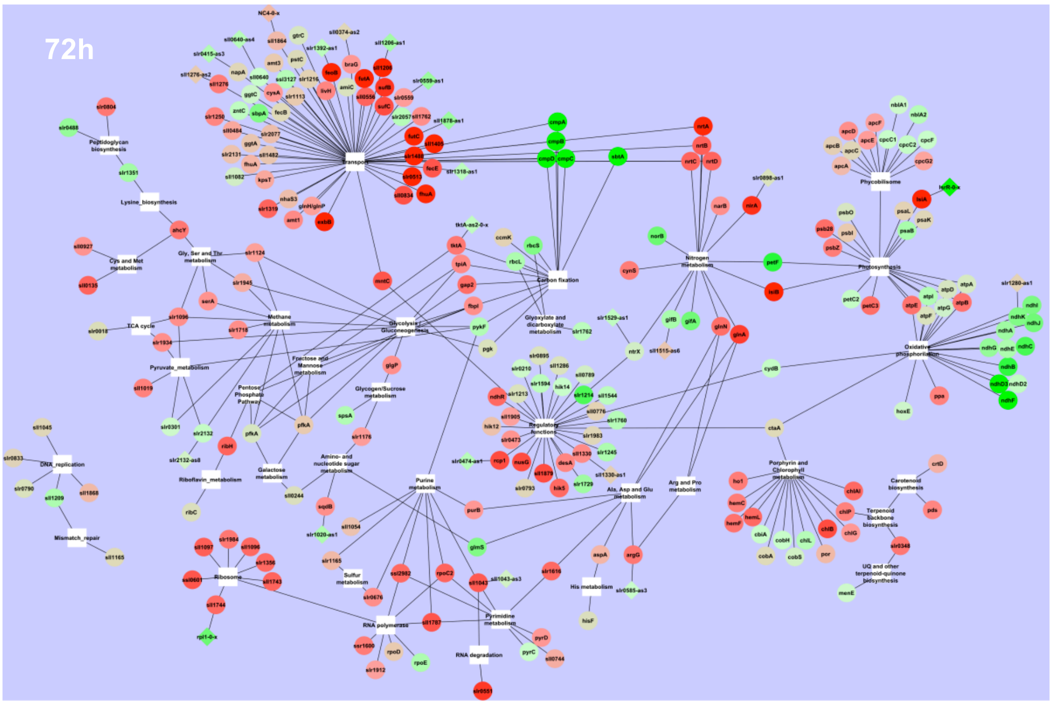

Figure S6 Functional networks based on KEGG pathways. Protein-coding genes (represented by circular nodes), which were differentially expressed under iron limiting conditions, are linked to their corresponding KEGG pathway (square nodes). Furthermore, differentially expressed asRNAs (diamond nodes) are linked to their complementary protein-coding genes. Genes with functional roles in more than one pathway are linked to all of them (such as glnA and glnN, which are key elements of three KEGG pathways “Nitrogen metabolism”, “Arginine and proline metabolism” and “Alanine, aspartate, and glutamate metabolism”). The magnitude of expression changes at different time points are color-coded with shades of red indicating induction and shades of green indicating repression.

Table S1 List of primers used to generate single-stranded RNA probes to test gene expression under iron limiting conditions.

Region targeted	Primer name	Sequence
5’UTR-isiA	5´UTR-isiA-fw	5’-TAATACGACTCACTATAGG GCACAGAATTGCCTCCTTAATTGA-3’
	5`UTR-isiA-rev	5’-ATCAGTGGTTTGAGCTTAGTCC-3’
NC-181	NC-181-T7-fw	5’-TAATACGACTCACTATAGG GCAAAAAGTTAACAACGGACACG-3’
	NC-181-rev	5’-AGTGTTCTCTTCTCAAGGATTCAG-3’
NC-1321	NC-1321-T7-fw	5’-TAATACGACTCACTATAGG GCAAGGTAGATTGGCAGTGGG-3’
	NC-1321-rev	5’-CATGGTCAACAACAGGAGAAGG-3’
NC-350	NC-350-T7-fw	5’-TAATACGACTCACTATAGG GGAGCCGGTGGGGAGAG-3’
	NC-350-rev	5´-CGAAGTAAGTTTTTCGATTCCGTC-3’
rnpB	rnpB-T7-fw	5’-TAATACGACTCACTATAGG CCTTTTCAGTGGTCAGTTACC-3’
	rnpB-rev	5’-AGGCCAAACTTGCTGGGTAAC-3’

Table S2 Soft clustering membership values. Membership values were calculated for each gene in the corresponding clusters; the highest membership value for each gene indicates the pertinence to that specific cluster.

Available for download at http://www.g3journal.org/lookup/suppl/doi:10.1534/g3.112.003863/-/DC1.

Table S3 EADEG and GSEA analyses of genes differentially expressed. Functional categories (gene sets) were taken from the Cyanobase as well as KEGG pathways (light green). Cyanobase categories (light blue) and sub-categories (white) were enlarged by an extra category "non-coding" that includes sRNAs and asRNAs. Only results for those categories which were significantly affected (False discovery rate (FDR) < 0.2) for at least one time point are shown. **(A)** EADEG results. N: total number of genes included in the functional category; M: Genes differentially expressed. **(B)** GSEA results. Z-value is a linear-scaled value that indicates the expression value of genes within a category compared with the overall mean expression value. To calculate the z-values for a given gene set and infer its significance value (FDR) against a standard normal distribution, we used the Bioconductor package PGSEA.

Available for download at http://www.g3journal.org/lookup/suppl/doi:10.1534/g3.112.003863/-/DC1.

Table S4 Protein-coding genes differentially expressed. Genes differentially expressed ($|Log_2FC| \geq 1$ and q- value < 0.05) at least at one of the measured time points during iron limiting conditions are displayed in the table below. Non protein-coding genes differentially expressed have been compiled separately in tables S5 (asRNAs) and S6 (sRNAs).

Available for download at http://www.g3journal.org/lookup/suppl/doi:10.1534/g3.112.003863/-/DC1.

Table S5 Genes detected as differentially expressed in all iron-stress studies compared in this article.

Gene ID	NAME	Houot et al.				Sch	Singh et al.			This study				
		+Fe 4h	+Fe 6h	-Fe A	-Fe B	SHC	3h	12h	24h	3 h	12 h	24 h	48 h	72 h
slr0452	dihydroxyacid dehydratase	0.55	1.31	-0.58	-0.27	-0.66	-0.72	-0.95	-0.55	0.37	-0.20	-0.54	-0.68	-0.50
slr0899	cyanate lyase	-1.73	-3.32	2.49	2.41	0.77	-0.08	-0.56	-0.93	1.46	0.62	1.26	0.12	0.65
sll0247	iron-stress chlorophyll-binding protein, isiA	-3.17	-5.89	5.45	3.49	3.51	3.38	4.27	4.47	2.52	5.99	5.53	6.14	6.15
sll0248	flavodoxin, IsiB	-3.81	-6.91	6.49	4.19	2.40	3.83	3.53	3.19	2.83	6.21	5.44	6.66	6.86
sll1408	transcriptional regulator	-1.62	-4.26	1.91	1.40	1.16	1.02	0.99	0.83	-0.51	0.76	0.17	0.87	0.78
slr1214	two-component response regulator PatA subfamily	-0.14	-1.81	1.03	2.08	1.47	0.71	-0.10	-0.19	-2.03	-3.40	-1.46	-2.31	-2.07
slr0955	probable tRNA/rRNA methyltransfer ase	0.3	0.6	-0.40	-1.06	-0.55	-0.39	0.03	0.97	0.72	0.96	0.81	0.86	0.66
sll1406	ferrichrome-iron receptor	-3.26	-6.19	4.48	3.05	2.34	1.08	1.06	1.20	1.54	3.51	2.43	2.75	2.52
sll1878	iron(III)-transport ATP-binding protein	-1.54	-3.07	1.27	1.40	1.85	1.23	1.23	1.20	1.53	2.15	1.98	2.29	2.60
slr0447	ABC-type urea transport system substrate-binding protein	-1.43	-3.01	1.63	0.86	-0.66	-0.36	-1.18	-1.50	2.15	0.84	0.90	-0.87	-0.57
slr0513	iron transport system substrate-binding protein	-1.85	-4.55	3.30	2.67	2.71	1.92	2.09	1.93	3.32	4.00	3.64	4.00	3.96
slr1318	iron(III) dicitrate transport system ATP-binding protein	-1.91	-3.59	1.08	1.11	2.54	0.82	0.64	0.73	0.32	1.70	1.18	1.55	1.39
sll1407	probable methyltransfer ase	-4.31	-5.58	3.80	2.81	0.73	0.70	0.77	0.55	0.53	2.35	1.46	2.10	1.95
sll0249	hypothetical protein	-7.13	-6.55	4.45	2.60	3.75	3.60	3.78	3.82	1.39	4.74	3.64	5.34	5.33
sll0451	hypothetical protein	0.37	-1.51	-0.51	-0.18	-0.62	-0.41	-0.53	-0.60	-1.20	-0.96	-0.20	-0.49	-0.73
sll0529	hypothetical protein	-0.17	1.15	-0.24	0.32	0.53	-0.53	-0.38	-0.79	0.98	1.30	0.79	0.36	0.81
sll0662	4Fe-4S type iron-sulfur protein	-0.68	-1.42	0.55	0.84	0.80	1.57	1.94	1.54	0.18	0.68	0.35	0.72	1.14
sll1549	salt-enhanced periplasmic protein	-3.7	-7.33	4.88	3.57	3.00	2.33	2.11	2.14	1.74	3.93	3.12	3.70	3.87
sll1734	protein involved in	-0.63	3.48	0.21	0.21	-0.68	-0.37	-0.41	-0.56	-3.20	-4.05	-2.99	-3.62	-3.52

slr1512	CO2 uptake sodium-dependent bicarbonate transporter	-0.26	4.84	3.45	-0.38	-1.25	-0.53	-0.33	-0.58	-3.67	-4.30	-3.46	-5.54	-5.28
ssl0461	hypothetical protein	-0.21	-3.34	1.03	-0.09	3.96	1.16	0.96	0.74	0.89	2.83	1.87	3.37	3.42
ssl1263	hypothetical protein	-0.25	-1.62	0.16	1.03	0.74	0.81	0.86	0.96	0.36	0.92	0.41	0.85	1.13
sll1862	unknown protein	-0.32	-2.98	-4.27	-0.86	2.18	0.31	0.73	1.10	-0.28	0.24	0.80	3.58	4.36
sll1863	unknown protein	-0.27	-2.97	-2.66	-0.83	2.03	0.75	0.82	0.80	-0.92	-0.26	0.59	3.53	4.51
slr0006	unknown protein	0.37	3.07	-0.56	-0.25	-1.15	-0.70	-0.36	-0.86	-1.61	-1.28	-1.43	-2.07	-2.15
slr1484	unknown protein	-2.82	-4.99	2.17	2.23	1.80	1.91	2.37	1.91	1.73	3.60	2.89	3.47	3.77
slr1485	putative phosphatidylinositol phosphate kinase	-2.92	-6.31	3.19	3.38	3.74	1.82	1.85	1.10	2.09	3.92	3.46	3.86	4.47
slr1544	LilA, light-harvesting-like (Lil) protein A	-0.24	1.21	-0.84	0.42	0.61	0.86	1.10	1.08	-1.19	-1.76	0.97	-0.05	-0.73

Table S6 Antisense RNAs differentially expressed. asRNAs are ordered and colored similarly to the two clusters defined in figure 4B (cluster I, red shade; cluster II, blue shade).

Gene ID	Description	3 hours	12 hours	24 hours	48 hours	72 hours	*q*-value
accA-0-x	sll0728-as	-1.69	-1.57	-0.27	-0.57	-1.21	$2.10 \cdot 10^{-5}$
cysT-0-x	slr1453-as	-0.26	-0.12	1.41	0.88	0.62	$4.79 \cdot 10^{-5}$
fbp-0-x	sll1636-as	-2.73	-2.63	-1.88	-1.60	-1.63	$1.29 \cdot 10^{-5}$
hik31-0-x	sll0790-as	-0.28	-0.33	1.26	0.51	0.22	$4.78 \cdot 10^{-6}$
kaiA-as1-0-x	slr0756-as	-1.57	-1.66	-0.55	-0.59	-0.79	$8.16 \cdot 10^{-6}$
sll0006-as1	asRNA	-1.49	-1.46	-0.82	-0.39	-0.76	$7.03 \cdot 10^{-8}$
sll0019-as2	asRNA	-2.17	-2.00	-0.75	-0.18	-1.04	$3.44 \cdot 10^{-4}$
sll0026-as3	asRNA	-1.63	-1.63	-0.15	-0.12	-0.85	$1.13 \cdot 10^{-3}$
sll0033-as1	asRNA	-1.85	-1.85	-0.31	0.06	-0.82	$1.71 \cdot 10^{-7}$
sll0034-as1	asRNA	-1.53	-1.71	-1.01	-1.01	-1.37	$1.43 \cdot 10^{-5}$
sll0040-as2	asRNA	-0.97	-1.04	0.29	0.34	-0.32	$2.23 \cdot 10^{-5}$
sll0067-as2	asRNA	-1.98	-2.00	-0.21	-0.46	-1.00	$6.41 \cdot 10^{-7}$
sll0067-as3	asRNA	-1.61	-1.77	-0.84	-0.60	-1.10	$2.07 \cdot 10^{-5}$
sll0068-as3	asRNA	-1.44	-1.37	0.21	-0.14	-0.72	$1.08 \cdot 10^{-4}$
sll0068-as4	asRNA	-1.87	-1.88	-0.21	-0.32	-1.06	$1.06 \cdot 10^{-4}$
sll0068-as6	asRNA	-1.64	-1.73	-0.06	0.01	-0.80	$1.66 \cdot 10^{-7}$
sll0083-as1	asRNA	-1.13	-1.03	-0.13	-0.20	-0.42	$1.37 \cdot 10^{-6}$
sll0095-as1	asRNA	-1.34	-1.28	0.19	-0.17	-0.46	$2.31 \cdot 10^{-6}$
sll0157-as1	asRNA	-1.23	-1.29	-0.17	-0.26	-0.66	$1.25 \cdot 10^{-3}$
sll0172-as1	asRNA	-1.14	-1.20	-0.20	-0.56	-1.03	$2.09 \cdot 10^{-7}$
sll0172-as2	asRNA	-1.54	-1.49	0.43	-0.33	-0.77	$1.11 \cdot 10^{-6}$
sll0174-as1	asRNA	-1.28	-1.63	-0.10	-0.01	-0.58	$9.62 \cdot 10^{-5}$
sll0204-as2	asRNA	-0.20	-0.12	1.01	0.55	0.47	$7.31 \cdot 10^{-5}$
sll0225-as1	asRNA	-1.23	-1.13	-0.03	-0.03	-0.62	$5.30 \cdot 10^{-7}$
sll0226-as1	asRNA	-1.59	-1.45	0.29	-0.15	-0.87	$1.56 \cdot 10^{-6}$
sll0227-as1	asRNA	-1.51	-1.44	-0.15	-0.02	-0.71	$7.51 \cdot 10^{-4}$
sll0230-as1	asRNA	-1.25	-0.97	0.50	-0.15	-0.49	$8.22 \cdot 10^{-7}$
sll0238-as1	asRNA	-1.20	-1.19	-0.16	-0.33	-0.89	$4.33 \cdot 10^{-6}$
sll0297-as1	asRNA	-1.01	-1.07	0.02	-0.24	-0.77	$1.30 \cdot 10^{-6}$
sll0330-as2	asRNA	-1.26	-1.21	-0.73	0.14	-0.74	$1.68 \cdot 10^{-4}$
sll0370-as1	asRNA	-1.03	-1.05	-0.57	-0.15	-0.77	$2.34 \cdot 10^{-4}$
sll0374-as2	asRNA	-1.05	-0.89	-0.19	-0.41	-0.45	$2.11 \cdot 10^{-4}$
sll0416-as2	asRNA	-1.15	-1.20	-0.32	-0.45	-0.96	$6.58 \cdot 10^{-6}$
sll0424-as1	asRNA	-1.13	-1.65	-0.82	-0.54	-1.12	$5.41 \cdot 10^{-6}$
sll0474-as1	asRNA	-1.94	-1.93	-0.24	-0.03	-0.96	$6.21 \cdot 10^{-5}$
sll0477-as1	asRNA	-2.04	-2.07	-0.90	-0.20	-1.30	$5.12 \cdot 10^{-5}$
sll0480-as1	asRNA	-2.32	-2.17	-0.96	-0.28	-1.12	$7.00 \cdot 10^{-5}$
sll0495-as1	asRNA	-1.15	-1.21	0.00	-0.47	-0.81	$3.58 \cdot 10^{-7}$
sll0502-as1	asRNA	-2.06	-2.02	-0.27	-0.05	-0.86	$1.74 \cdot 10^{-6}$
sll0503-as1	asRNA	-1.76	-1.67	-0.63	-0.69	-1.10	$1.97 \cdot 10^{-5}$
sll0506-as1	asRNA	-1.49	-1.56	-0.37	0.08	-0.48	$5.80 \cdot 10^{-6}$
sll0518-as1	asRNA	-0.82	-1.25	-0.25	-0.35	-0.76	$4.61 \cdot 10^{-5}$
sll0540-as1	asRNA	-1.08	-0.83	-0.34	0.10	0.21	$4.54 \cdot 10^{-6}$
sll0543-as1	asRNA	-1.33	-1.32	0.13	-0.31	-0.82	$3.64 \cdot 10^{-7}$
sll0574-as1	asRNA	-1.33	-1.51	-0.11	0.23	-0.48	$4.91 \cdot 10^{-5}$
sll0616-as1	asRNA	-1.90	-1.90	-0.02	-0.37	-1.02	$4.36 \cdot 10^{-8}$
sll0638-as1	asRNA	-1.19	-1.10	0.57	0.33	-0.33	$1.63 \cdot 10^{-3}$
sll0640-as4	asRNA	-1.91	-1.99	-0.32	-0.09	-0.98	$4.21 \cdot 10^{-5}$
sll0732-as2	asRNA	-0.96	-1.01	0.53	-0.25	-0.57	$2.60 \cdot 10^{-4}$
sll0759-as2	asRNA	-1.11	-0.89	0.00	-0.33	-0.48	$3.72 \cdot 10^{-6}$
sll0778-0-x	asRNA	-1.19	-1.20	0.46	-0.02	-0.46	$5.97 \cdot 10^{-6}$
sll0779-as1	asRNA	-1.37	-1.28	-0.58	-0.34	-0.84	$2.98 \cdot 10^{-3}$
sll0807-as1	asRNA	-1.52	-1.38	-0.47	-0.35	-0.53	$3.30 \cdot 10^{-6}$
sll0814-as2	asRNA	-0.20	-0.14	1.02	0.49	0.47	$1.28 \cdot 10^{-5}$
sll0825-as1	asRNA	-1.22	-1.10	-0.15	0.15	-0.46	$3.28 \cdot 10^{-7}$
sll0830-as1	asRNA	-1.14	-1.13	0.47	0.01	-0.82	$1.53 \cdot 10^{-7}$
sll0843-as1	asRNA	-2.21	-2.29	-0.64	-0.34	-1.07	$2.39 \cdot 10^{-6}$
sll0887-as1	asRNA	-1.85	-2.16	-1.28	-1.15	-1.69	$3.36 \cdot 10^{-7}$
sll0901-as1	asRNA	-1.02	-1.16	0.20	-0.16	-0.61	$1.93 \cdot 10^{-6}$
sll0913-as1	asRNA	-1.09	-0.95	-0.16	-0.47	-0.66	$1.86 \cdot 10^{-6}$

sll0920-as1	asRNA	-1.90	-1.90	-0.63	-0.53	-1.14	$1.64 \cdot 10^{-6}$
sll0926-1-x	asRNA	-1.08	-0.77	0.02	-0.02	-0.34	$1.00 \cdot 10^{-4}$
sll0931-as2	asRNA	-1.47	-1.53	-0.26	-0.36	-0.95	$2.07 \cdot 10^{-5}$
sll0992-as1	asRNA	-2.06	-2.17	-1.04	-0.41	-1.04	$3.29 \cdot 10^{-8}$
sll0992-as2	asRNA	-1.25	-1.26	0.34	0.12	-0.25	$1.43 \cdot 10^{-4}$
sll1023-as2	asRNA	-1.11	-1.03	0.33	0.36	-0.23	$3.50 \cdot 10^{-6}$
sll1043-as3	asRNA	-1.31	-1.30	0.54	-0.06	-0.70	$4.18 \cdot 10^{-7}$
sll1049-0-x	asRNA	-1.36	-1.30	0.19	-0.05	-0.51	$8.54 \cdot 10^{-6}$
sll1060-as1	asRNA	-1.46	-1.47	0.09	-0.06	-0.51	$1.57 \cdot 10^{-6}$
sll1119-as4	asRNA	-1.23	-1.36	0.17	-0.19	-0.63	$7.02 \cdot 10^{-7}$
sll1123-as1	asRNA	-0.72	-0.87	-0.20	-0.46	-1.07	$3.91 \cdot 10^{-4}$
sll1130-as1	asRNA	-1.15	-1.03	-0.27	-0.06	-0.72	$8.91 \cdot 10^{-4}$
sll1131-as1	asRNA	-0.96	-1.10	-0.38	-0.24	-0.74	$5.93 \cdot 10^{-7}$
sll1172-as1	asRNA	-1.26	-1.24	-0.65	-0.85	-1.28	$4.18 \cdot 10^{-7}$
sll1178-as2	asRNA	-1.78	-1.67	-0.60	-0.39	-0.89	$2.63 \cdot 10^{-5}$
sll1200-as1	asRNA	-1.38	-1.40	-0.62	-0.62	-0.49	$8.17 \cdot 10^{-7}$
sll1200-as2	asRNA	-1.20	-1.12	-0.34	-0.67	-0.36	$5.43 \cdot 10^{-5}$
sll1204-as1	asRNA	-1.51	-1.35	-0.05	-0.08	-0.66	$1.17 \cdot 10^{-4}$
sll1206-as1	asRNA	-1.52	-1.76	-0.53	-0.86	-1.21	$1.30 \cdot 10^{-5}$
sll1275-as1	asRNA	-2.09	-1.68	-0.66	-0.73	-1.08	$3.58 \cdot 10^{-4}$
sll1276-as2	asRNA	-1.04	-1.08	0.43	0.38	-0.34	$1.21 \cdot 10^{-5}$
sll1283-as2	asRNA	-1.89	-1.74	-0.32	-0.59	-1.09	$6.69 \cdot 10^{-8}$
sll1285-as1	asRNA	-1.56	-1.66	0.10	-0.20	-0.65	$1.73 \cdot 10^{-7}$
sll1293-as1	asRNA	-1.21	-0.95	-0.32	-0.26	-0.54	$1.43 \cdot 10^{-6}$
sll1293-as2	asRNA	-1.17	-1.09	-0.20	0.01	-0.51	$2.05 \cdot 10^{-4}$
sll1296-as3	asRNA	-1.56	-1.56	-0.36	-0.40	-0.77	$1.04 \cdot 10^{-6}$
sll1330-as1	asRNA	-1.34	-1.35	-0.41	-0.42	-0.40	$1.09 \cdot 10^{-4}$
sll1334-as3	asRNA	-1.61	-1.88	-0.10	-0.38	-0.87	$3.65 \cdot 10^{-7}$
sll1341-as1	asRNA	-1.62	-1.81	-0.46	-0.49	-1.06	$1.69 \cdot 10^{-5}$
sll1354-as3	asRNA	-1.32	-1.23	-0.25	-0.26	-0.90	$7.28 \cdot 10^{-4}$
sll1378-as2	asRNA	-1.85	-1.93	-0.78	-0.30	-1.07	$6.63 \cdot 10^{-8}$
sll1386-as1	asRNA	-1.69	-1.74	-0.19	-0.25	-0.98	$7.90 \cdot 10^{-8}$
sll1432-as1	asRNA	-0.83	-1.00	-0.25	-0.47	-0.67	$9.69 \cdot 10^{-5}$
sll1434-as1	asRNA	-2.02	-2.03	-0.17	-0.26	-0.82	$5.94 \cdot 10^{-7}$
sll1459-as1	asRNA	-1.21	-1.23	-0.47	-0.15	-0.70	$7.12 \cdot 10^{-6}$
sll1486-as1	asRNA	-2.18	-2.06	-0.11	-0.46	-0.98	$1.45 \cdot 10^{-6}$
sll1500-as1	asRNA	-1.43	-1.48	0.41	0.31	-0.26	$2.61 \cdot 10^{-5}$
sll1515-as6	asRNA	-1.19	-1.02	-0.07	-0.14	-0.31	$3.89 \cdot 10^{-3}$
sll1525-as1	asRNA	-1.37	-1.37	-0.64	-0.29	-0.88	$1.68 \cdot 10^{-4}$
sll1538-as1	asRNA	-1.17	-1.18	-0.29	-0.50	-0.81	$1.88 \cdot 10^{-5}$
sll1601-as1	asRNA	-0.16	-0.77	-0.39	-0.54	-1.01	$3.78 \cdot 10^{-4}$
sll1612-as1	asRNA	-2.23	-1.92	-0.75	-0.52	-1.25	$4.41 \cdot 10^{-4}$
sll1635-as1	asRNA	-1.87	-1.90	-0.52	0.00	-0.82	$7.81 \cdot 10^{-3}$
sll1641-as1	asRNA	-1.70	-1.78	0.16	-0.23	-0.76	$2.84 \cdot 10^{-6}$
sll1677-as1	asRNA	-1.69	-1.65	0.36	0.05	-0.59	$3.72 \cdot 10^{-6}$
sll1685-as3	asRNA	-1.17	-1.06	-0.65	-0.29	-0.58	0.01
sll1722-as1	asRNA	-1.78	-1.80	-0.21	-0.09	-0.88	$1.87 \cdot 10^{-6}$
sll1724-as1	asRNA	-1.60	-1.58	-0.26	-0.23	-0.76	$3.26 \cdot 10^{-5}$
sll1750-as4	asRNA	-0.98	-1.05	-0.36	-0.29	-0.78	$1.84 \cdot 10^{-4}$
sll1800-as3	asRNA	-0.75	-0.94	-0.40	-0.55	-1.01	$1.24 \cdot 10^{-5}$
sll1853-as1	asRNA	-1.79	-1.88	-0.59	-0.42	-1.12	$2.68 \cdot 10^{-8}$
sll1866-as1	asRNA	-1.61	-1.71	-1.22	-1.28	-1.25	$8.47 \cdot 10^{-7}$
sll1870-as1	asRNA	-1.02	-1.20	0.41	0.16	-0.04	$5.93 \cdot 10^{-5}$
sll1878-as1	asRNA	-0.62	-1.25	-0.51	-0.81	-1.31	$1.45 \cdot 10^{-4}$
sll1878-as2	asRNA	-0.90	-1.10	0.66	0.73	-0.07	$1.37 \cdot 10^{-4}$
sll1886-as1	asRNA	-1.16	-1.12	-0.34	-0.12	-0.59	$1.53 \cdot 10^{-4}$
sll1906-as1	asRNA	0.07	-0.01	1.17	0.36	0.20	$1.76 \cdot 10^{-6}$
sll1927-as1	asRNA	-1.43	-1.54	0.35	-0.16	-0.76	$4.77 \cdot 10^{-6}$
sll1987-as1	asRNA	-1.68	-1.67	-0.44	-0.44	-0.98	$2.81 \cdot 10^{-6}$
sll2002-as1	asRNA	-0.81	-1.01	0.83	0.93	0.06	$3.25 \cdot 10^{-3}$
sll2003-as3	asRNA	-1.01	-1.04	0.18	-0.10	-0.59	$4.52 \cdot 10^{-4}$
sll2008-as1	asRNA	-1.03	-1.08	0.29	0.06	-0.48	$2.12 \cdot 10^{-6}$
sll7029-as1	asRNA	-1.38	-1.52	-0.56	-0.85	-1.13	$2.42 \cdot 10^{-6}$
sll7063-as1	asRNA	-1.09	-1.13	-0.14	-0.35	-0.88	$2.57 \cdot 10^{-7}$
sll7077-as1	asRNA	-1.70	-1.88	-0.01	-0.28	-0.95	$4.66 \cdot 10^{-6}$

slr0007-as1	asRNA	-1.01	-1.11	-0.35	-0.48	-0.76	$5.60·10^{-7}$
slr0079-as1	asRNA	-1.45	-1.42	-0.25	-0.40	-0.77	$5.00·10^{-6}$
slr0080-as2	asRNA	-1.34	-0.98	-0.57	-0.55	-0.65	$4.94·10^{-6}$
slr0208-as1	asRNA	-1.86	-1.91	0.02	-0.18	-0.91	$2.41·10^{-6}$
slr0208-as2	asRNA	-1.16	-1.29	-0.43	-0.26	-0.58	$3.94·10^{-6}$
slr0211-as2	asRNA	-1.01	-1.09	0.37	-0.33	-0.80	$6.57·10^{-7}$
slr0244-as3	asRNA	-0.97	-1.02	-0.53	-0.68	-0.74	$6.89·10^{-3}$
slr0252-as1	asRNA	-1.80	-1.87	-0.98	-0.90	-1.29	$2.93·10^{-3}$
slr0293-as1	asRNA	-1.68	-1.63	0.08	0.07	-0.81	$1.21·10^{-4}$
slr0304-as1	asRNA	-1.46	-1.53	-0.62	-0.60	-0.90	$4.25·10^{-6}$
slr0327-as1	asRNA	-1.32	-1.16	-0.03	0.03	-0.71	$1.56·10^{-5}$
slr0331-as2	asRNA	-1.21	-1.14	-0.69	-0.79	-0.78	$2.50·10^{-4}$
slr0337-as1	asRNA	-1.25	-1.35	0.36	-0.24	-0.74	$2.38·10^{-7}$
slr0345-as1	asRNA	-1.14	-1.11	0.43	-0.01	-0.60	$3.05·10^{-6}$
slr0359-as2	asRNA	-1.09	-1.11	0.07	0.03	-0.72	$6.65·10^{-4}$
slr0370-as2	asRNA	-1.00	-0.84	-0.24	-0.23	-0.63	$3.78·10^{-6}$
slr0370-as3	asRNA	-1.40	-1.19	-0.69	-0.27	-0.96	$1.46·10^{-4}$
slr0377-as1	asRNA	-1.48	-1.37	-0.72	-0.63	-0.91	$2.83·10^{-6}$
slr0408-0-x	asRNA	-2.13	-2.11	-0.65	-0.28	-0.95	$5.51·10^{-6}$
slr0408-1-x	asRNA	-1.84	-1.80	0.19	-0.05	-0.82	$6.77·10^{-4}$
slr0408-as11	asRNA	-1.67	-1.77	0.22	-0.14	-0.83	$1.10·10^{-7}$
slr0415-as3	asRNA	-1.49	-1.35	-0.63	-0.62	-1.07	$5.37·10^{-7}$
slr0467-as1	asRNA	-1.31	-1.39	-0.22	-0.07	-0.77	$6.84·10^{-6}$
slr0474-as1	asRNA	-0.85	-1.32	-0.13	-0.12	-1.00	$5.24·10^{-6}$
slr0488-as1	asRNA	-1.21	-1.14	0.38	0.25	-0.27	$3.16·10^{-4}$
slr0519-as1	asRNA	-1.14	-0.98	0.28	-0.60	-0.39	$9.03·10^{-5}$
slr0534-as5	asRNA	-1.80	-1.90	-0.52	-0.48	-0.83	$2.59·10^{-7}$
slr0541-as1	asRNA	0.62	0.54	1.69	0.80	0.59	$8.98·10^{-6}$
slr0559-as1	asRNA	-1.62	-1.70	-1.09	-1.18	-1.33	$2.12·10^{-6}$
slr0579-as1	asRNA	-1.25	-1.23	0.60	0.27	-0.24	$9.94·10^{-7}$
slr0585-as3	asRNA	-1.71	-1.76	0.34	-0.10	-0.78	$1.11·10^{-5}$
slr0593-as1	asRNA	-2.09	-1.95	-0.94	0.04	-1.09	$9.62·10^{-4}$
slr0599-as1	asRNA	-1.48	-1.52	-0.02	0.10	-0.50	$3.27·10^{-5}$
slr0619-as1	asRNA	-1.29	-1.24	-0.46	-0.42	-0.59	$1.08·10^{-5}$
slr0711-as1	asRNA	-1.66	-1.74	0.26	-0.23	-0.91	$1.29·10^{-6}$
slr0727-as1	asRNA	-1.38	-1.50	-0.05	0.17	-0.62	$2.60·10^{-4}$
slr0822-as2	asRNA	-1.38	-1.16	-0.17	-0.56	-1.01	$3.89·10^{-4}$
slr0842-as1	asRNA	-1.31	-1.33	0.54	0.07	-0.57	$3.48·10^{-5}$
slr0848-0-x	asRNA	-0.99	-1.13	-0.30	-0.46	-0.87	$1.11·10^{-5}$
slr0872-as1	asRNA	-1.21	-1.24	-0.27	-0.41	-0.90	$1.72·10^{-4}$
slr0872-as2	asRNA	-1.04	-1.03	0.19	0.07	-0.28	$1.10·10^{-5}$
slr0898-as1	asRNA	-1.28	-1.40	-0.23	0.12	-0.57	$6.49·10^{-6}$
slr0900-as1	asRNA	-1.28	-1.43	-0.96	-0.88	-1.22	$1.34·10^{-6}$
slr0905-as3	asRNA	-1.60	-1.45	0.32	0.09	-0.54	$9.04·10^{-7}$
slr0936-as2	asRNA	-1.84	-1.94	-0.24	-0.25	-1.02	$1.87·10^{-6}$
slr0942-as1	asRNA	-0.99	-1.11	-0.72	0.01	-0.71	0.01
slr0993-as4	asRNA	-0.99	-1.04	0.30	-0.12	-0.43	$3.29·10^{-7}$
slr1020-as1	asRNA	-1.75	-1.64	-0.46	-0.51	-1.08	$1.67·10^{-4}$
slr1022-as1	asRNA	-1.57	-1.59	-0.22	-0.11	-0.83	$6.35·10^{-8}$
slr1028-as15	asRNA	-1.03	-1.07	-0.37	-0.38	-0.77	$4.55·10^{-5}$
slr1039-as2	asRNA	-2.66	-2.41	-0.57	-0.07	-0.92	$2.17·10^{-5}$
slr1050-as1	asRNA	-1.47	-1.48	-0.44	-0.22	-0.79	$7.52·10^{-6}$
slr1051-as2	asRNA	-1.47	-1.41	-0.50	-0.36	-0.83	$5.54·10^{-5}$
slr1101-as1	asRNA	-1.92	-1.96	-0.42	-0.50	-0.94	$2.91·10^{-5}$
slr1102-0-x	asRNA	-1.17	-1.06	-0.49	-0.37	-0.61	$6.75·10^{-6}$
slr1103-as4	asRNA	-1.50	-1.54	-0.11	-0.36	-1.08	$1.21·10^{-5}$
slr1104-as3	asRNA	-1.20	-1.33	0.33	0.03	-0.48	$4.82·10^{-6}$
slr1123-as1	asRNA	-1.57	-1.64	-0.16	-0.10	-0.67	$8.61·10^{-8}$
slr1161-as1	asRNA	-1.72	-2.38	-0.32	-0.05	-0.76	$1.71·10^{-7}$
slr1174-as1	asRNA	-1.14	-1.21	0.28	-0.22	-0.94	$1.07·10^{-6}$
slr1181-as1	asRNA	-1.78	-1.90	-0.06	-0.36	-1.00	$2.65·10^{-6}$
slr1207-0-x	asRNA	-1.28	-1.30	0.61	0.27	-0.36	$6.51·10^{-7}$
slr1207-as2	asRNA	-0.49	-0.52	1.12	0.61	0.12	$6.10·10^{-4}$
slr1219-as2	asRNA	-1.18	-1.19	-0.22	0.00	-0.63	$9.39·10^{-6}$
slr1228-as1	asRNA	-2.32	-2.18	0.14	-0.30	-0.94	$3.08·10^{-7}$

slr1229-as1	asRNA	-0.14	-0.23	1.43	0.86	0.62	$1.34 \cdot 10^{-4}$
slr1254-as1	asRNA	-1.16	-1.07	0.28	-0.04	-0.51	$1.38 \cdot 10^{-5}$
slr1272-as1	asRNA	-2.95	-2.82	-1.03	-0.38	-1.43	$4.22 \cdot 10^{-4}$
slr1280-as1	asRNA	-1.16	-1.17	-0.41	0.02	-0.41	$1.71 \cdot 10^{-5}$
slr1293-as1	asRNA	-1.21	-1.27	-0.40	-0.22	-0.70	$8.39 \cdot 10^{-6}$
slr1305-as1	asRNA	-2.29	-2.15	-0.69	-0.27	-1.44	$4.14 \cdot 10^{-6}$
slr1318-as1	asRNA	-0.57	-1.11	-0.52	-0.67	-0.94	$8.30 \cdot 10^{-6}$
slr1324-as3	asRNA	-1.19	-1.15	-0.53	-0.59	-1.04	$8.19 \cdot 10^{-5}$
slr1347-as1	asRNA	-1.02	-1.03	0.15	-0.03	-0.53	$1.77 \cdot 10^{-6}$
slr1367-as1	asRNA	-1.05	-1.21	0.05	0.11	-0.32	$3.27 \cdot 10^{-4}$
slr1383-as1	asRNA	-1.64	-1.55	-0.17	-0.23	-0.81	$8.74 \cdot 10^{-5}$
slr1392-as1	asRNA	-1.82	-1.96	-0.46	-0.17	-0.95	$4.24 \cdot 10^{-5}$
slr1403-as6	asRNA	-1.55	-1.52	-0.62	-0.01	-0.78	$1.06 \cdot 10^{-4}$
slr1403-as8	asRNA	-1.16	-1.17	-0.72	-0.09	-0.67	$1.19 \cdot 10^{-6}$
slr1428-as1	asRNA	-0.66	-0.63	1.06	0.45	0.12	$8.21 \cdot 10^{-6}$
slr1435-as1	asRNA	-1.23	-1.30	0.46	-0.16	-0.41	$4.90 \cdot 10^{-5}$
slr1462-as1	asRNA	-2.20	-2.18	-0.99	-0.61	-1.19	$1.97 \cdot 10^{-7}$
slr1462-as2	asRNA	-1.36	-1.38	-0.43	-0.08	-0.54	$7.26 \cdot 10^{-6}$
slr1489-as1	asRNA	-1.84	-1.61	-0.79	-0.51	-0.84	$5.99 \cdot 10^{-6}$
slr1512-as1	asRNA	-1.41	-1.51	0.10	0.01	-0.74	$3.60 \cdot 10^{-7}$
slr1529-as1	asRNA	-1.73	-1.73	0.22	-0.18	-0.89	$1.16 \cdot 10^{-4}$
slr1535-as1	asRNA	-1.00	-1.10	-0.12	-0.39	-0.70	$1.15 \cdot 10^{-5}$
slr1550-as1	asRNA	-1.05	-1.09	-0.12	-0.16	-0.46	$6.82 \cdot 10^{-5}$
slr1579-as1	asRNA	-1.73	-1.95	0.05	-0.32	-0.84	$1.09 \cdot 10^{-6}$
slr1591-as1	asRNA	-2.26	-2.52	-1.88	-2.04	-1.82	$4.24 \cdot 10^{-6}$
slr1609-as2	asRNA	-1.14	-1.21	-0.18	-0.06	-0.71	$6.52 \cdot 10^{-7}$
slr1666-as1	asRNA	-1.45	-1.51	0.10	-0.32	-0.73	$4.50 \cdot 10^{-6}$
slr1673-as2	asRNA	-1.51	-1.38	-0.41	-0.49	-0.95	$8.02 \cdot 10^{-6}$
slr1676-as1	asRNA	-1.05	-1.02	-0.26	-0.33	-0.83	$3.82 \cdot 10^{-4}$
slr1679-as1	asRNA	-1.48	-1.67	-0.46	-0.10	-0.41	$1.18 \cdot 10^{-3}$
slr1691-as2	asRNA	-1.08	-0.94	-0.51	-0.18	-0.94	$3.00 \cdot 10^{-5}$
slr1704-as2	asRNA	-2.29	-2.27	-0.90	-0.29	-1.29	$1.87 \cdot 10^{-6}$
slr1727-as1	asRNA	-1.84	-1.89	-0.10	0.23	-0.92	$6.52 \cdot 10^{-3}$
slr1753-as2	asRNA	-1.11	-1.80	0.22	0.23	-0.48	$3.88 \cdot 10^{-6}$
slr1772-as1	asRNA	0.02	0.13	1.15	0.74	0.54	$8.67 \cdot 10^{-4}$
slr1777-as2	asRNA	-1.90	-2.07	-0.28	-0.47	-1.14	$8.45 \cdot 10^{-7}$
slr1780-as2	asRNA	-1.79	-1.65	-0.49	-0.45	-0.81	$3.43 \cdot 10^{-7}$
slr1839-as1	asRNA	-1.42	-1.43	0.02	-0.22	-0.64	$3.08 \cdot 10^{-7}$
slr1864-as1	asRNA	-1.49	-1.53	0.20	0.11	-0.63	$2.53 \cdot 10^{-5}$
slr1866-as1	asRNA	-1.34	-1.30	0.52	-0.06	-0.29	$2.25 \cdot 10^{-6}$
slr1876-as2	asRNA	-0.99	-1.09	-0.61	-0.46	-0.39	$3.87 \cdot 10^{-7}$
slr1876-as5	asRNA	-0.86	-1.01	-0.55	-0.41	-0.43	$1.77 \cdot 10^{-6}$
slr1908-as1	asRNA	-1.44	-1.26	0.28	-0.24	-0.69	$1.01 \cdot 10^{-6}$
slr1929-as1	asRNA	-1.31	-1.36	0.22	-0.31	-0.60	$3.16 \cdot 10^{-7}$
slr1942-as1	asRNA	-1.48	-1.37	0.26	-0.02	-0.68	$1.28 \cdot 10^{-5}$
slr1944-as2	asRNA	-1.57	-1.69	-0.35	-0.10	-0.81	$3.45 \cdot 10^{-5}$
slr1968-as1	asRNA	-2.46	-2.61	-1.84	-1.76	-1.85	$1.71 \cdot 10^{-5}$
slr1968-as3	asRNA	-1.21	-1.07	-0.06	-0.17	-0.11	$4.35 \cdot 10^{-6}$
slr2009-as1	asRNA	-1.10	-1.04	-0.06	-0.22	-0.75	$7.73 \cdot 10^{-6}$
slr2023-as1	asRNA	-1.48	-1.48	0.48	-0.01	-0.49	$3.13 \cdot 10^{-5}$
slr2046-as5	asRNA	-1.79	-1.25	-0.03	-0.45	-0.81	$9.26 \cdot 10^{-6}$
slr2046-as6	asRNA	-1.35	-1.31	-0.32	-0.31	-0.76	$3.59 \cdot 10^{-3}$
slr2076-as2	asRNA	-0.76	-1.07	-0.35	-0.42	-0.67	$1.62 \cdot 10^{-3}$
slr2141-as1	asRNA	-1.49	-1.41	-0.83	-0.25	-0.96	$3.39 \cdot 10^{-4}$
spoIID-0-x	sll1283-as	-2.02	-1.93	-0.67	-0.22	-0.91	$6.42 \cdot 10^{-8}$
ssl0294-as1	asRNA	-1.11	-1.00	-0.33	-0.25	-0.53	$7.66 \cdot 10^{-4}$
ssl0426-as2	asRNA	-0.66	-0.59	1.08	0.52	-0.25	$1.43 \cdot 10^{-4}$
ssl3177-as1	asRNA	-1.09	-1.04	-0.01	0.02	-0.49	$2.58 \cdot 10^{-5}$
ssl3436-as1	asRNA	-1.10	-1.22	0.08	0.39	-0.16	0.04
ssr1375-as1	asRNA	-0.96	-1.23	-0.42	-0.43	-0.53	$5.63 \cdot 10^{-6}$
ssr1407-as1	asRNA	-1.62	-1.65	-0.41	0.18	-0.75	$1.45 \cdot 10^{-5}$
ssr1558-as1	asRNA	-0.24	-0.35	1.05	0.31	-0.20	$1.19 \cdot 10^{-4}$
ssr1604-as1	asRNA	-1.11	-0.97	0.16	-0.06	-0.50	$6.10 \cdot 10^{-5}$
ssr2318-as1	asRNA	-1.17	-1.07	-0.16	0.02	-0.79	$8.53 \cdot 10^{-6}$
tktA-as2-0-x	asRNA	-1.36	-1.21	-0.33	-0.43	-0.89	$5.16 \cdot 10^{-5}$

6803t13-0-x	slr1740-as	0.51	0.48	-0.03	0.48	1.77	$1.53\cdot10^{-7}$
IsrR-0-x	sll0247-as	-1.09	-7.43	-6.56	-7.25	-7.55	$1.13\cdot10^{-10}$
NC4-0-x	sll1864-as	-1.97	-1.39	-1.46	0.17	-0.20	$1.38\cdot10^{-5}$
NC5-0-x	sll1864-as	-1.43	-0.79	-1.09	0.87	0.31	$6.51\cdot10^{-5}$
rpl1-0-x	sll1744-as	0.02	-0.24	-0.21	-1.17	-1.78	$1.90\cdot10^{-7}$
rpl1-1-x	sll1744-as	0.07	-0.16	-0.06	-1.09	-1.62	$4.20\cdot10^{-7}$
sll0002-as1	asRNA	-1.05	-1.10	-1.18	-0.84	-1.22	$1.86\cdot10^{-3}$
sll0027-as1	asRNA	-0.65	-0.82	-0.73	-1.02	-1.08	$1.49\cdot10^{-5}$
sll0107-as1	asRNA	-0.31	-1.12	-0.99	-0.95	-1.27	$3.68\cdot10^{-7}$
sll0217-as2	asRNA	0.98	1.17	0.86	1.06	1.29	$4.44\cdot10^{-7}$
sll0247-as2	asRNA	-1.06	-7.04	-5.93	-6.61	-6.89	$1.98\cdot10^{-10}$
sll0270-as1	asRNA	-0.34	-0.57	-1.02	-0.80	-1.05	$1.89\cdot10^{-5}$
sll0456-as1	asRNA	1.05	1.47	1.69	1.08	1.19	$3.70\cdot10^{-4}$
sll0477-as2	asRNA	3.18	3.96	3.78	4.12	4.27	$5.44\cdot10^{-8}$
sll0898-as1	asRNA	-2.31	-1.35	-1.70	-0.71	-0.79	$9.93\cdot10^{-8}$
sll1098-as1	asRNA	0.12	0.18	0.01	-0.84	-1.14	$1.43\cdot10^{-6}$
sll1198-as1	asRNA	2.80	3.97	3.82	4.34	4.54	$2.10\cdot10^{-9}$
sll1289-as2	asRNA	1.10	0.95	0.68	0.34	0.07	$1.48\cdot10^{-7}$
sll1319-as1	asRNA	-1.19	-0.84	-1.21	-0.72	-0.09	$2.45\cdot10^{-7}$
sll1321-as1	asRNA	-1.92	-1.39	-1.27	-0.44	-0.34	$6.42\cdot10^{-7}$
sll1374-as3	asRNA	-1.11	-1.37	-1.19	-0.85	-1.05	$1.33\cdot10^{-4}$
sll1658-as1	asRNA	1.01	0.78	0.83	0.29	0.26	$2.32\cdot10^{-3}$
sll1723-as1	asRNA	1.01	0.64	0.25	-0.23	-0.17	$1.58\cdot10^{-6}$
sll1851-as1	asRNA	0.28	0.19	-0.14	-0.03	1.26	$2.32\cdot10^{-5}$
sll1860-as1	asRNA	0.97	1.09	0.54	0.37	0.60	$1.93\cdot10^{-6}$
sll1864-as1	asRNA	-1.82	-1.08	-1.55	0.60	0.15	$9.12\cdot10^{-5}$
sll1867-as1	asRNA	-0.42	0.28	-1.77	-1.16	-0.65	$1.04\cdot10^{-4}$
sll1900-as1	asRNA	-0.07	0.69	-0.59	1.10	1.03	$5.14\cdot10^{-6}$
sll1939-as1	asRNA	-0.20	-0.05	-0.41	-1.03	-1.33	$2.25\cdot10^{-7}$
slr0015-as1	asRNA	0.78	-0.92	-0.55	-2.34	-1.71	$5.54\cdot10^{-9}$
slr0144-as1	asRNA	1.50	1.29	0.90	0.96	1.02	$9.61\cdot10^{-5}$
slr0300-as1	asRNA	-1.11	-0.30	-0.92	-0.84	-0.45	$1.63\cdot10^{-5}$
slr0431-as3	asRNA	0.97	1.06	0.44	0.39	0.36	$6.00\cdot10^{-7}$
slr1053-as2	asRNA	1.08	0.31	-0.97	-1.11	-1.13	$2.22\cdot10^{-6}$
slr1053-as3	asRNA	0.44	-0.28	-1.23	-1.15	-1.34	$2.78\cdot10^{-8}$
slr1053-as4	asRNA	0.45	-0.28	-1.33	-1.26	-1.40	$1.20\cdot10^{-8}$
slr1118-as1	asRNA	-1.11	-0.82	-0.95	-0.41	-0.26	$4.81\cdot10^{-4}$
slr1464-as2	asRNA	-1.16	-1.06	-1.34	-0.19	0.63	$5.09\cdot10^{-7}$
slr1467-as1	asRNA	0.81	0.79	1.57	0.58	0.89	$7.38\cdot10^{-5}$
slr1636-as1	asRNA	1.39	1.19	0.68	0.72	0.73	$1.24\cdot10^{-3}$
slr1740-as1	asRNA	0.24	0.25	-0.18	0.18	1.09	$2.03\cdot10^{-5}$
slr1776-as2	asRNA	-1.19	-1.46	-1.46	-1.12	-1.25	$9.92\cdot10^{-7}$
slr1878-as1	asRNA	0.99	1.07	0.60	0.55	0.72	$2.27\cdot10^{-6}$
slr1900-as2	asRNA	1.64	1.24	0.50	0.47	0.09	$2.64\cdot10^{-6}$
slr1919-as1	asRNA	1.42	1.32	0.96	0.94	0.90	$2.92\cdot10^{-3}$
slr1935-as4	asRNA	-1.05	-0.94	-0.92	-0.55	-0.66	$2.79\cdot10^{-6}$
slr1964-0-x	asRNA	1.20	1.26	0.94	1.23	1.11	$2.90\cdot10^{-6}$
slr2026-as2	asRNA	1.02	0.74	0.35	-0.23	-0.20	$7.68\cdot10^{-7}$
slr2042-as1	asRNA	-0.12	-0.45	-0.42	-0.75	-1.05	$9.27\cdot10^{-6}$
slr2132-as8	asRNA	-1.04	-0.84	-1.02	-0.56	-0.71	$1.70\cdot10^{-4}$
slr2143-as1	asRNA	1.08	0.92	0.54	0.15	-0.20	$9.97\cdot10^{-7}$
ssl3382-as2	asRNA	0.55	1.01	0.72	0.78	1.20	$3.27\cdot10^{-3}$

Table S7 Small RNAs differentially expressed. sRNAs are divided and colored like the three clusters defined in figure 4C (top cluster I; cluster II, light green; bottom cluster III).

Gene ID	Description	3 hours	12 hours	24 hours	48 hours	72 hours	*q*-value
NC-1082	sRNA	1.42	1.34	0.74	0.32	-0.01	$5.04 \cdot 10^{-6}$
NC-1096	sRNA	1.15	1.21	0.88	0.76	0.88	$6.71 \cdot 10^{-4}$
NC-1171	sRNA	-1.59	-1.74	-2.13	-2.88	-2.48	$3.04 \cdot 10^{-9}$
NC-1188	sRNA	-1.67	-1.46	-1.16	-2.07	-2.15	$1.85 \cdot 10^{-7}$
NC-119	sRNA	1.37	1.11	0.73	0.93	0.73	$1.84 \cdot 10^{-7}$
NC-1190	sRNA	-1.52	-1.29	-1.12	-2.09	-2.10	$5.86 \cdot 10^{-7}$
NC-1248	sRNA	1.93	2.11	0.78	-0.82	0.29	$1.40 \cdot 10^{-4}$
NC-1261	sRNA	1.01	0.75	0.74	0.22	0.41	$1.01 \cdot 10^{-5}$
NC-1399	sRNA	1.25	1.04	0.20	0.14	-0.30	$5.65 \cdot 10^{-6}$
NC-1410	sRNA	-2.67	-1.80	-2.13	-3.34	-2.98	$6.89 \cdot 10^{-6}$
NC-1413	sRNA	-2.58	-1.43	-2.22	-3.33	-2.86	$6.01 \cdot 10^{-6}$
NC-1414	sRNA	1.19	1.29	0.38	0.07	0.17	$5.09 \cdot 10^{-5}$
NC-1435	sRNA	-0.85	-0.86	-0.75	-1.27	-1.02	$9.07 \cdot 10^{-5}$
NC-1442	sRNA	1.22	1.08	0.94	0.82	0.80	$3.01 \cdot 10^{-5}$
NC-1481	sRNA	-1.43	-1.55	-2.27	-2.89	-2.64	$1.21 \cdot 10^{-8}$
NC-156	sRNA	0.97	0.98	-0.39	-0.79	-1.06	$7.15 \cdot 10^{-7}$
NC-1606	sRNA	0.08	-0.97	-1.09	-1.08	-1.15	$3.80 \cdot 10^{-6}$
NC-1637	sRNA	0.21	-0.59	-0.36	-1.14	-1.14	$1.24 \cdot 10^{-6}$
NC-1673	sRNA	-3.17	-4.12	-3.07	-4.60	-4.17	$1.07 \cdot 10^{-10}$
NC-1690	sRNA	1.10	1.01	0.69	0.35	0.39	$9.27 \cdot 10^{-4}$
NC-17	sRNA	-0.11	-0.63	-1.16	-1.07	-1.07	$2.04 \cdot 10^{-7}$
NC-172	sRNA	-0.66	-0.12	-0.85	-1.23	-0.79	$8.79 \cdot 10^{-6}$
NC-176	sRNA	-0.59	0.00	-1.11	-1.27	-0.84	$3.01 \cdot 10^{-6}$
NC-196	sRNA	3.65	2.94	3.04	1.55	2.95	$1.32 \cdot 10^{-7}$
NC-271	sRNA	1.21	0.82	0.47	0.13	0.03	$9.28 \cdot 10^{-6}$
NC-285	sRNA	1.03	0.83	0.57	0.16	0.36	$1.80 \cdot 10^{-3}$
NC-3	sRNA	-0.43	-0.56	-0.97	-1.04	-0.93	$5.01 \cdot 10^{-7}$
NC-318	sRNA	1.78	1.21	1.36	0.12	0.61	$5.03 \cdot 10^{-6}$
NC-334	sRNA	1.80	2.49	0.81	0.47	0.38	$3.18 \cdot 10^{-8}$
NC-349	sRNA	1.29	0.84	0.08	-0.28	-0.45	$2.32 \cdot 10^{-7}$
NC-380	sRNA	1.86	1.62	1.57	0.68	0.91	$1.19 \cdot 10^{-6}$
NC-392	sRNA	-1.44	-1.53	-2.00	-2.54	-2.21	$6.71 \cdot 10^{-7}$
NC-407	sRNA	0.99	0.80	1.11	0.71	0.78	$3.29 \cdot 10^{-4}$
NC-430	sRNA	0.99	1.34	0.61	0.64	0.64	$3.47 \cdot 10^{-6}$
NC-431	sRNA	1.17	1.73	1.18	1.01	1.20	$1.12 \cdot 10^{-6}$
NC-445	sRNA	1.51	1.30	1.04	0.92	0.67	$3.32 \cdot 10^{-7}$
NC-492	sRNA	1.36	1.19	0.40	0.40	0.60	$3.38 \cdot 10^{-7}$
NC-520	sRNA	-0.50	-0.95	-1.32	-0.87	-0.97	$7.75 \cdot 10^{-7}$
NC-711	sRNA	2.12	1.86	0.73	0.12	0.00	$1.21 \cdot 10^{-6}$
NC-717	sRNA	1.17	0.86	-0.24	-0.37	-0.37	$2.22 \cdot 10^{-5}$
NC-83	sRNA	1.64	0.56	0.49	-0.02	-0.18	$7.73 \cdot 10^{-6}$
NC-841	sRNA	-1.14	-1.15	-1.49	-1.46	-1.42	$3.19 \cdot 10^{-5}$
NC-954	sRNA	-0.22	-0.75	-0.57	-1.38	-1.21	$8.41 \cdot 10^{-8}$
NC-995	sRNA	1.77	1.07	1.70	0.96	0.74	$7.37 \cdot 10^{-5}$
SyR2-0-x	sRNA	1.79	1.52	0.16	-0.09	-0.35	$4.92 \cdot 10^{-9}$
NC-108	sRNA	-1.47	-1.42	-0.56	-0.15	-0.89	$1.93 \cdot 10^{-5}$
NC1-0-x	sRNA	0.12	0.00	1.00	0.18	-0.13	$3.72 \cdot 10^{-4}$
NC-1103	sRNA	-0.59	-0.58	-0.54	-0.71	-1.70	$1.35 \cdot 10^{-6}$
NC-1134	sRNA	-1.30	-1.85	-0.81	-0.37	-0.68	$1.09 \cdot 10^{-6}$
NC-1136	sRNA	-1.29	-1.83	-0.66	-0.35	-0.44	$5.93 \cdot 10^{-7}$
NC-1148	sRNA	-1.48	-1.49	-0.28	-0.34	-0.72	$2.33 \cdot 10^{-5}$
NC-1331	sRNA	-1.21	-1.23	-0.06	-0.43	-0.92	$7.90 \cdot 10^{-8}$
NC-1484	sRNA	-1.20	-1.10	-0.32	-0.45	-1.05	$2.20 \cdot 10^{-6}$
NC-1491	sRNA	-1.86	-1.83	-0.37	-0.01	-1.07	$5.10 \cdot 10^{-7}$
NC-1499	sRNA	-1.44	-1.59	-1.29	-1.57	-1.85	$2.73 \cdot 10^{-7}$
NC-163	sRNA	-1.07	-1.33	-0.42	-0.41	-0.57	$6.28 \cdot 10^{-5}$
NC-1687	sRNA	-1.07	-0.89	-0.52	-0.23	-0.30	$2.04 \cdot 10^{-6}$
NC-1703	sRNA	-1.07	-1.33	0.00	-0.03	-0.57	$1.86 \cdot 10^{-4}$
NC-1706	sRNA	1.24	0.53	1.39	0.92	0.54	$6.68 \cdot 10^{-4}$
NC-1722	sRNA	0.88	0.16	1.31	0.97	0.34	$8.79 \cdot 10^{-6}$

NC-1731	sRNA	-1.45	-1.34	-0.37	0.23	-0.37	$3.01{\cdot}10^{-6}$
NC-1737	sRNA	-2.22	-2.22	-0.86	-0.63	-1.25	$3.30{\cdot}10^{-6}$
NC-207	sRNA	-0.23	-0.17	1.03	0.56	0.62	$5.46{\cdot}10^{-4}$
NC2-0-x	sRNA	0.65	0.66	1.09	0.66	0.81	$2.08{\cdot}10^{-5}$
NC-211	sRNA	-1.07	-1.34	-0.52	0.04	-0.57	$2.60{\cdot}10^{-3}$
NC-232	sRNA	-0.73	-1.16	-0.04	0.17	0.04	$2.06{\cdot}10^{-6}$
NC-244	sRNA	0.01	-0.62	1.55	0.88	0.37	$6.99{\cdot}10^{-5}$
NC-247	sRNA	-0.61	-1.17	-0.19	-0.03	-0.12	$8.11{\cdot}10^{-7}$
NC-265	sRNA	0.27	0.48	1.85	1.40	1.52	$2.49{\cdot}10^{-3}$
NC-269	sRNA	-1.21	-0.54	0.01	0.42	0.13	$3.64{\cdot}10^{-3}$
NC-272	sRNA	-1.27	-1.12	-0.56	-0.17	-0.15	$1.32{\cdot}10^{-6}$
NC-282	sRNA	-1.98	-2.27	-1.08	-1.20	-1.84	$1.92{\cdot}10^{-6}$
NC-288	sRNA	-1.65	-1.79	-0.18	0.22	-0.22	$2.03{\cdot}10^{-6}$
NC-29	sRNA	-0.69	-1.10	0.57	-0.65	-1.03	$5.19{\cdot}10^{-6}$
NC-296	sRNA	-1.39	-1.18	-0.97	-0.82	-0.82	$6.04{\cdot}10^{-6}$
NC-298	sRNA	-1.72	-1.42	-0.46	-0.46	-0.52	$9.97{\cdot}10^{-7}$
NC-309	sRNA	0.49	0.09	0.53	1.67	0.87	$8.52{\cdot}10^{-5}$
NC-347	sRNA	-1.95	-1.94	-0.86	-0.80	-1.38	$1.71{\cdot}10^{-7}$
NC-41	sRNA	-0.94	-1.22	0.07	-0.20	0.05	$1.29{\cdot}10^{-6}$
NC-421	sRNA	-1.08	-1.11	-0.59	-0.32	-0.56	$2.62{\cdot}10^{-6}$
NC-425	sRNA	-1.08	-1.55	-0.58	-0.17	-0.30	$4.26{\cdot}10^{-8}$
NC-433	sRNA	-1.93	-1.93	0.00	-0.23	-0.61	$1.96{\cdot}10^{-7}$
NC-449	sRNA	0.68	0.55	0.99	1.37	1.30	$3.41{\cdot}10^{-6}$
NC-516	sRNA	-0.71	-0.83	-0.71	-0.77	-1.01	$3.85{\cdot}10^{-4}$
NC-667	sRNA	0.02	0.11	1.22	0.20	0.21	$1.31{\cdot}10^{-5}$
NC-681	sRNA	-0.32	-0.96	1.06	0.77	-0.40	$5.08{\cdot}10^{-5}$
NC-693	sRNA	-1.10	-1.11	0.60	0.13	-0.24	$3.66{\cdot}10^{-4}$
NC-764	sRNA	-0.69	-1.08	-0.64	-0.27	-0.24	$1.74{\cdot}10^{-5}$
NC-766	sRNA	-1.05	-1.05	-0.32	-0.02	-0.61	$1.13{\cdot}10^{-5}$
NC-882	sRNA	-2.41	-2.60	-1.55	-1.61	-1.69	$5.16{\cdot}10^{-7}$
NC-895	sRNA	-0.59	-1.41	-0.87	-1.36	-1.13	$1.40{\cdot}10^{-6}$
NC-980	sRNA	-0.82	-1.49	-0.54	-0.80	-0.50	$2.42{\cdot}10^{-6}$
NC-981	sRNA	-0.39	-1.11	-0.44	-0.75	-0.57	$1.84{\cdot}10^{-5}$
ncRNA 983563:983626	sRNA	-0.69	-1.09	0.59	-0.67	-1.04	$4.90{\cdot}10^{-6}$
SyR4-0-x	sRNA	-1.61	-1.44	-1.03	-1.29	-1.27	$9.53{\cdot}10^{-5}$
NC-1036	sRNA	1.94	1.75	1.64	1.72	3.82	$7.75{\cdot}10^{-7}$
NC-110	sRNA	-2.05	-1.83	-1.97	-1.62	-1.77	$9.02{\cdot}10^{-7}$
NC-111	sRNA	0.81	1.03	0.30	0.69	0.81	$4.79{\cdot}10^{-7}$
NC-1208	sRNA	-0.10	0.12	0.22	0.68	1.49	$1.66{\cdot}10^{-7}$
NC-1321	sRNA	0.40	1.36	0.68	1.54	1.67	$5.32{\cdot}10^{-8}$
NC-137	sRNA	-1.51	-0.41	-2.02	-0.71	-0.55	$1.77{\cdot}10^{-4}$
NC-1371	sRNA	-1.72	-1.90	-1.64	-1.50	-1.18	$5.96{\cdot}10^{-6}$
NC-1382	sRNA	0.83	1.00	-0.29	0.59	0.72	$3.05{\cdot}10^{-6}$
NC-142	sRNA	-0.41	0.26	-1.43	-0.13	0.09	$2.93{\cdot}10^{-5}$
NC-1453	sRNA	-1.14	-0.89	-0.75	-0.08	0.11	$8.07{\cdot}10^{-7}$
NC-1567	sRNA	-3.25	-1.65	-2.22	-3.05	-2.37	$1.79{\cdot}10^{-5}$
NC-1619	sRNA	-0.48	-0.07	-1.06	-0.63	-0.28	$2.66{\cdot}10^{-3}$
NC-1630	sRNA	0.24	0.53	-0.17	-0.17	1.15	$1.52{\cdot}10^{-6}$
NC-168	sRNA	-1.00	-0.52	-2.15	-0.70	-0.10	$2.40{\cdot}10^{-4}$
NC-181	sRNA	1.54	3.32	3.34	3.75	3.77	$3.85{\cdot}10^{-8}$
NC-233	sRNA	-0.67	0.37	-1.02	-0.68	0.00	$1.83{\cdot}10^{-4}$
NC-253	sRNA	-1.37	-1.21	-1.24	-1.09	-0.99	$7.48{\cdot}10^{-5}$
NC-316	sRNA	0.64	0.61	0.38	1.14	0.51	$4.39{\cdot}10^{-4}$
NC-319	sRNA	-0.74	1.68	0.94	0.22	0.85	$6.46{\cdot}10^{-6}$
NC-324	sRNA	-1.23	0.62	0.61	0.22	0.39	$6.50{\cdot}10^{-4}$
NC-330	sRNA	-0.82	-0.18	-1.09	-0.54	-0.67	$1.49{\cdot}10^{-3}$
NC-350	sRNA	0.38	1.07	0.51	0.84	1.52	$8.03{\cdot}10^{-8}$
NC-361	sRNA	-0.18	0.35	-1.53	-0.08	0.10	$3.11{\cdot}10^{-5}$
NC-404	sRNA	-0.22	0.33	-1.66	-0.02	-0.04	$3.47{\cdot}10^{-5}$
NC-436	sRNA	0.75	0.76	0.75	1.38	1.30	$4.85{\cdot}10^{-5}$
NC-52	sRNA	-1.40	-1.24	-1.13	-0.56	-0.73	$1.25{\cdot}10^{-6}$
NC-688	sRNA	-0.14	0.39	-0.02	0.55	1.01	$1.50{\cdot}10^{-3}$
NC-833	sRNA	-2.12	-1.31	-1.25	0.19	0.23	$4.38{\cdot}10^{-7}$
NC-870	sRNA	0.76	1.15	0.86	0.69	1.09	$8.96{\cdot}10^{-6}$

NC-91	sRNA	-1.11	-0.63	-0.86	-0.51	-0.50	$1.33 \cdot 10^{-4}$

Table S8 Predicted targets for the sRNAs induced during iron starvation, i.e. NC-181, NC-1321, NC-265, and NC-350 (*r*: spearman correlation coefficient)

NC-181 Targets	Description	Energy score	*r*	3 h	12 h	24 h	48 h	72 h	*q*-value
slr1920	unknown protein	-10.19	-0.98	1.50	0.80	0.92	0.63	0.73	$1.74{\cdot}10^{-6}$
sll0017	glutamate-1-semialdehyde aminomutase	-11.52	-0.96	1.83	1.07	1.12	0.76	1.04	$3.53{\cdot}10^{-6}$
ssr0330	ferredoxin-thioredoxin reductase	-12.00	-0.95	1.12	0.71	0.62	0.32	0.46	$2.18{\cdot}10^{-6}$
slr1516	superoxide dismutase	-11.42	-0.94	-0.27	-1.42	-1.06	-1.88	-1.58	$8.29{\cdot}10^{-8}$
sll1029	carbon dioxide concentrating mechanism protein	-14.12	-0.93	1.05	0.36	0.10	-0.43	-0.42	$2.70{\cdot}10^{-7}$
slr0708	periplasmic protein, function unknown	-11.29	-0.92	0.06	-1.03	-1.36	-1.03	-1.11	$2.24{\cdot}10^{-6}$
sll1323	ATP synthase subunit b' of CF(0)	-10.48	-0.91	1.16	0.47	-0.03	-0.45	-0.71	$6.72{\cdot}10^{-8}$
ssl0020	ferredoxin I	-20.56	-0.90	0.33	-1.57	-0.82	-2.72	-2.40	$9.52{\cdot}10^{-9}$
slr1165	sulfate adenylyltransferase	-13.33	-0.90	1.04	0.56	0.38	-0.03	-0.20	$2.68{\cdot}10^{-6}$
sll1326	ATP synthase alpha chain	-18.82	-0.90	1.05	0.46	-0.20	-0.54	-0.64	$1.39{\cdot}10^{-7}$
sll1119	hypothetical protein	-13.29	-0.89	1.50	0.84	0.62	0.11	0.68	$3.64{\cdot}10^{-7}$
slr1200	urea transport system permease protein	-14.39	-0.89	1.79	1.01	0.93	-0.07	0.47	$9.96{\cdot}10^{-8}$
slr0447	ABC-type urea transport system substrate-binding protein	-10.84	-0.88	2.15	0.84	0.90	-0.87	-0.57	$9.16{\cdot}10^{-8}$
slr1281	NADH dehydrogenase subunit I	-10.82	-0.88	-1.01	-1.32	-1.62	-1.96	-1.73	$1.20{\cdot}10^{-7}$
sll0108	ammonium/methylammonium permease	-14.15	-0.87	2.01	1.41	0.89	0.05	0.36	$7.15{\cdot}10^{-8}$
slr1329	ATP synthase beta subunit	-11.50	-0.86	1.61	1.33	1.14	0.70	0.82	$2.46{\cdot}10^{-5}$
sll1069	3-oxoacyl-[acyl-carrier-protein] synthase II	-11.35	-0.86	1.19	0.96	0.70	0.50	0.34	$4.15{\cdot}10^{-7}$
slr1166	UDP-glucose:tetrahydrobiopterin glucosyltransferase	-10.62	-0.86	1.01	0.45	0.55	0.36	-0.14	$6.82{\cdot}10^{-5}$
slr1431	hypothetical protein	-12.04	-0.84	1.24	0.94	0.89	0.38	0.30	$1.37{\cdot}10^{-6}$
slr0434	elongation factor P	-10.02	-0.83	1.12	0.90	0.64	0.18	0.15	$8.13{\cdot}10^{-7}$
slr1945	2,3-bisphosphoglycerate-independent phosphoglycerate mutase	-13.87	-0.82	1.26	0.93	0.60	-0.19	-0.09	$3.76{\cdot}10^{-5}$
slr1280	NADH dehydrogenase subunit	-25.97	-0.81	-0.90	-1.03	-1.27	-1.64	-1.54	$2.35{\cdot}10^{-5}$
slr1986	allophycocyanin beta subunit	-11.93	-0.81	1.31	1.01	0.07	0.02	-0.37	$3.19{\cdot}10^{-7}$
ssr3383	phycobilisome small core linker polypeptide	-10.86	-0.80	1.53	1.21	0.15	0.03	-0.45	$2.09{\cdot}10^{-7}$
sll0421	adenylosuccinate lyase	-10.50	-0.80	1.24	0.98	0.83	0.27	0.12	$3.72{\cdot}10^{-7}$
ssr0390	photosystem I reaction center subunit X	-10.86	-0.80	1.08	0.85	-0.12	-0.30	-0.44	$4.44{\cdot}10^{-8}$
sll0630	unknown protein	-10.47	-0.80	1.14	0.85	0.27	-0.46	-0.60	$1.58{\cdot}10^{-7}$
sll0374	urea transport system ATP-binding	-11.58	-0.79	1.11	0.90	0.75	0.10	0.09	$7.28{\cdot}10^{-7}$

	protein								
slr1853	carboxymuconolactone decarboxylase	-11.96	-0.78	1.12	0.89	-0.36	-0.38	-0.68	$2.53 \cdot 10^{-8}$
slr1756	glutamate--ammonia ligase	-11.53	-0.78	2.53	2.20	1.73	1.19	1.85	$6.69 \cdot 10^{-8}$
slr1279	NADH dehydrogenase subunit 3	-13.60	-0.76	-1.17	-1.29	-1.67	-2.29	-2.14	$2.16 \cdot 10^{-5}$
sll1942	unknown protein	-14.01	-0.75	1.22	1.09	0.98	0.47	0.39	$1.83 \cdot 10^{-5}$
slr1351	UDP-N-acetylmuramoylalanyl-D-glutamyl-2 6-diaminopimelate--D-alanyl-D-alanine ligase	-11.68	-0.74	-0.37	-0.94	-0.44	-0.99	-1.23	$1.02 \cdot 10^{-5}$
slr0338	probable oxidoreductase	-14.22	-0.74	1.30	1.21	1.04	0.50	0.60	$5.10 \cdot 10^{-7}$
sll1398	photosystem II reaction center 13 kDa protein	-10.31	-0.73	1.57	1.56	1.04	0.86	0.92	$1.22 \cdot 10^{-6}$
sll1281	photosystem II PsbZ protein	-12.93	-0.73	1.25	0.86	0.07	-0.05	0.66	$1.41 \cdot 10^{-7}$
slr0993	putative peptidase, lipoprotein NlpD	-16.05	-0.72	1.21	1.02	0.83	-0.38	-0.26	$3.73 \cdot 10^{-5}$
slr0423	hypothetical protein	-12.88	-0.72	1.15	1.07	0.85	0.28	0.59	$4.81 \cdot 10^{-7}$
ssr1600	similar to anti-sigma f factor antagonist	-13.62	-0.70	1.38	1.41	0.78	0.67	0.63	$9.18 \cdot 10^{-8}$
slr1187	unknown protein	-12.09	-0.70	1.00	1.02	0.62	0.13	0.04	$2.14 \cdot 10^{-6}$
slr1437	unknown protein	-12.40	-0.67	-0.34	-0.55	-1.76	-1.43	-1.13	$2.86 \cdot 10^{-4}$
sll1185	coproporphyrinogen III oxidase, aerobic (oxygen-dependent)	-11.60	-0.61	1.76	1.59	0.09	0.85	0.81	$4.21 \cdot 10^{-8}$
sll1242	hypothetical protein	-12.74	-0.56	1.29	1.46	0.92	0.72	0.89	$1.03 \cdot 10^{-5}$
slr1545	RNA polymerase ECF-type (group 3) sigma-E factor	-11.73	-0.54	-0.29	-0.19	-0.32	-0.73	-1.10	$9.86 \cdot 10^{-7}$
ssl2982	probable DNA-directed RNA polymerase omega subunit	-10.35	-0.50	1.14	1.17	0.74	0.80	1.02	$1.21 \cdot 10^{-5}$

**NC-1321 Targets**	**Description**	**Energy score**	***r***	**3 h**	**12 h**	**24 h**	**48 h**	**72 h**	***q*-value**
slr0923	hypothetical protein YCF65	-11.04	-0.53	1.46	1.40	0.79	0.55	0.70	$2.84 \cdot 10^{-7}$
slr1887	porphobilinogen deaminase	-12.39	-0.57	1.23	1.07	0.64	0.46	0.61	$2.50 \cdot 10^{-5}$
sll0615	hypothetical protein	-10.50	-0.60	-0.36	-1.83	-0.53	-0.74	-1.02	$2.03 \cdot 10^{-6}$
ssl0410	unknown protein	-10.04	-0.62	-0.46	-0.57	-0.86	-1.14	-0.93	$2.64 \cdot 10^{-5}$
slr1908	probable porin	-11.79	-0.63	1.13	1.21	0.89	0.64	0.34	$2.65 \cdot 10^{-7}$
slr1364	biotin synthetase	-10.67	-0.83	1.09	0.82	0.73	0.41	0.50	$5.53 \cdot 10^{-5}$

**NC-265 Targets**	**Description**	**Energy score**	***r***	**3 h**	**12 h**	**24 h**	**48 h**	**72 h**	***q*-value**
slr2002	cyanophycin synthetase	-10.48	-0.51	1.46	1.02	1.00	0.43	1.10	$2.65 \cdot 10^{-7}$
slr1545	RNA polymerase ECF-type (group 3) sigma-E factor	-11.45	-0.53	-0.29	-0.19	-0.32	-0.73	-1.10	$9.86 \cdot 10^{-7}$
sll1654	hypothetical protein	-14.39	-0.55	-0.46	-0.22	-1.14	-0.36	-0.30	$4.48 \cdot 10^{-3}$
ssr1155	hypothetical protein	-12.68	-0.58	-0.69	-0.19	-0.83	-1.17	-0.70	$1.06 \cdot 10^{-6}$

slr1166	UDP-glucose:tetrahydrobiopterin glucosyltransferase	-10.63	-0.59	1.01	0.45	0.55	0.36	-0.14	$6.82{\cdot}10^{-5}$
slr0993	putative peptidase	-10.66	-0.61	1.21	1.02	0.83	-0.38	-0.26	$3.73{\cdot}10^{-5}$
slr0559	periplasmic binding protein of ABC transporter for natural amino acids	-11.17	-0.62	1.05	0.47	0.57	0.04	0.07	$6.07{\cdot}10^{-6}$
sll1583	unknown protein	-10.76	-0.66	0.53	1.02	-0.28	0.54	0.64	$1.78{\cdot}10^{-3}$
slr0338	probable oxidoreductase	-10.77	-0.67	1.30	1.21	1.04	0.50	0.60	$5.10{\cdot}10^{-7}$
sll1270	Bgt permease for basic amino acids and glutamine BgtB	-11.60	-0.70	1.62	1.24	0.86	-0.30	0.02	$1.10{\cdot}10^{-7}$
slr1364	biotin synthetase	-10.07	-0.73	1.09	0.82	0.73	0.41	0.50	$5.53{\cdot}10^{-5}$
slr1165	sulfate adenylyltransferase	-10.14	-0.76	1.04	0.56	0.38	-0.03	-0.20	$2.68{\cdot}10^{-6}$
slr1622	soluble inorganic pyrophosphatase	-12.83	-0.76	1.55	1.15	0.95	0.46	0.60	$4.94{\cdot}10^{-7}$
sll1029	carbon dioxide concentrating mechanism protein	-13.01	-0.78	1.05	0.36	0.10	-0.43	-0.42	$2.70{\cdot}10^{-7}$
sll1342	NAD(P)-dependent glyceraldehyde-3-phosphate dehydrogenase	-17.09	-0.81	1.45	1.22	1.02	0.76	0.92	$1.68{\cdot}10^{-5}$
sll1898	hypothetical protein	-10.35	-0.82	0.52	1.20	-0.21	-0.59	-0.48	$3.90{\cdot}10^{-8}$
sll1069	3-oxoacyl-[acyl-carrier-protein] synthase II	-10.69	-0.83	1.19	0.96	0.70	0.50	0.34	$4.15{\cdot}10^{-7}$
slr1847	hypothetical protein	-10.61	-0.85	1.37	1.40	0.91	0.61	0.67	$2.94{\cdot}10^{-7}$
slr1708	probable peptidase	-11.79	-0.86	1.23	1.20	0.64	0.38	0.22	$8.49{\cdot}10^{-6}$
slr0348	hypothetical protein	-11.55	-0.86	1.09	1.02	0.85	0.72	0.78	$3.82{\cdot}10^{-7}$
ssl2982	probable DNA-directed RNA polymerase omega subunit	-15.71	-0.87	1.14	1.17	0.74	0.80	1.02	$1.21{\cdot}10^{-5}$
sll1326	ATP synthase alpha chain	-11.26	-0.88	1.05	0.46	-0.20	-0.54	-0.64	$1.39{\cdot}10^{-7}$
slr1020	sulfolipid biosynthesis protein	-11.94	-0.88	1.09	0.81	0.48	0.30	0.29	$9.50{\cdot}10^{-6}$
sll1398	photosystem II reaction center 13 kDa protein	-17.30	-0.91	1.57	1.56	1.04	0.86	0.92	$1.22{\cdot}10^{-6}$
slr1887	porphobilinogen deaminase (hydroxymethylbilane synthase, preuroporphyrinogen synthase)	-20.08	-0.91	1.23	1.07	0.64	0.46	0.61	$2.50{\cdot}10^{-5}$
ssl1498	hypothetical protein	-10.87	-0.93	1.07	0.81	0.03	-0.16	-0.23	$3.30{\cdot}10^{-7}$
ssr1600	similar to anti-sigma f factor antagonist	-11.31	-0.93	1.38	1.41	0.78	0.67	0.63	$9.18{\cdot}10^{-8}$
sll1091	geranylgeranyl hydrogenase	-10.44	-0.95	1.39	1.51	-0.23	0.28	0.59	$3.29{\cdot}10^{-8}$
slr0335	phycobilisome core-membrane linker polypeptide	-10.80	-0.99	1.26	1.01	-0.02	0.20	0.05	$3.25{\cdot}10^{-7}$

NC-350 Targets	Description	Energy score	*r*	3 h	12 h	24 h	48 h	72 h	*q*
slr1166	UDP-glucose:tetrahydrobiopterin glucosyltransferase	-11.28	-0.91	1.01	0.45	0.55	0.36	-0.14	$6.82{\cdot}10^{-5}$

slr1215	hypothetical protein	-11.63	-0.80	-0.78	-1.44	-0.73	-1.16	-1.24	$6.12\cdot10^{-7}$
slr1835	P700 apoprotein subunit Ib	-10.43	-0.75	0.34	-0.41	-0.54	-0.96	-1.16	$4.26\cdot10^{-8}$
slr0559	periplasmic binding protein of ABC transporter for natural amino acids	-11.36	-0.75	1.05	0.47	0.57	0.04	0.07	$6.07\cdot10^{-6}$
slr0676	adenylylsulfate kinase	-10.15	-0.74	1.26	0.97	0.98	0.33	0.26	$8.03\cdot10^{-7}$
sll0421	adenylosuccinate lyase	-12.55	-0.71	1.24	0.98	0.83	0.27	0.12	$3.72\cdot10^{-7}$
slr0447	periplasmic protein, ABC-type urea transport system substrate-binding protein	-10.55	-0.69	2.15	0.84	0.90	-0.87	-0.57	$9.16\cdot10^{-8}$
sll0262	acyl-lipid desaturase (delta 6)	-15.39	-0.66	1.11	0.89	0.72	0.13	0.09	$4.87\cdot10^{-5}$
sll1069	3-oxoacyl-[acyl-carrier-protein] synthase II	-12.79	-0.66	1.19	0.96	0.70	0.50	0.34	$4.15\cdot10^{-7}$
sll1029	carbon dioxide concentrating mechanism protein CcmK	-10.12	-0.65	1.05	0.36	0.10	-0.43	-0.42	$2.70\cdot10^{-7}$
slr0879	glycine decarboxylase complex H-protein	-13.00	-0.63	1.21	0.90	0.72	0.40	0.41	$5.73\cdot10^{-7}$
sll0689	Na+/H+ antiporter	-15.07	-0.62	1.17	0.83	0.57	-0.34	-0.25	$2.21\cdot10^{-4}$
slr1096	dihydrolipoamide dehydrogenase	-10.42	-0.61	1.03	0.65	0.51	0.19	0.27	$6.28\cdot10^{-5}$
sll0630	unknown protein	-10.66	-0.60	1.14	0.85	0.27	-0.46	-0.60	$1.58\cdot10^{-7}$
slr1622	soluble inorganic pyrophosphatase	-11.70	-0.59	1.55	1.15	0.95	0.46	0.60	$4.94\cdot10^{-7}$
slr0007	probable sugar-phosphate nucleotidyltransferase	-23.42	-0.57	-1.07	-0.92	-0.97	-1.57	-1.62	$6.41\cdot10^{-7}$
sll1638	hypothetical protein	-13.64	-0.57	1.58	1.38	1.00	0.98	0.79	$1.03\cdot10^{-7}$
ssr3383	phycobilisome small core linker polypeptide	-11.36	-0.55	1.53	1.21	0.15	0.03	-0.45	$2.09\cdot10^{-7}$
slr1020	sulfolipid biosynthesis protein SqdB	-11.71	-0.54	1.09	0.81	0.48	0.30	0.29	$9.50\cdot10^{-6}$
sll0045	sucrose phosphate synthase	-10.09	-0.54	-0.69	-0.59	-0.45	-0.20	-1.04	$7.34\cdot10^{-3}$
slr1986	allophycocyanin beta subunit	-13.06	-0.53	1.31	1.01	0.07	0.02	-0.37	$3.19\cdot10^{-7}$
sll0108	ammonium/methylammonium permease	-20.54	-0.53	2.01	1.41	0.89	0.05	0.36	$7.15\cdot10^{-8}$
sll1452	nitrate/nitrite transport system ATP-binding protein	-11.66	-0.52	1.25	0.59	1.69	0.92	1.10	$8.61\cdot10^{-8}$
