# Supplementary material for: Iron Deprivation in Synechocystis: Inference of Pathways, Non-coding RNAs, and Regulatory Elements from Comprehensive Expression Profiling
Source: G3 (Bethesda). 2012 Dec 1;2(12):1475–95. doi: 10.1534/g3.112.003863 (PMC3516471; doi:10.1534/g3.112.003863)
Supplement: Supporting Information [file supp_2.12.1475_FigureS1.pdf]

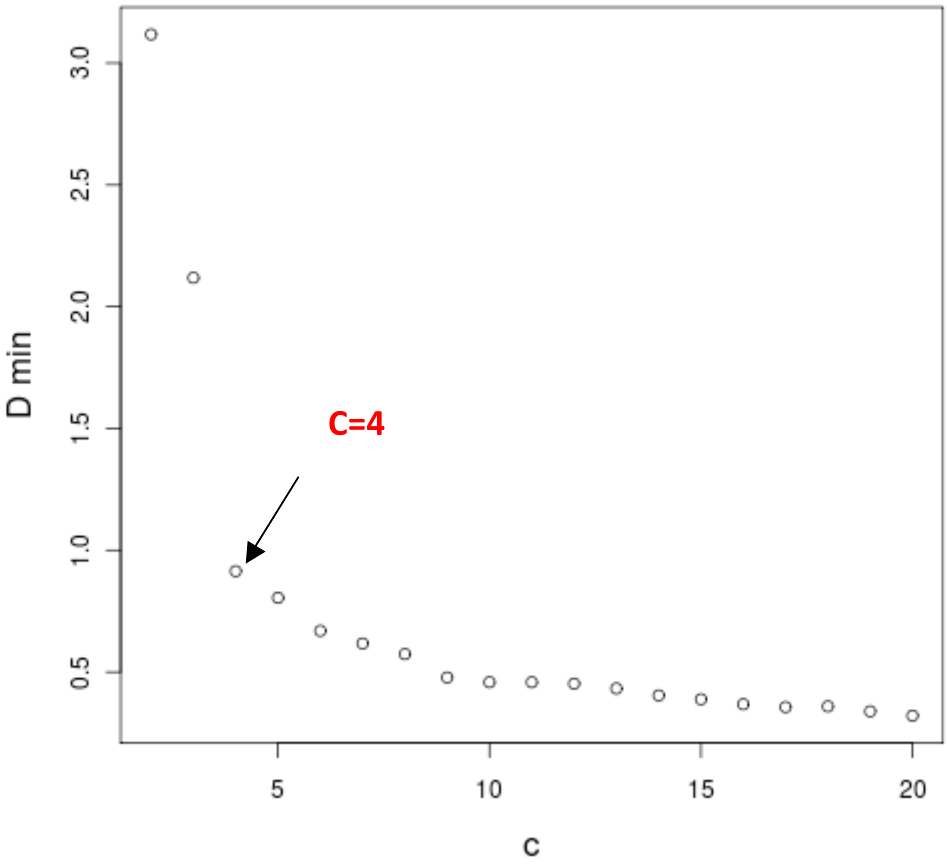

Figure S1 Minimum centroid distance for the variation of FCM parameter c. The expression data set was repeatedly clustered (N=10) for integer values of c from 2 to 20. For FCM clustering, the Bioconductor package *Mfuzz* was employed. After each clustering, the minimum centroid distance was calculated. The plot shows average minimum centroid distance with respect to parameter c. The average minimum centroid distance decreases notably less for c > 4, indicting c=4 as the optimal value.
