# Supplementary material for: Iron Deprivation in Synechocystis: Inference of Pathways, Non-coding RNAs, and Regulatory Elements from Comprehensive Expression Profiling
Source: G3 (Bethesda). 2012 Dec 1;2(12):1475–95. doi: 10.1534/g3.112.003863 (PMC3516471; doi:10.1534/g3.112.003863)
Supplement: Supporting Information [file supp_2.12.1475_FigureS2.pdf]

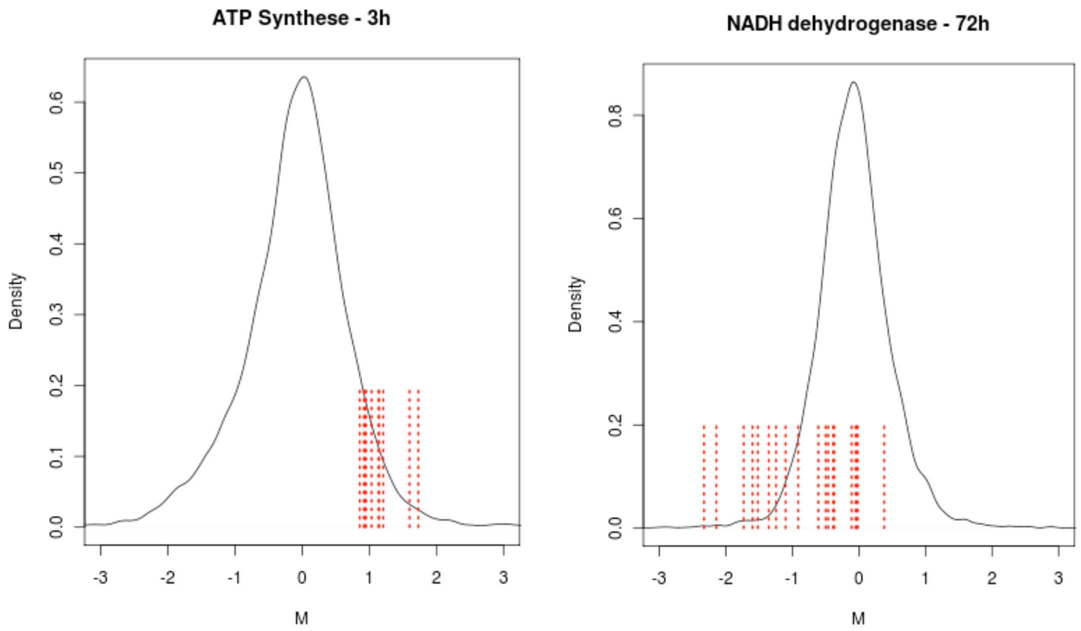

Figure S2 Differentially regulated gene sets detected by PGSEA. The plots show two examples of gene sets that were significant differentially regulated by parametric gene set enrichment analysis (PGSEA). The black line represents the distribution of logged fold changes (M) at time point 3h (left side) and 72h. The dashed red lines indicate the expression changes for genes associated with ATP synthase (left side) and NADH dehydrogenase (right side) in Cyanobase. Genes associated with ATP synthase tend to be up-regulated at time point 3h, whereas genes associated with NADH dehydrogenase tend to be down-regulated at time point 72h.
