# Supplementary material for: Iron Deprivation in Synechocystis: Inference of Pathways, Non-coding RNAs, and Regulatory Elements from Comprehensive Expression Profiling
Source: G3 (Bethesda). 2012 Dec 1;2(12):1475–95. doi: 10.1534/g3.112.003863 (PMC3516471; doi:10.1534/g3.112.003863)
Supplement: Supporting Information [file supp_2.12.1475_FigureS3.pdf]

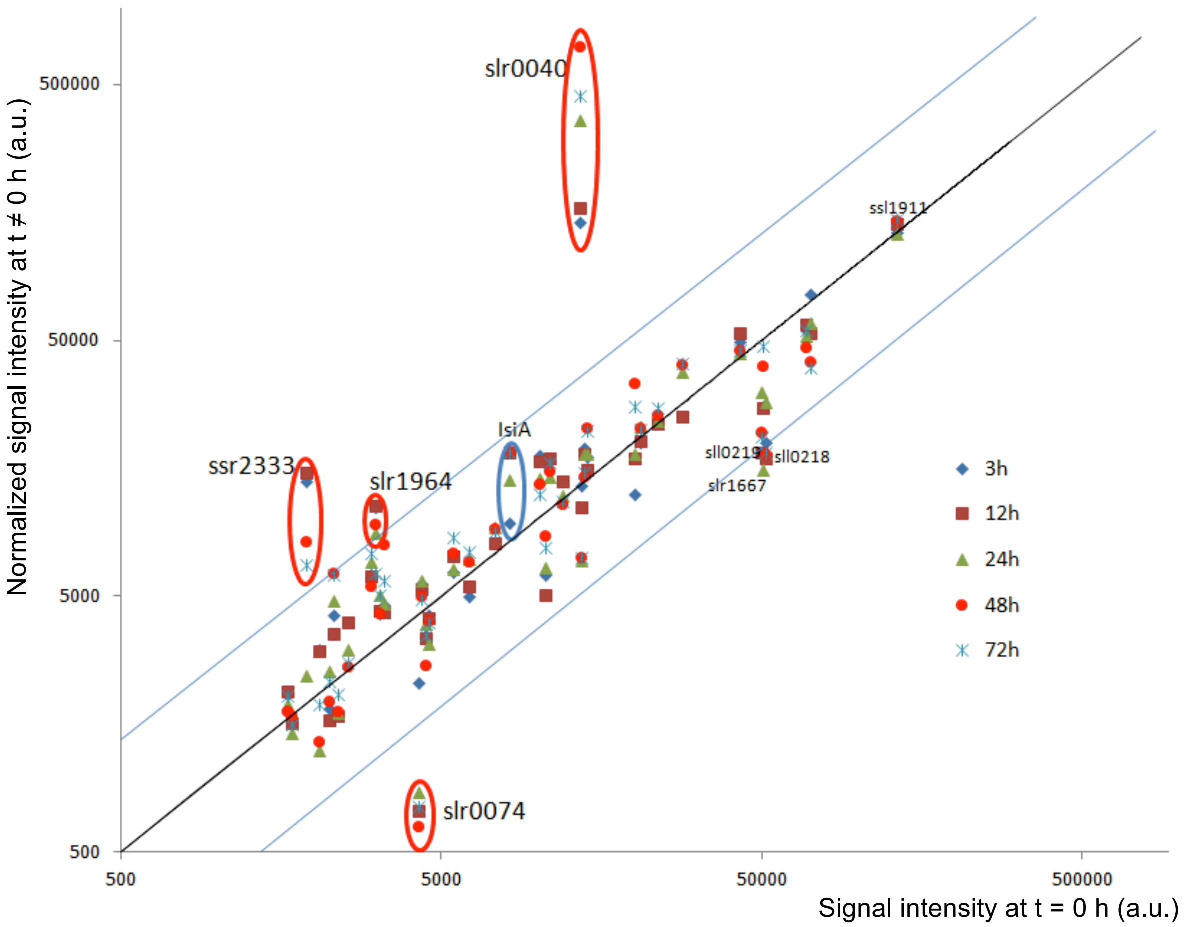

Normalized signal intensity at t ≠ 0 h (a.u.)
Signal intensity at t = 0 h (a.u.)
500000
50000
5000
500
500
5000
50000
500000
slr0040
ssl1911
IsiA
ssr2333
slr1964
sll0219
sll0218
slr1667
slr0074
3h
12h
24h
48h
72h


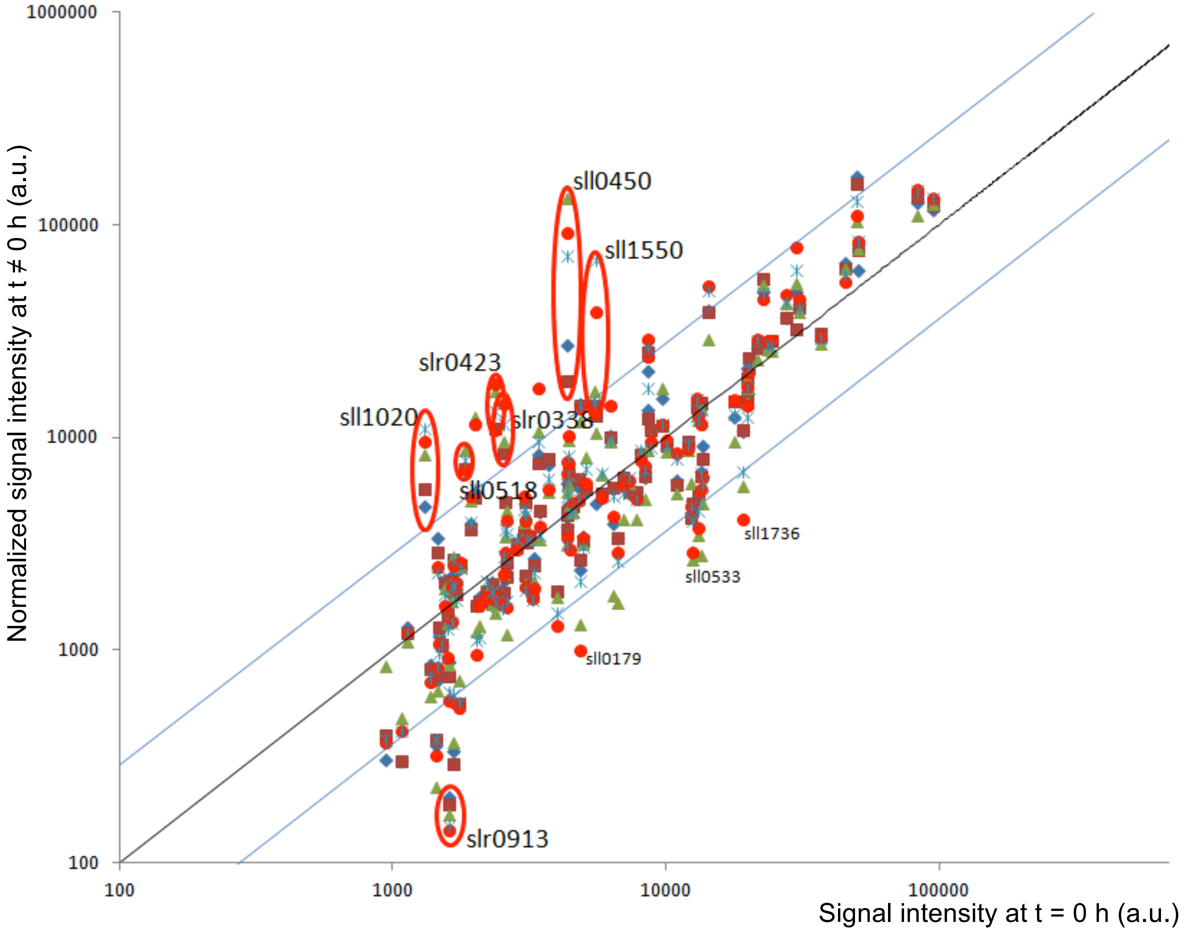

Normalized signal intensity at t ≠ 0 h (a.u.)
Signal intensity at t = 0 h (a.u.)
1000000
100000
10000
1000
100
100
1000
10000
100000
sll0450
sll1550
slr0423
sll1020
slr0338
sll0518
sll1736
sll0533
sll0179
slr0913

**Figure S3** Expression of protein-coding genes with respect to the corresponding 5'UTRs and intragenic elements. To determine whether the differences in signal intensity detected between 5'UTR or intragenic elements with respect to the main gene were due to differences in transcript levels or were simply artifacts caused by different probe affinity, the signals were normalized as follows: We used the ratio obtained from dividing the intensity of (A) the 5'UTR or (B) intragenic element at each time point (Xt) by their intensity at time point 0h (X0h) to normalize the gene intensity at each sampled time (Yt). As a result, when plotting the gene intensity at time 0h (Y0h in the X axis) against its normalized signal intensity at each time point (e.g. [(X0h/X3h)*Y3h]), pairs where transcripts vary in a similar manner will fall around a X=Y central line, while pairs in which the 5'UTR or intragenic element is regulated differently to the gene will fall over or under it (red-circled genes). We have marked only those pairs for which all time points were over the set threshold as differently regulated.
