# Supplementary figures and images for: Iron Deprivation in Synechocystis: Inference of Pathways, Non-coding RNAs, and Regulatory Elements from Comprehensive Expression Profiling
Source: G3 (Bethesda). 2012 Dec 1;2(12):1475–95. doi: 10.1534/g3.112.003863 (PMC3516471; doi:10.1534/g3.112.003863)

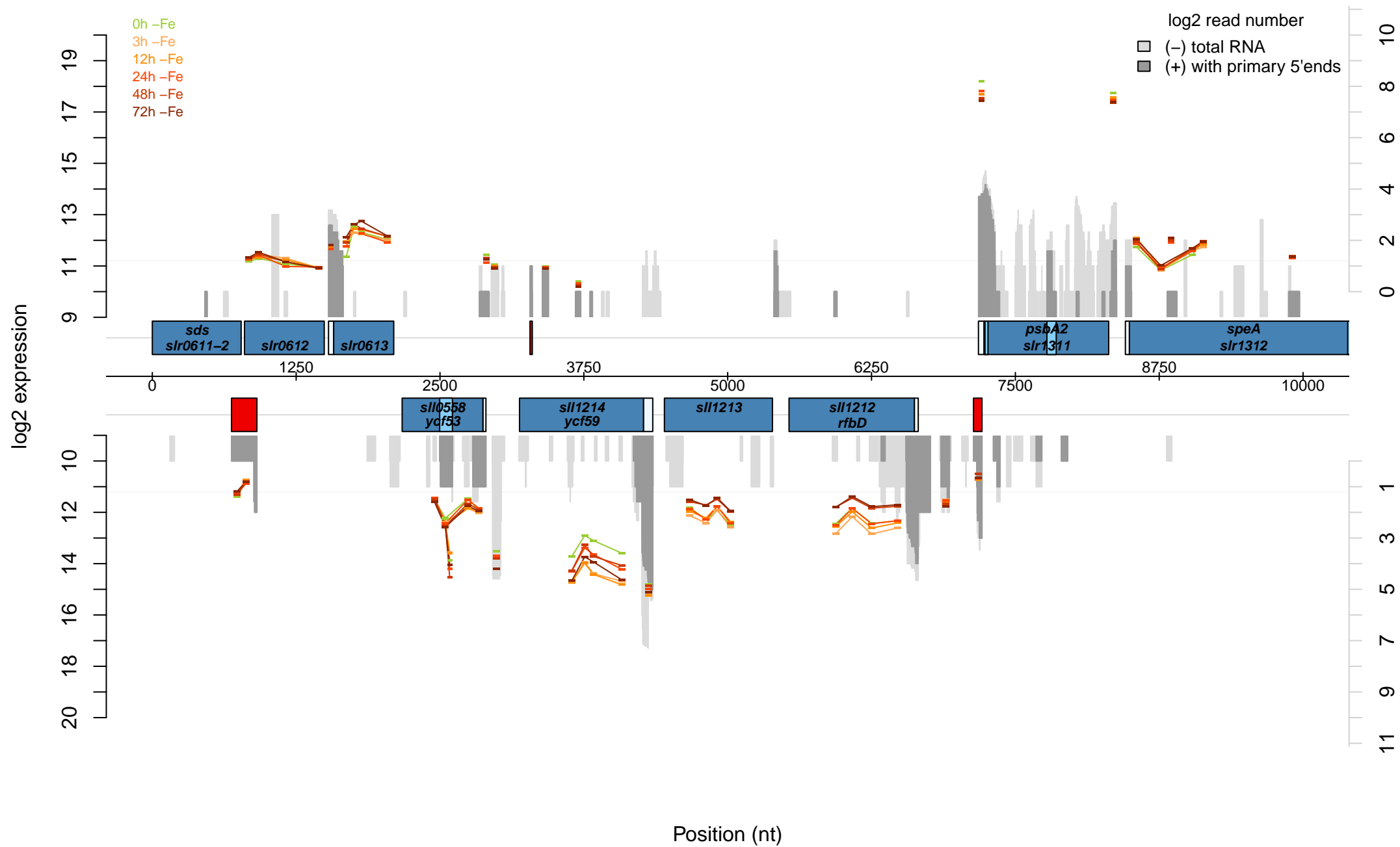

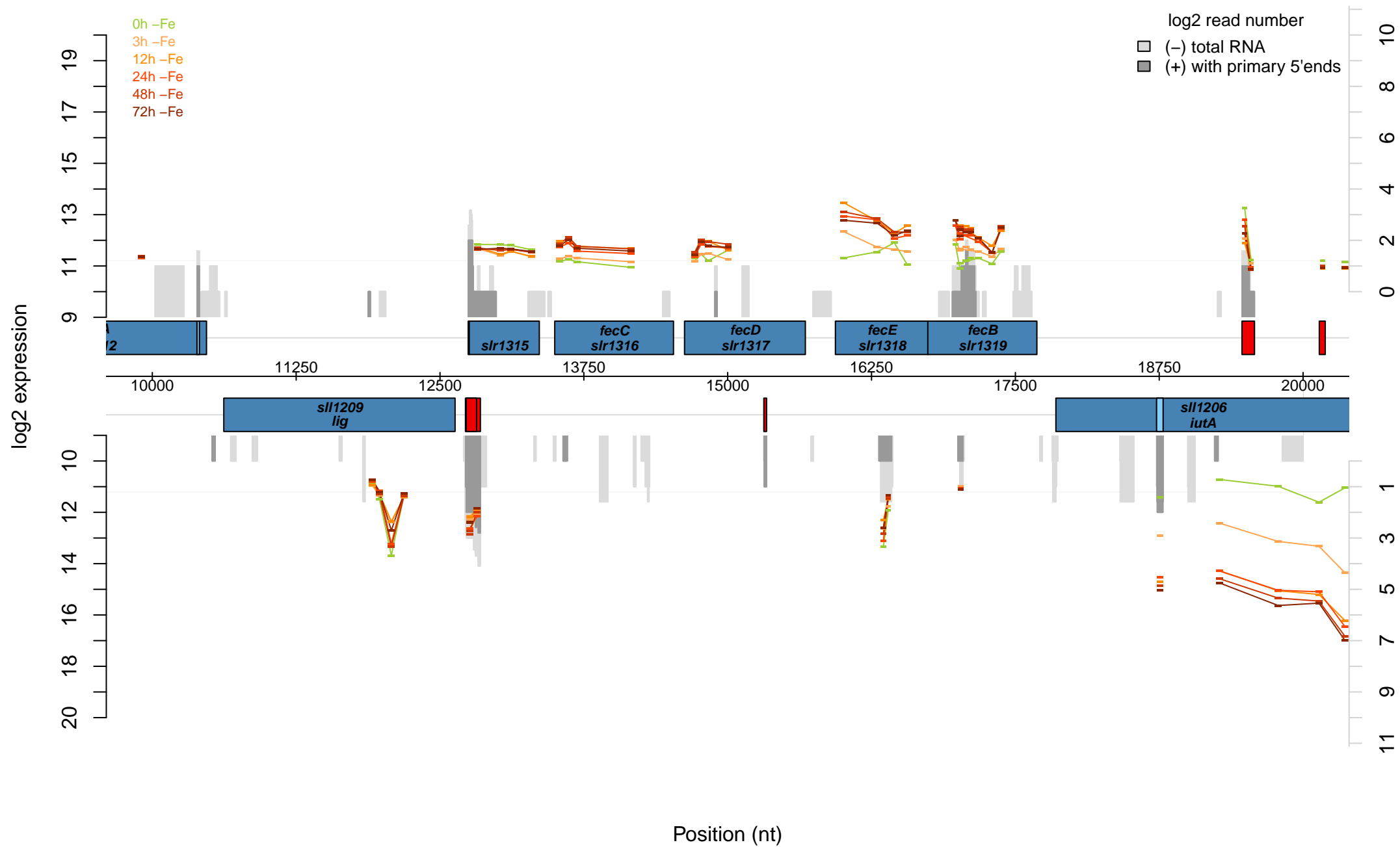

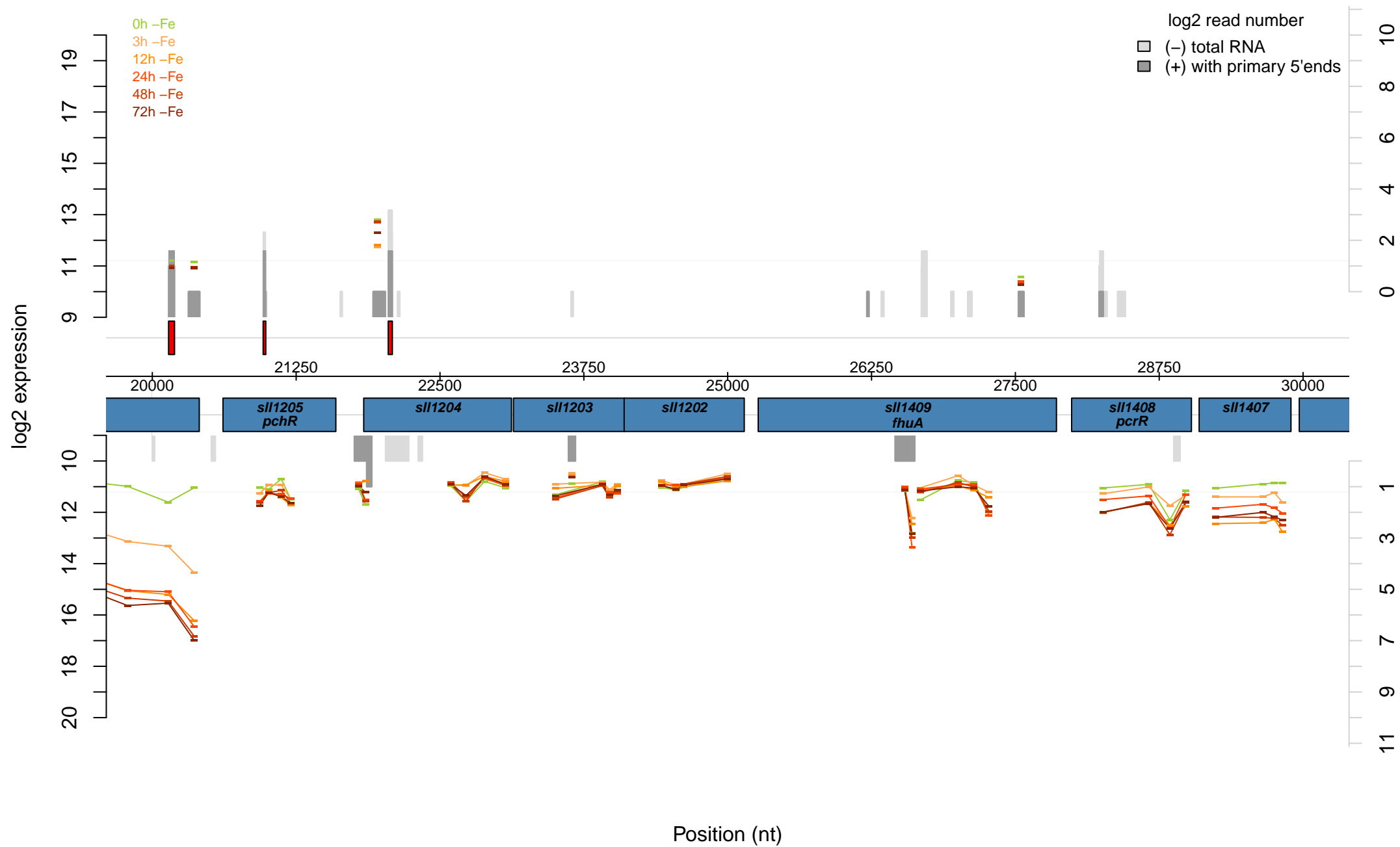

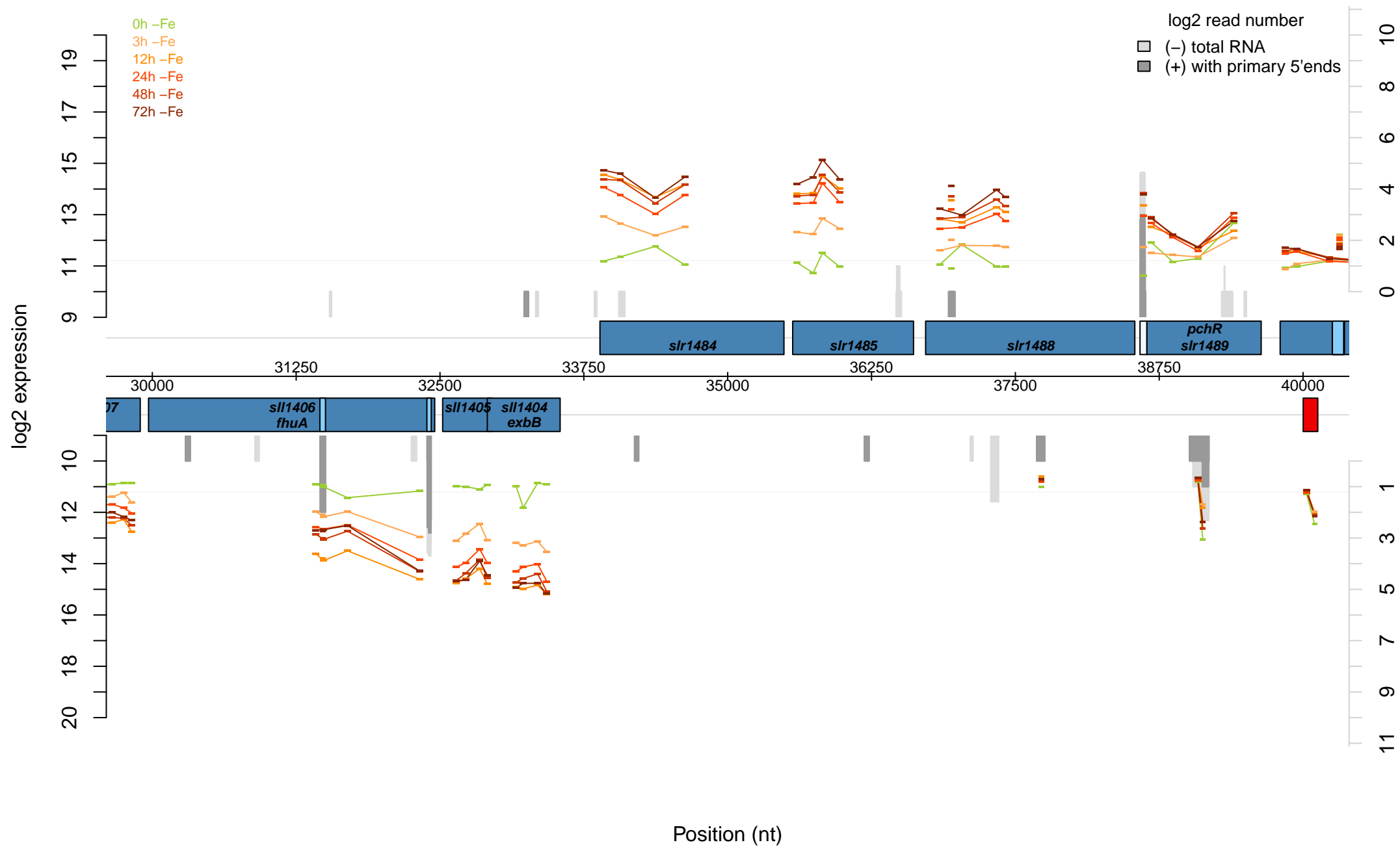

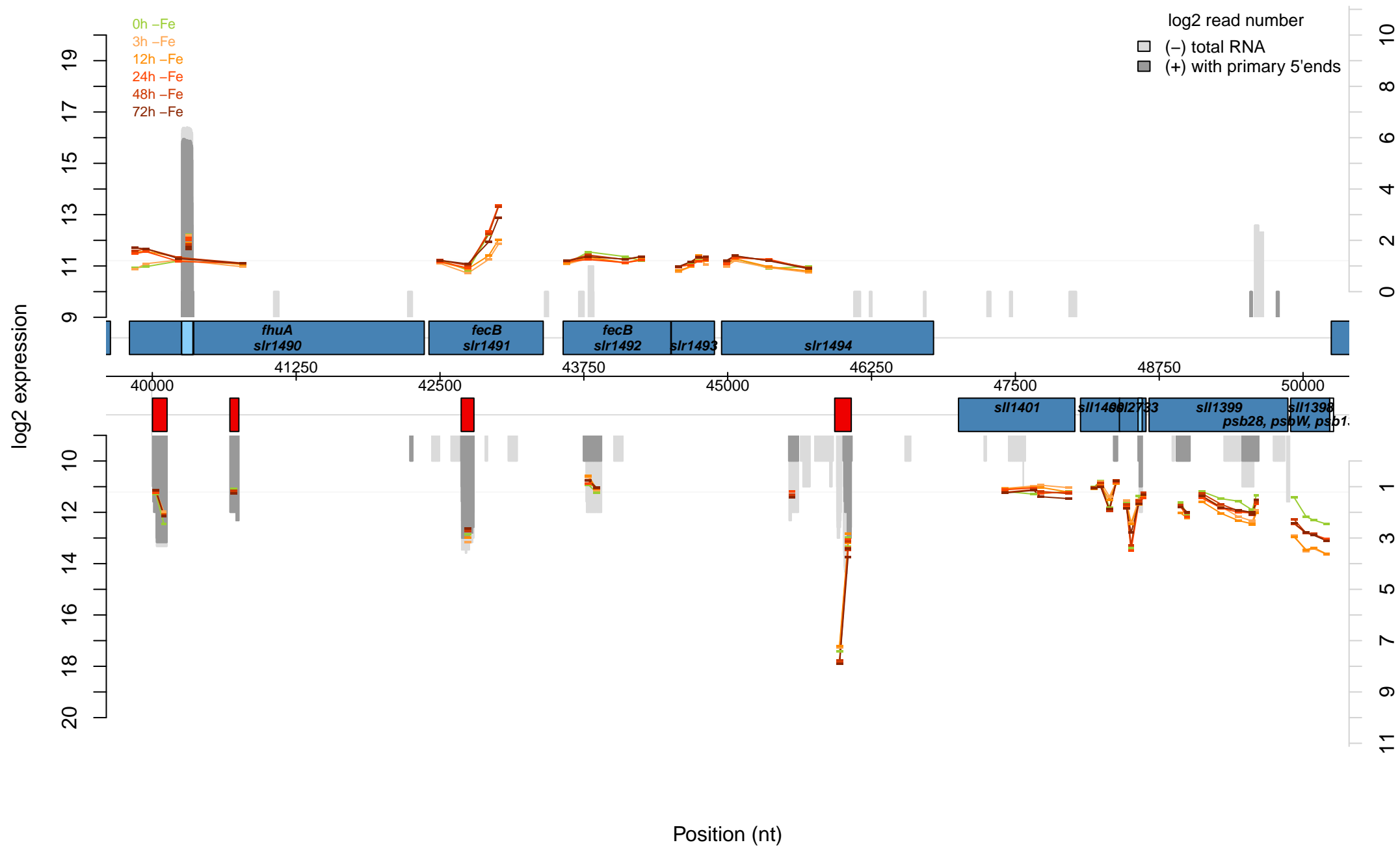

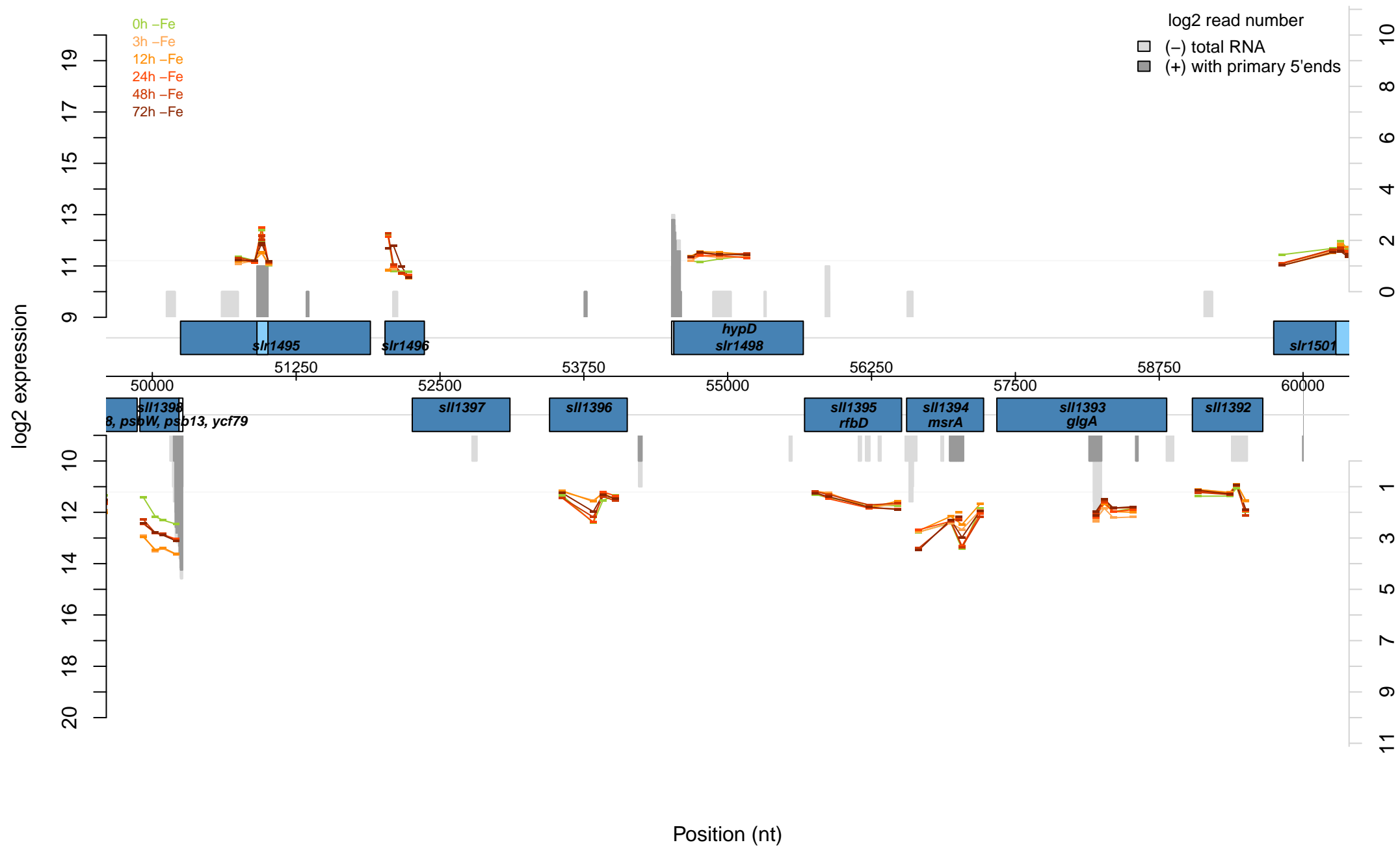

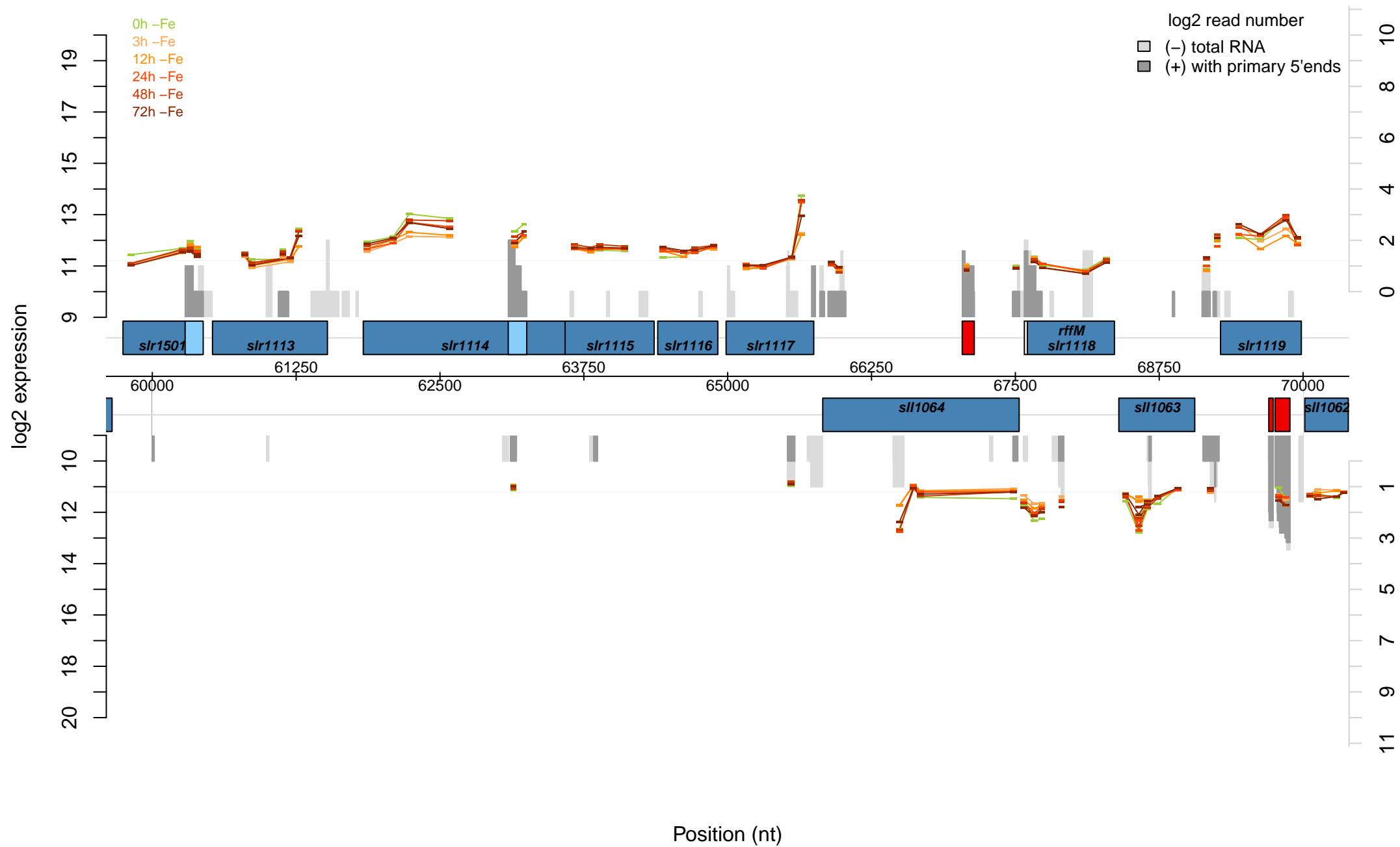

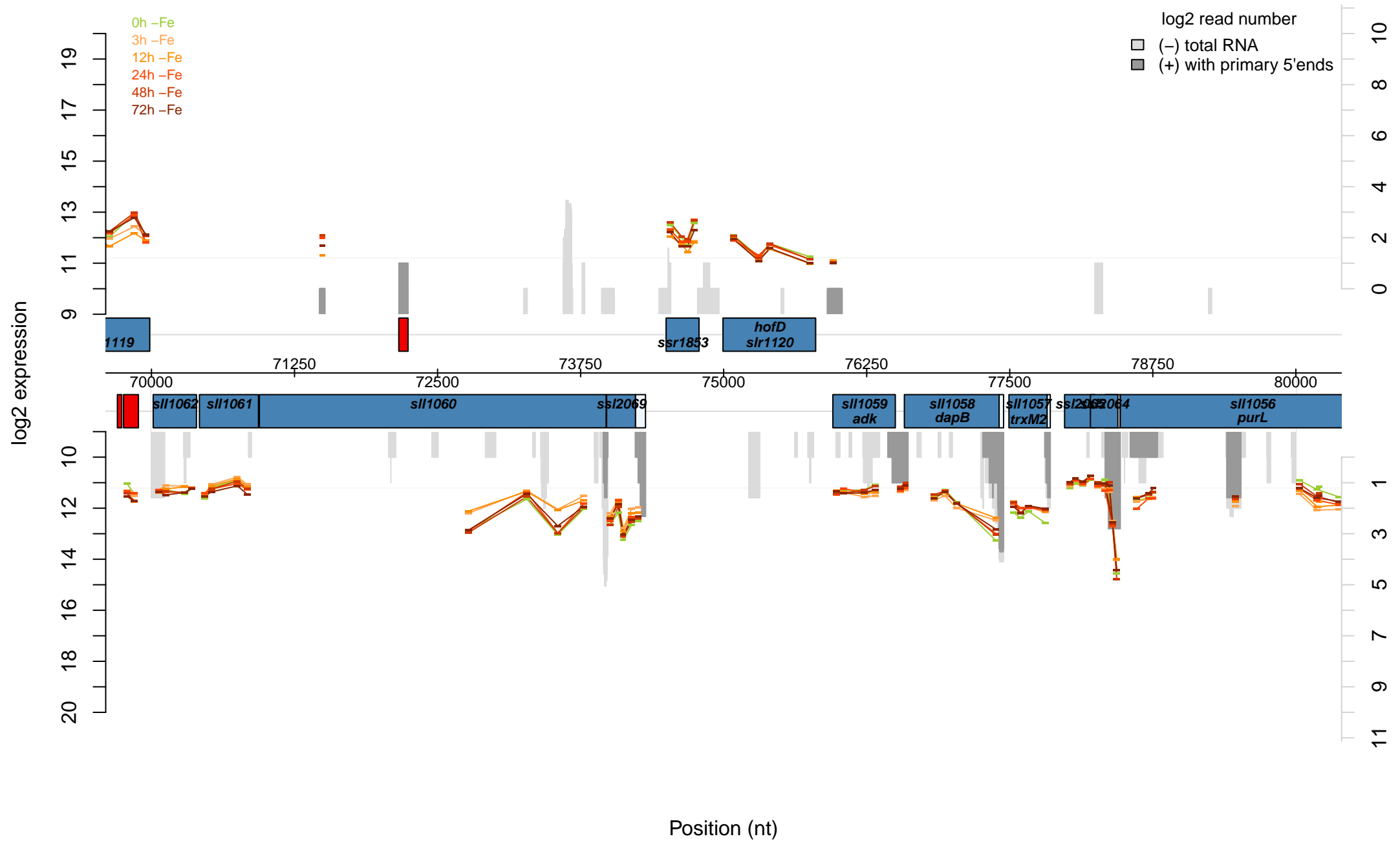

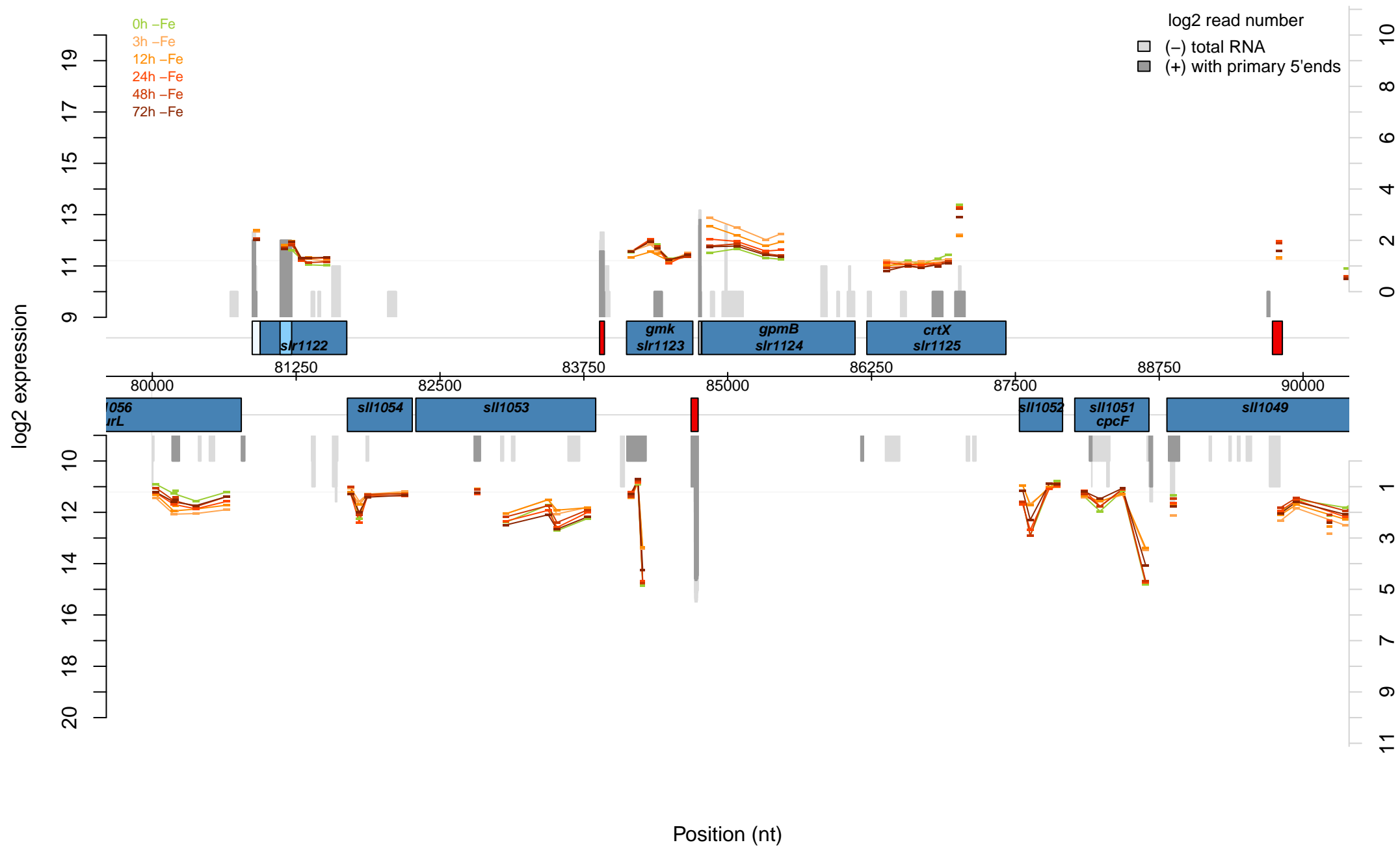

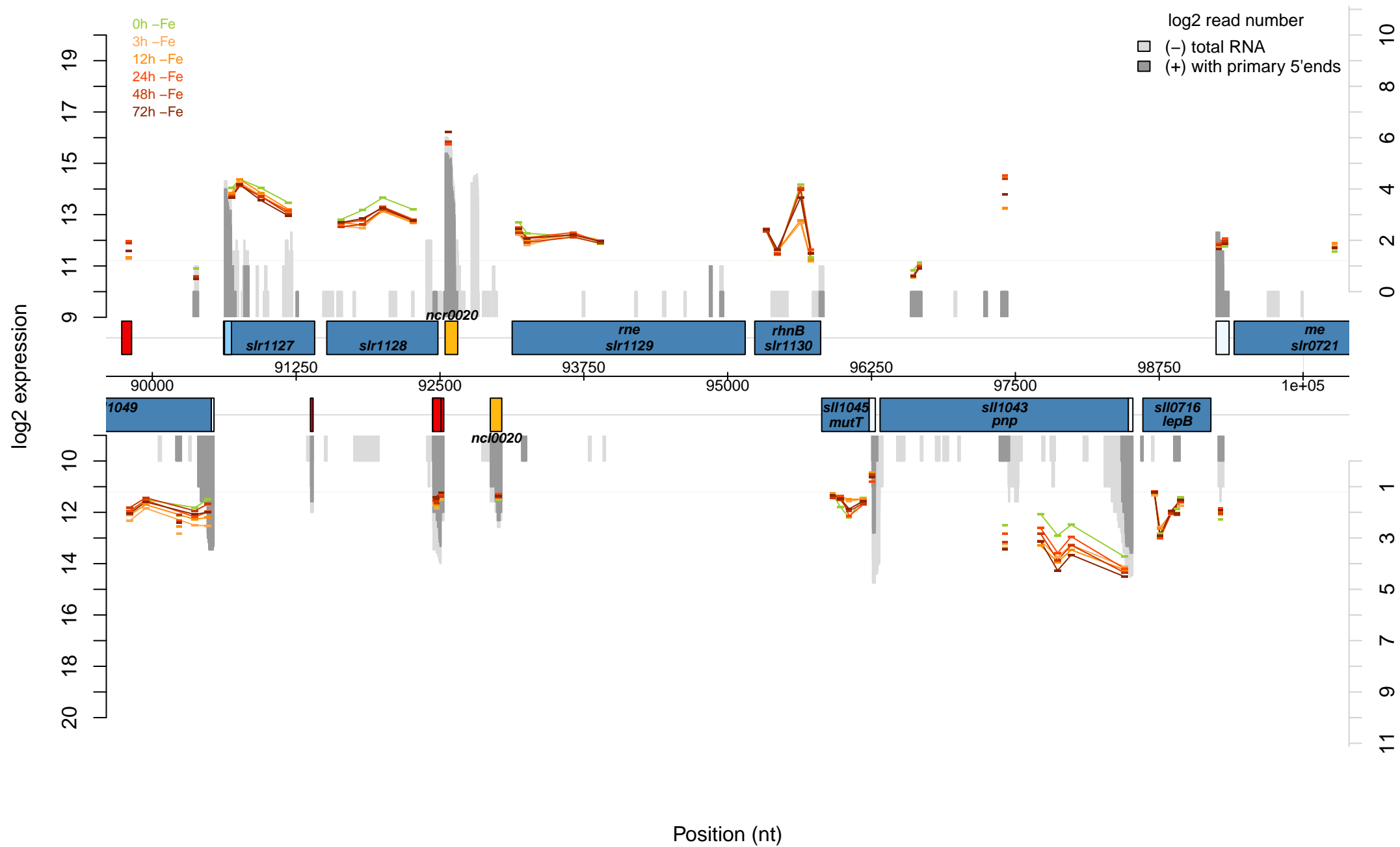

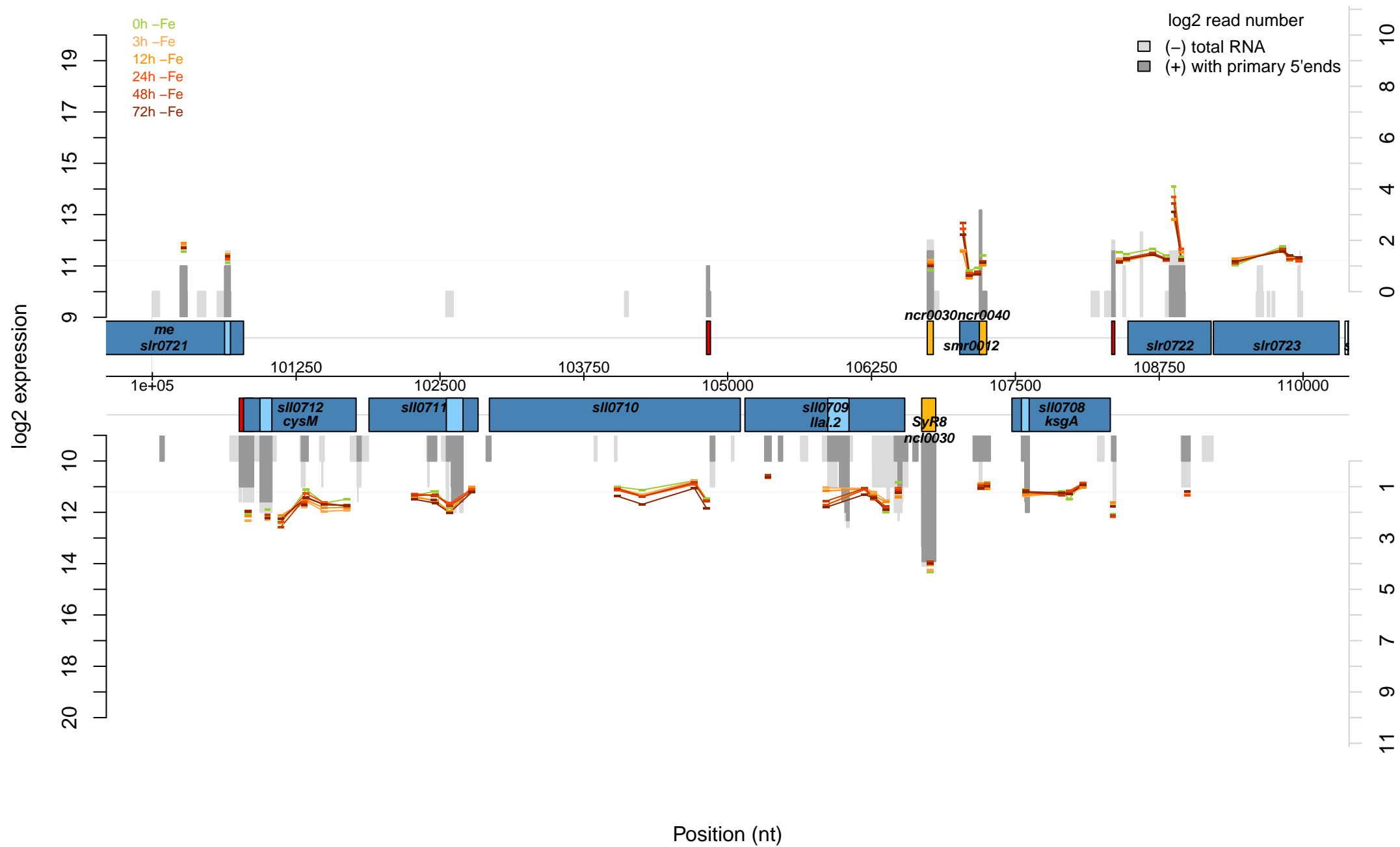

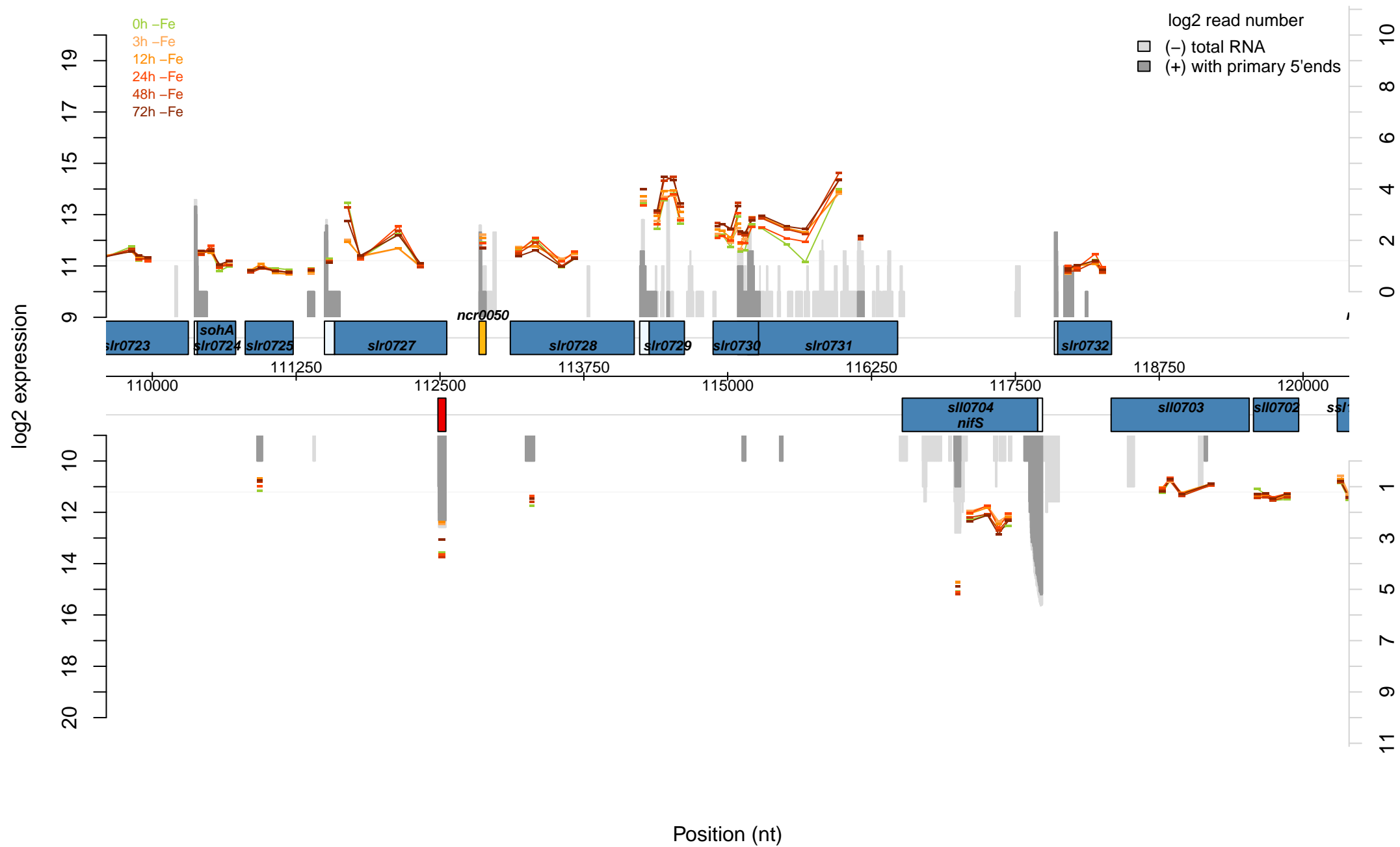

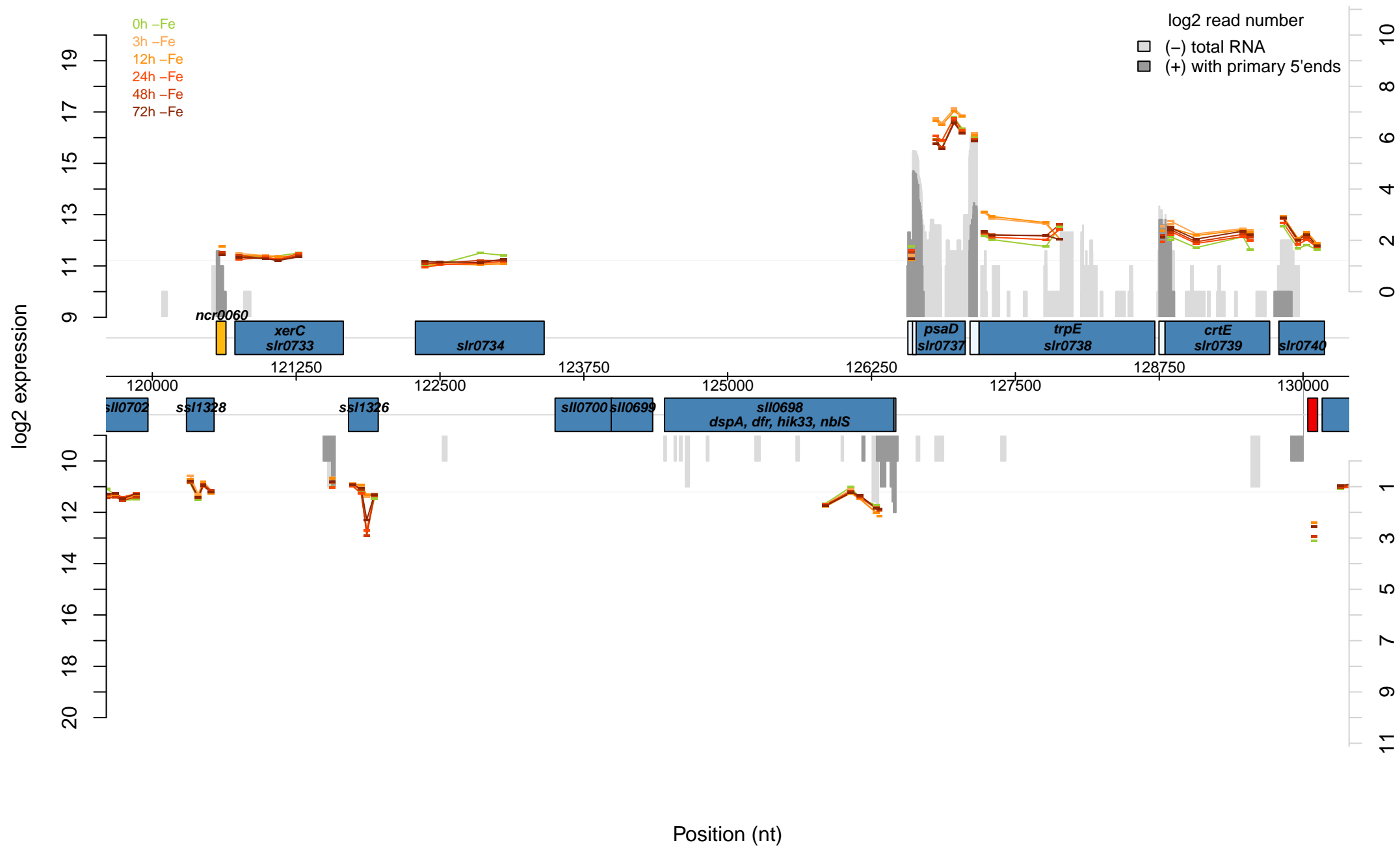

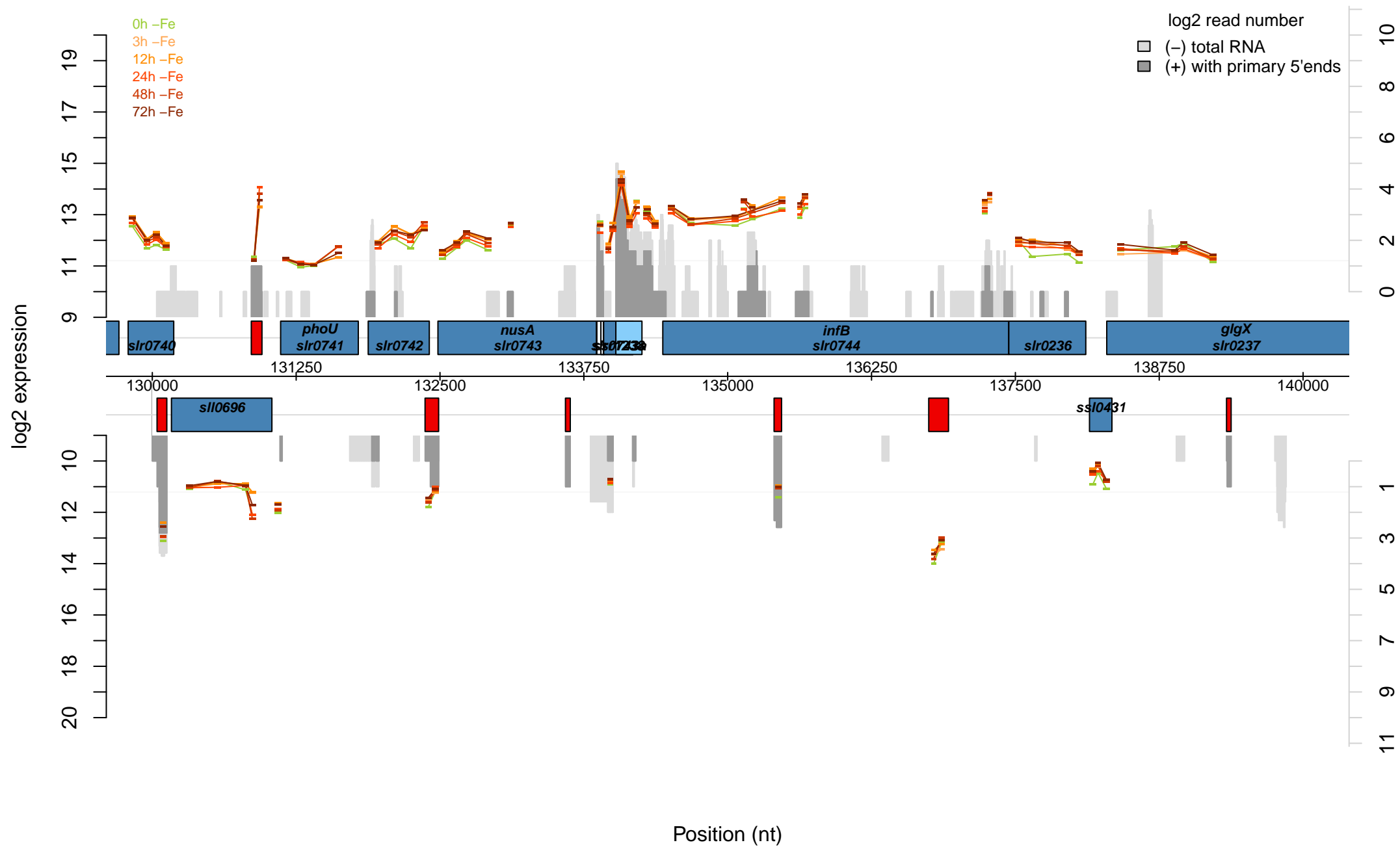

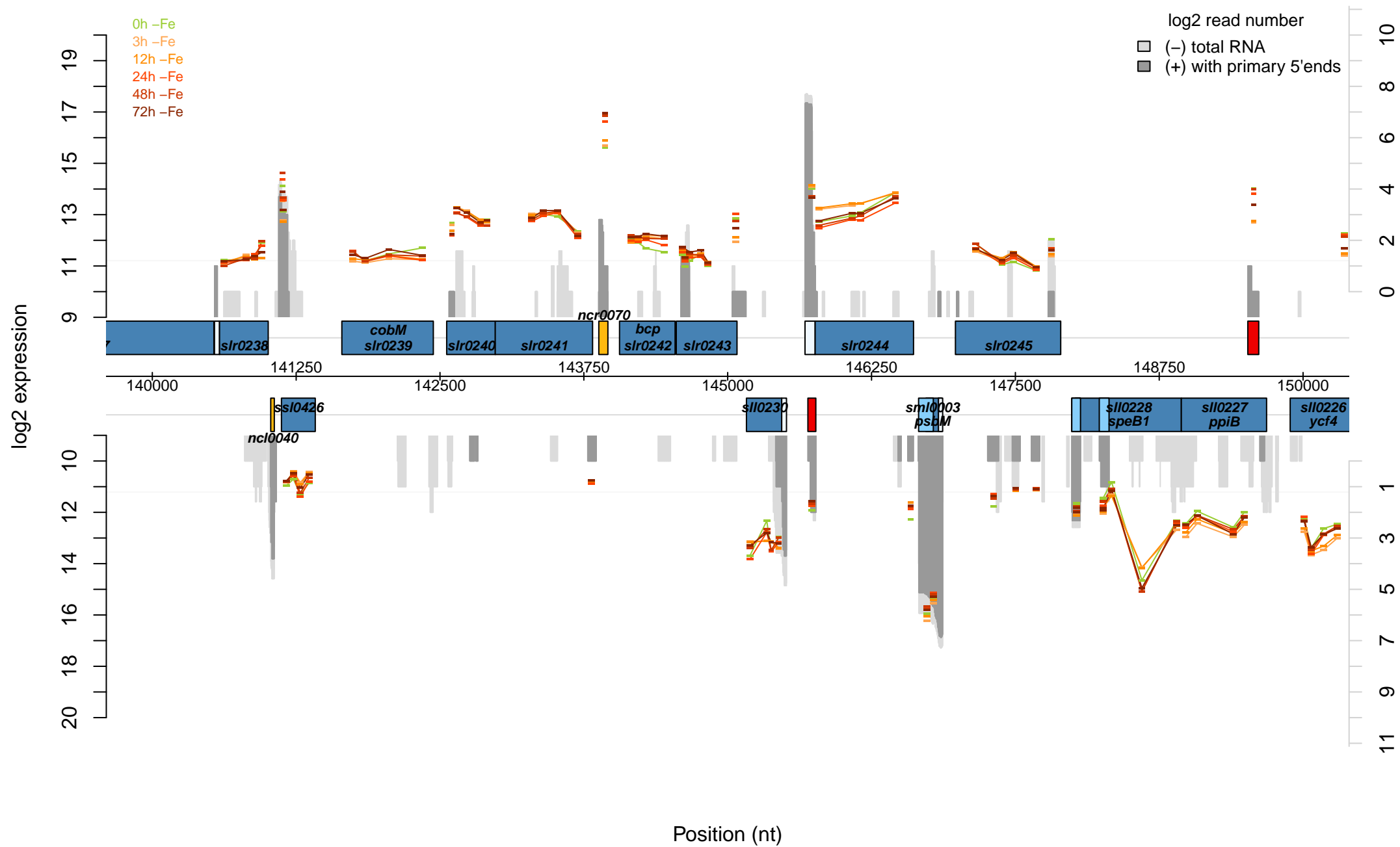

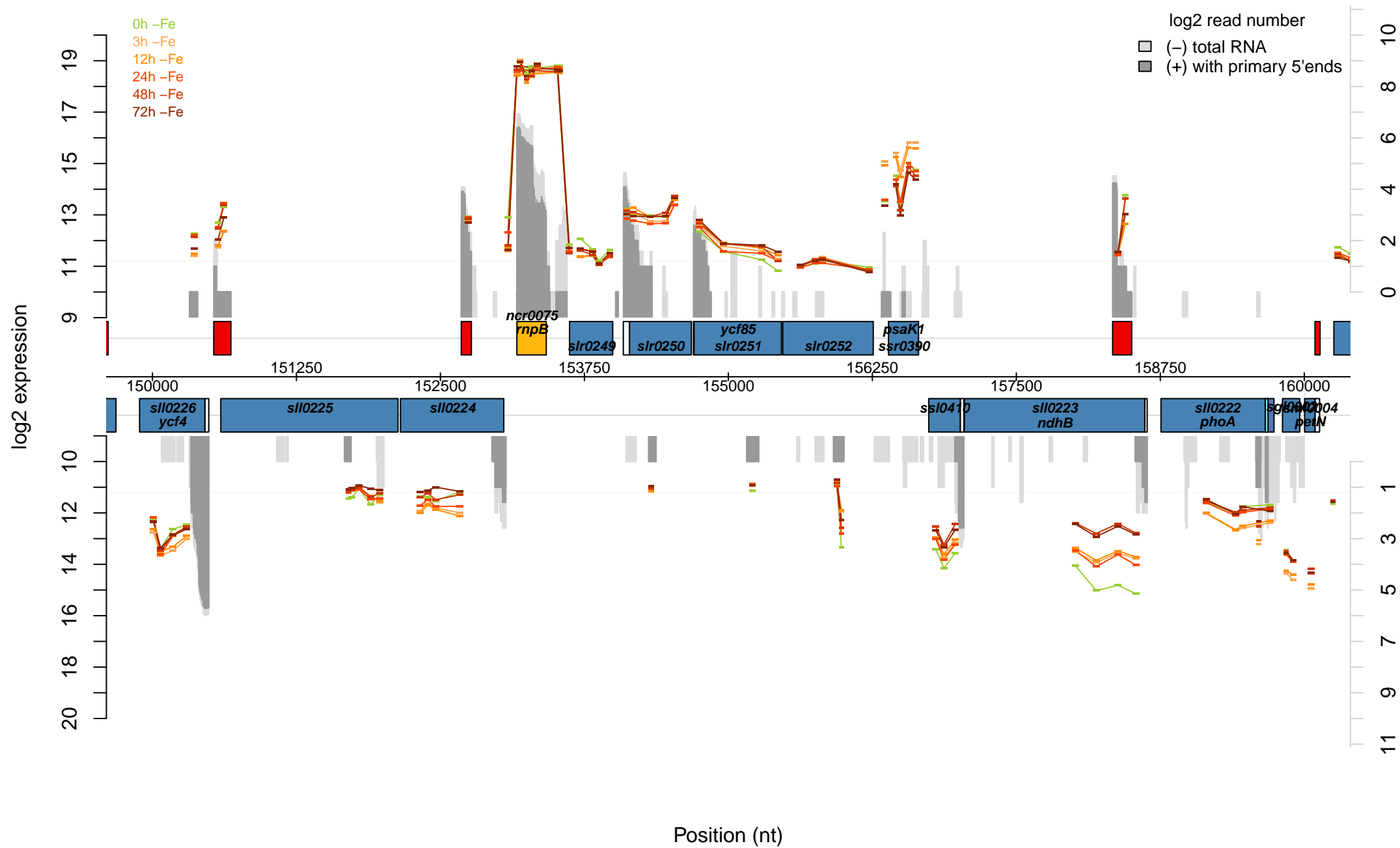

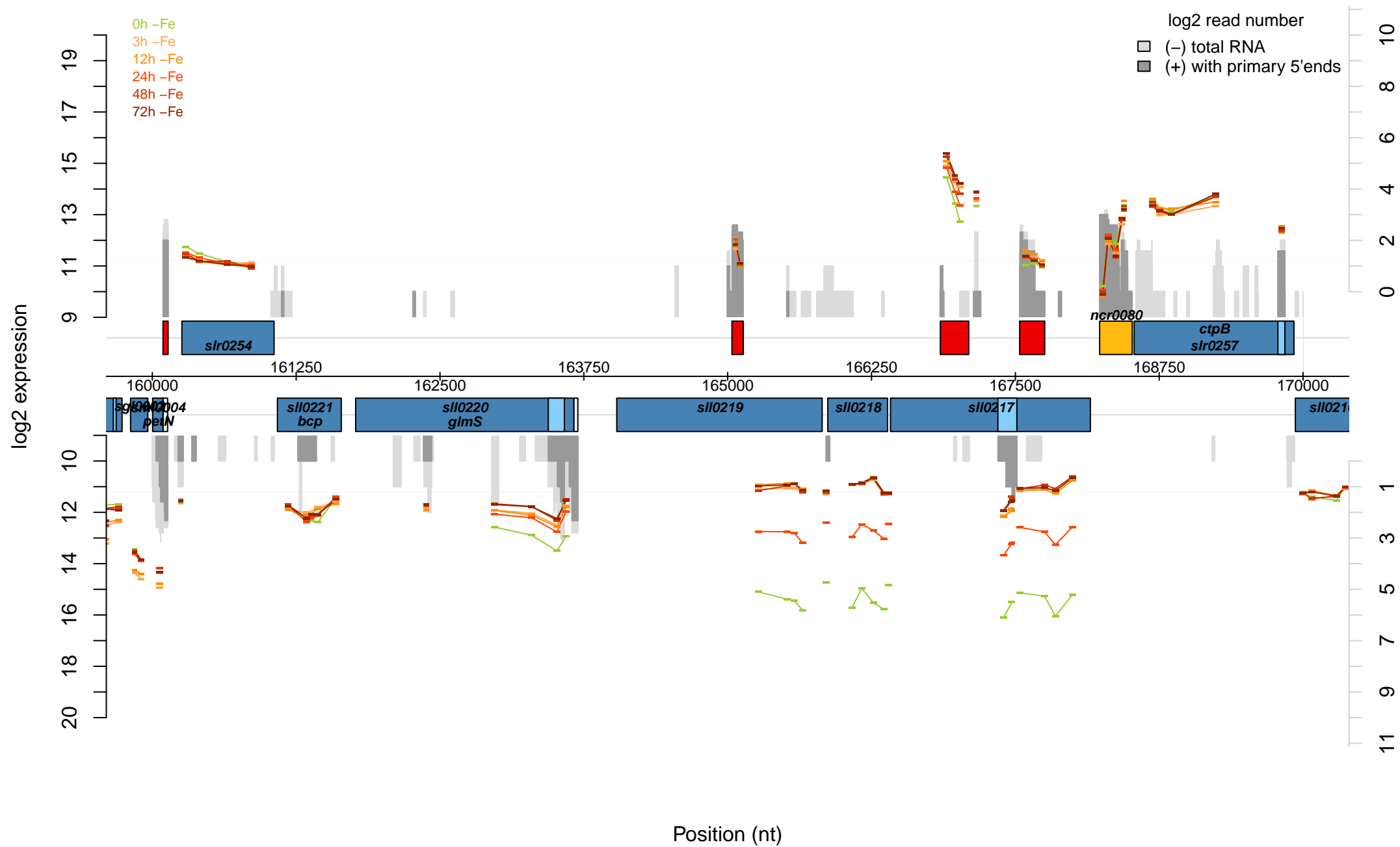

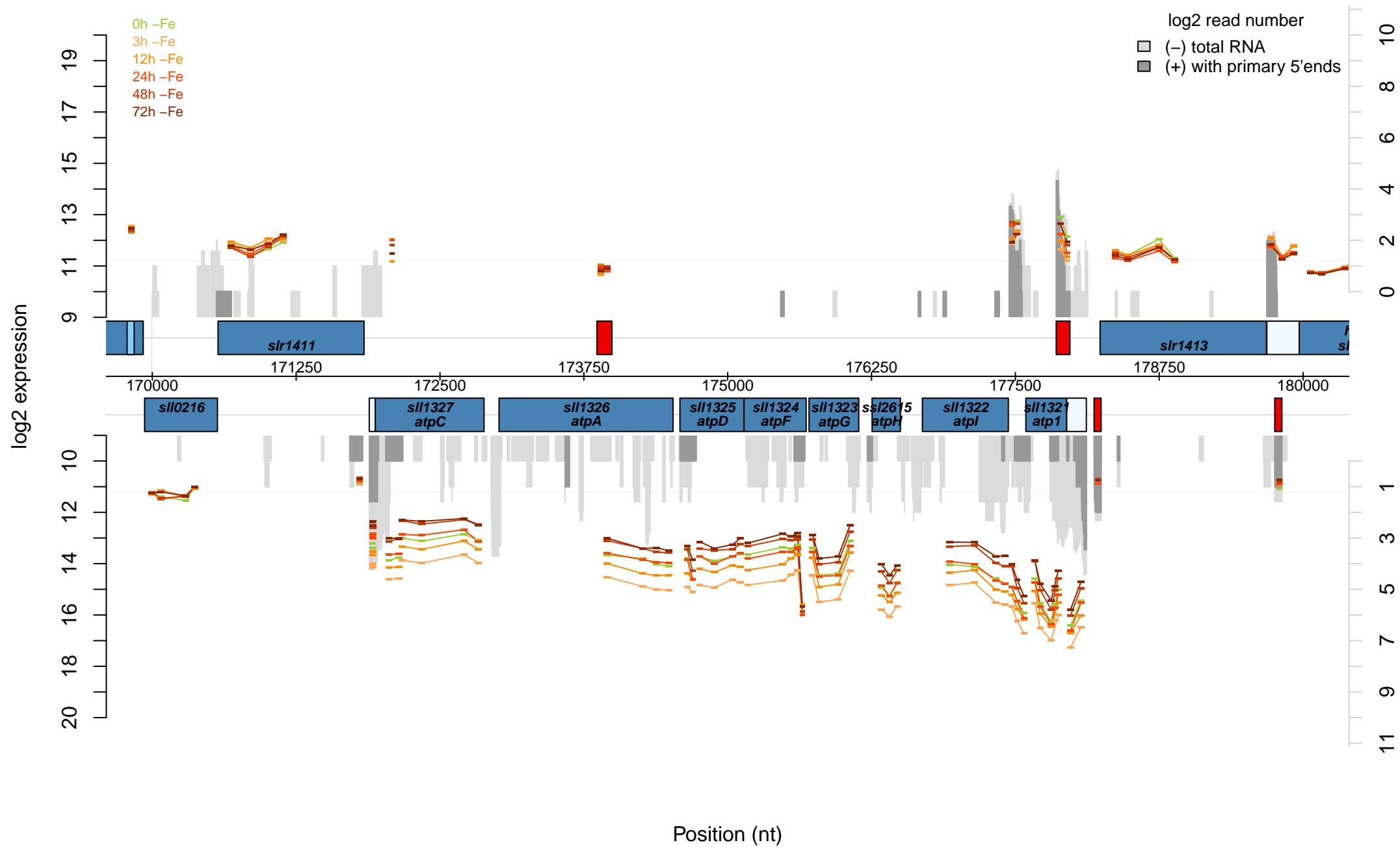

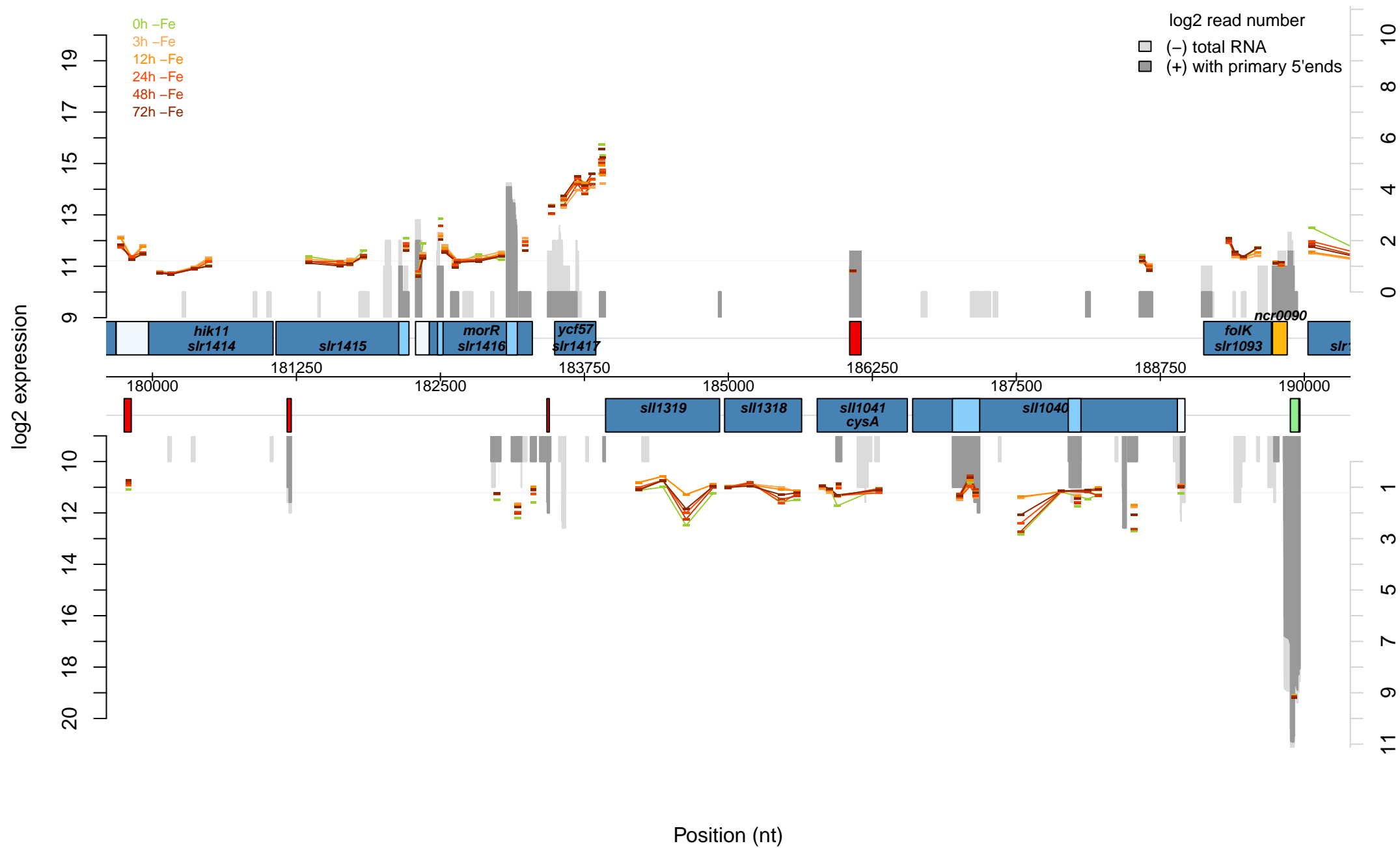

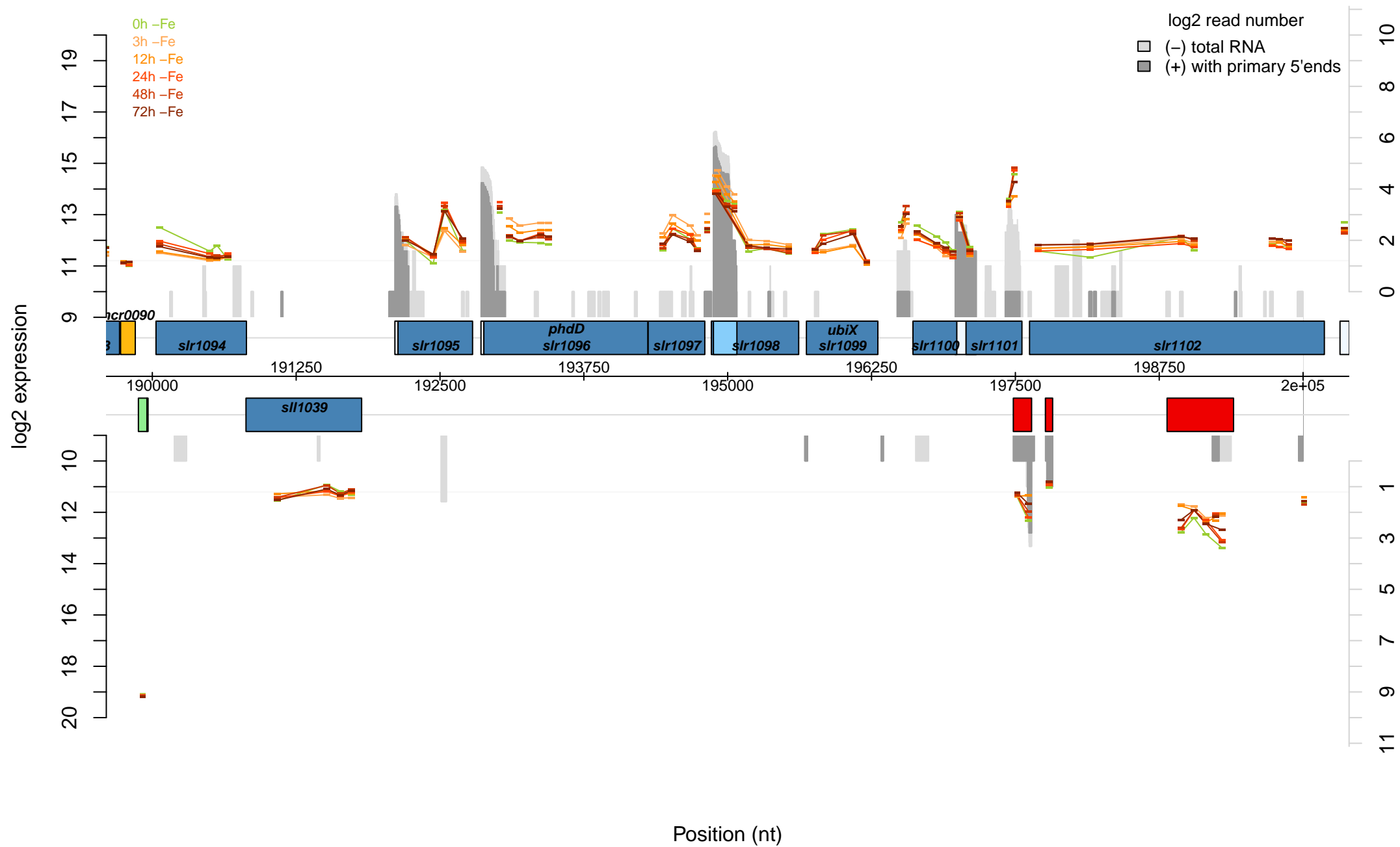

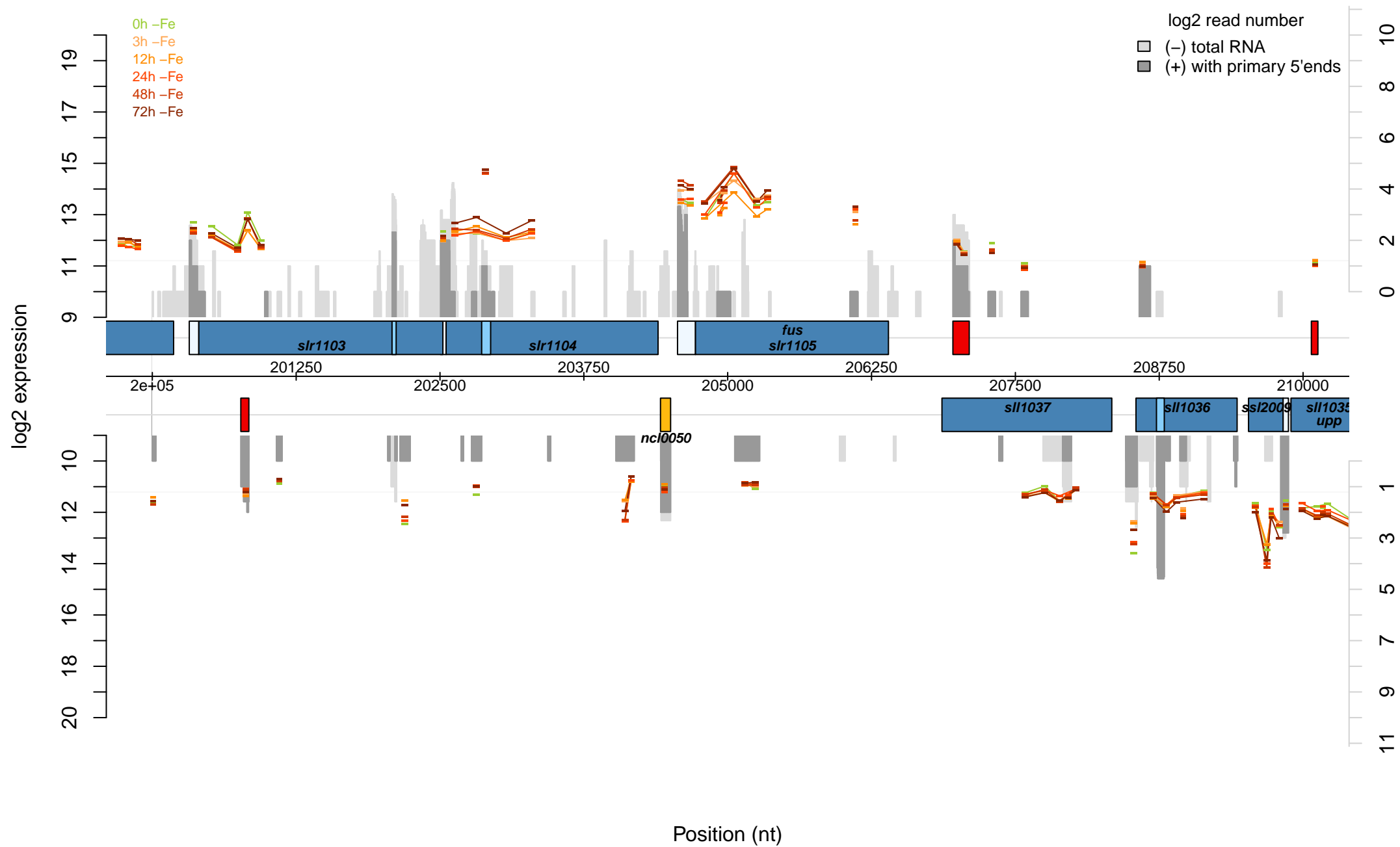

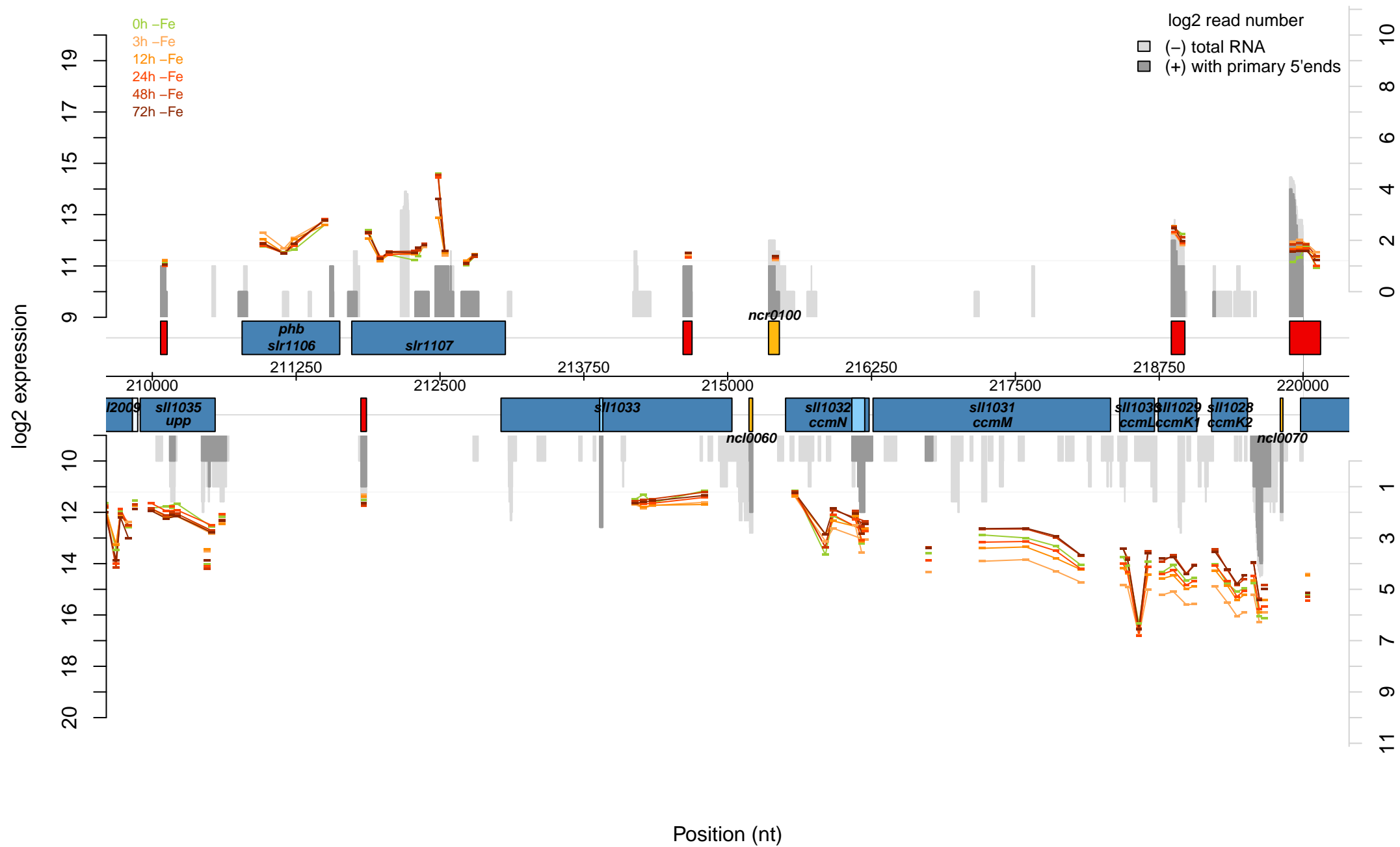

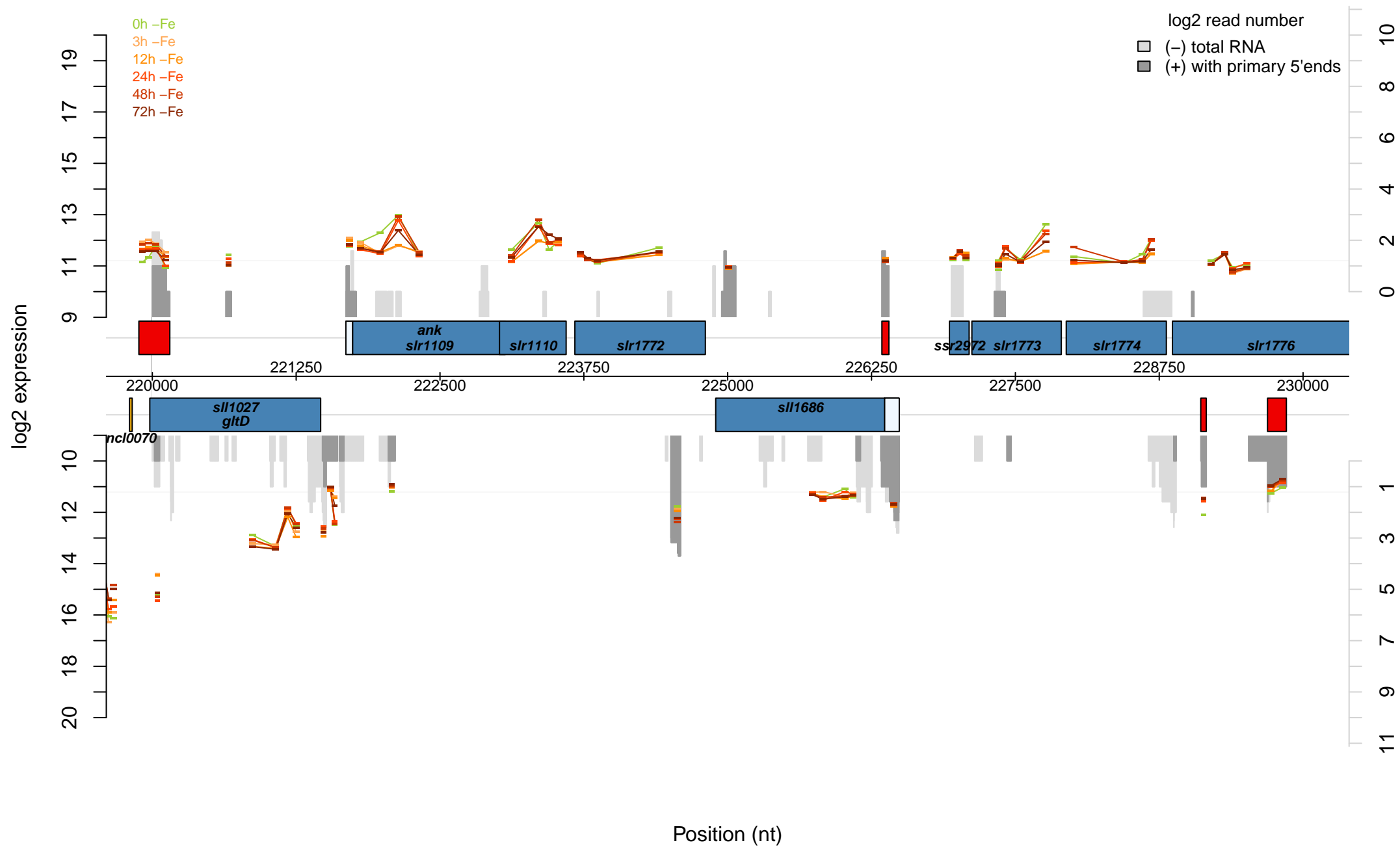

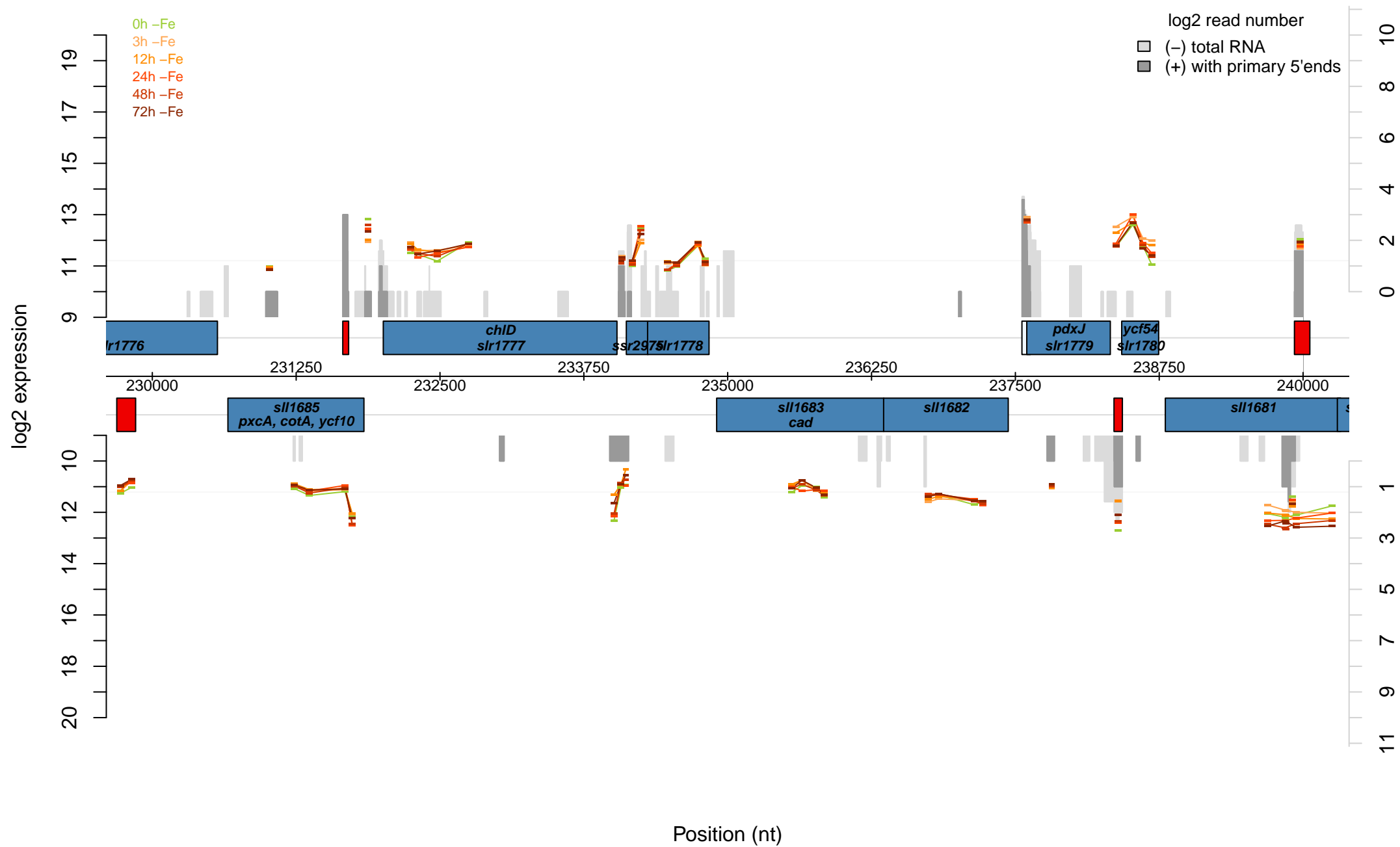

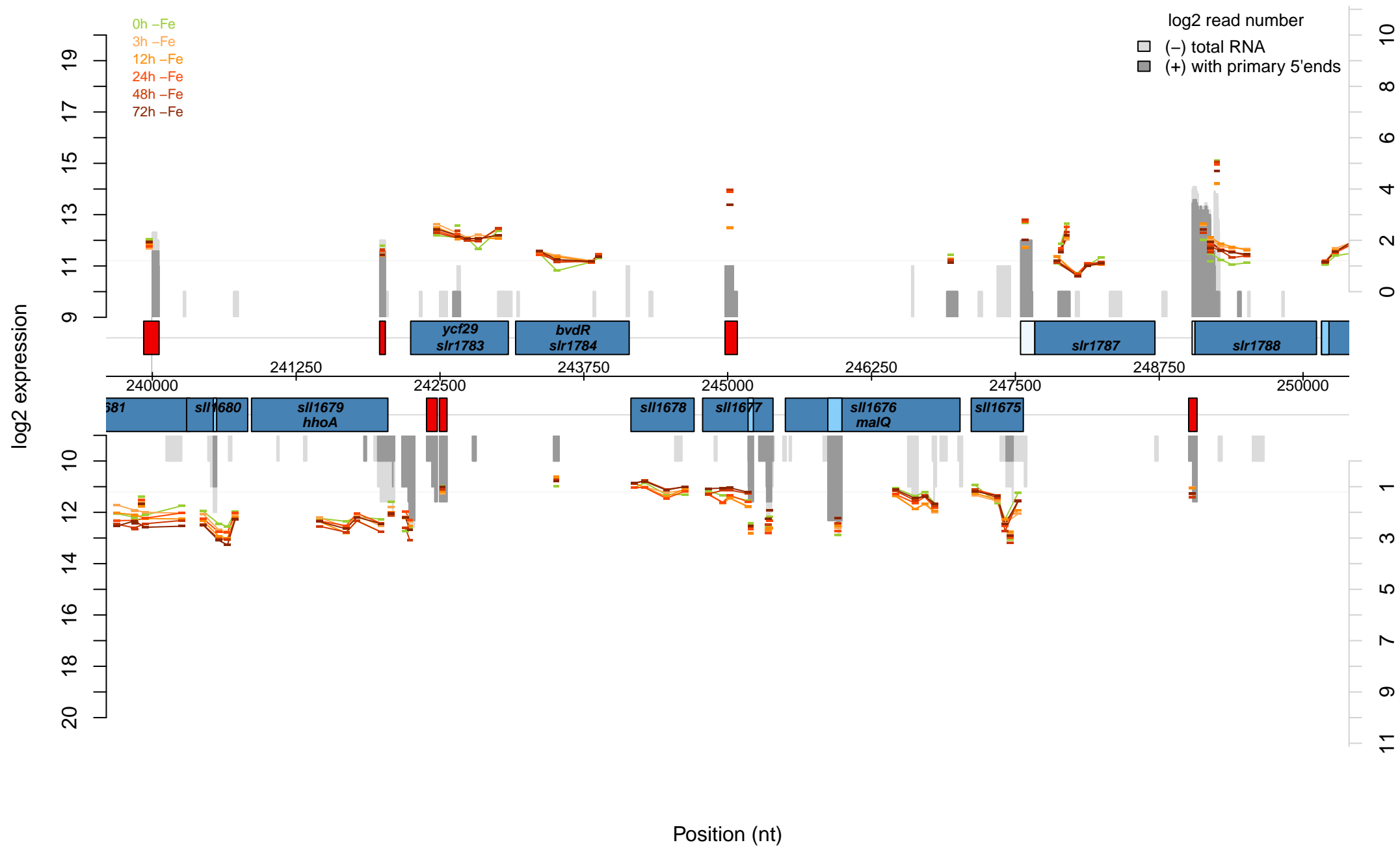

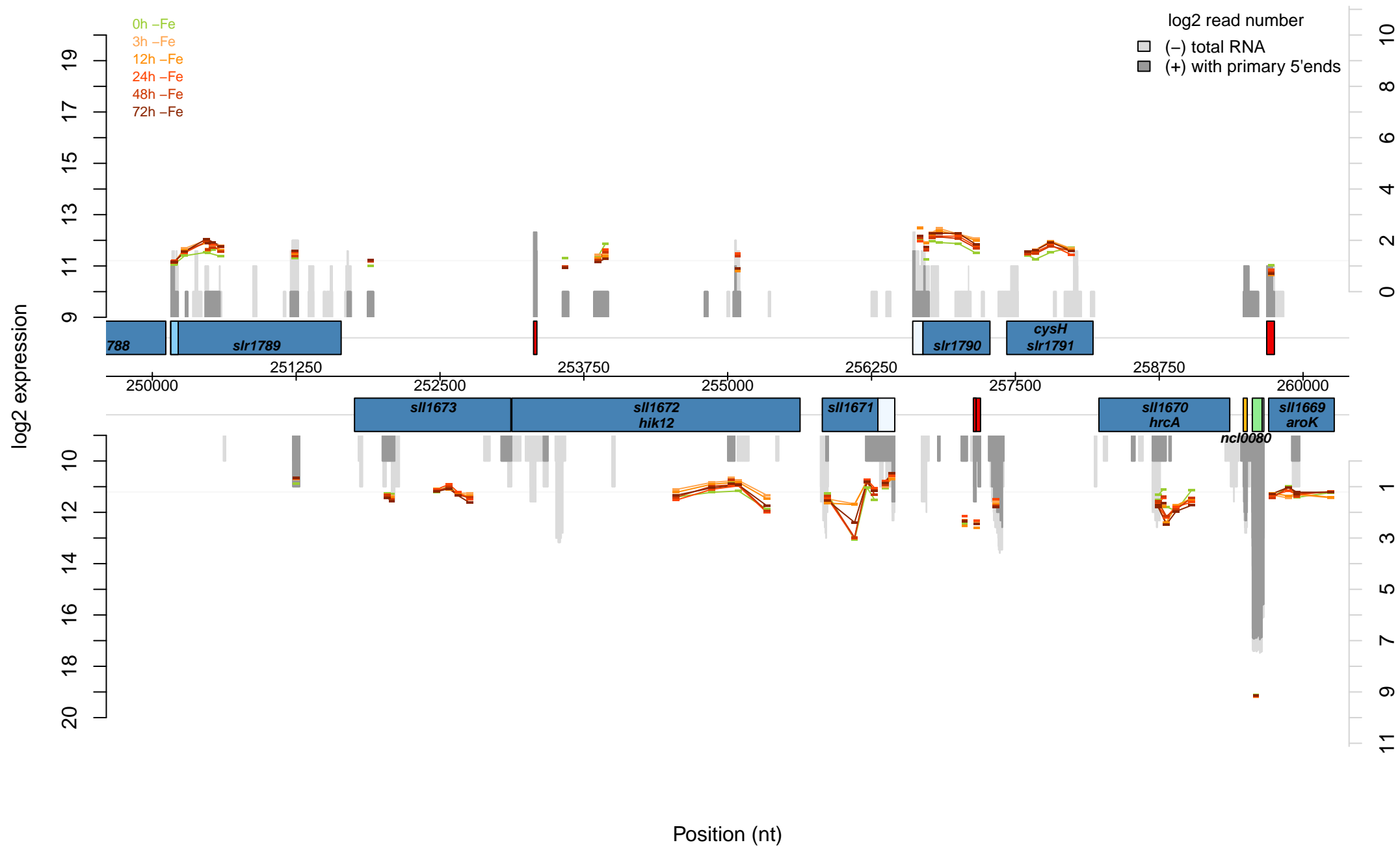

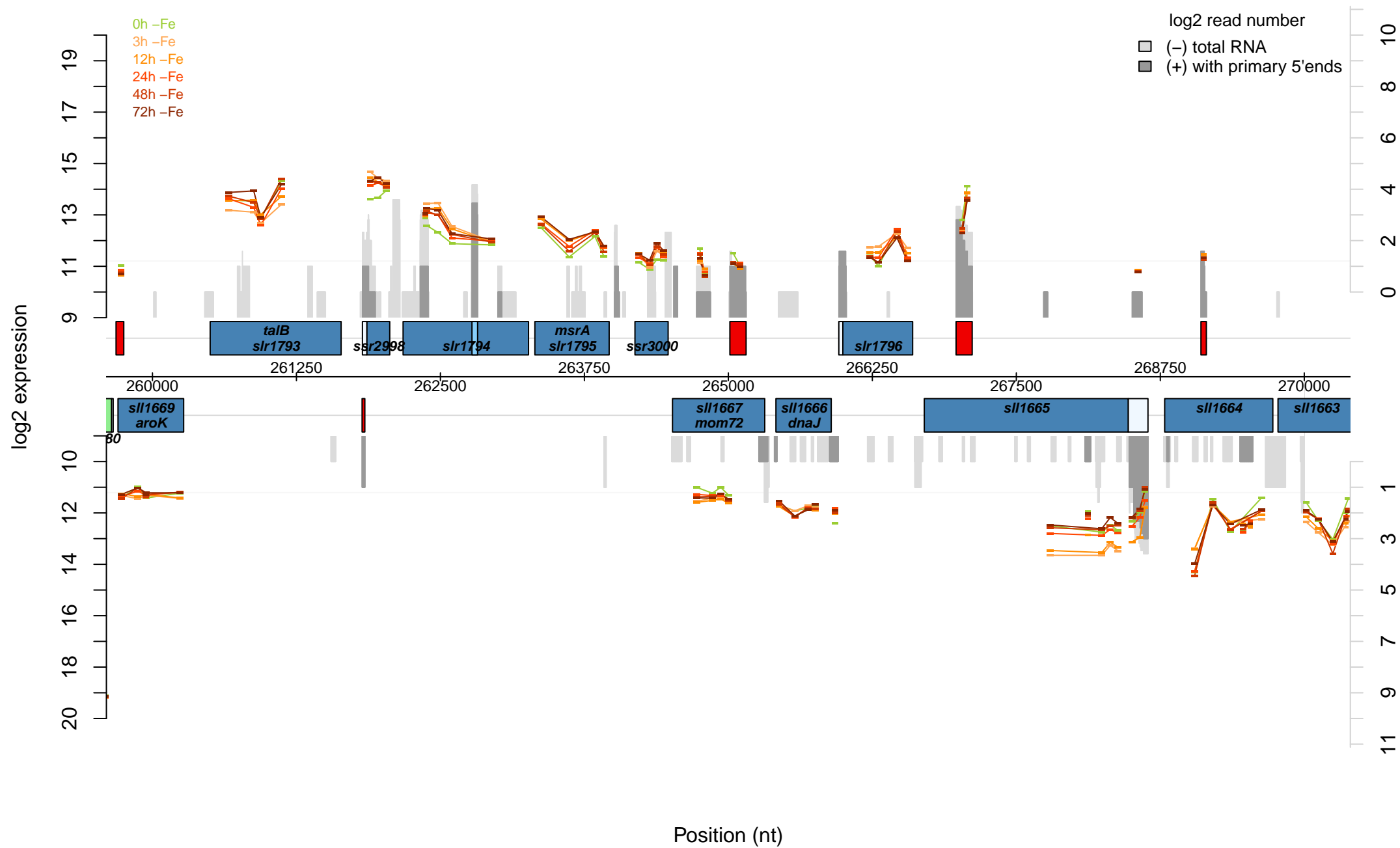

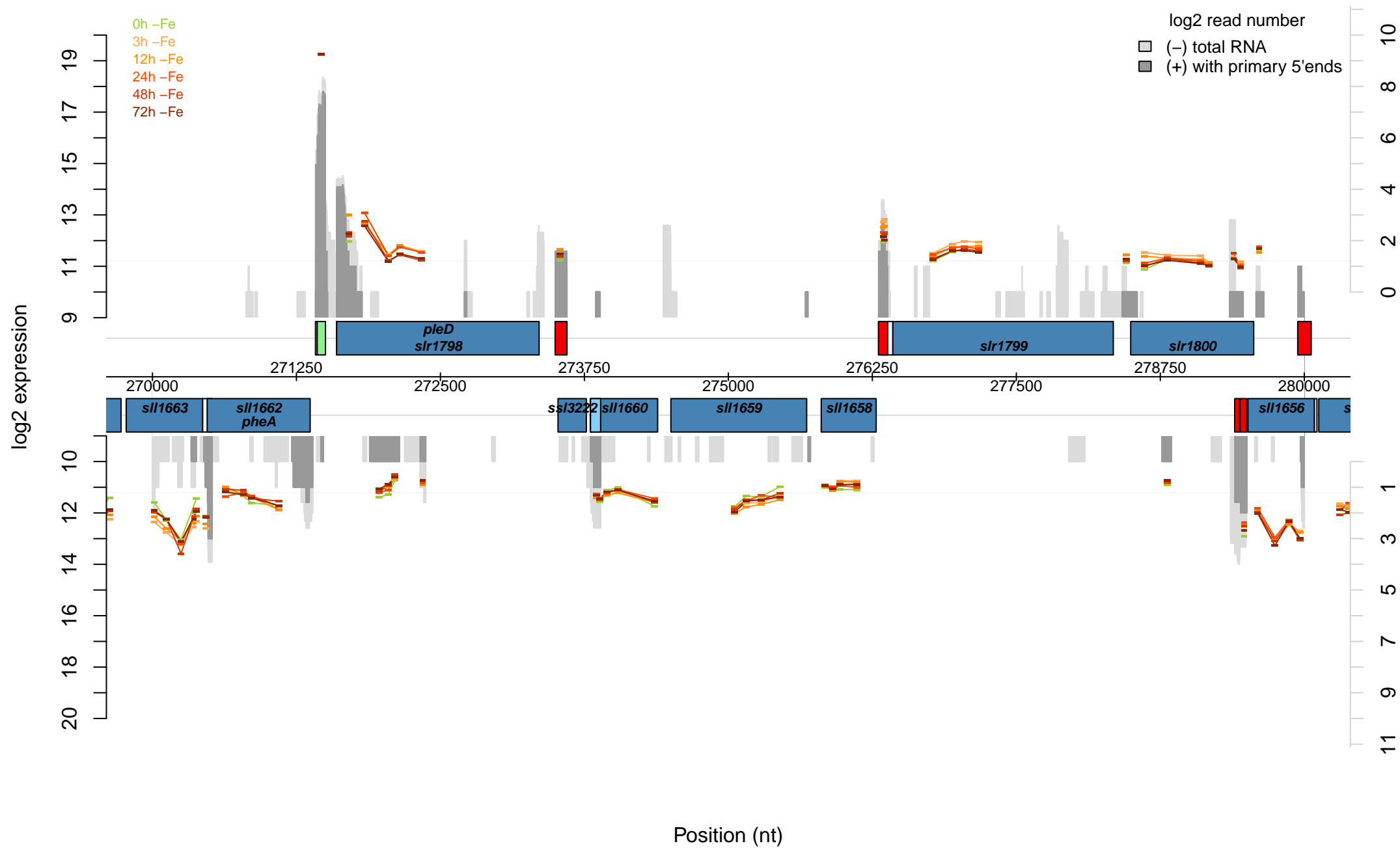

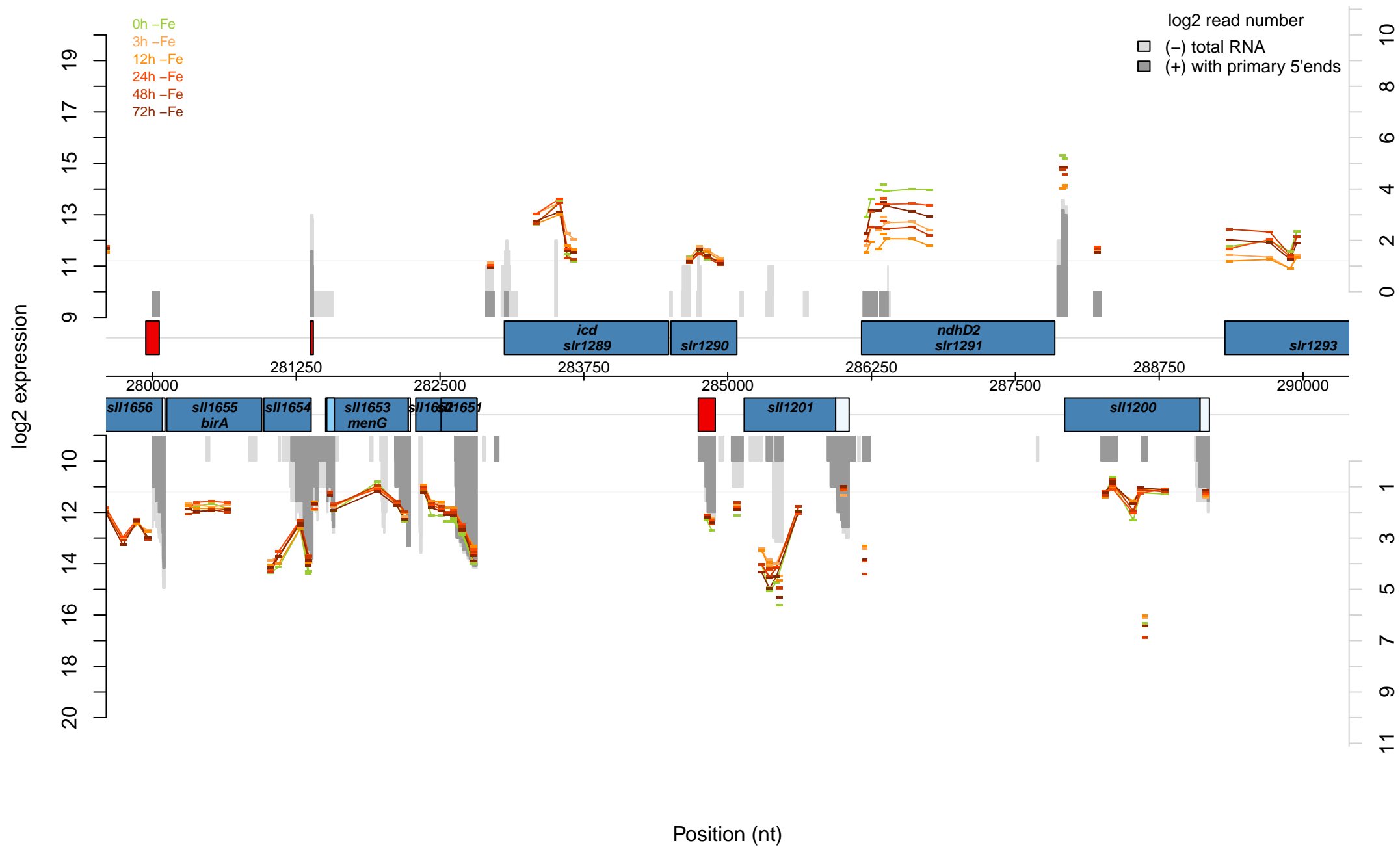

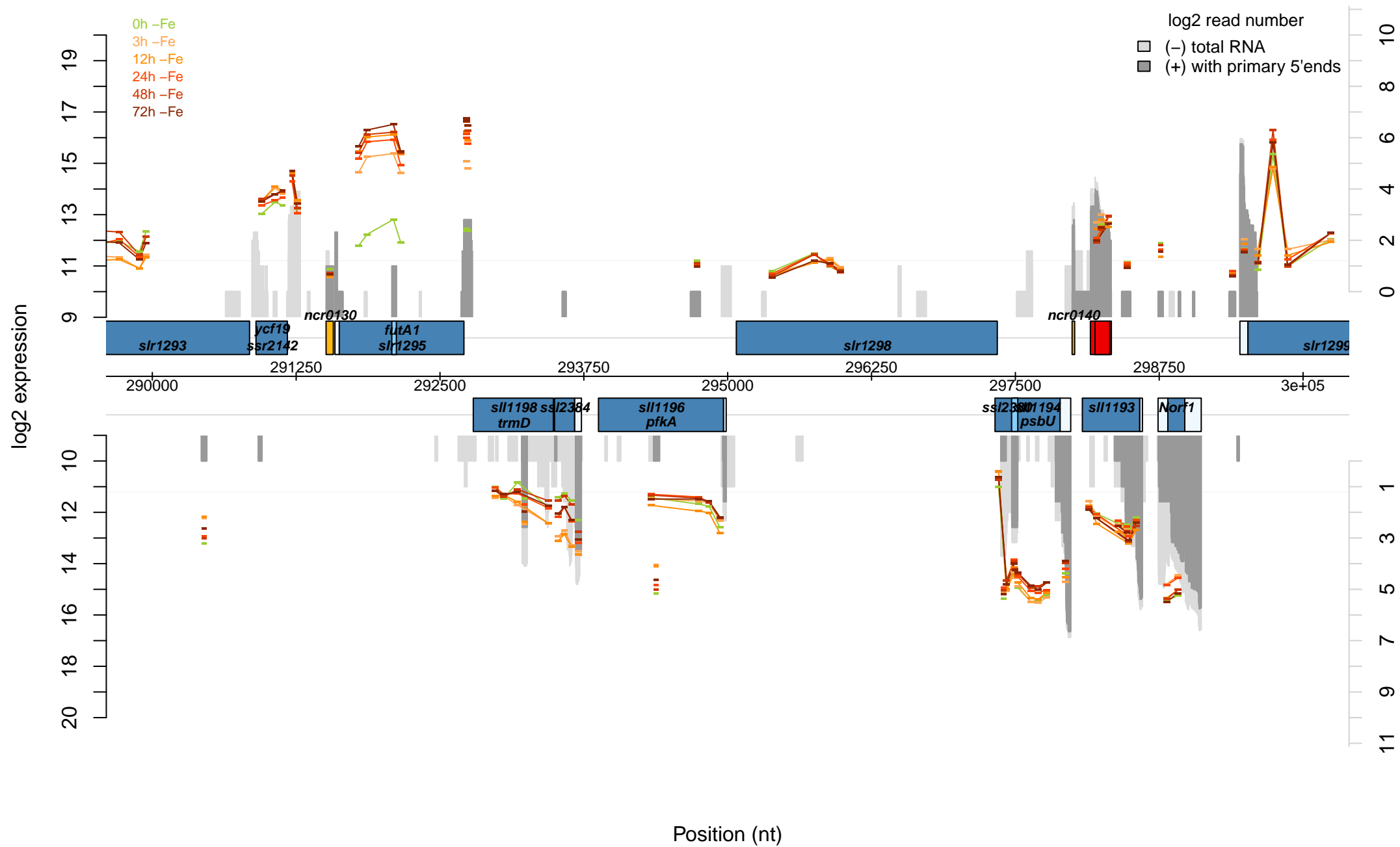

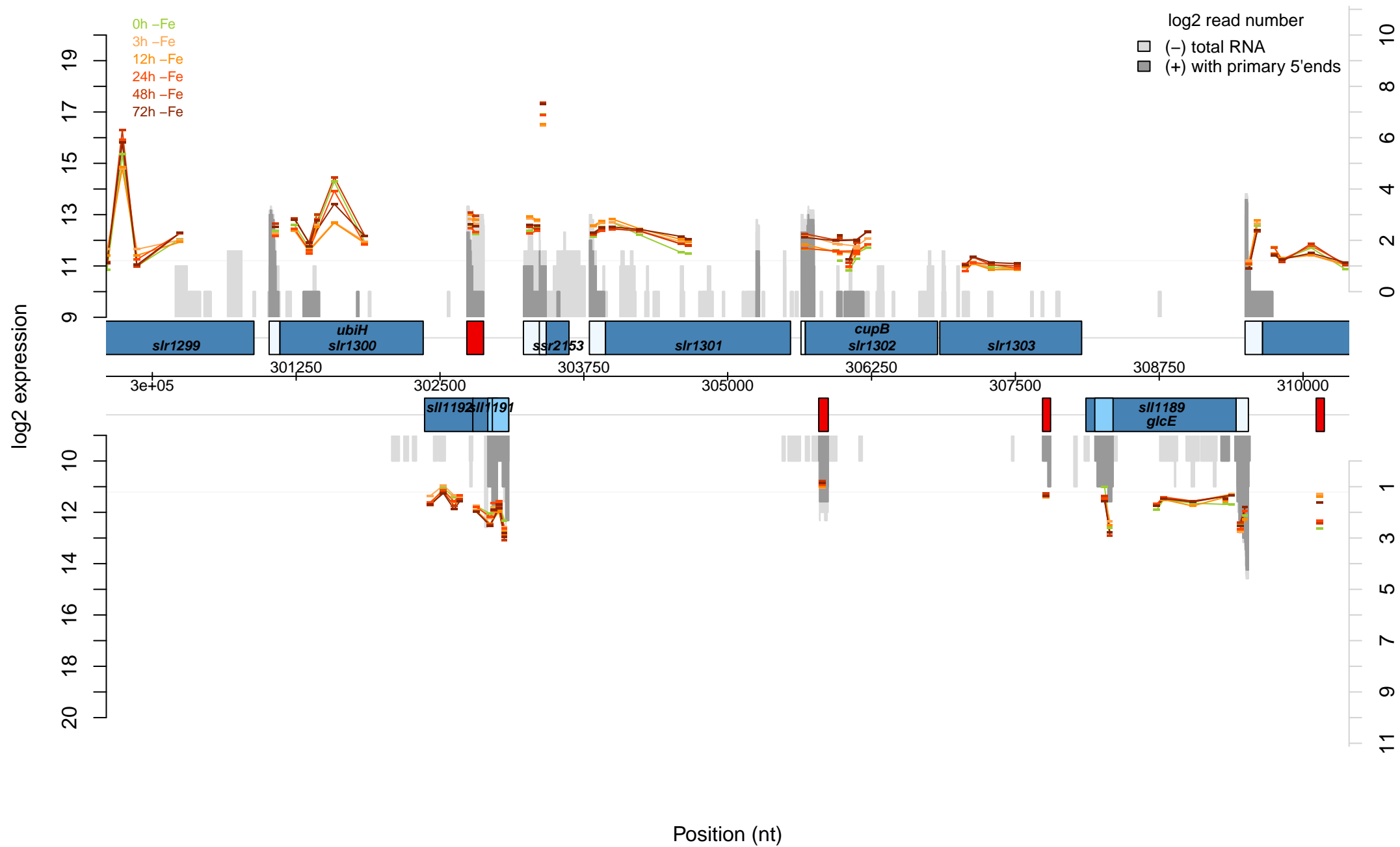

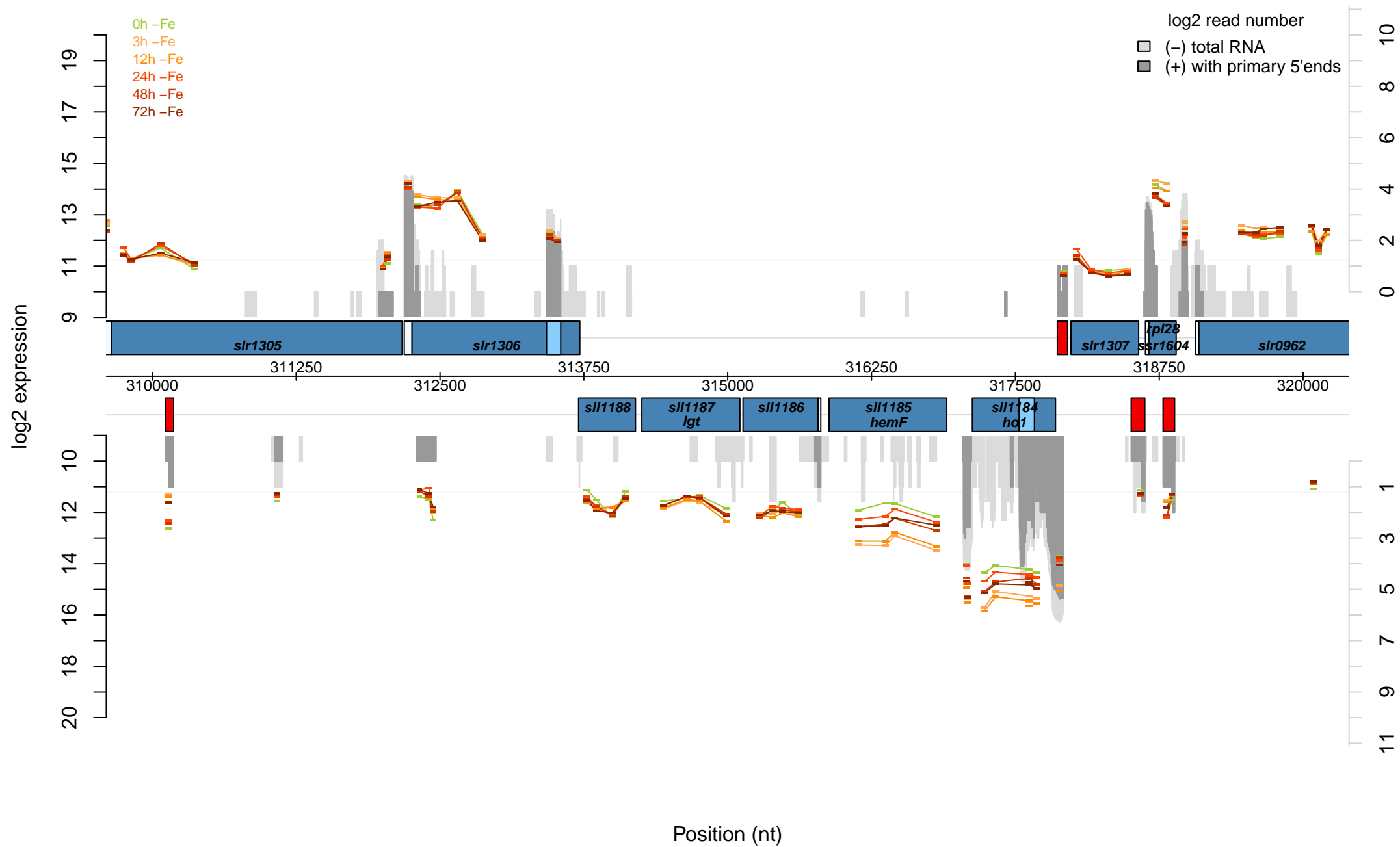

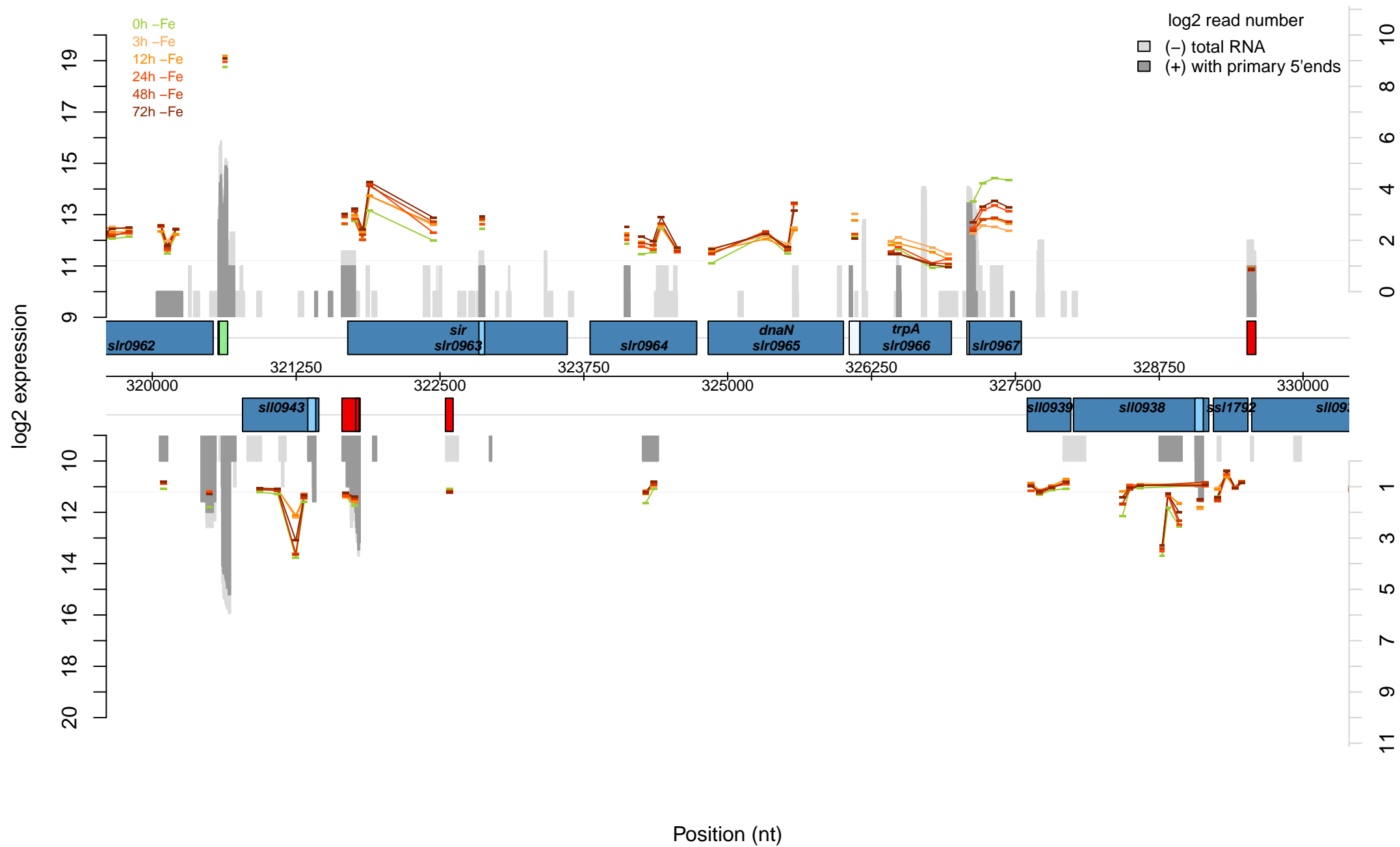

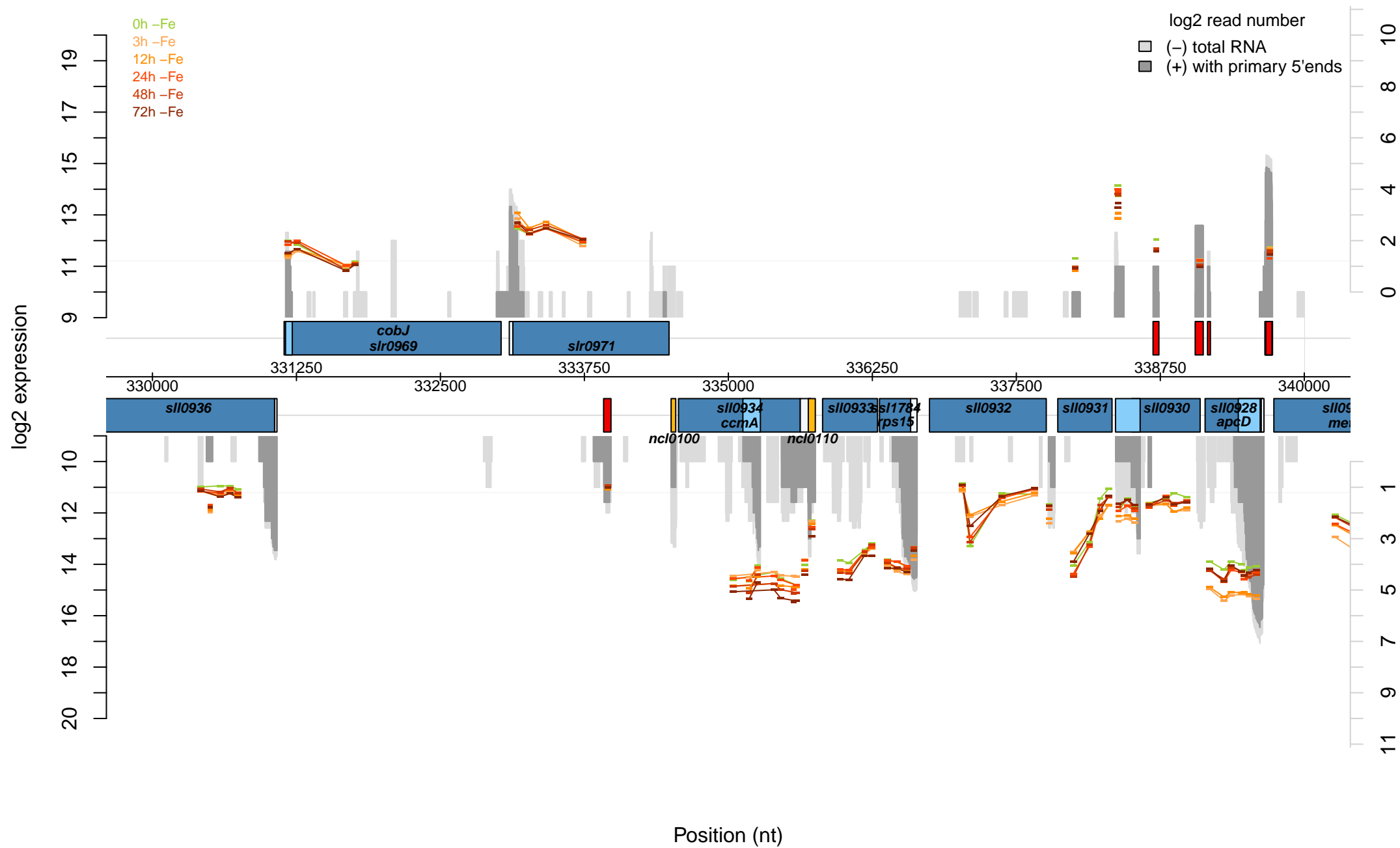

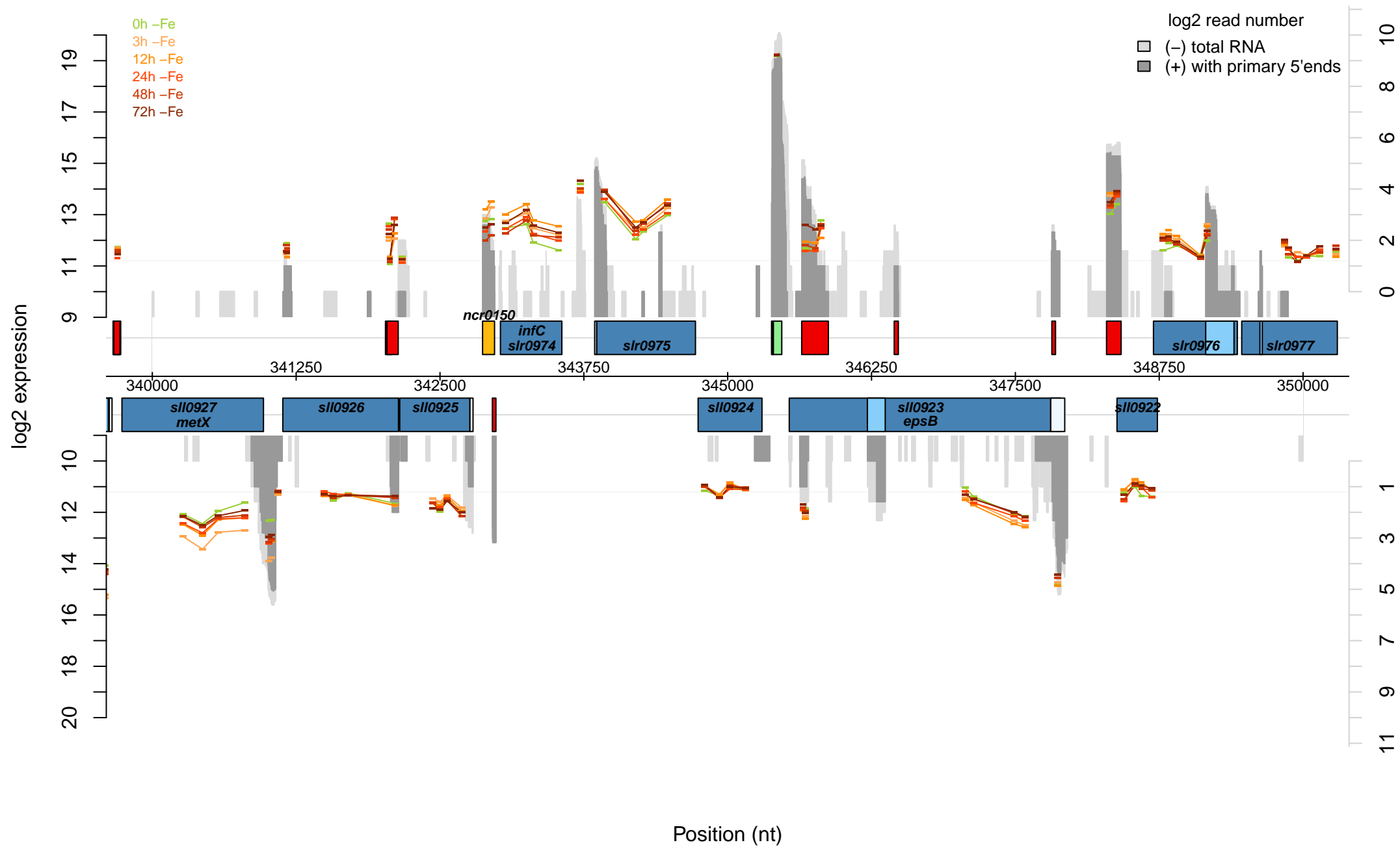

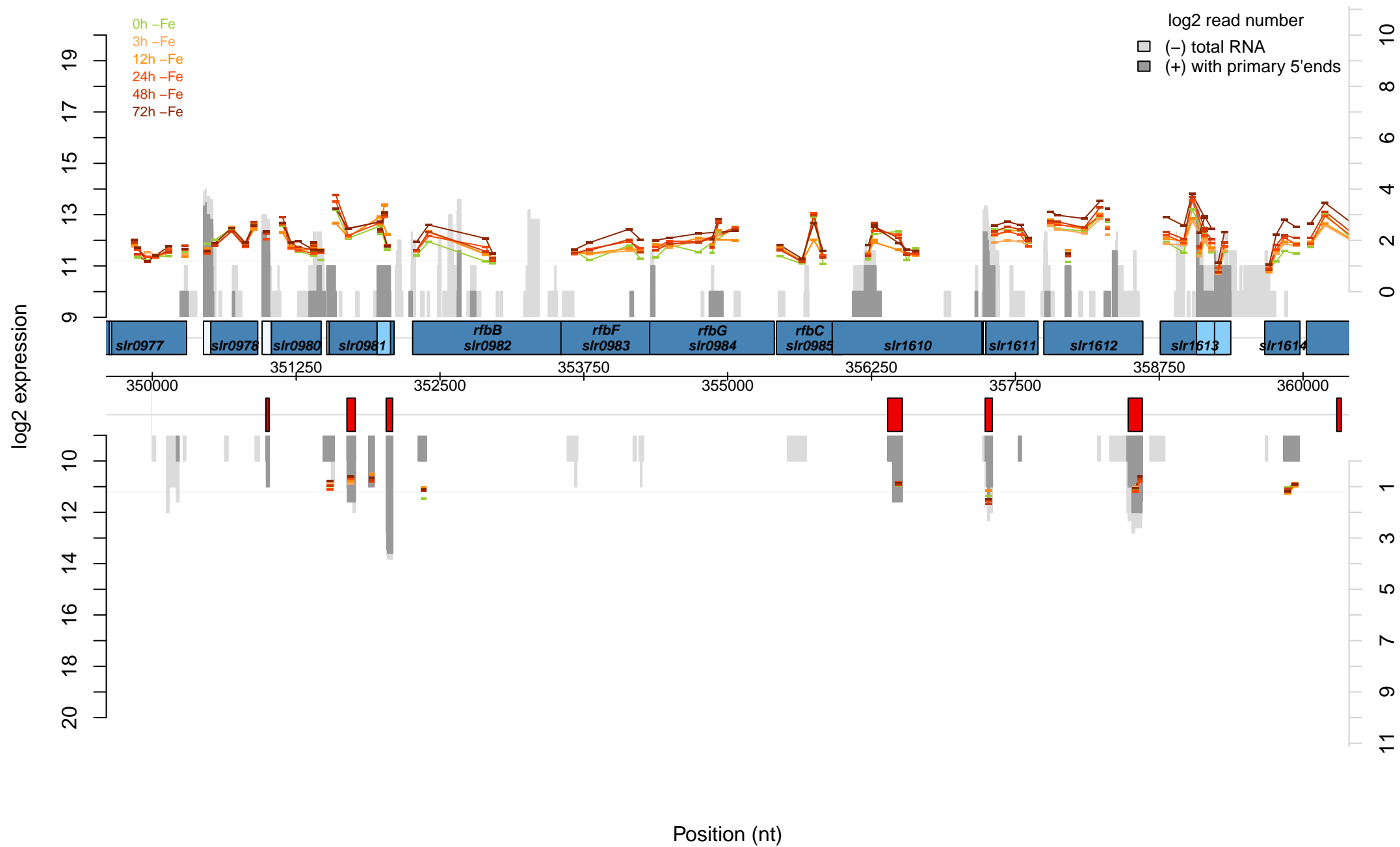

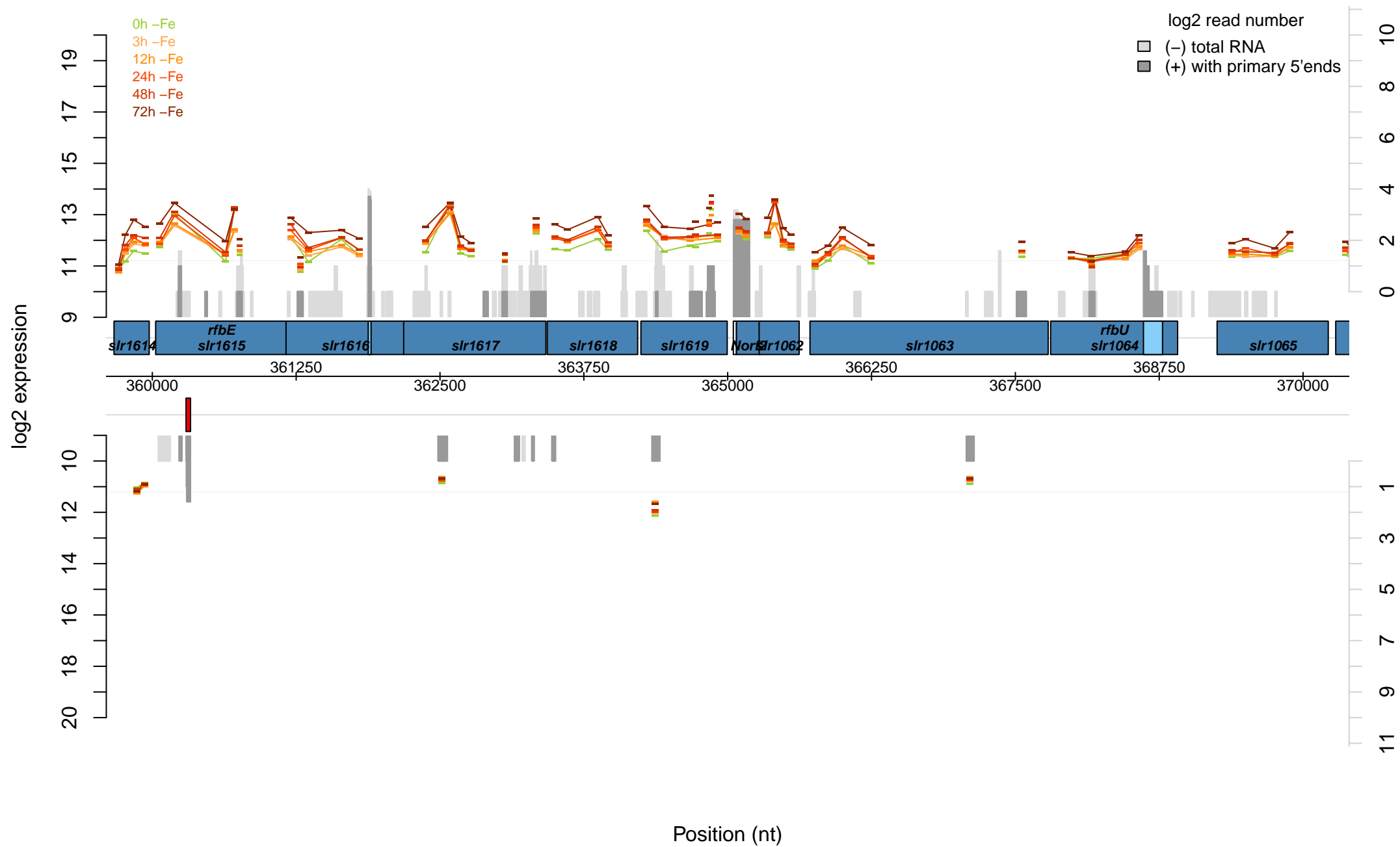

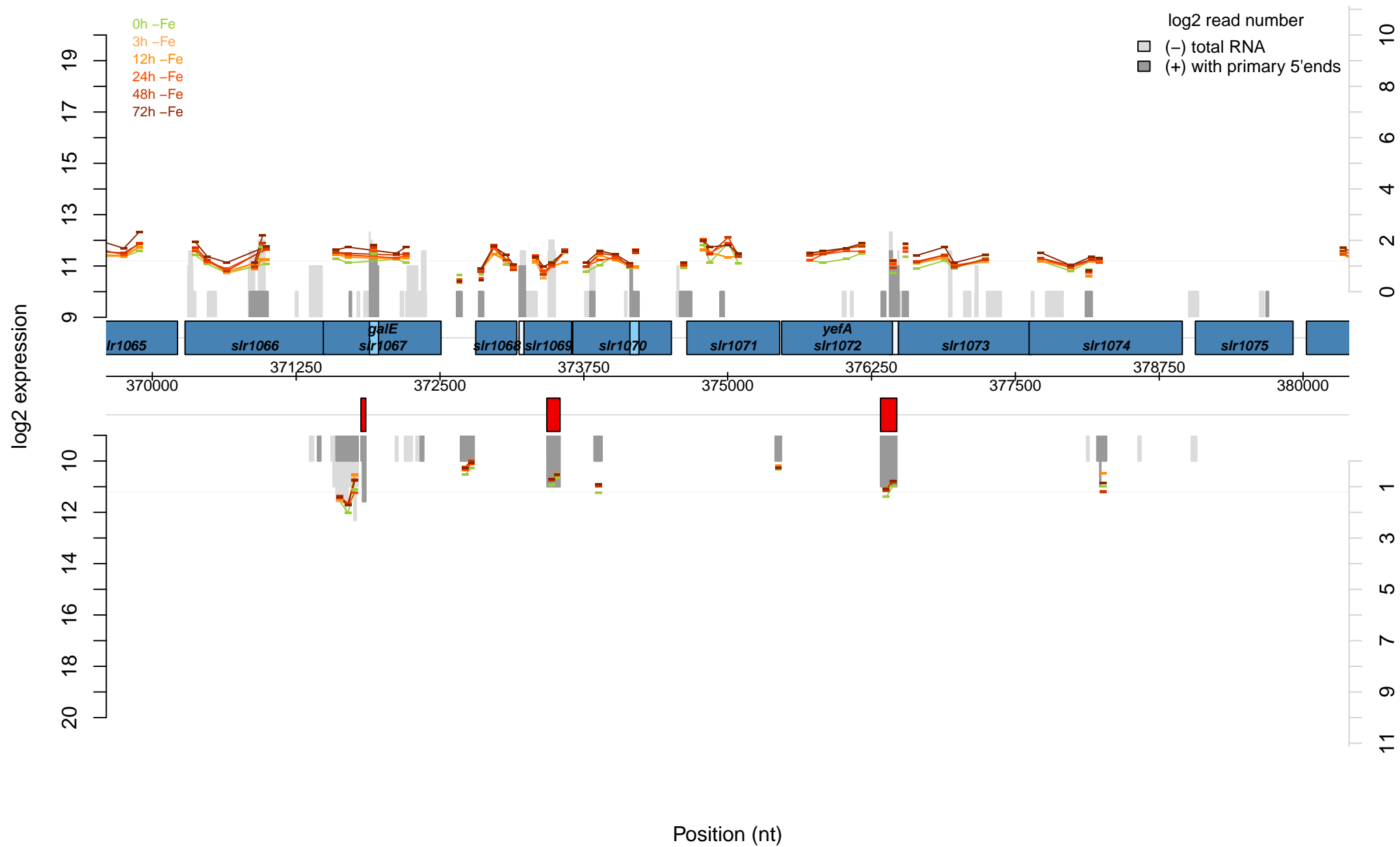

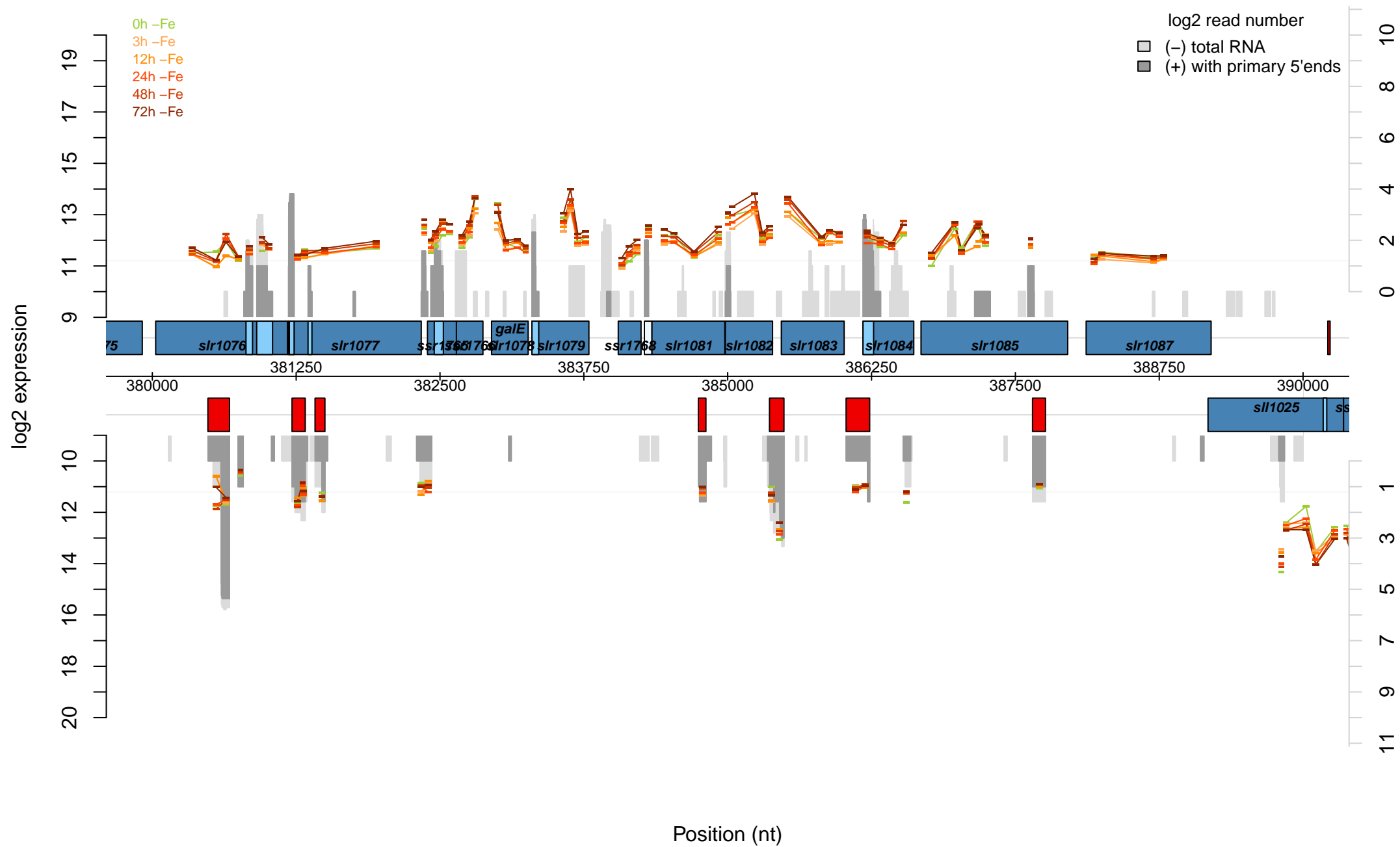

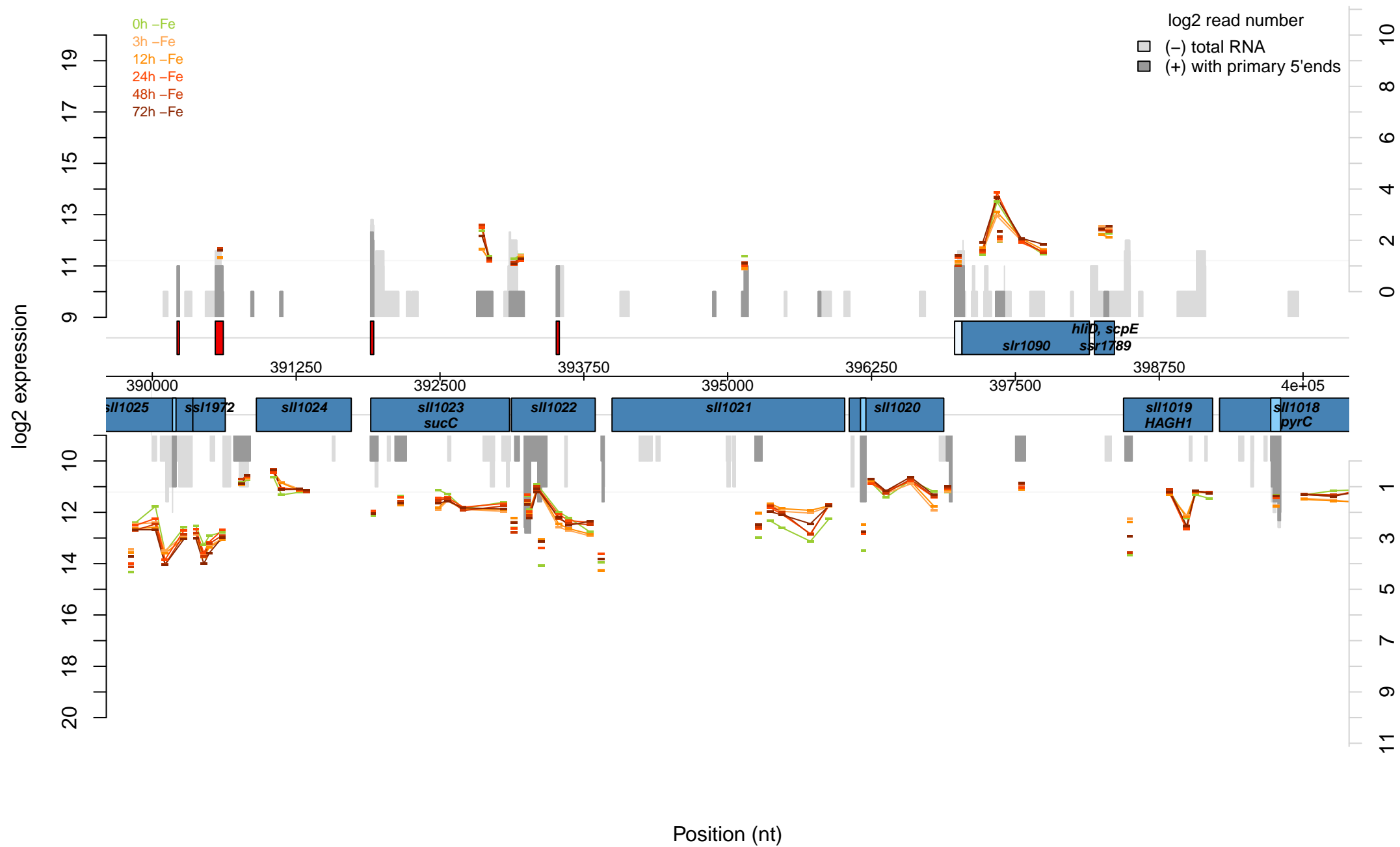

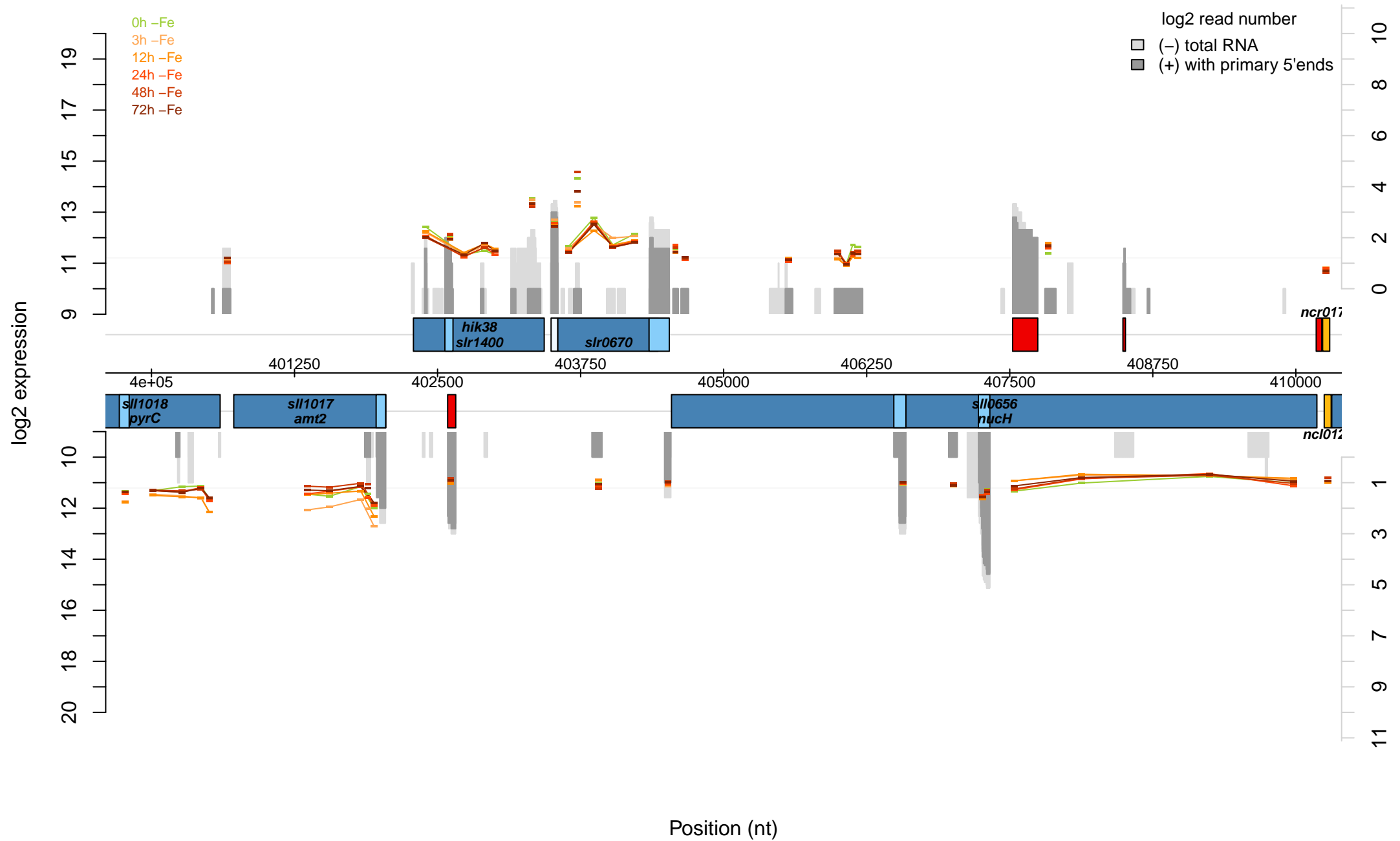

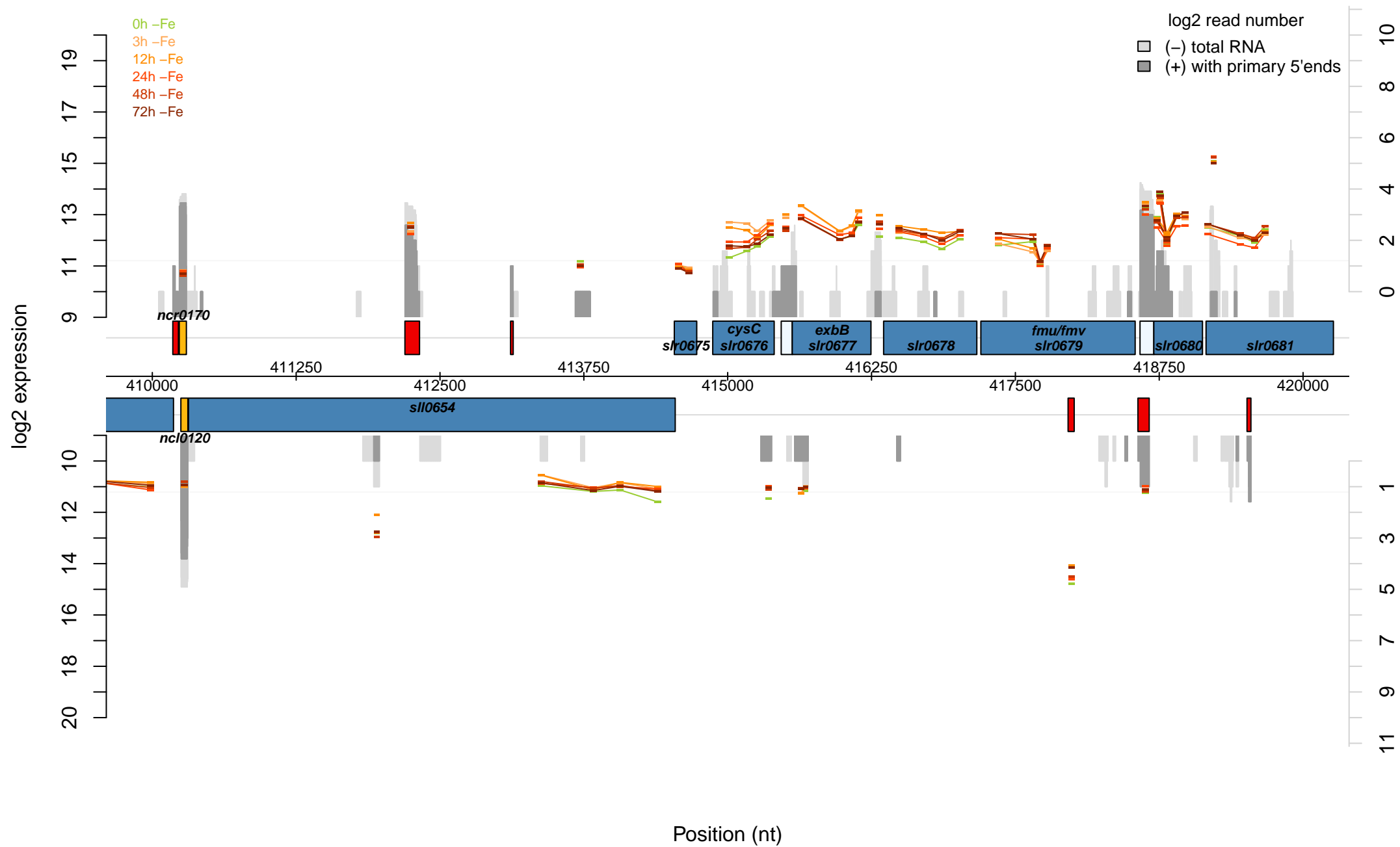

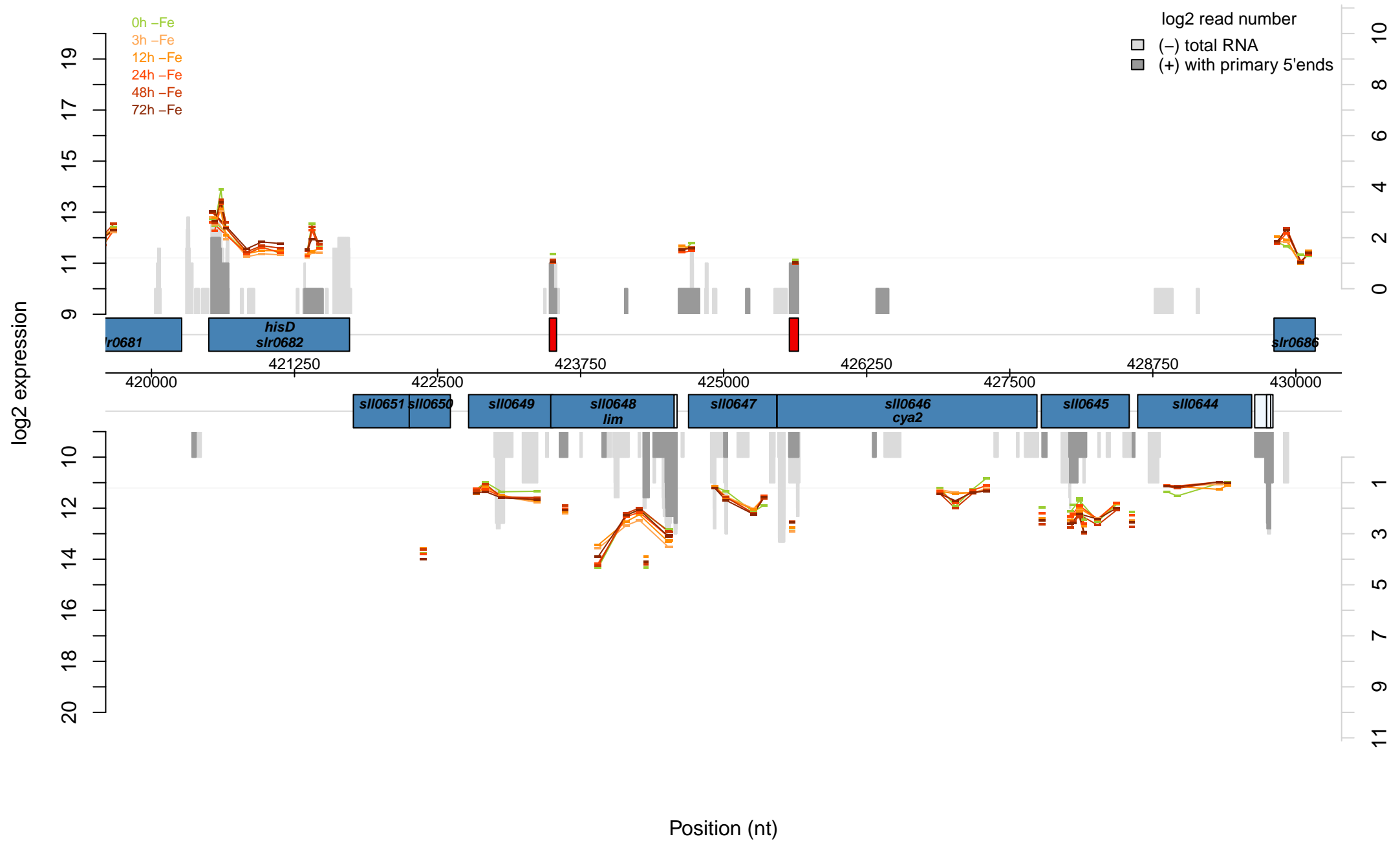

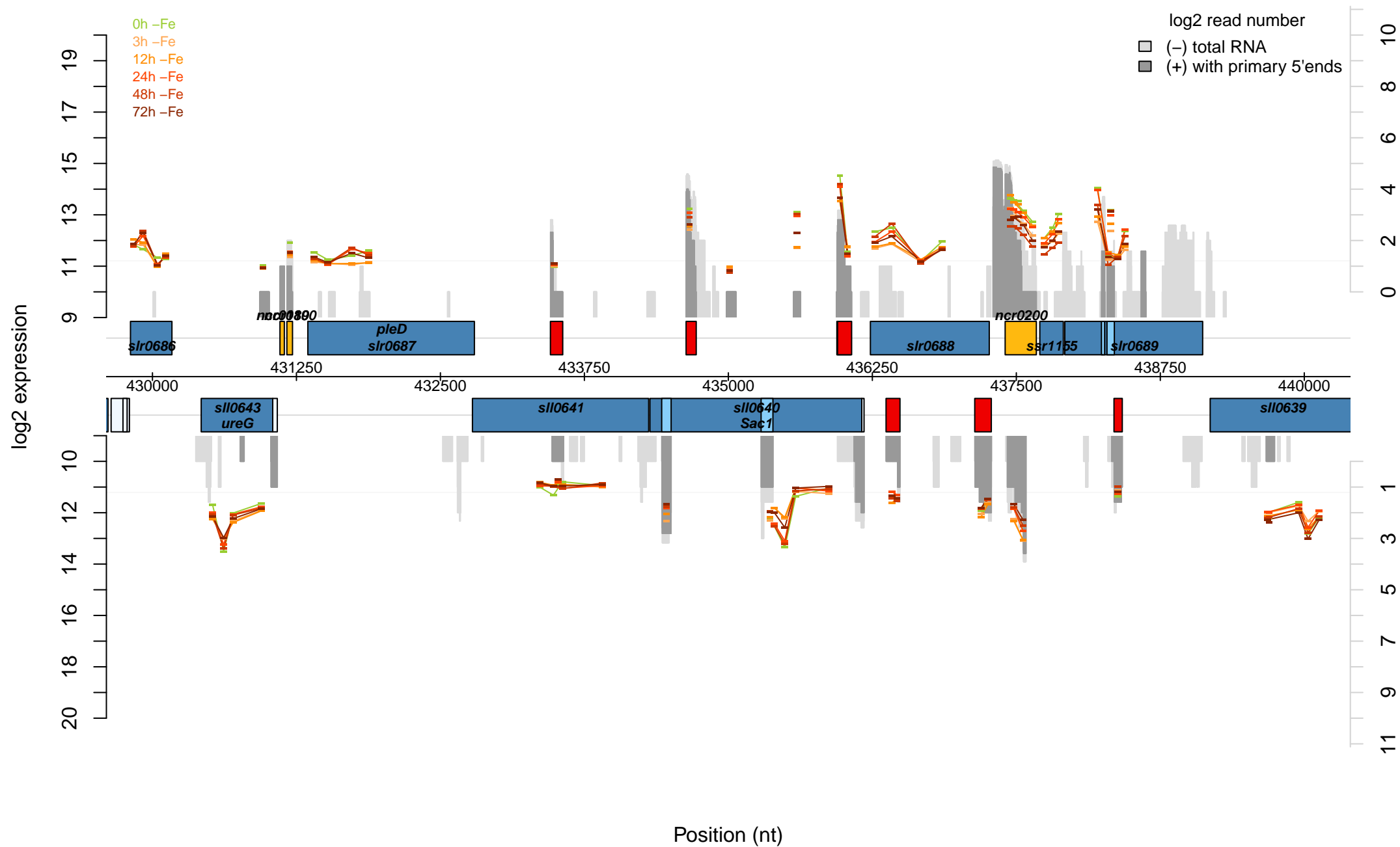

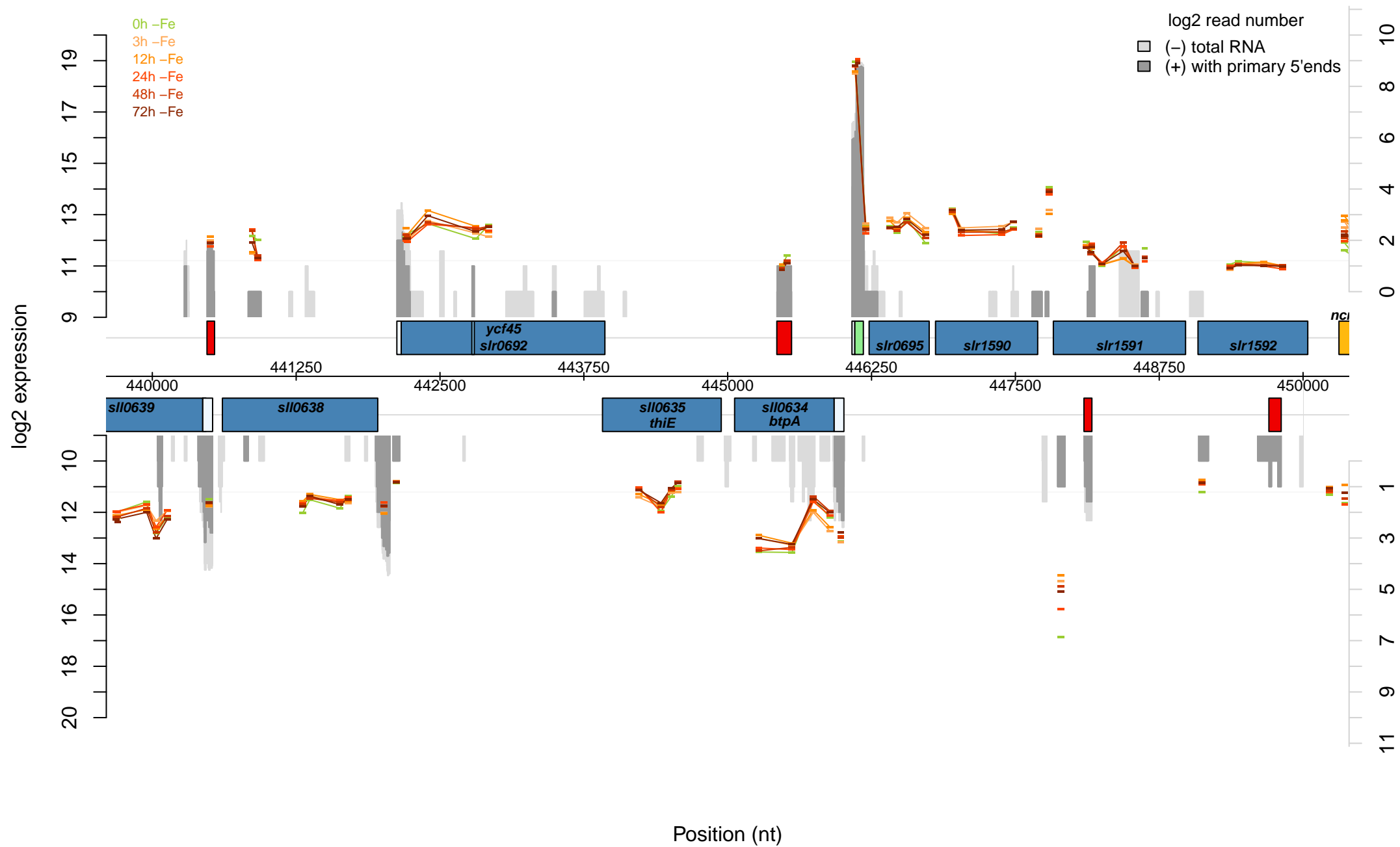

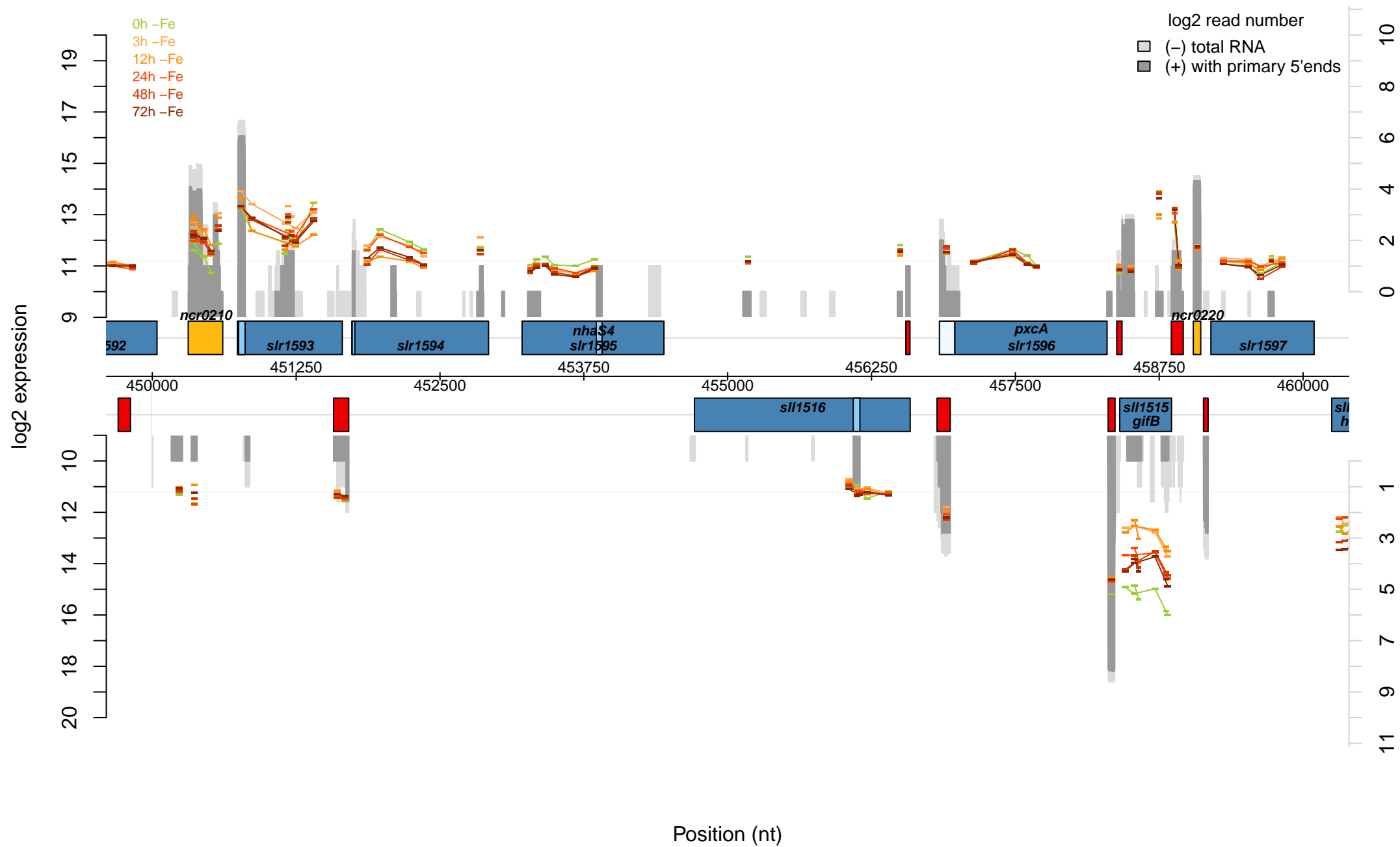

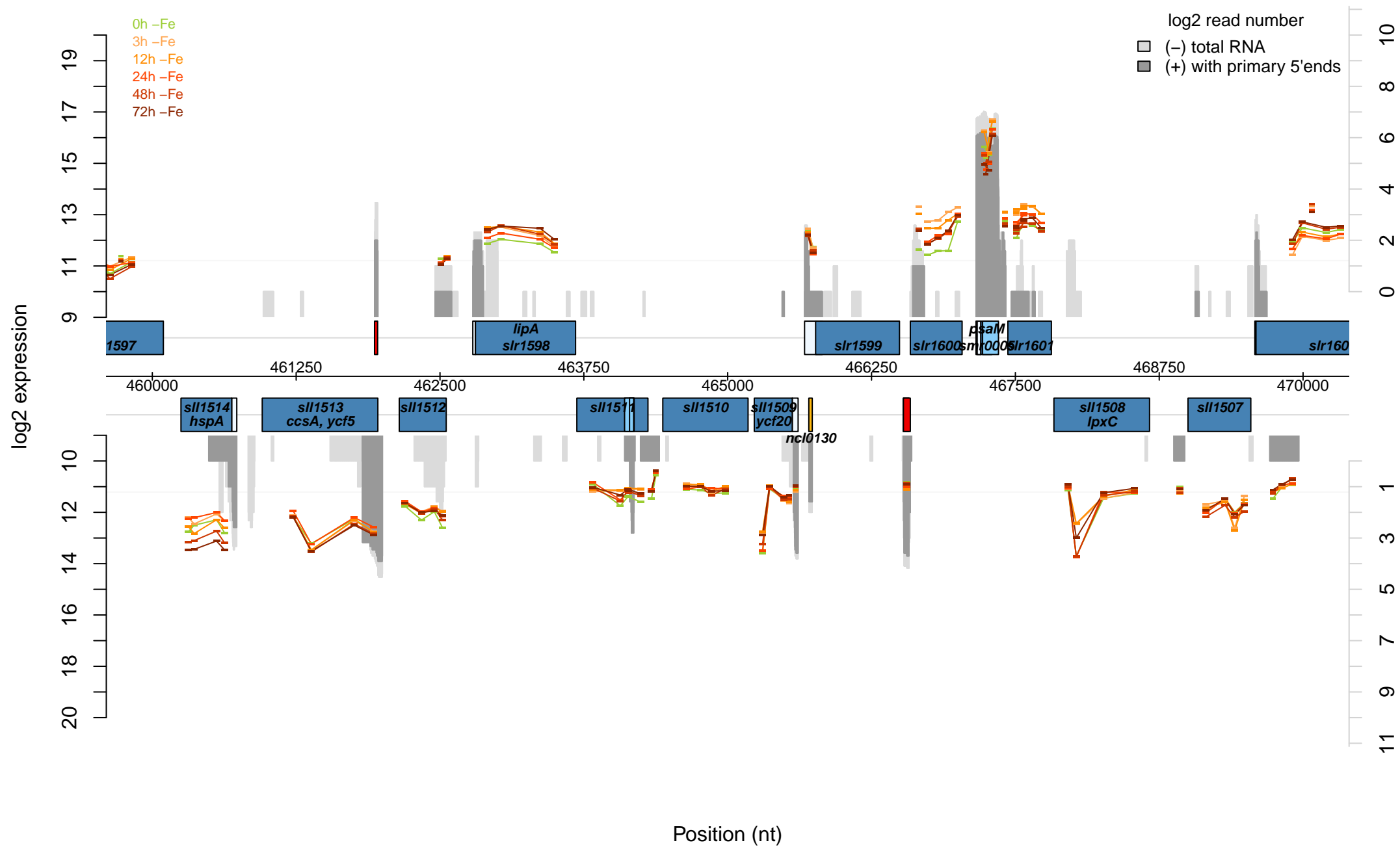

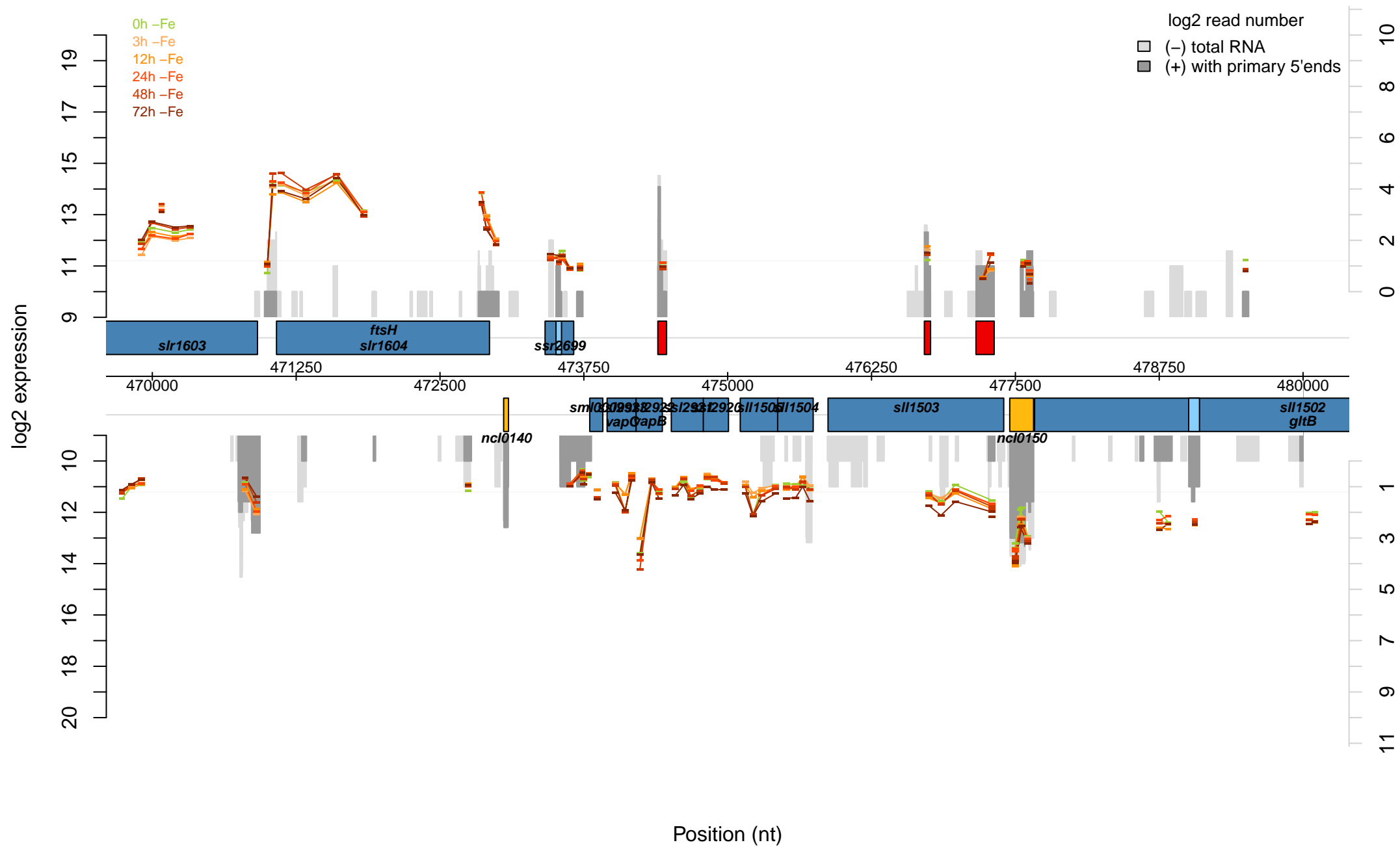

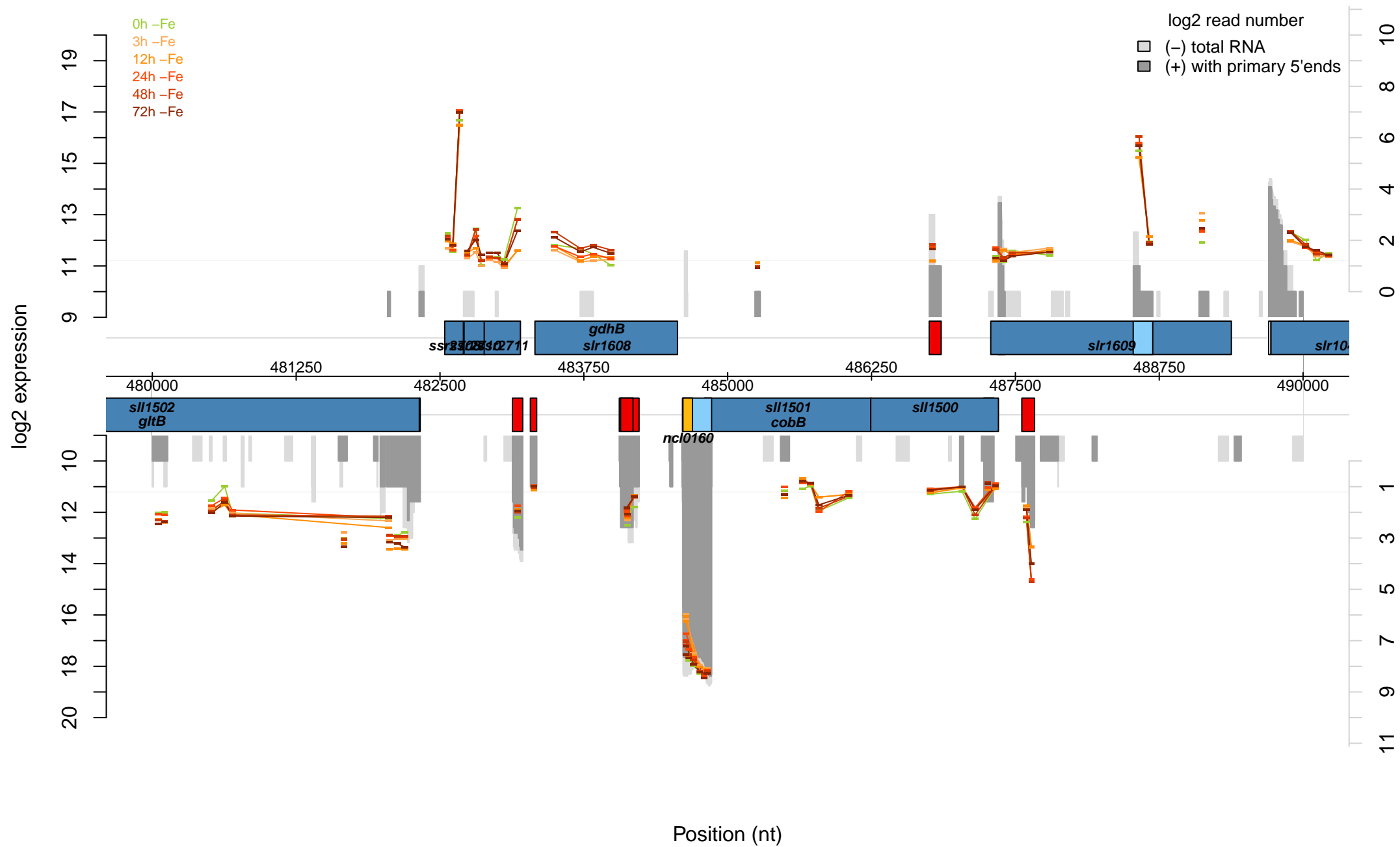

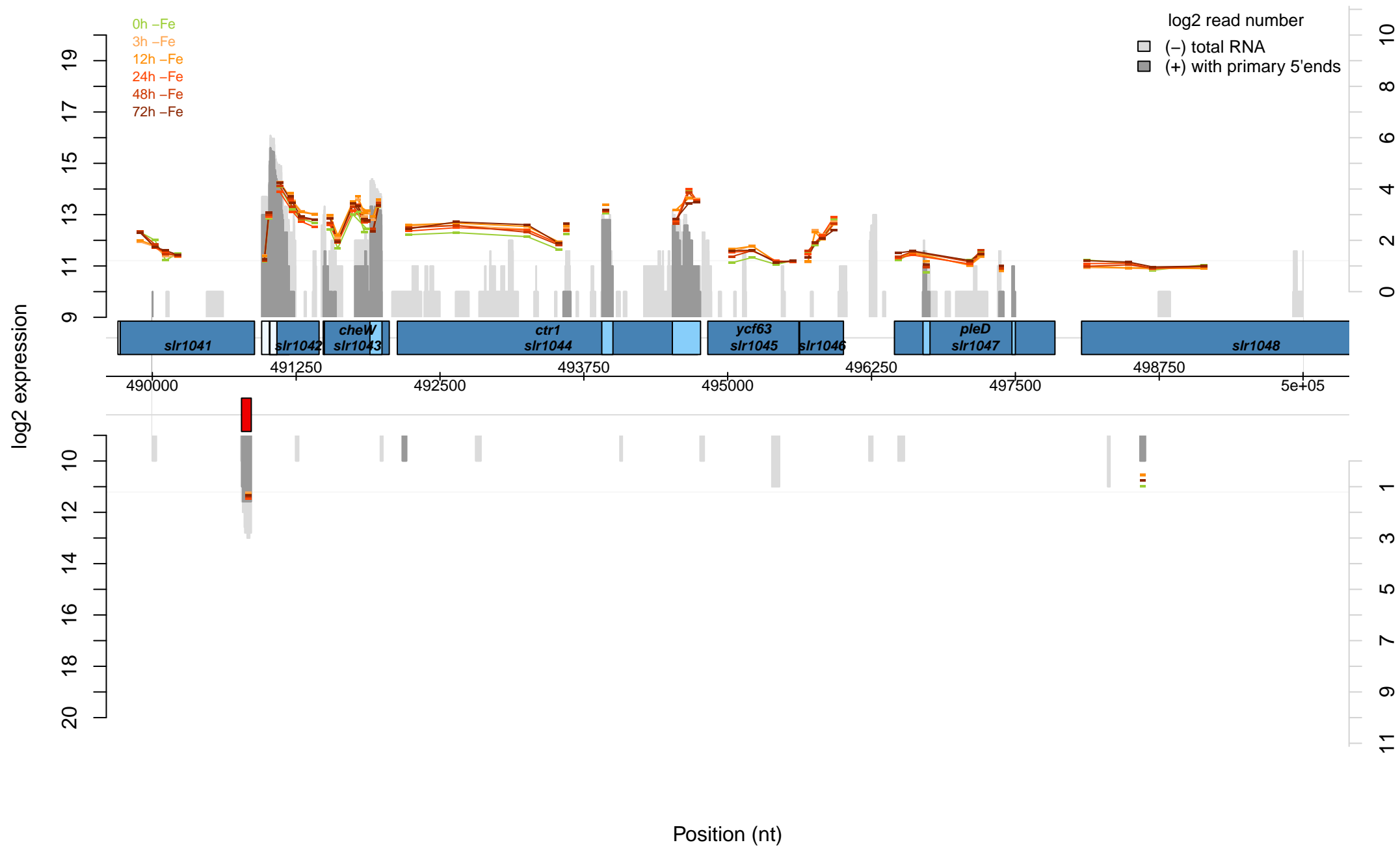

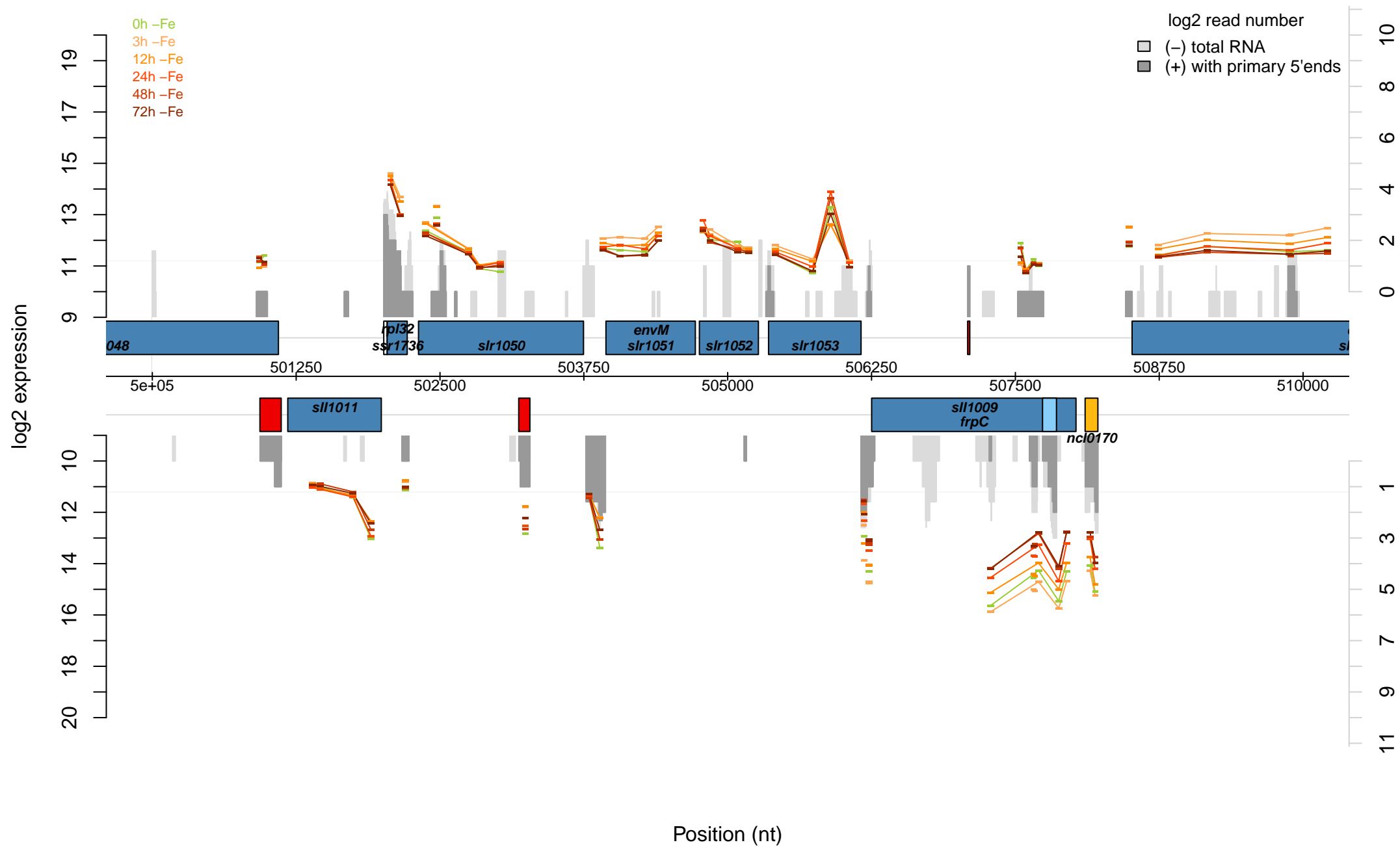

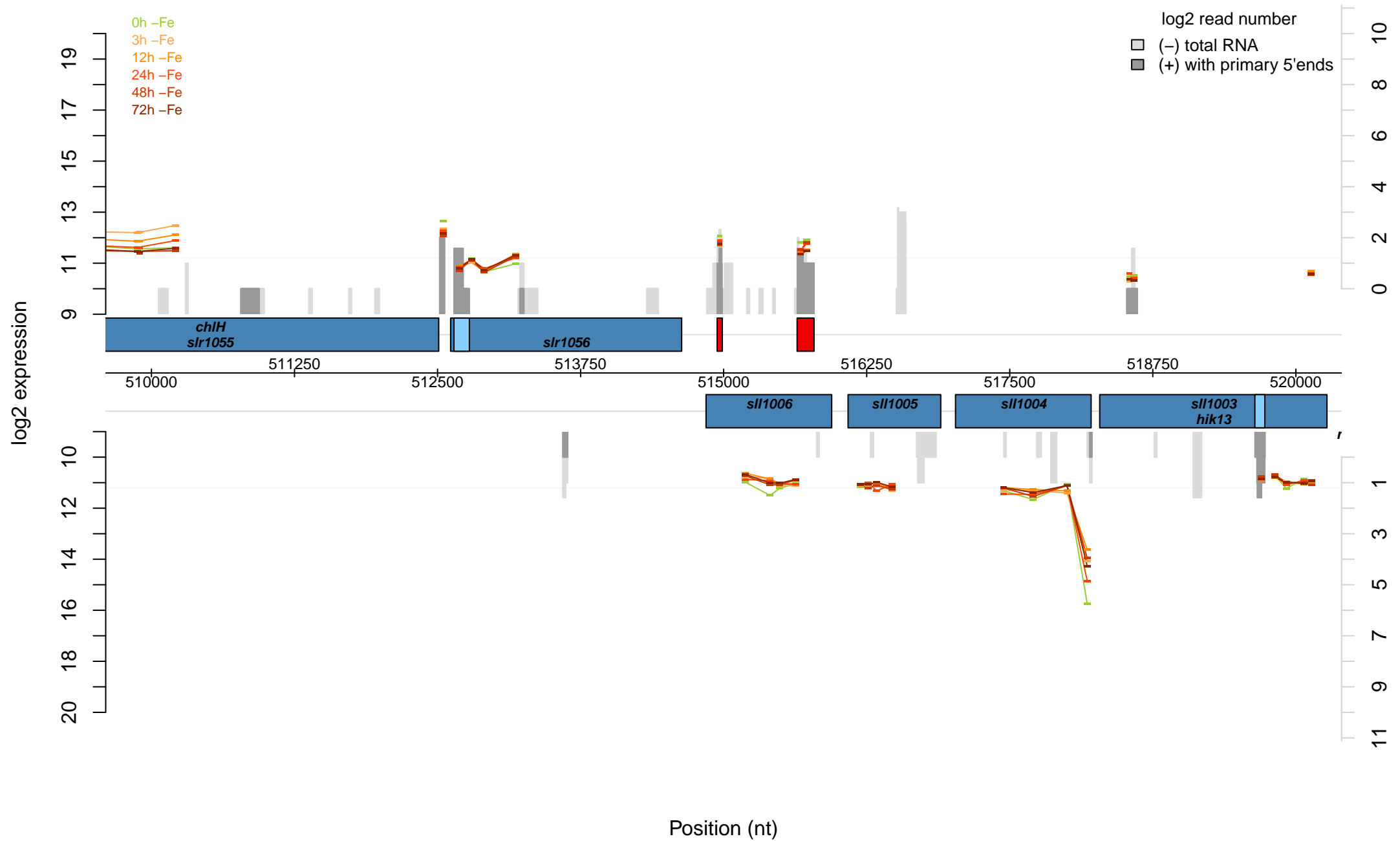

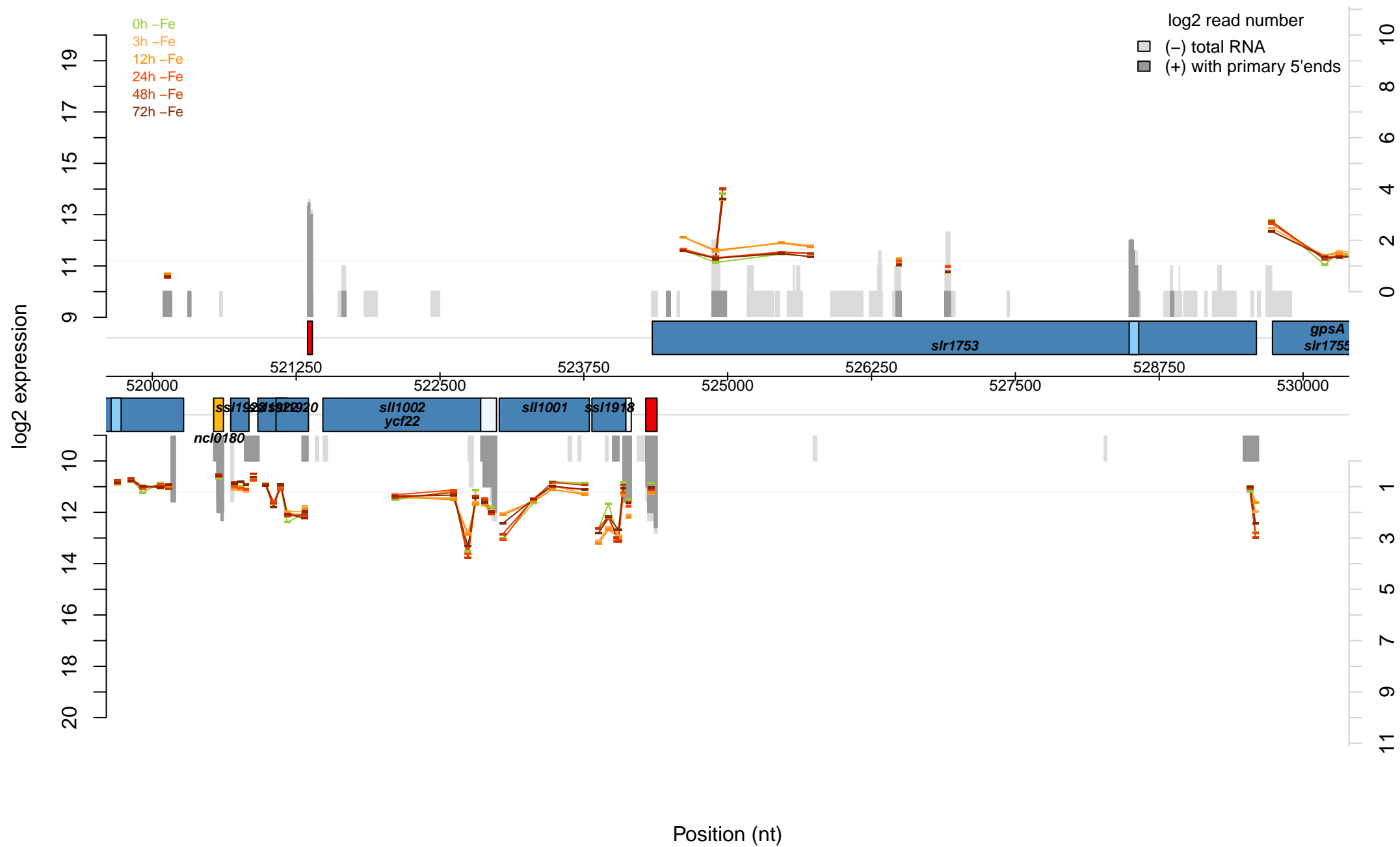

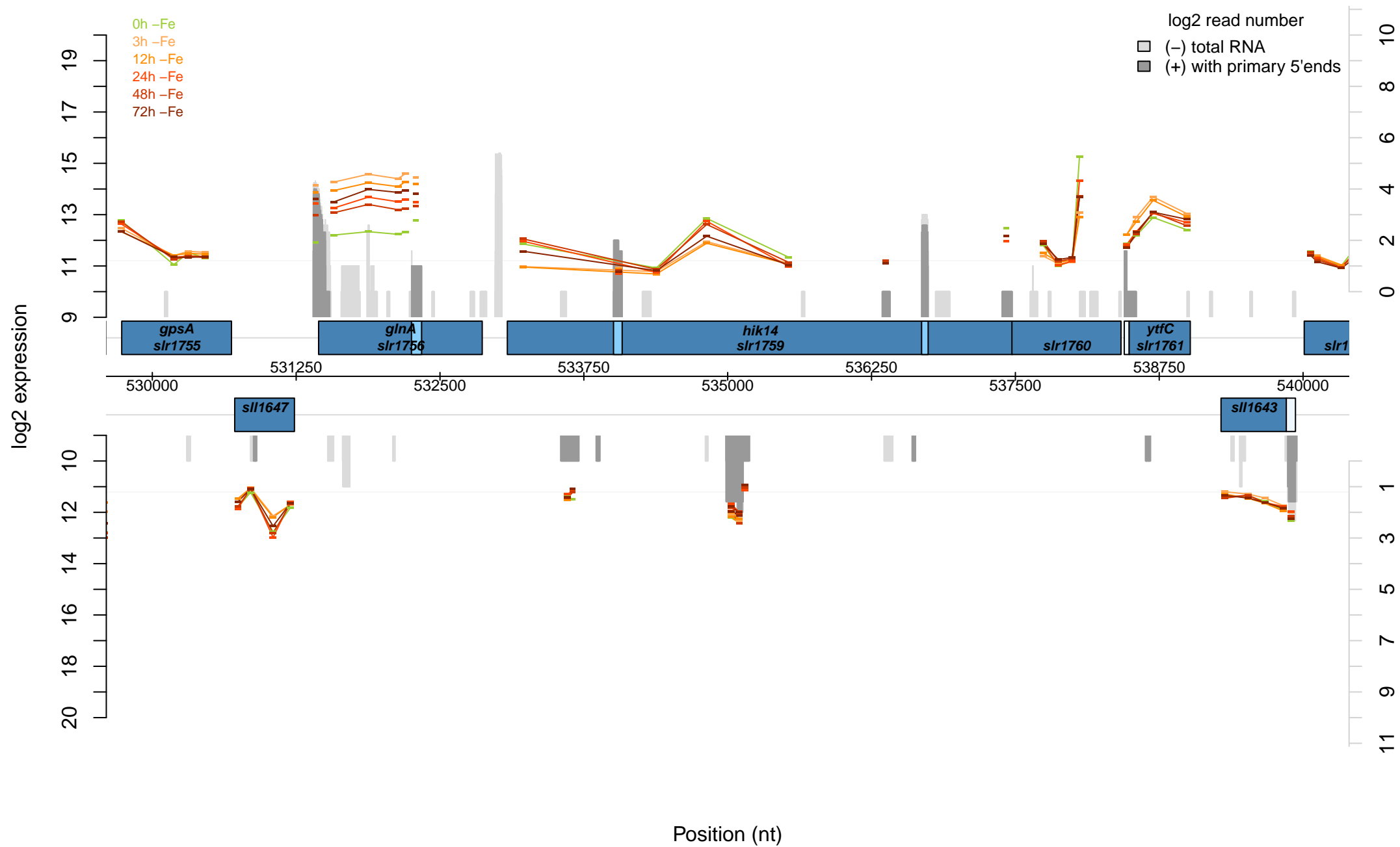

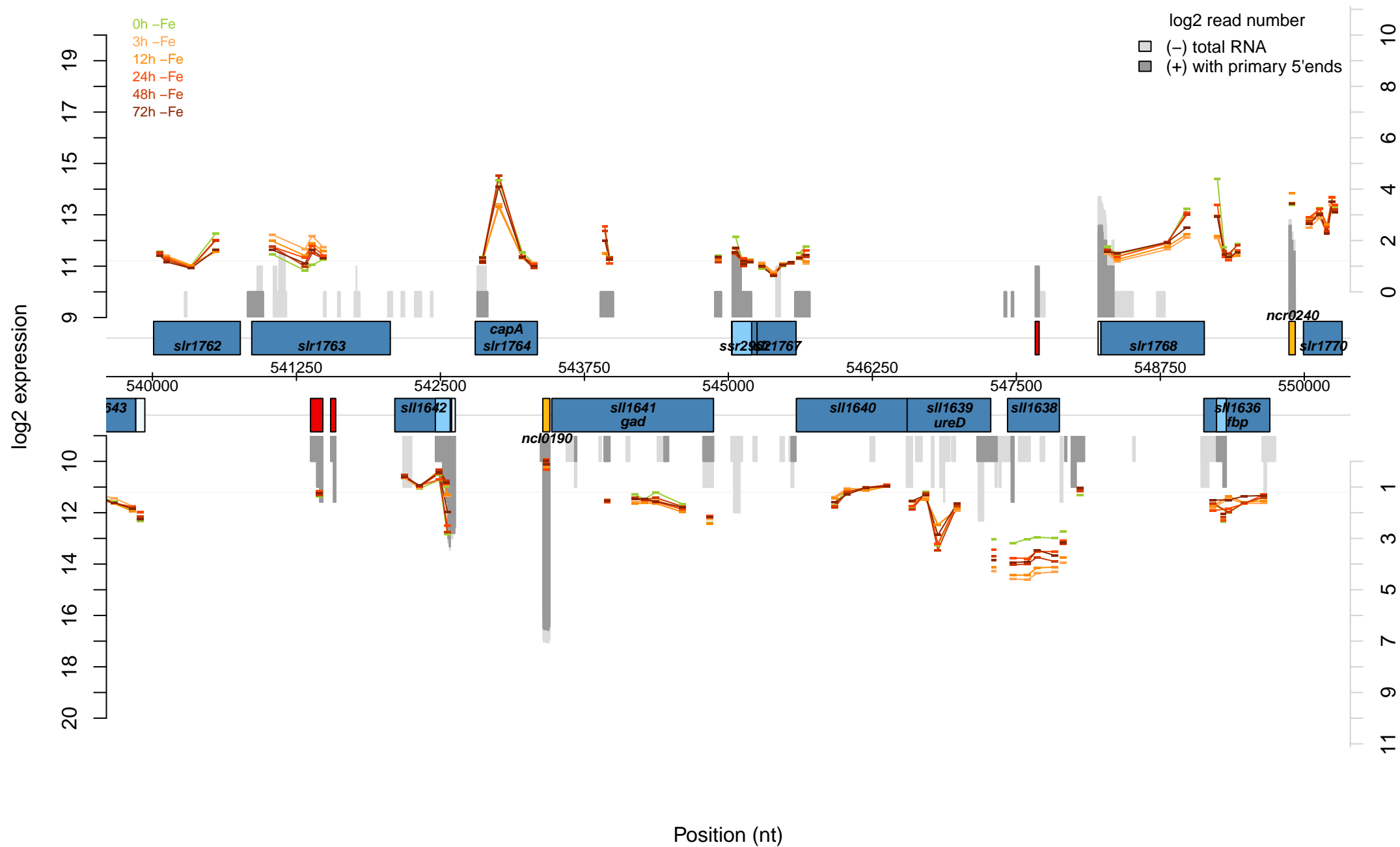

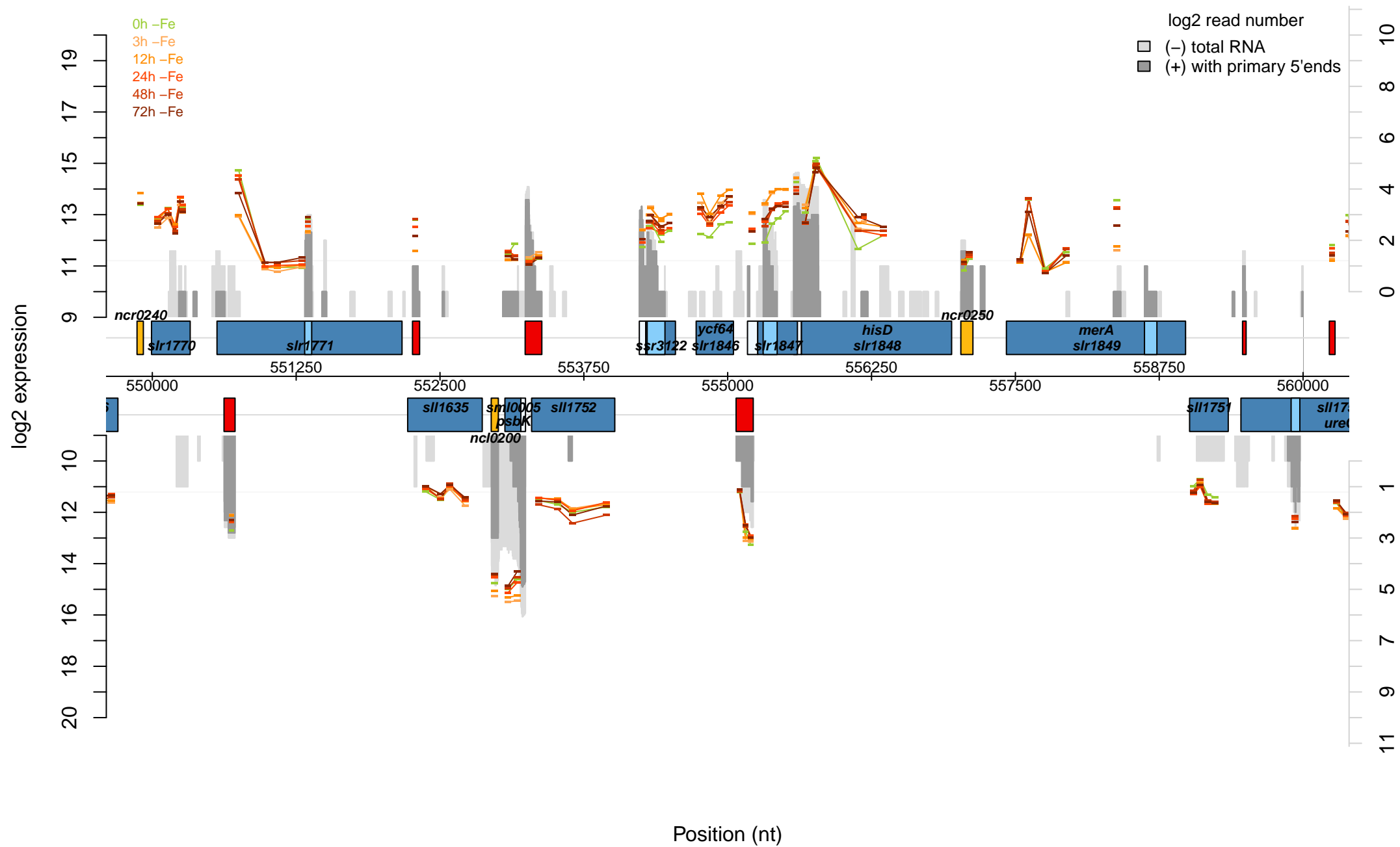

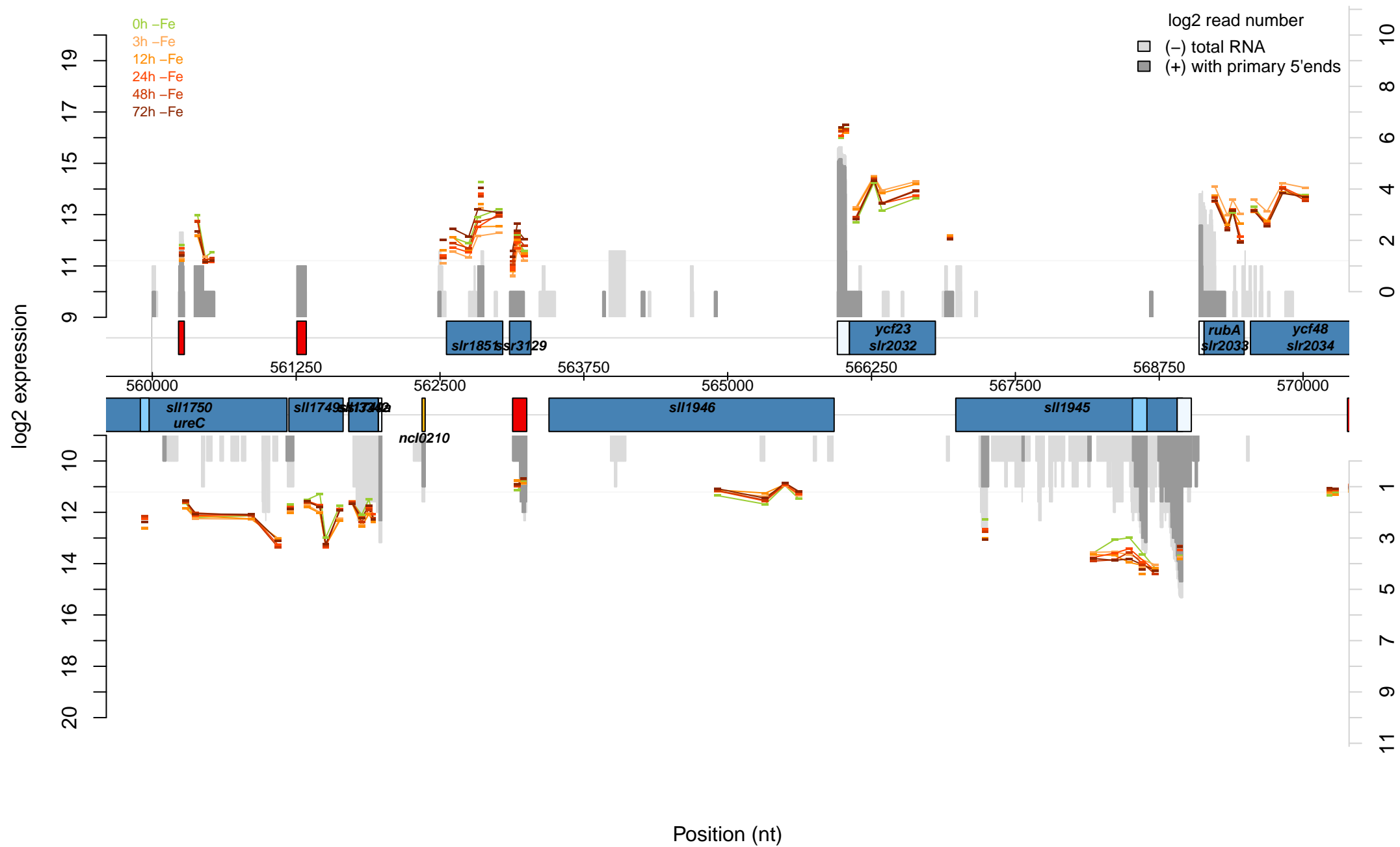

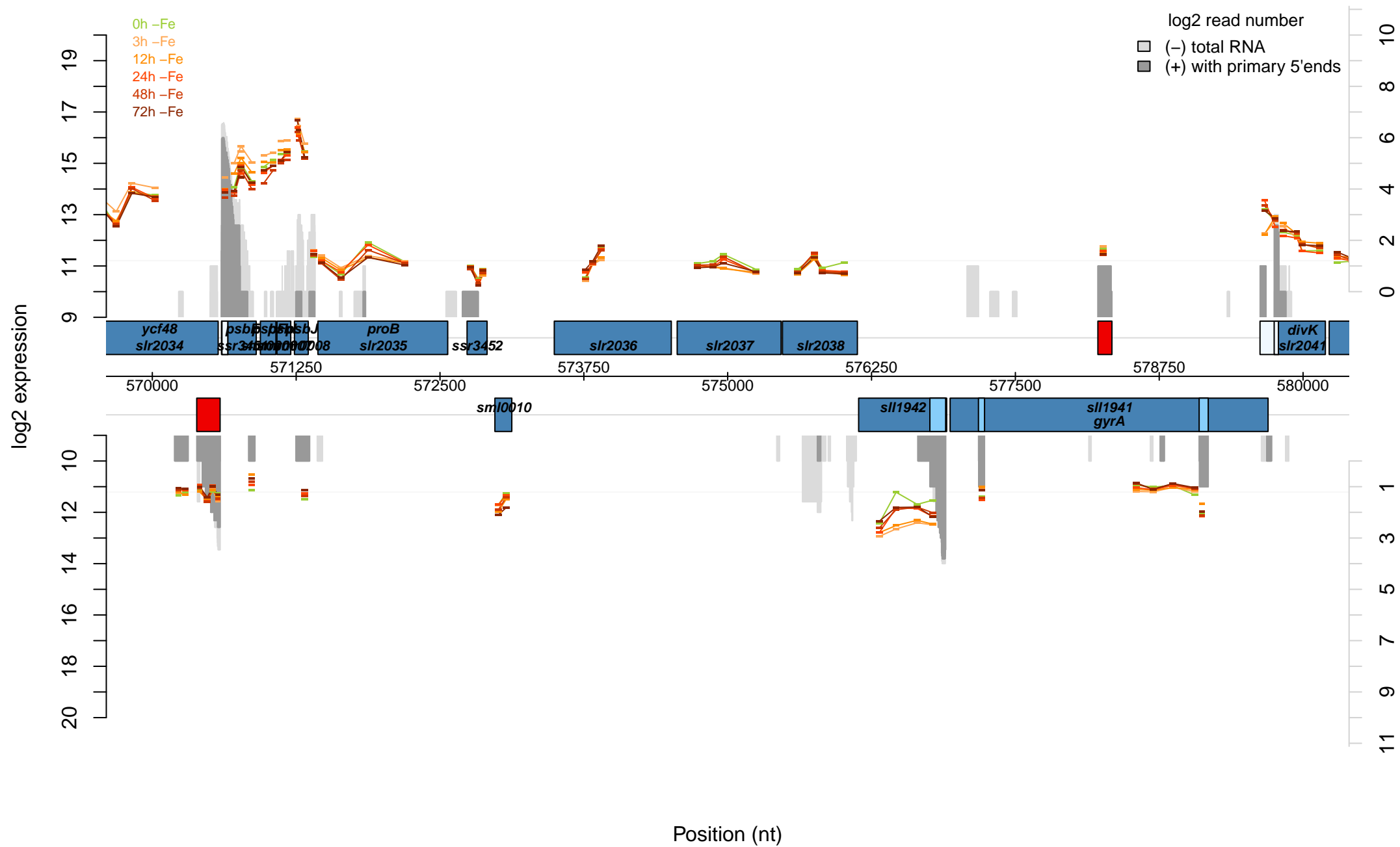

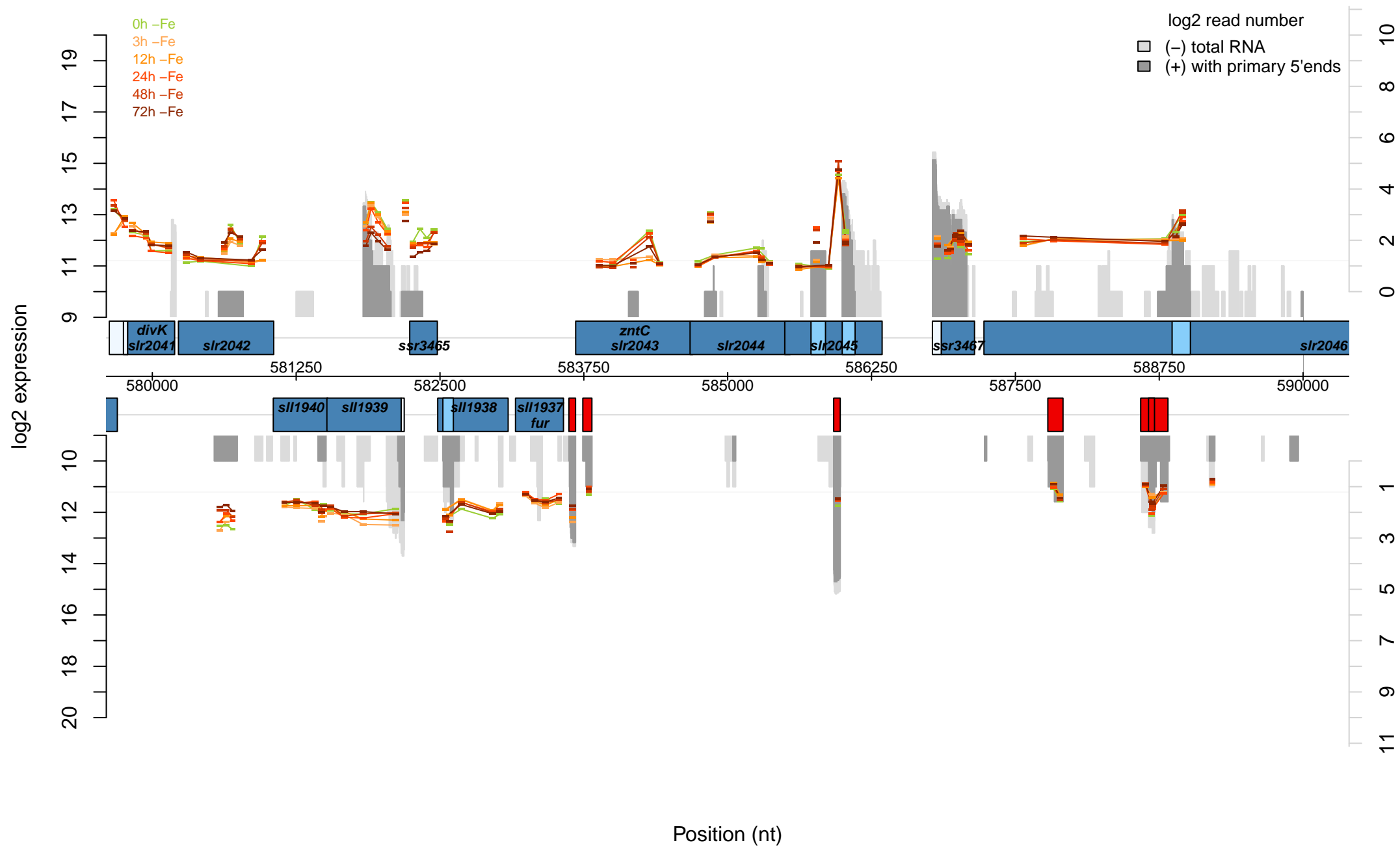

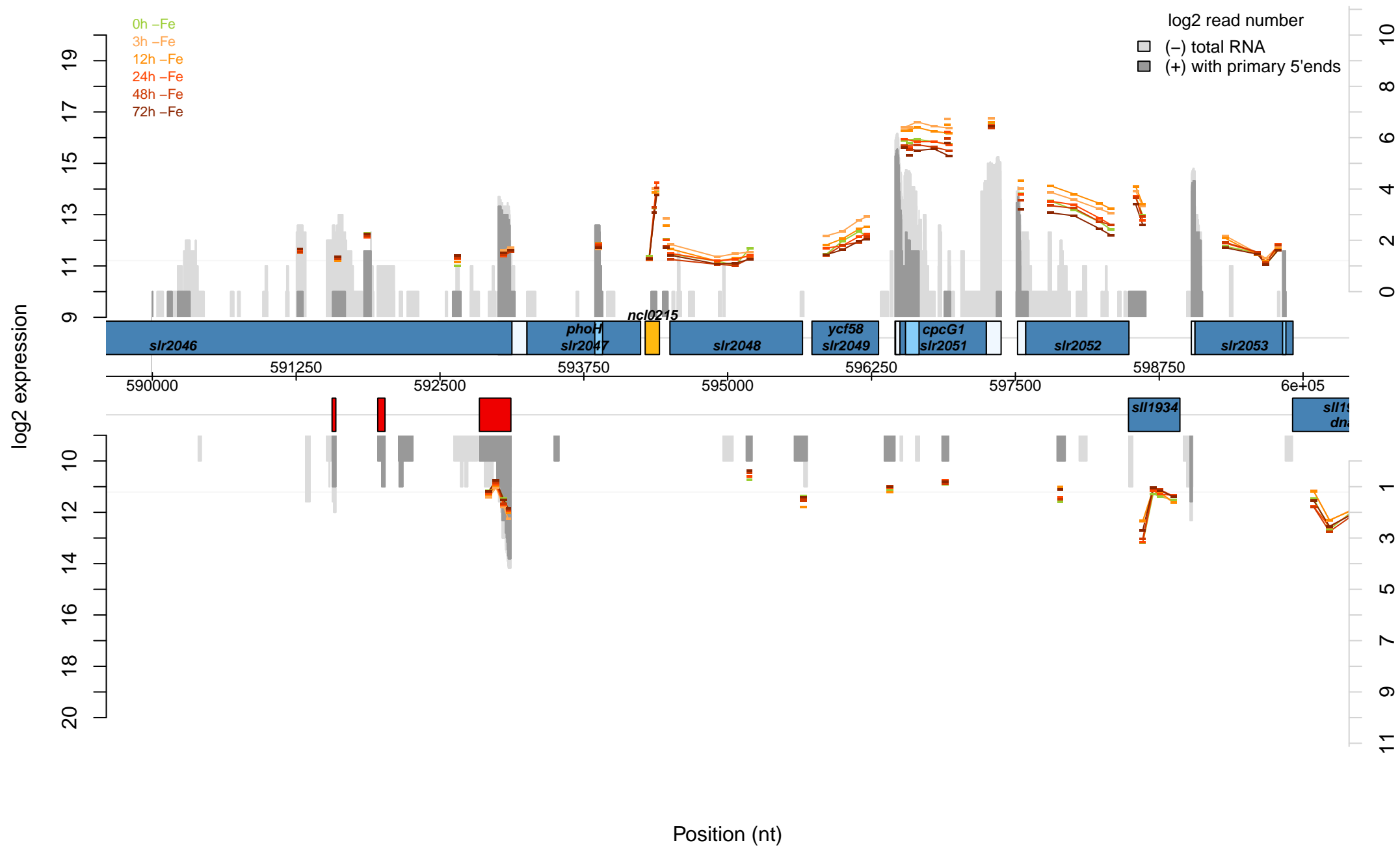

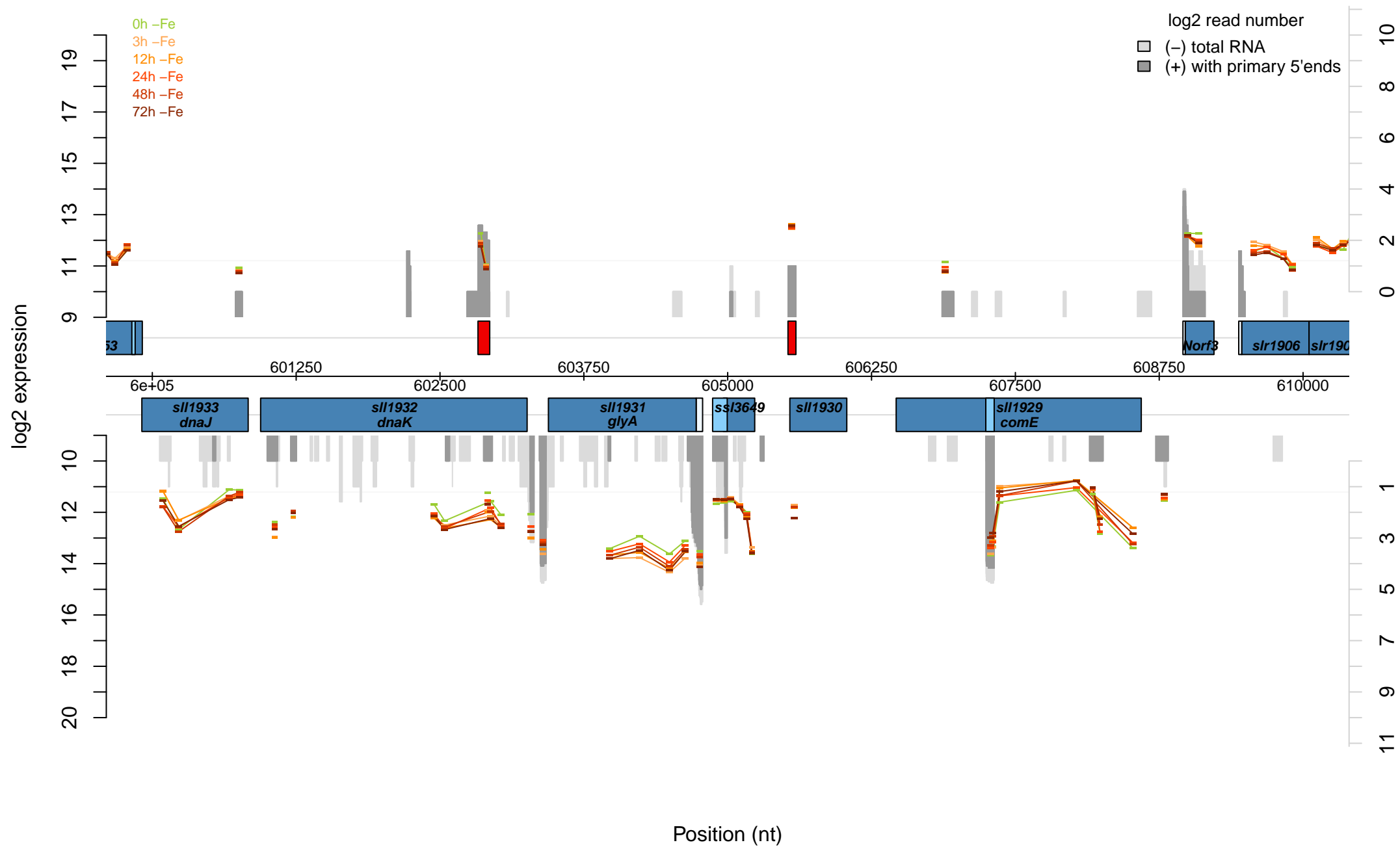

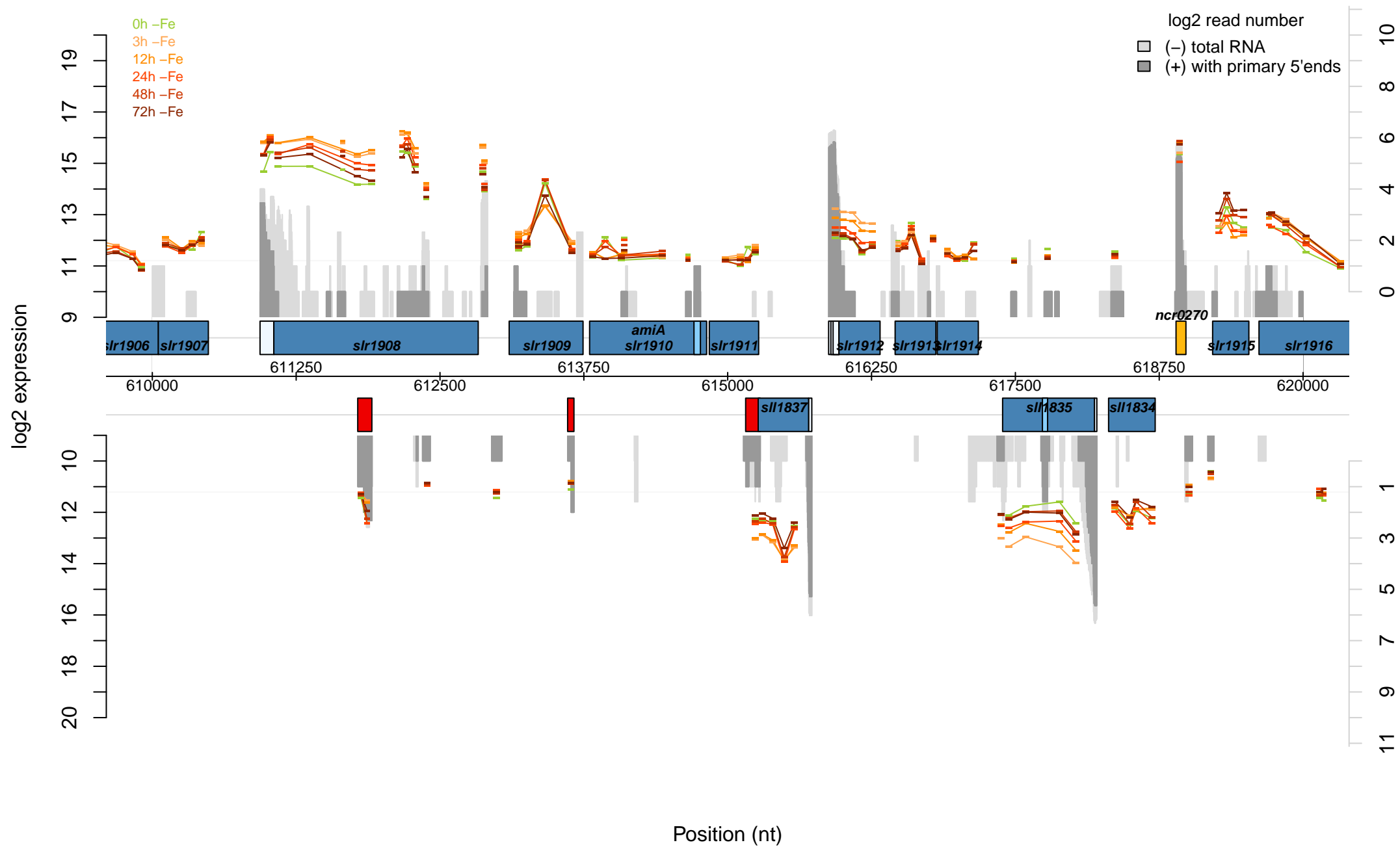

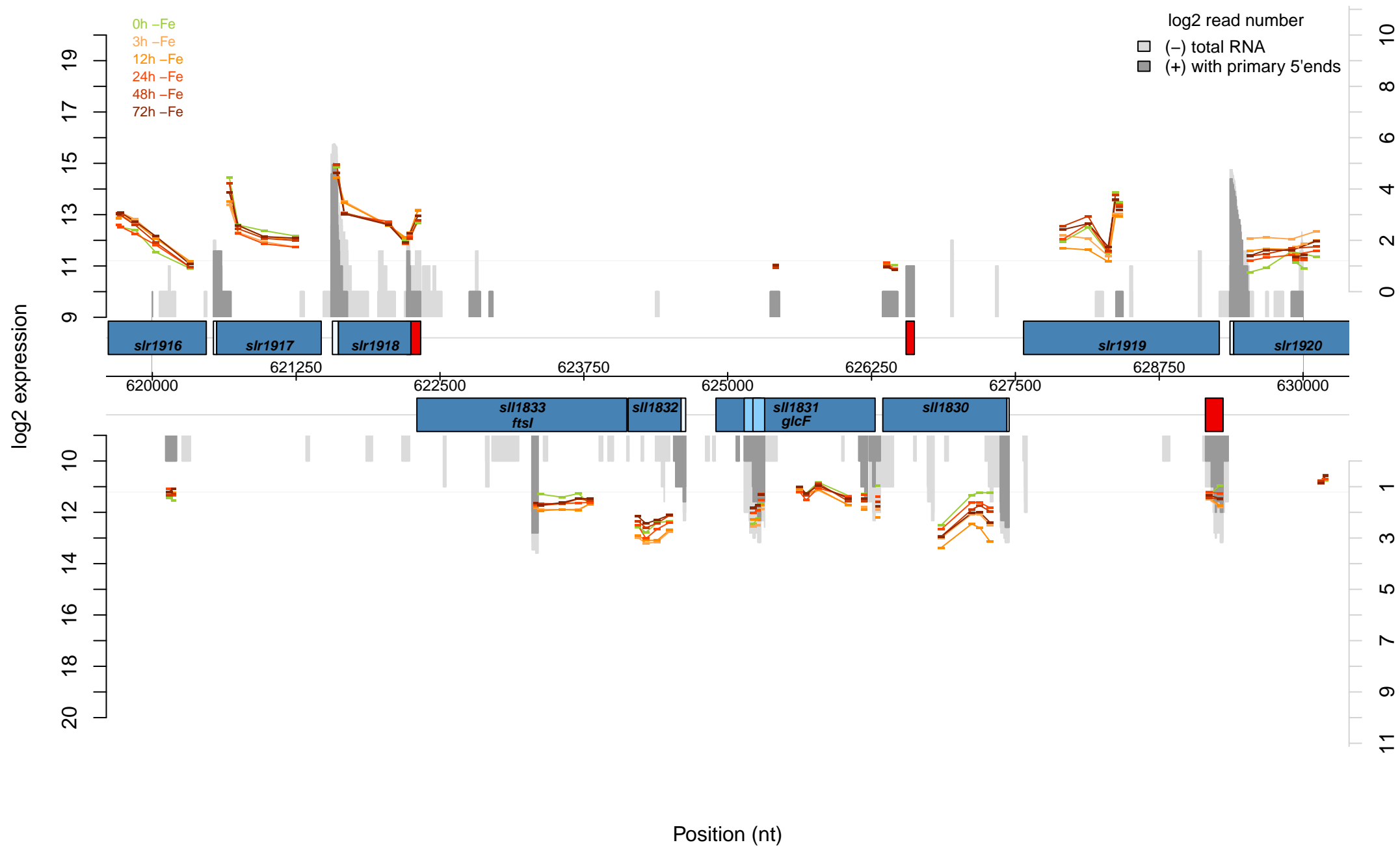

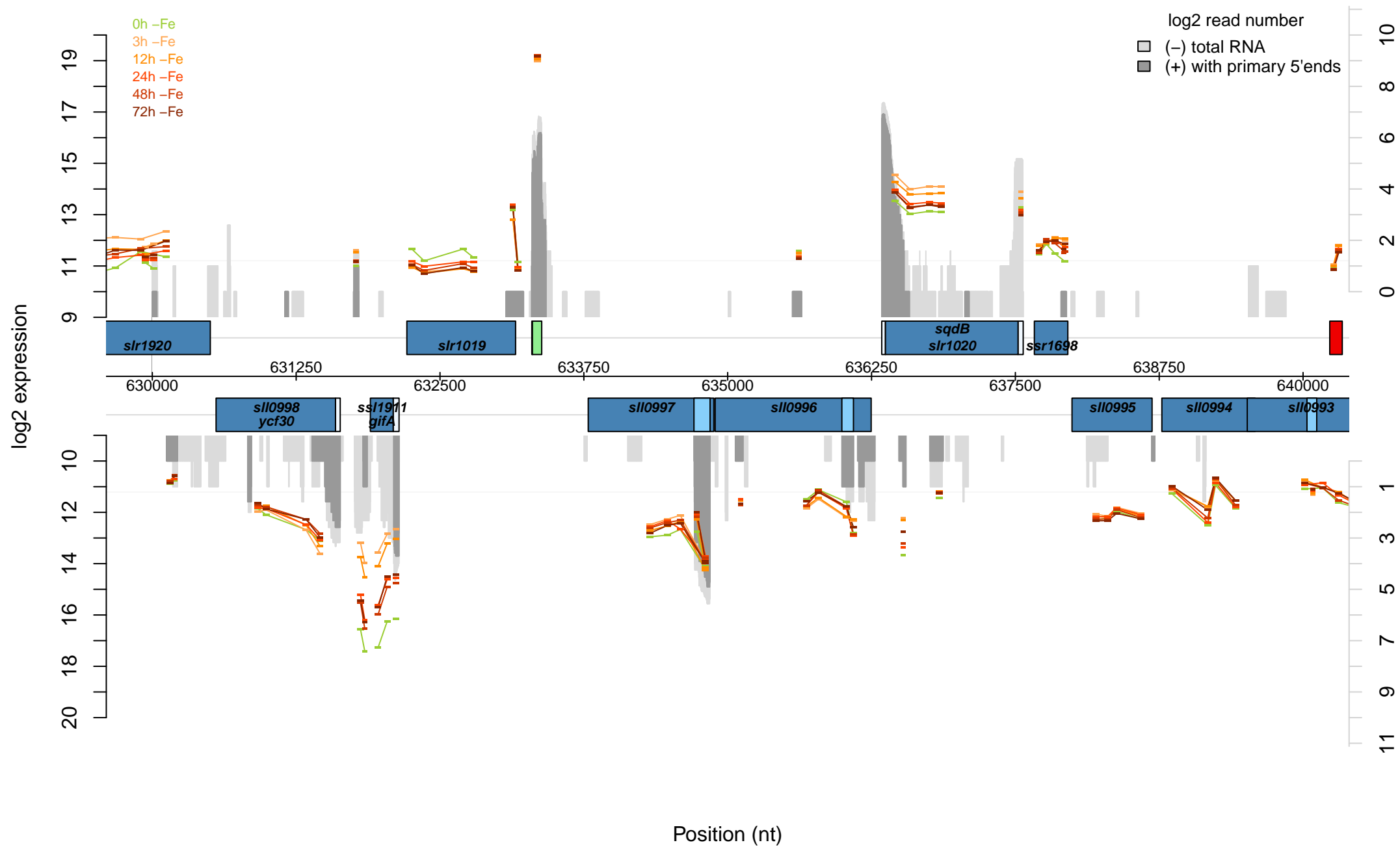

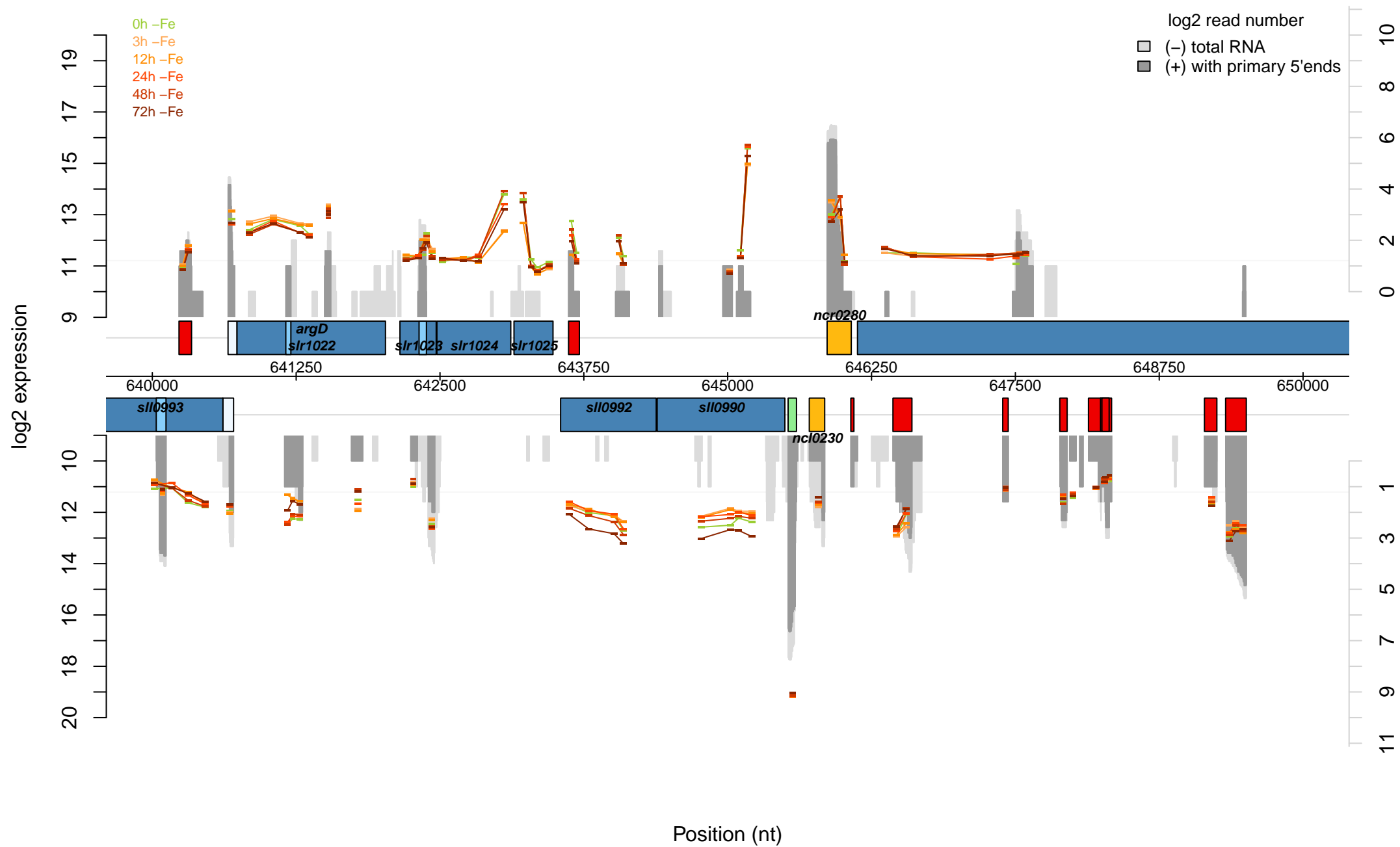

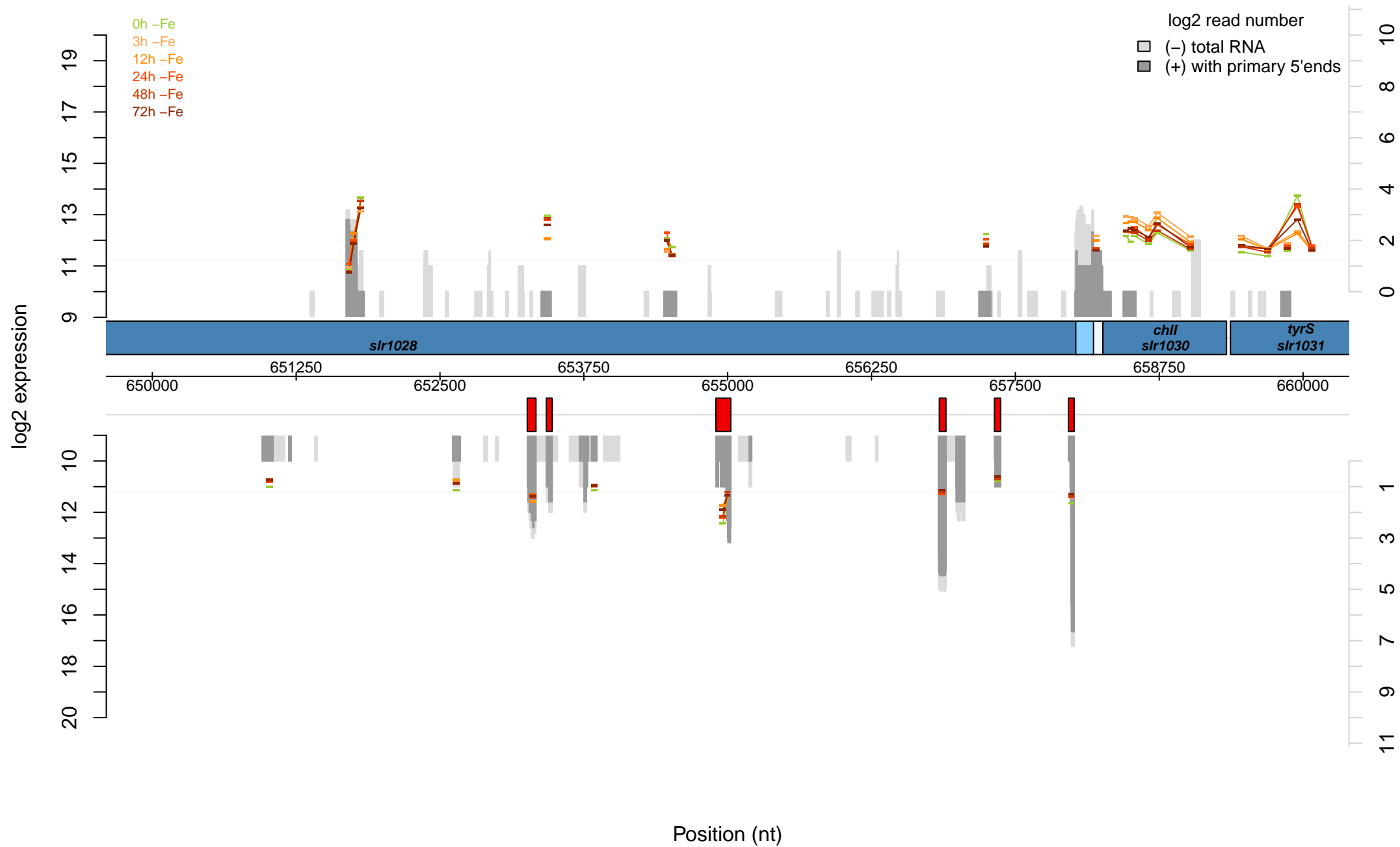

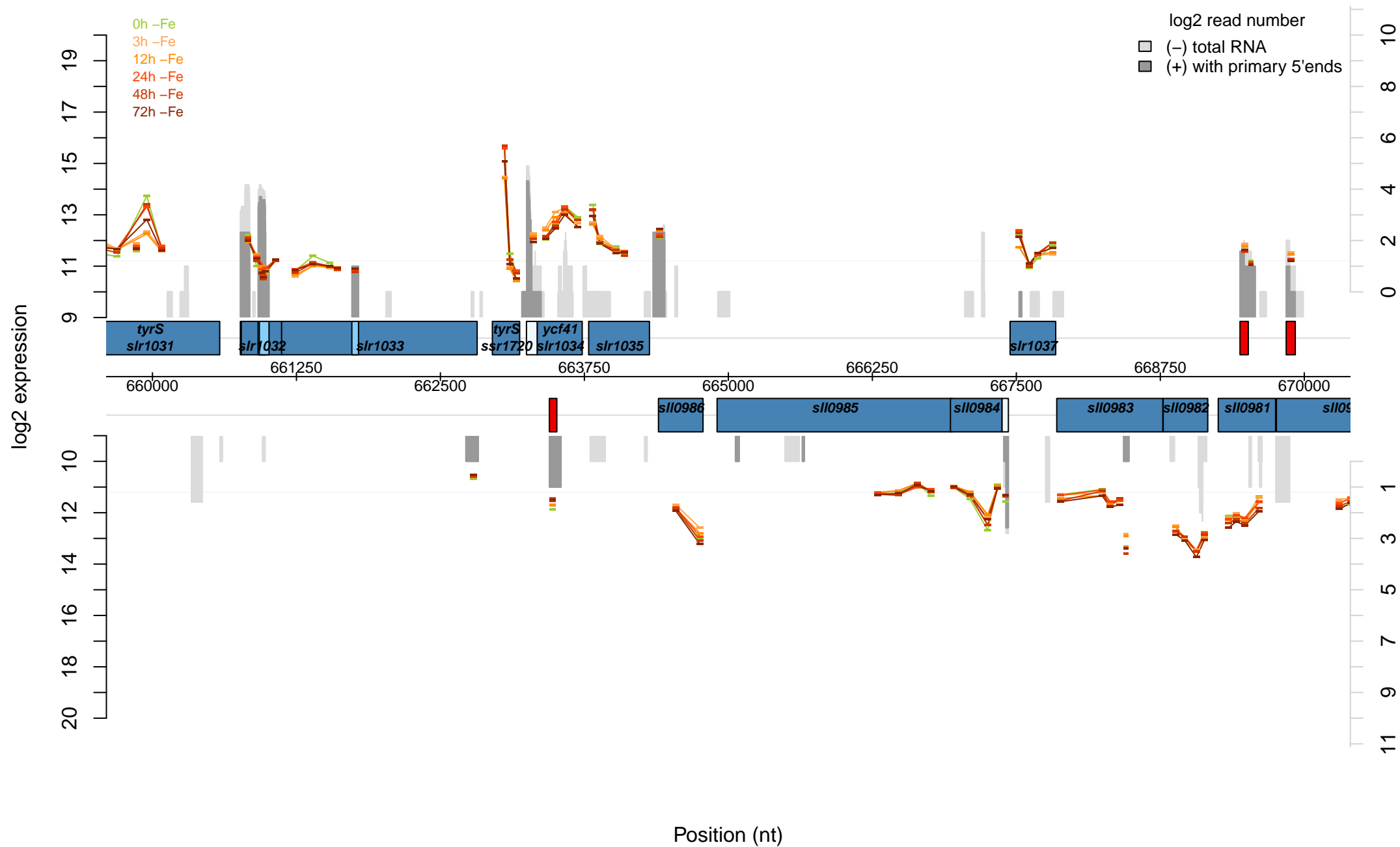

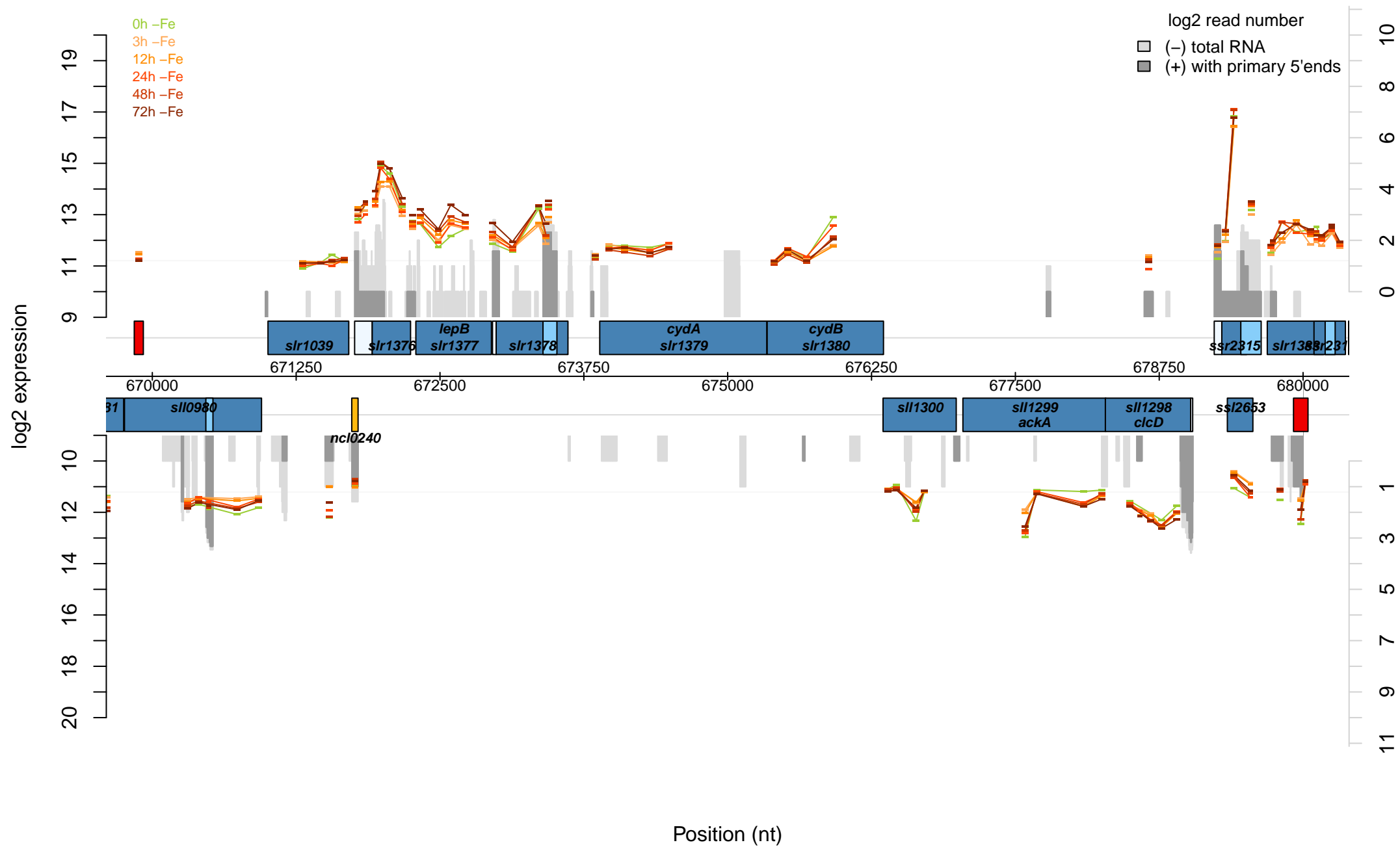

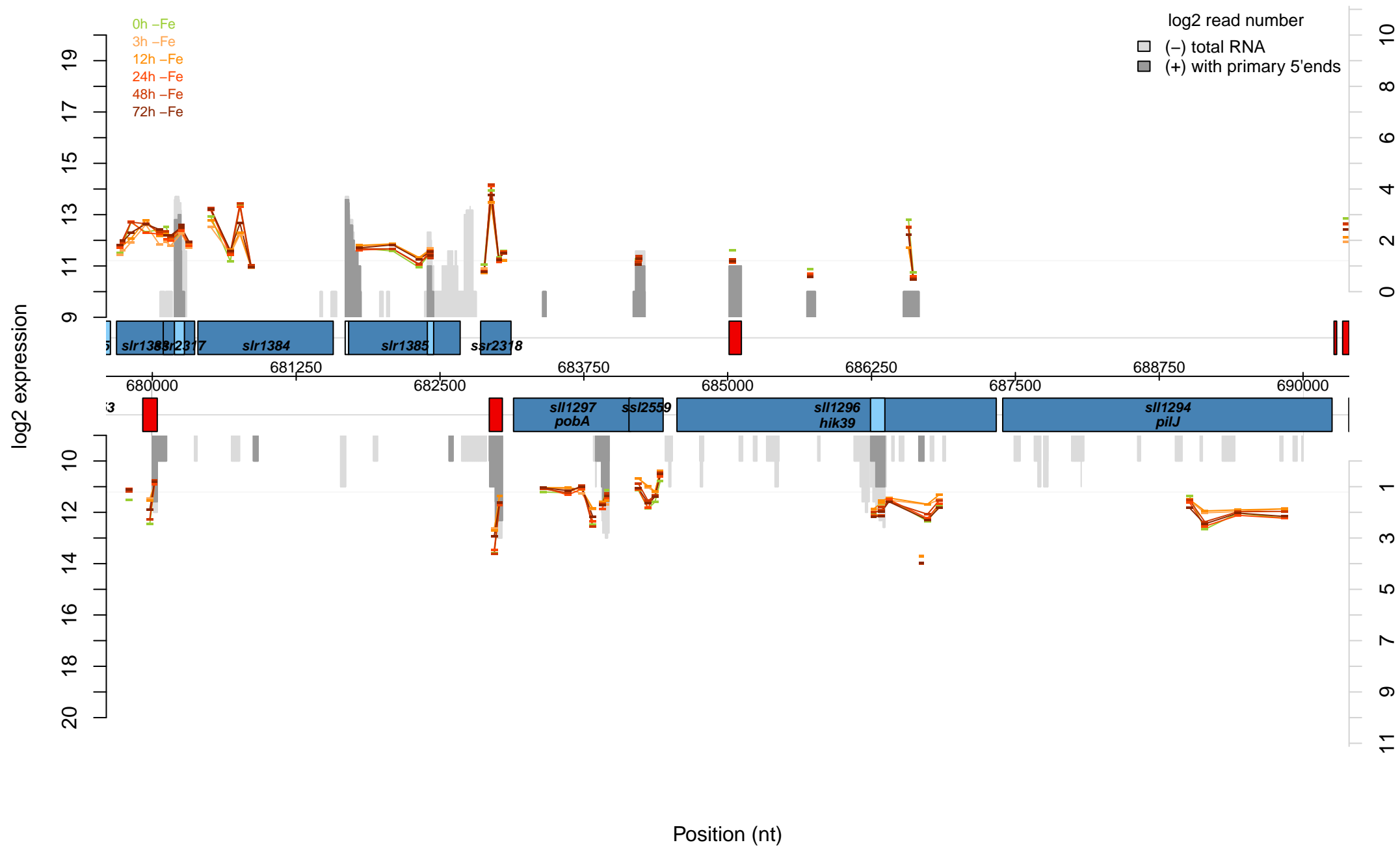

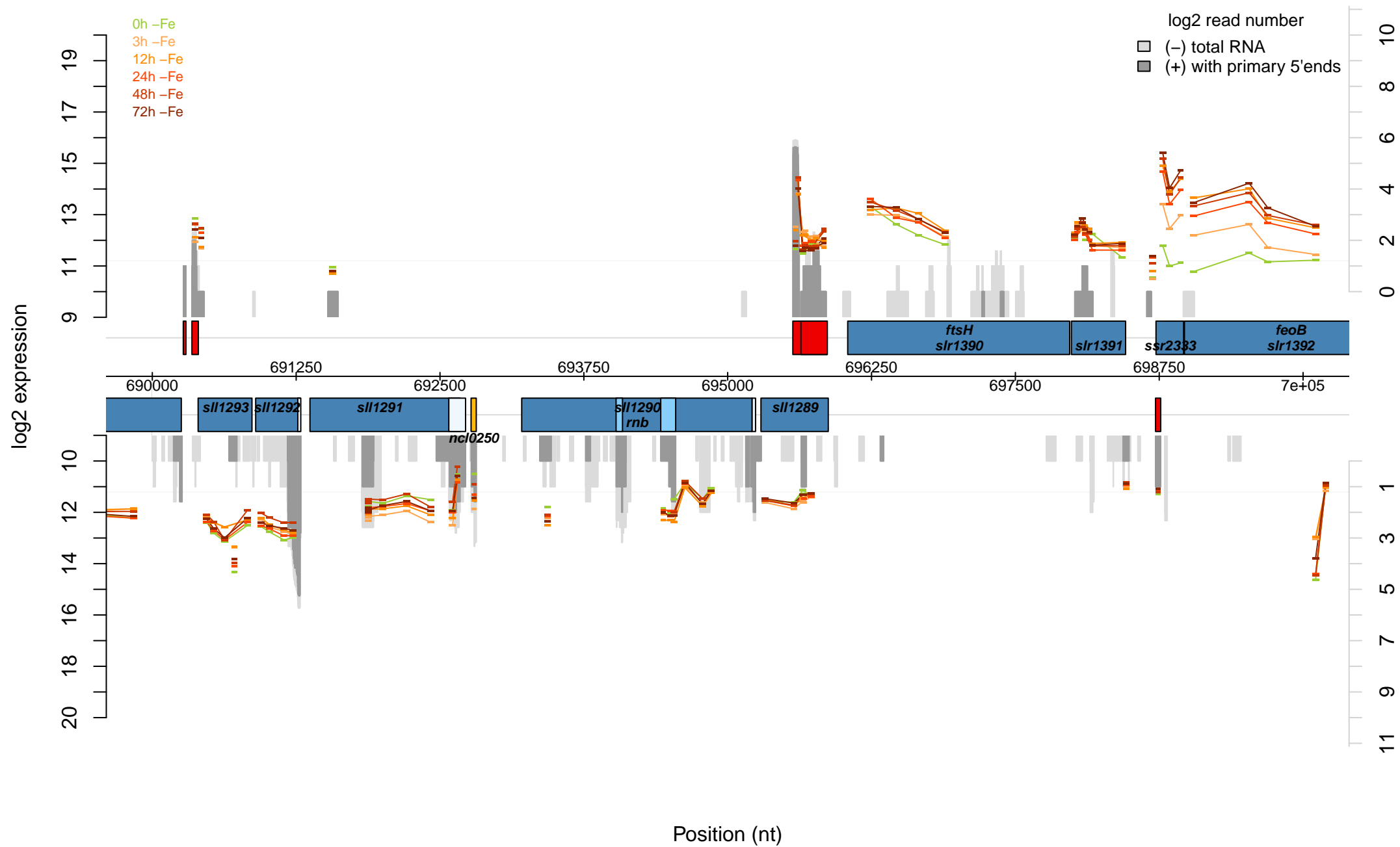

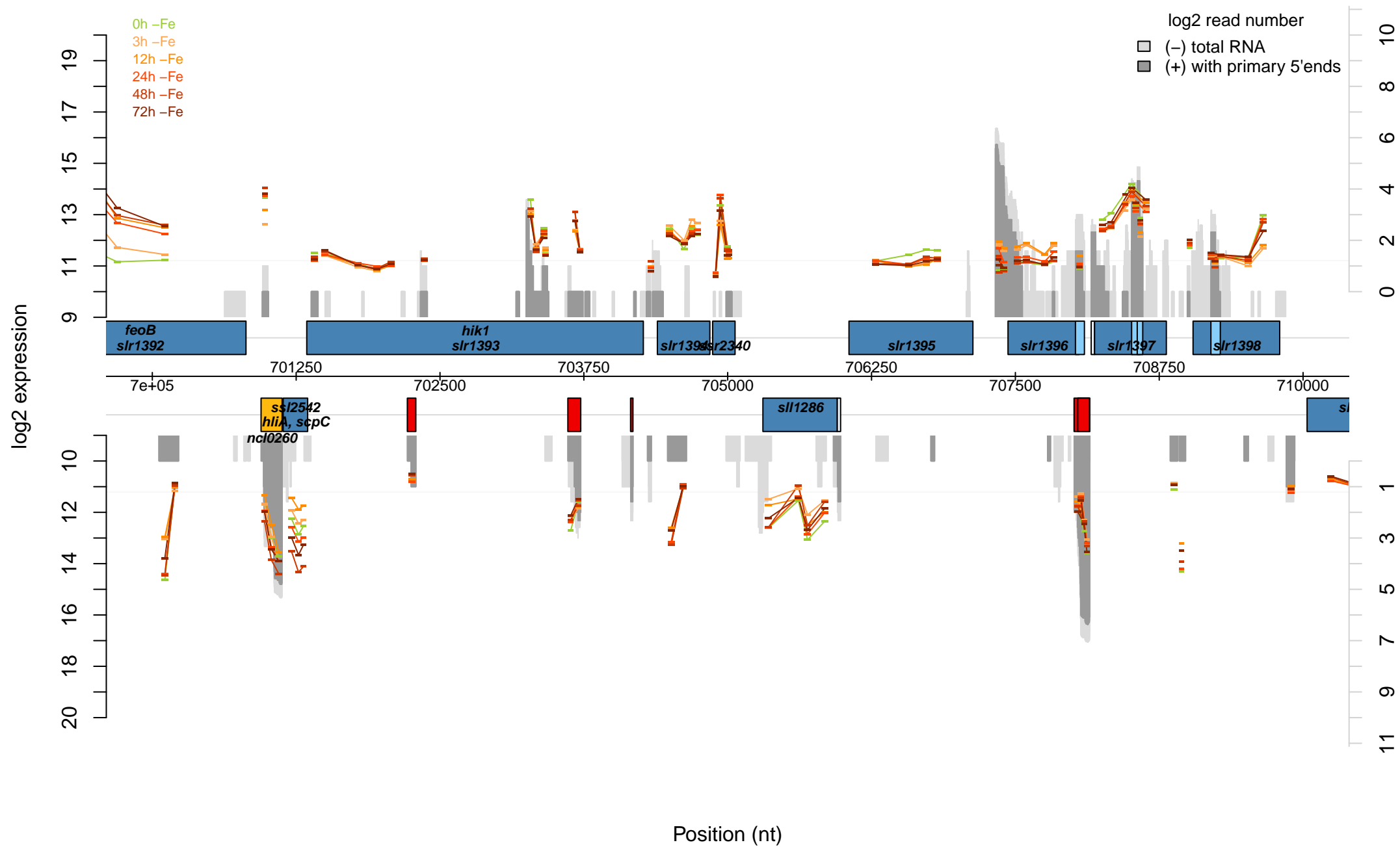

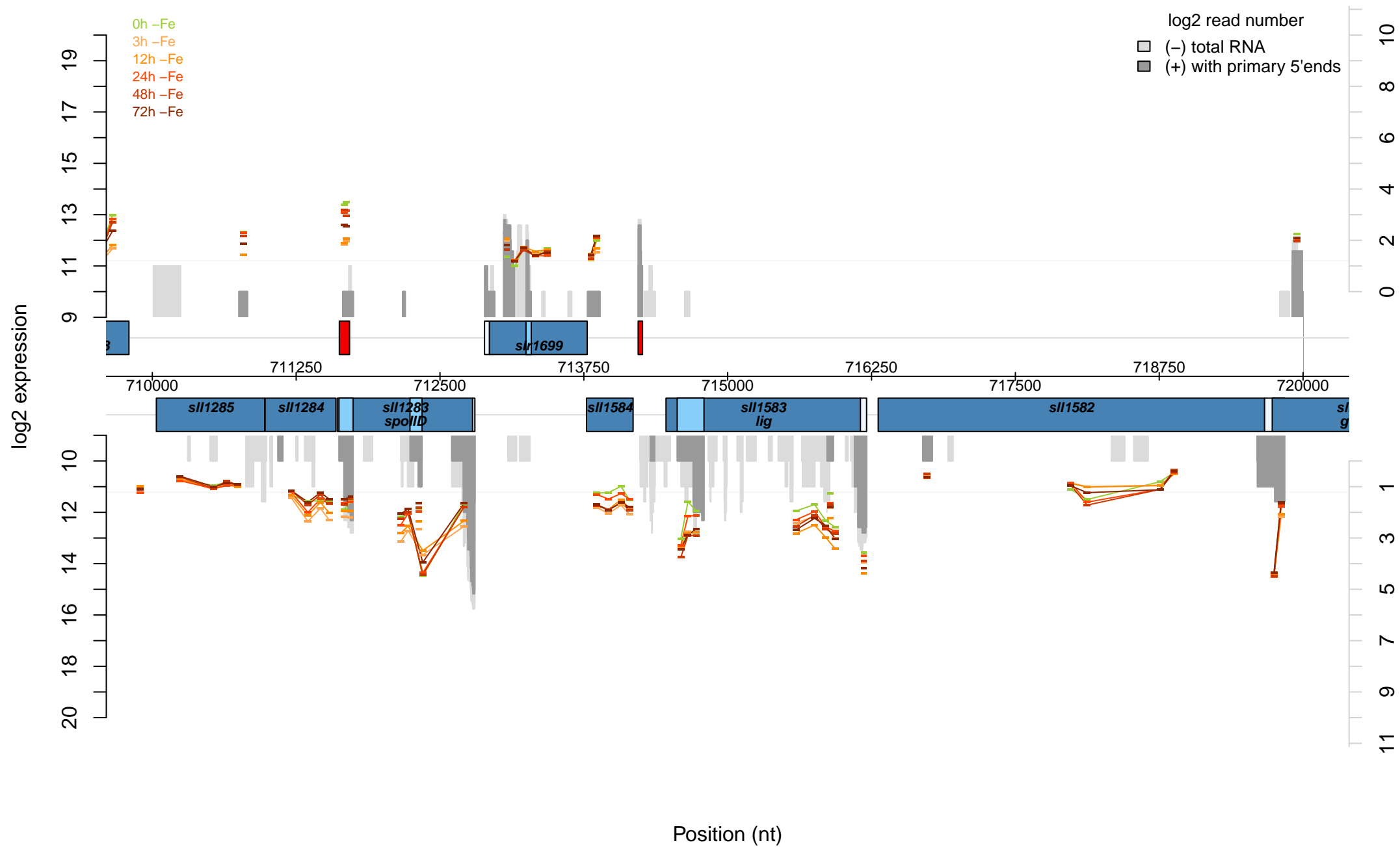

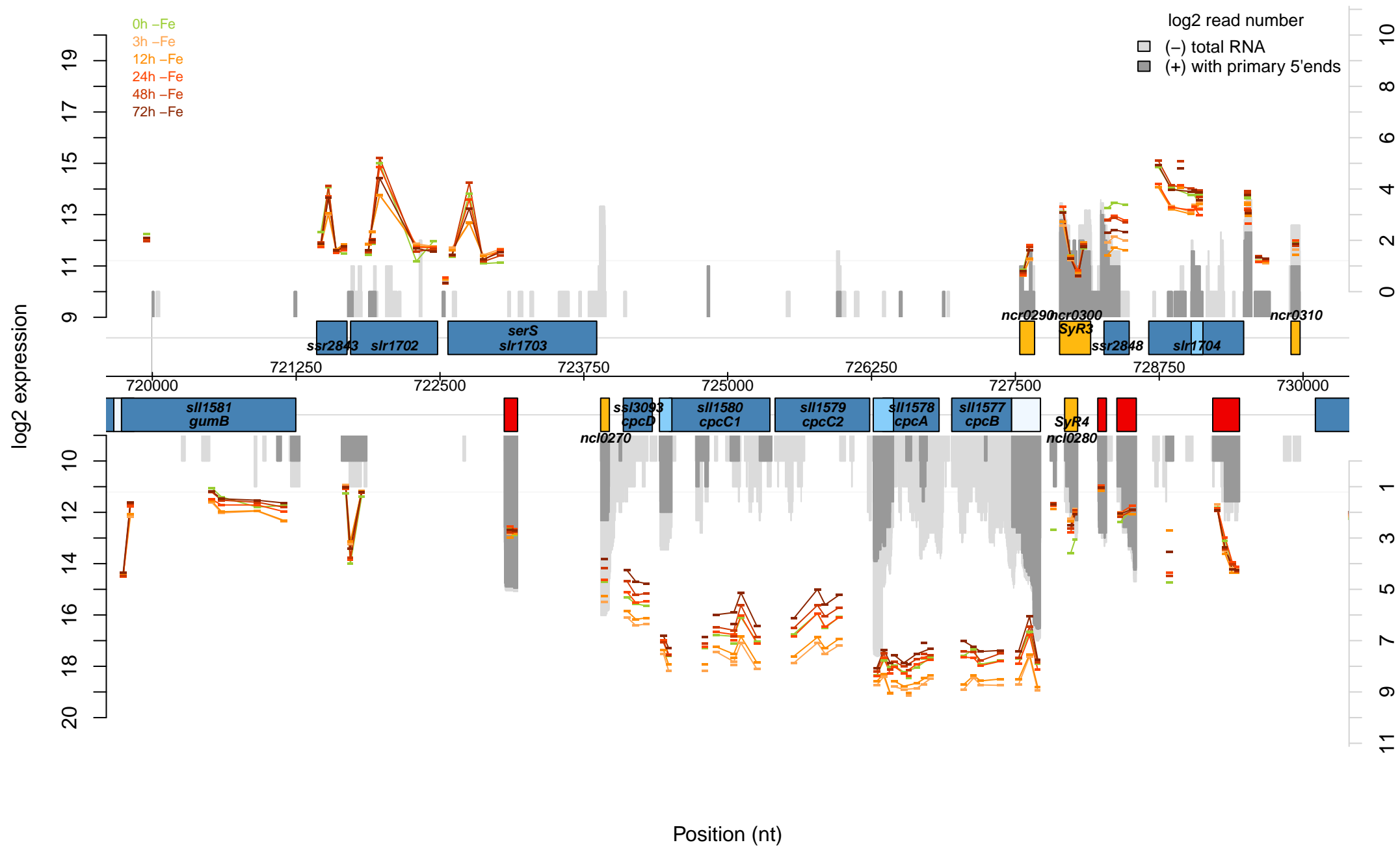

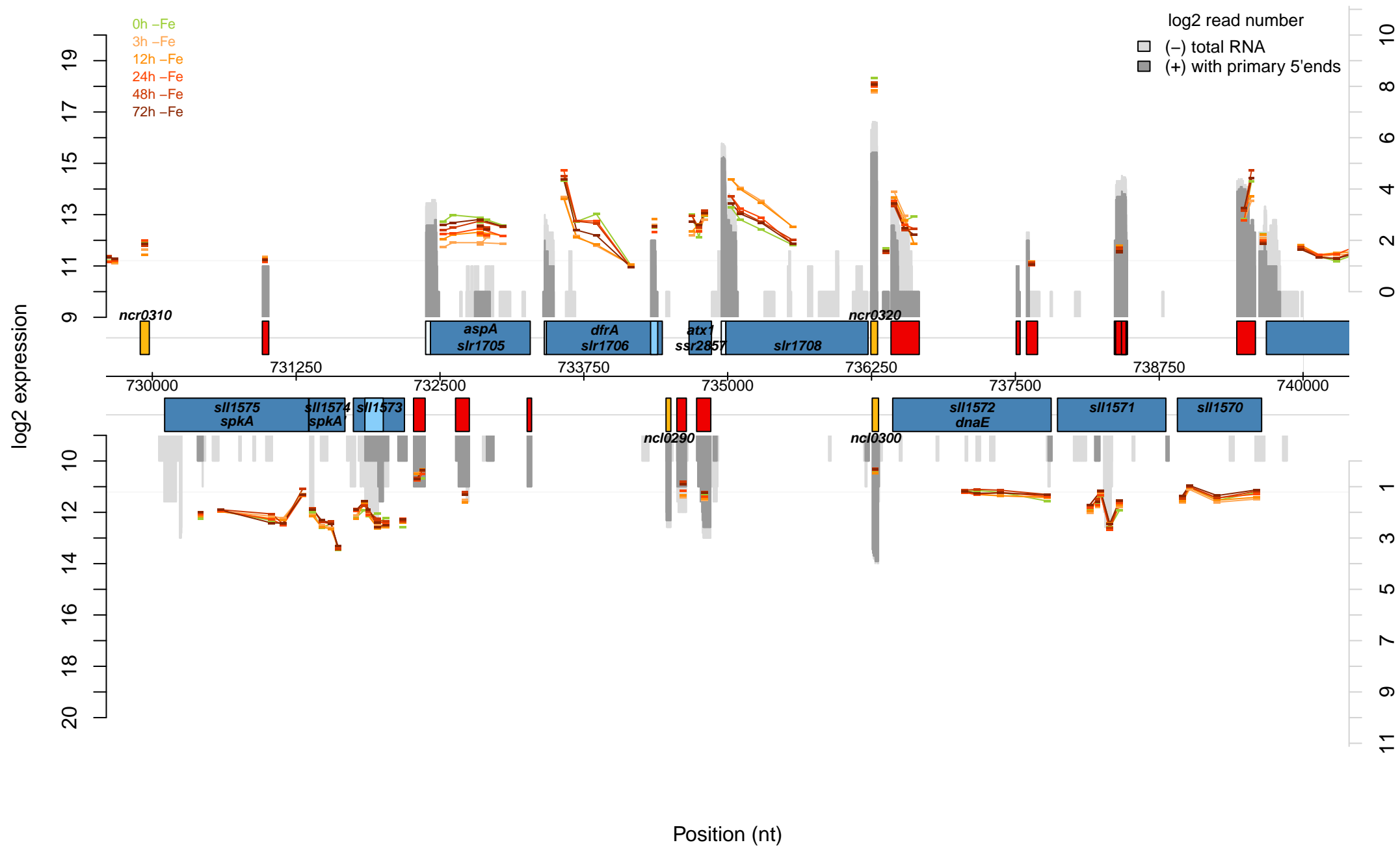

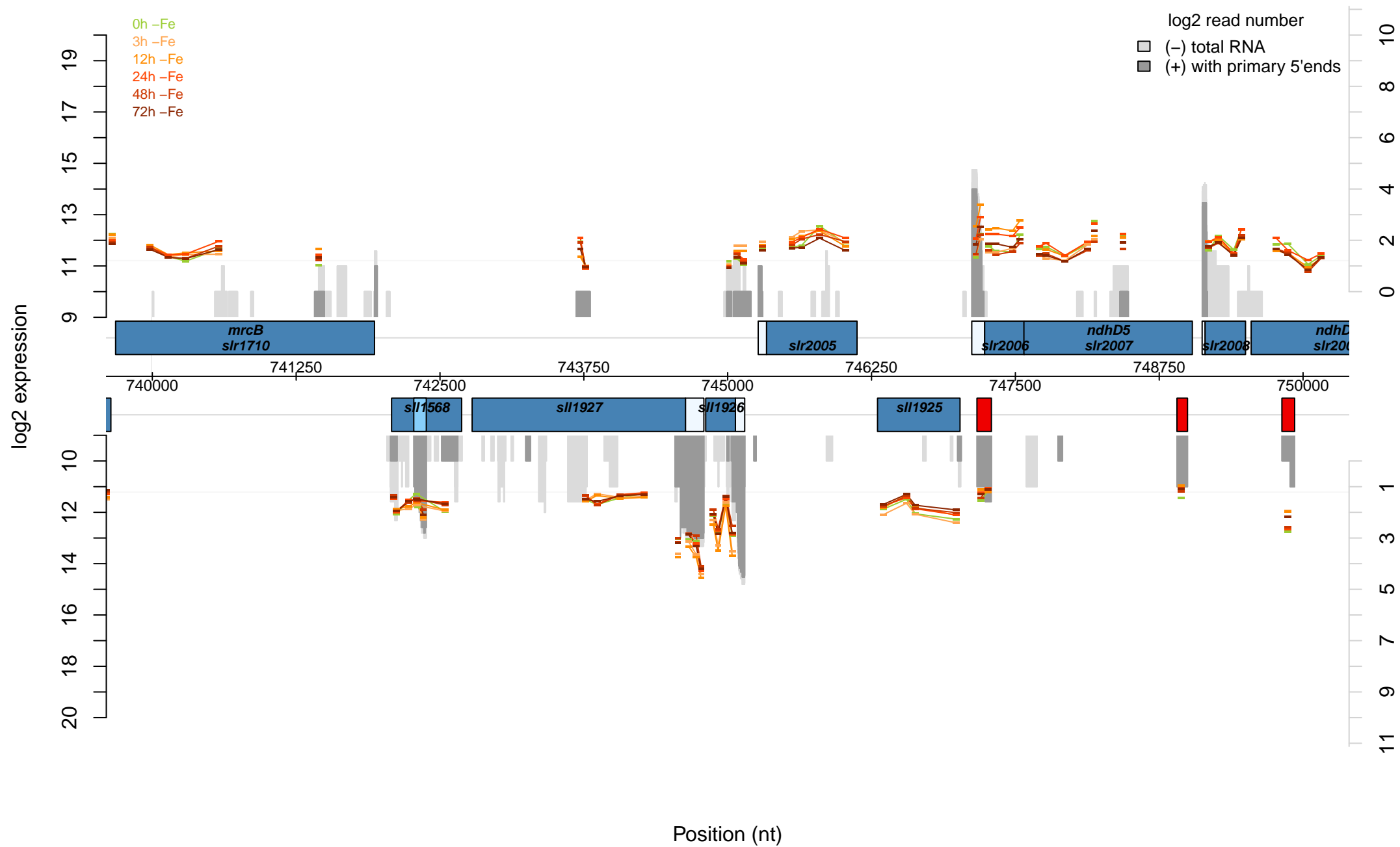

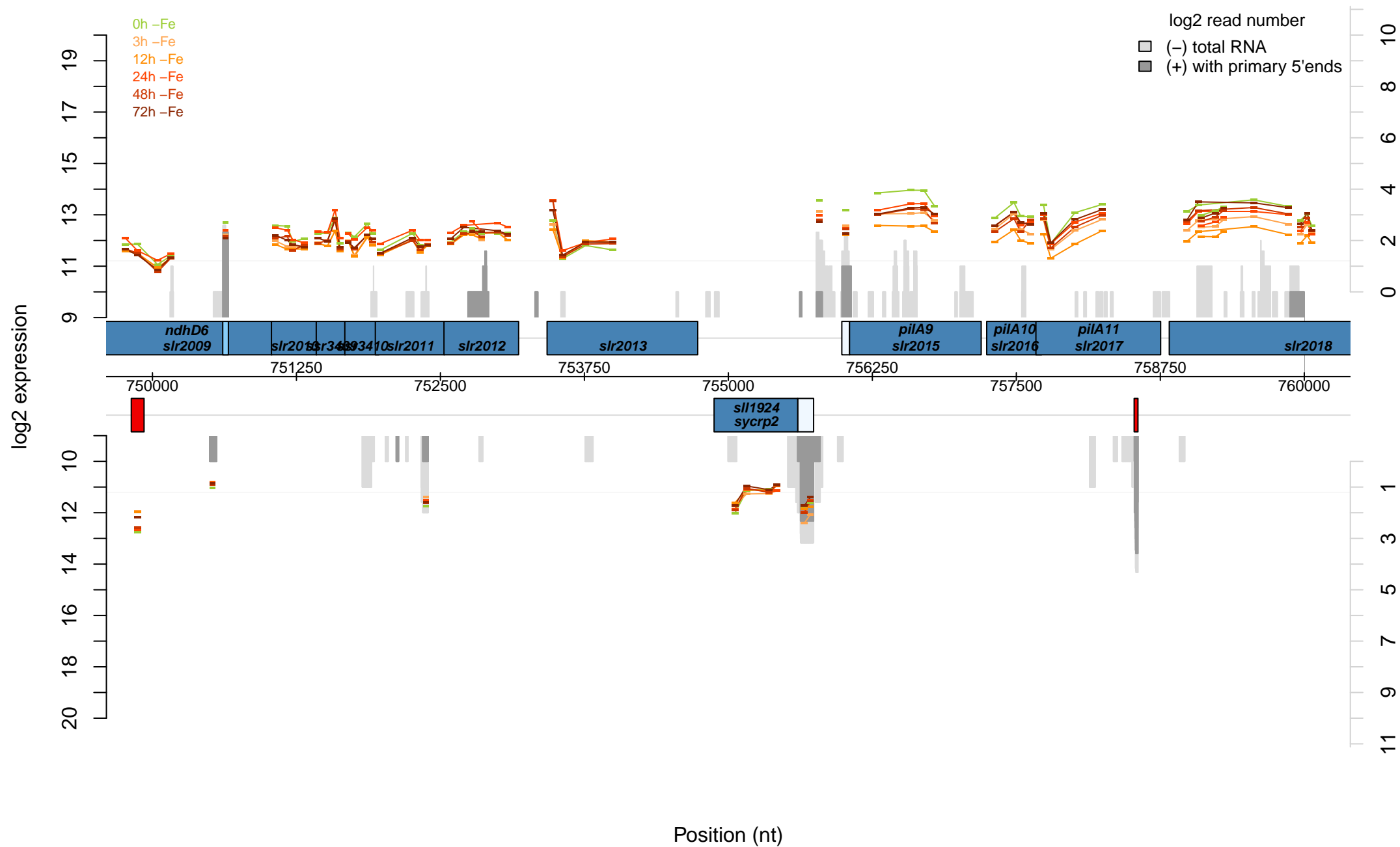

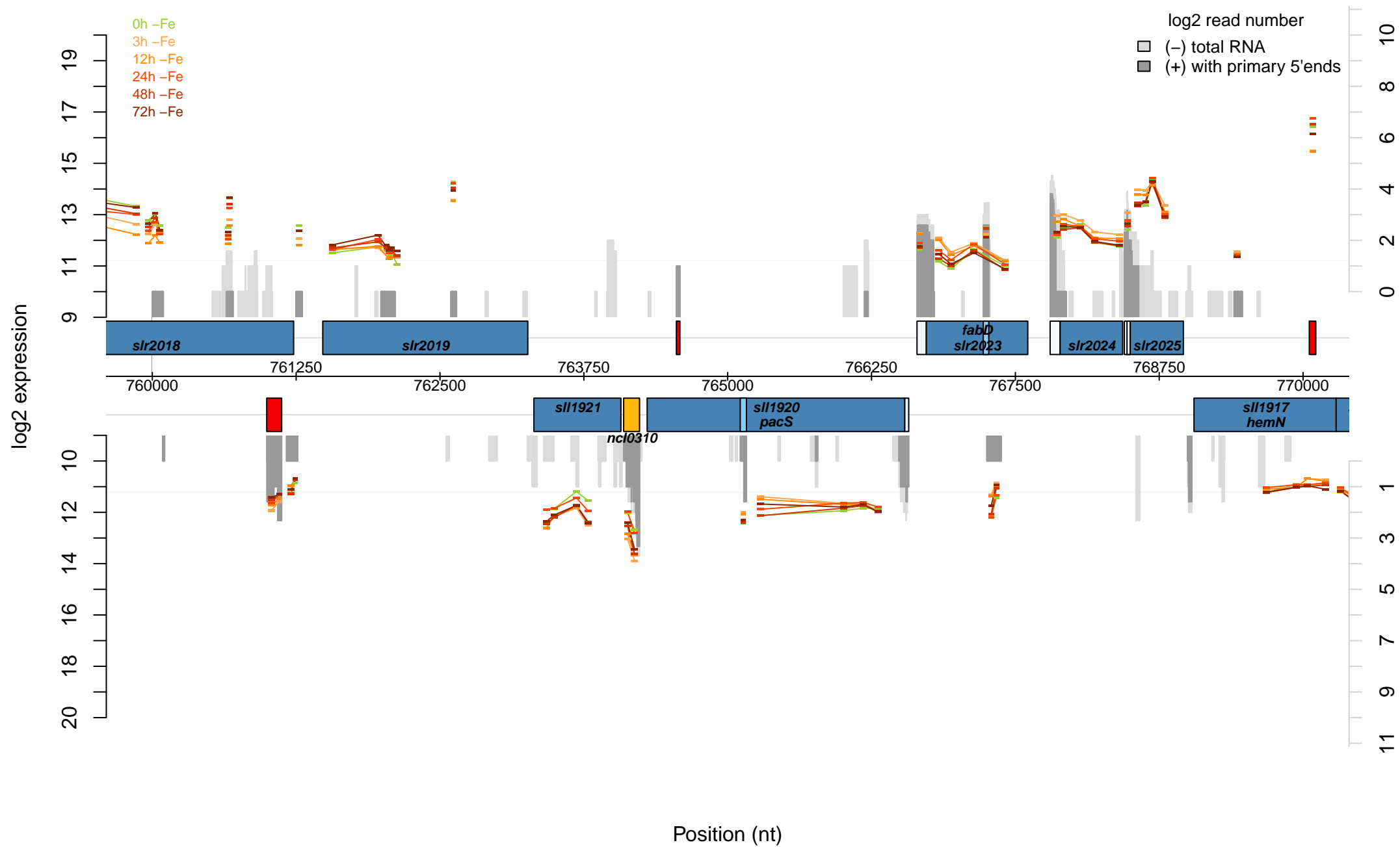

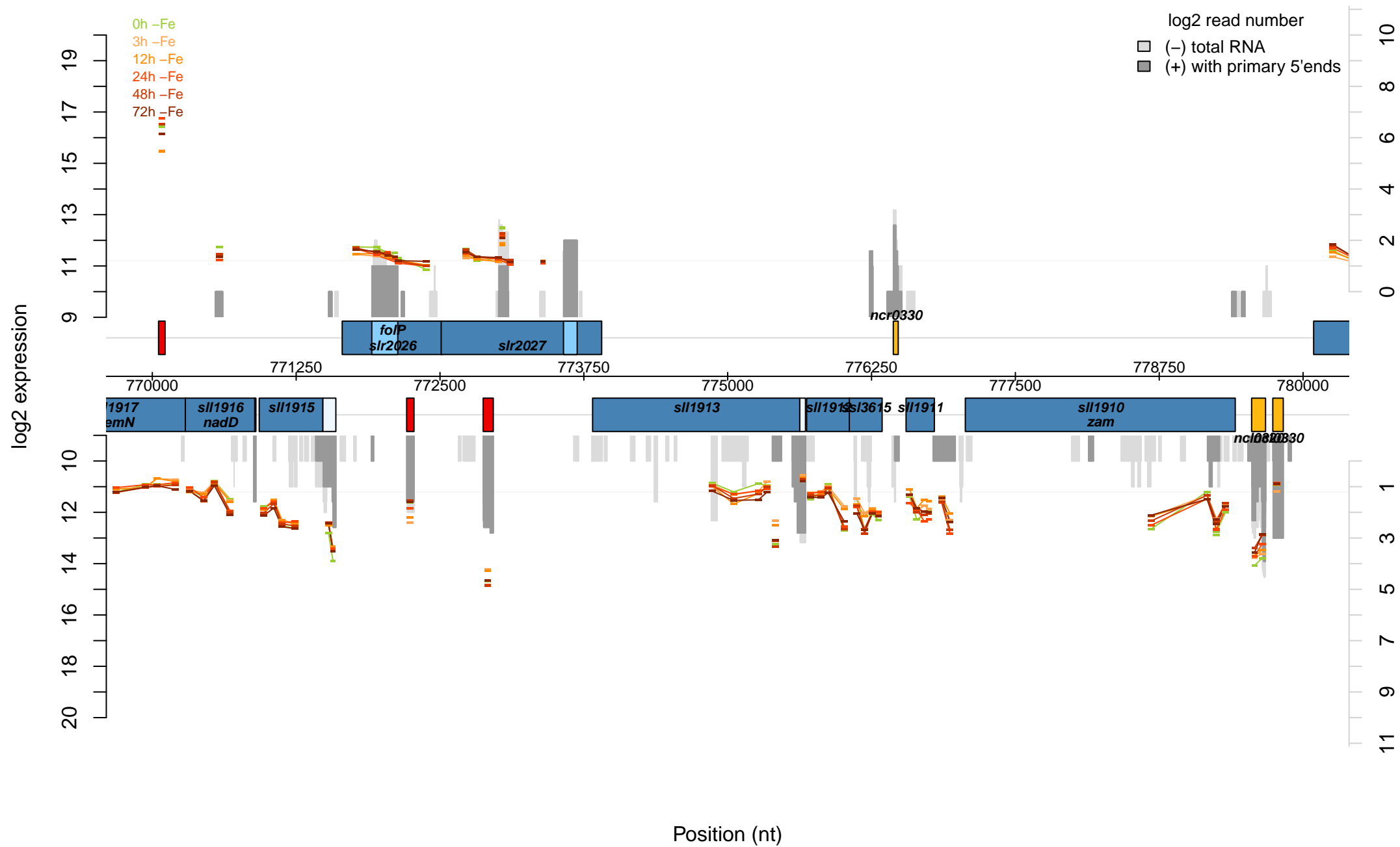

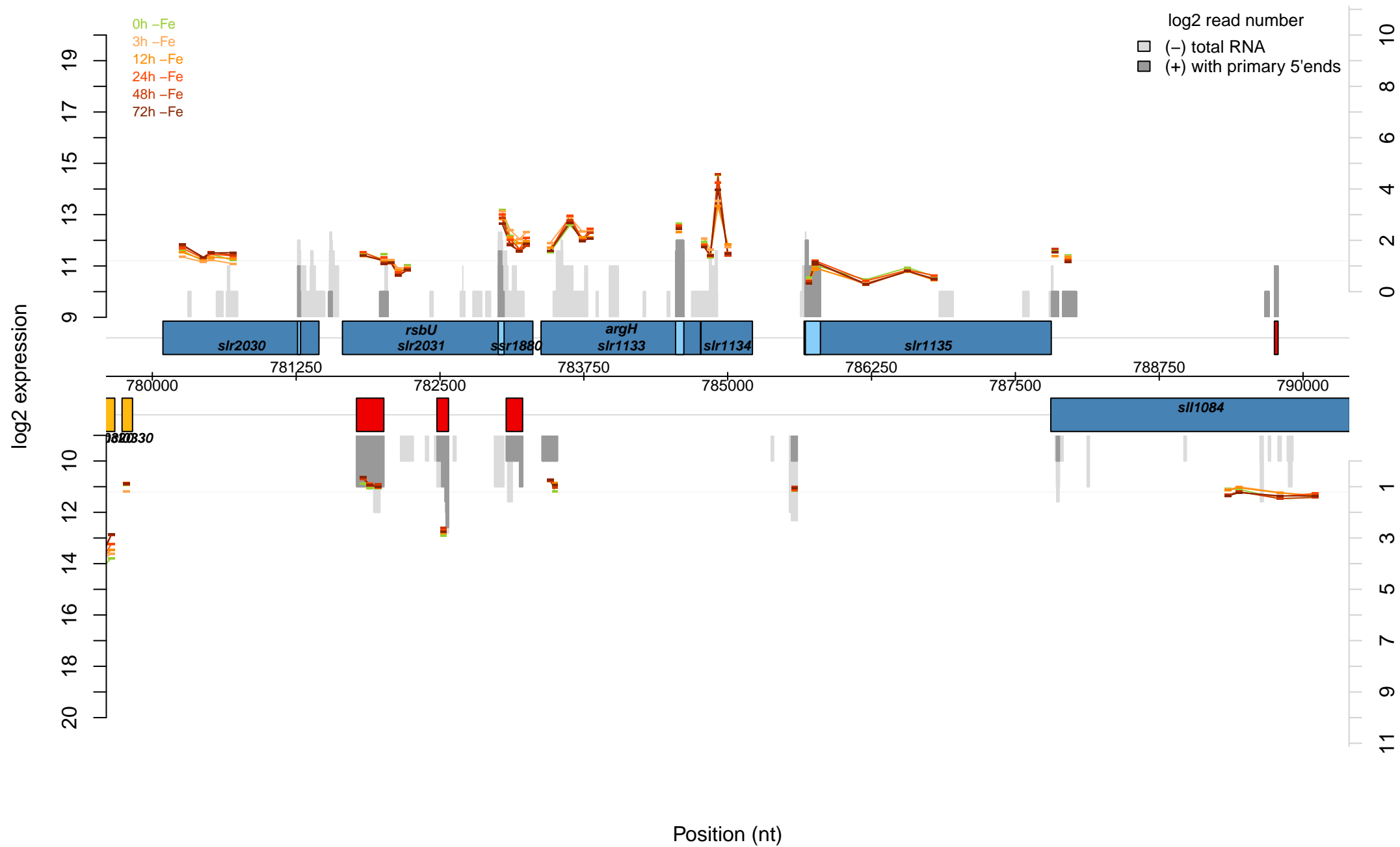

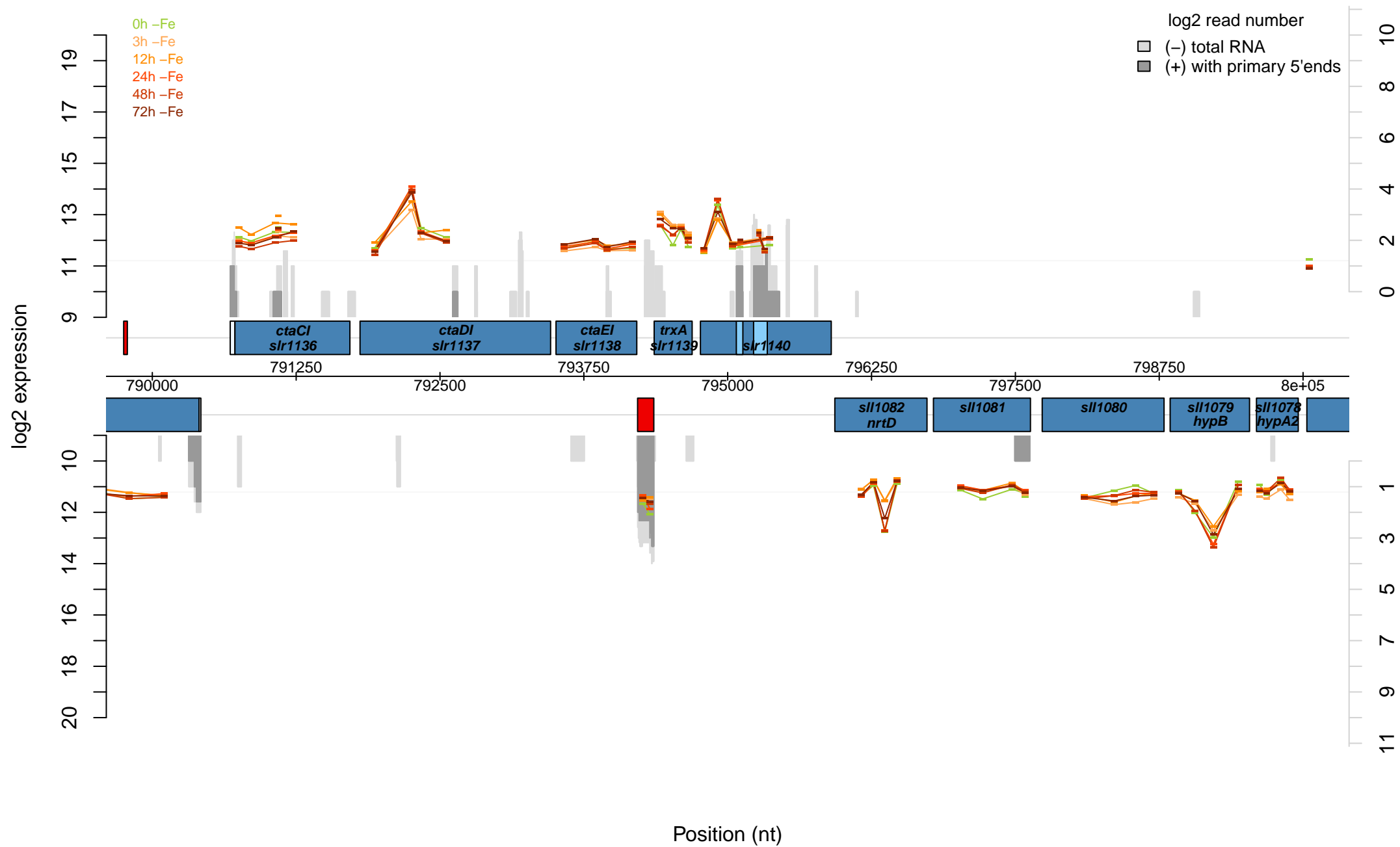

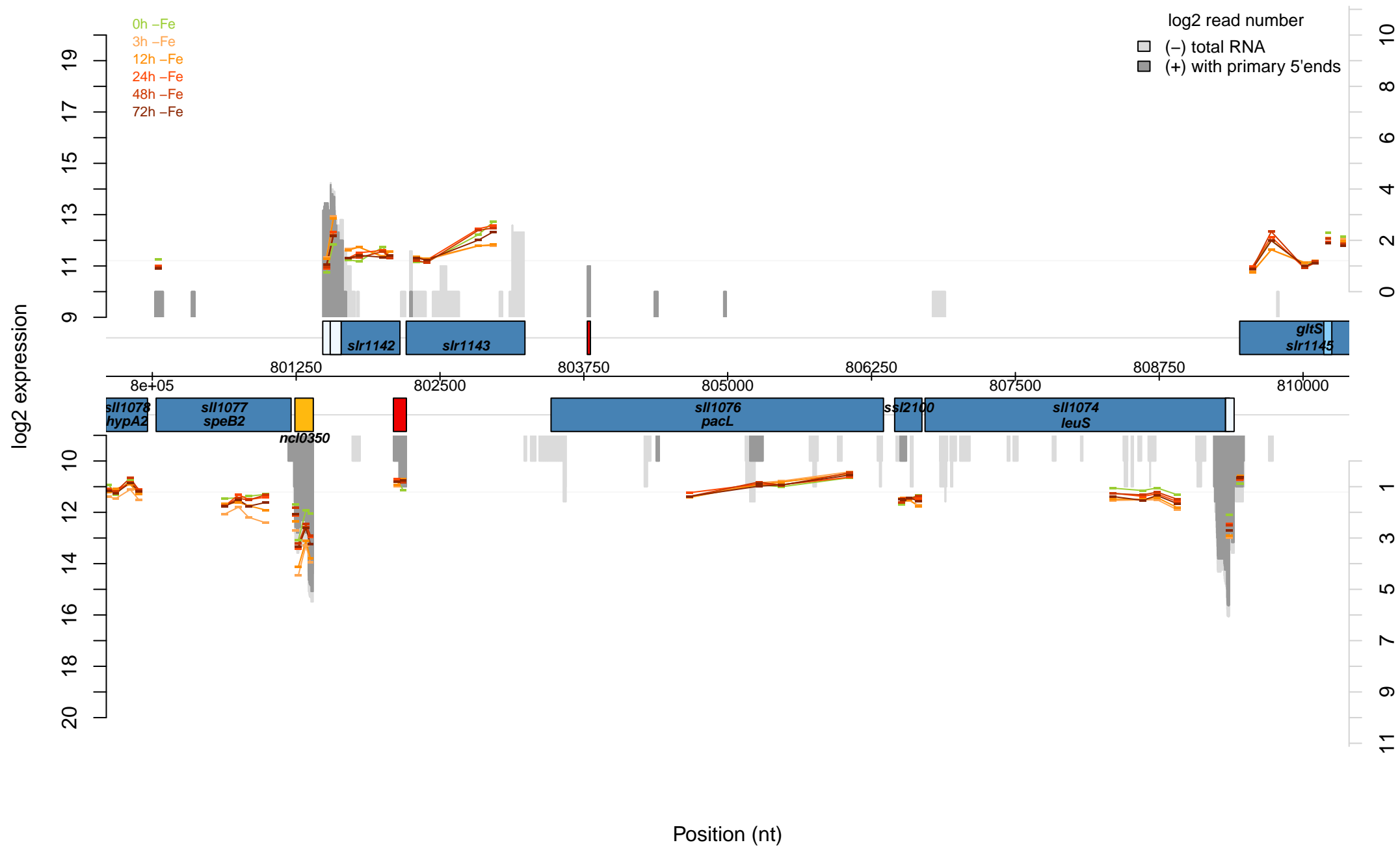

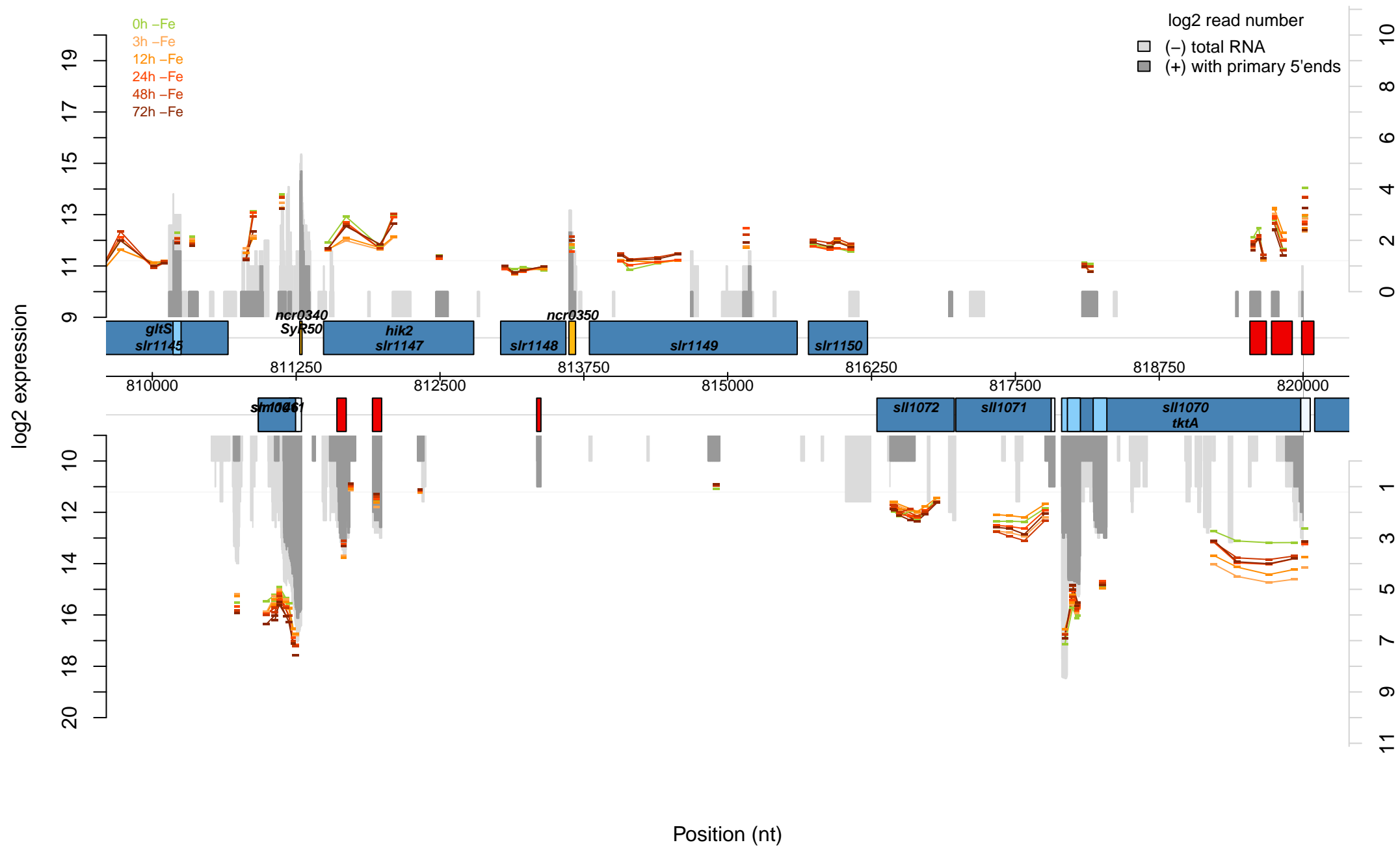

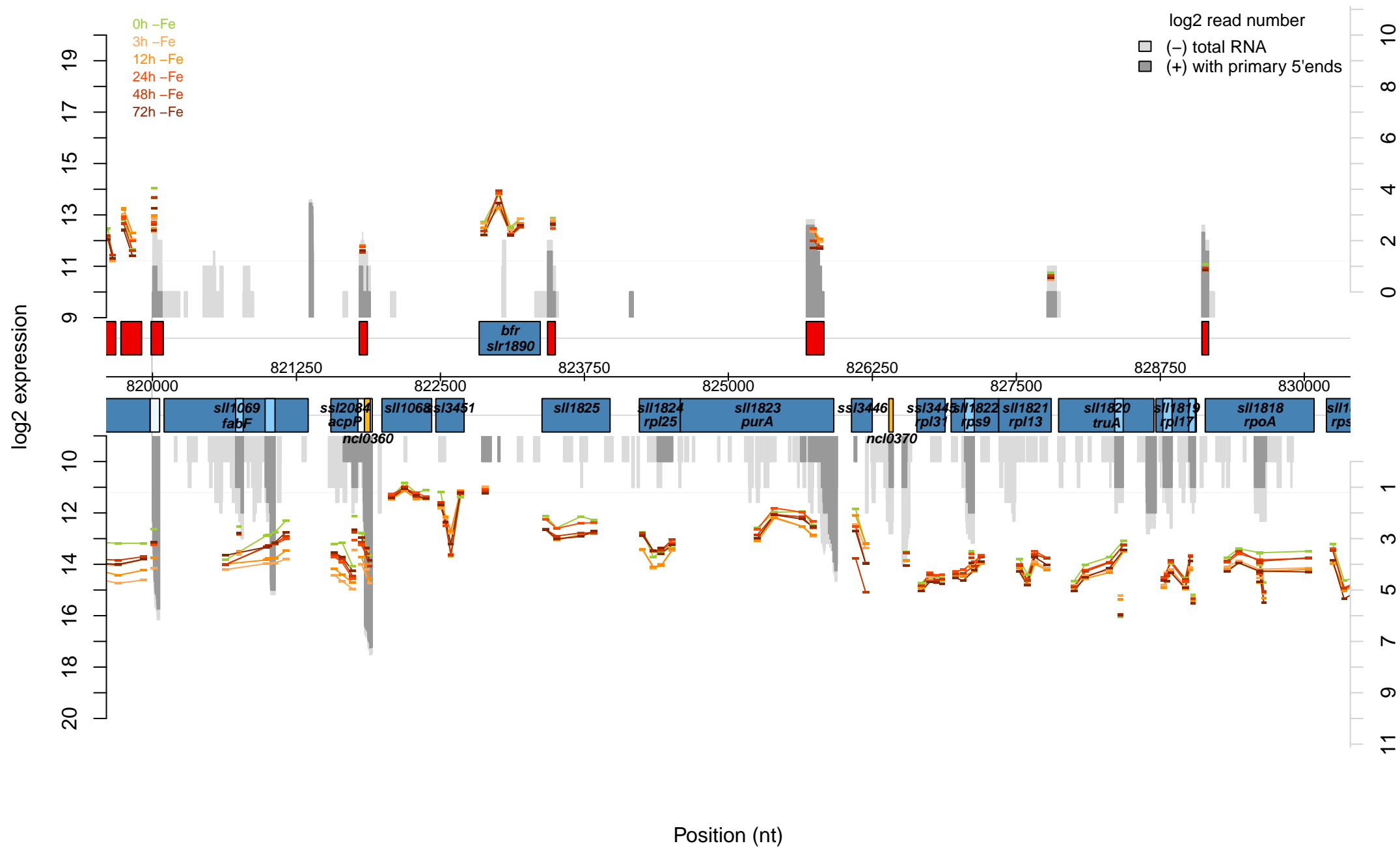

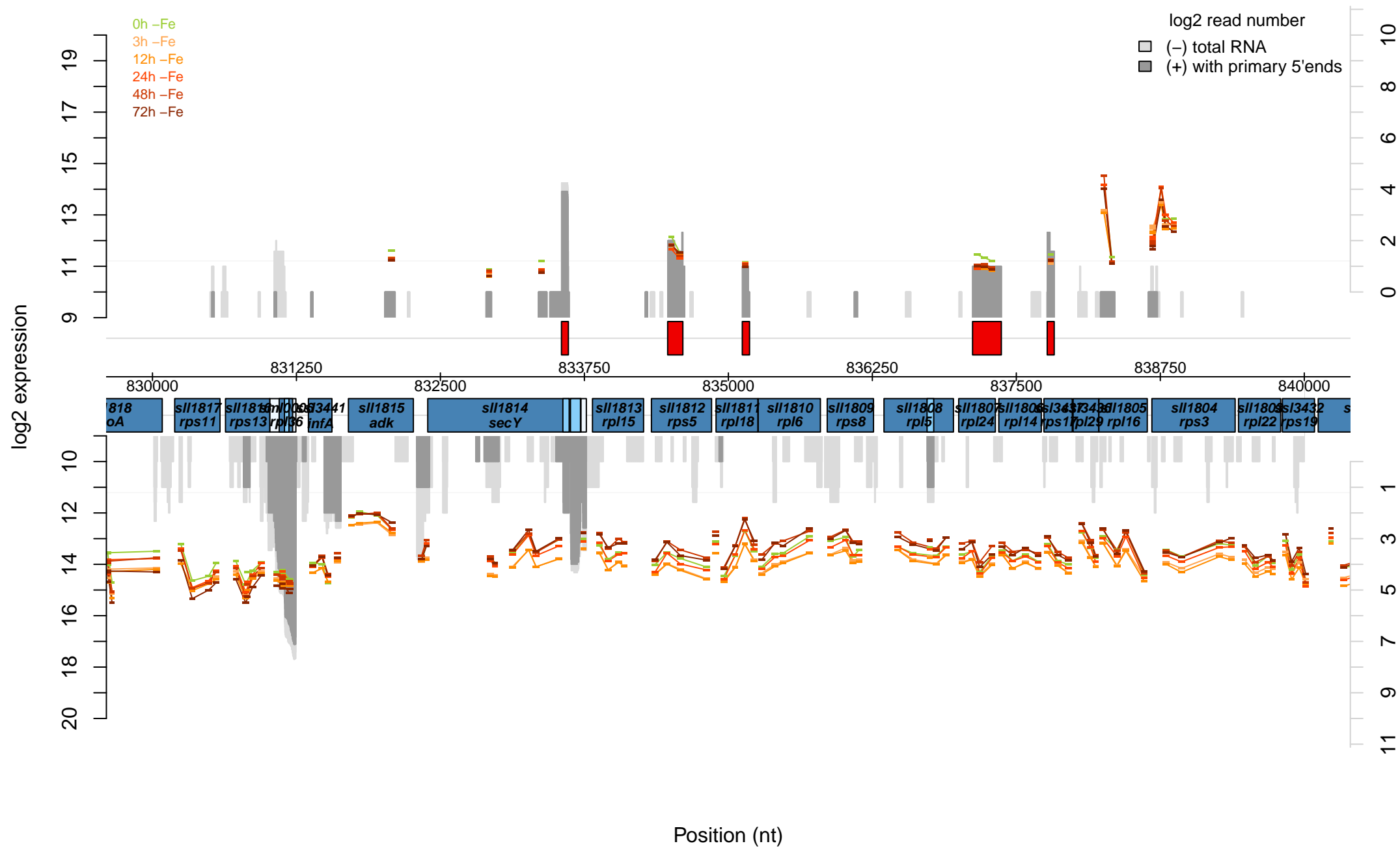

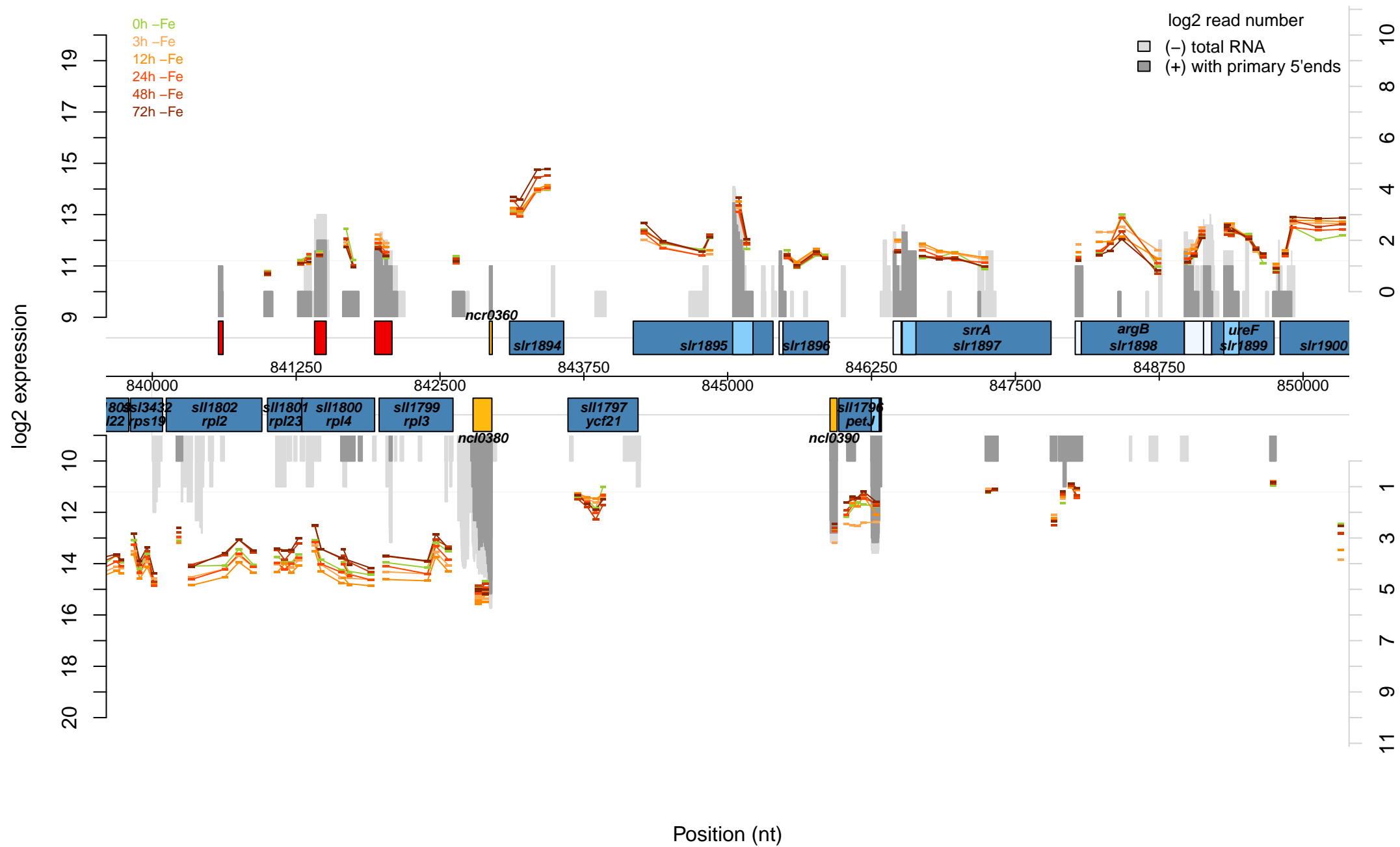

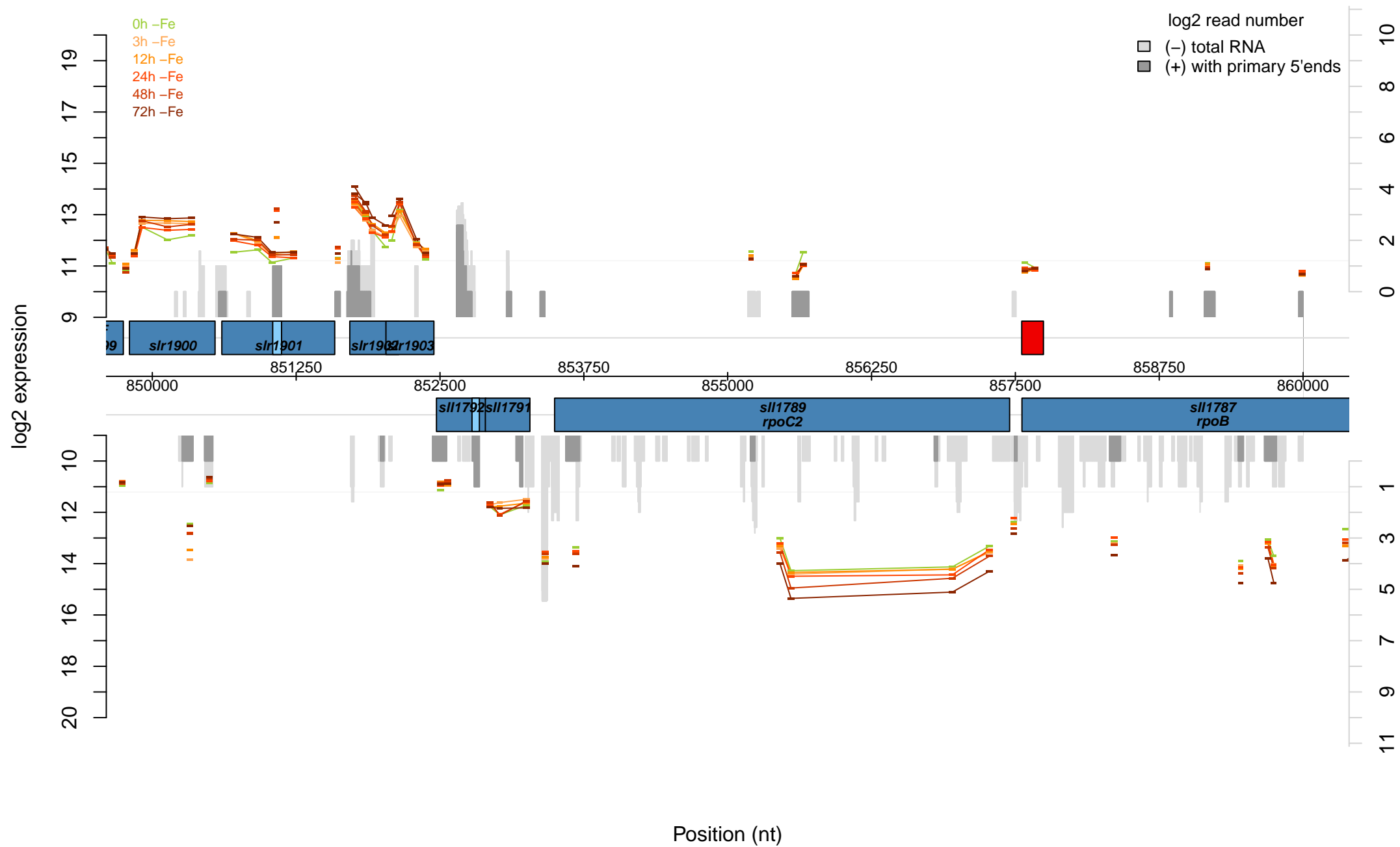

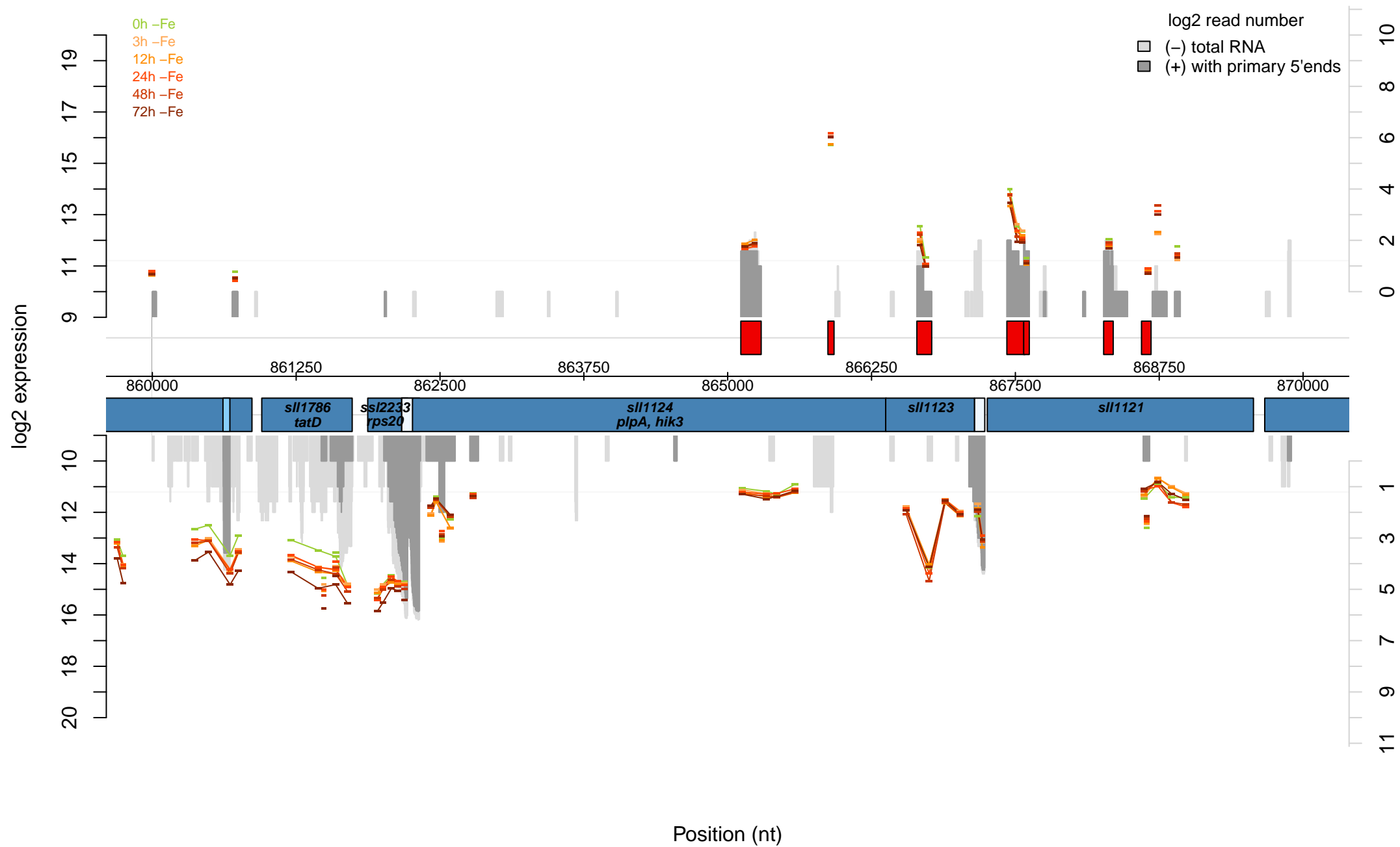

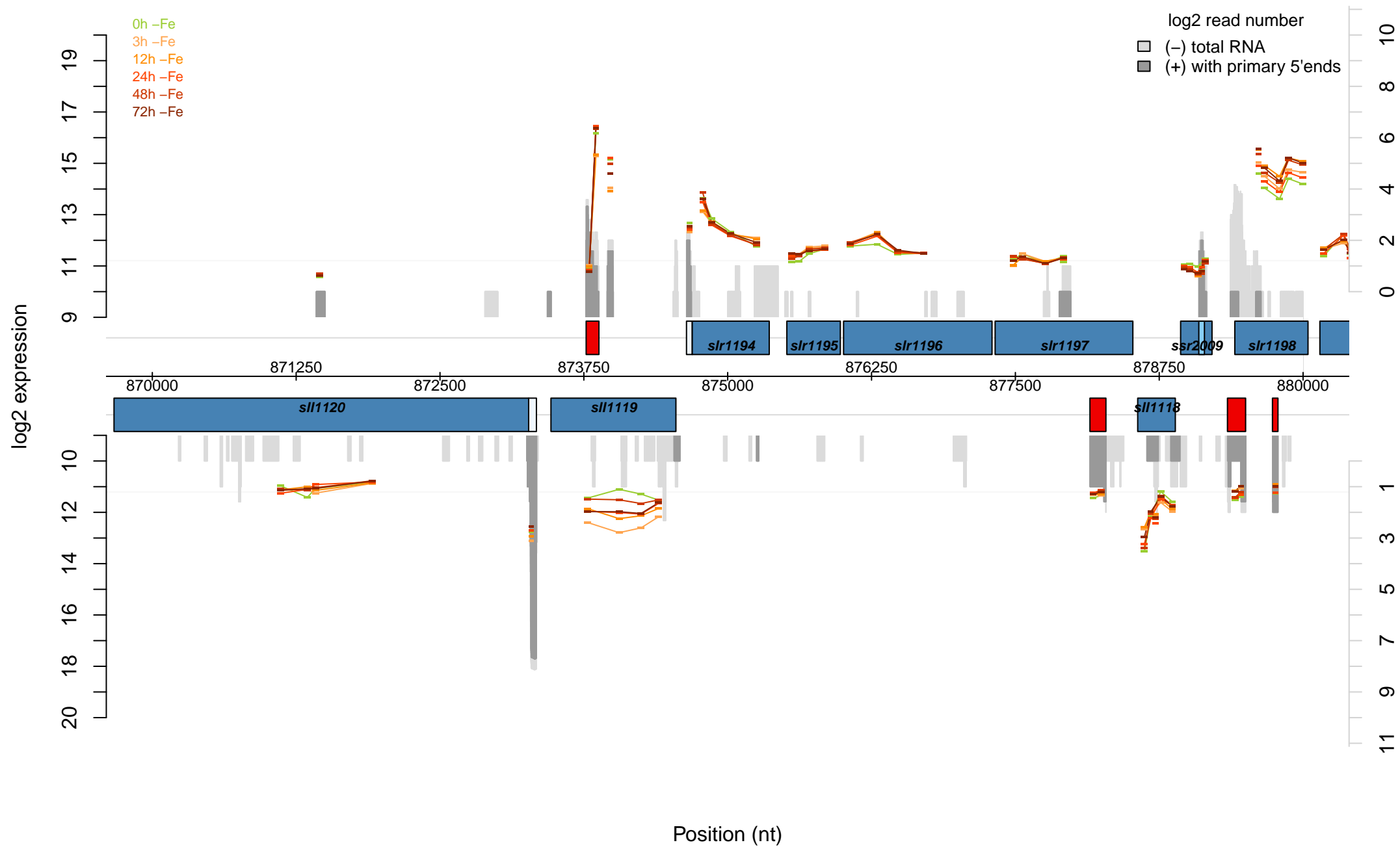

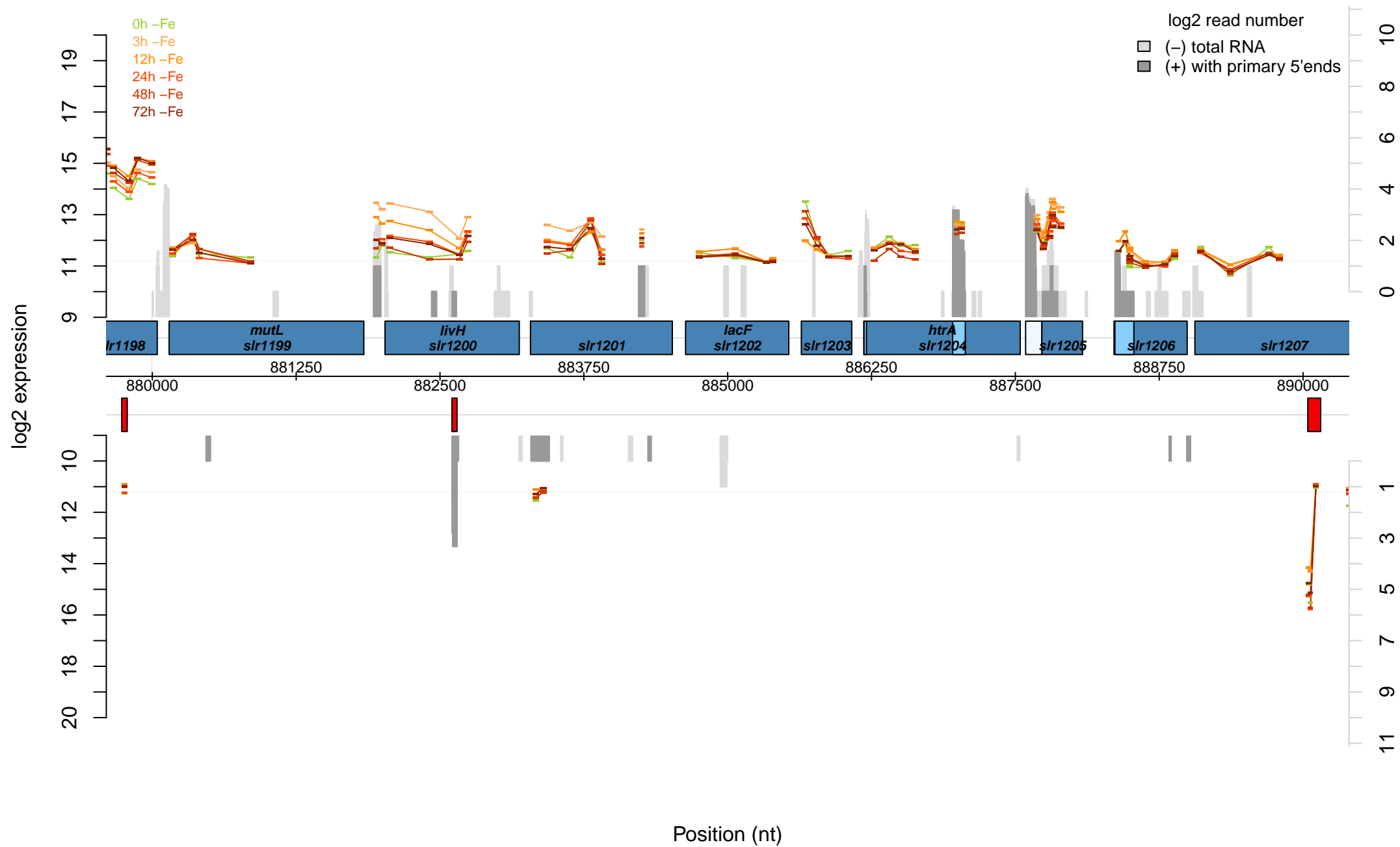

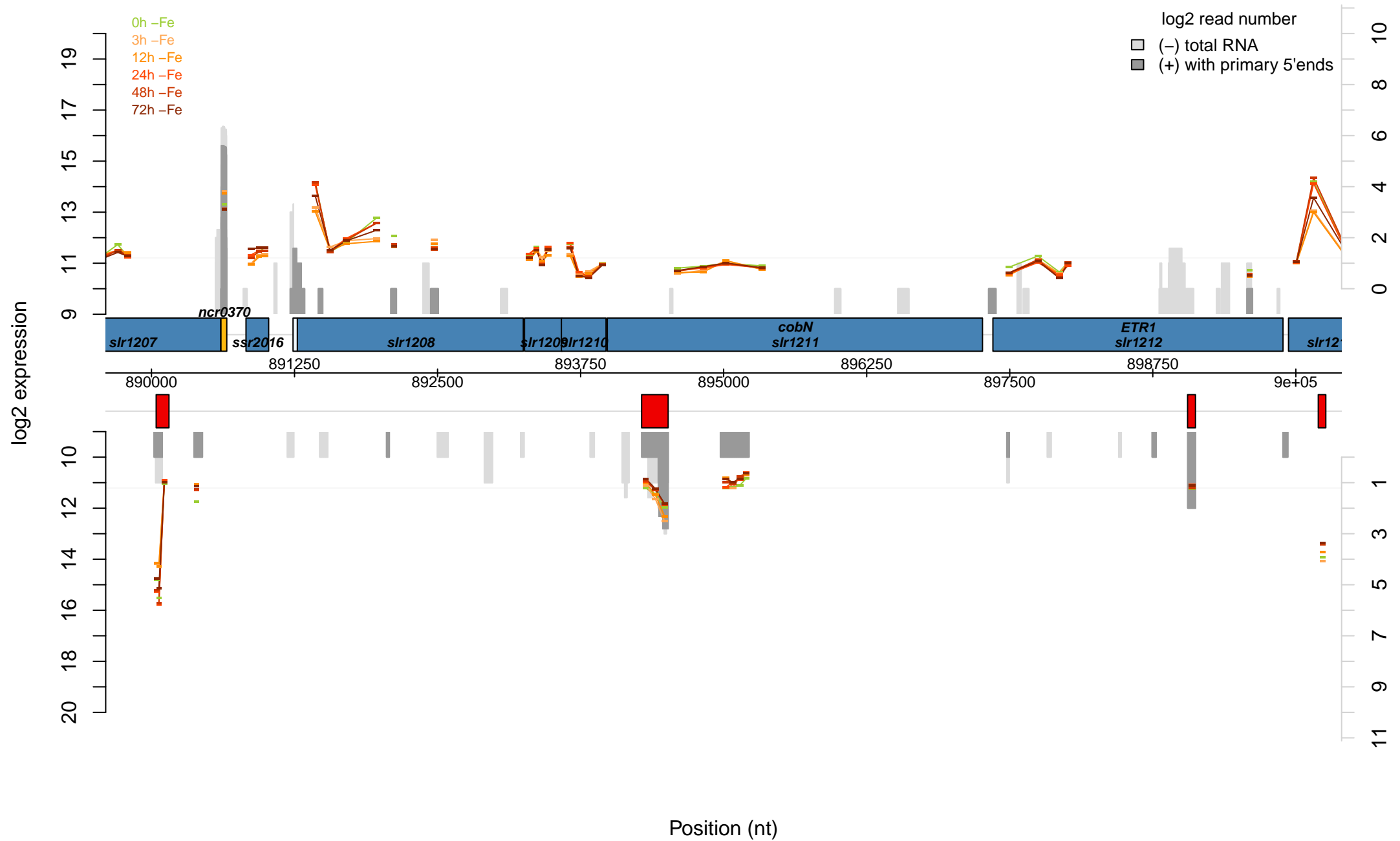

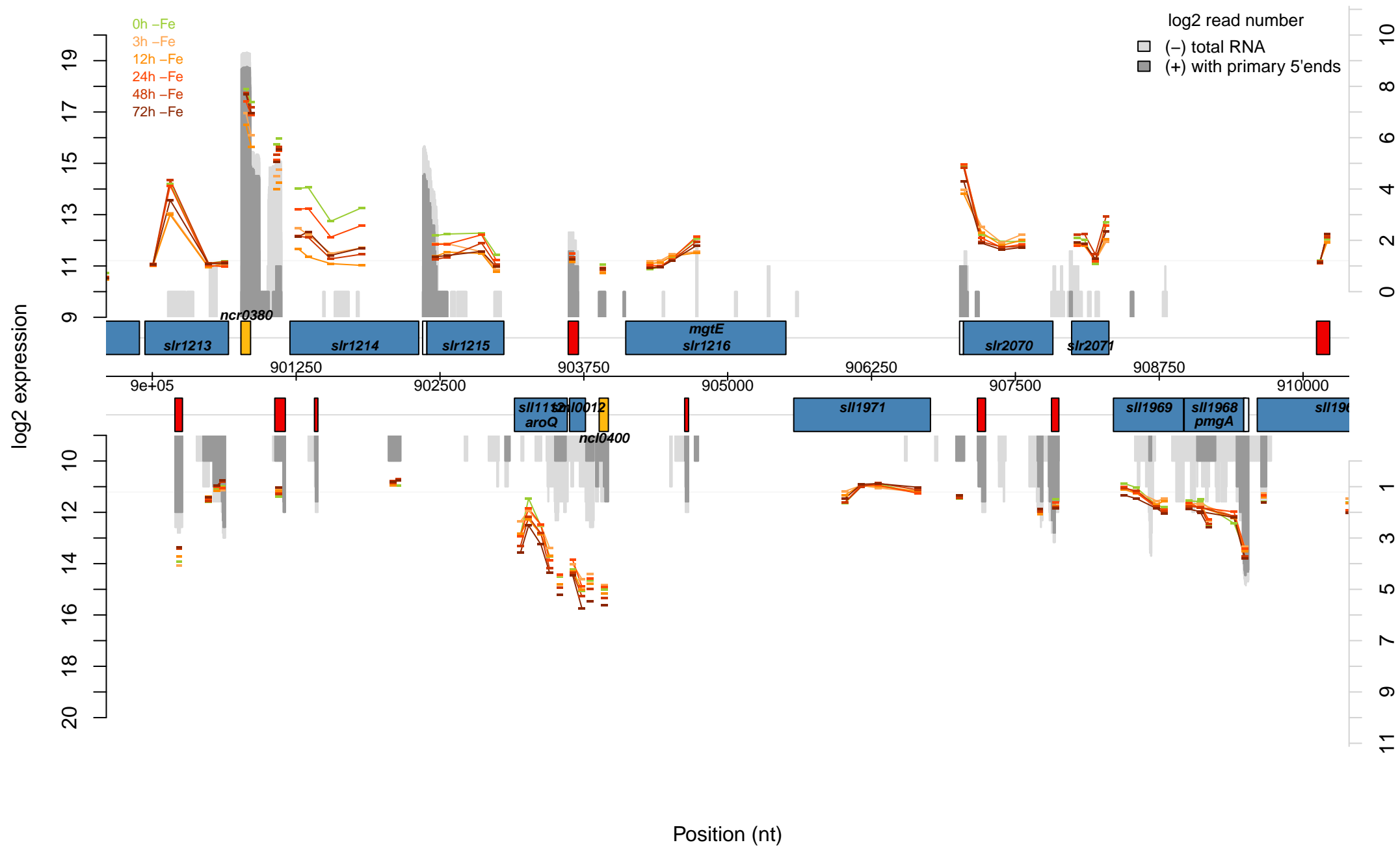

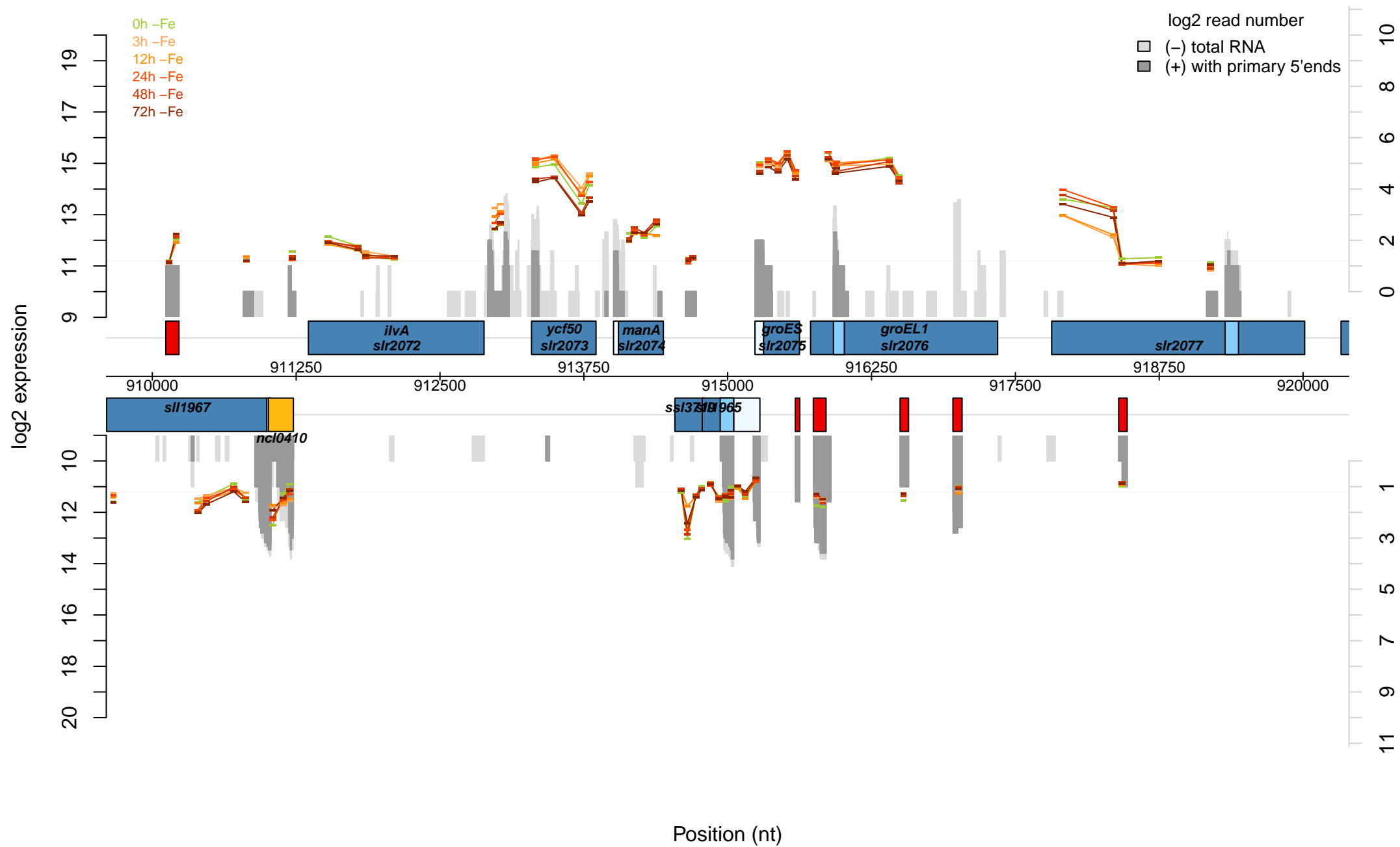

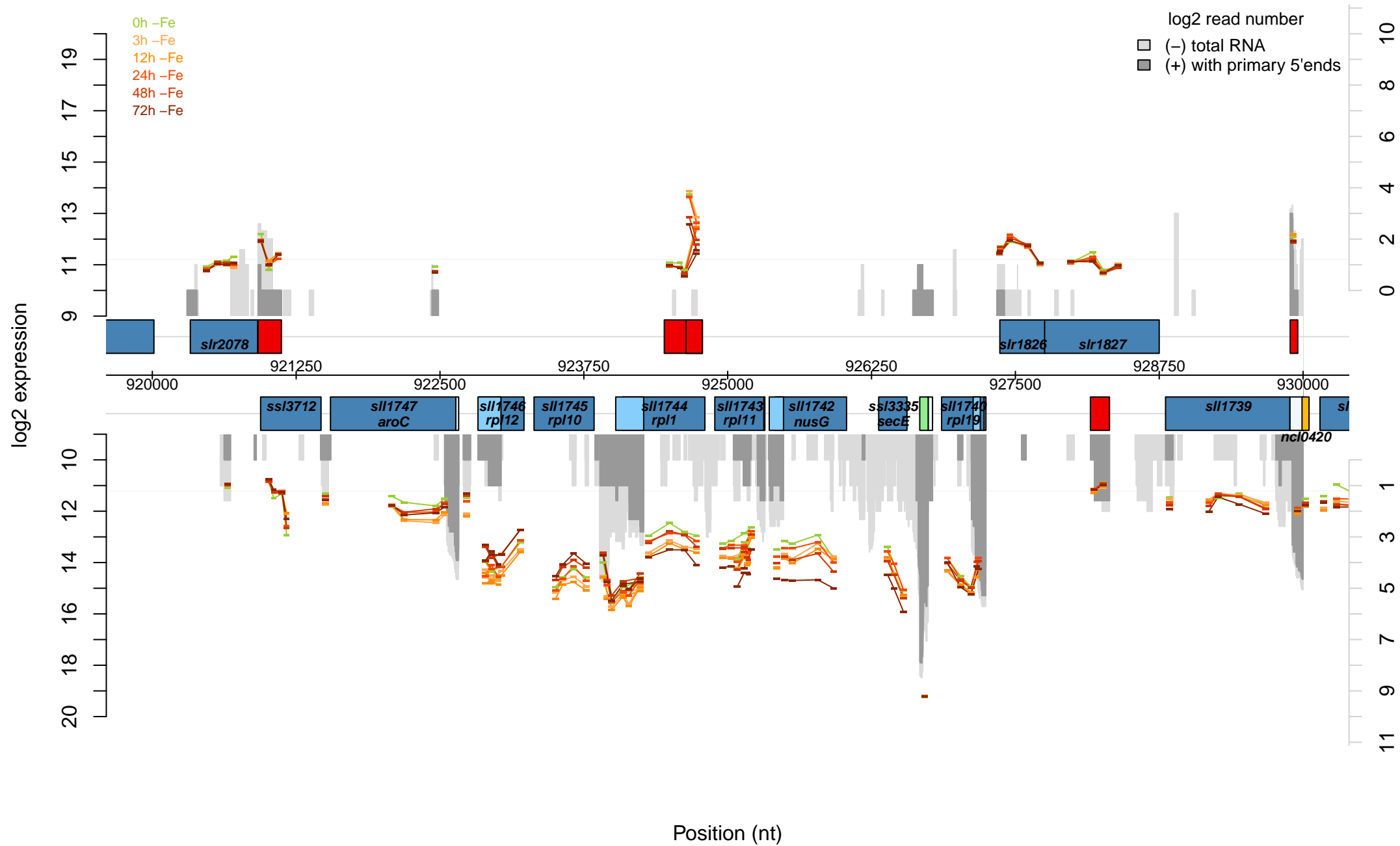

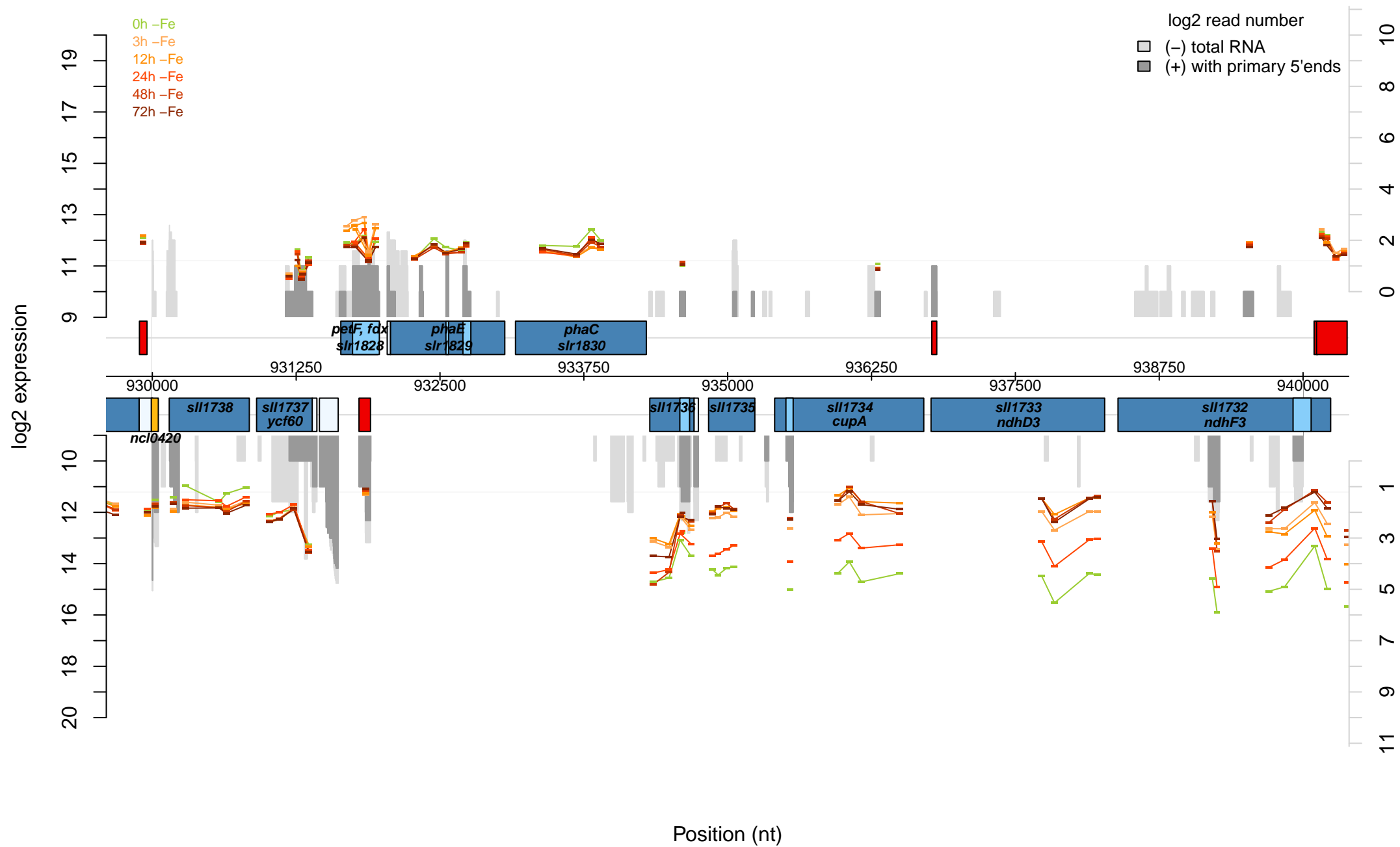

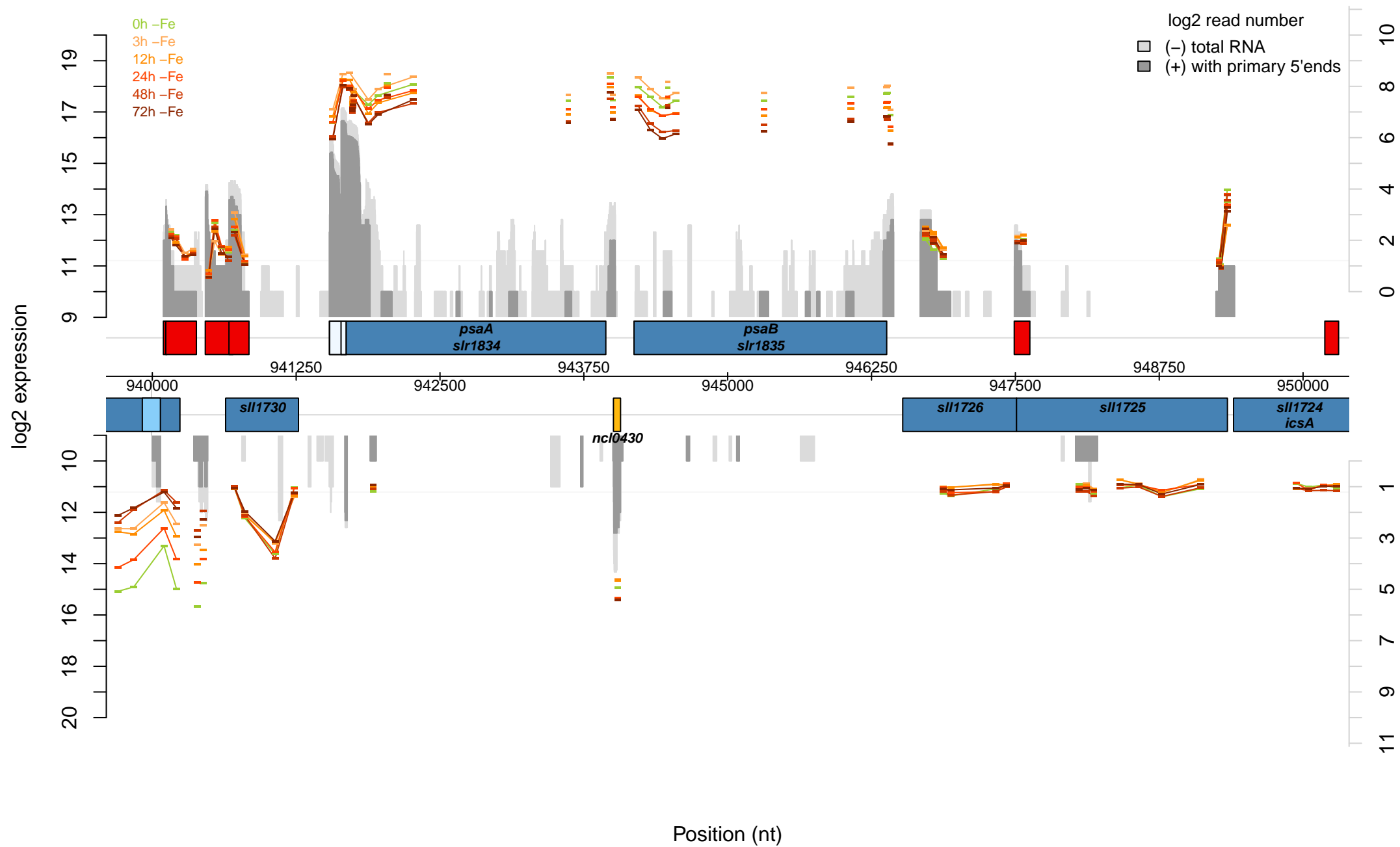

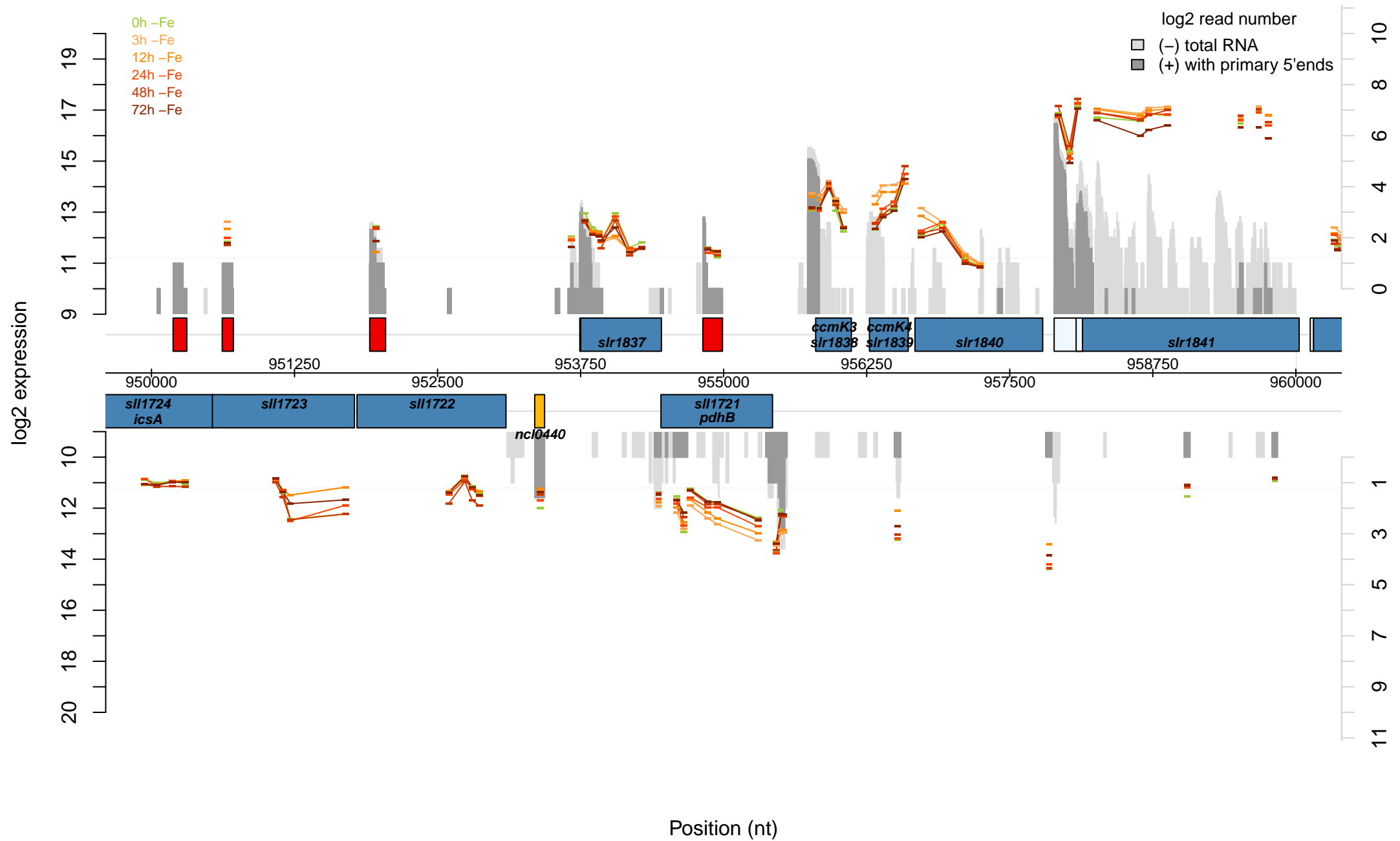



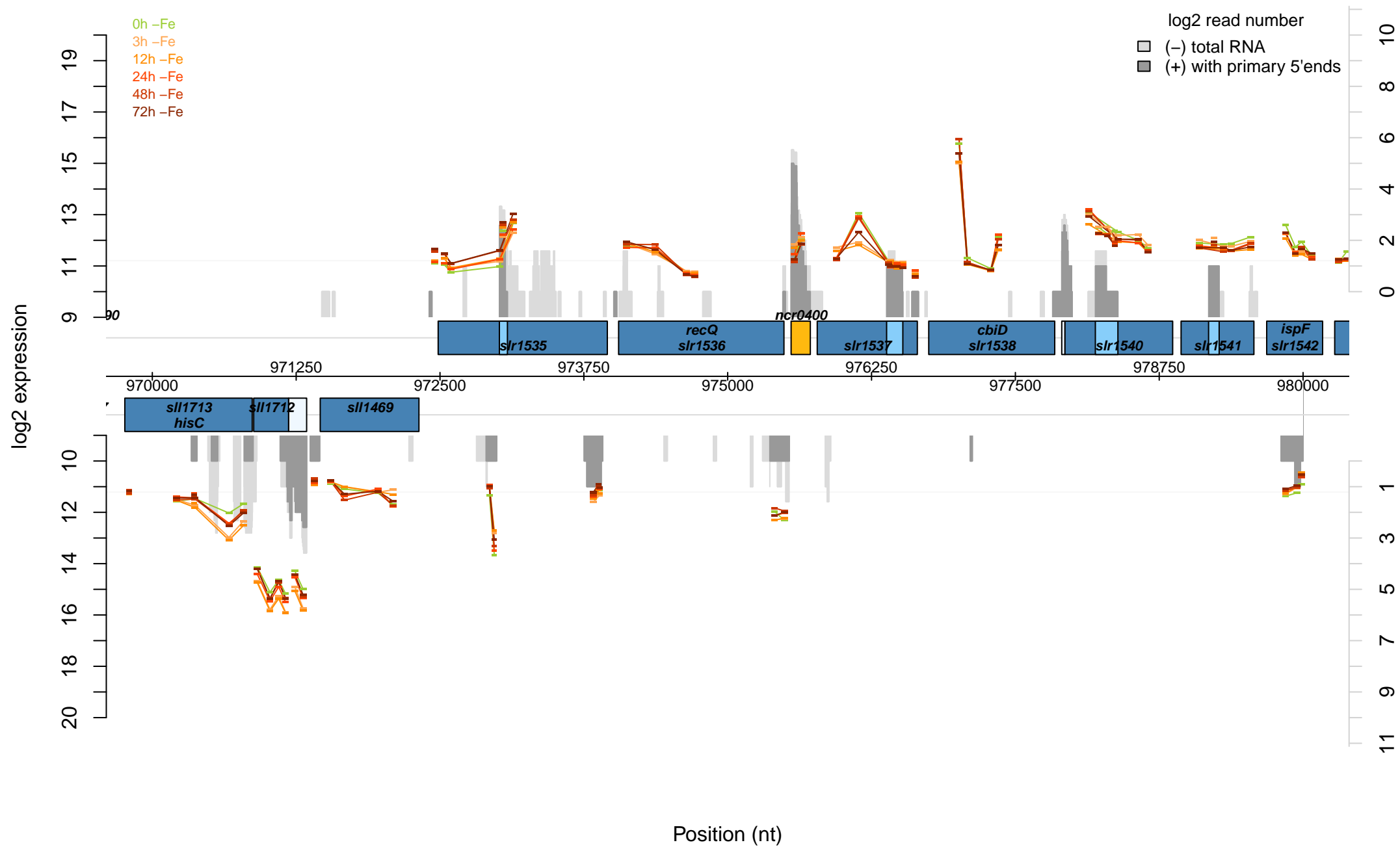

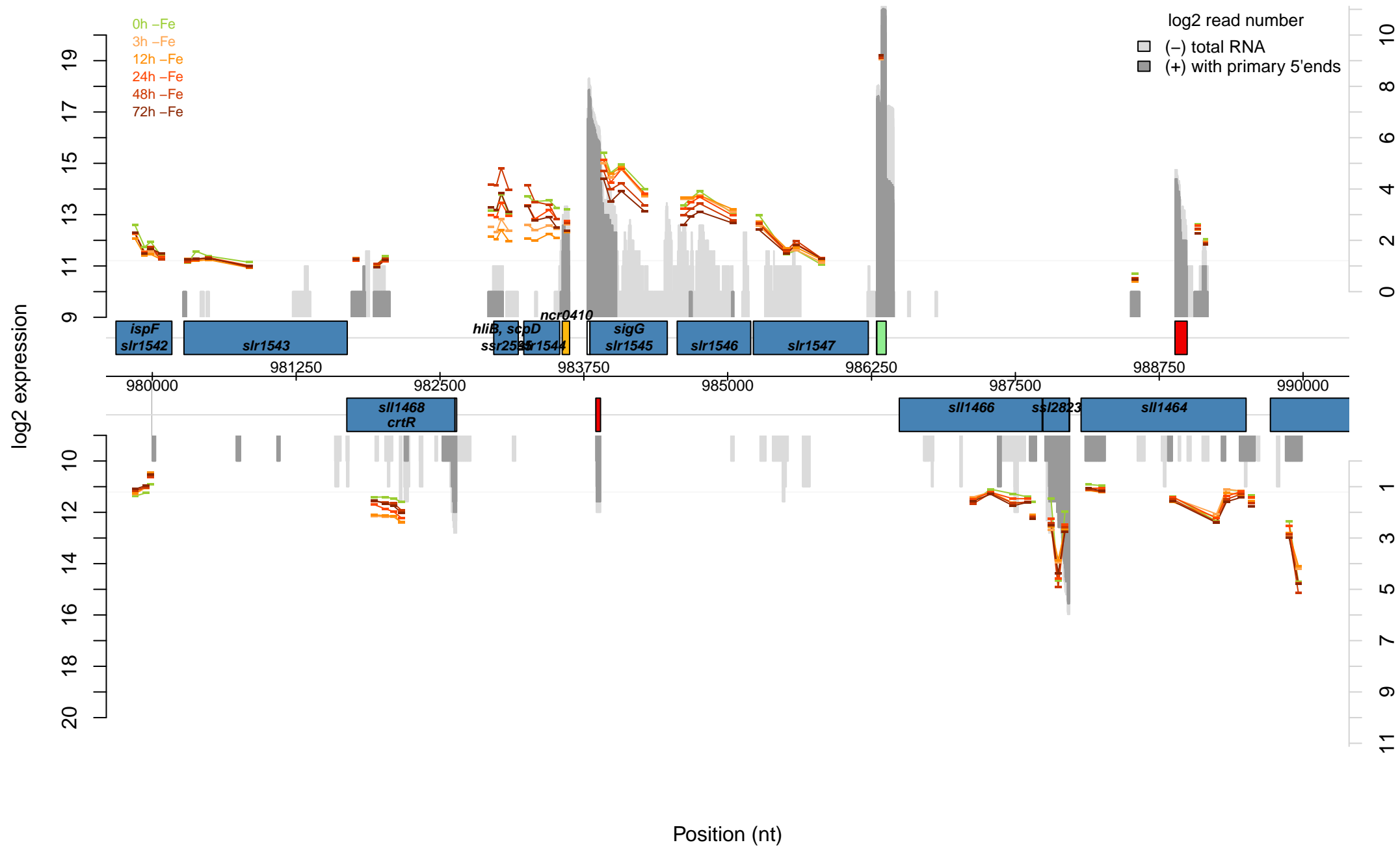

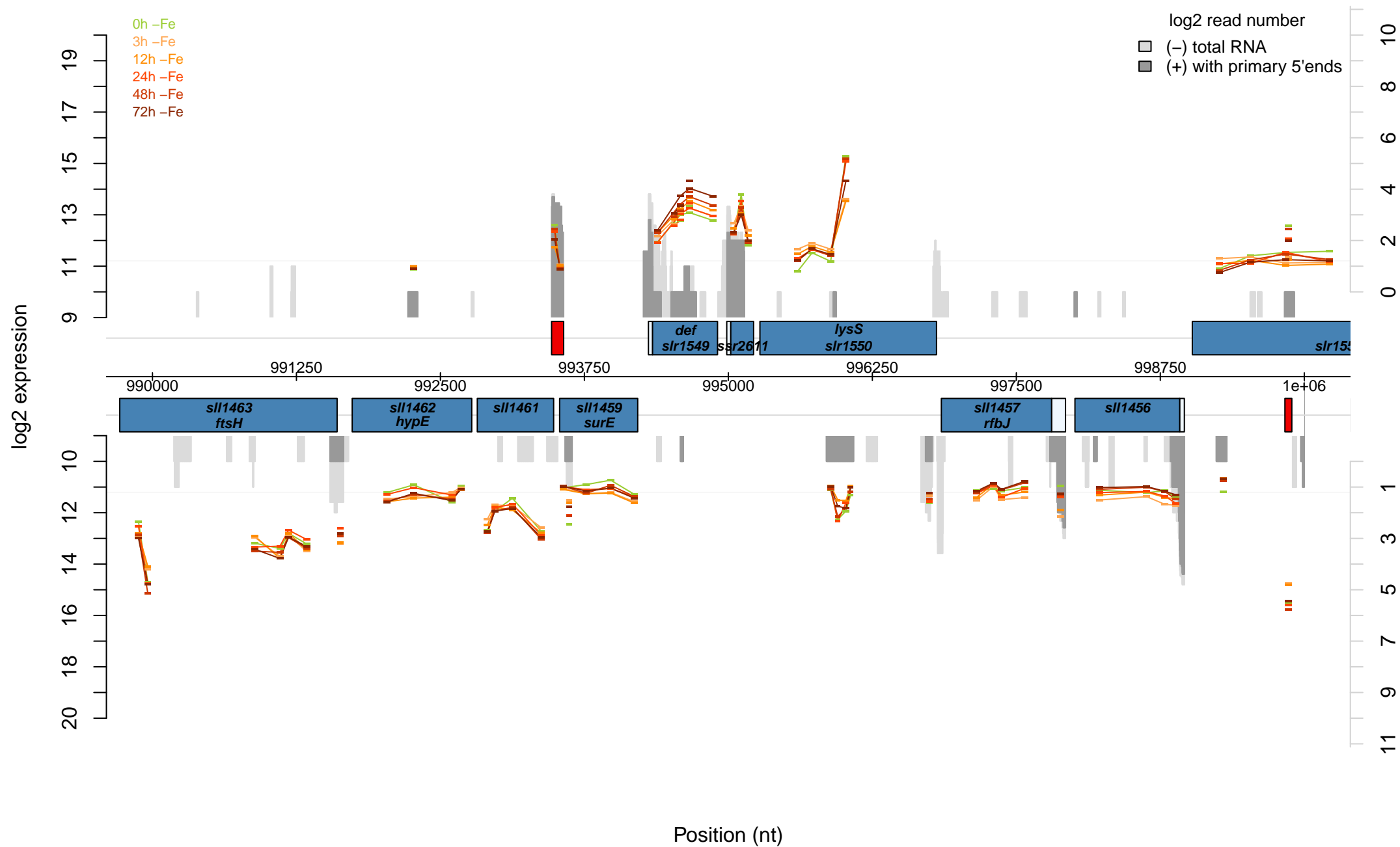

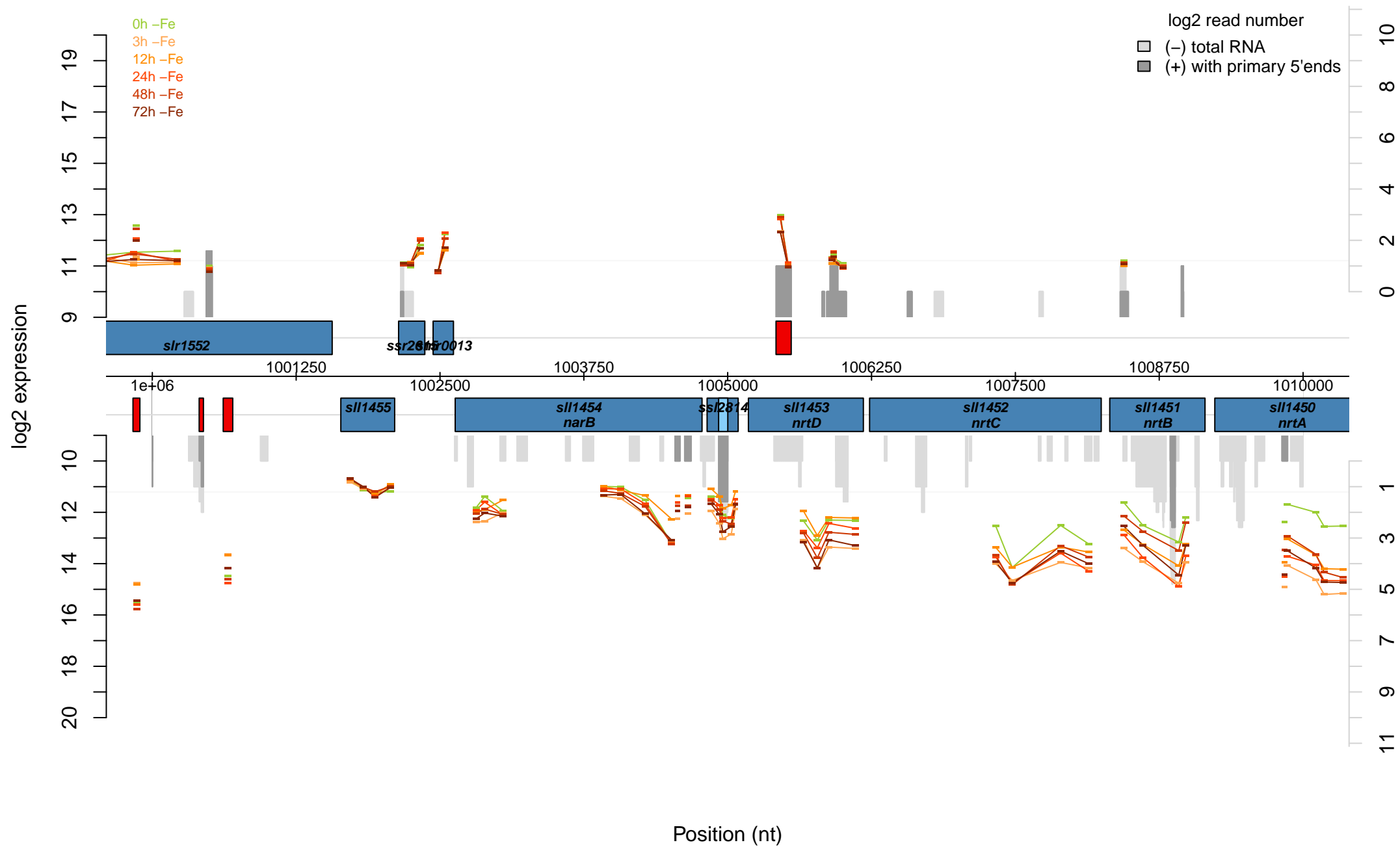

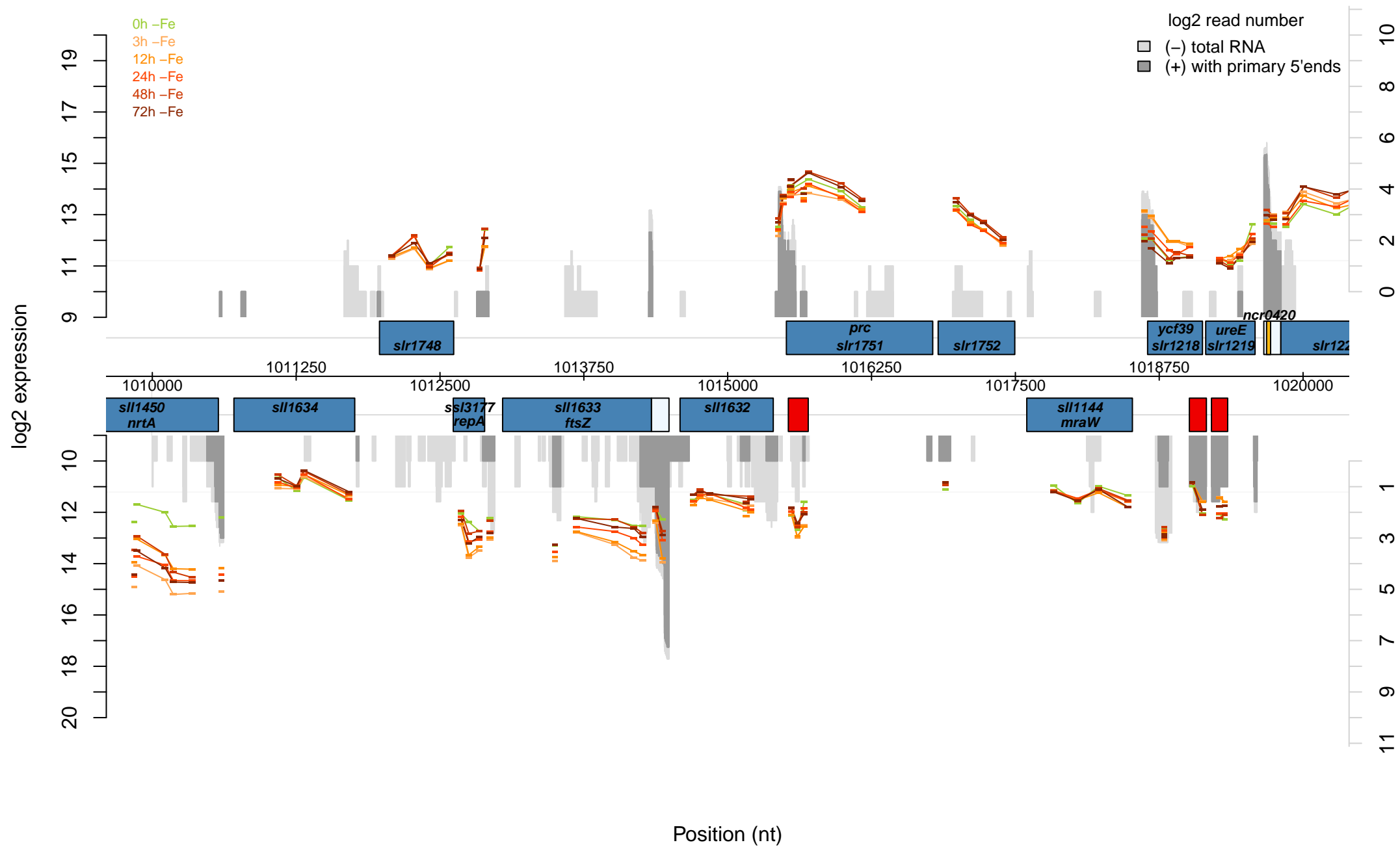

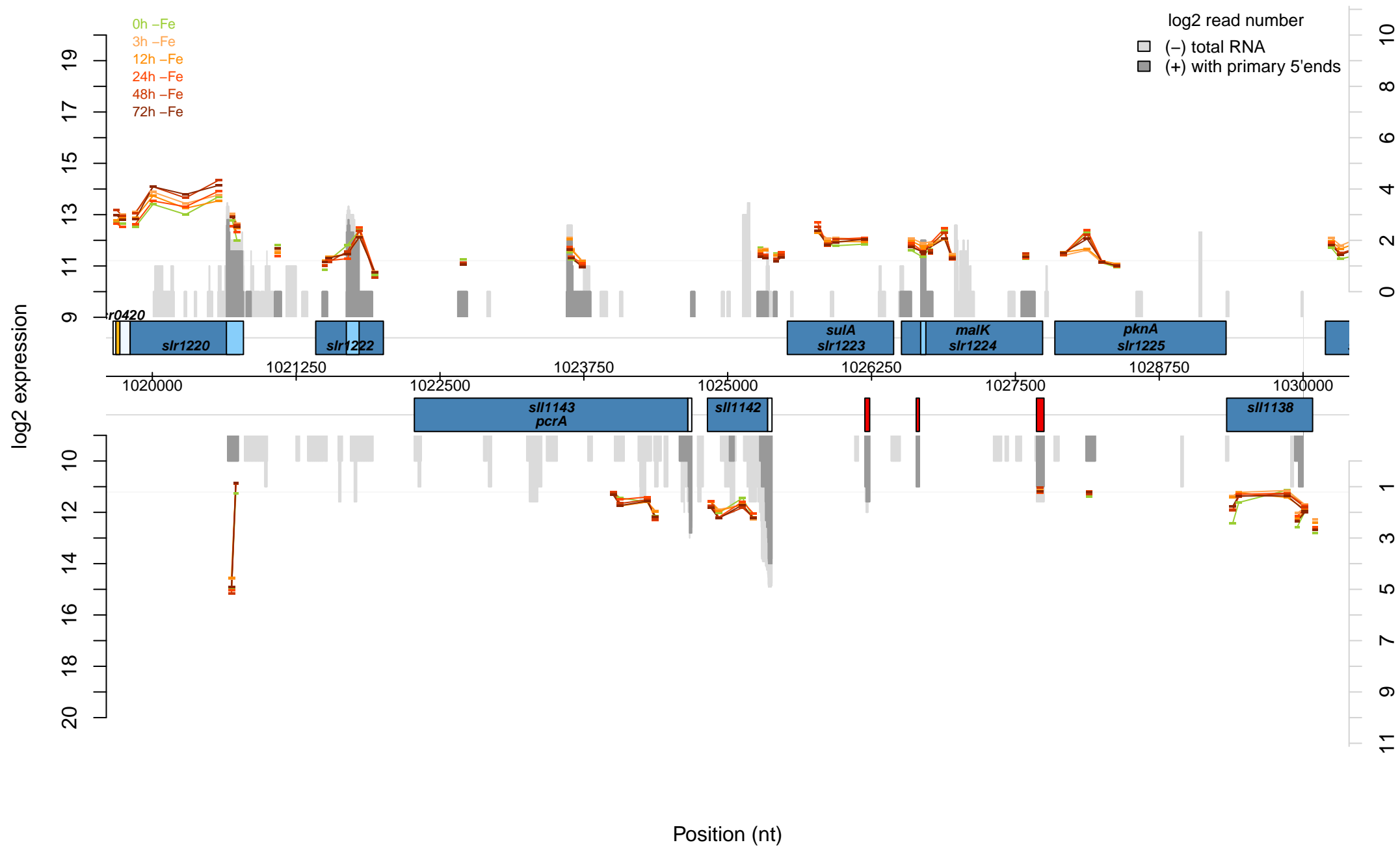

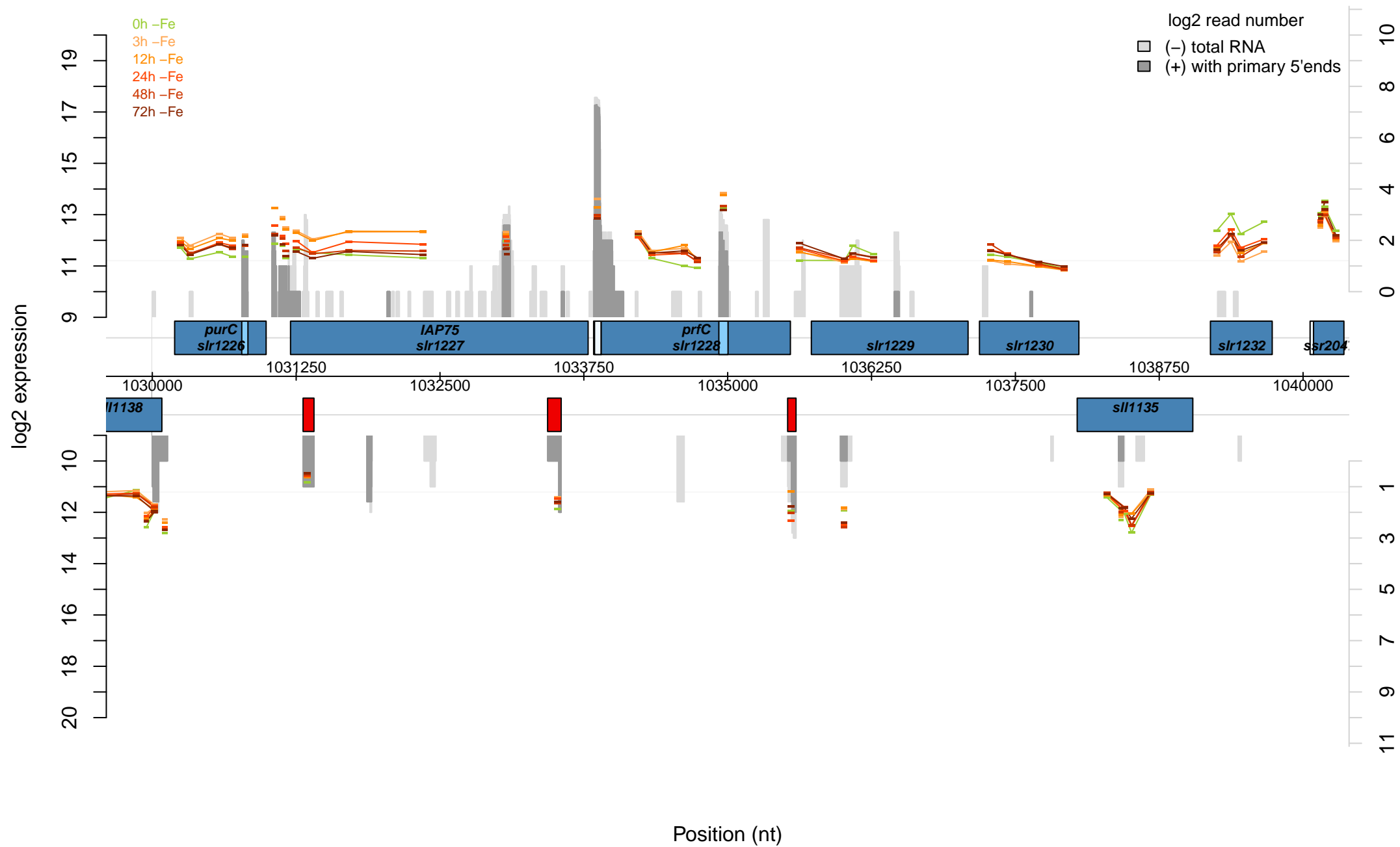

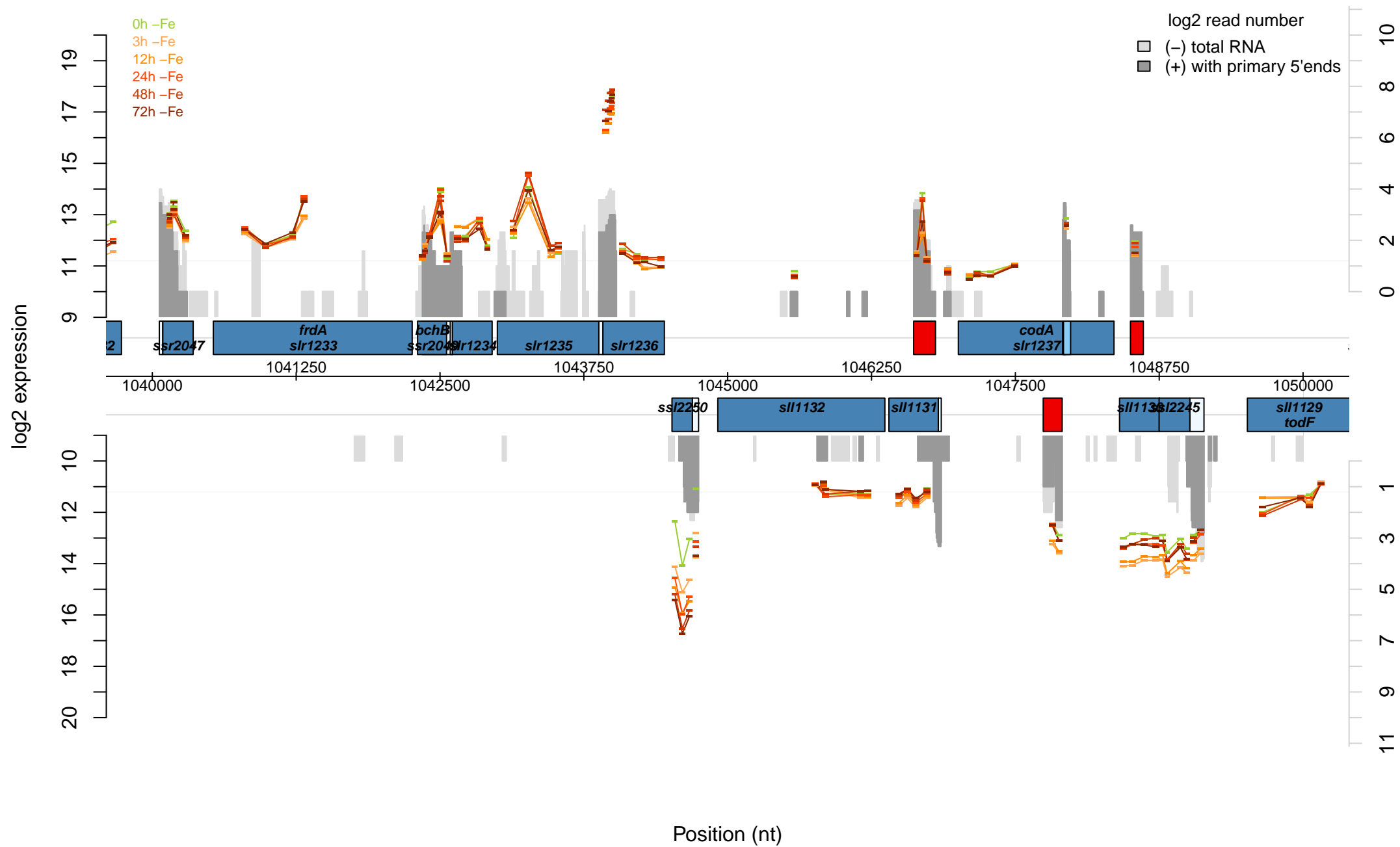

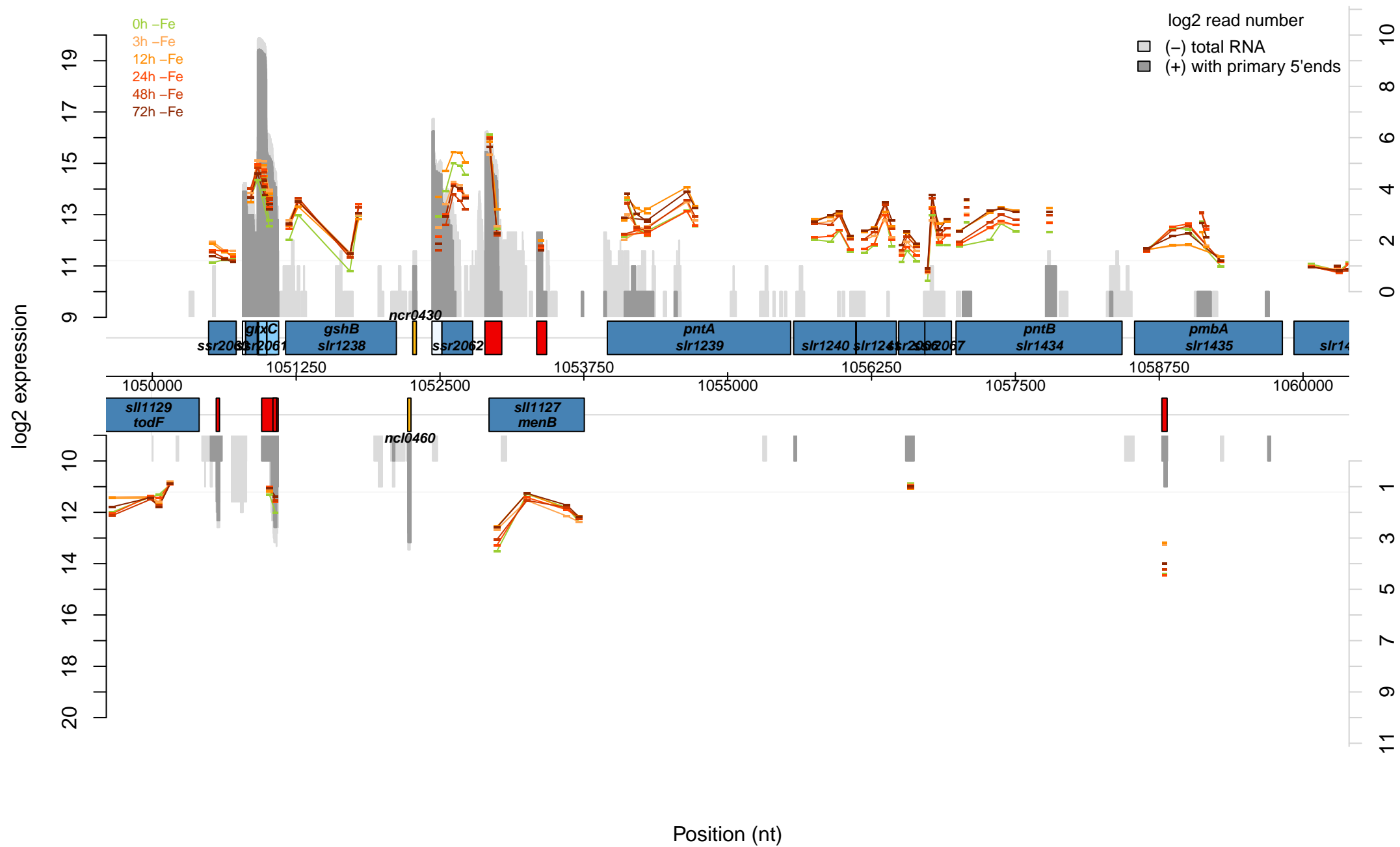

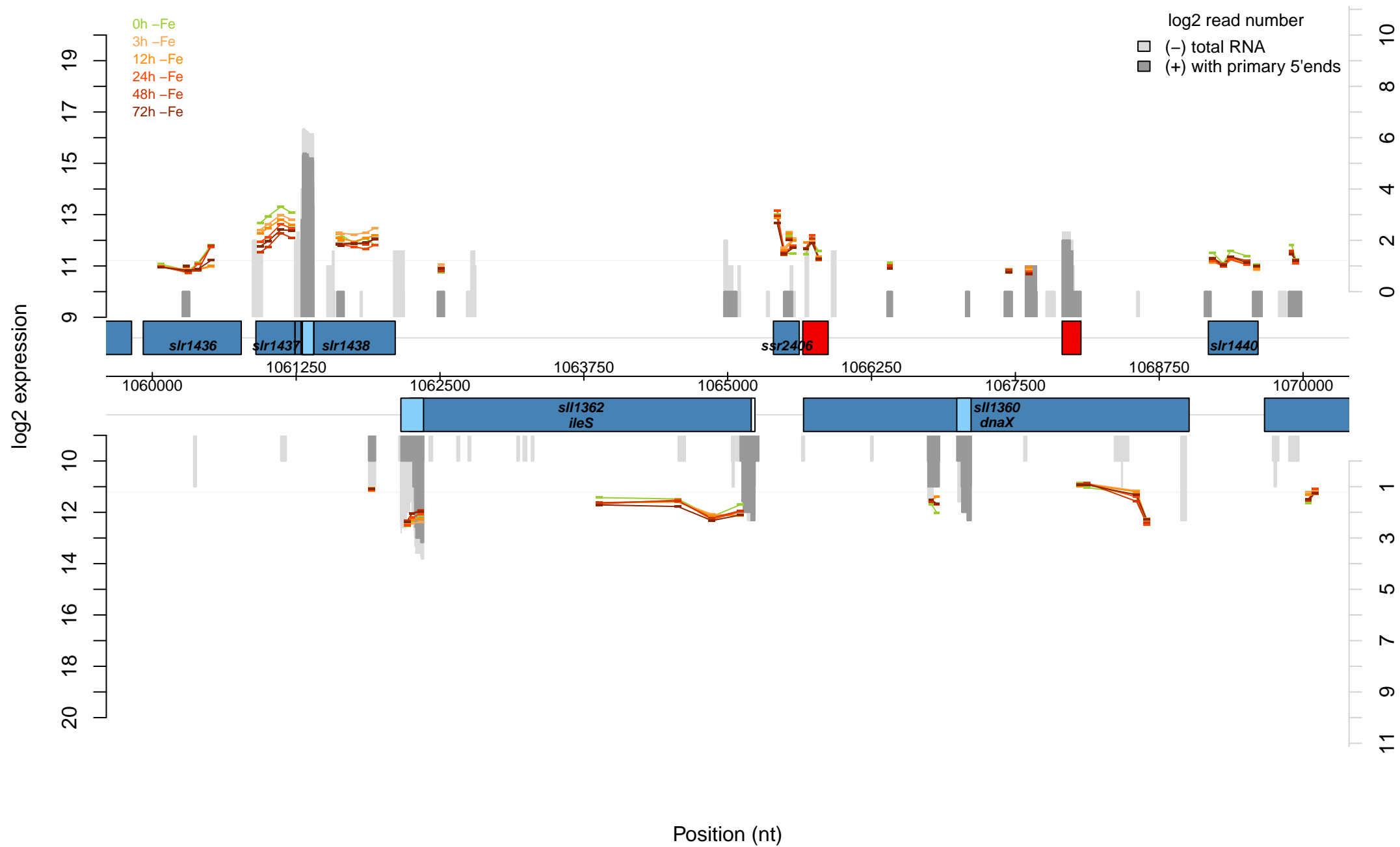

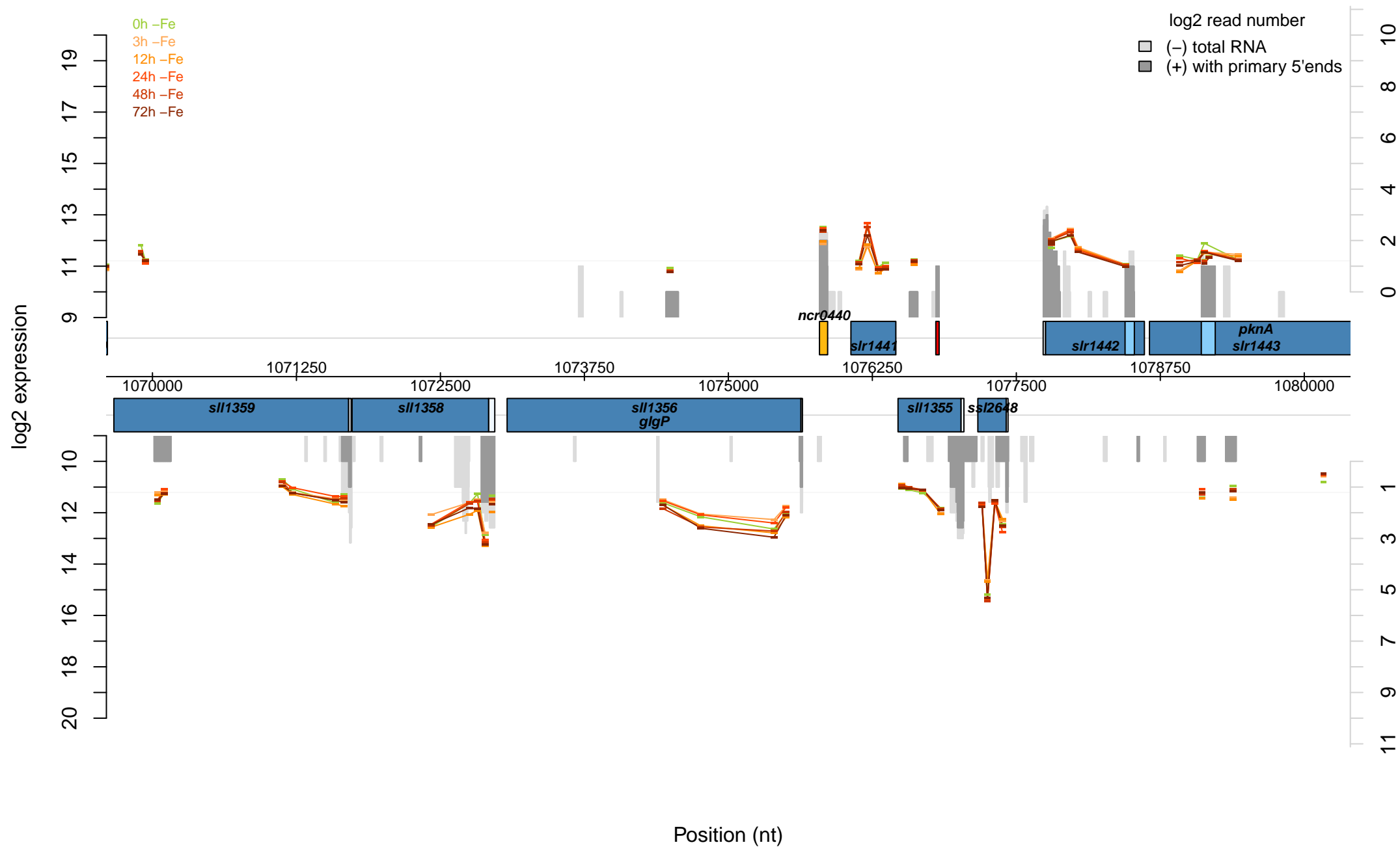

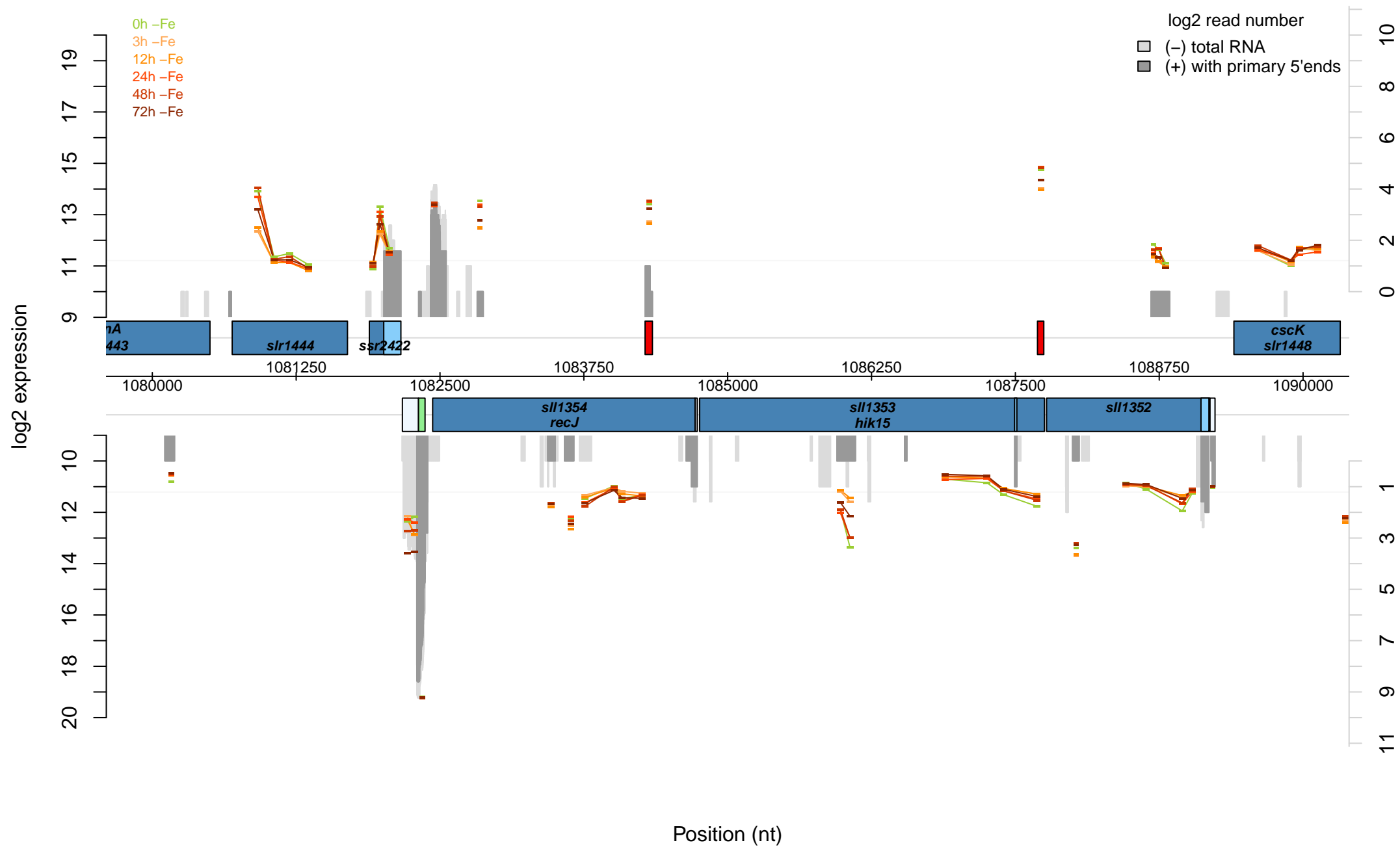

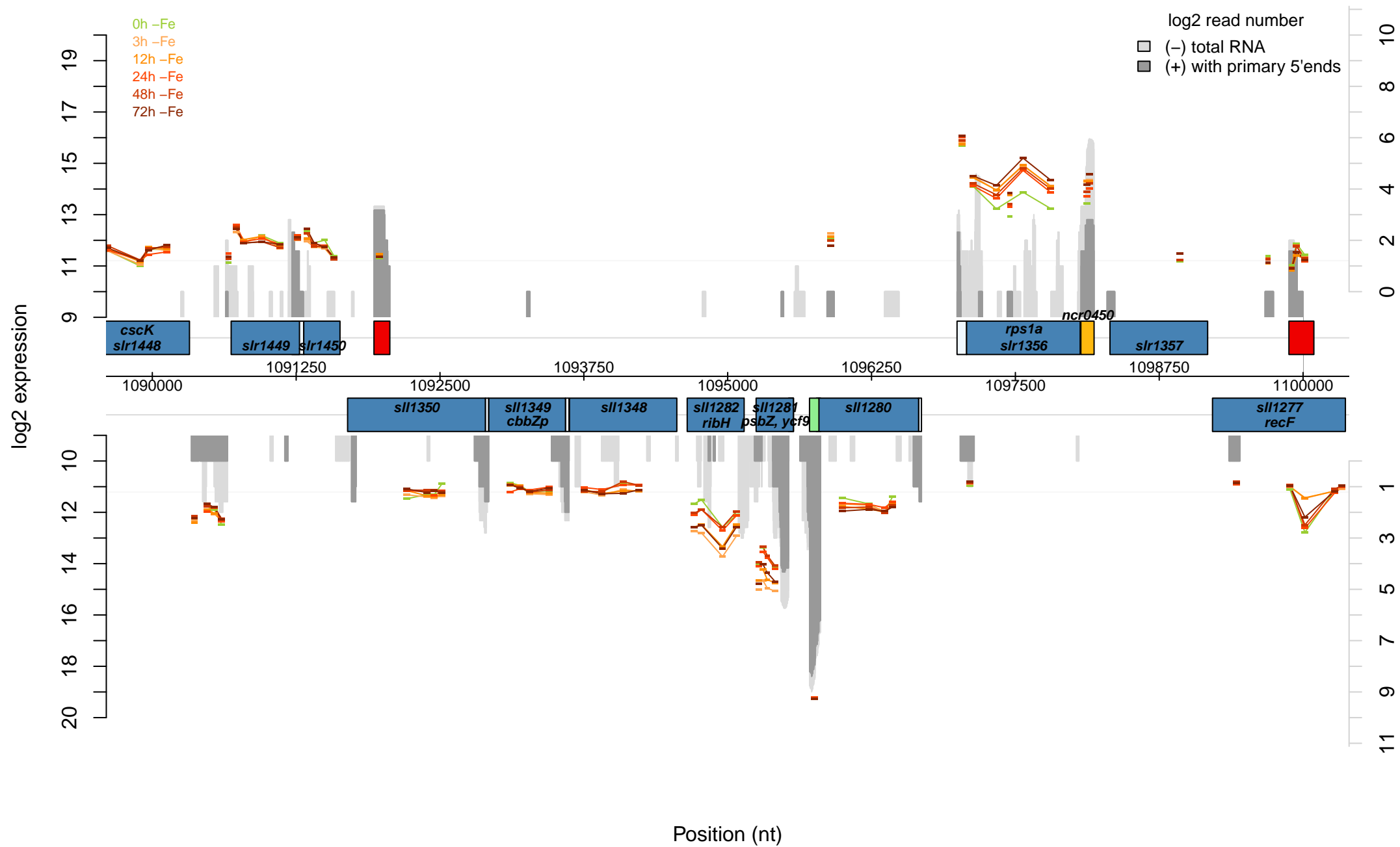

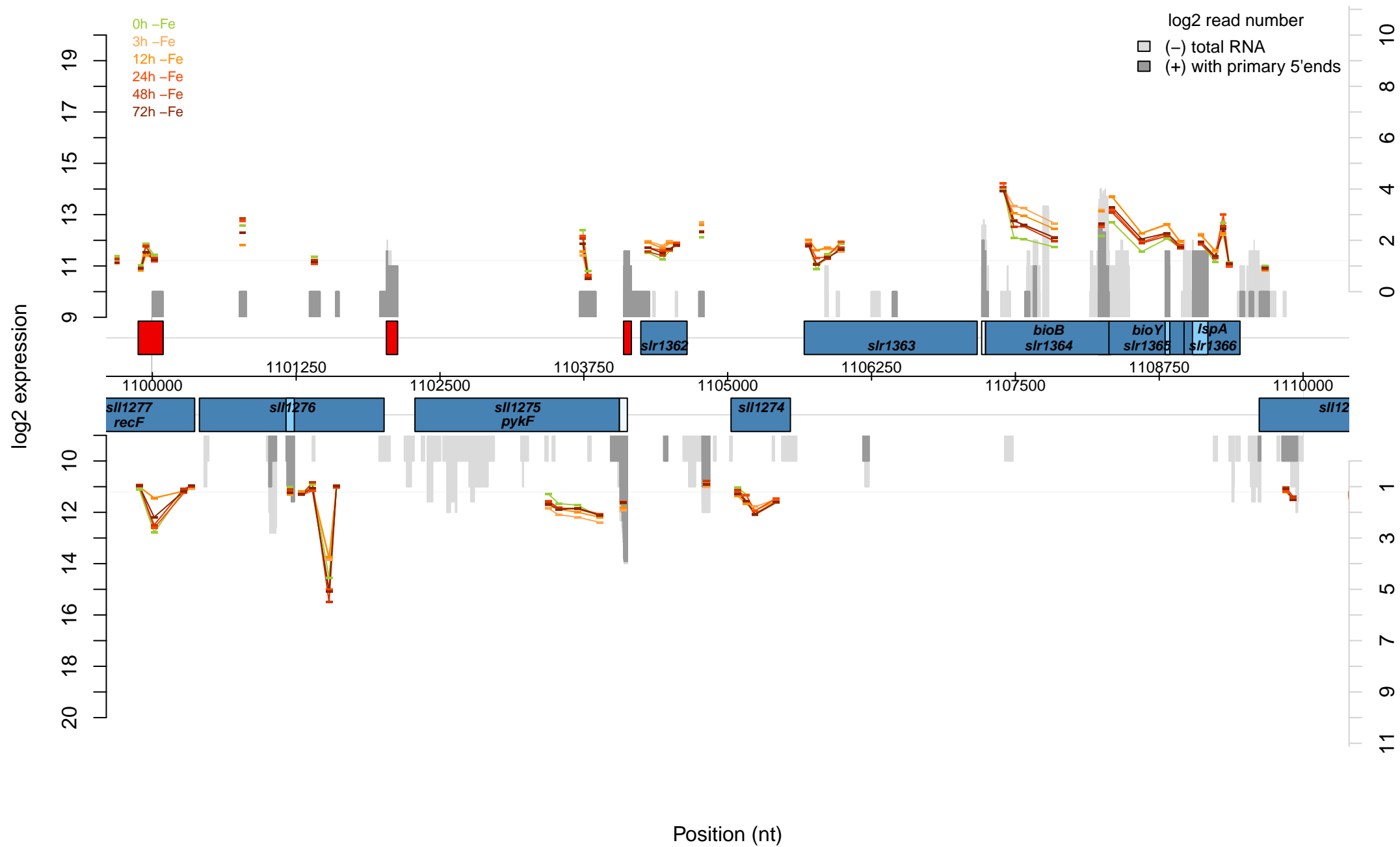

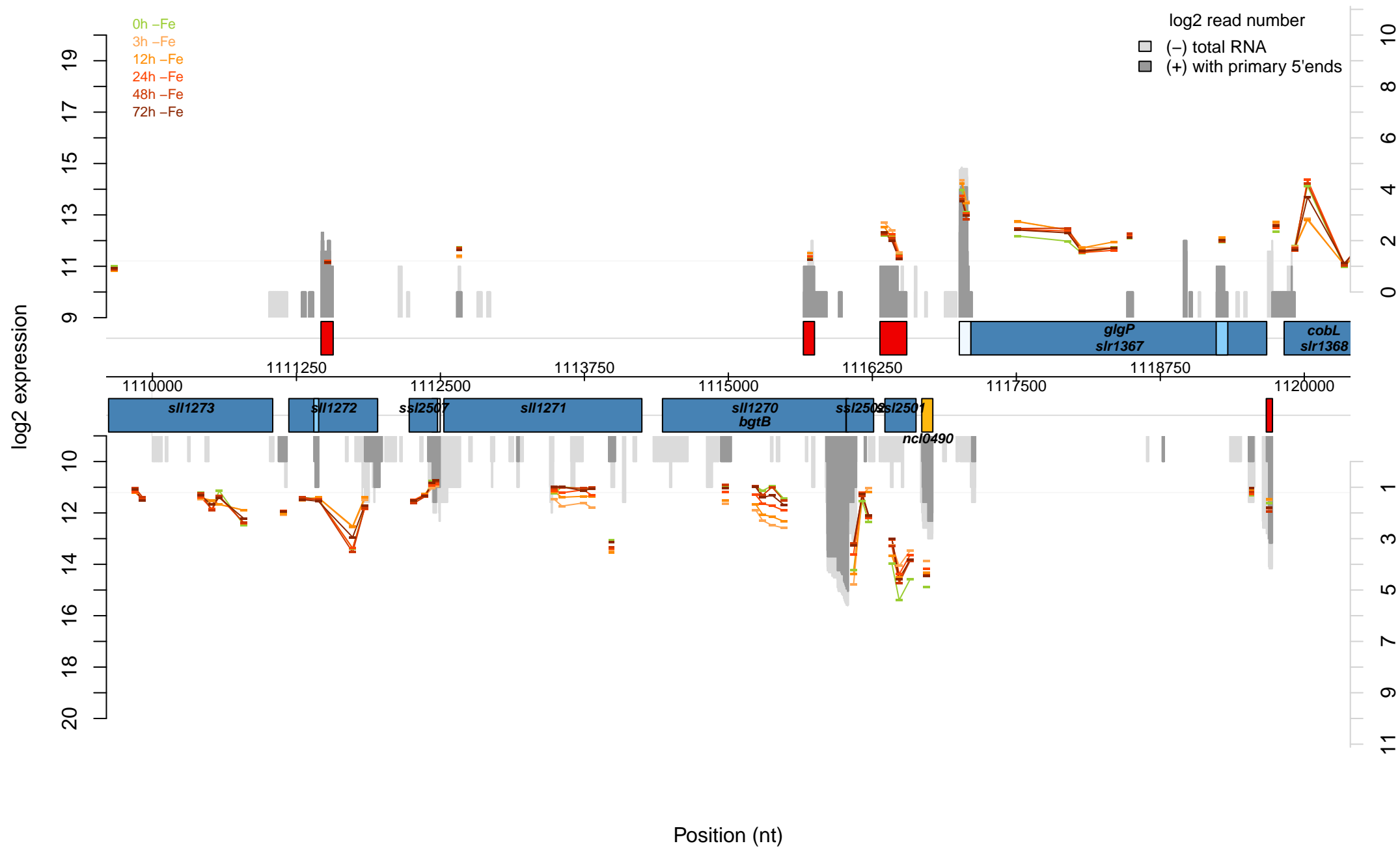

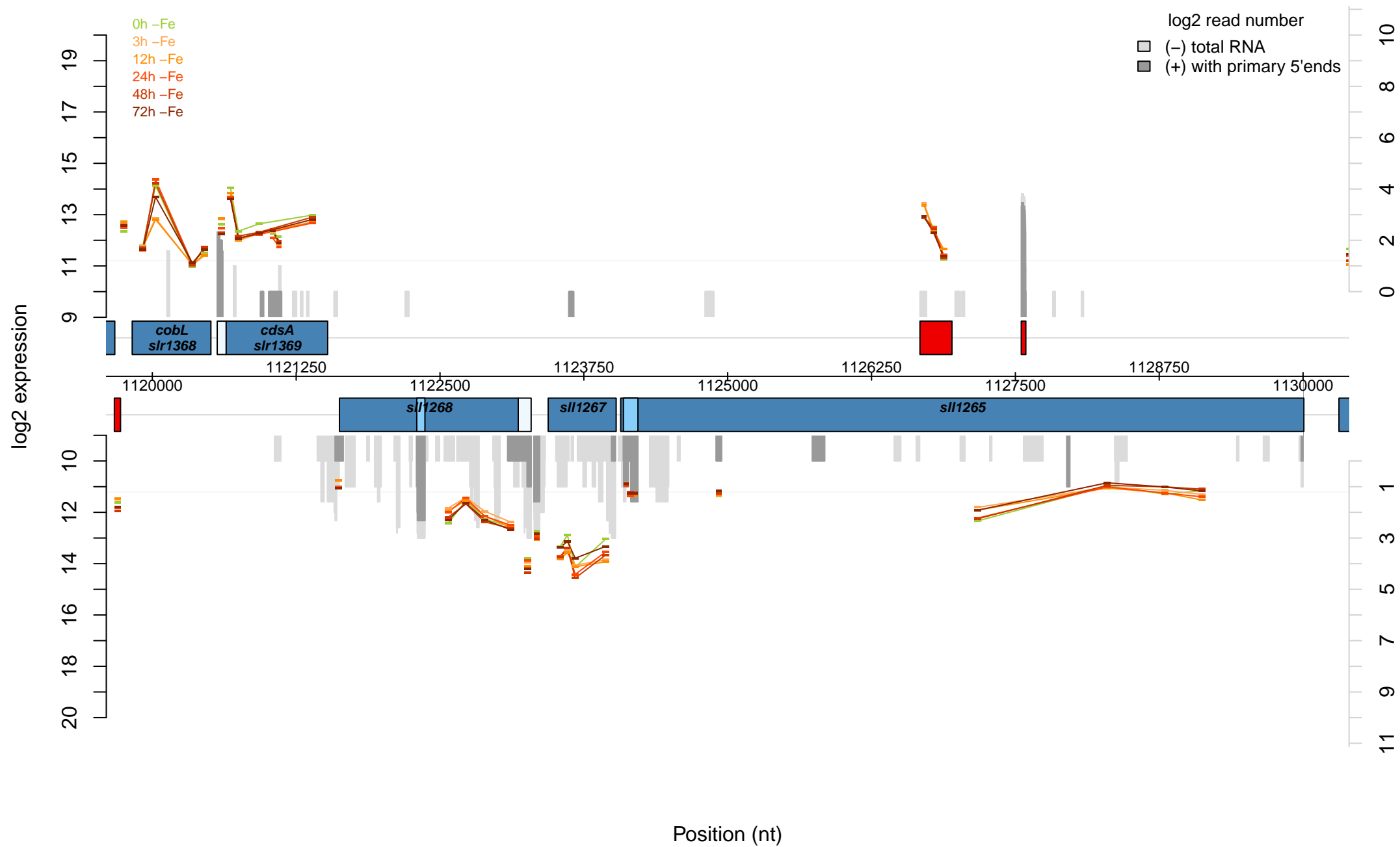

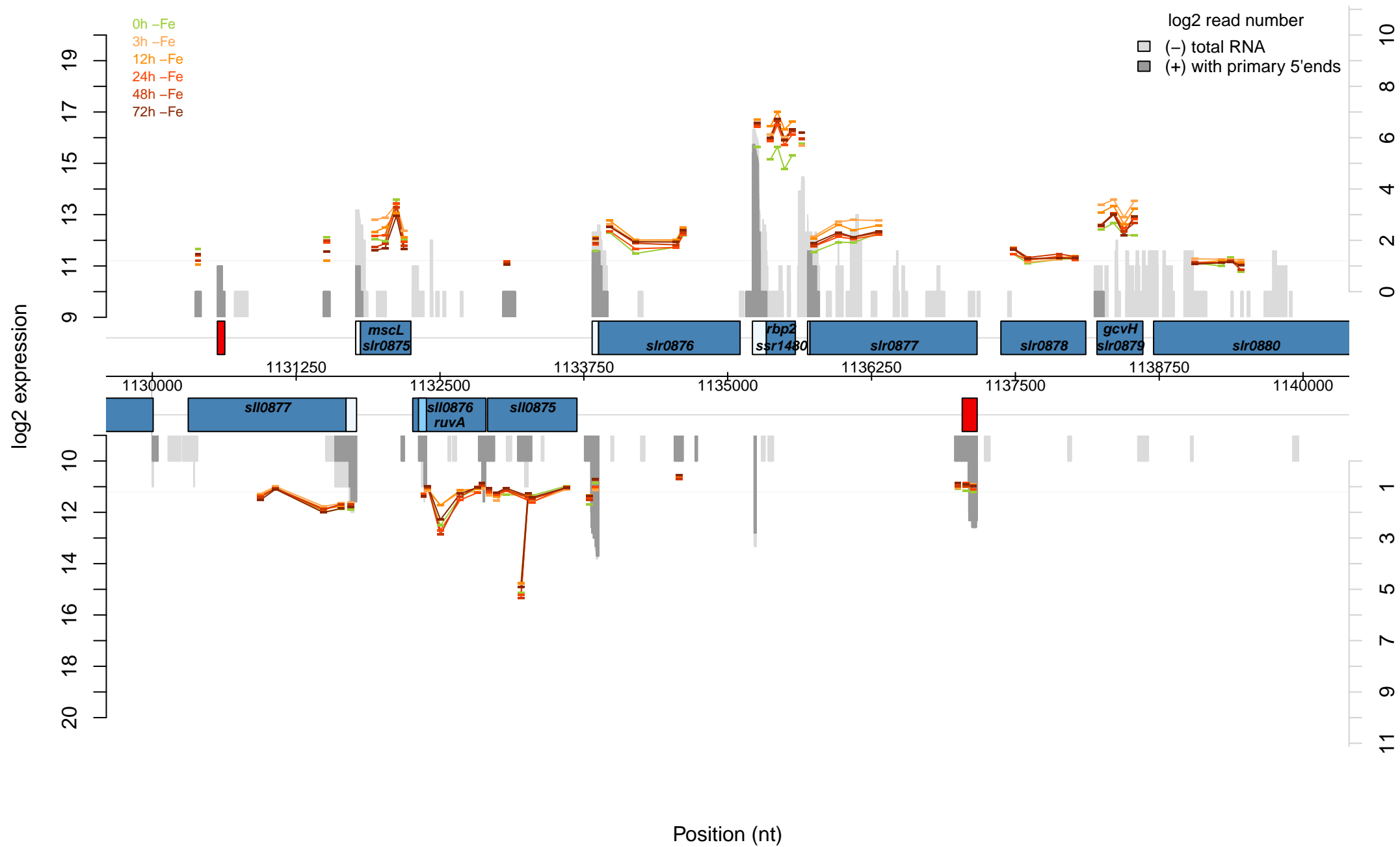

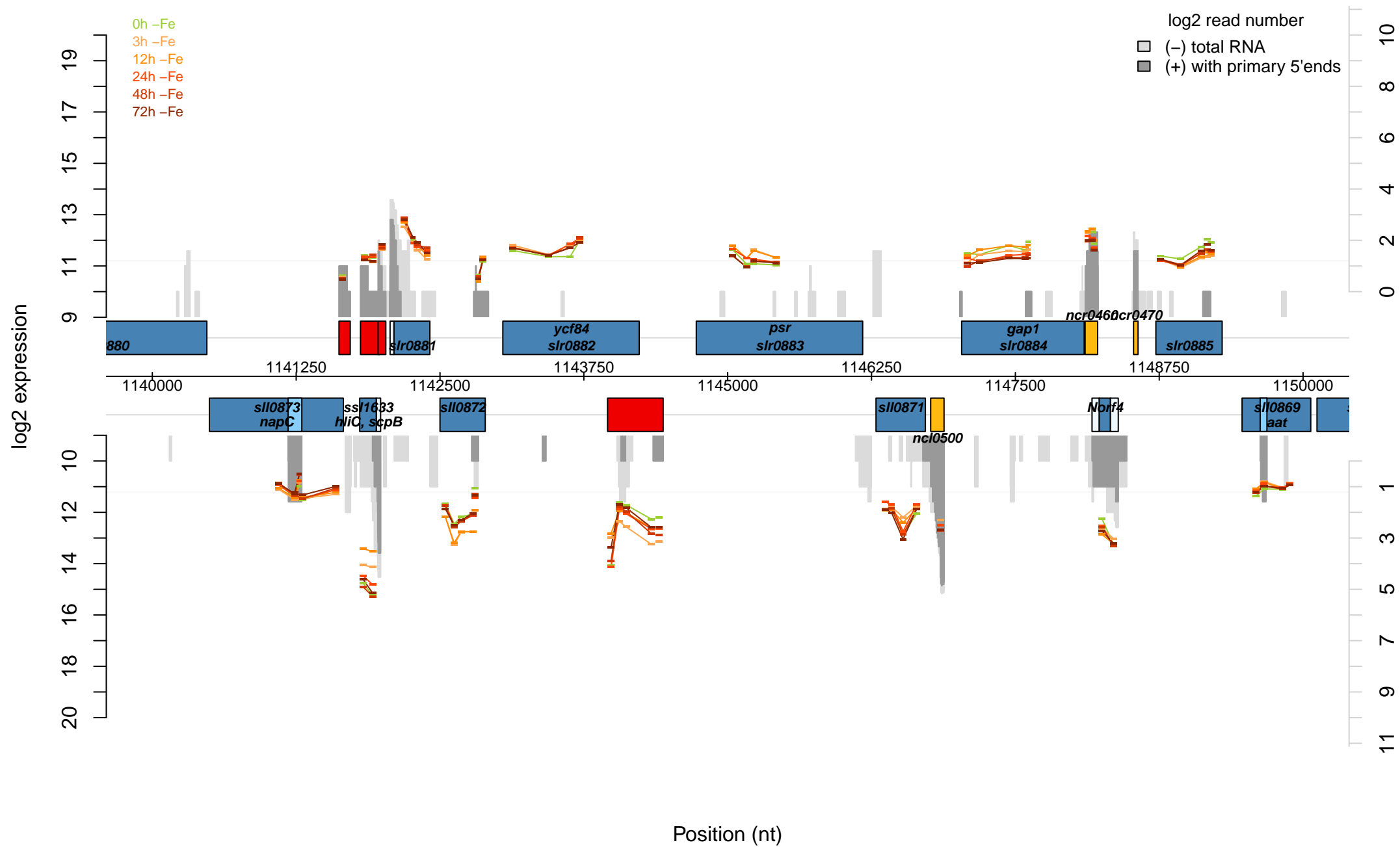

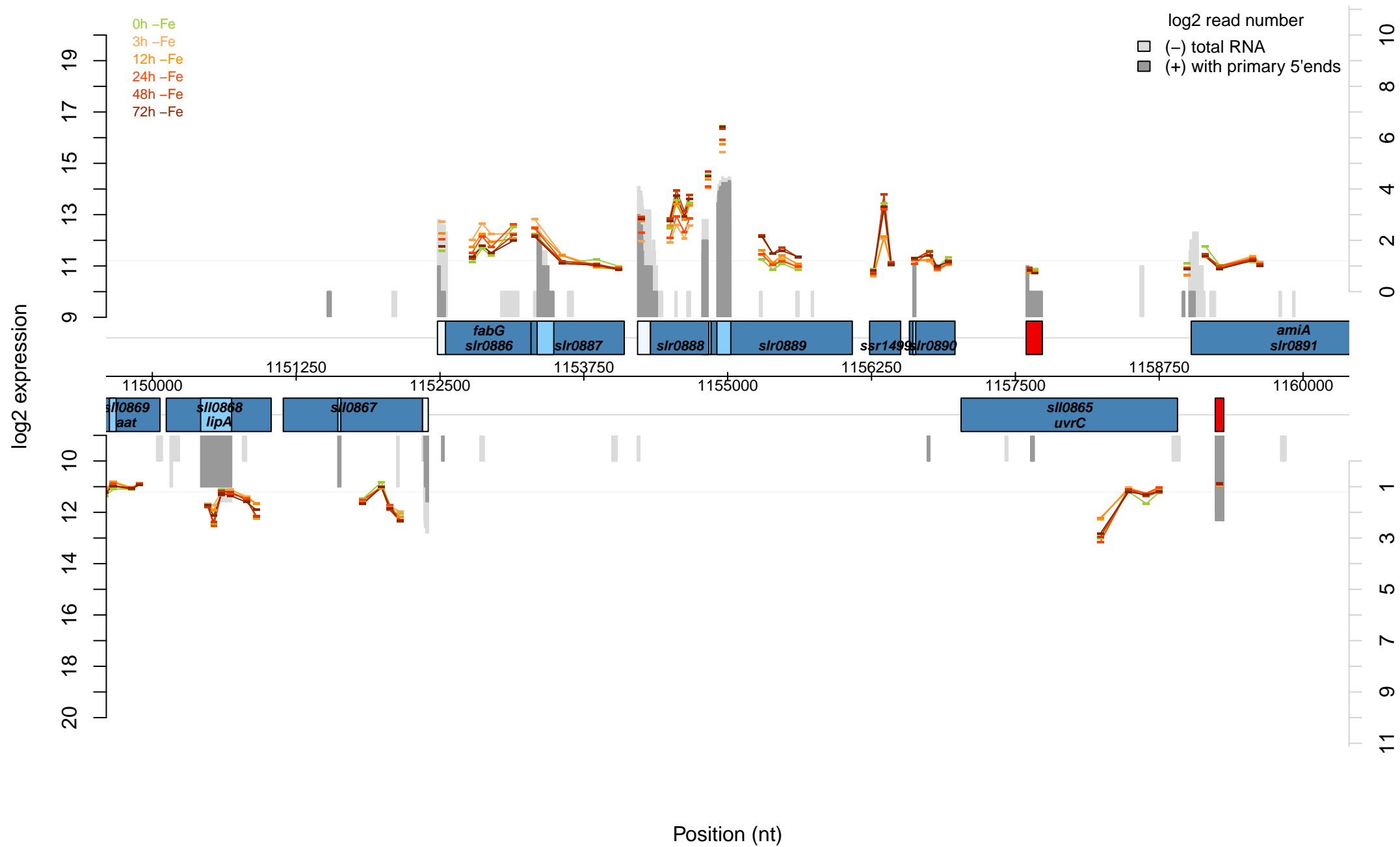

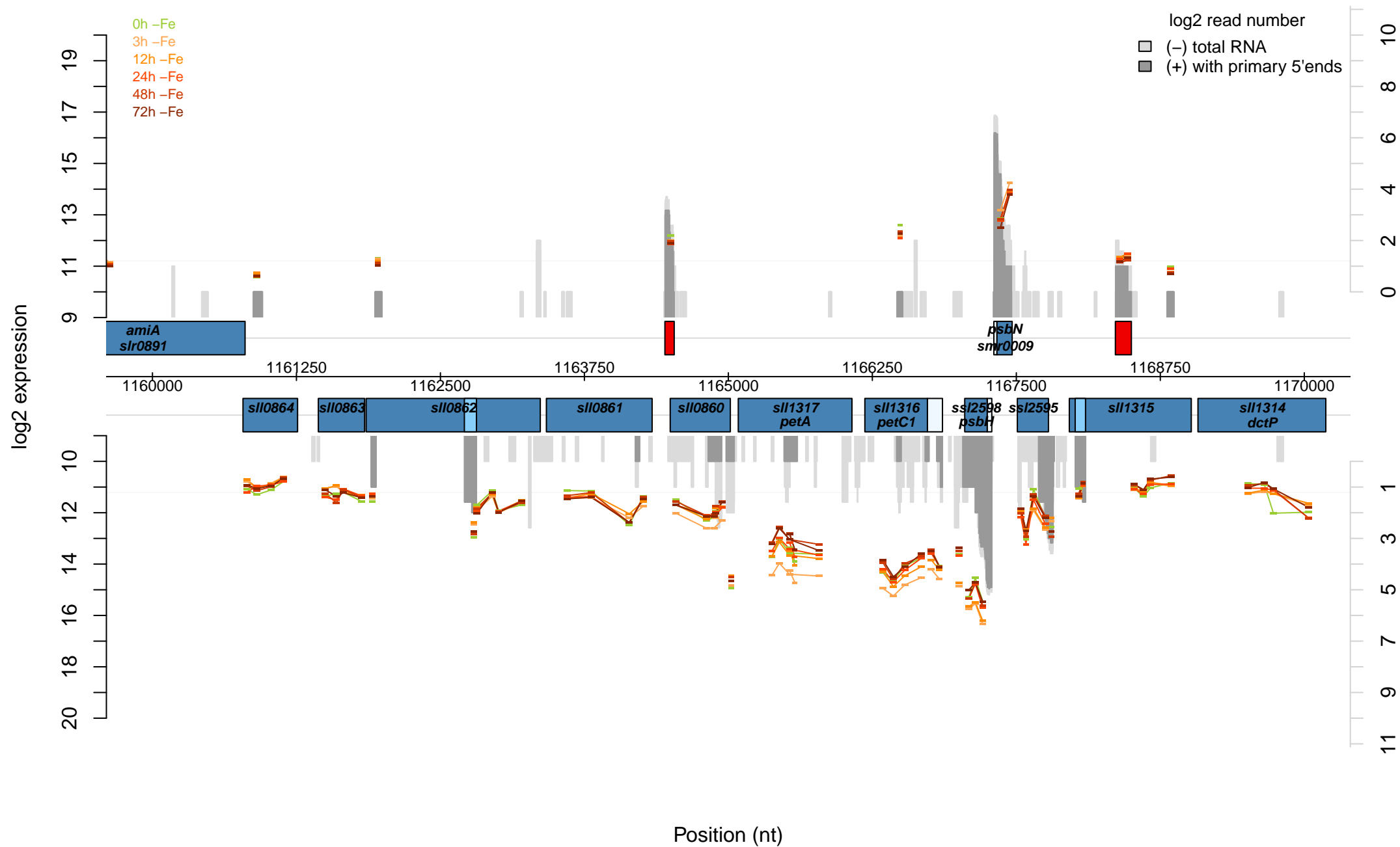

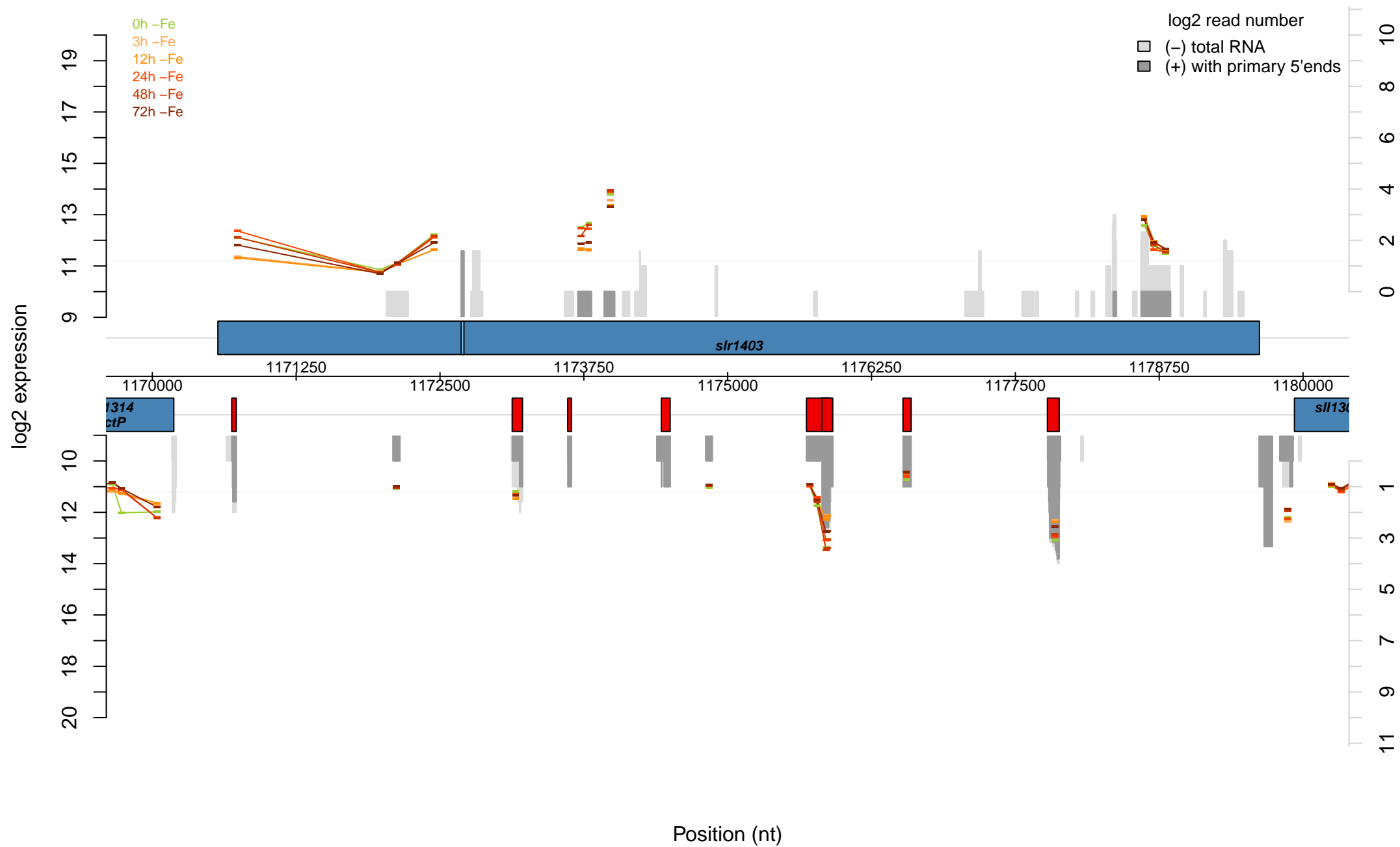

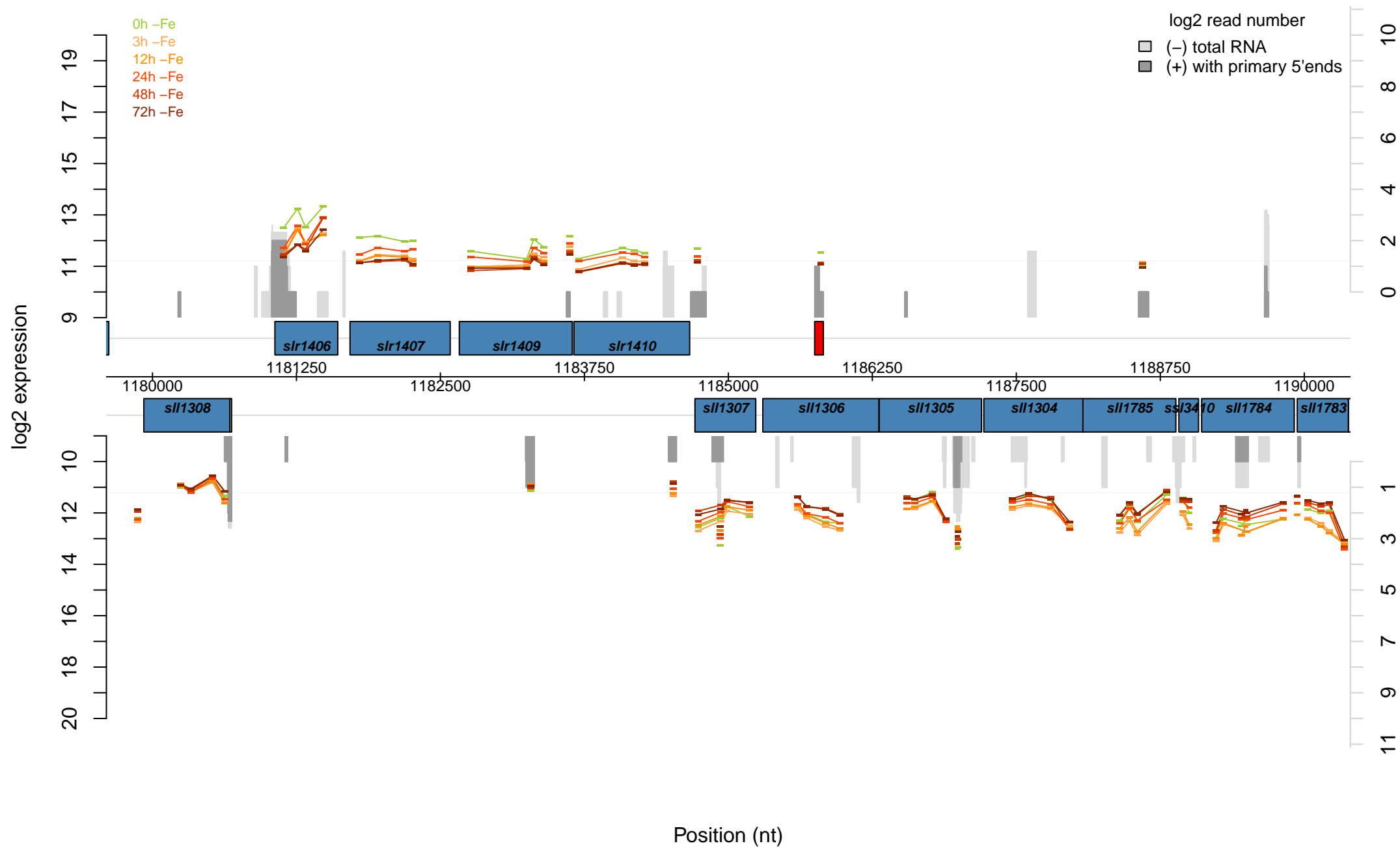

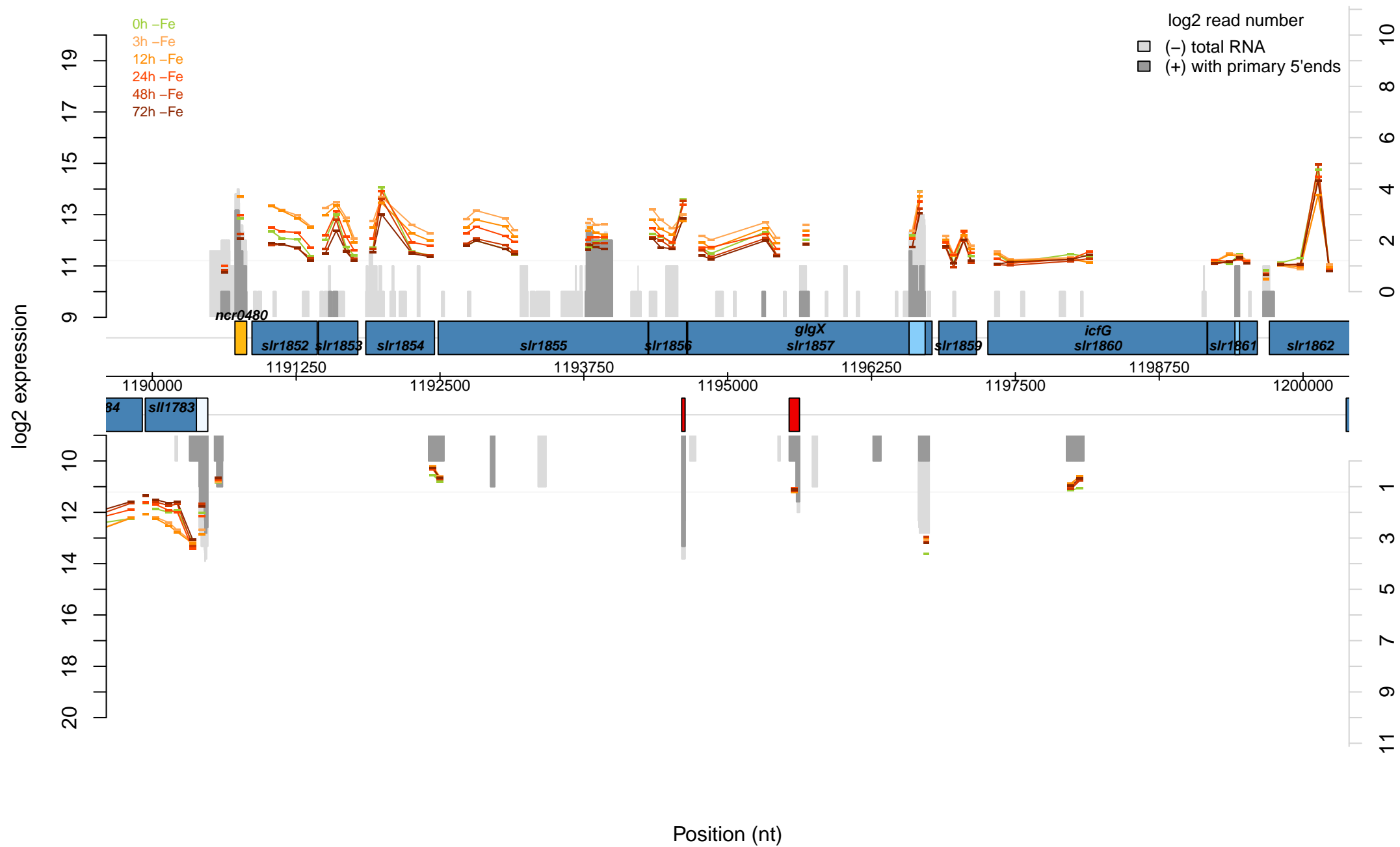

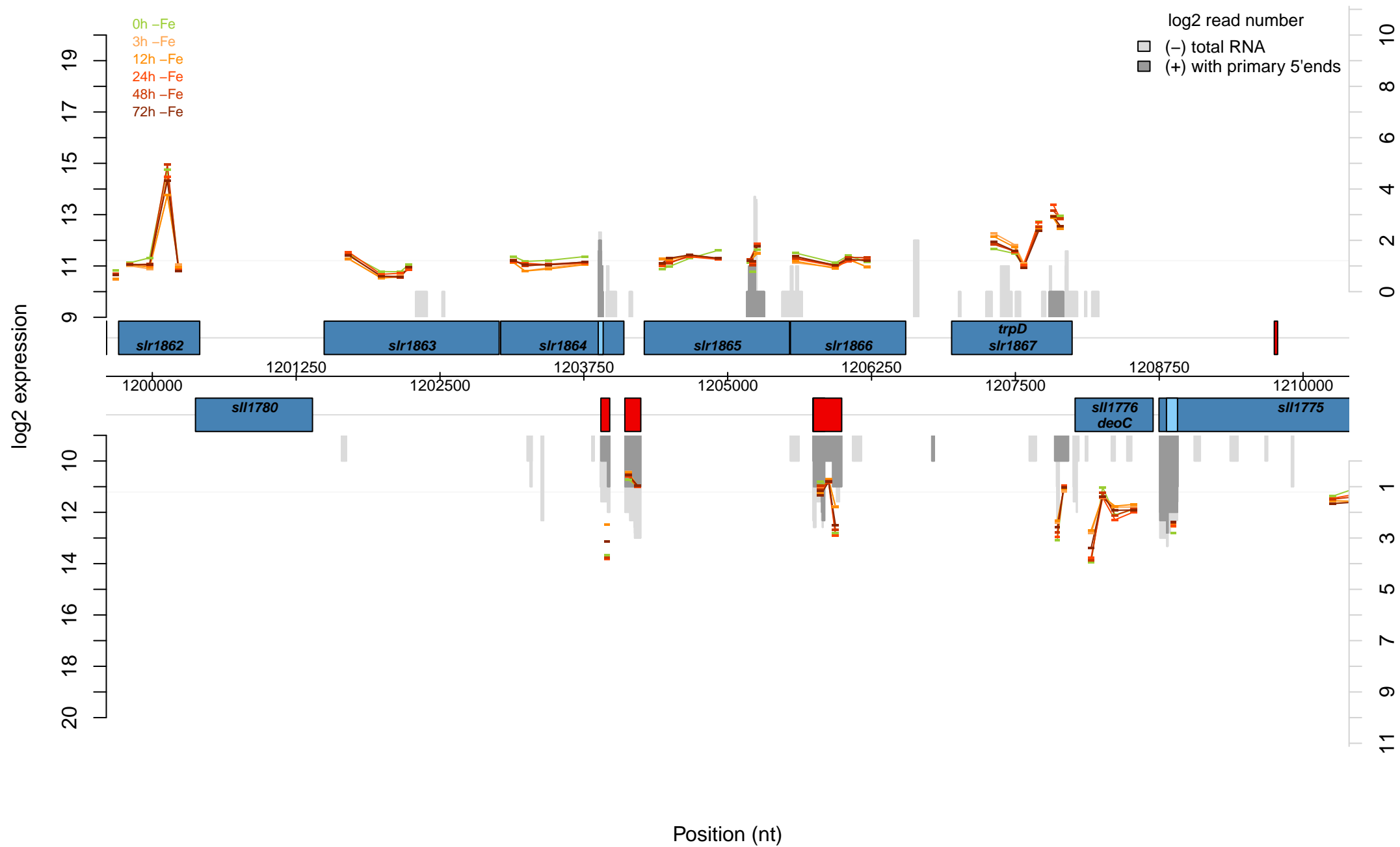

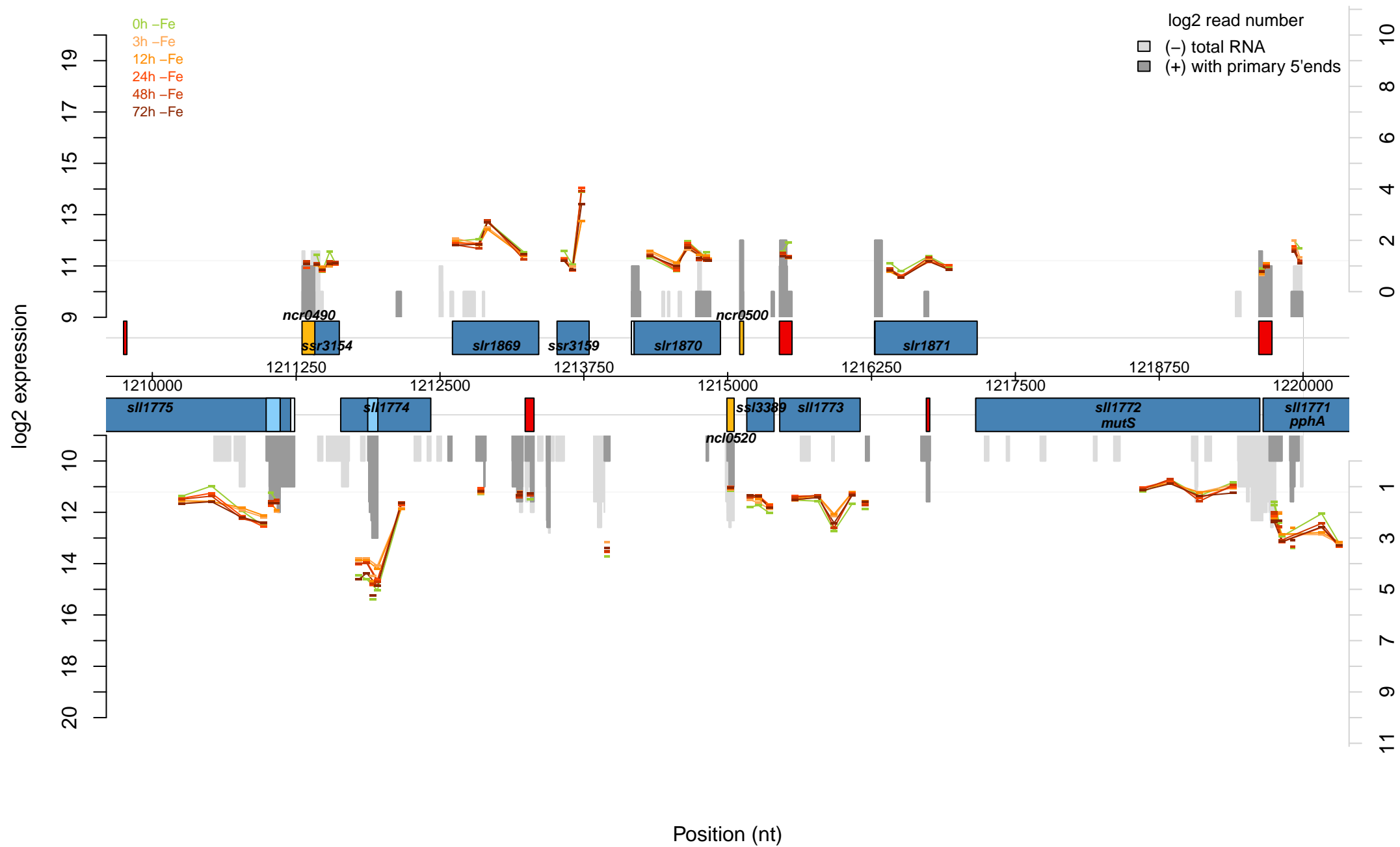

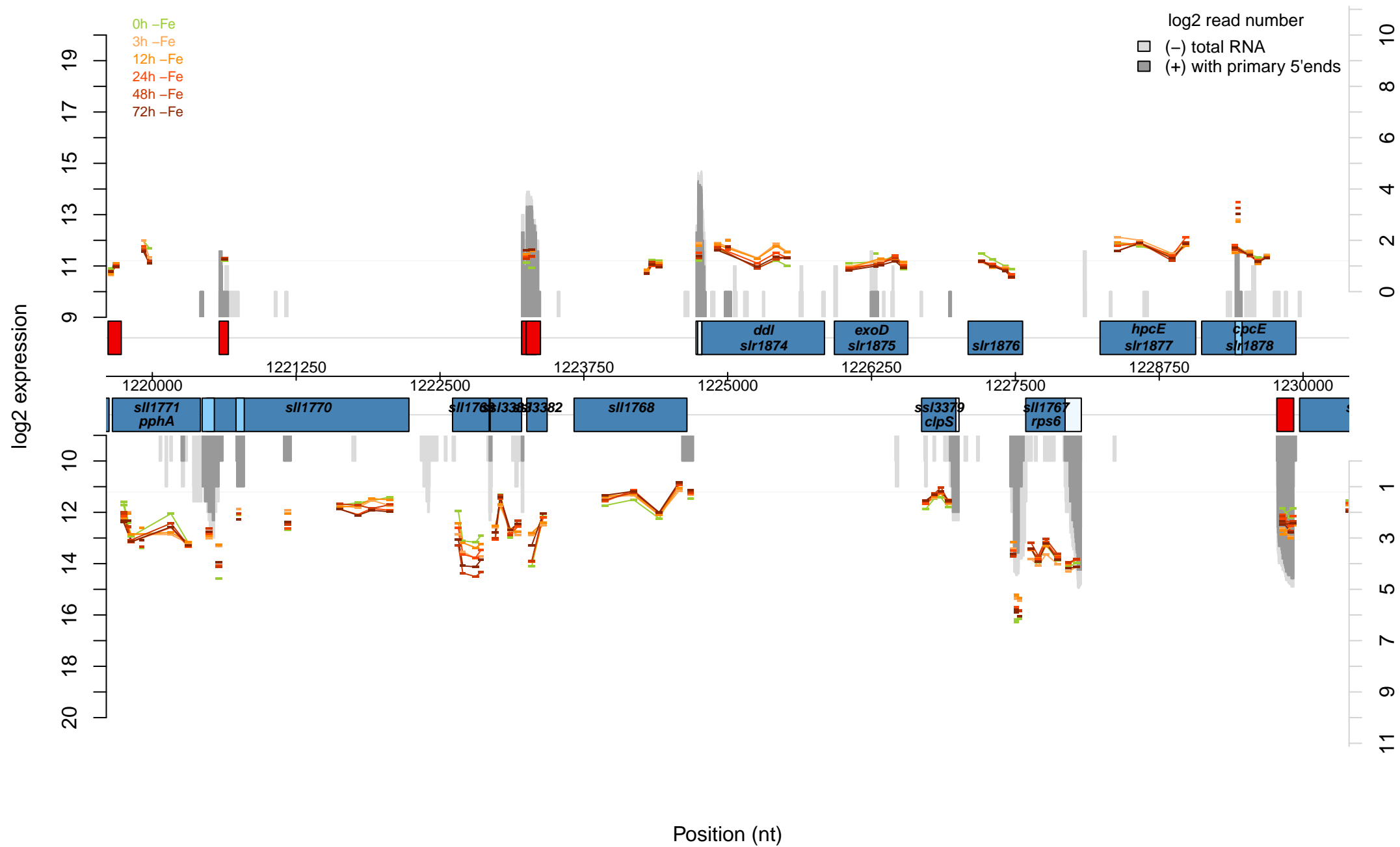

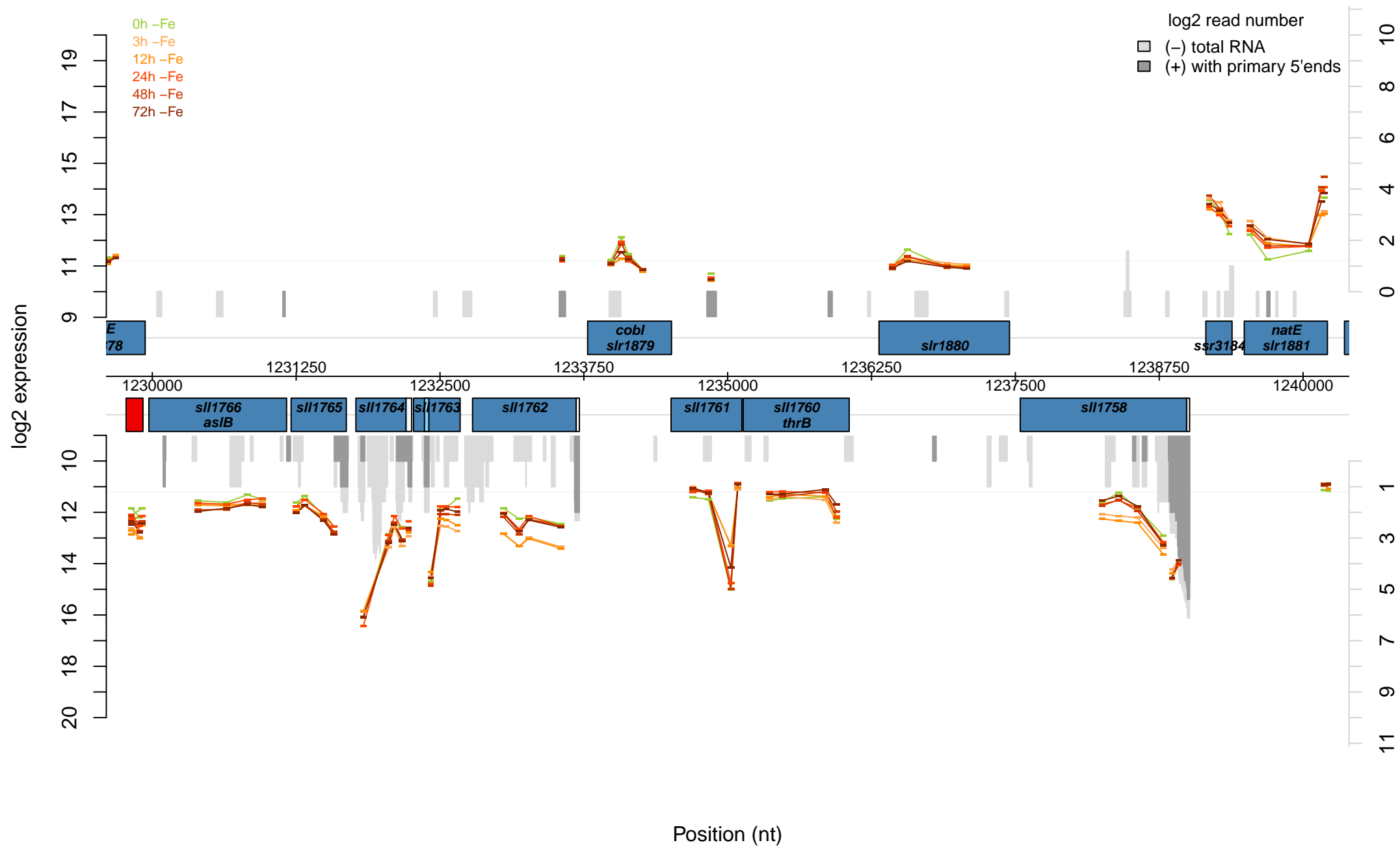

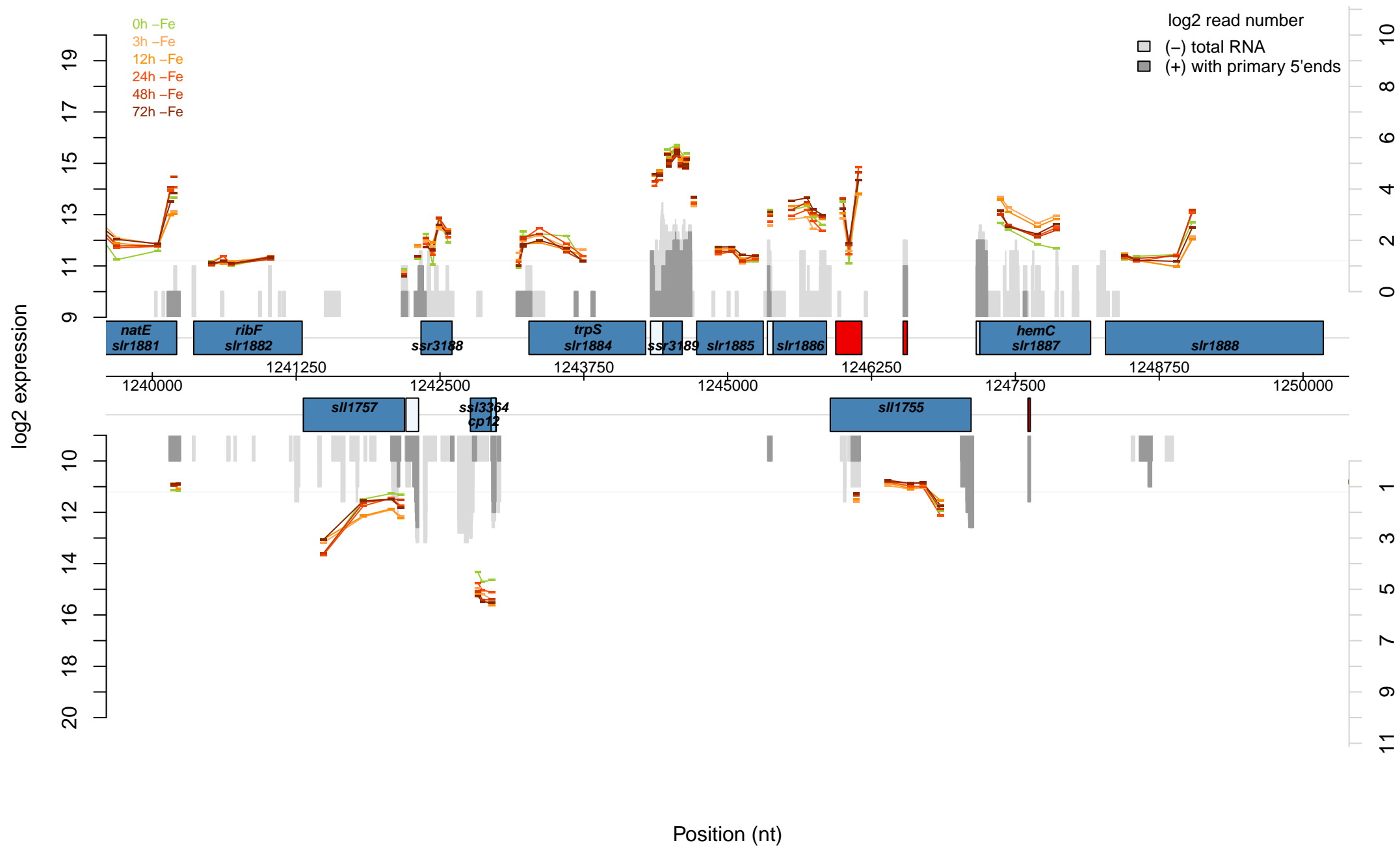

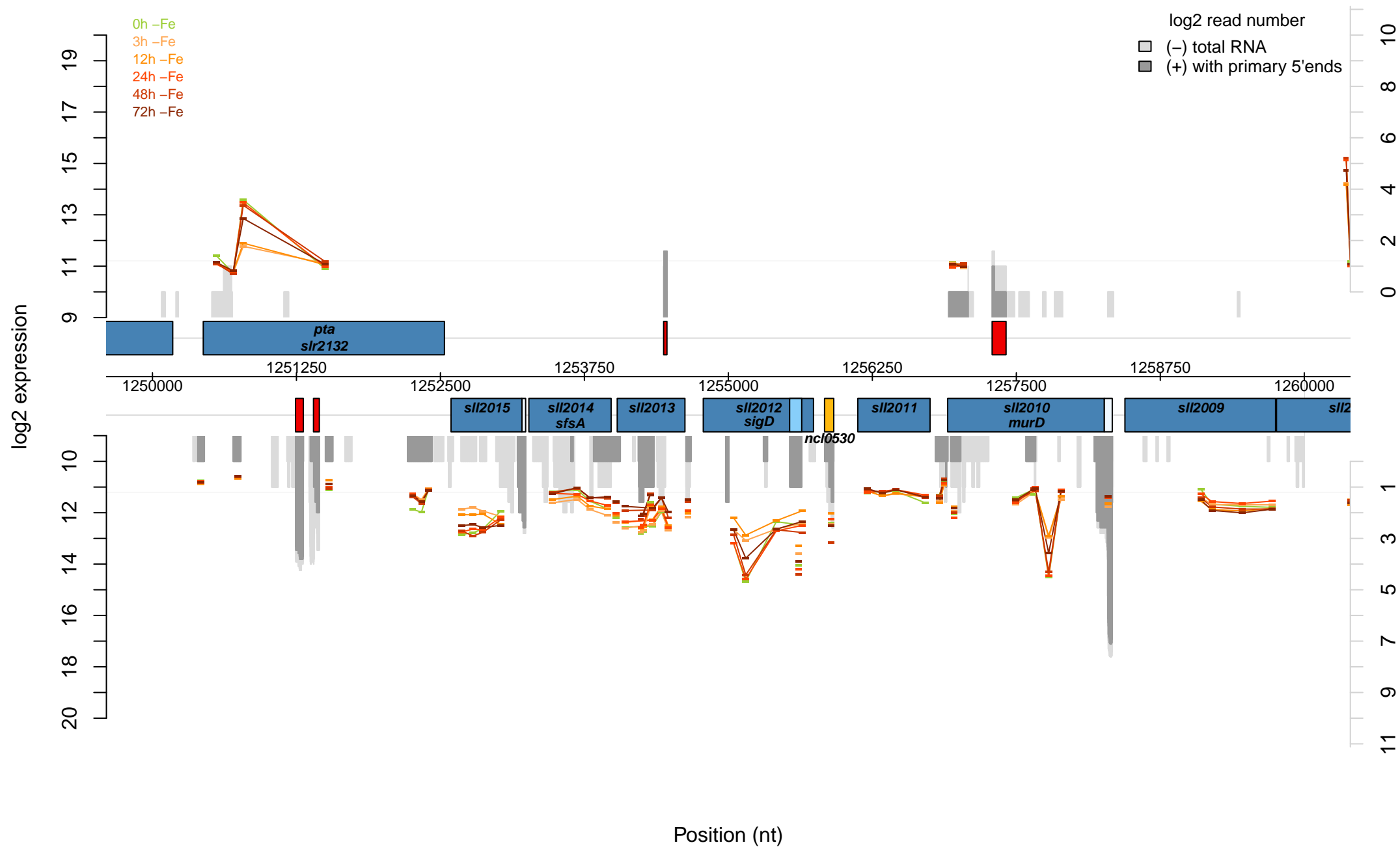

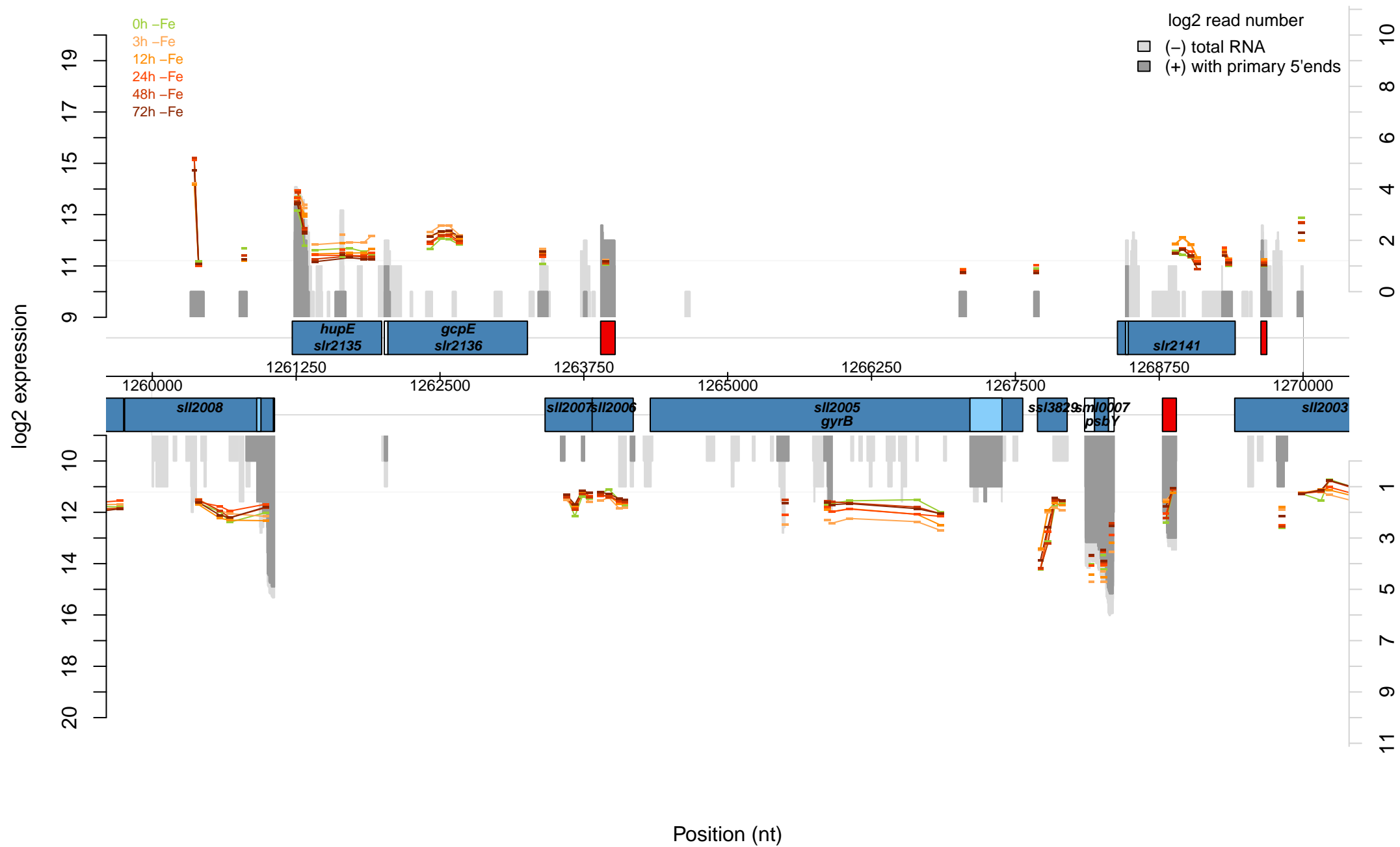

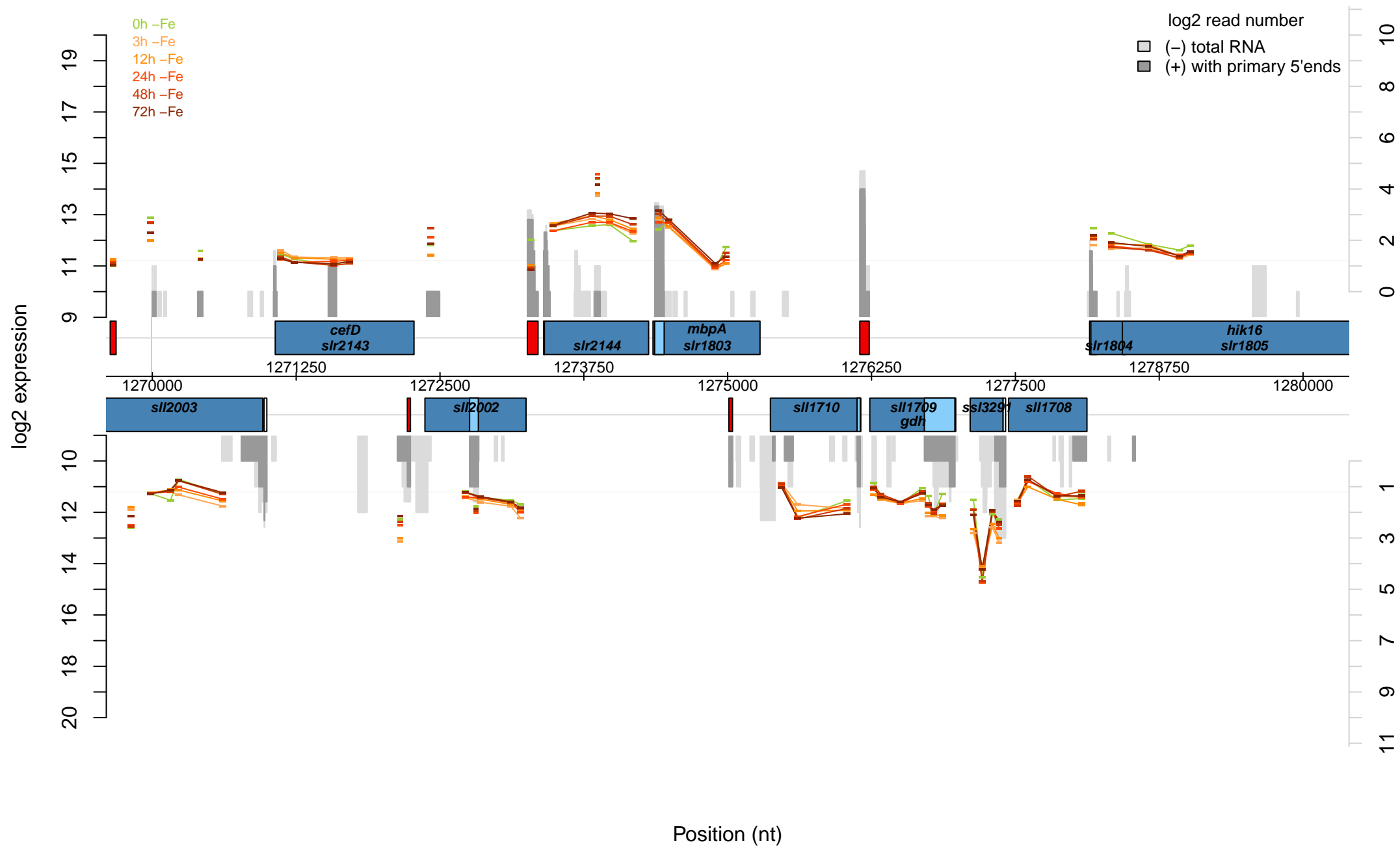

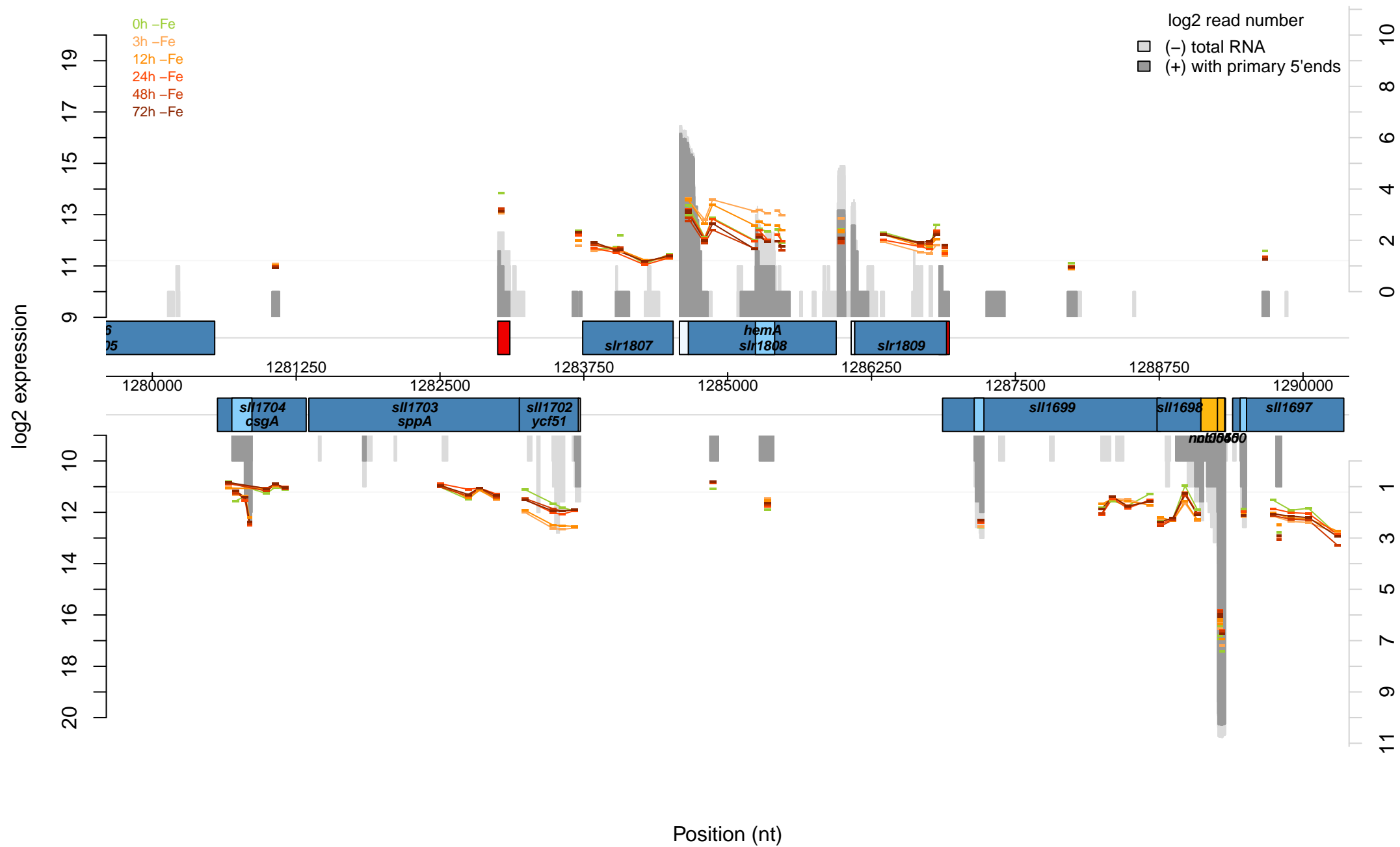

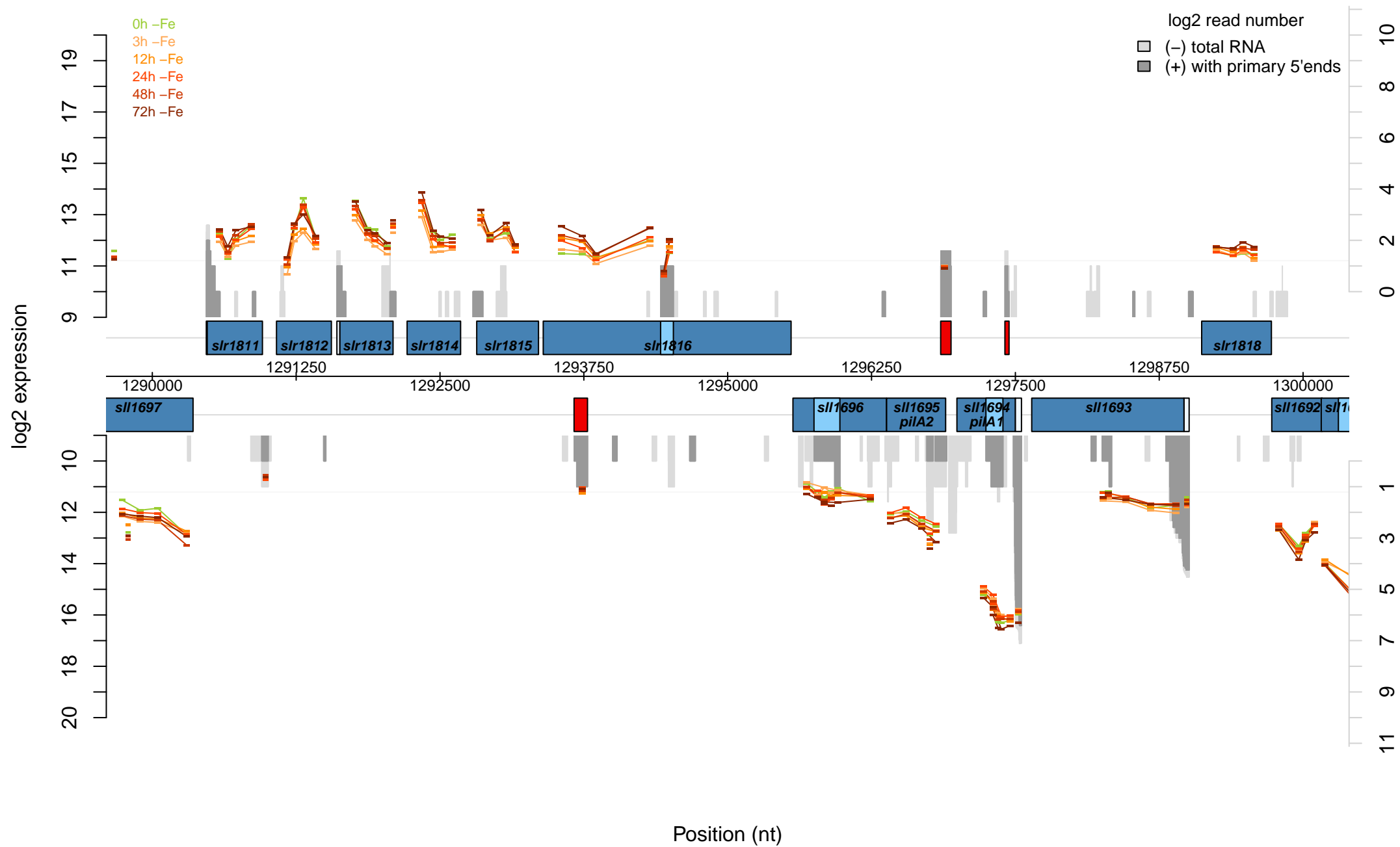

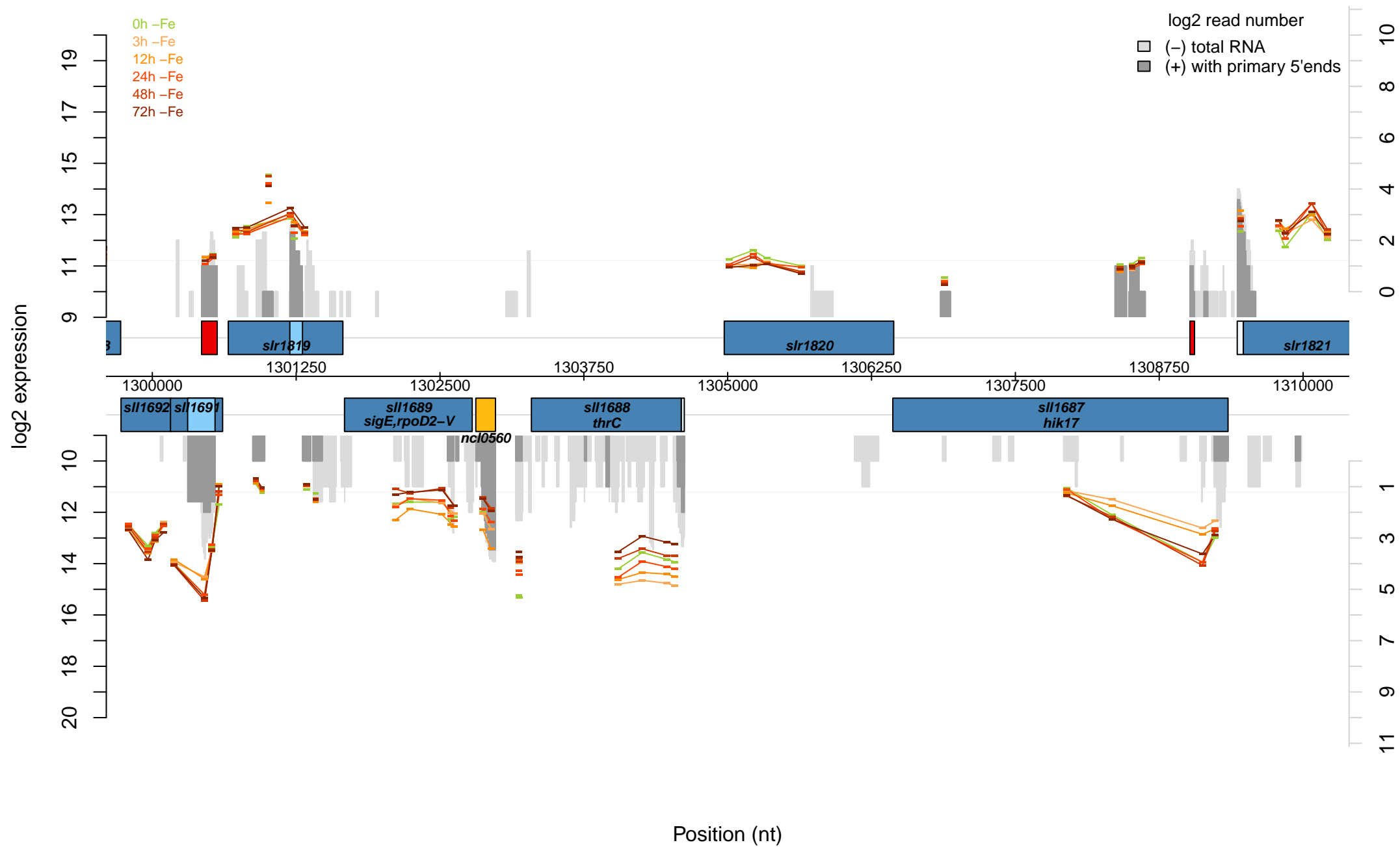

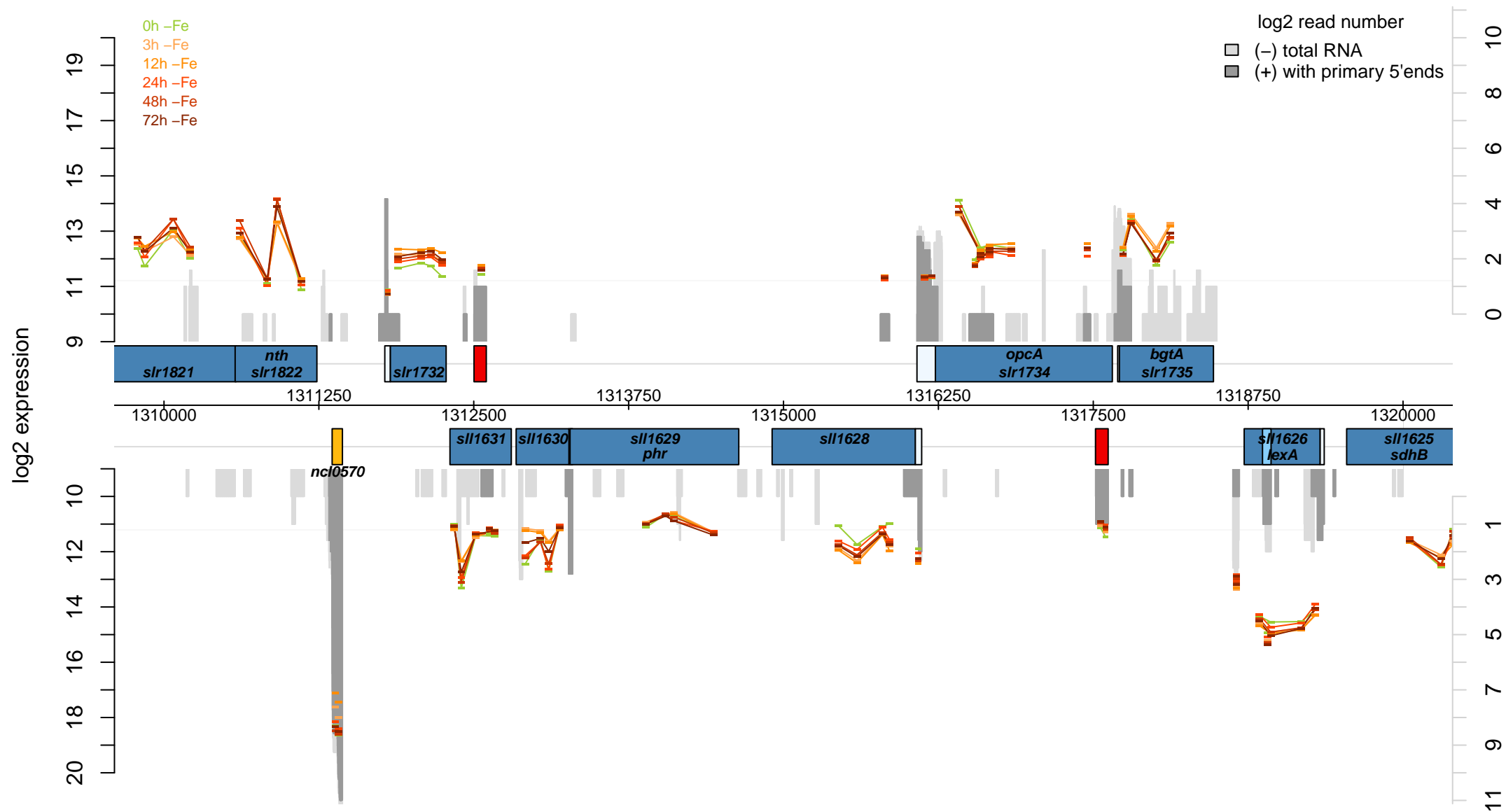

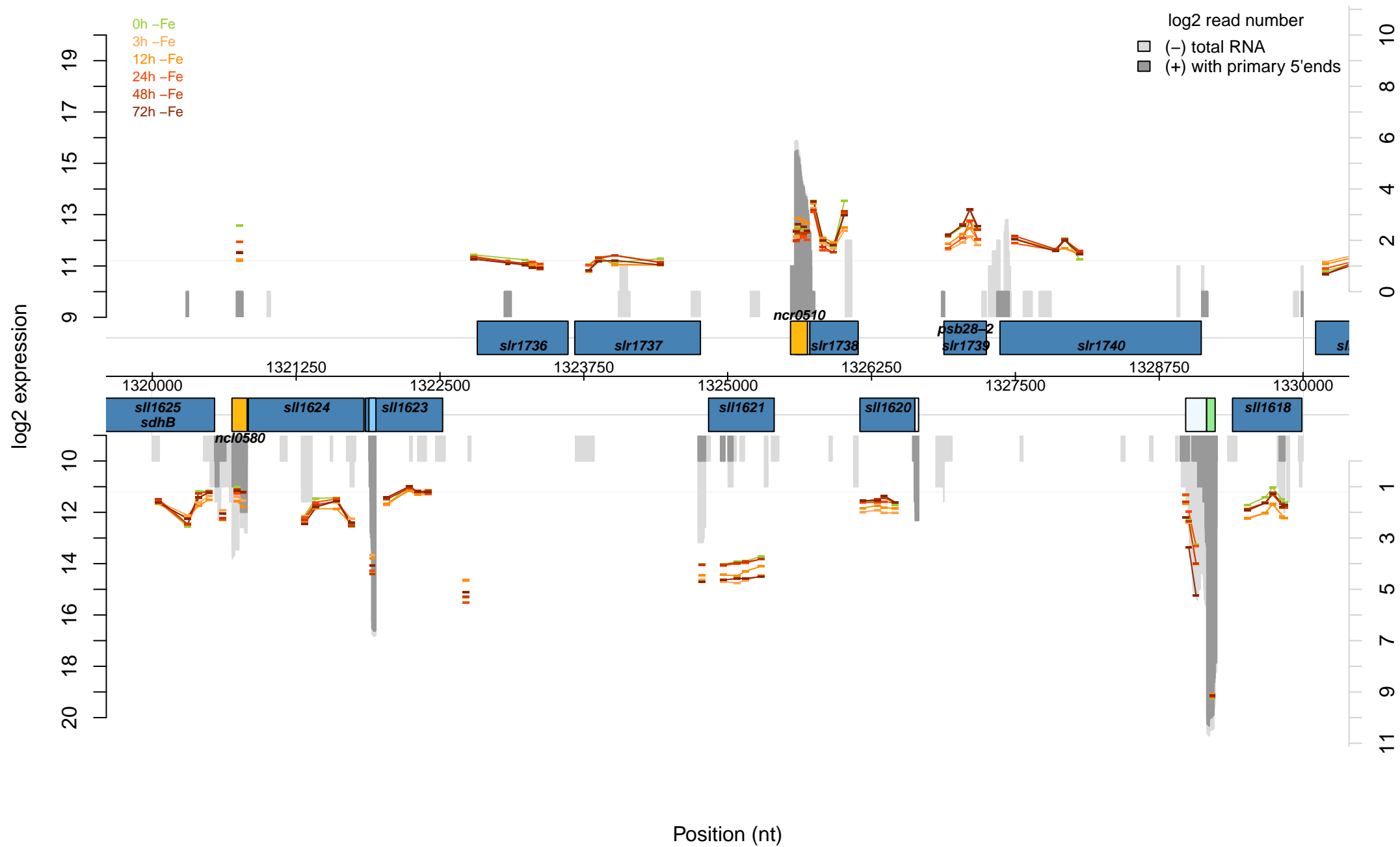

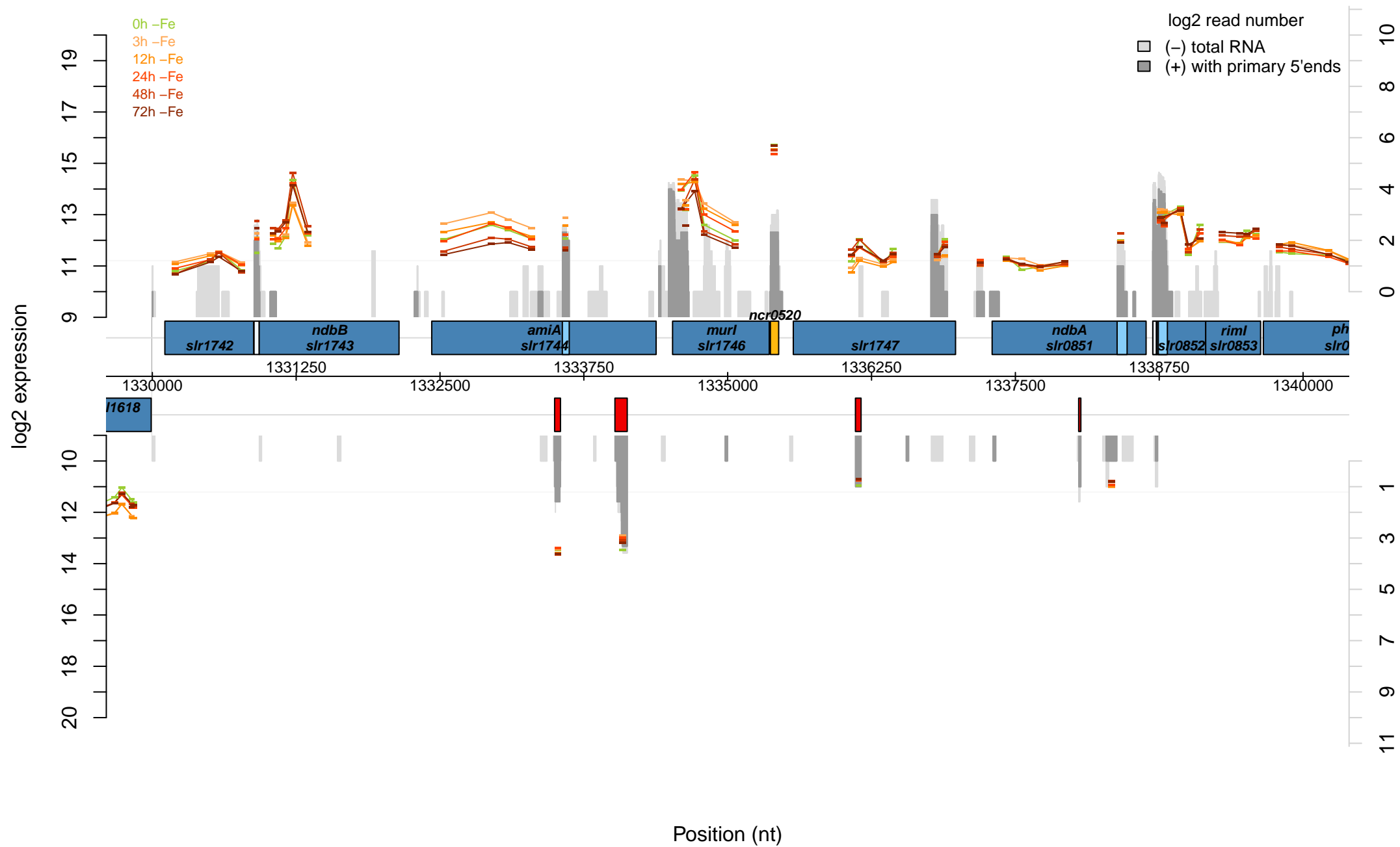

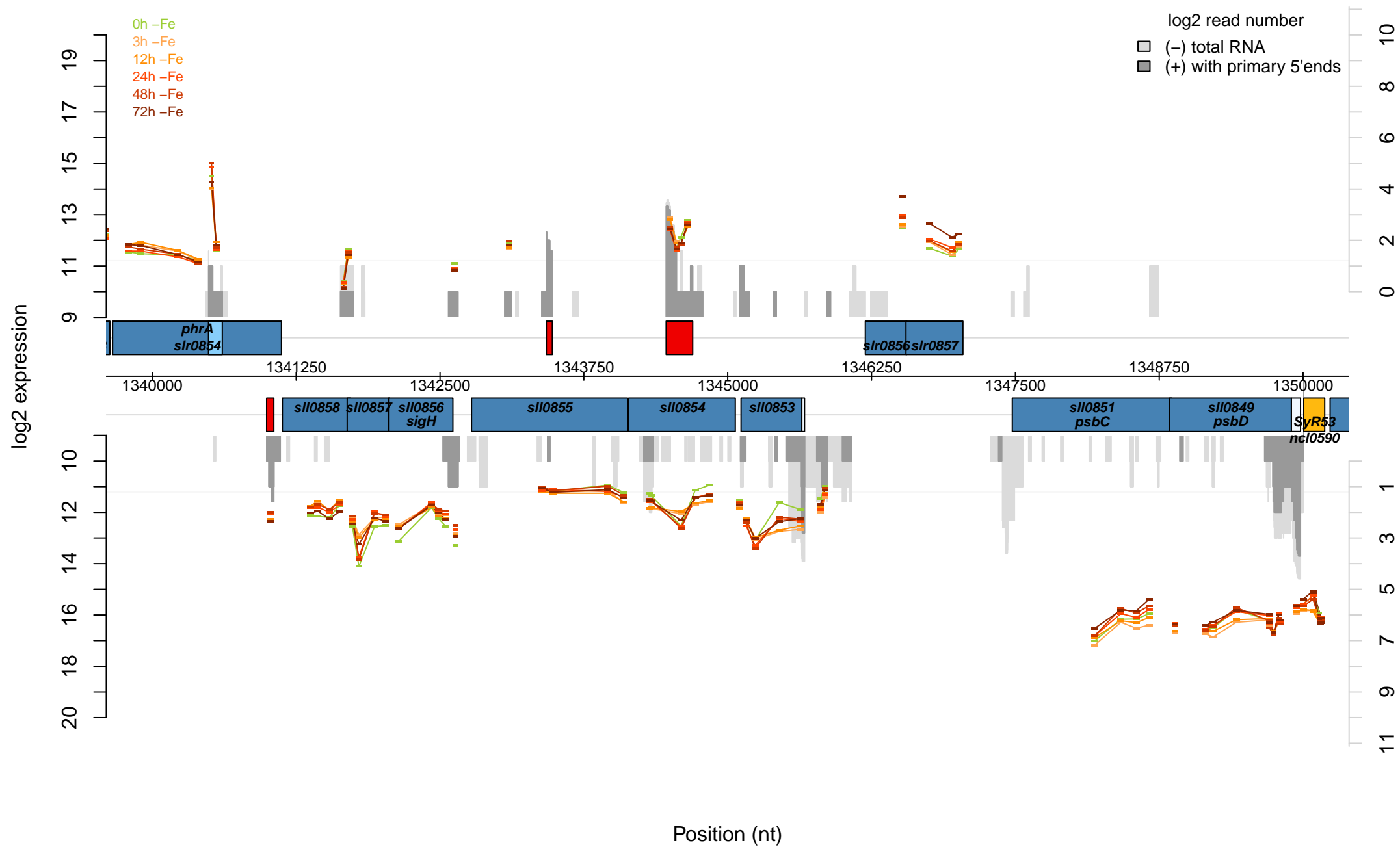

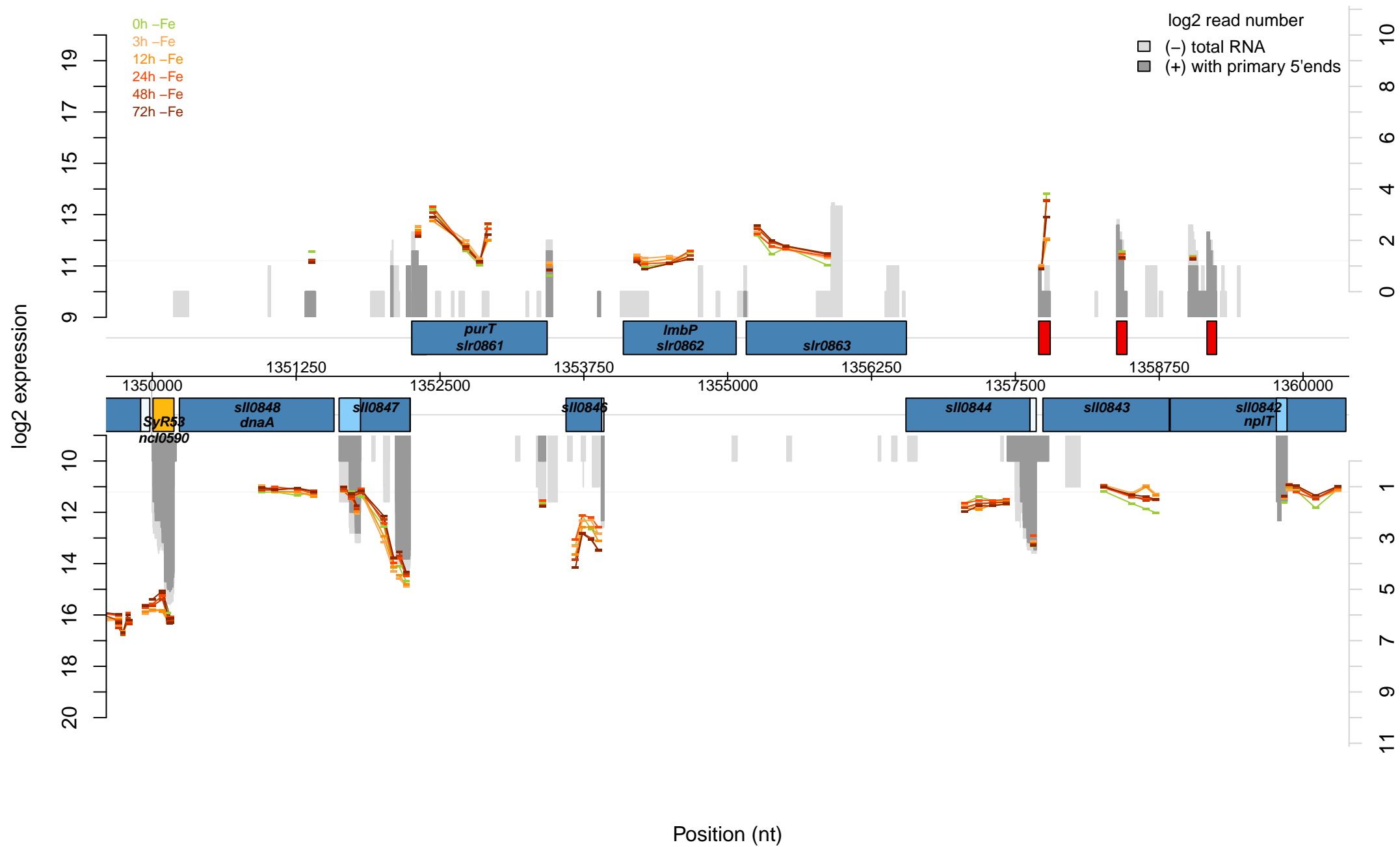

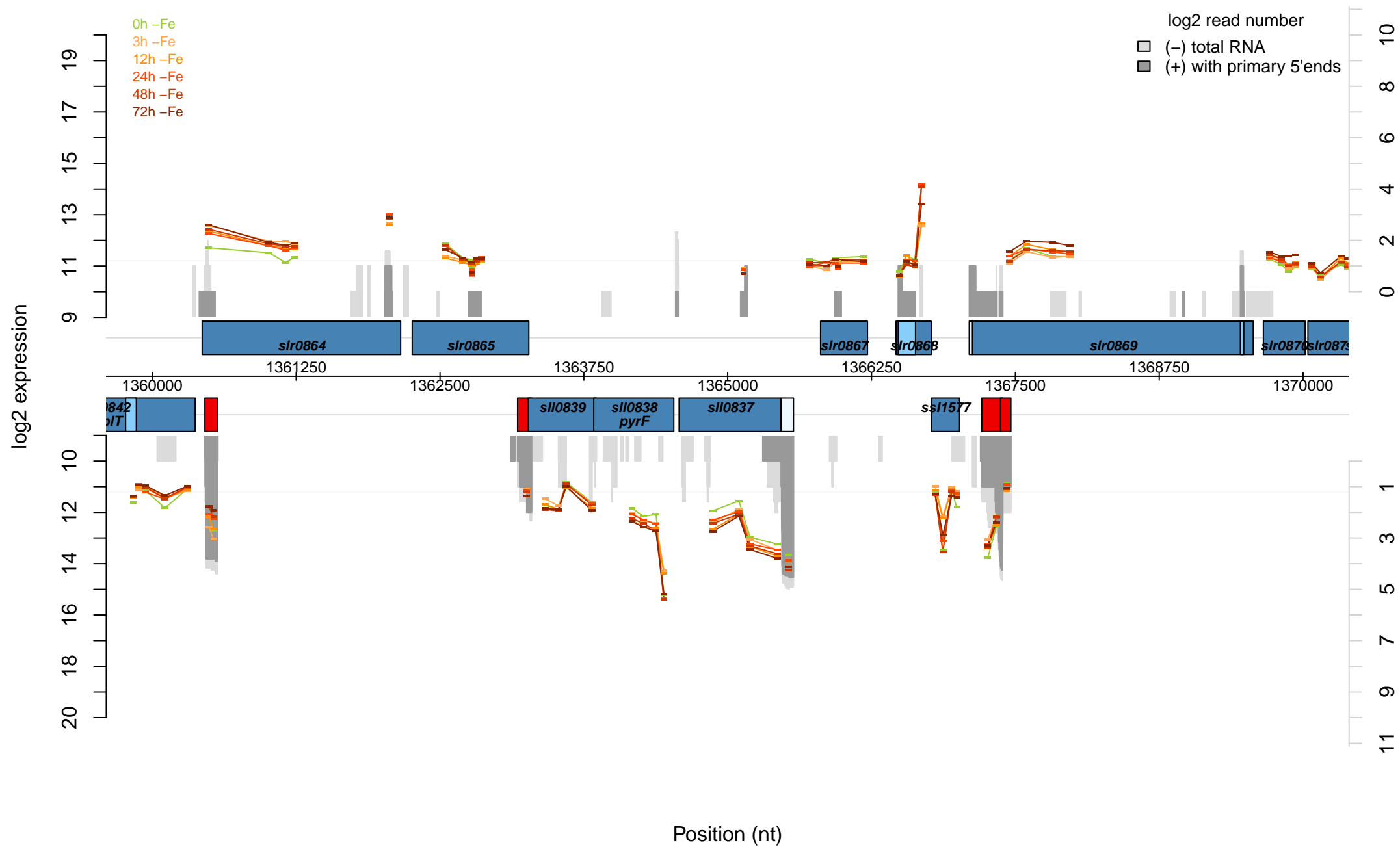

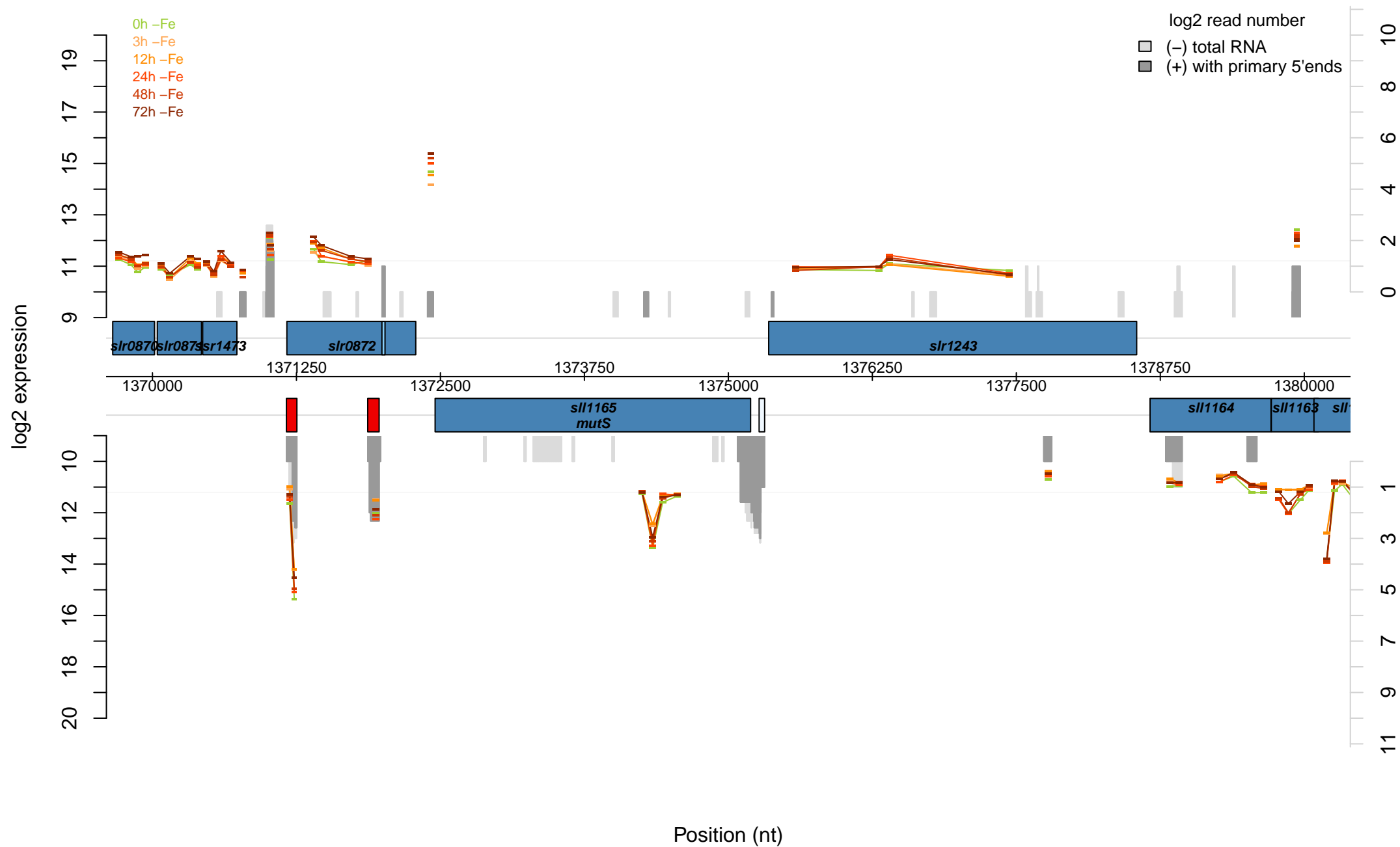

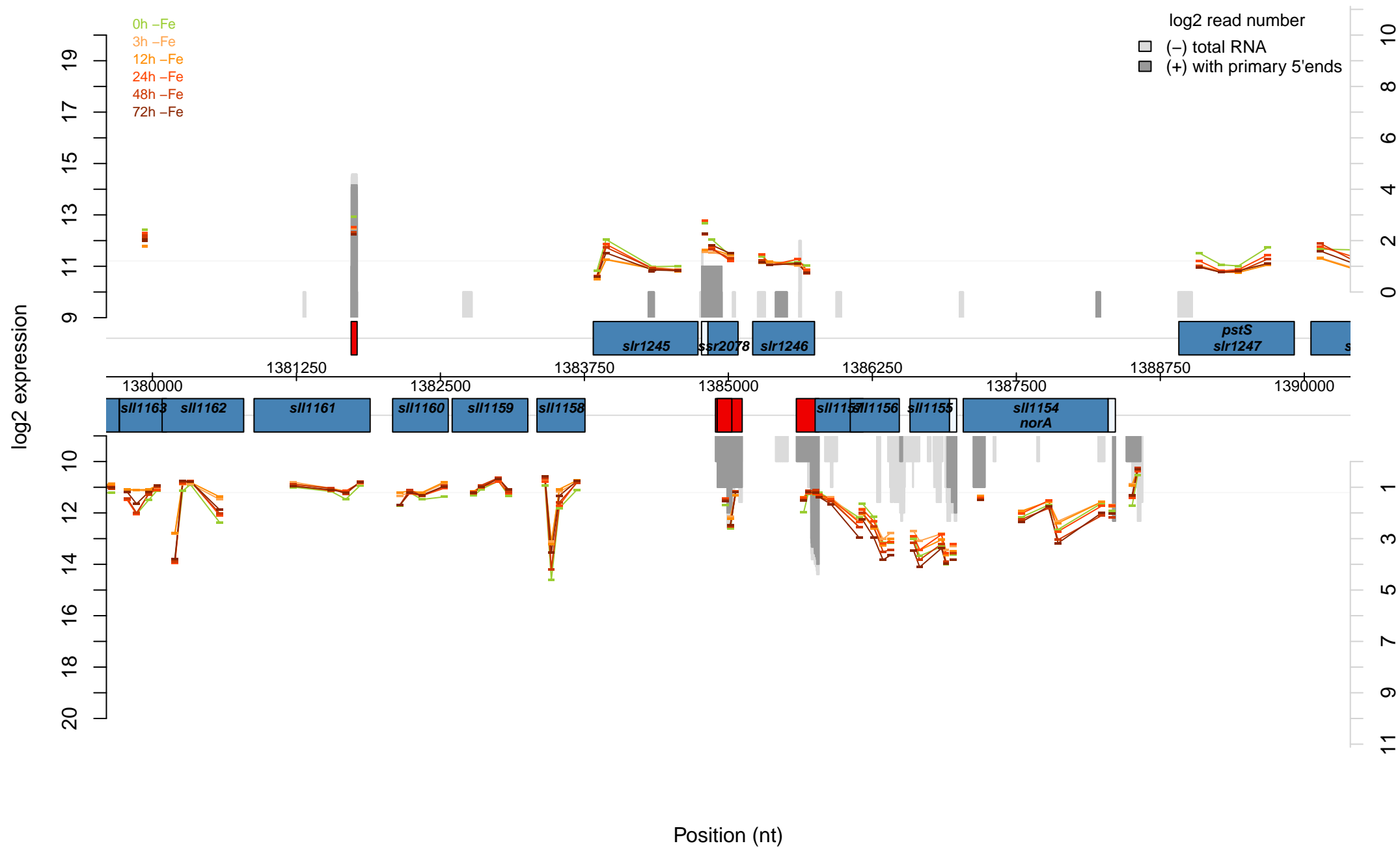

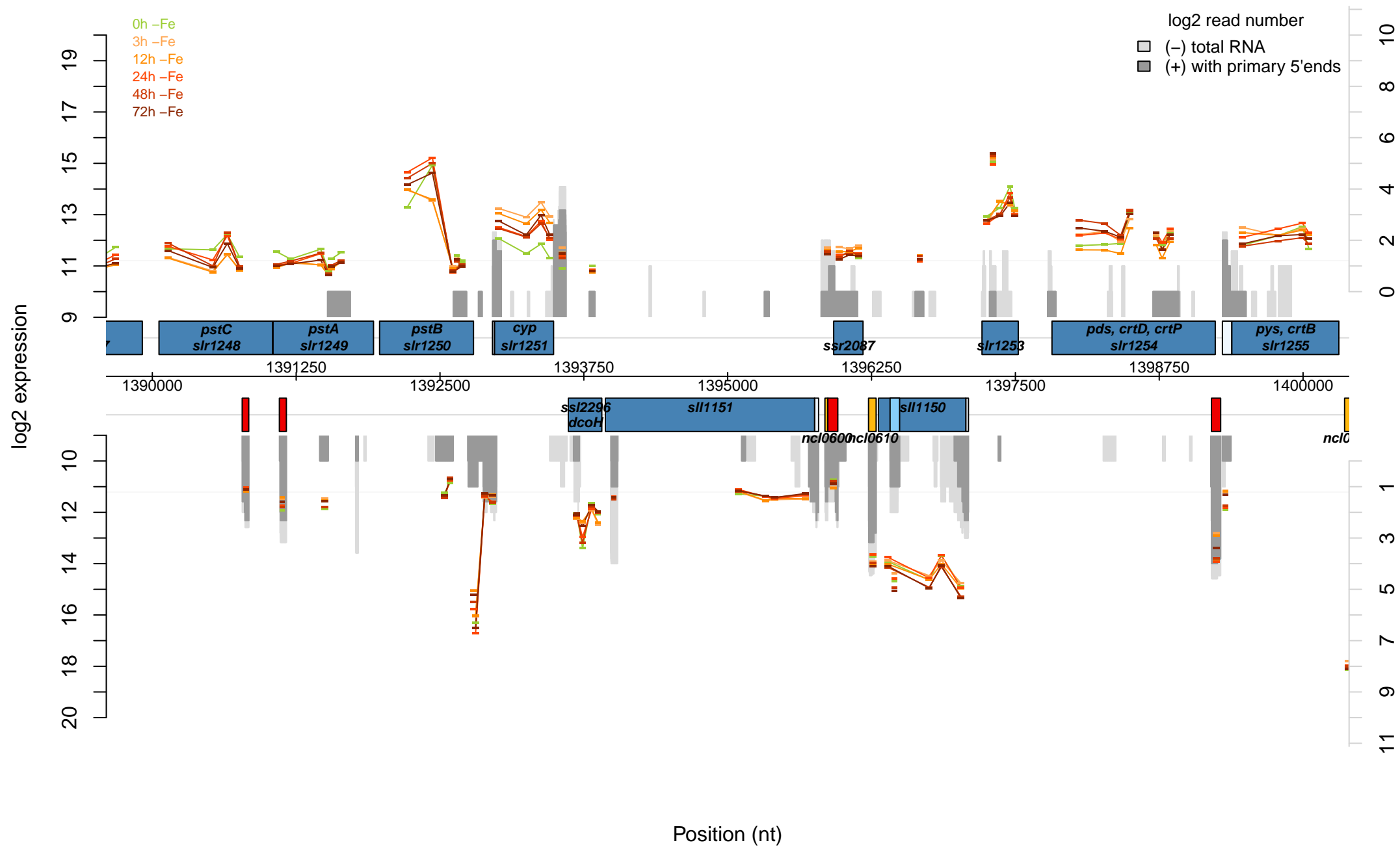

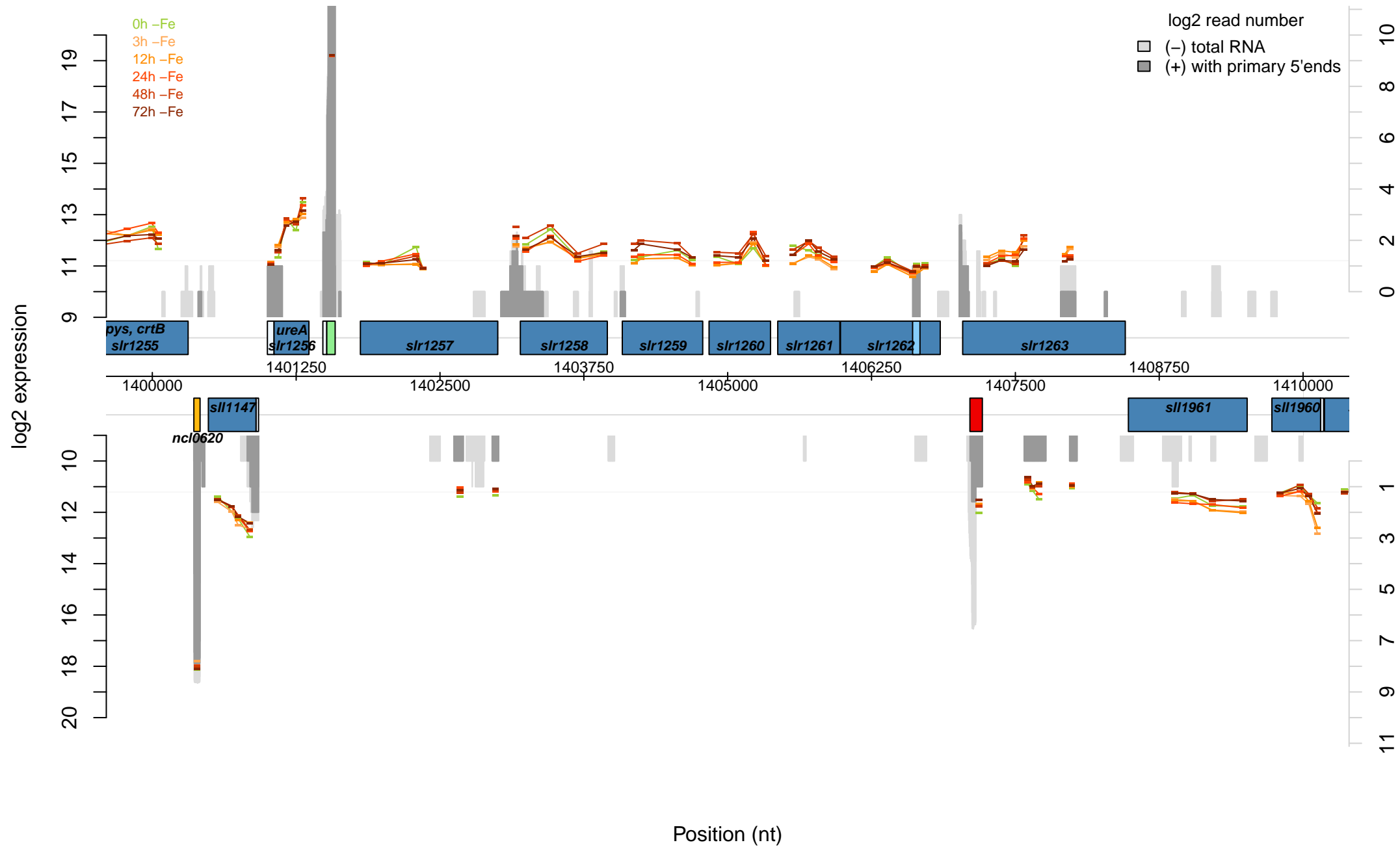

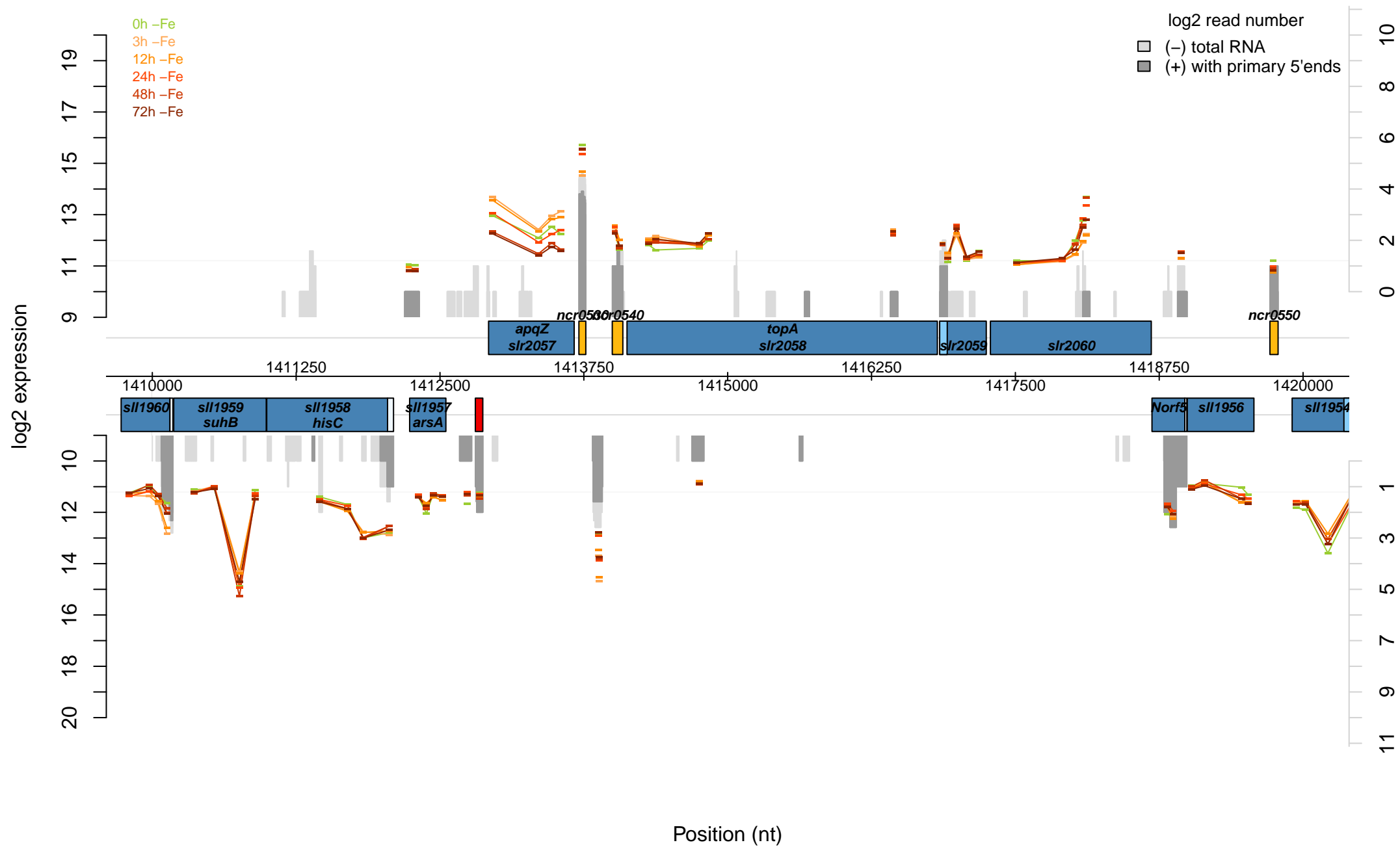

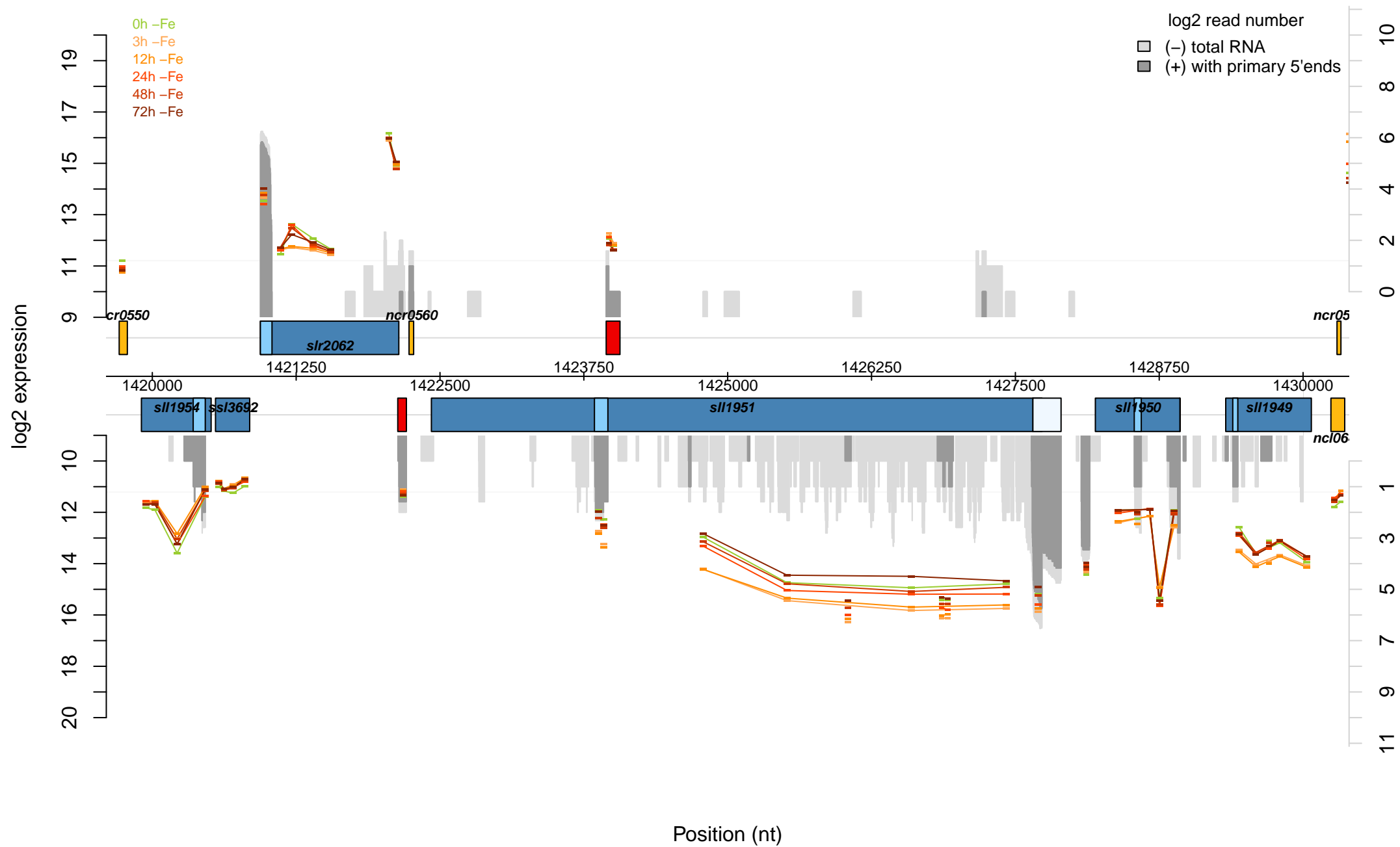

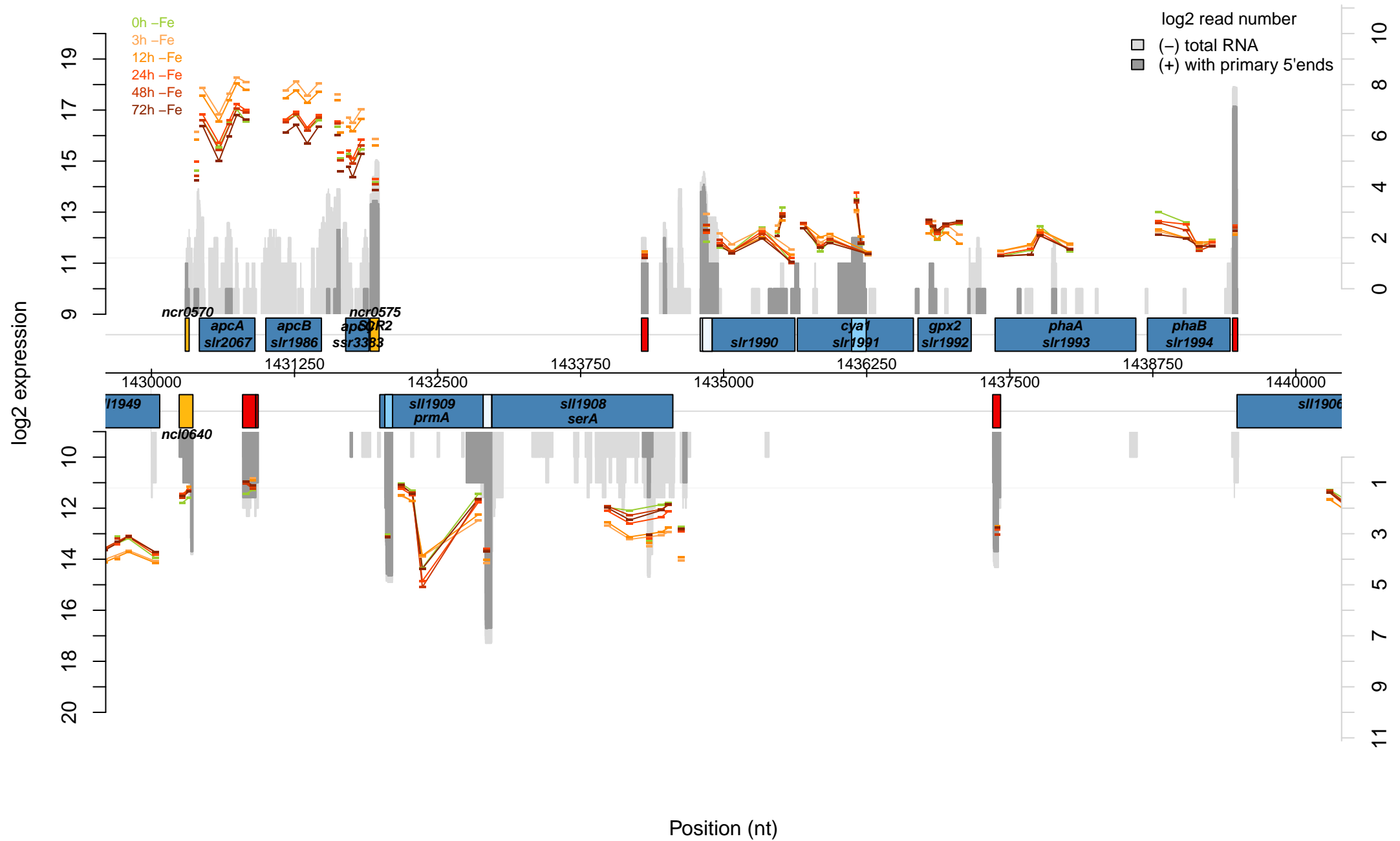

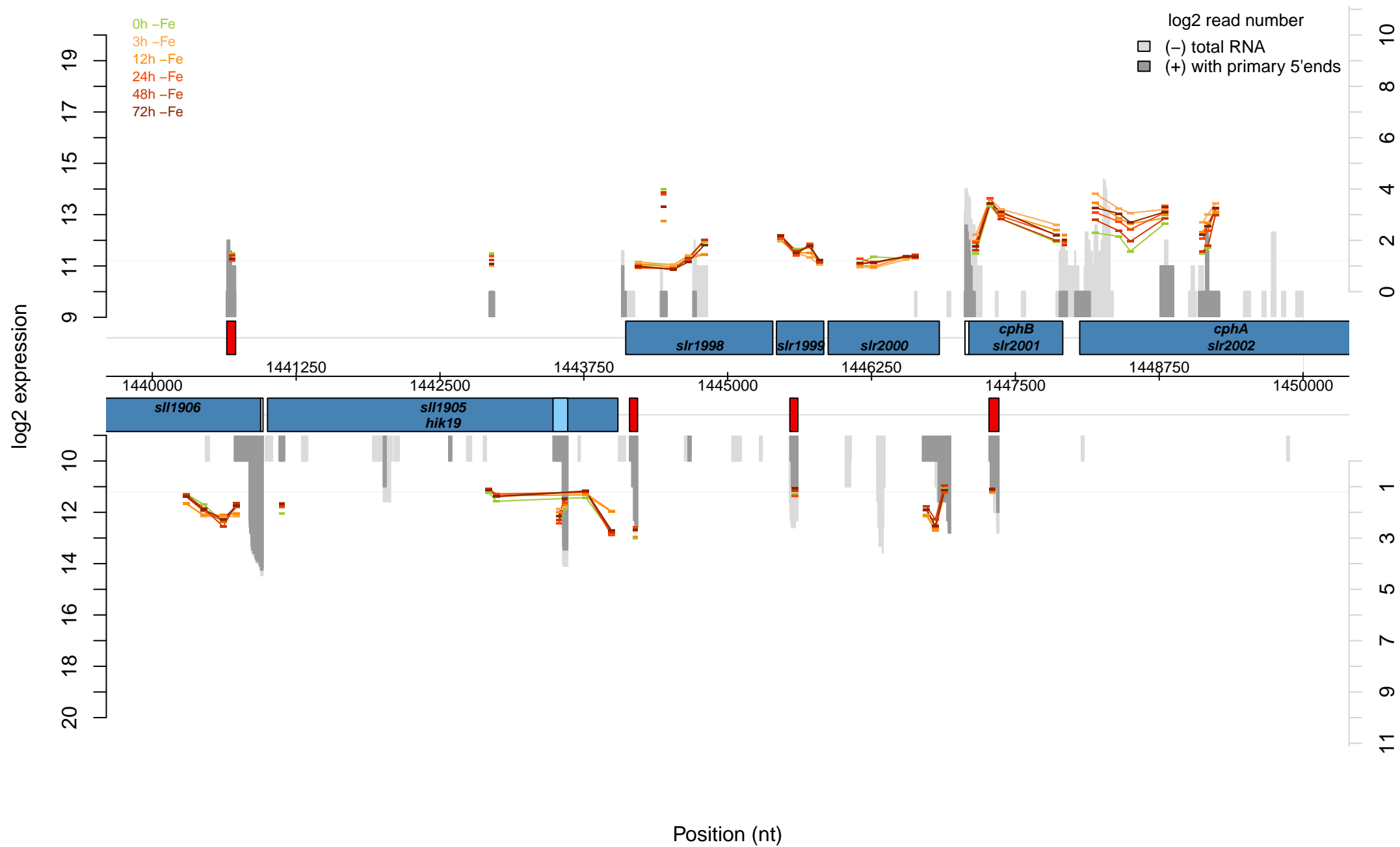

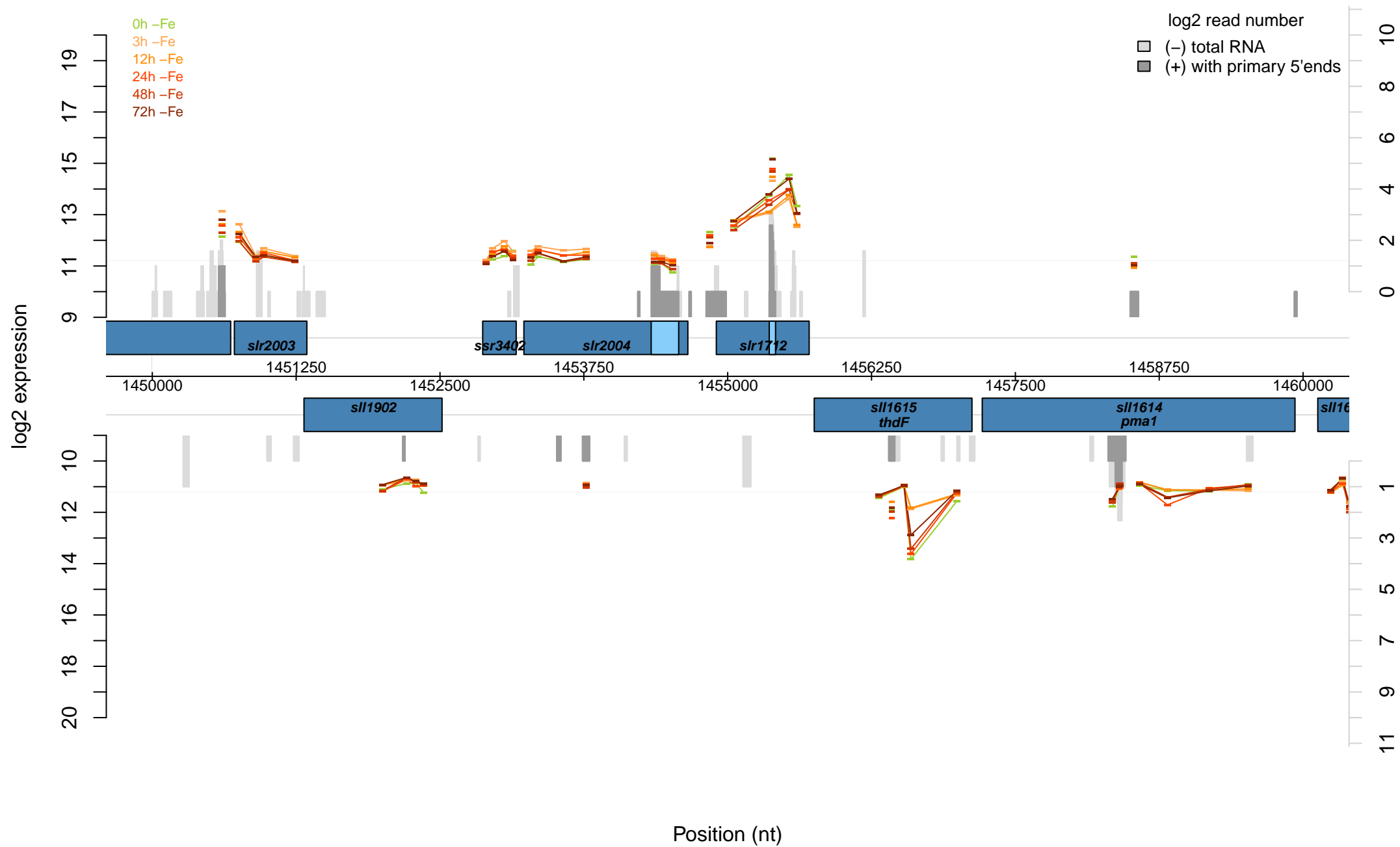

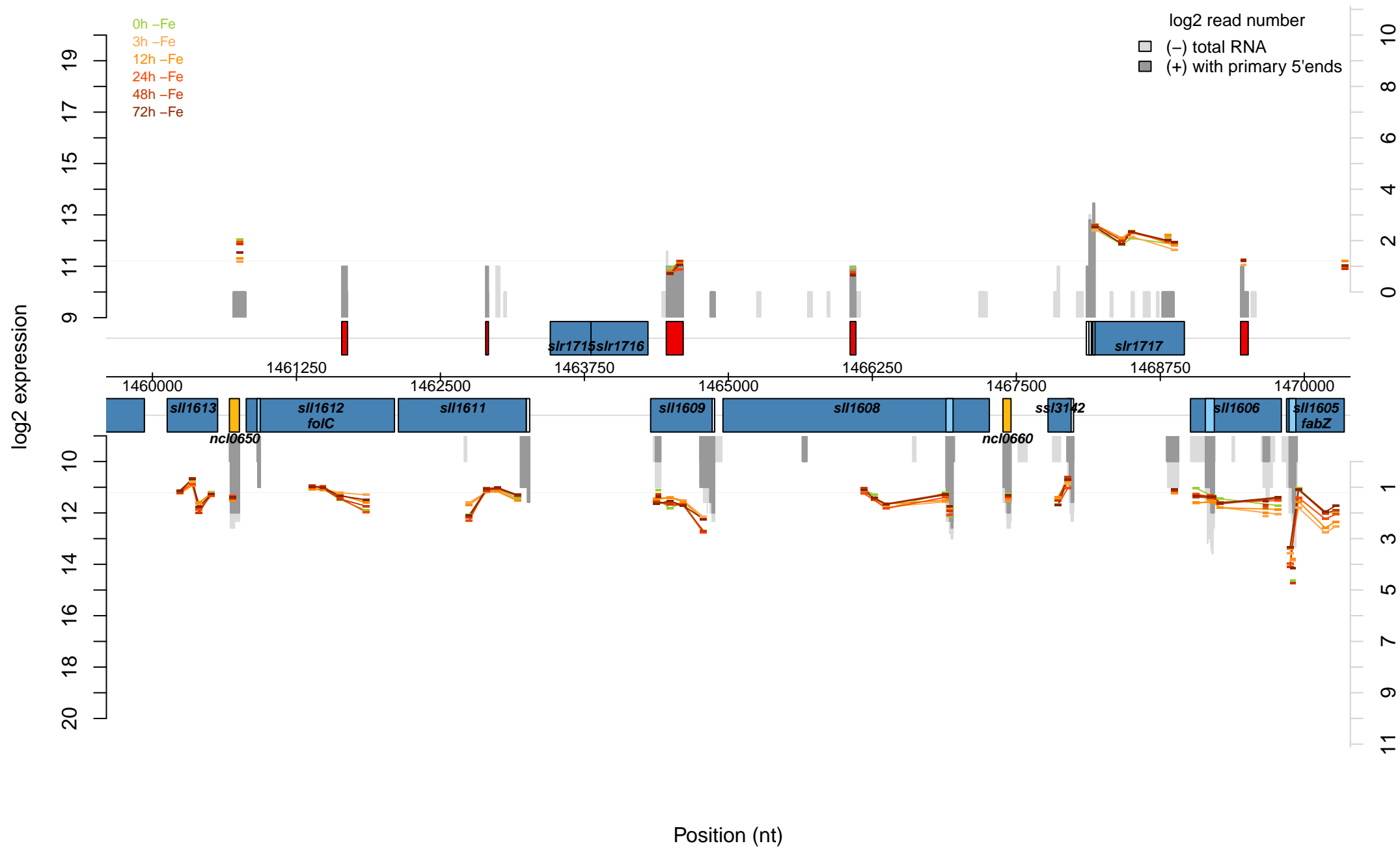

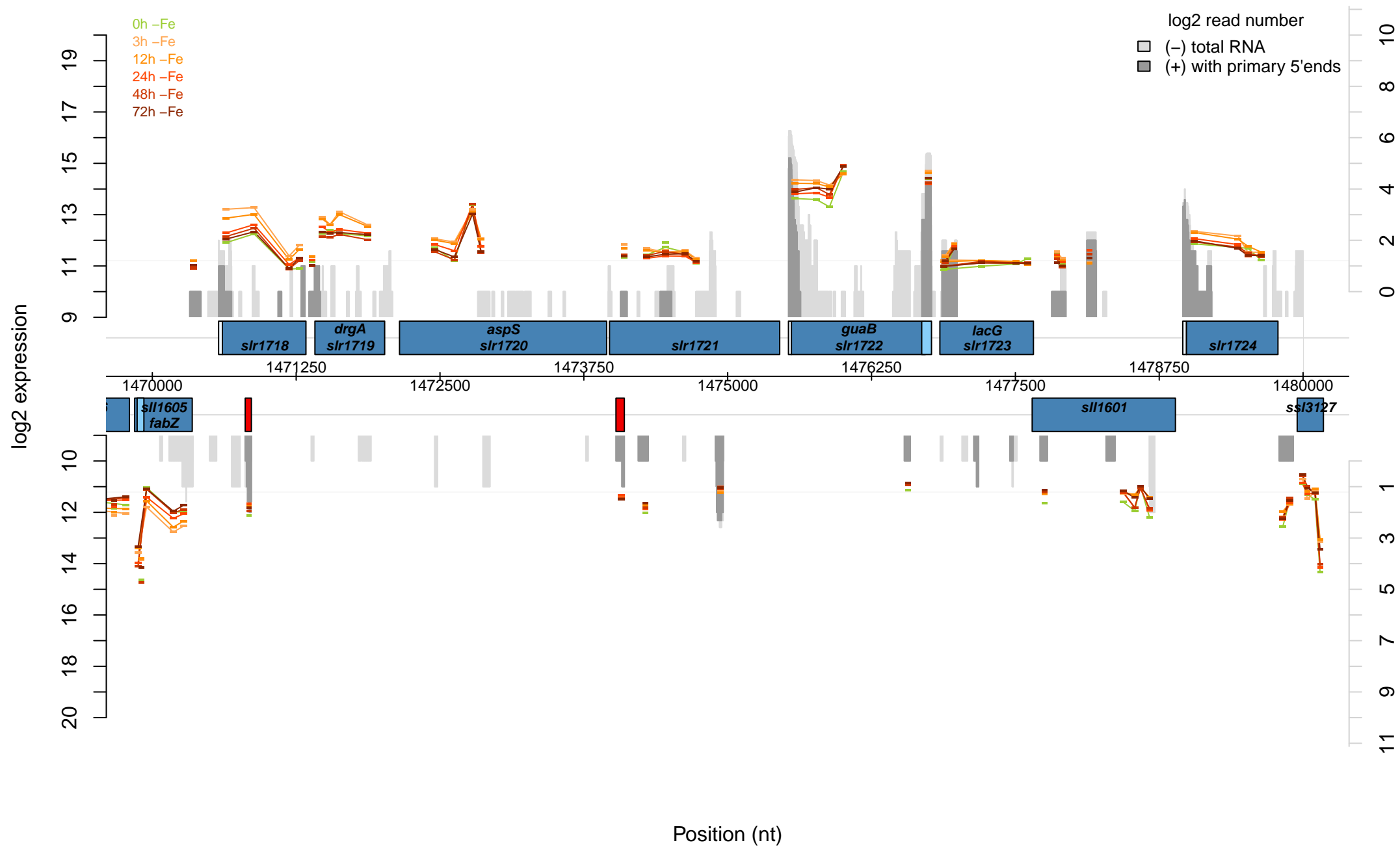

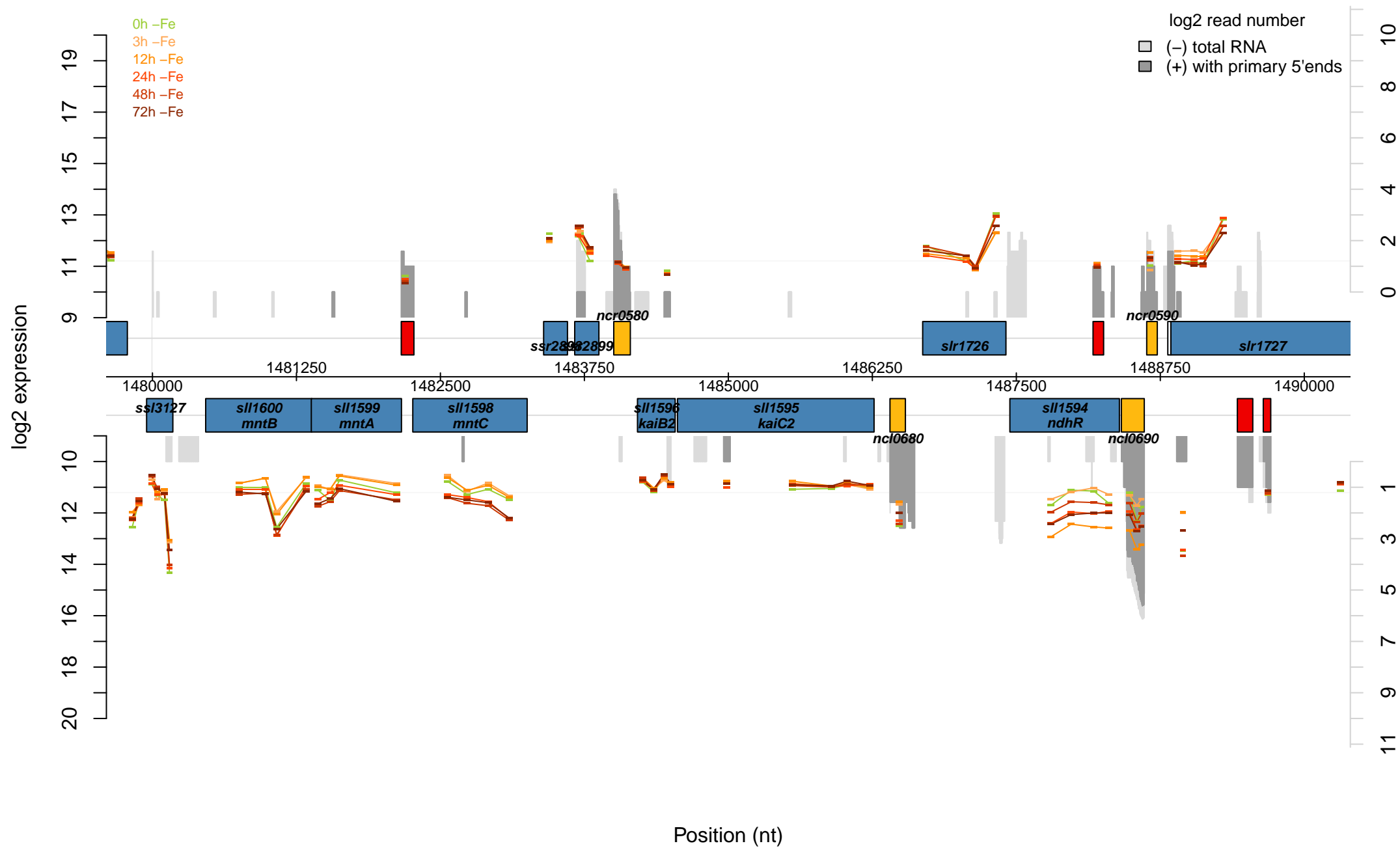

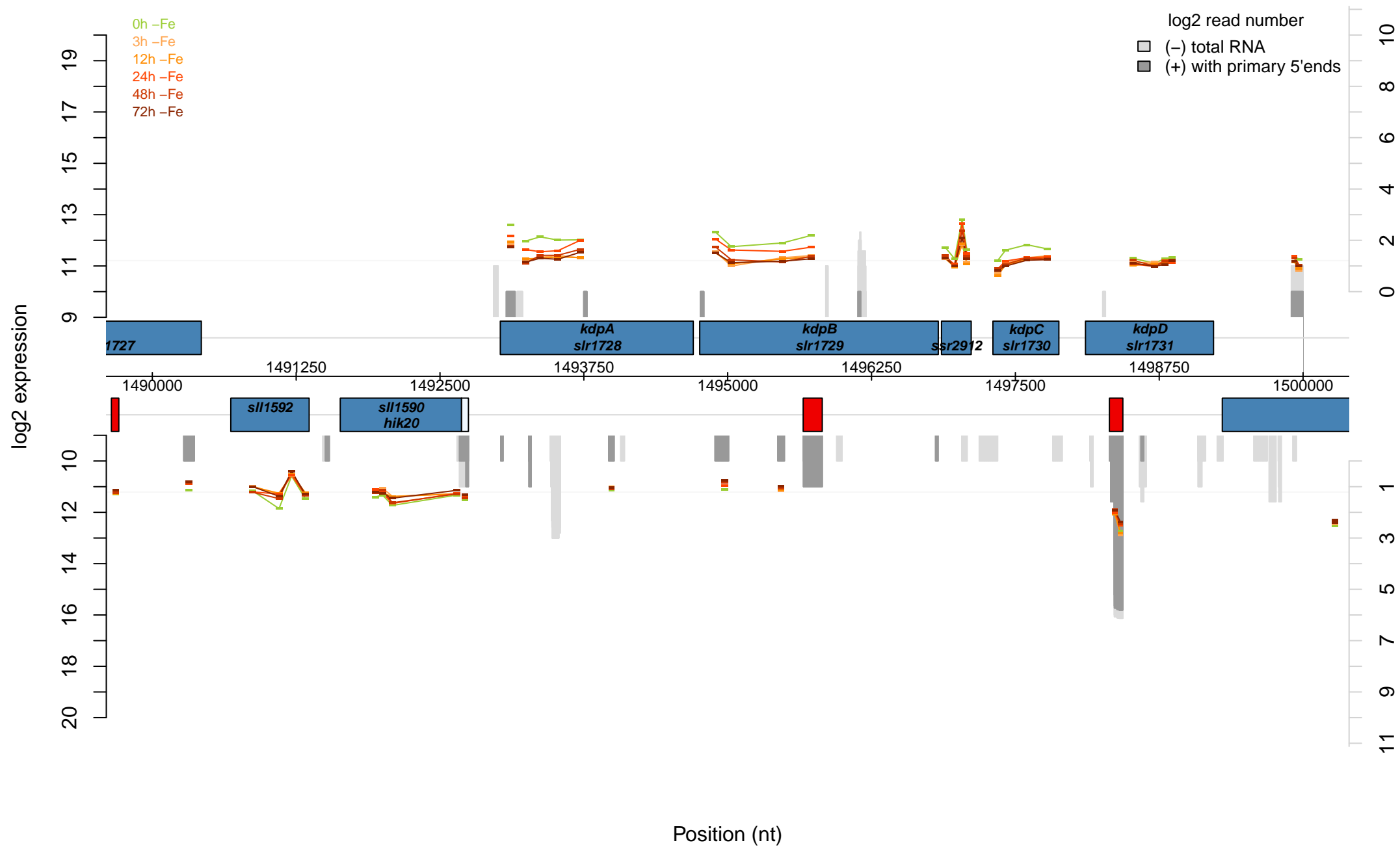

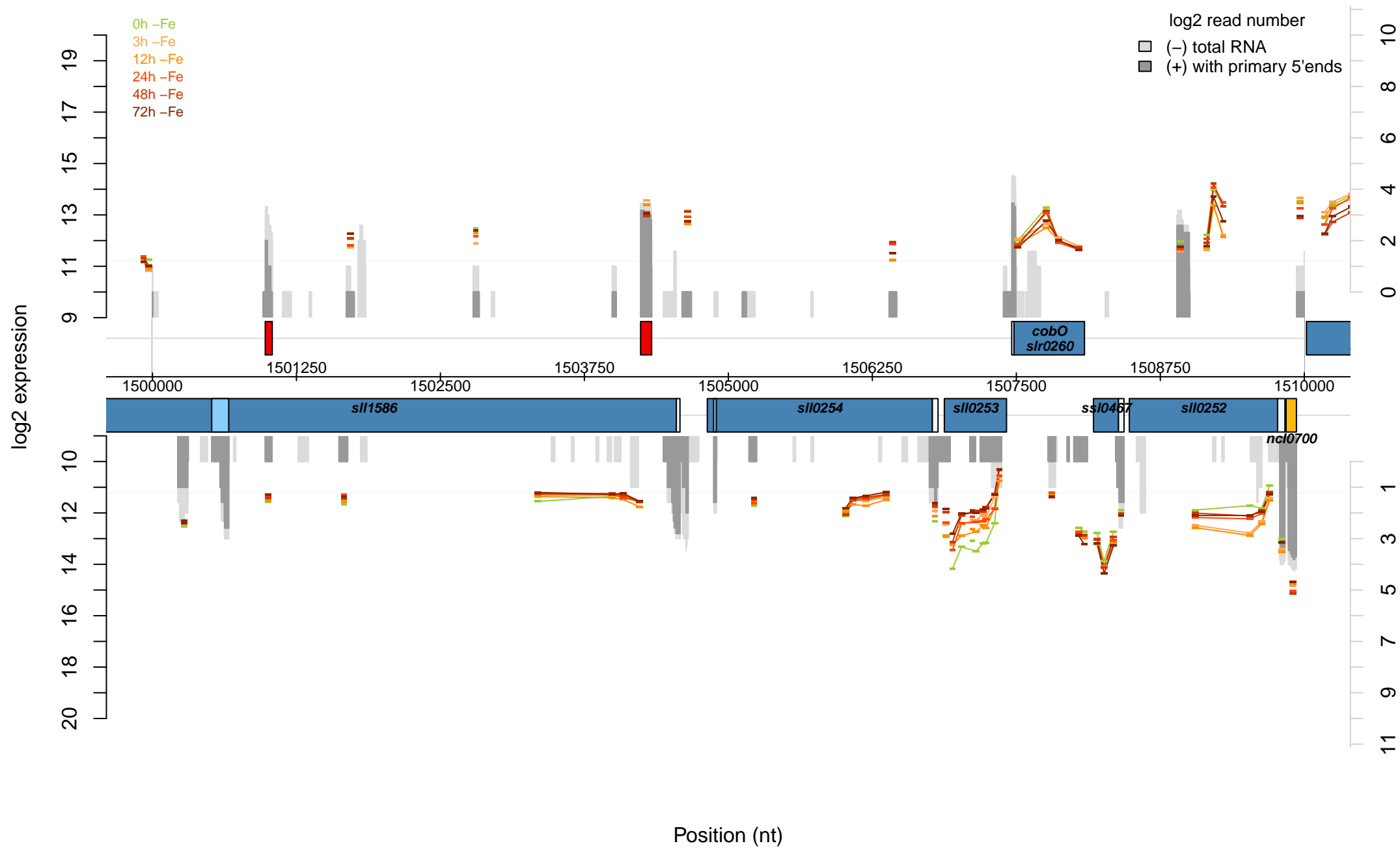

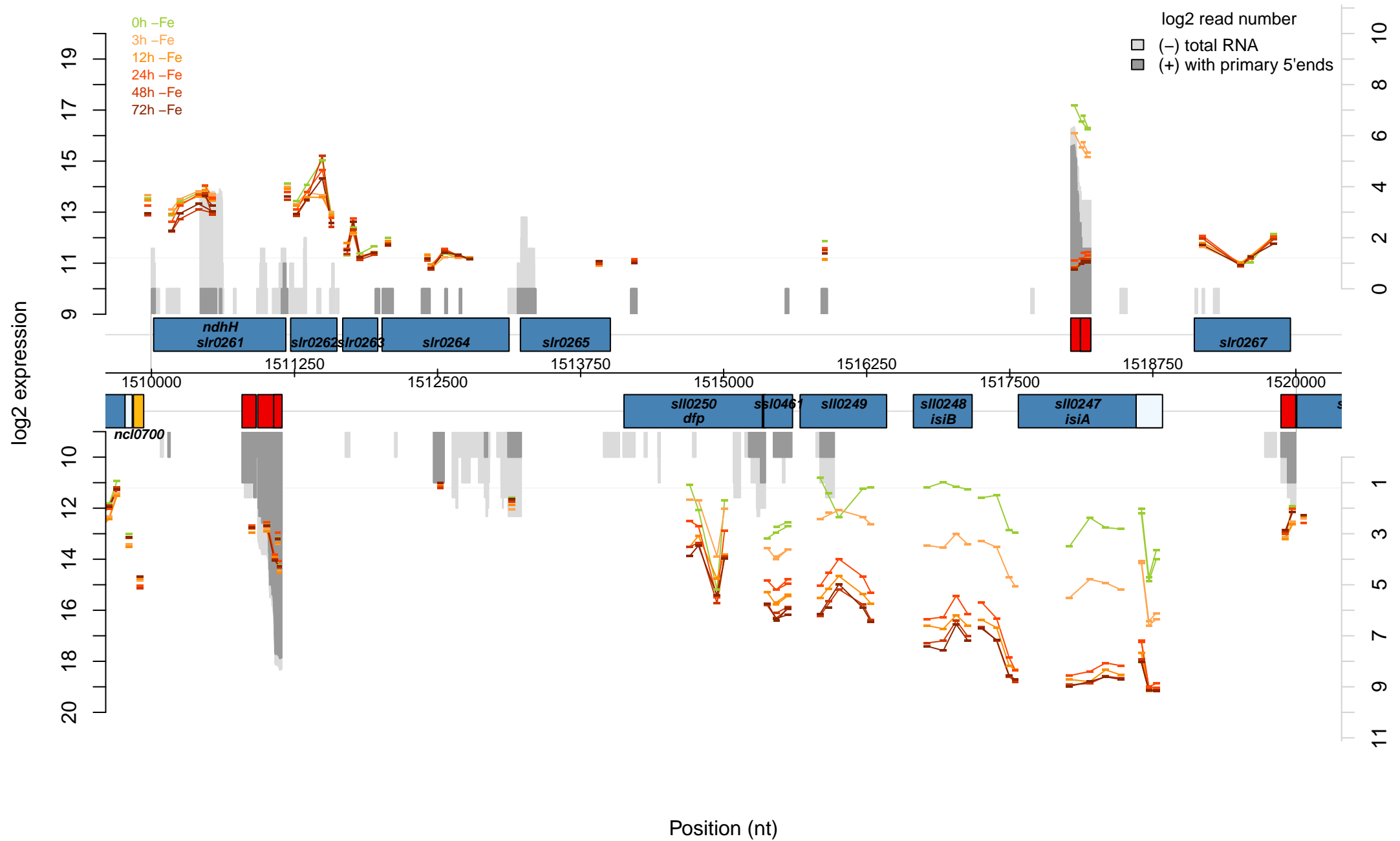

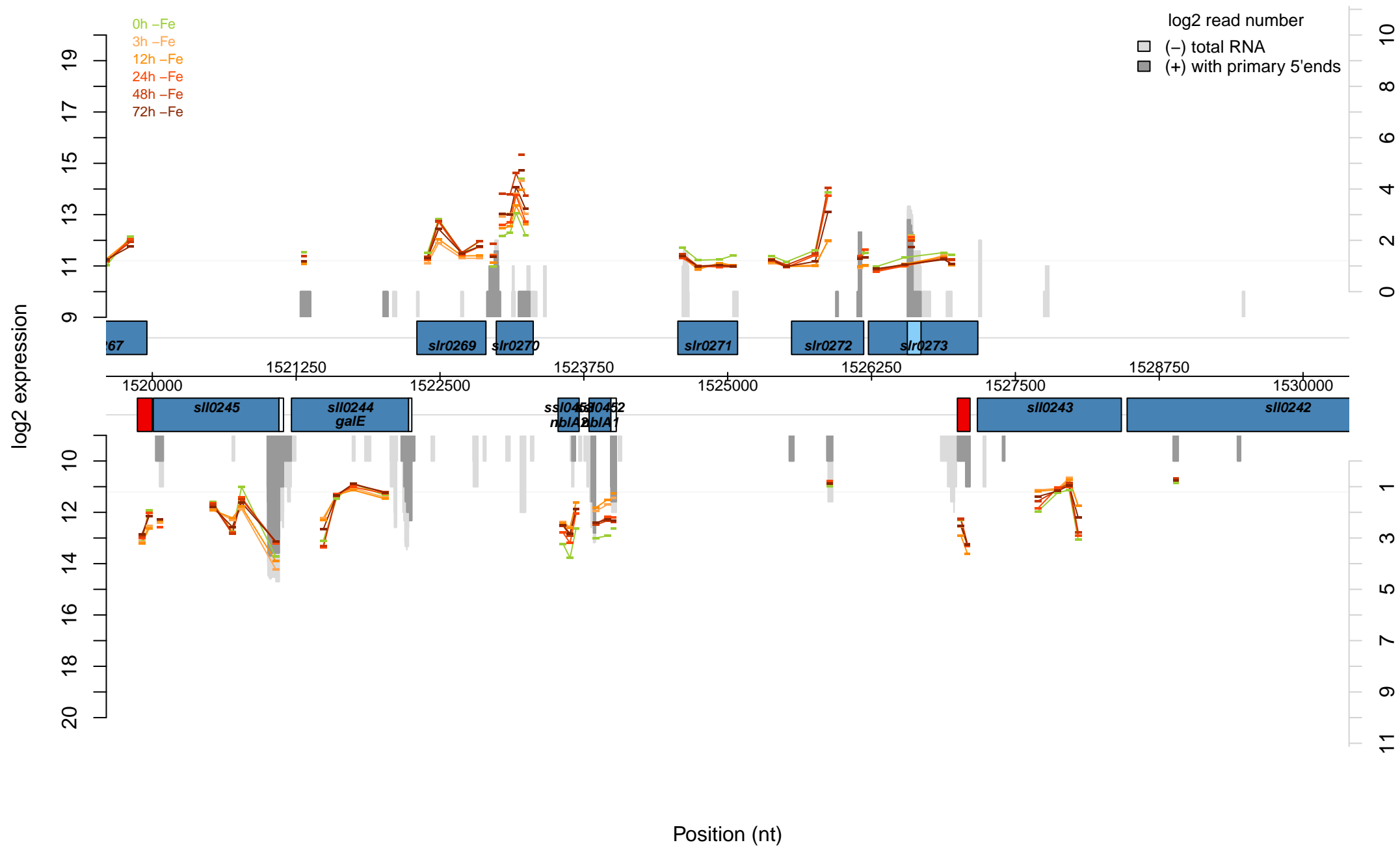

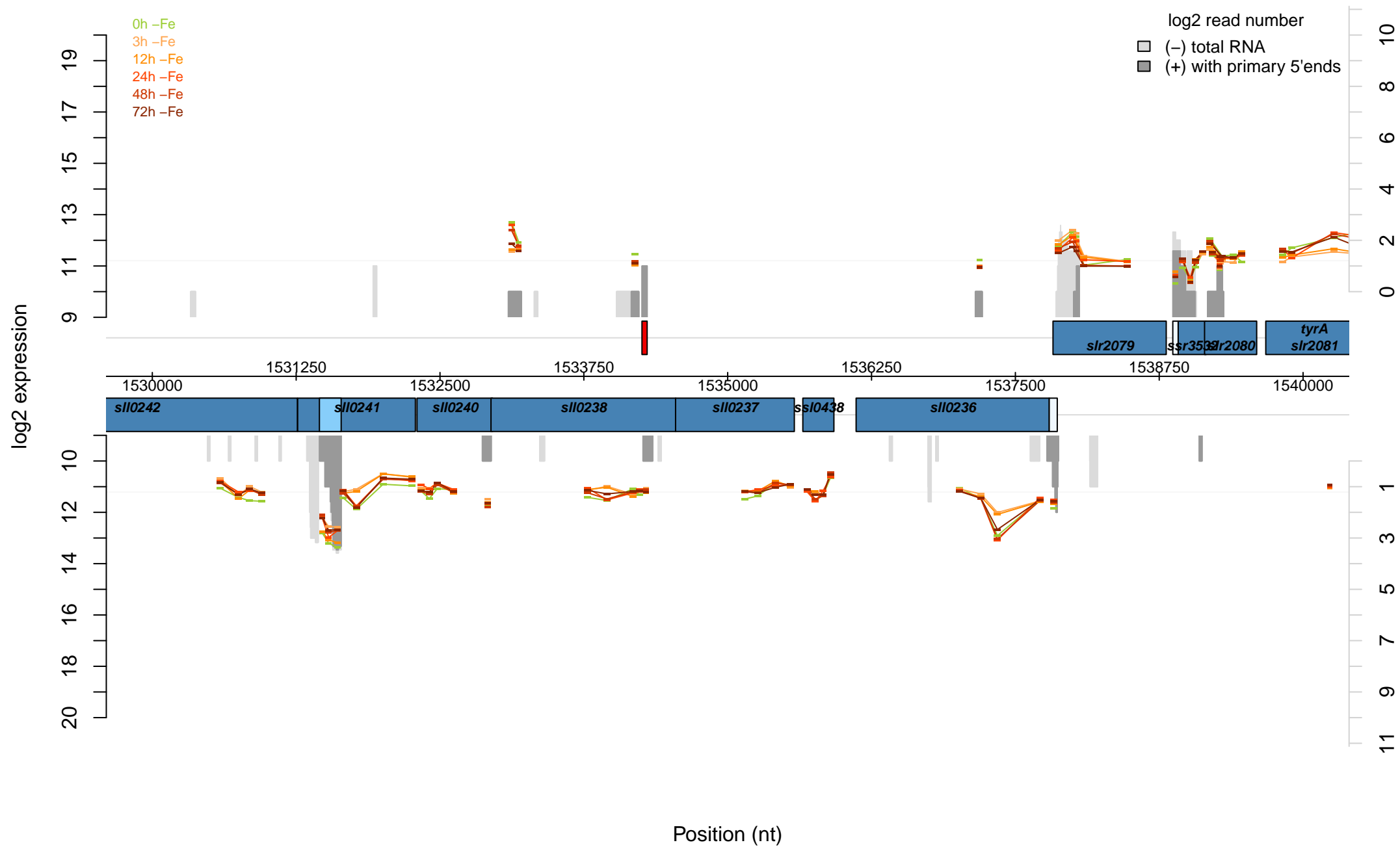

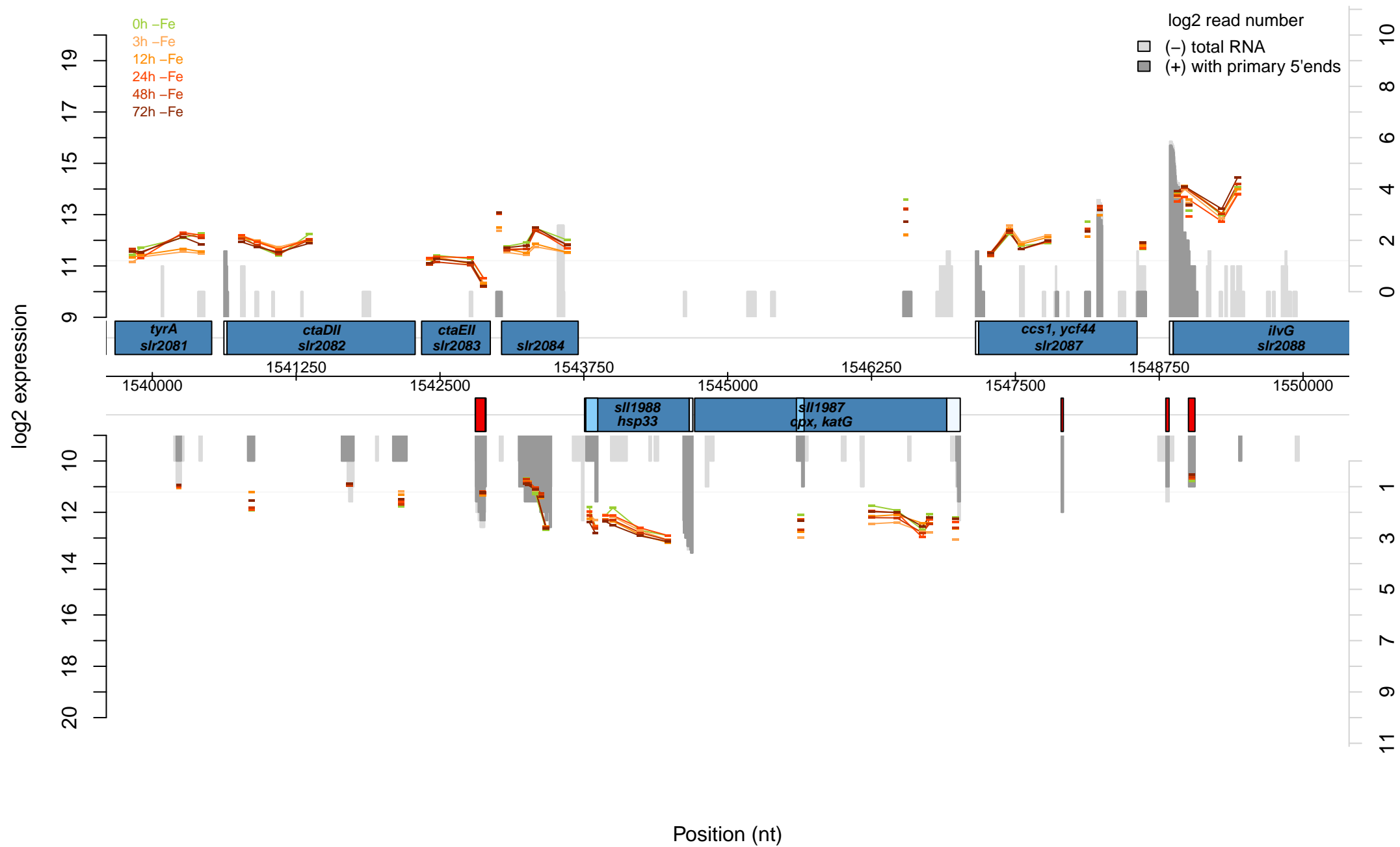

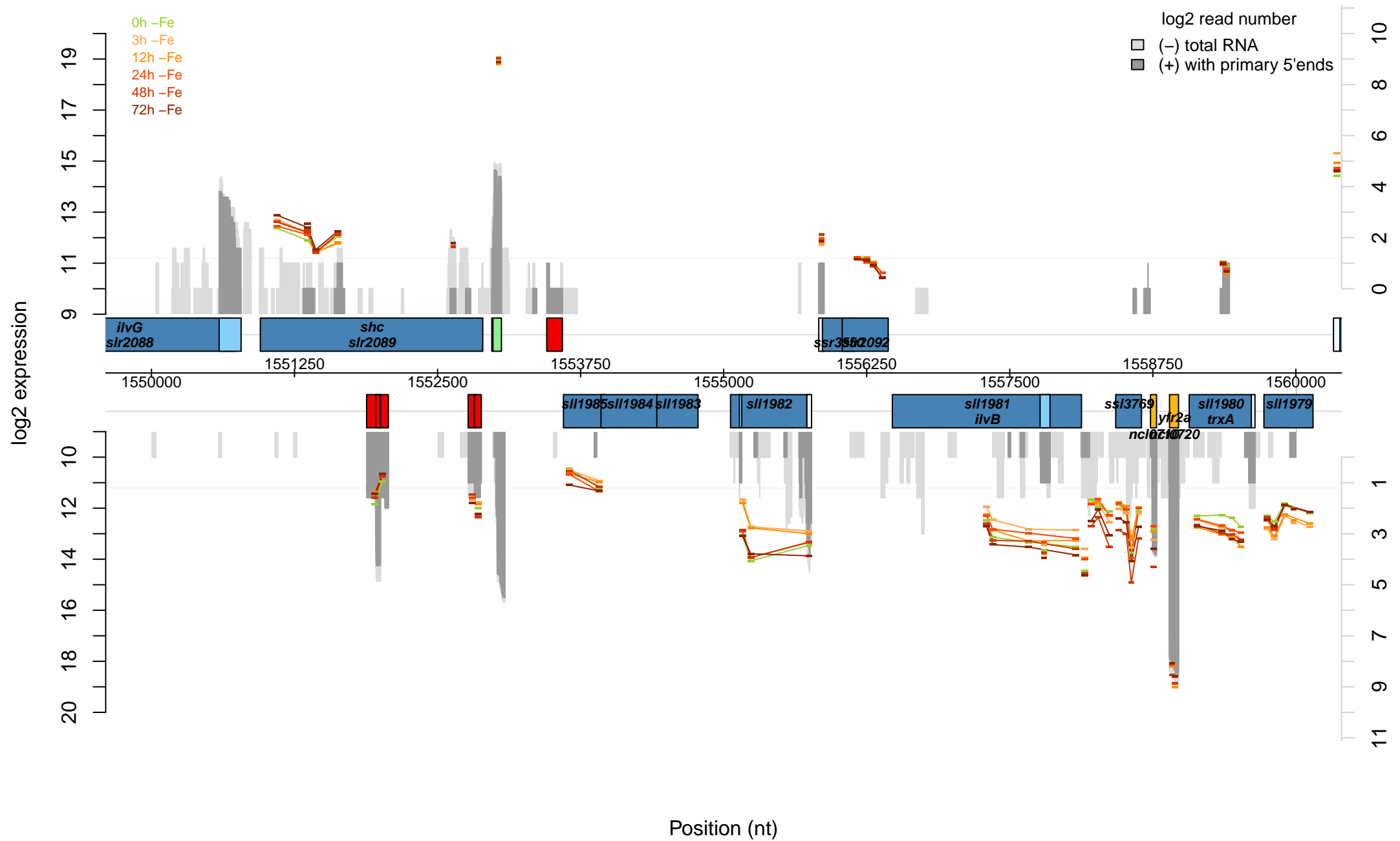

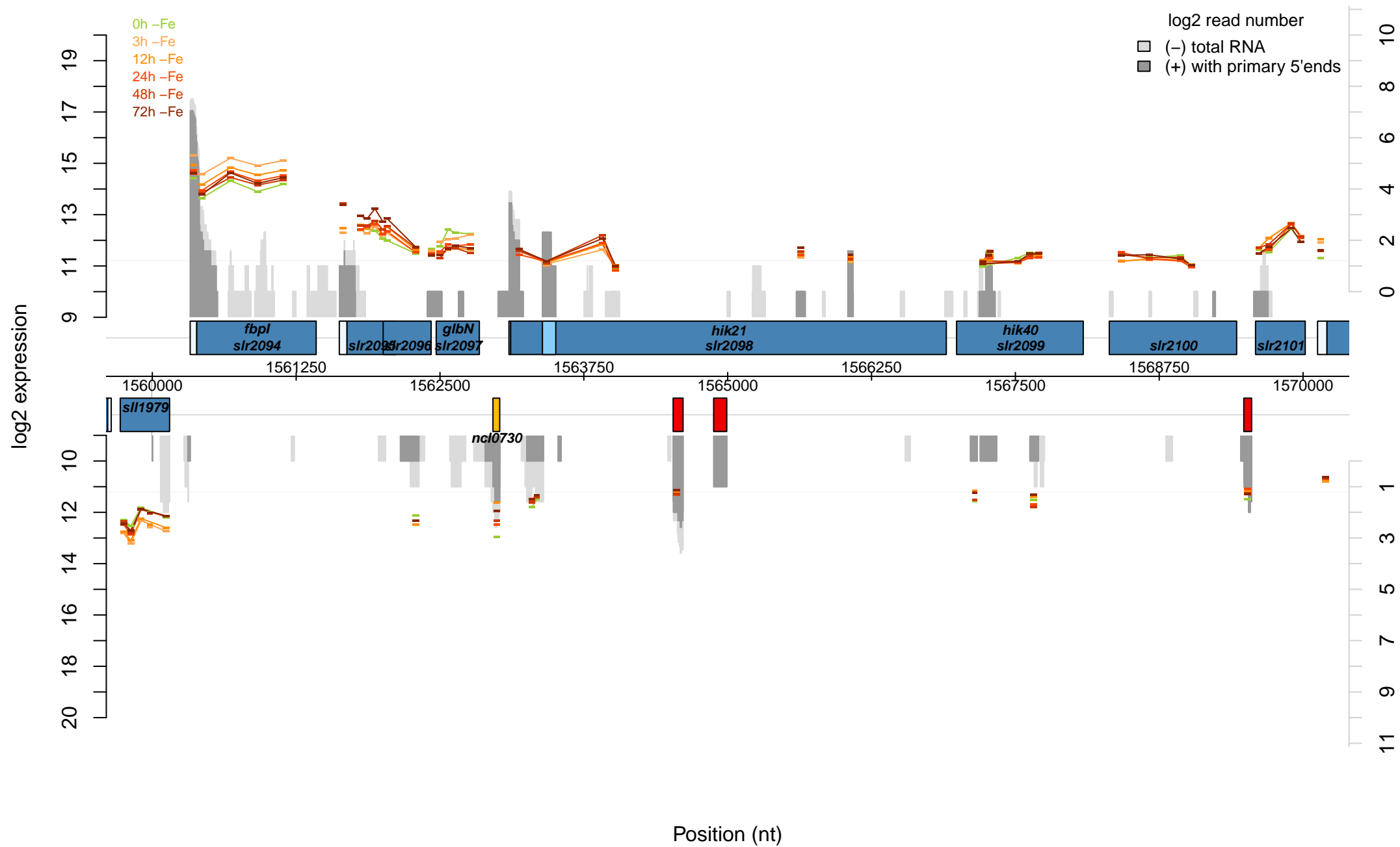

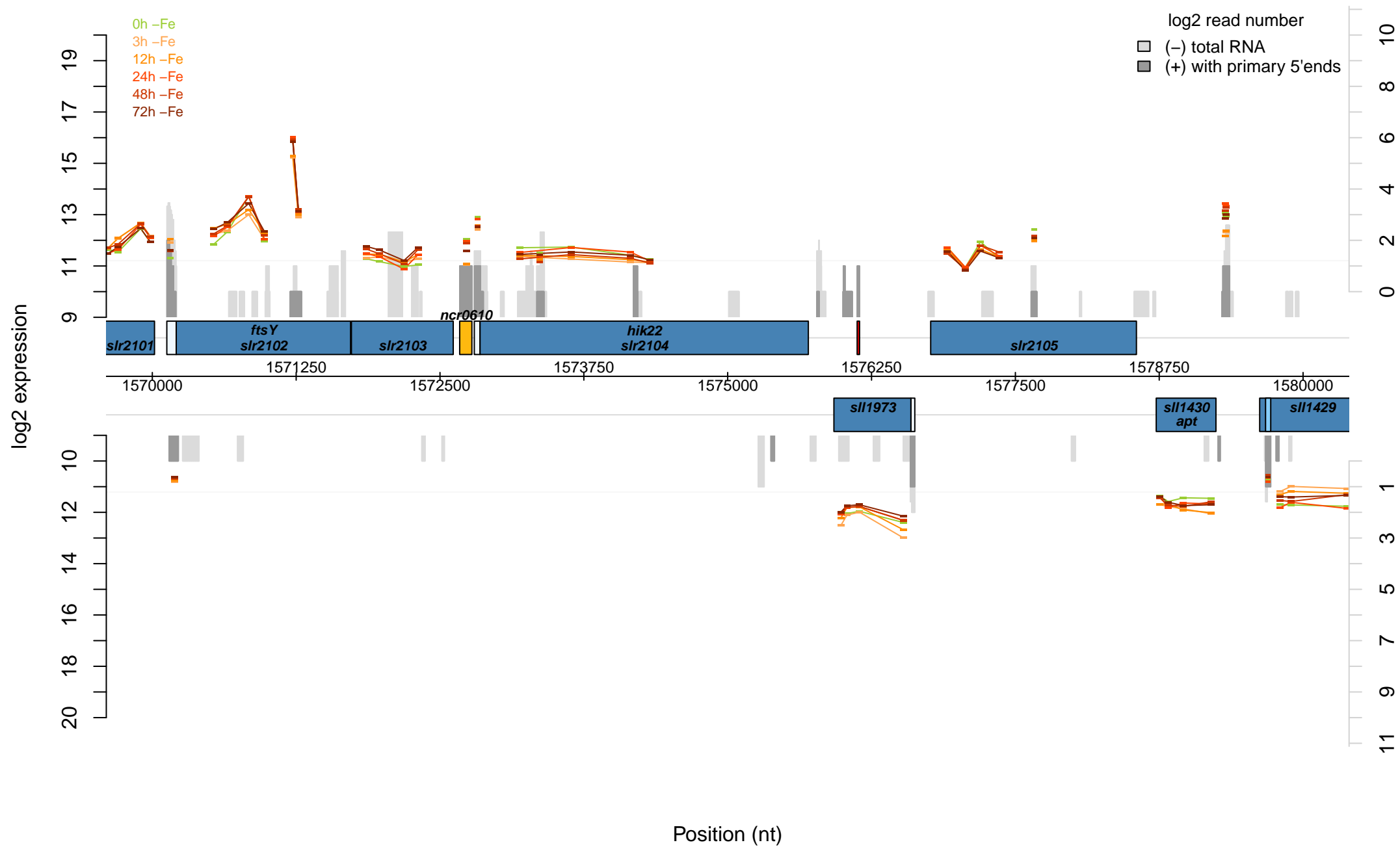

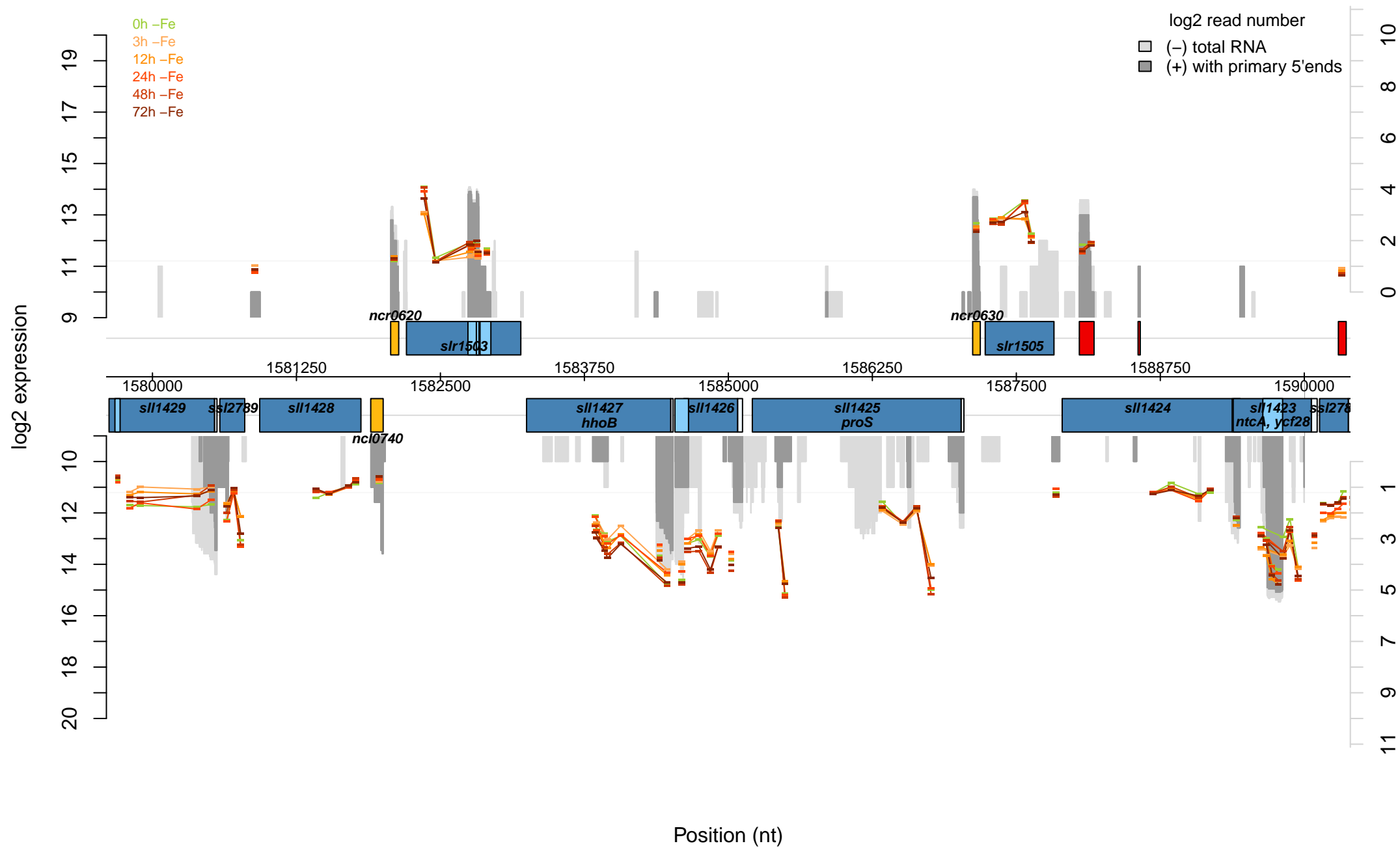

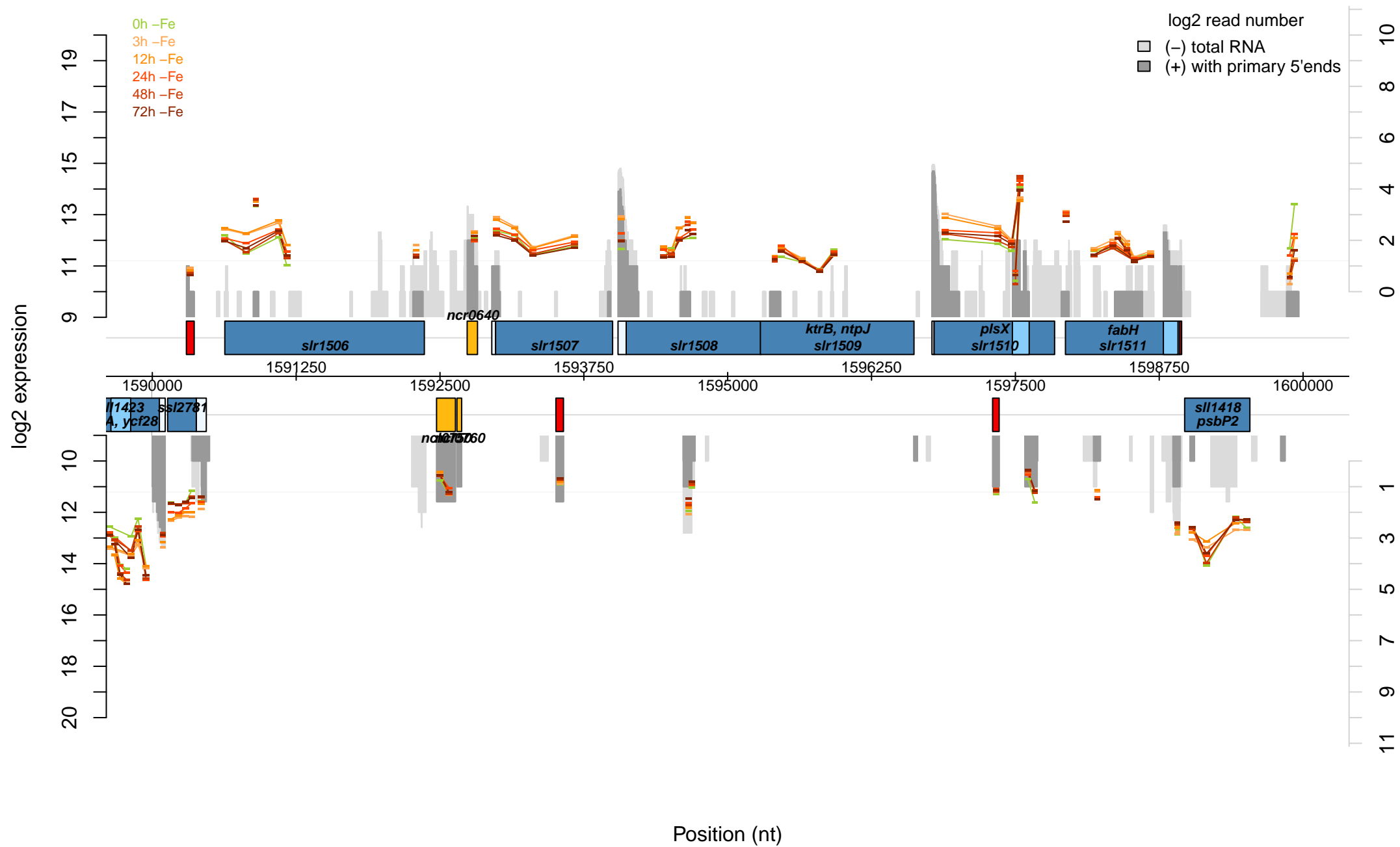

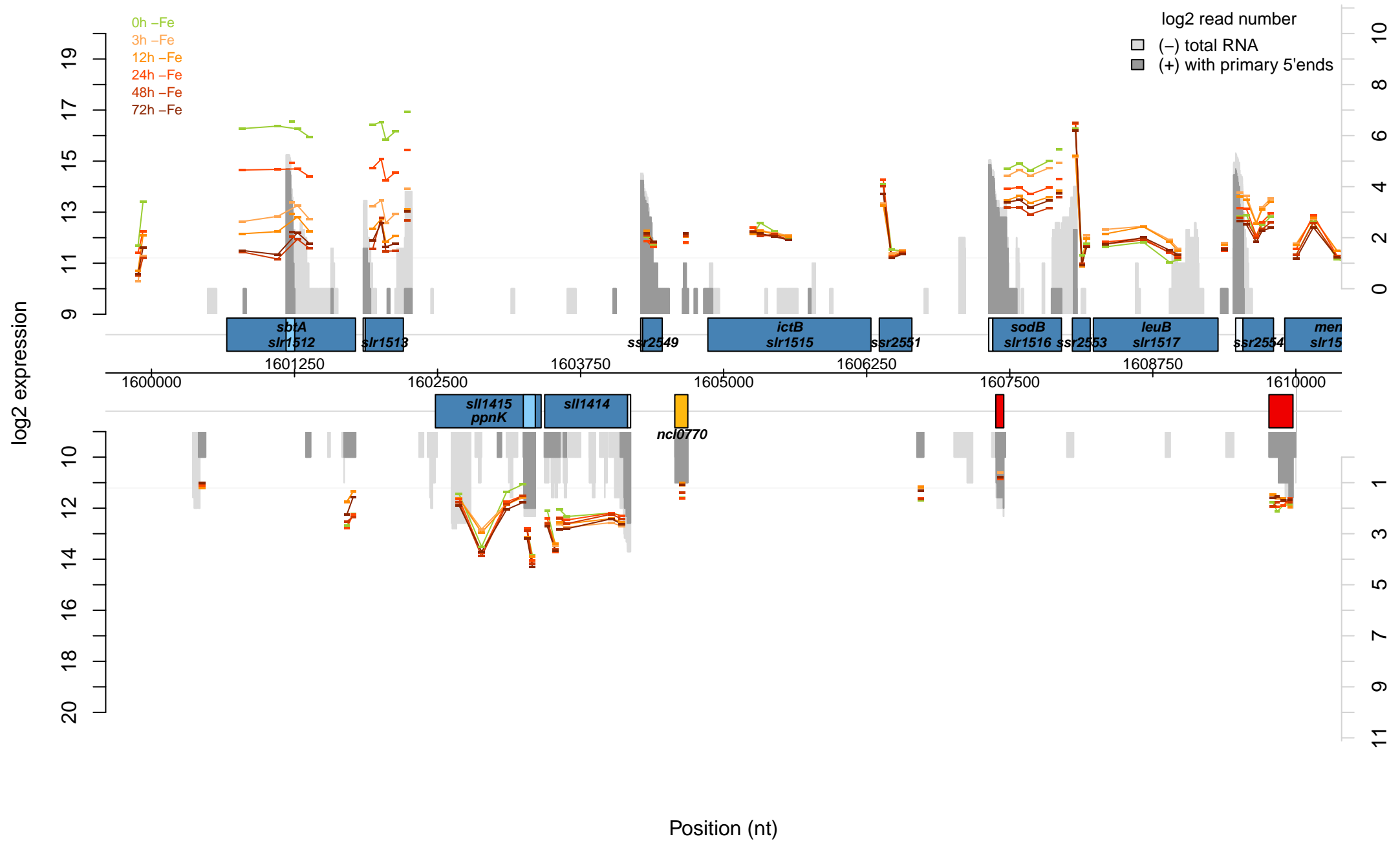

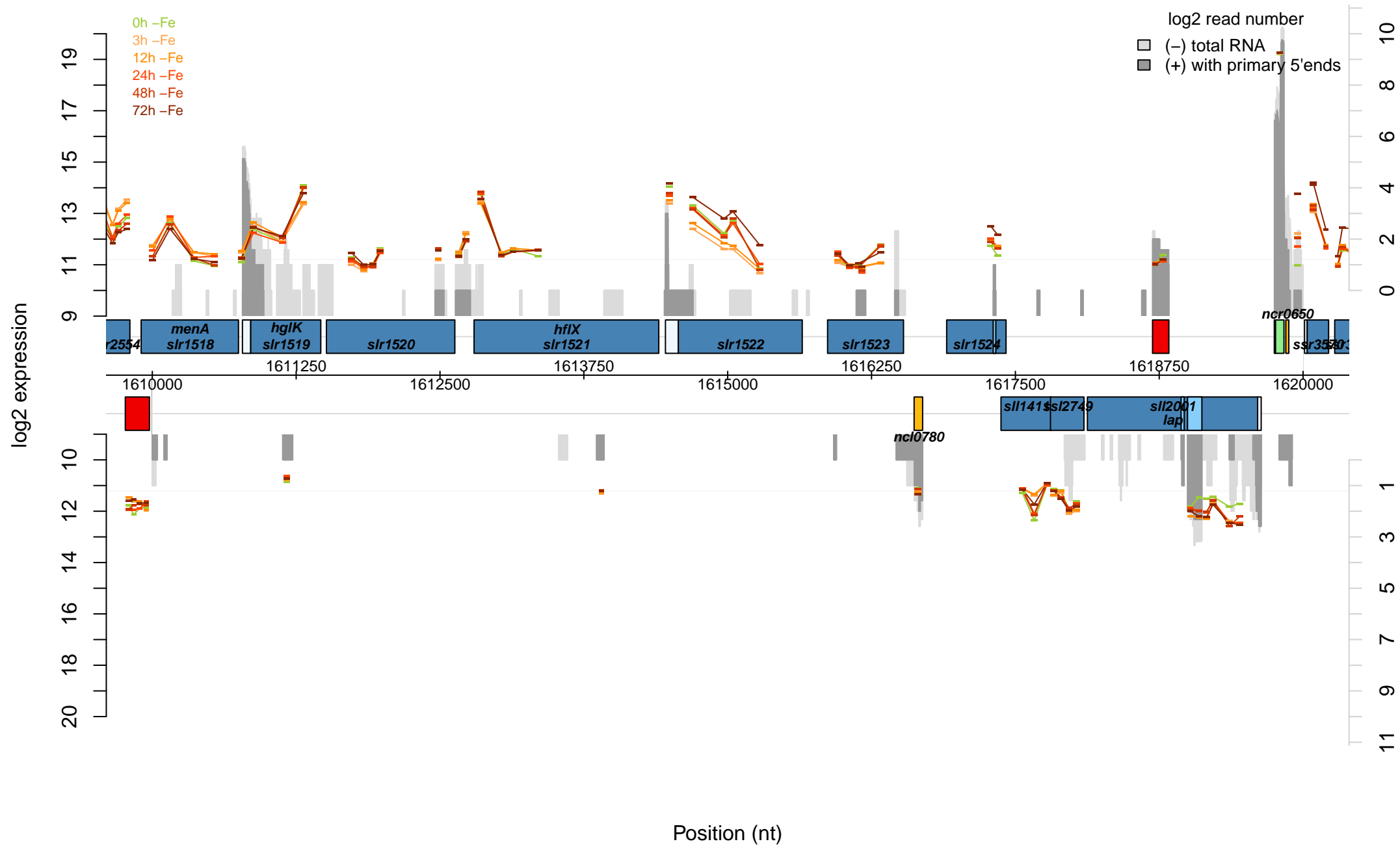

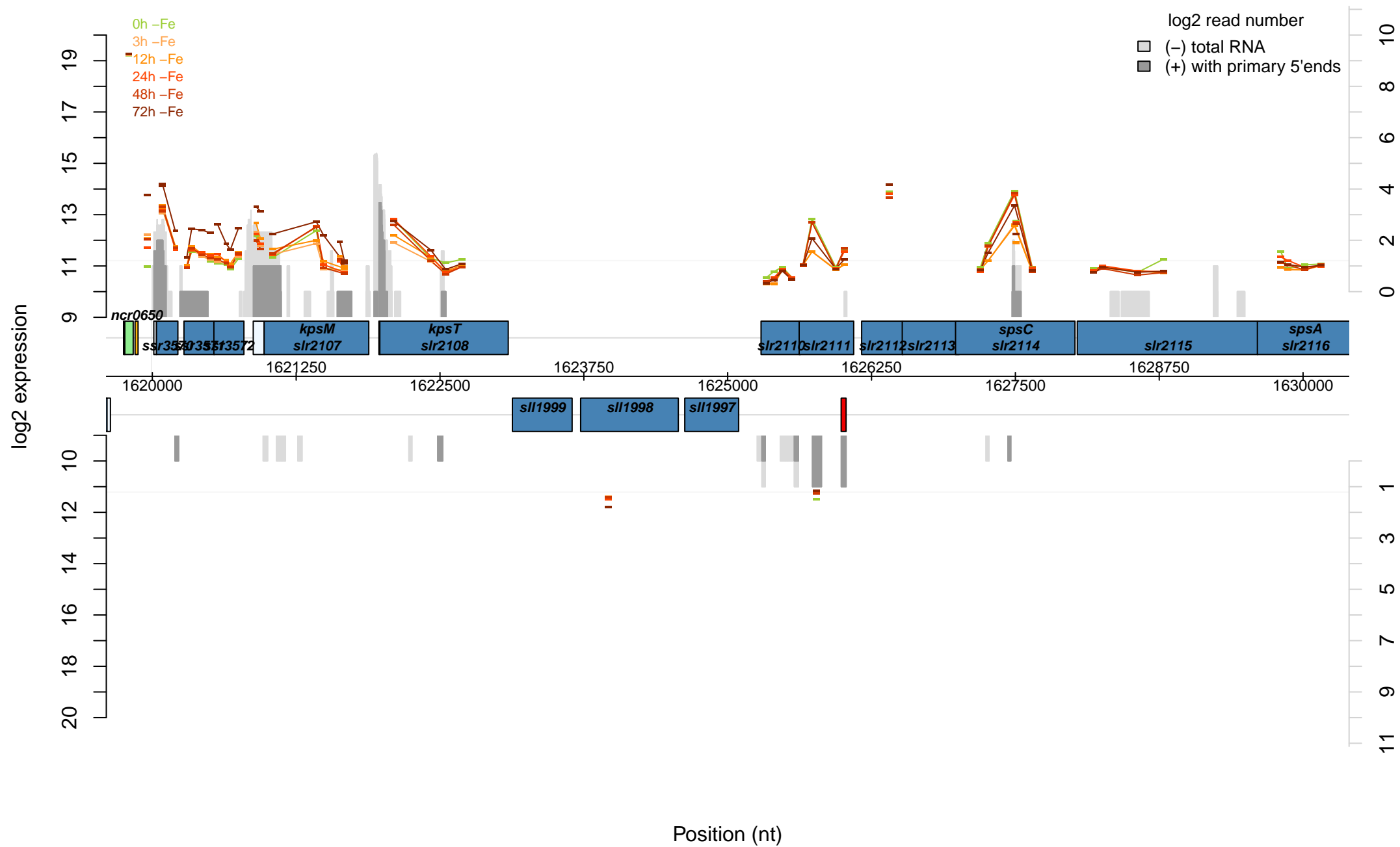

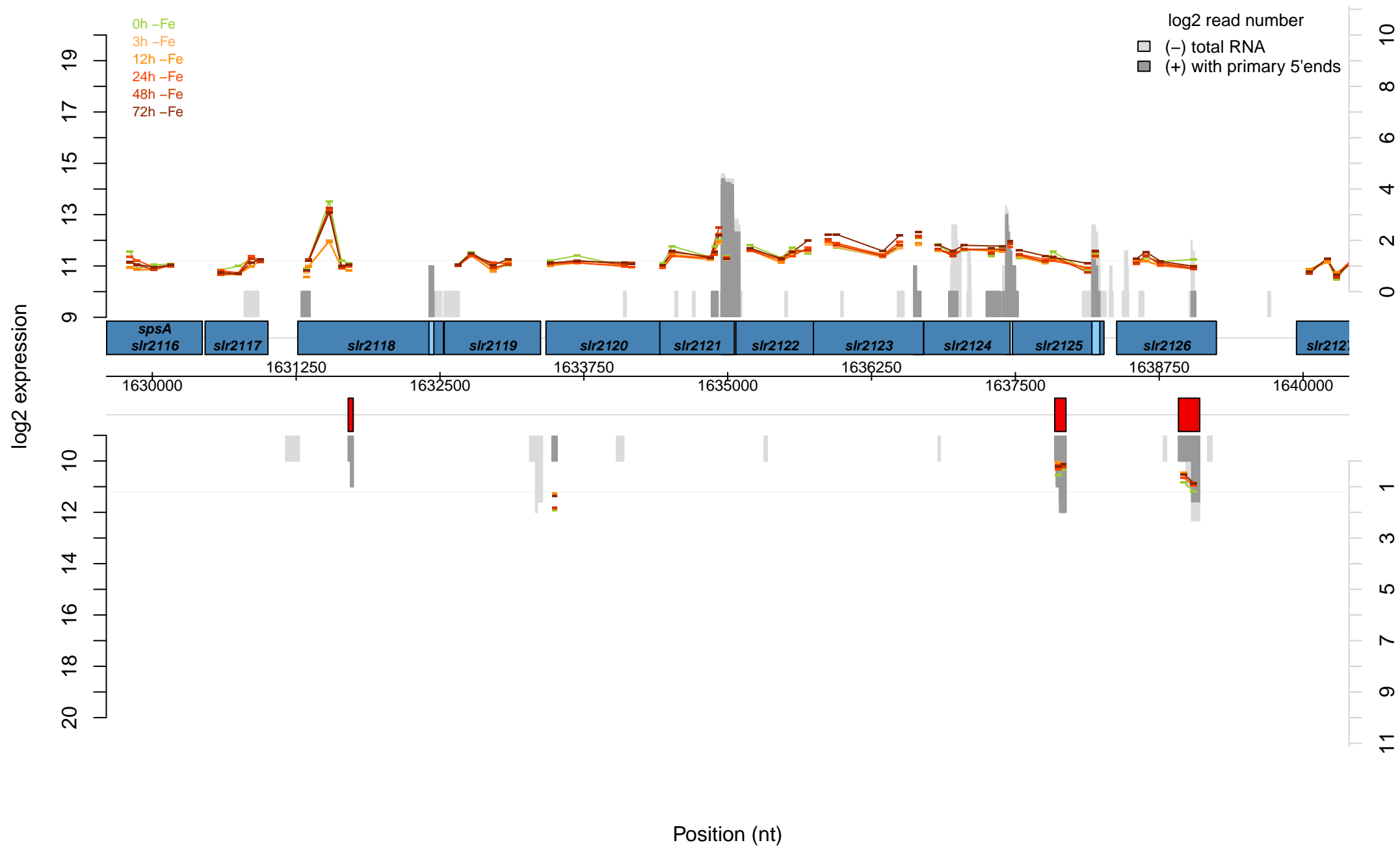

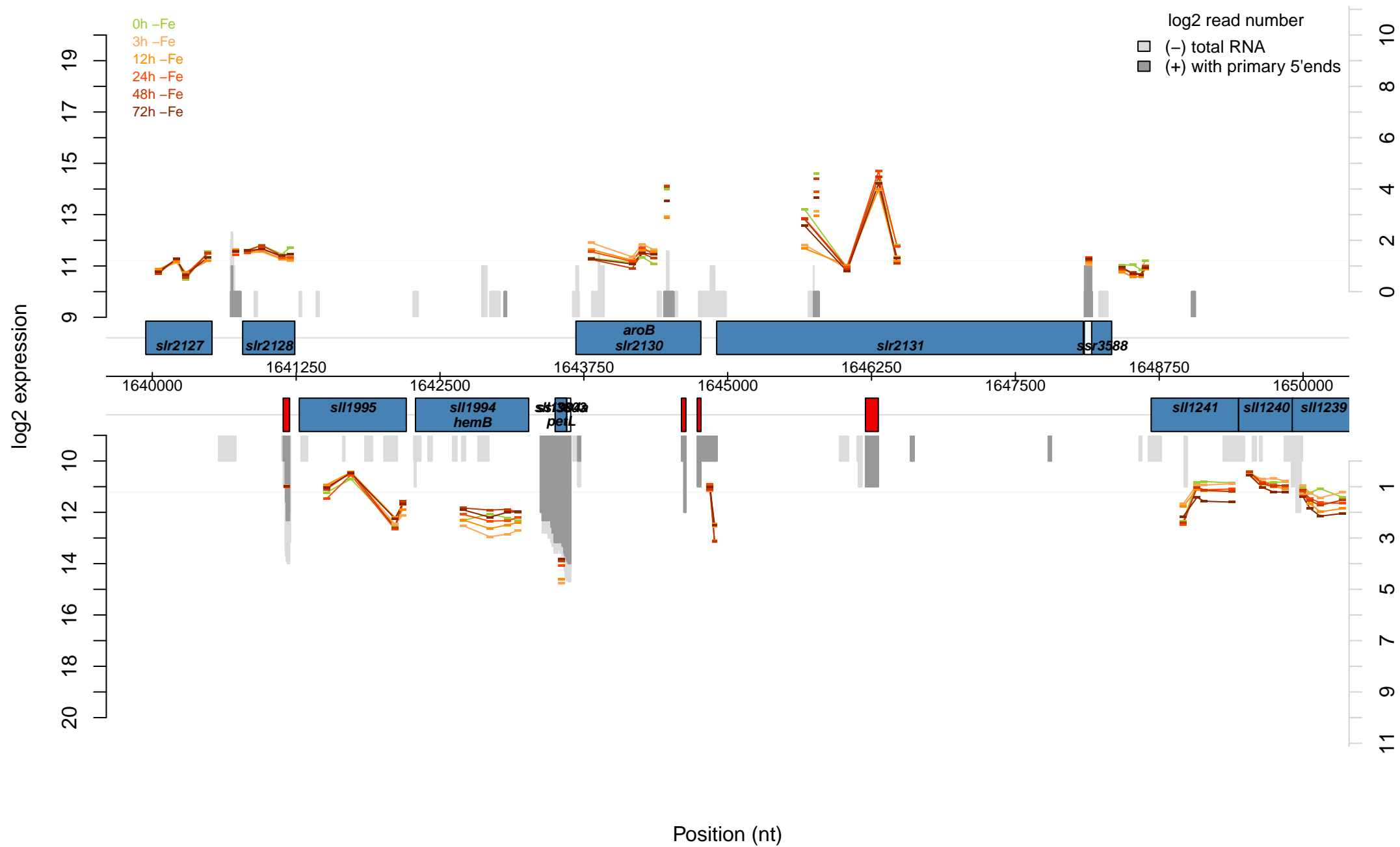

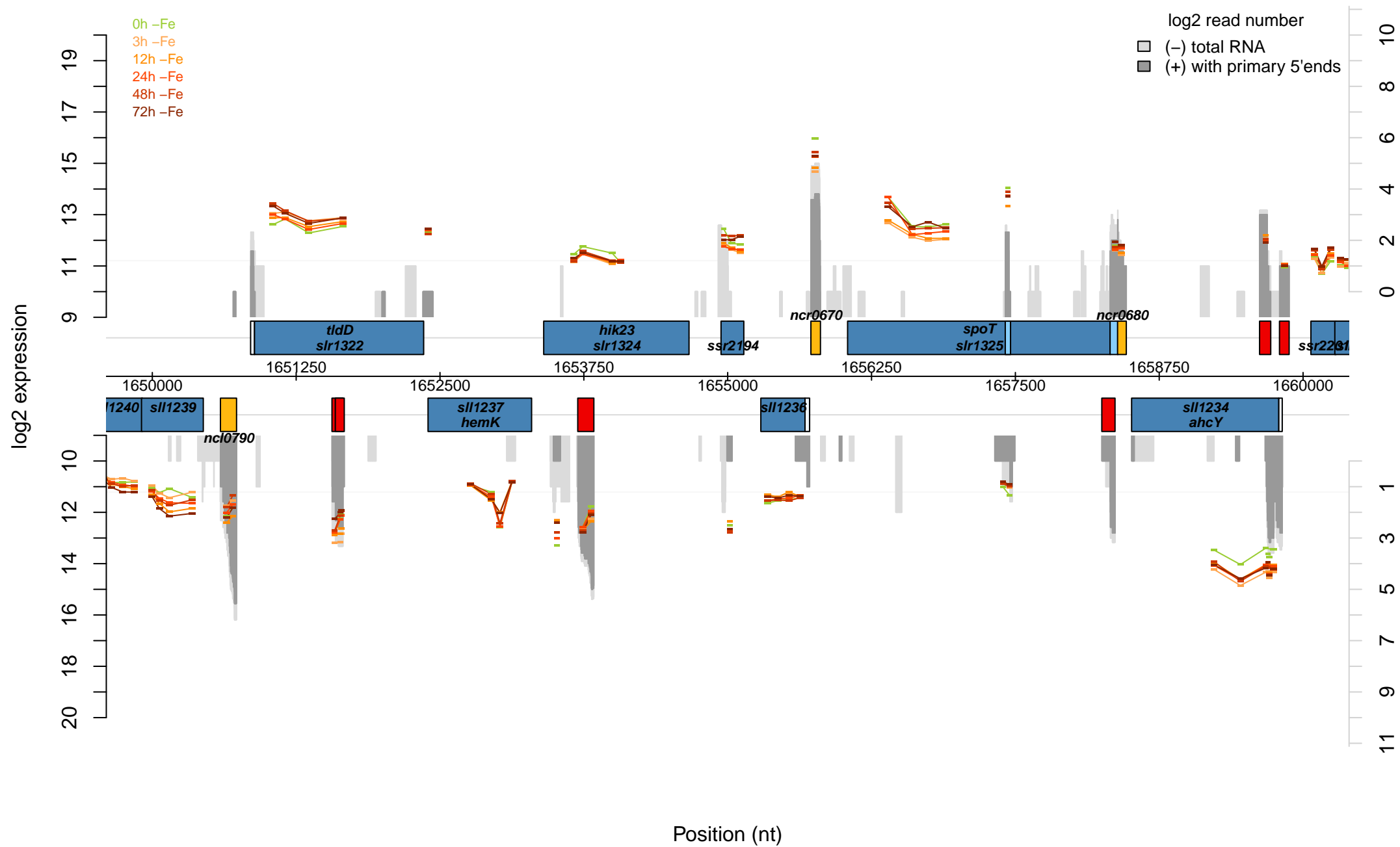

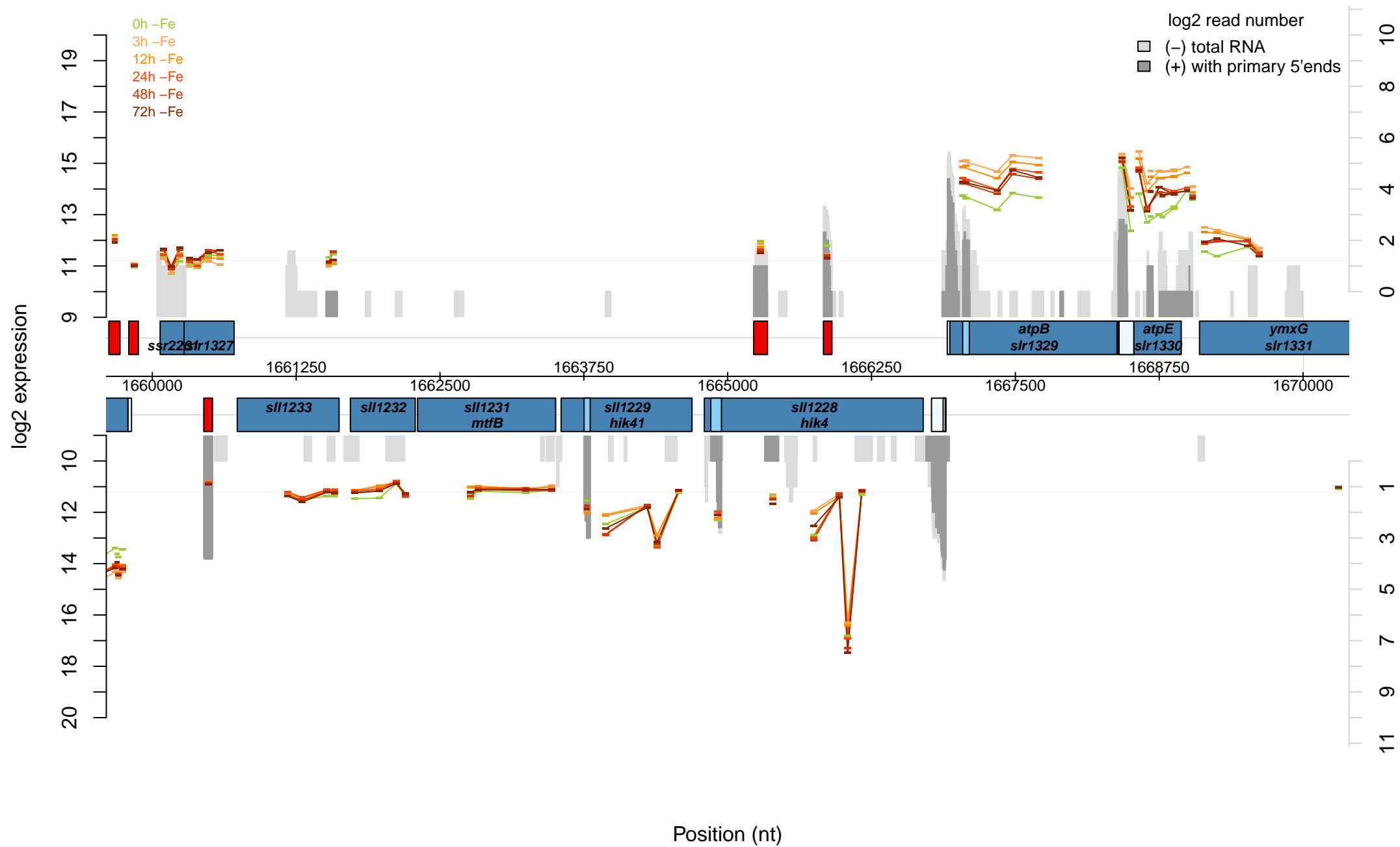

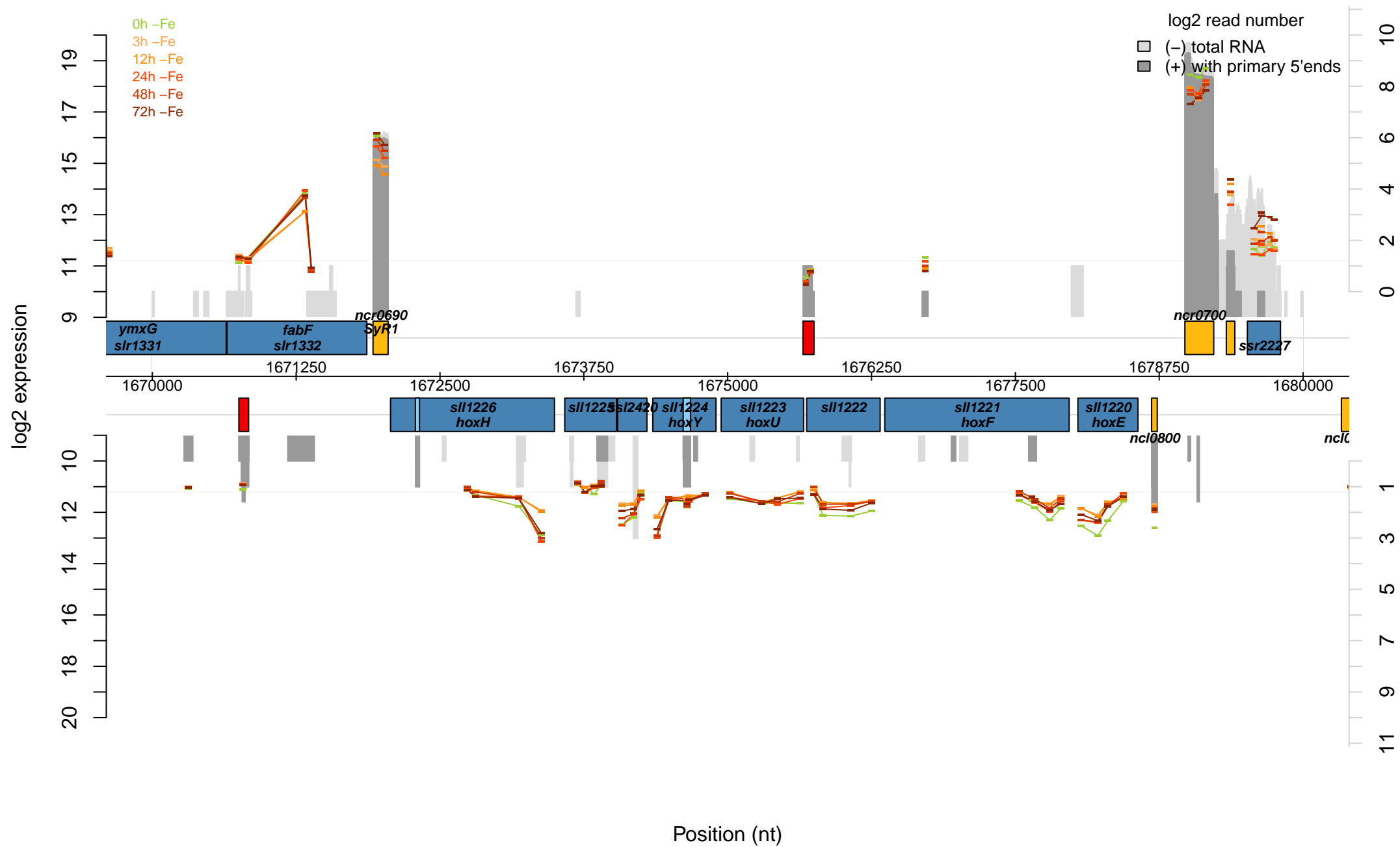

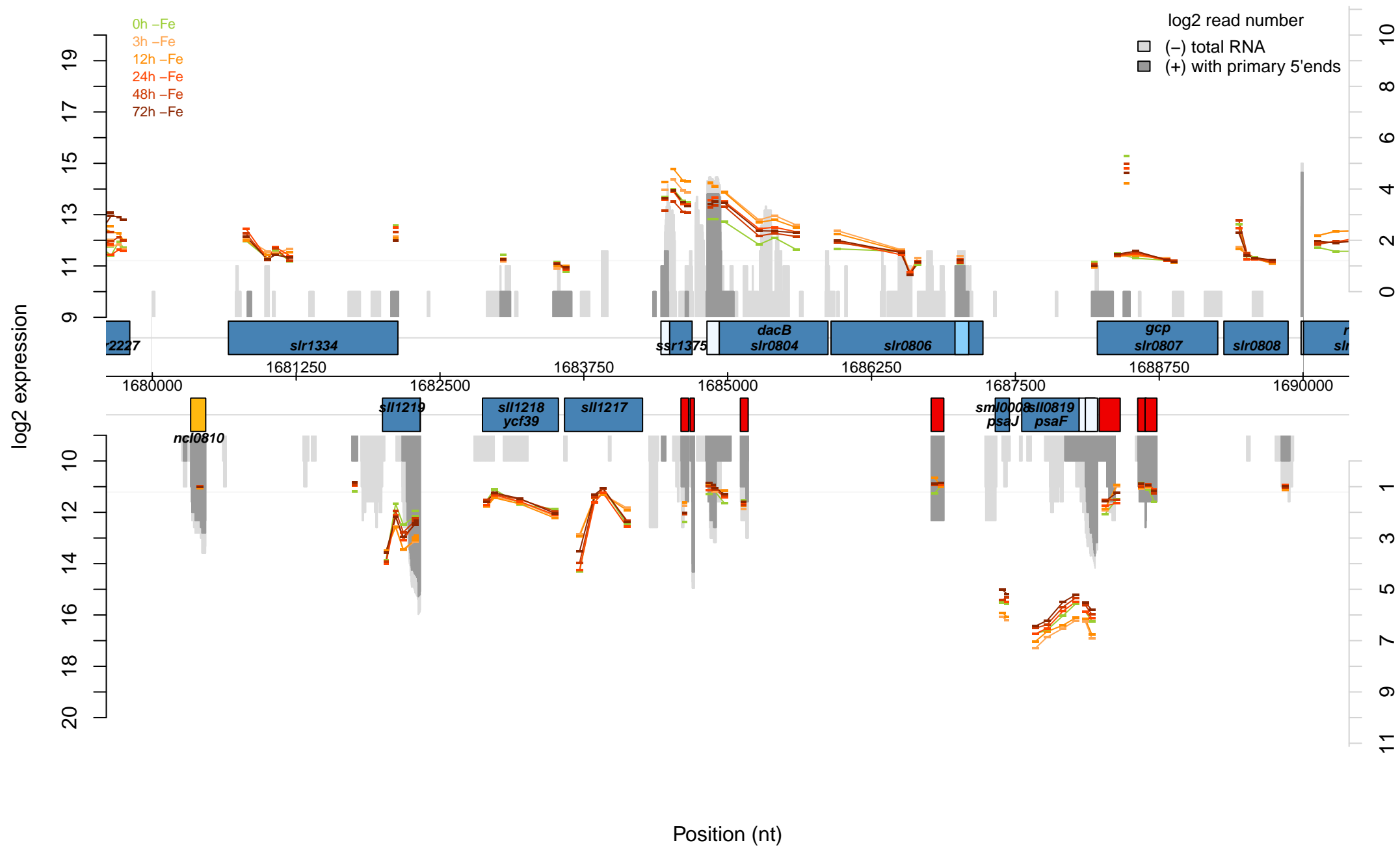

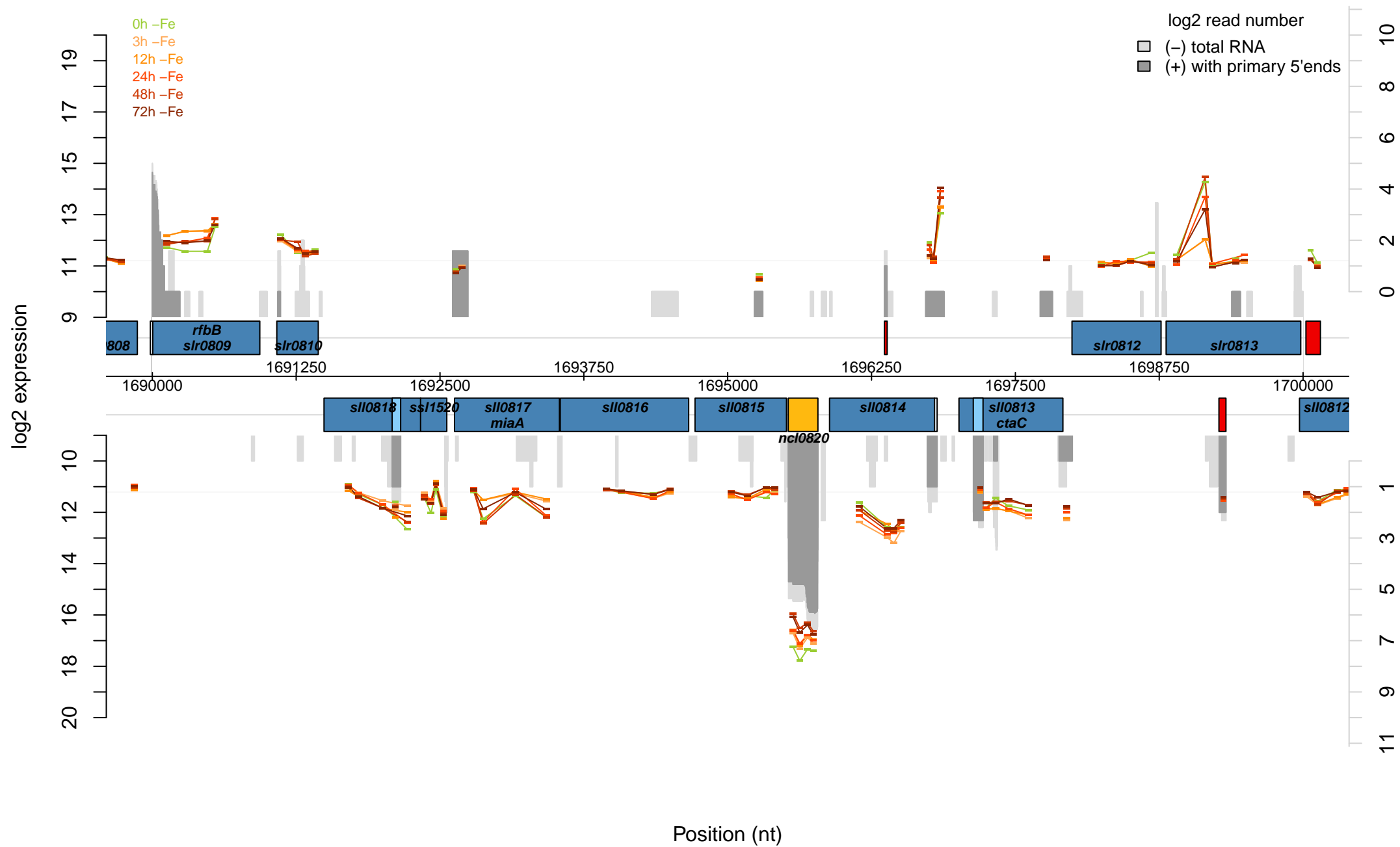

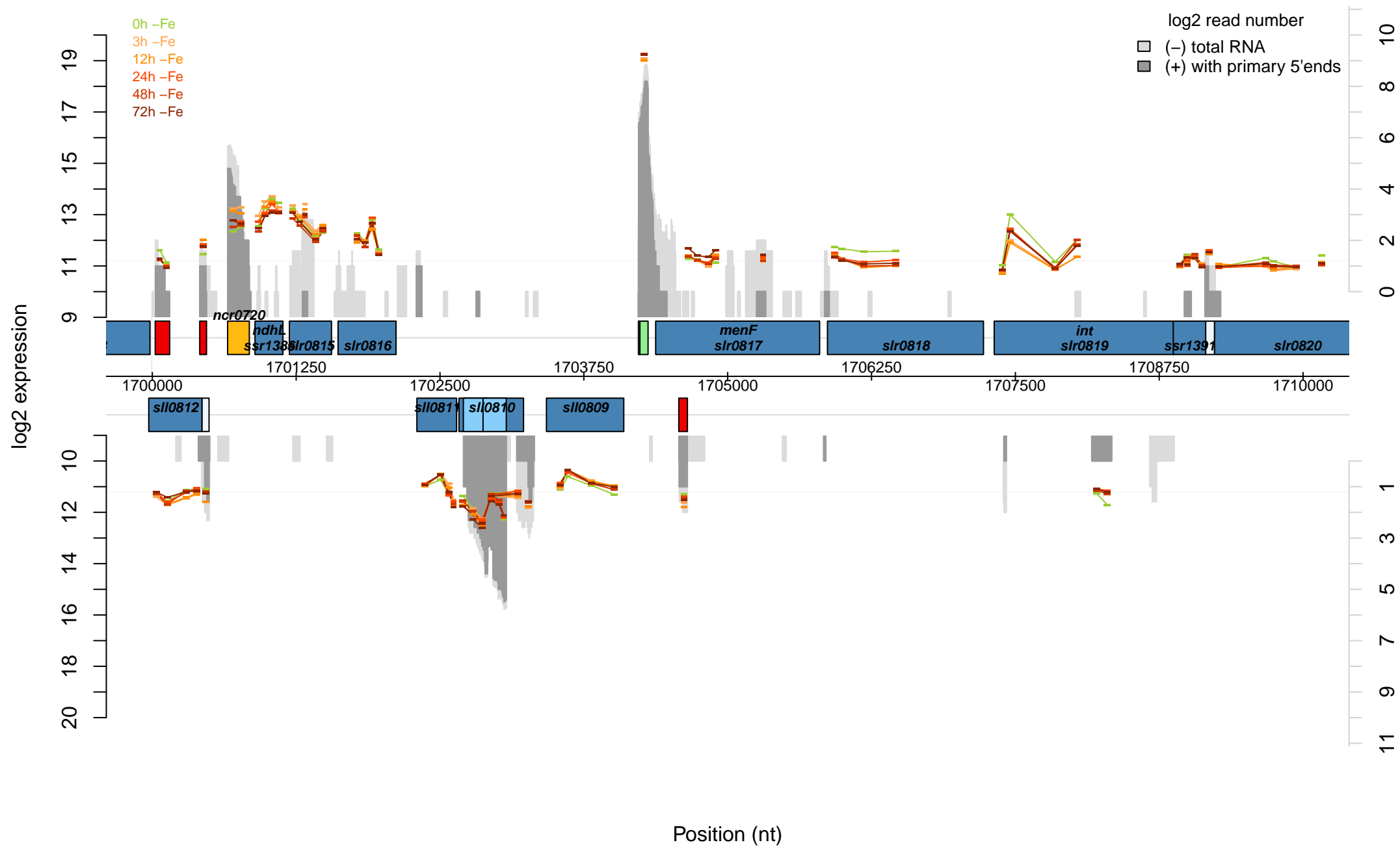

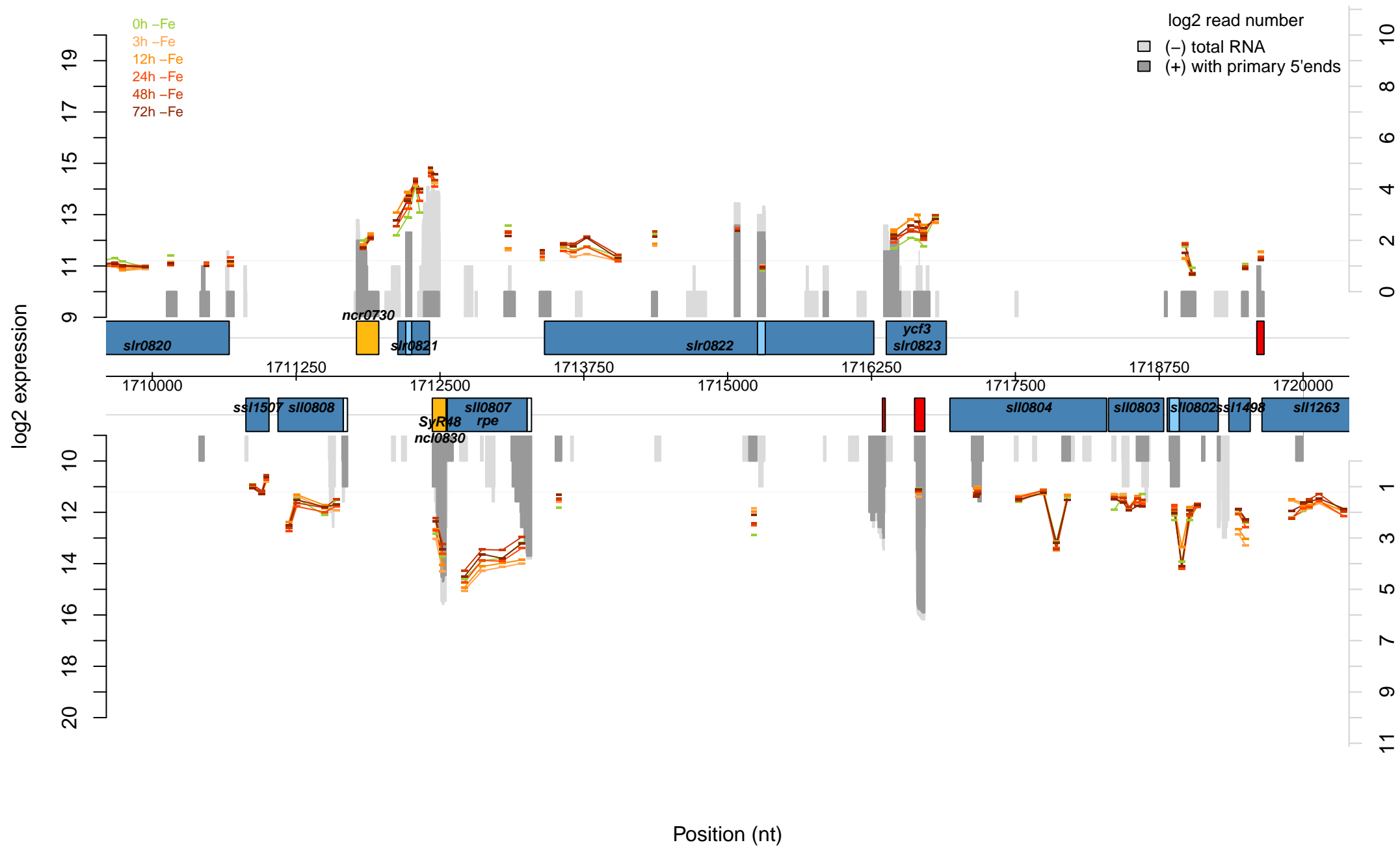

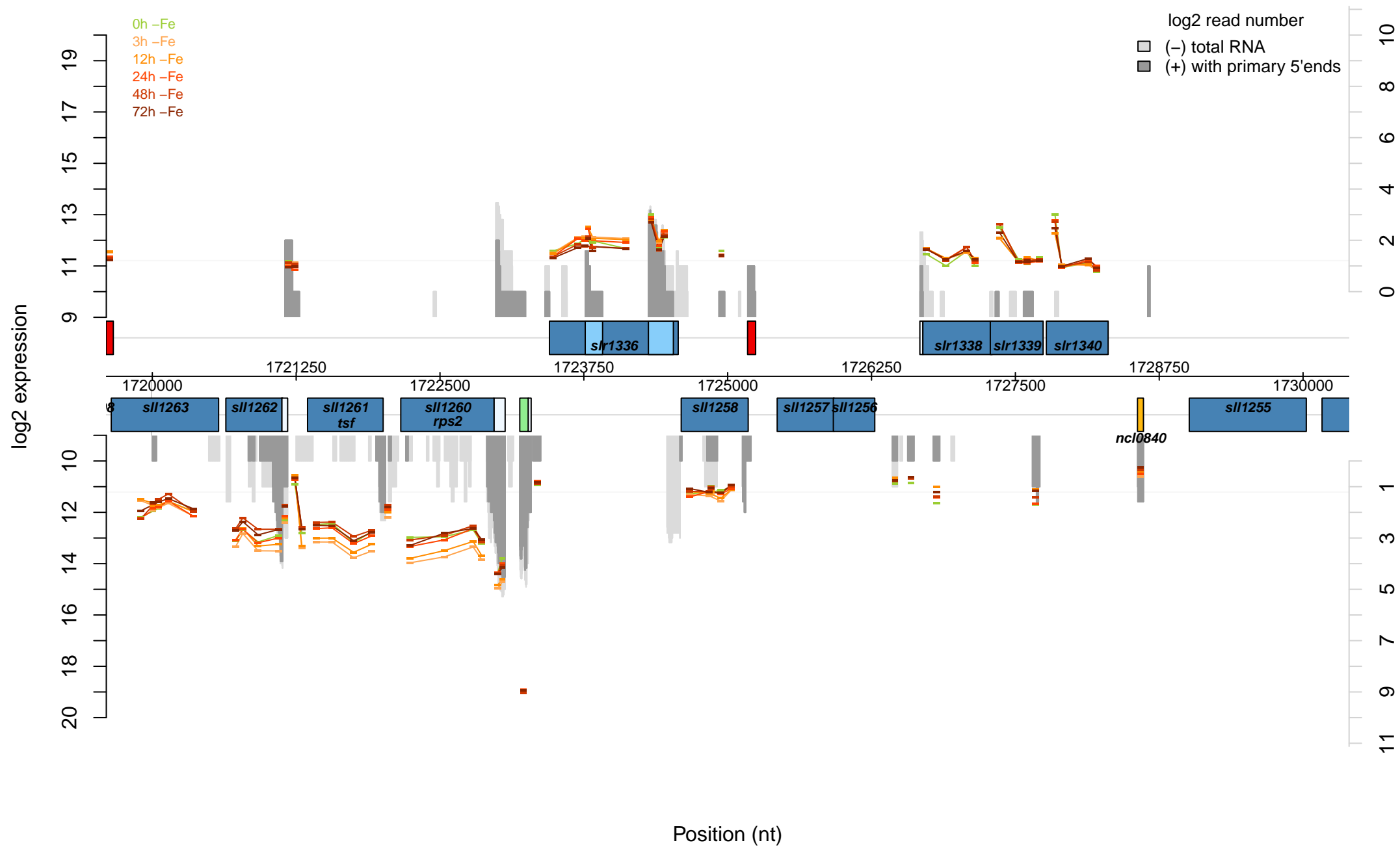

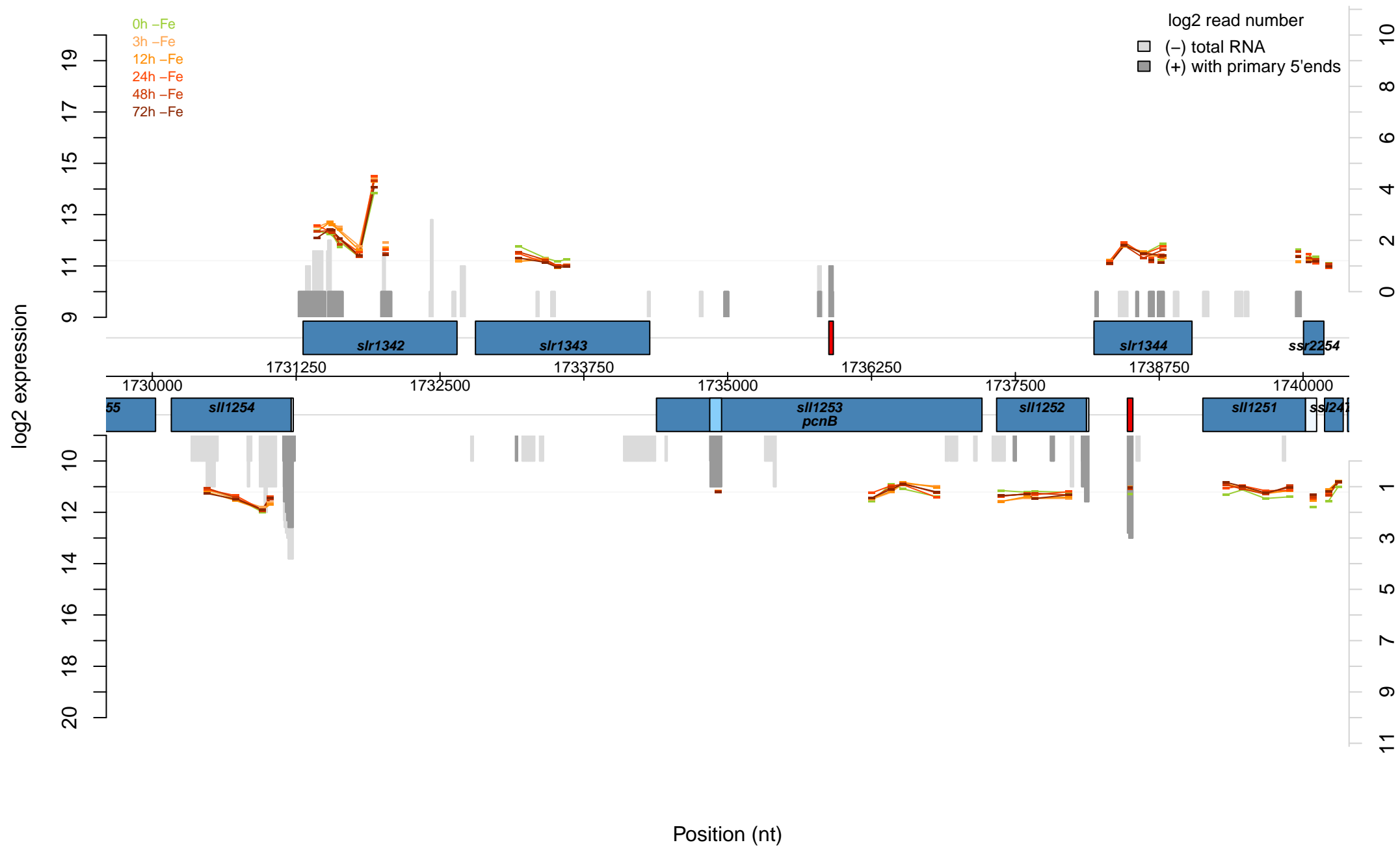

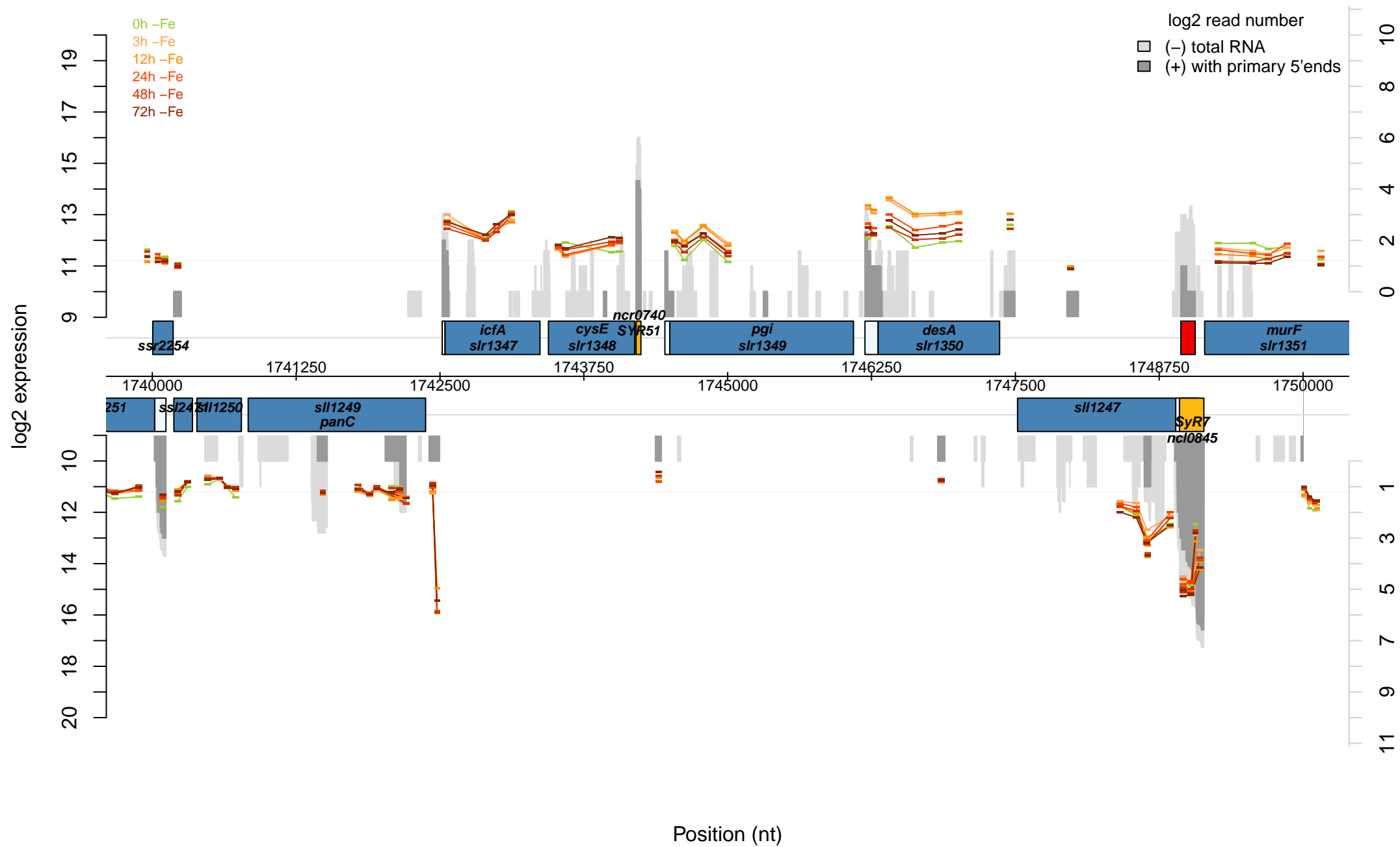

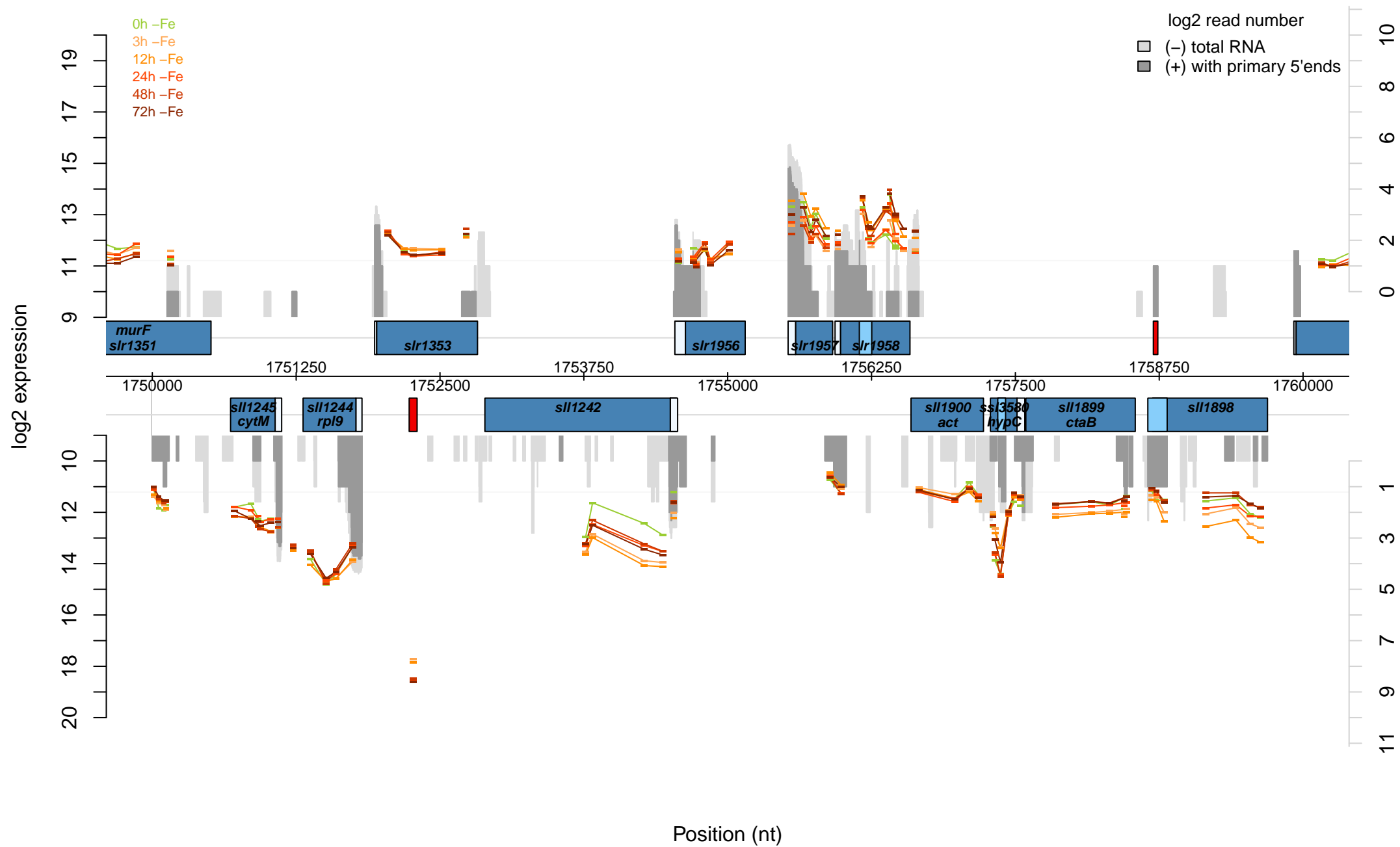

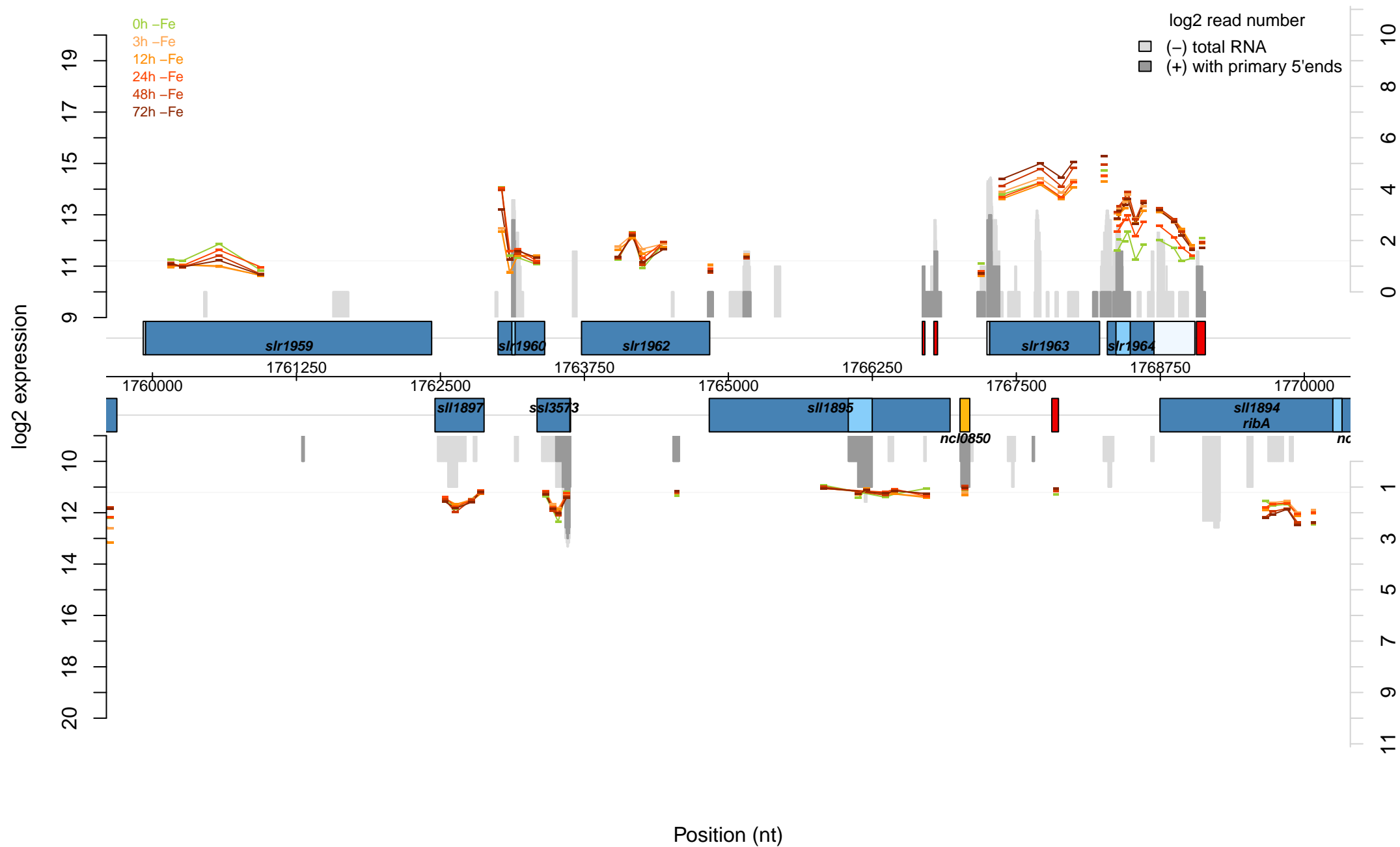

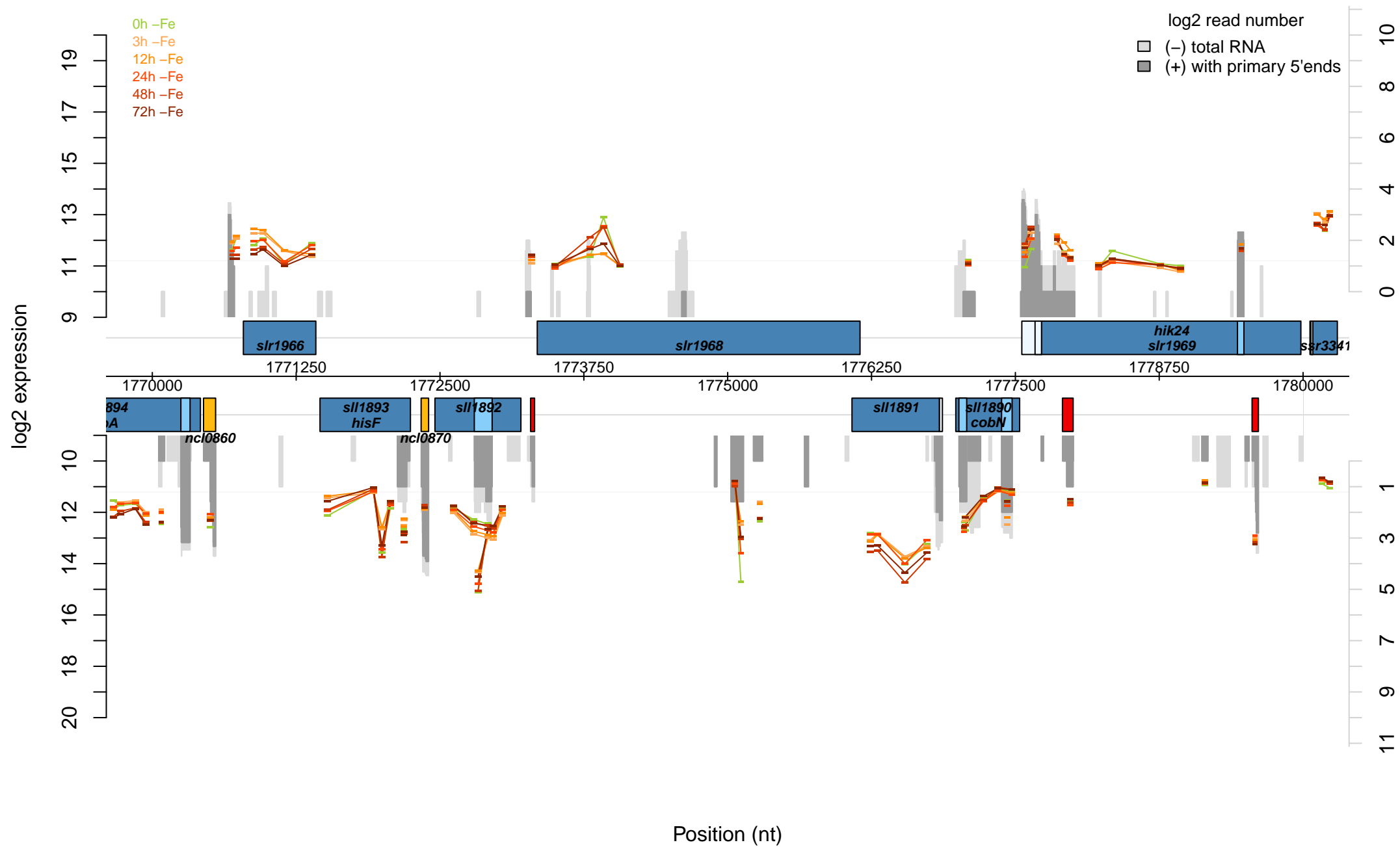

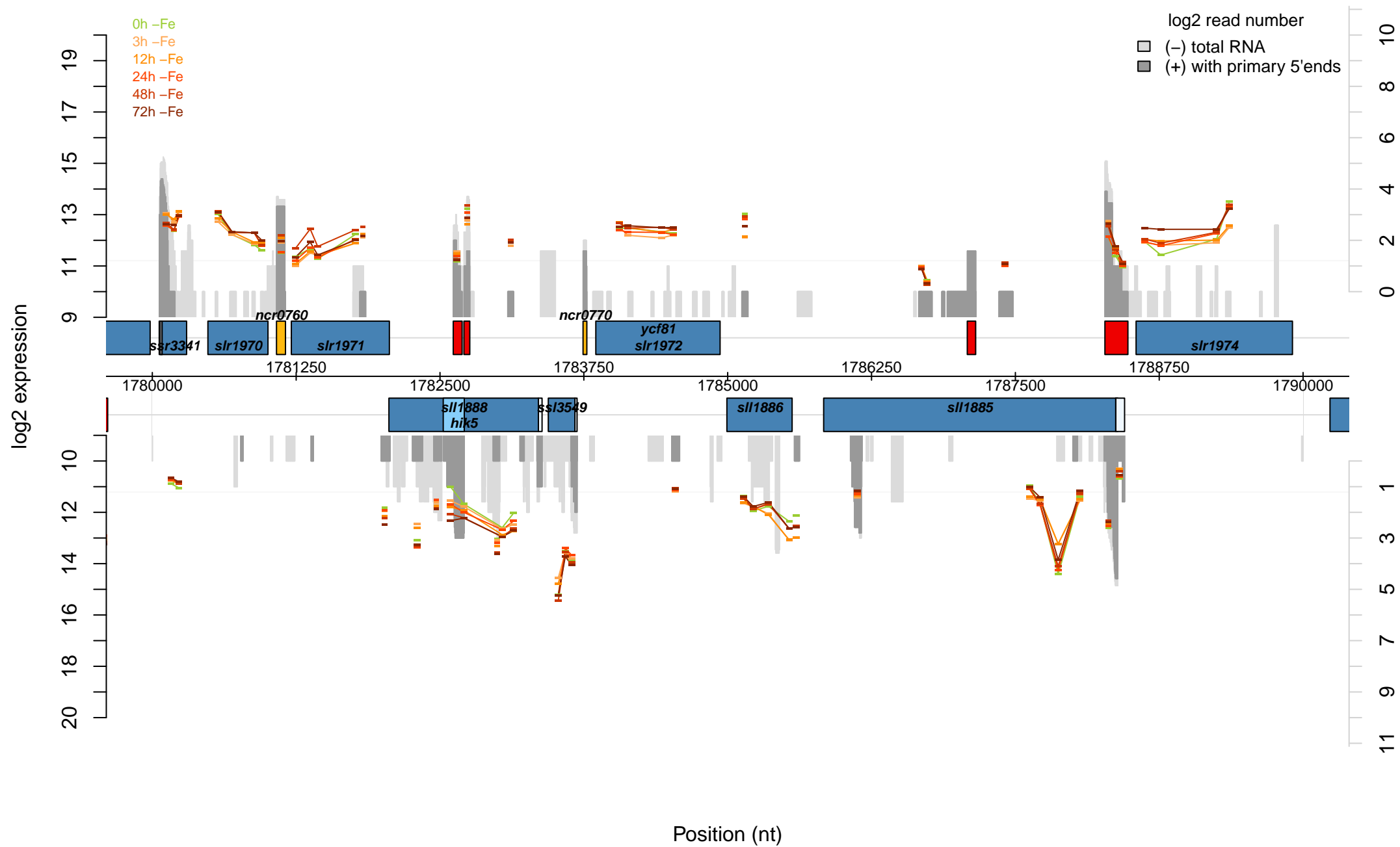

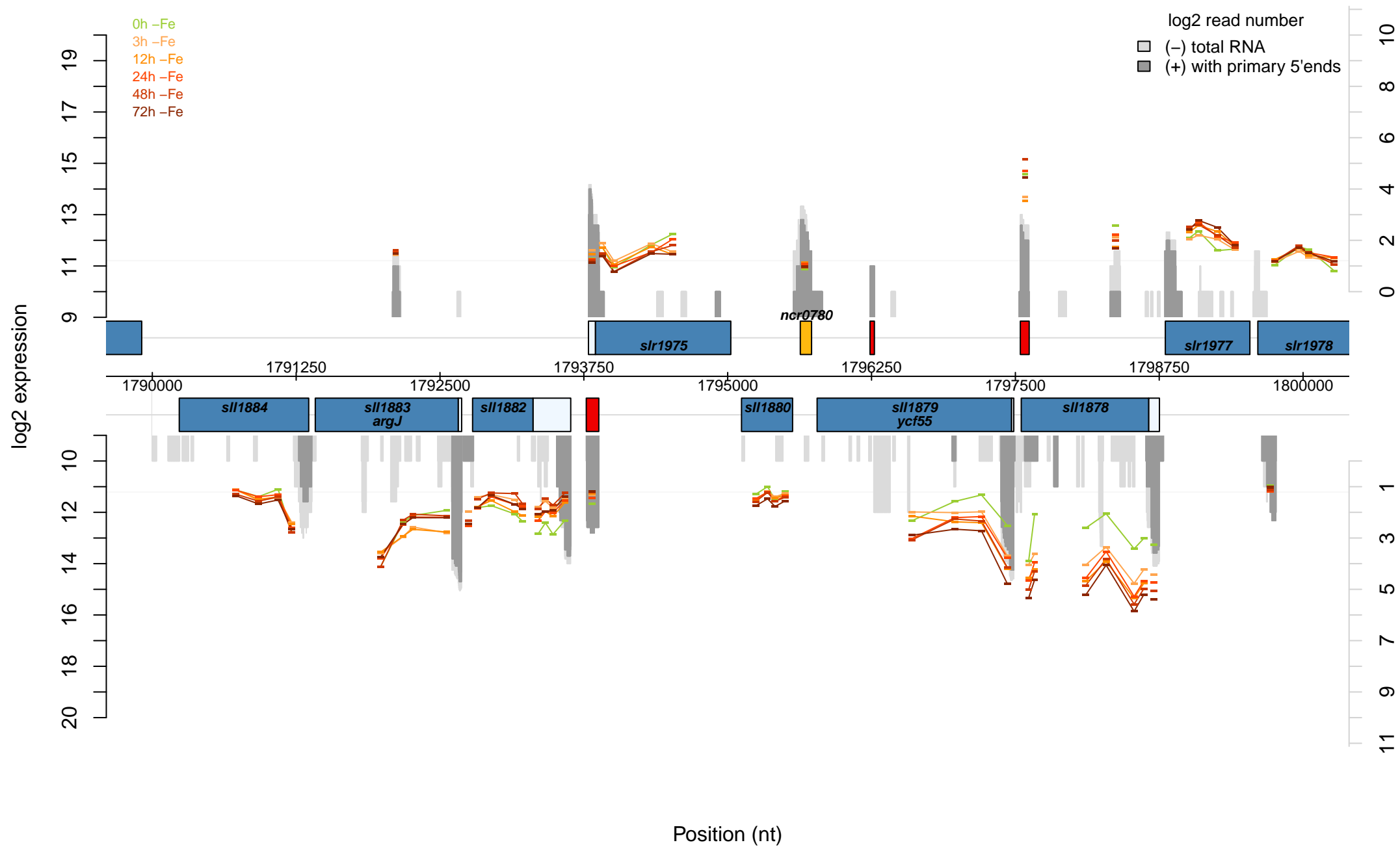

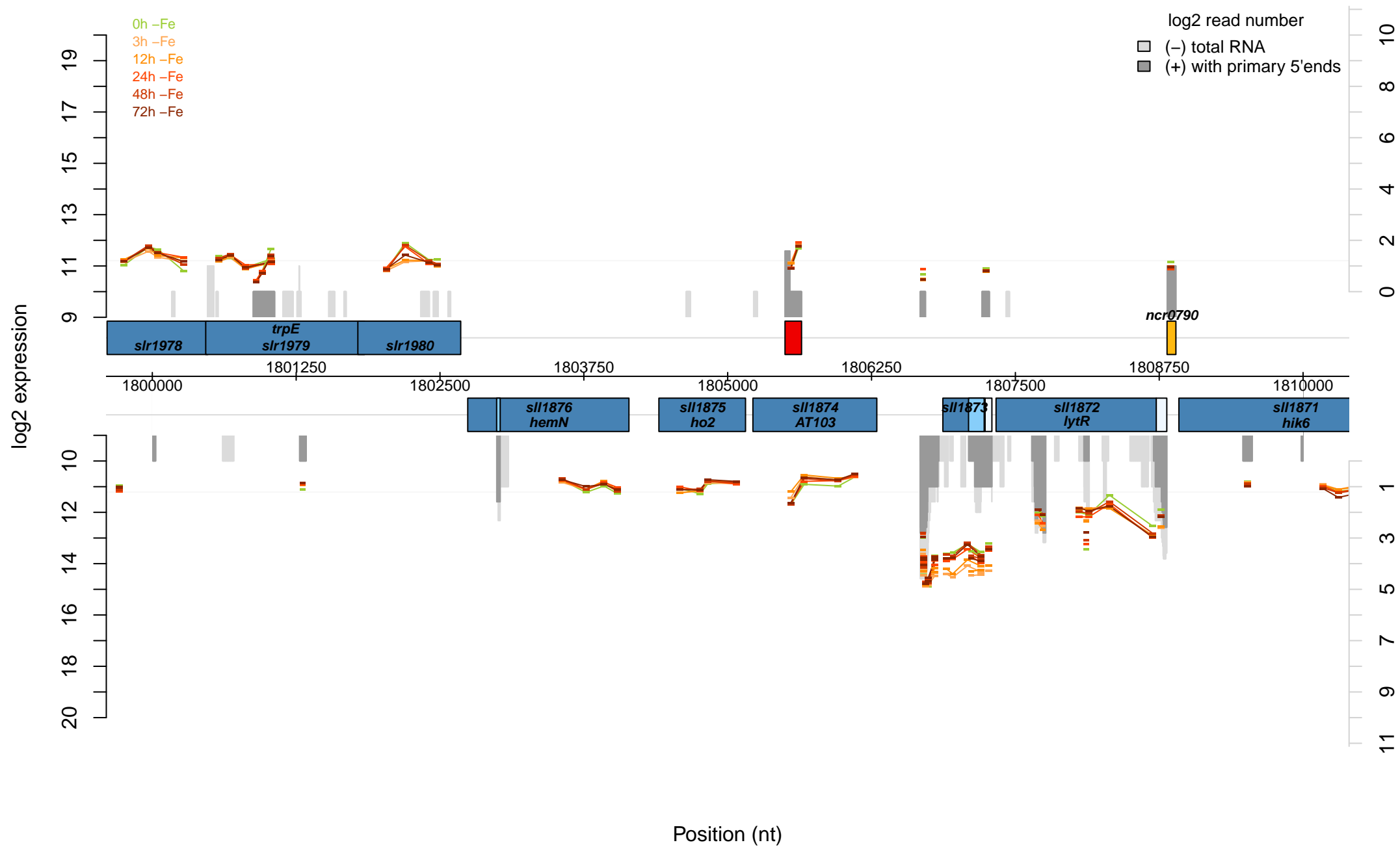

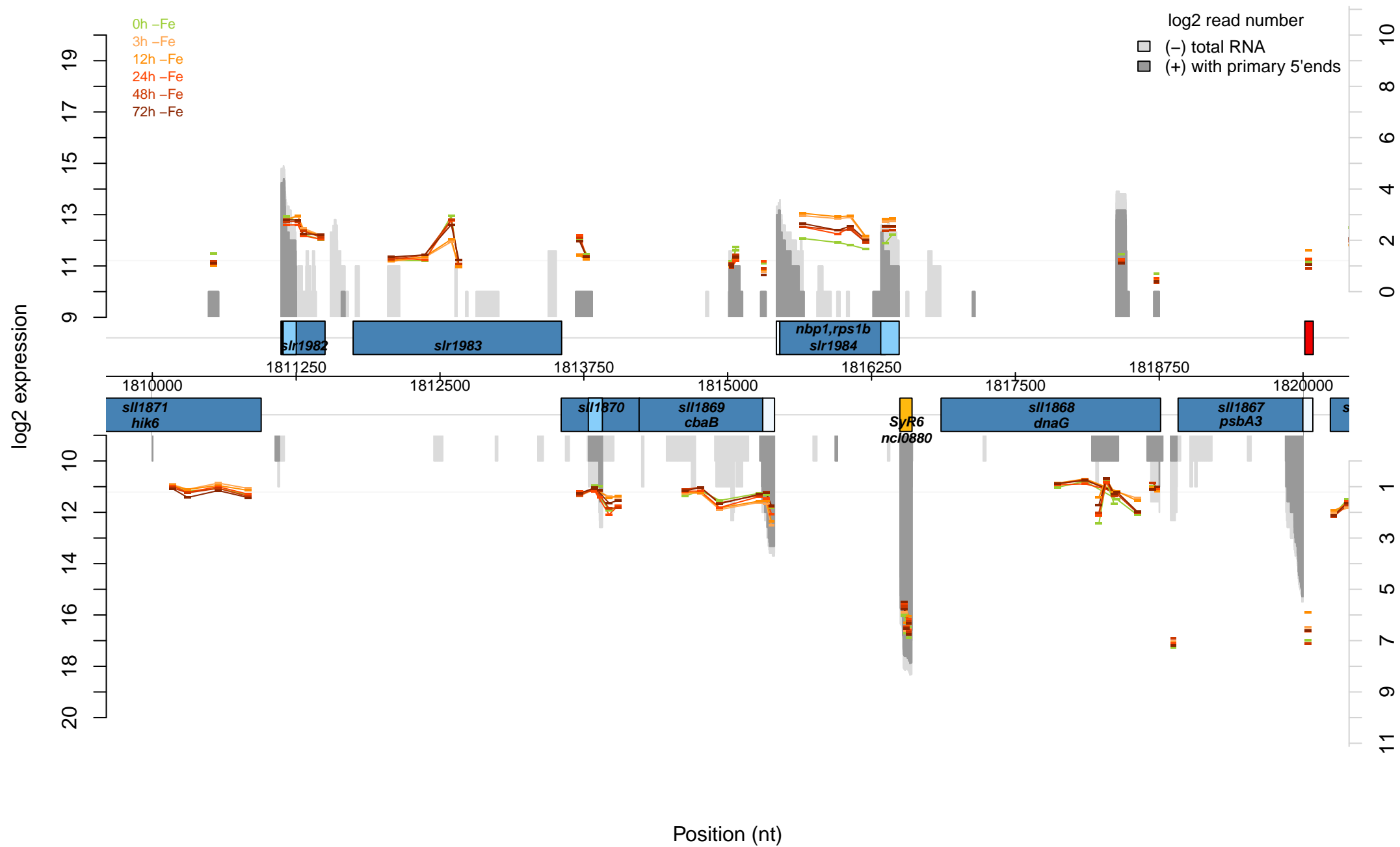

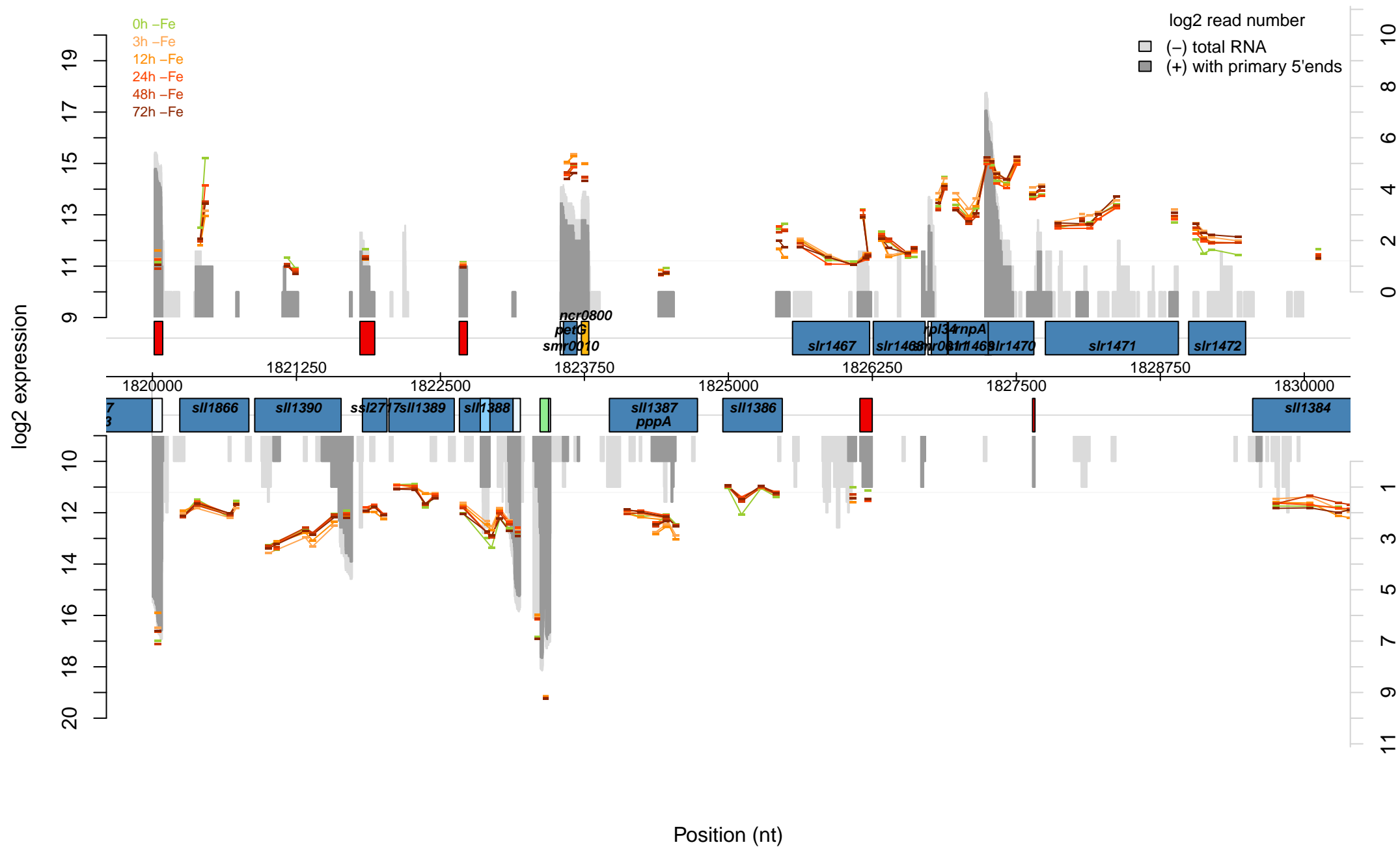

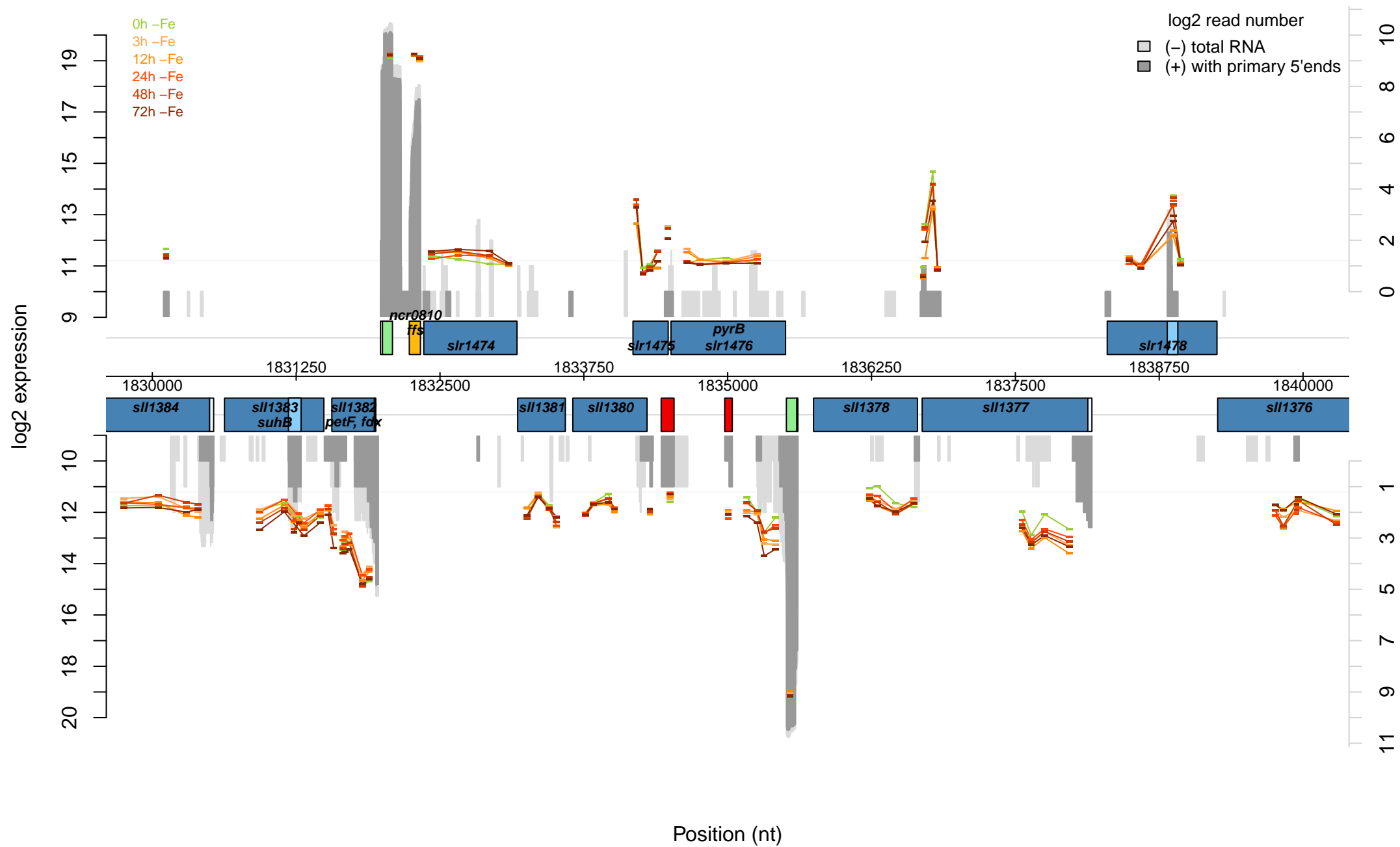

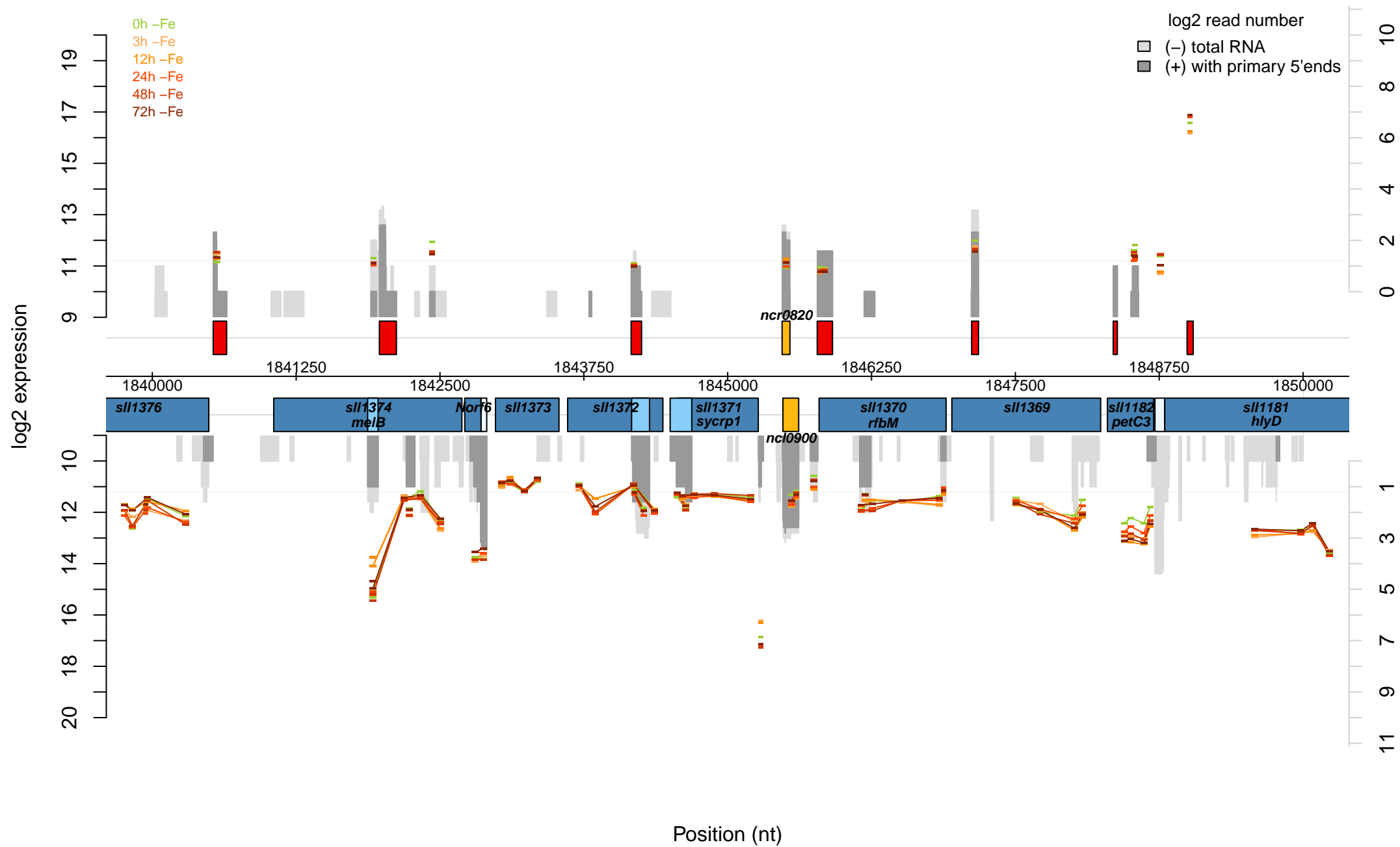

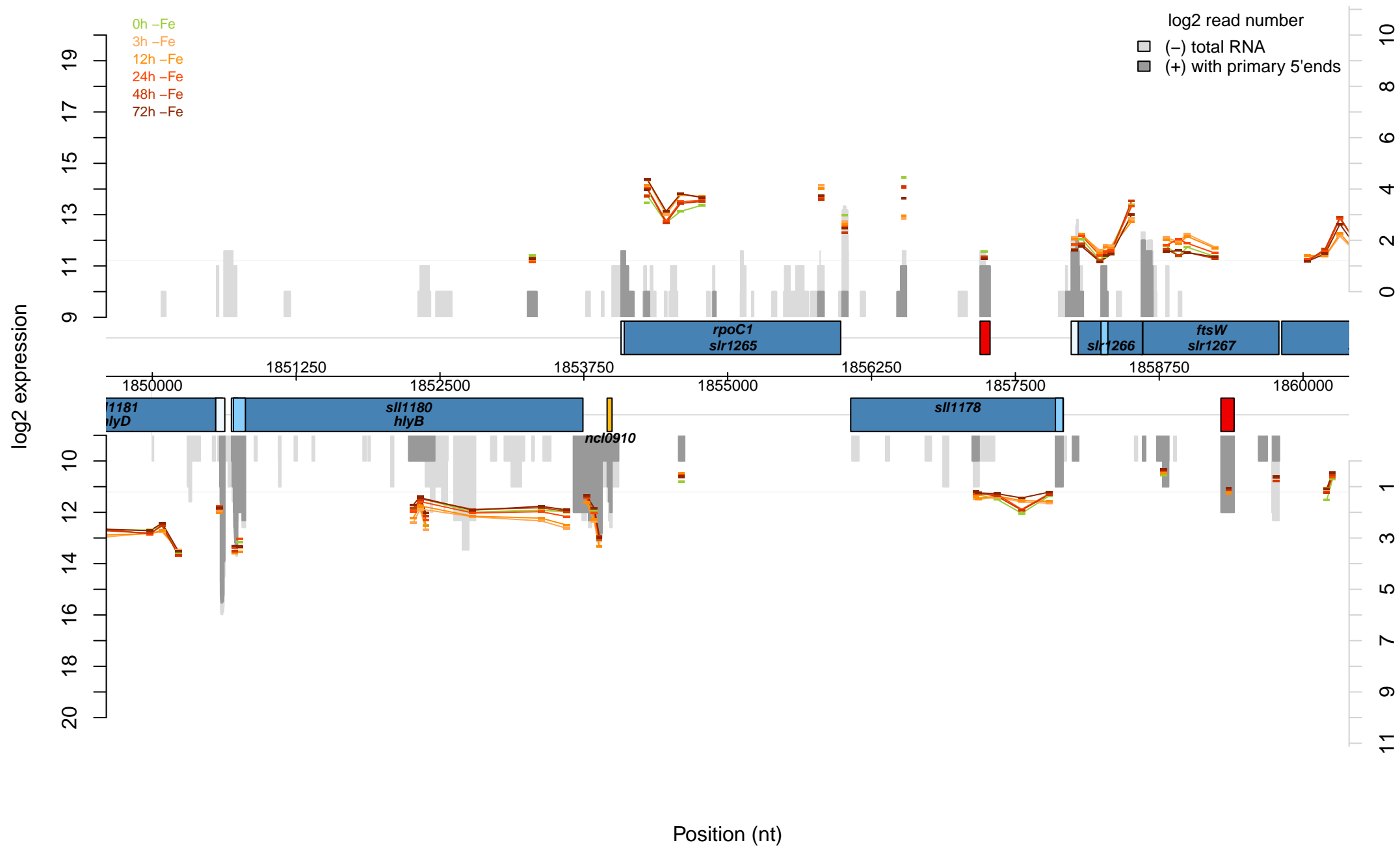

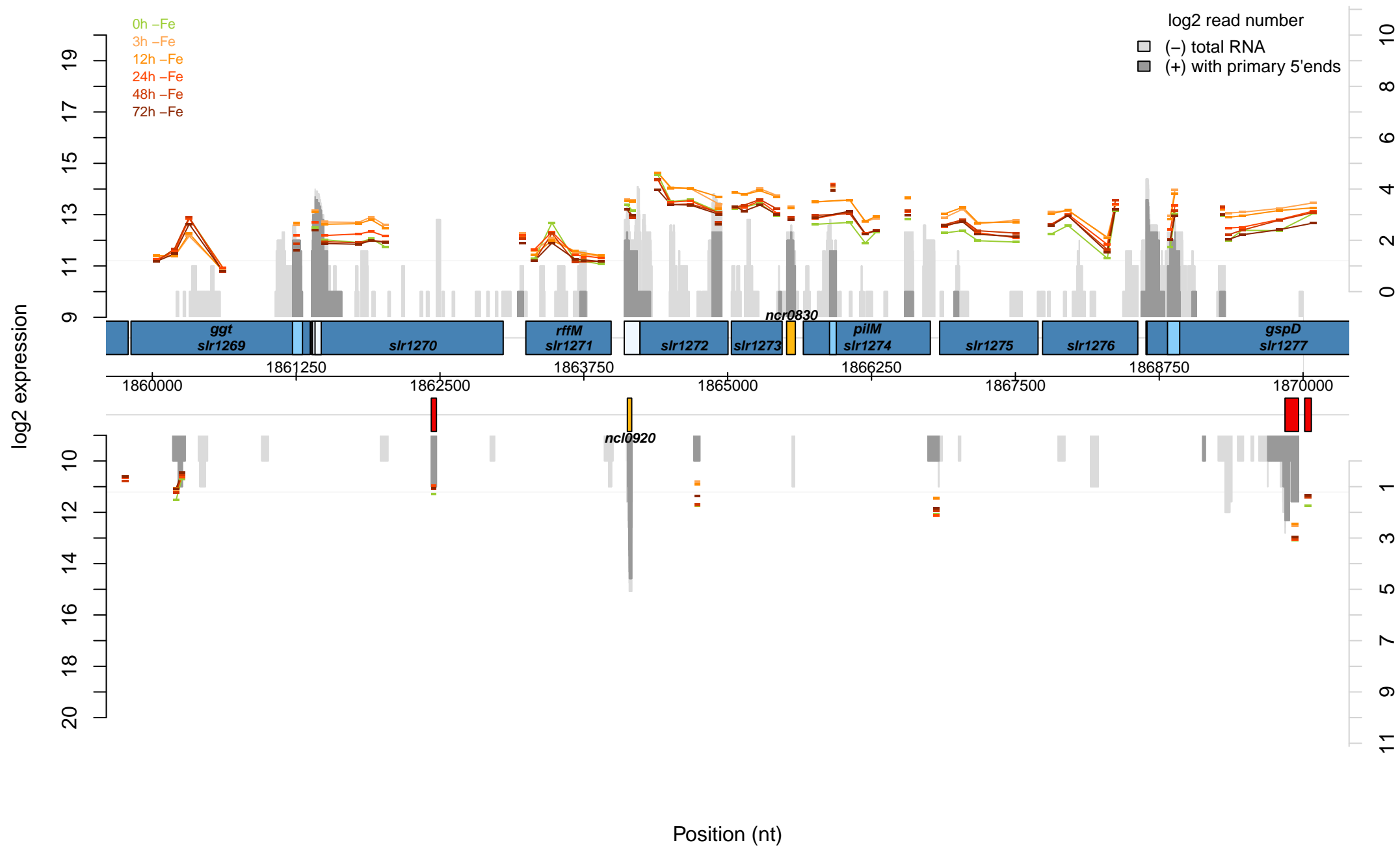

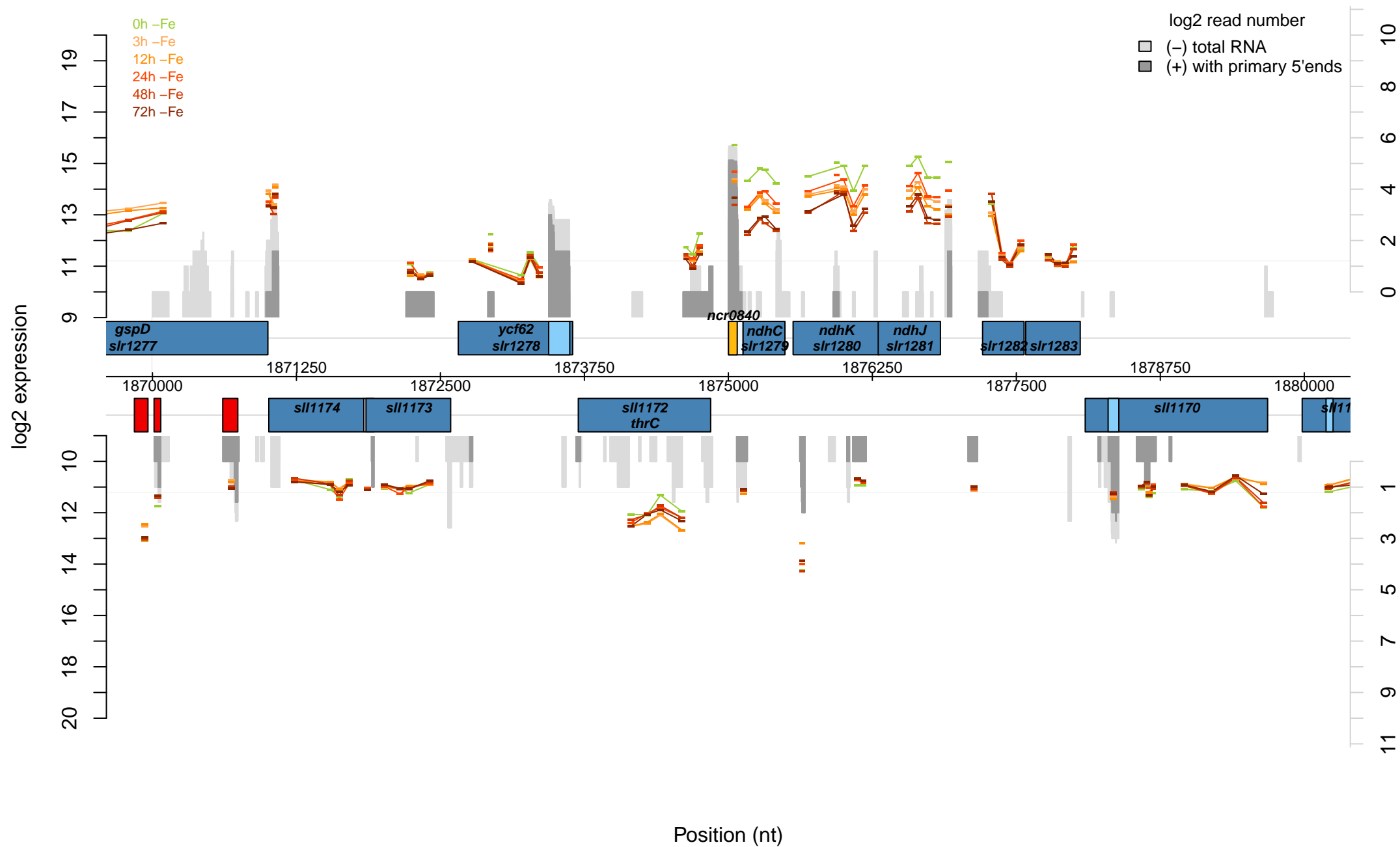

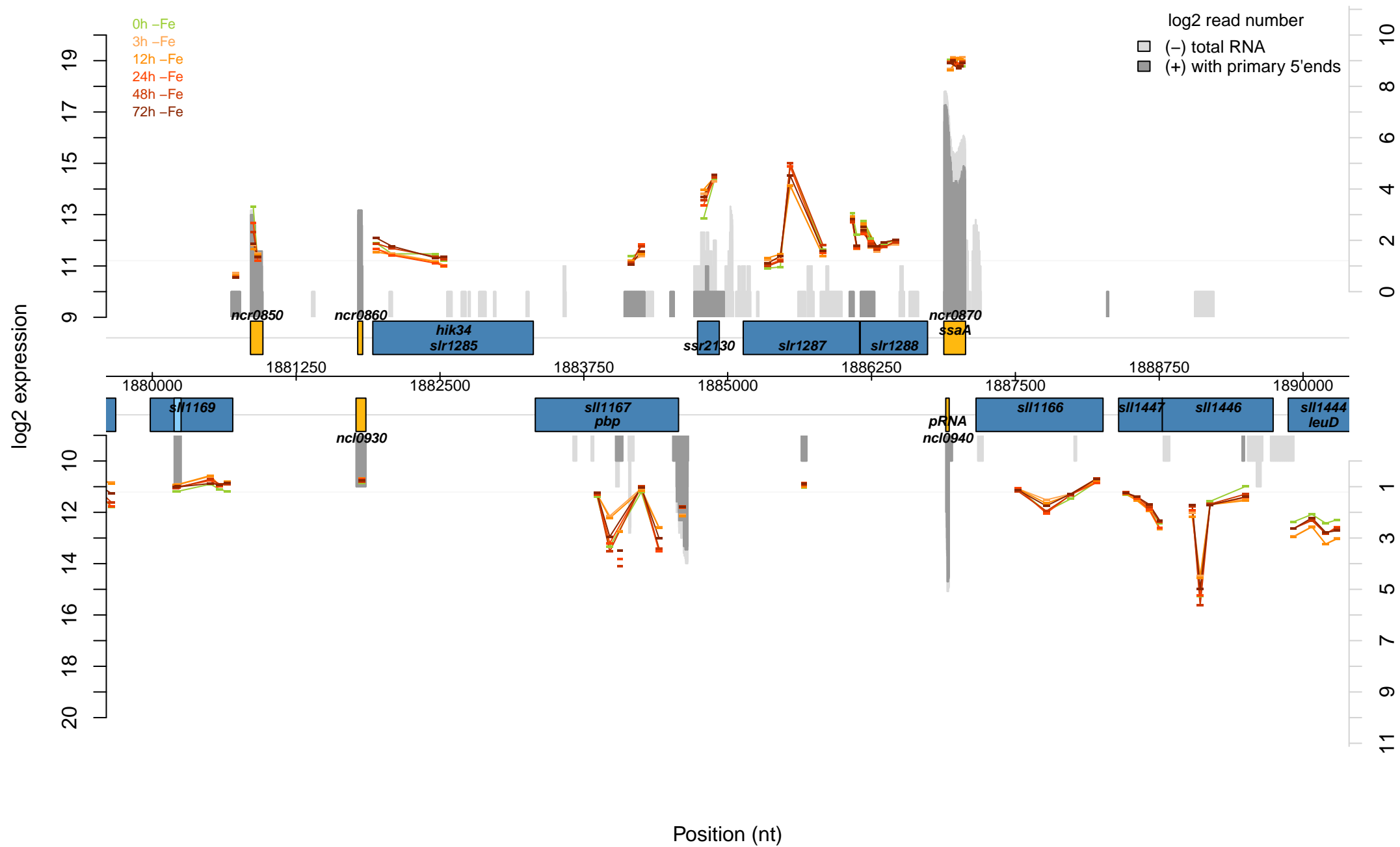

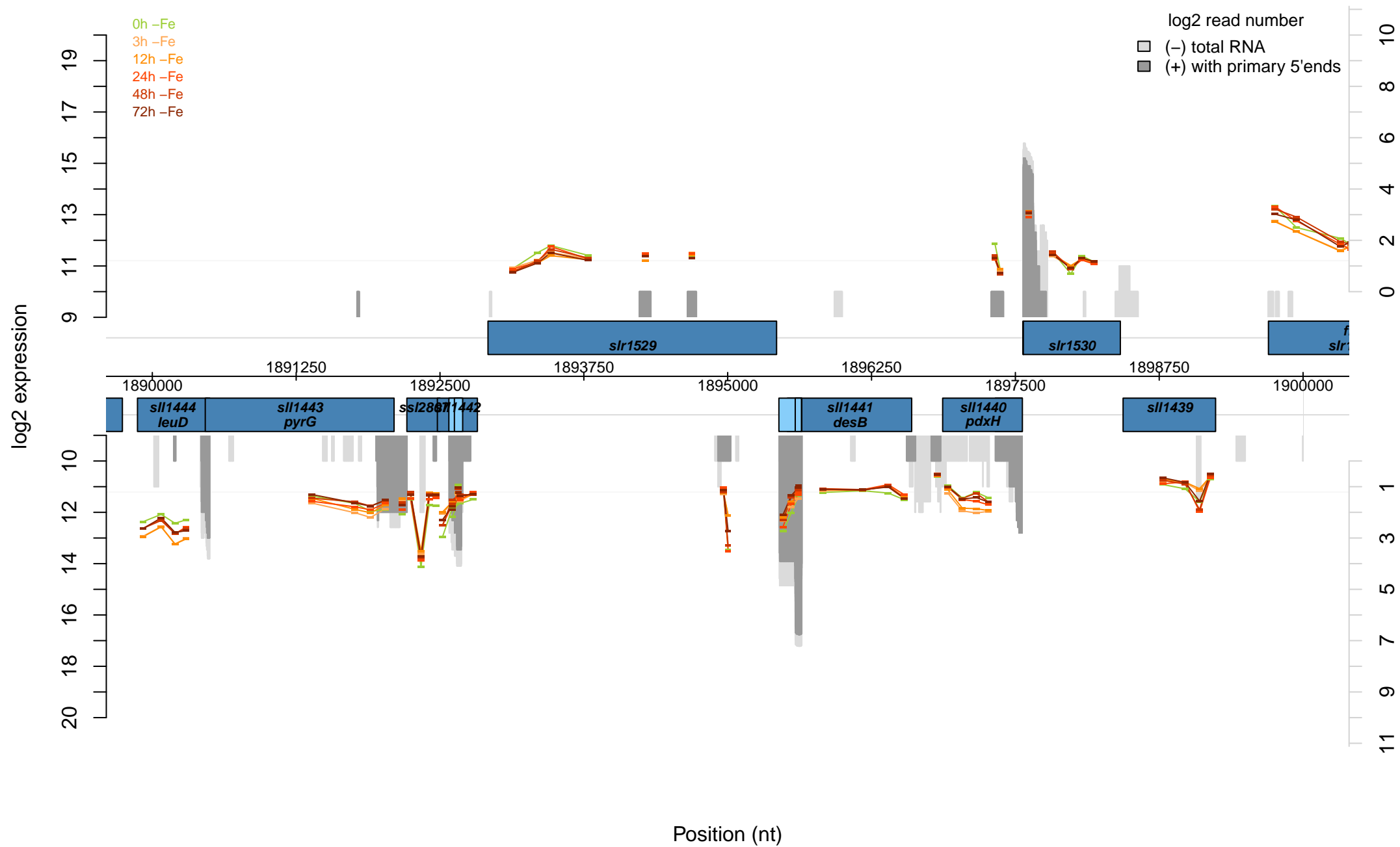

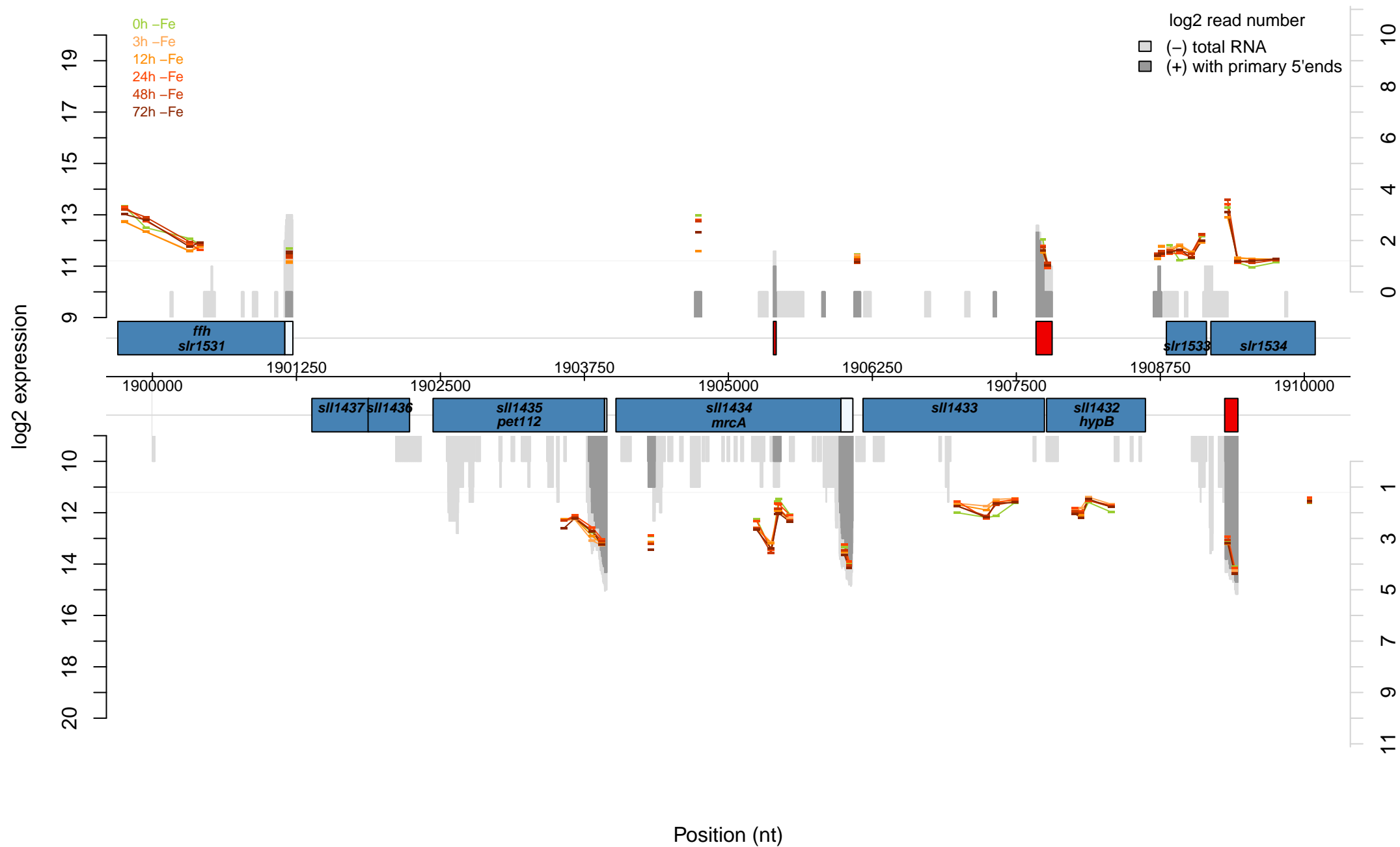

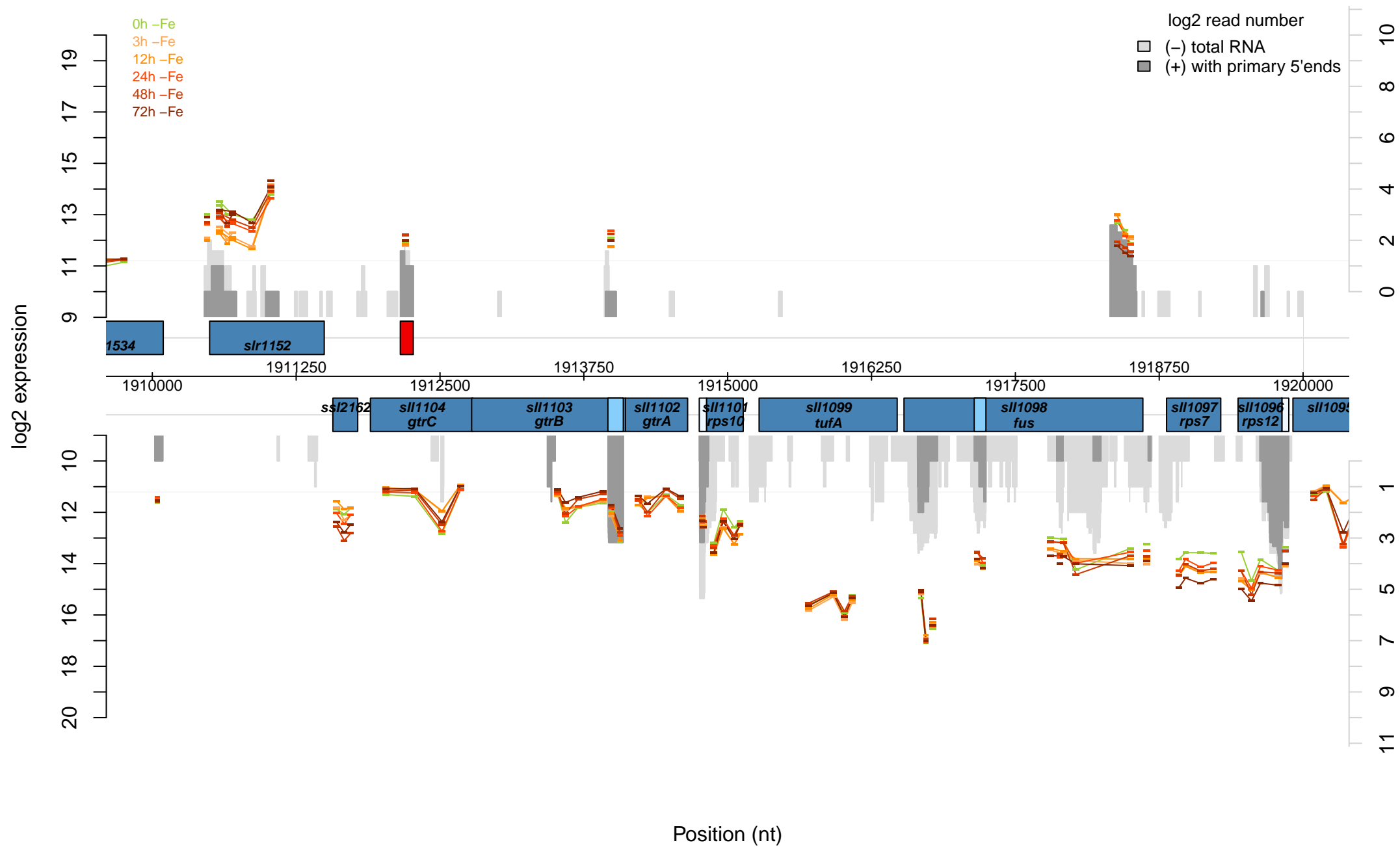

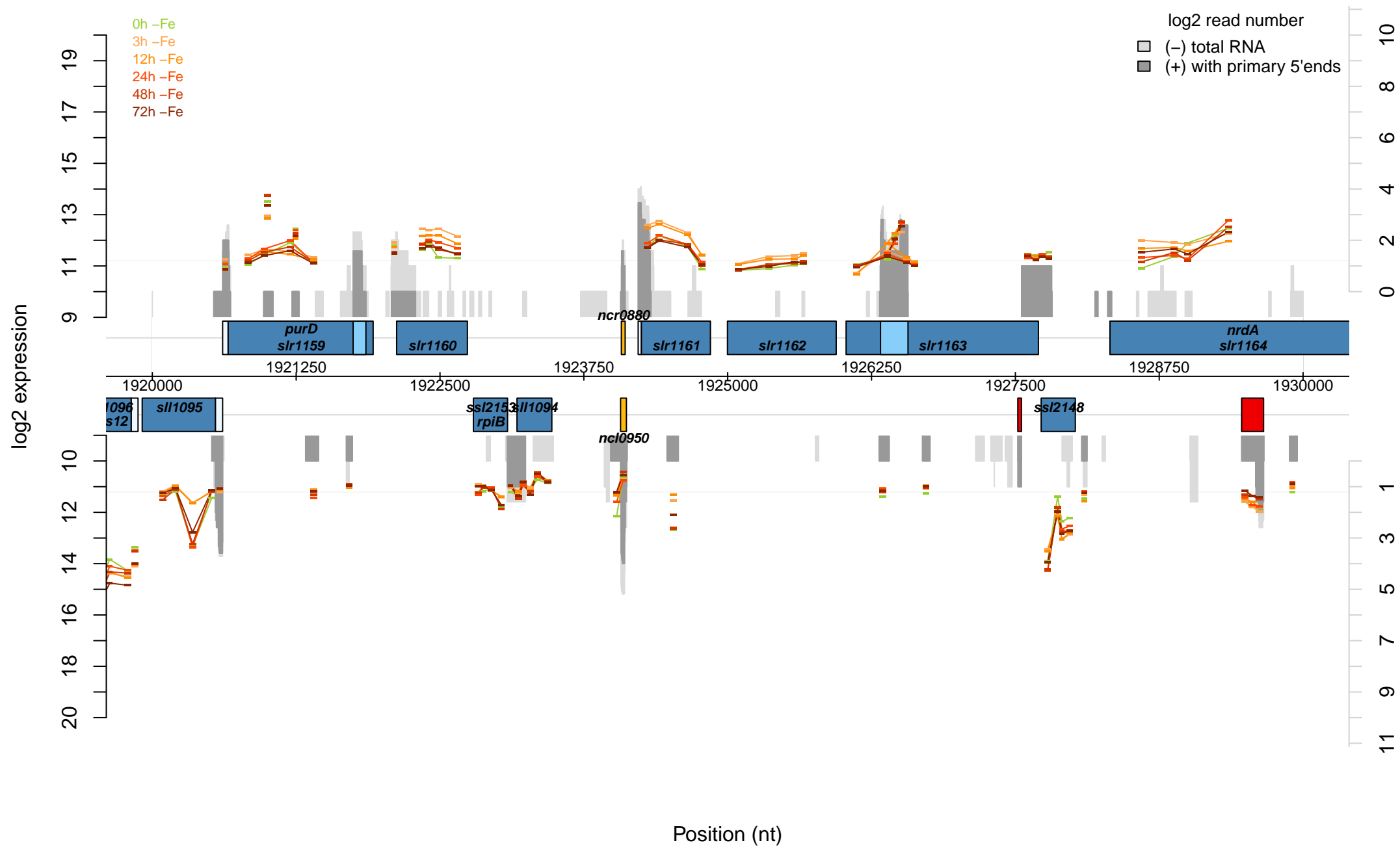

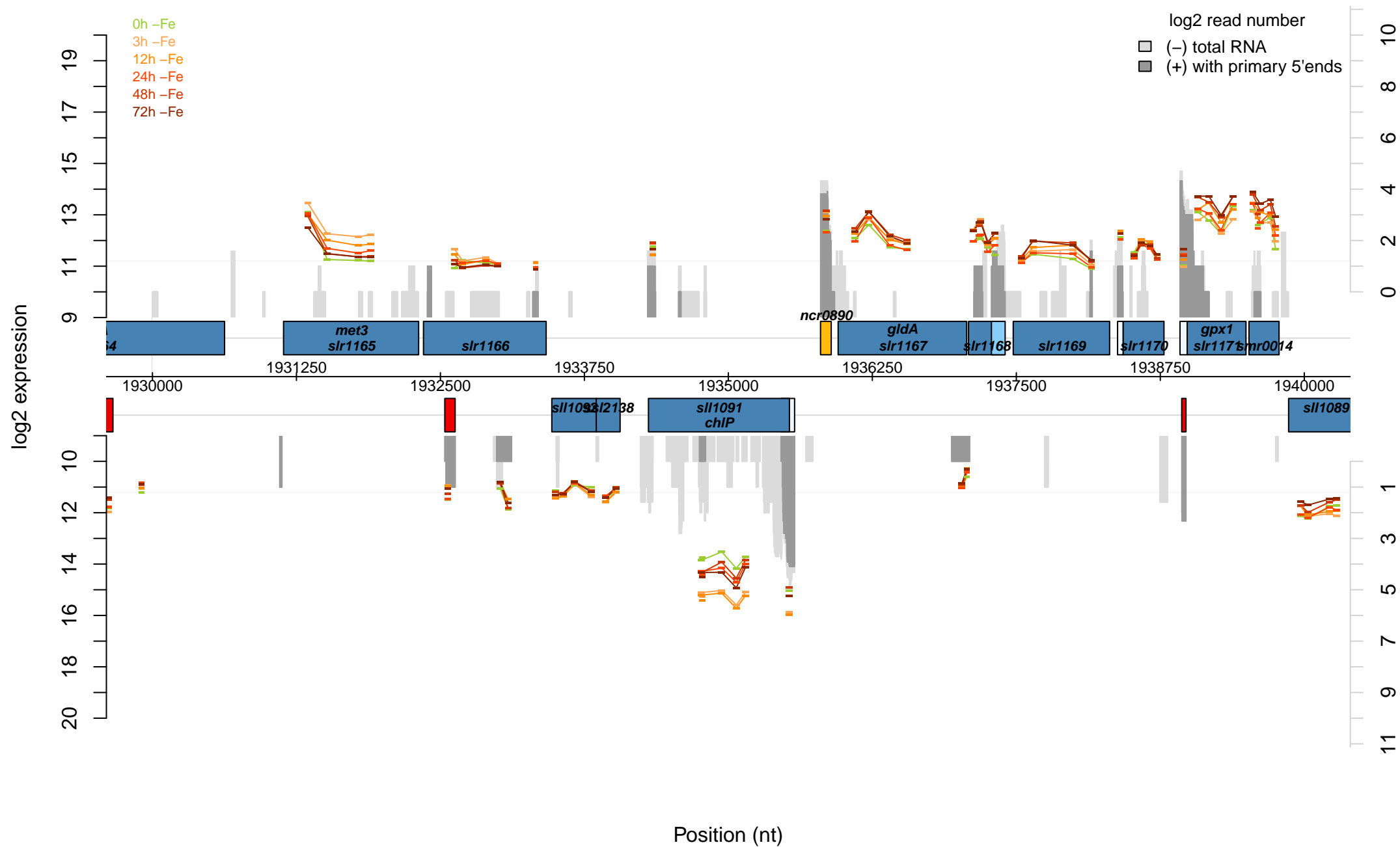

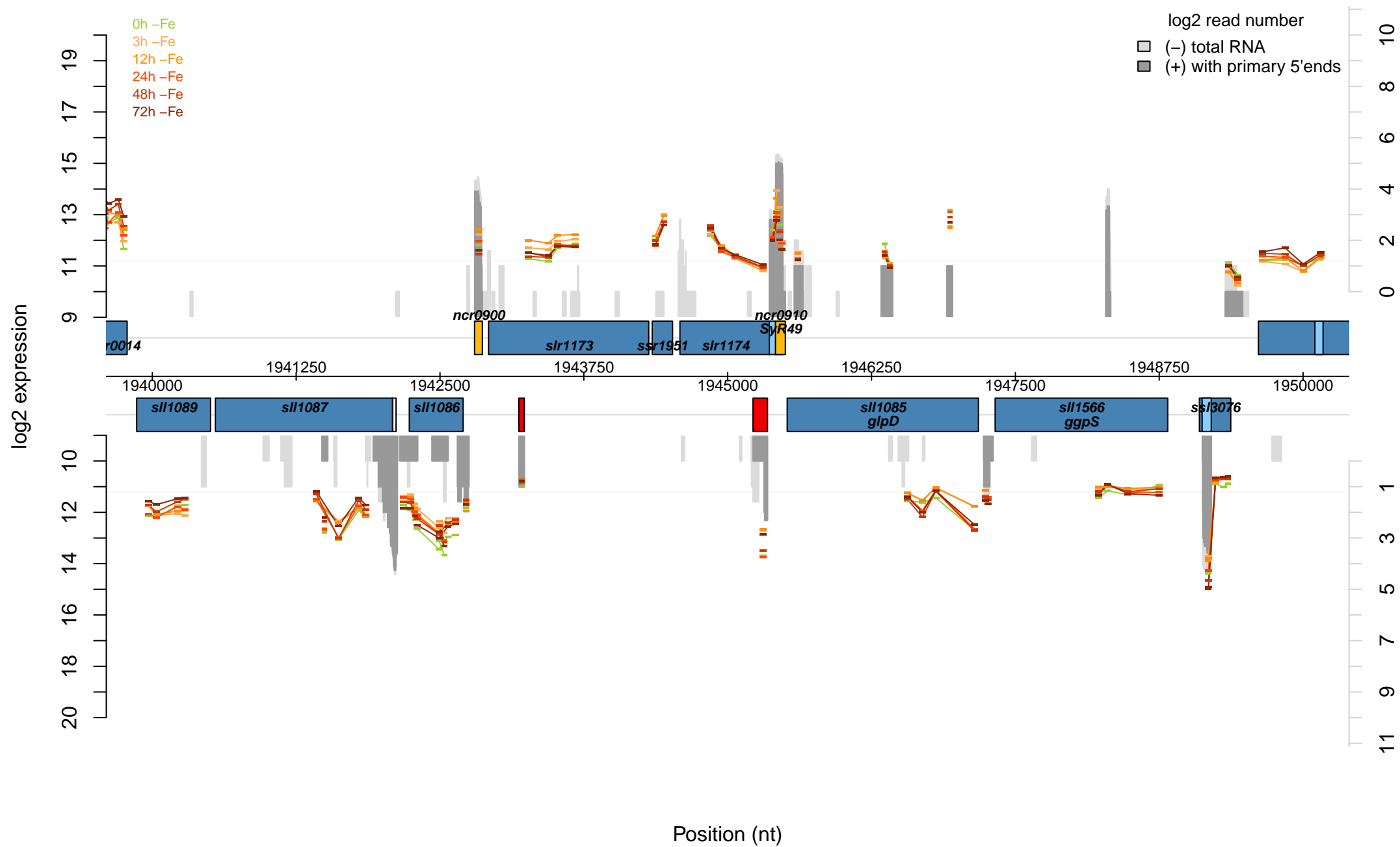

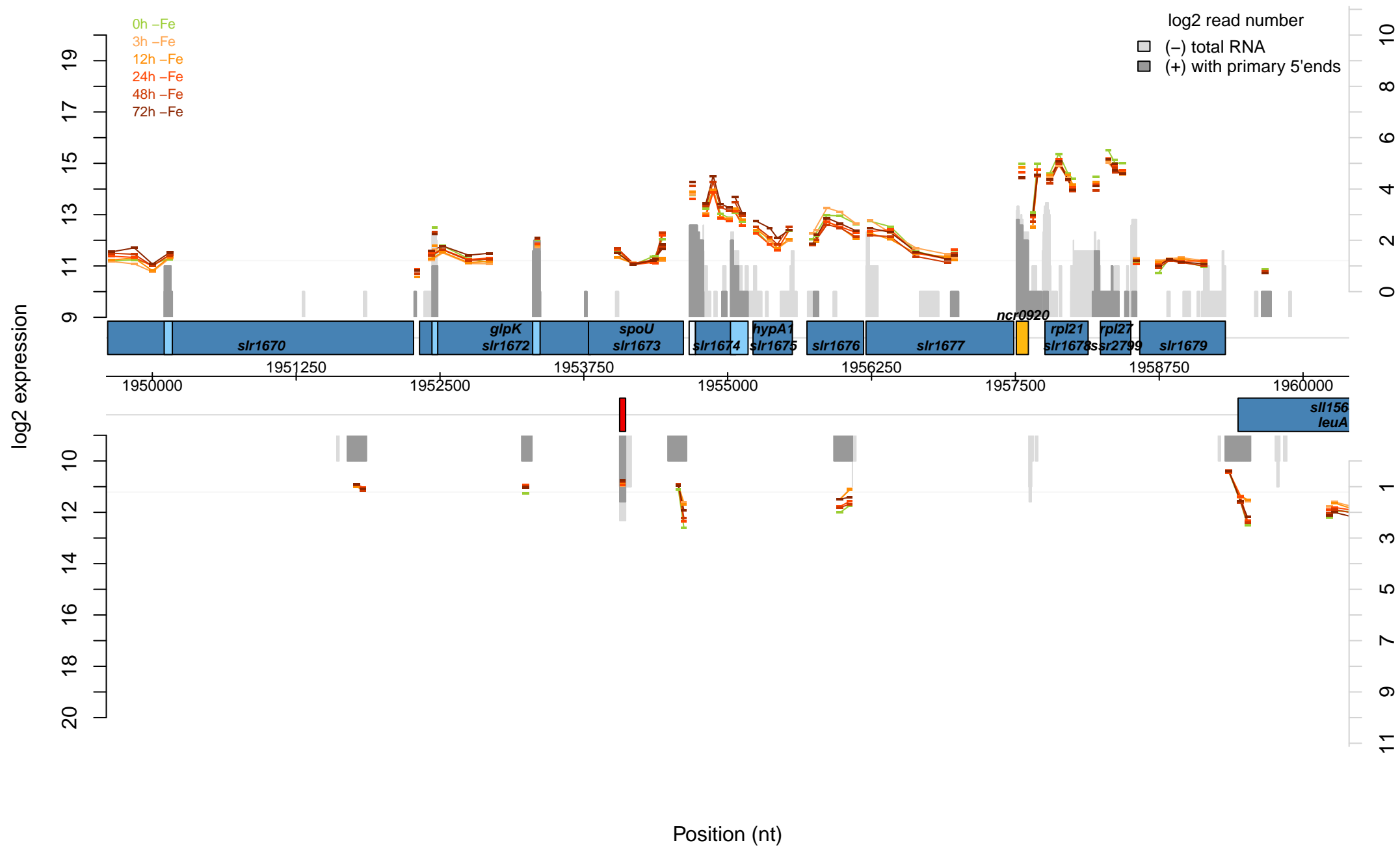

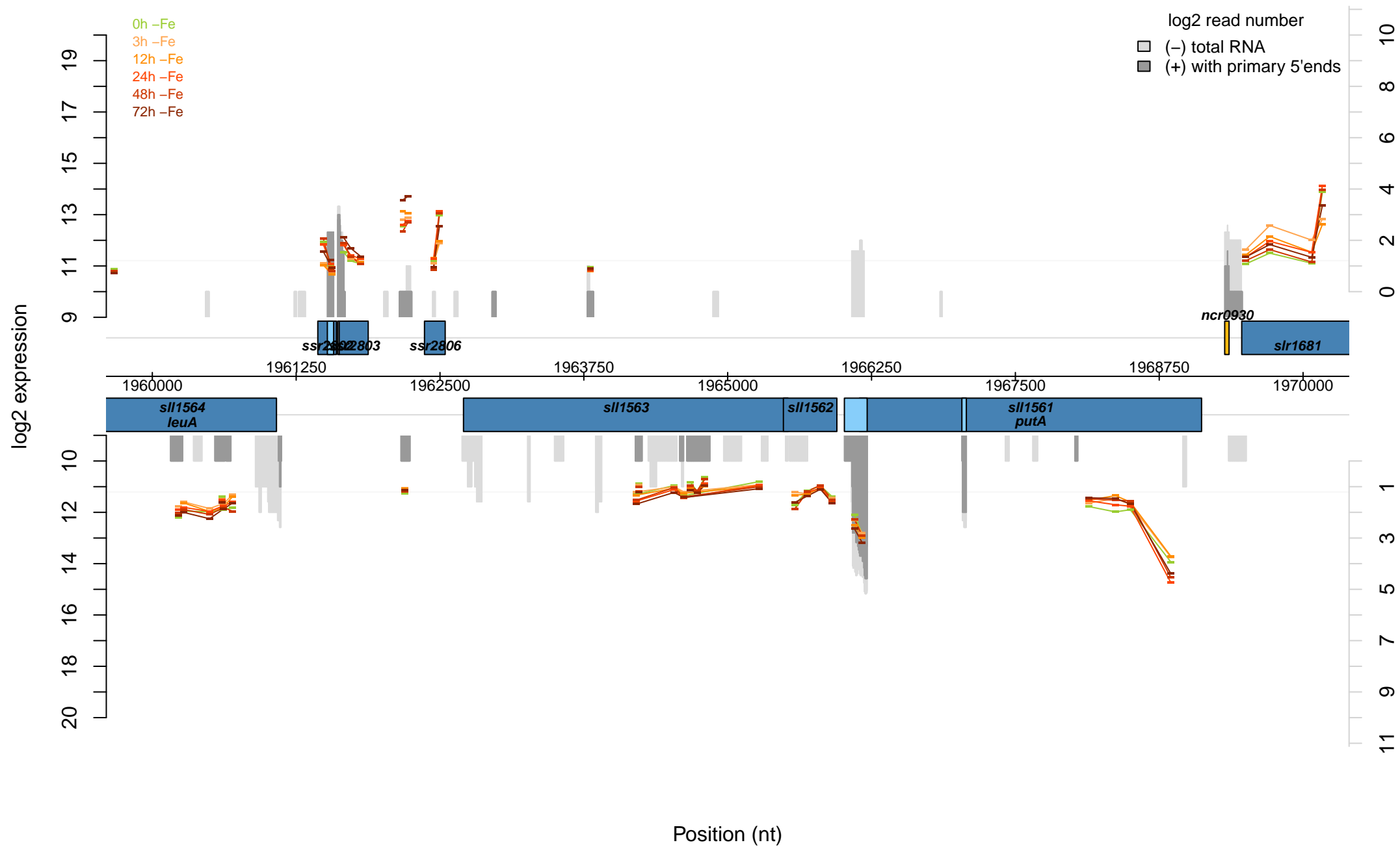

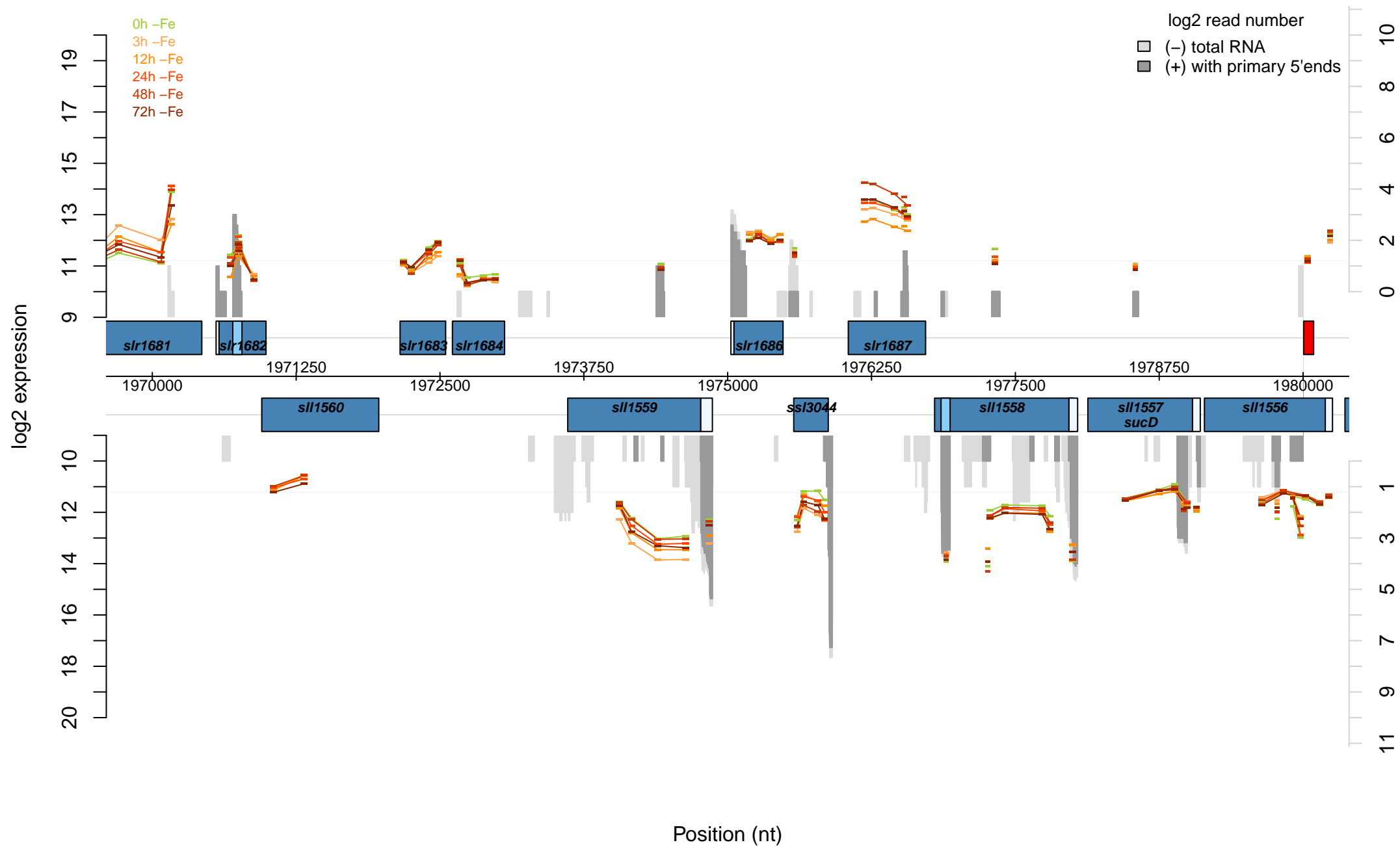

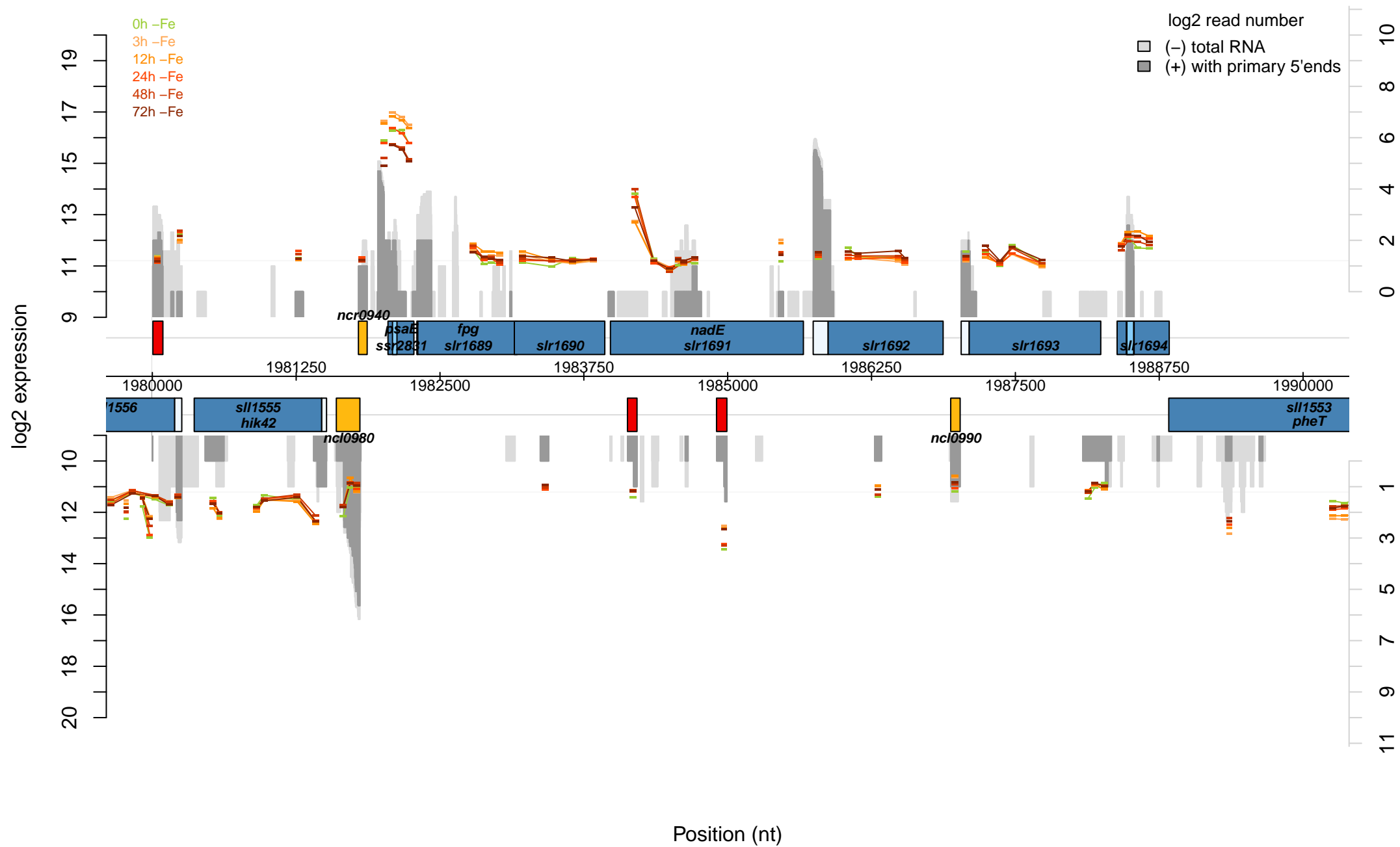

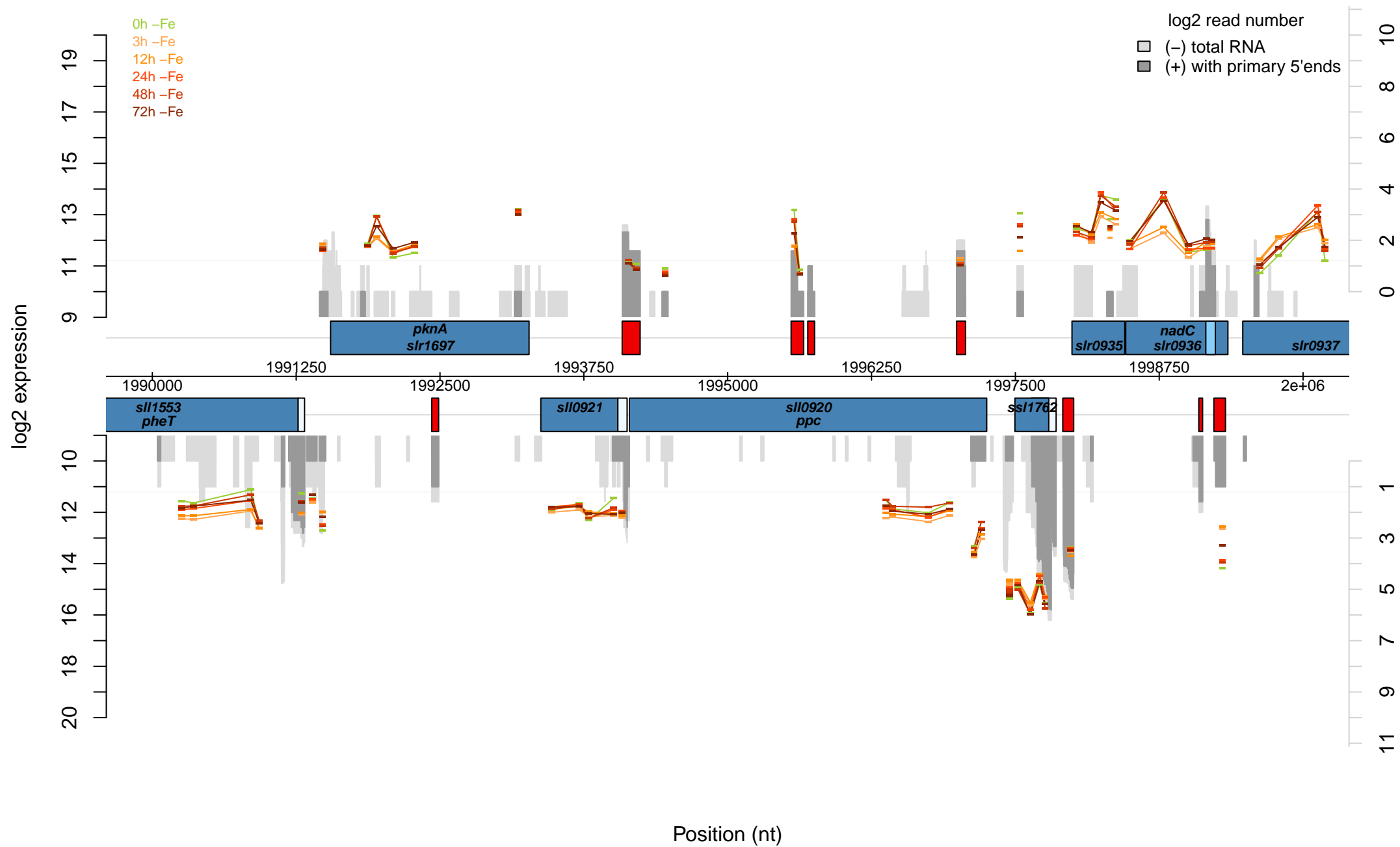

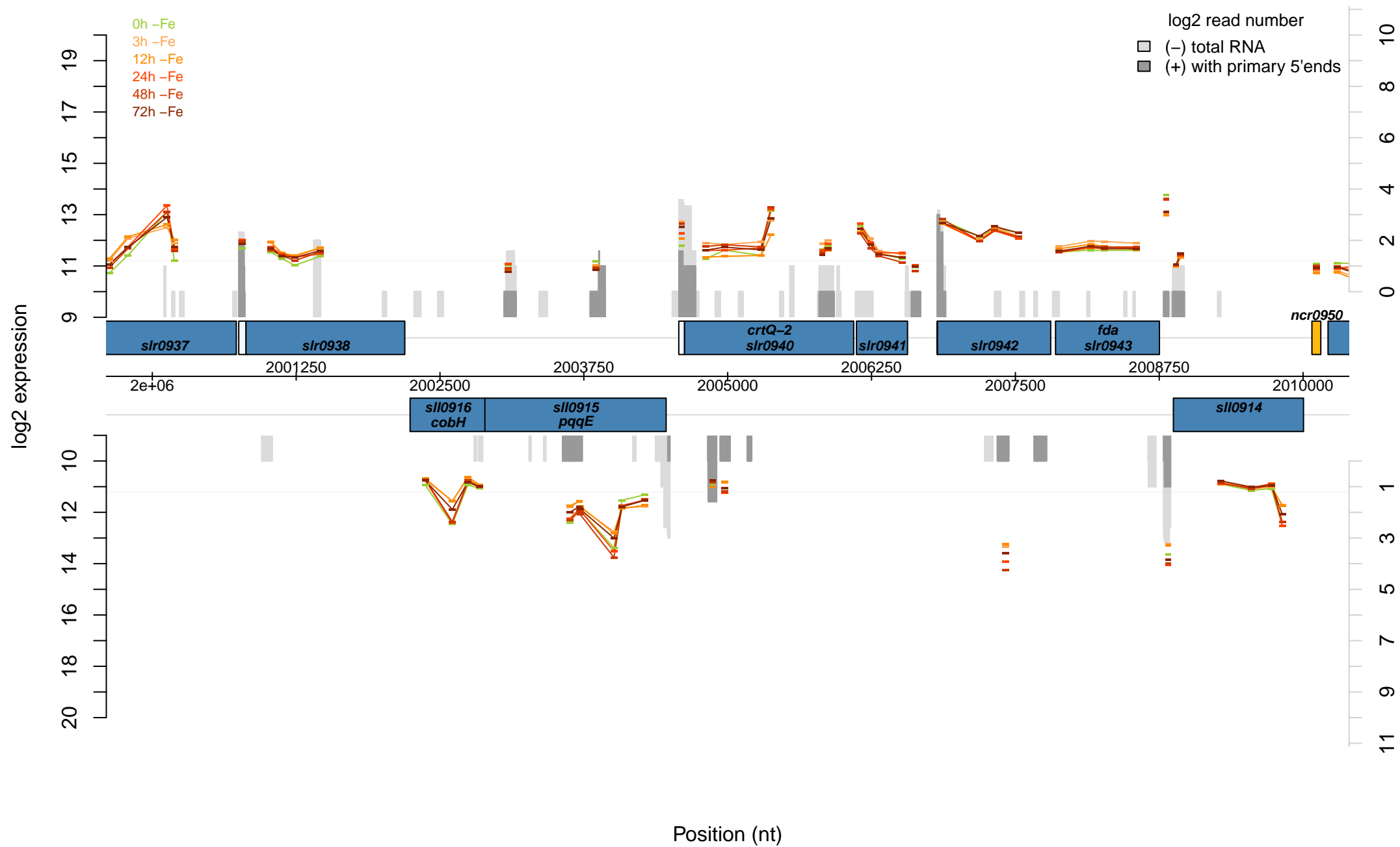

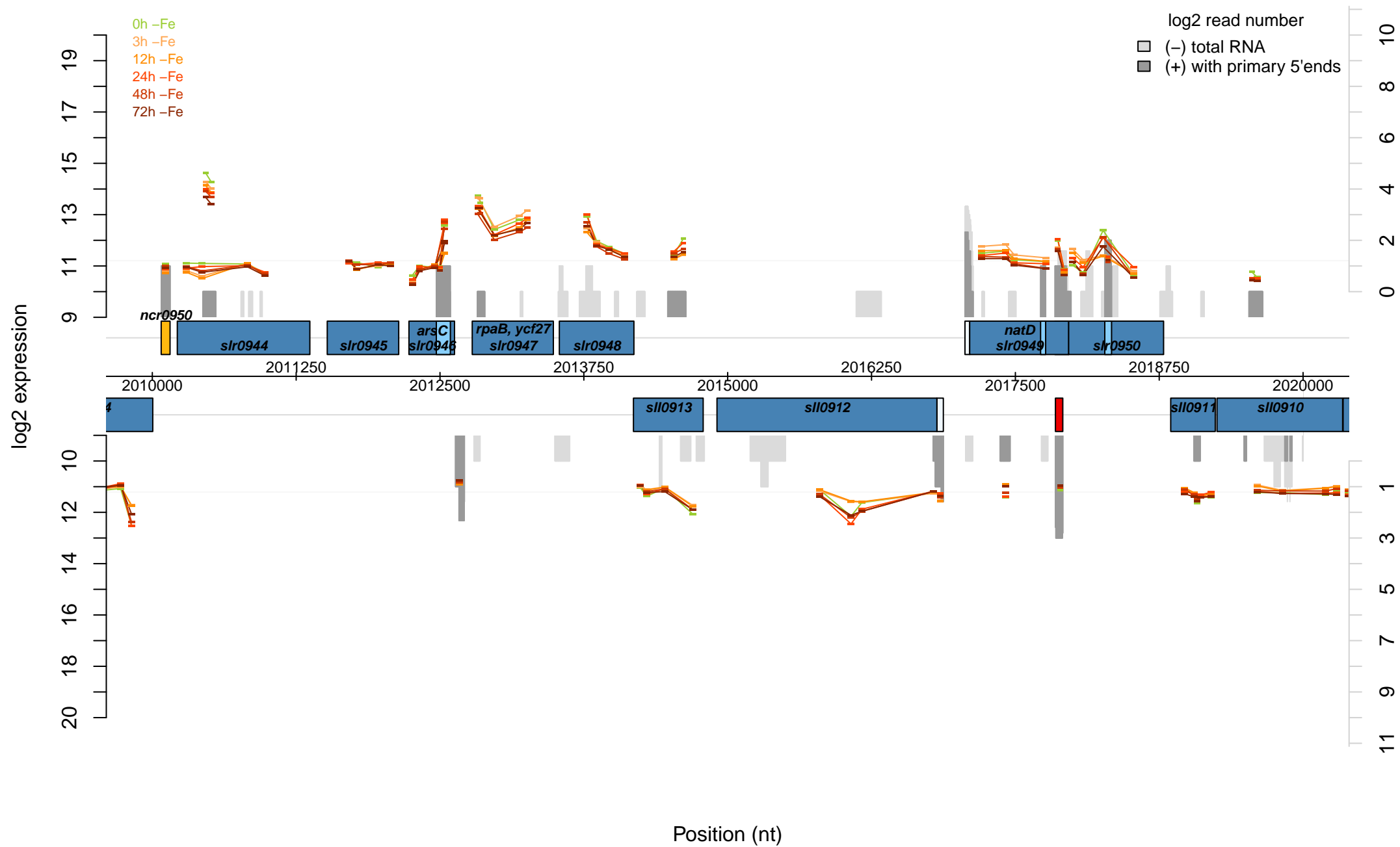

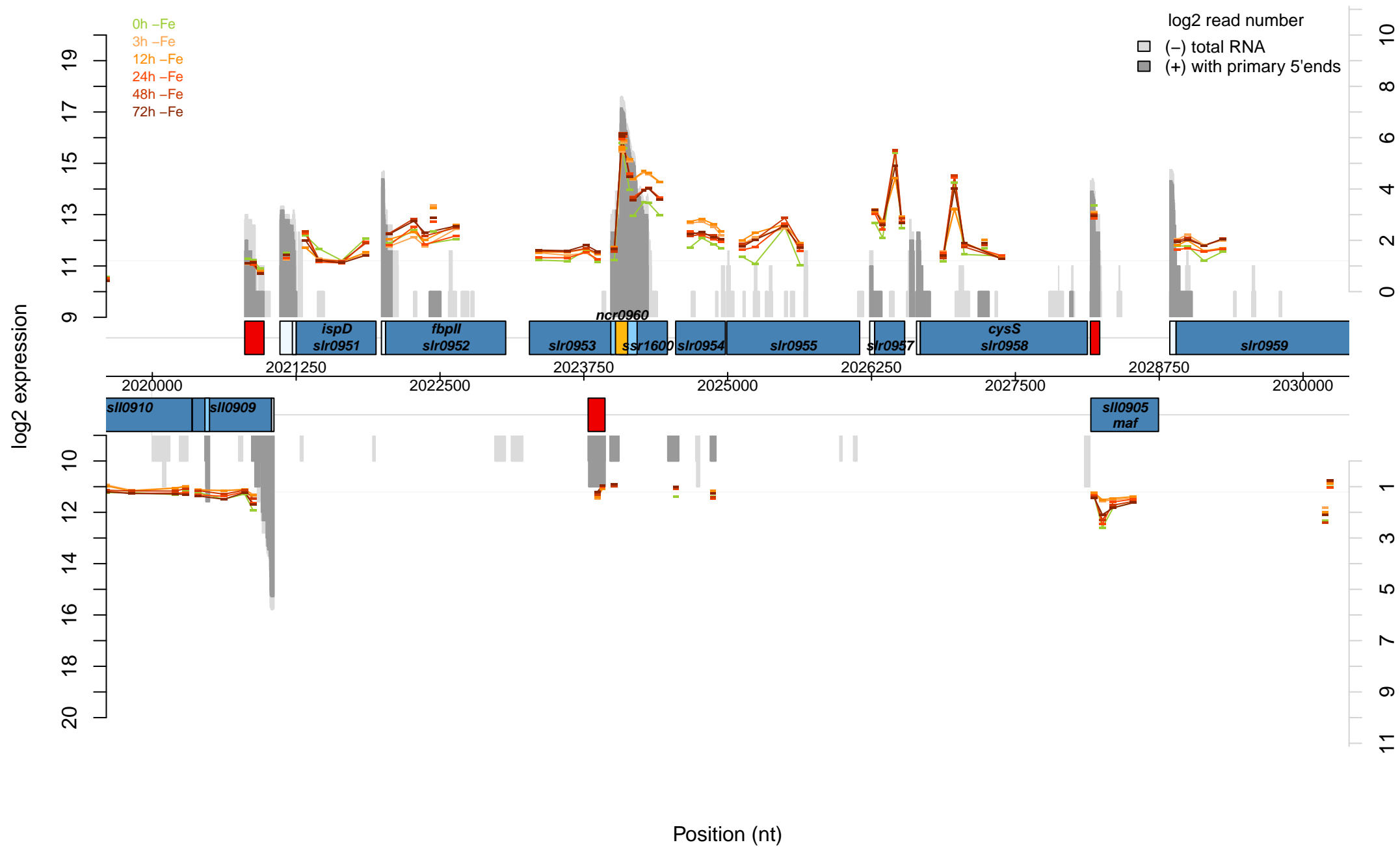

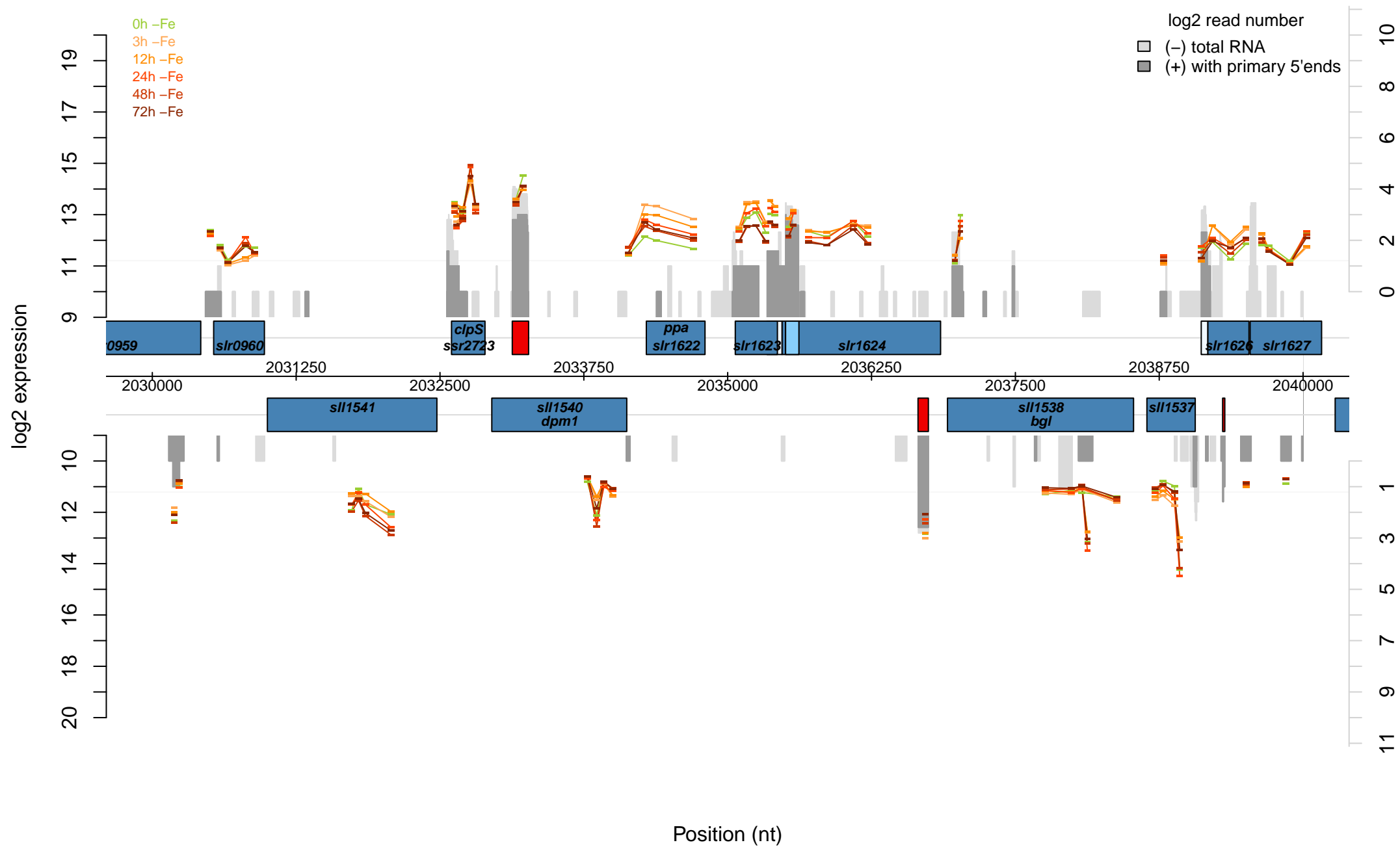

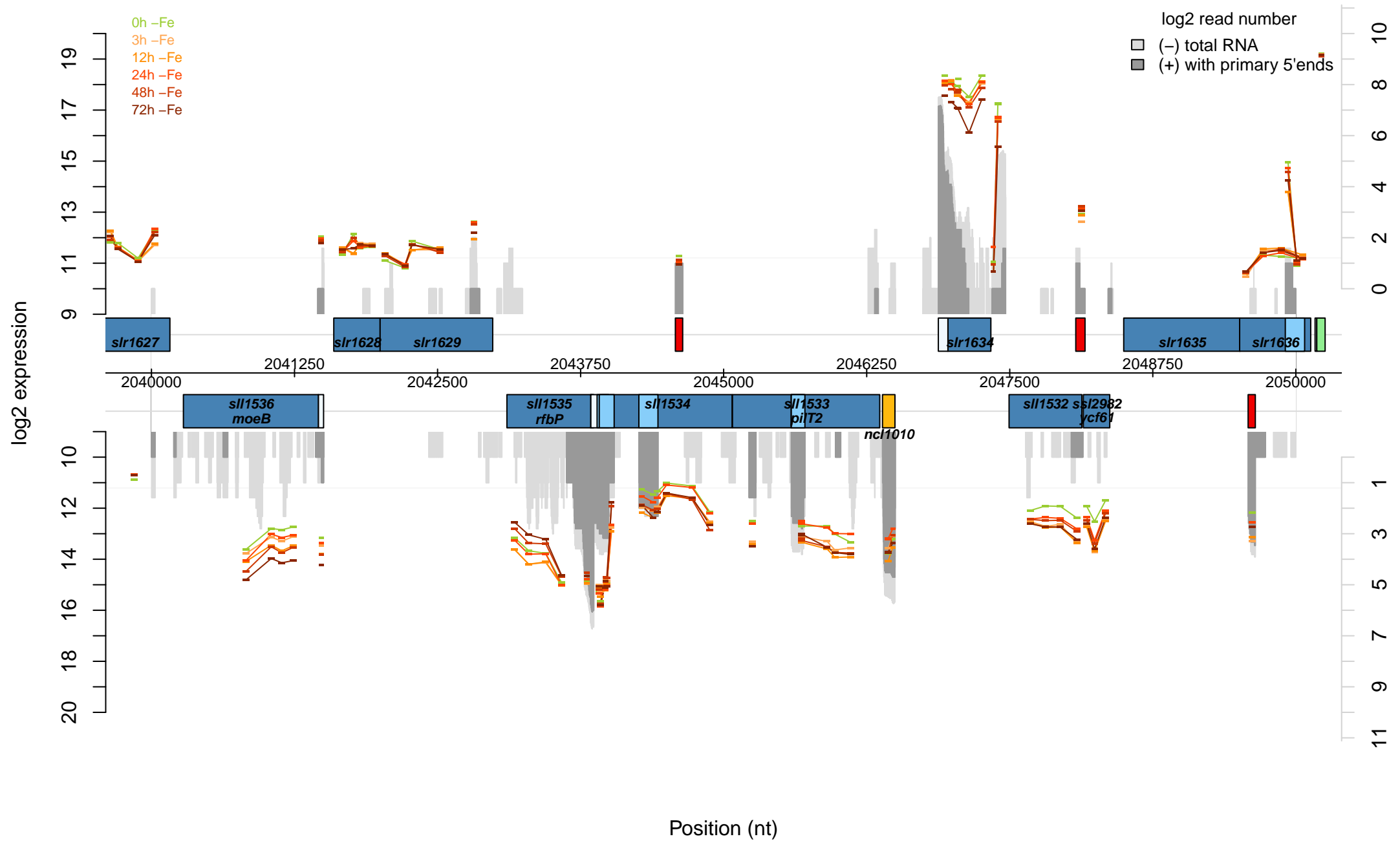

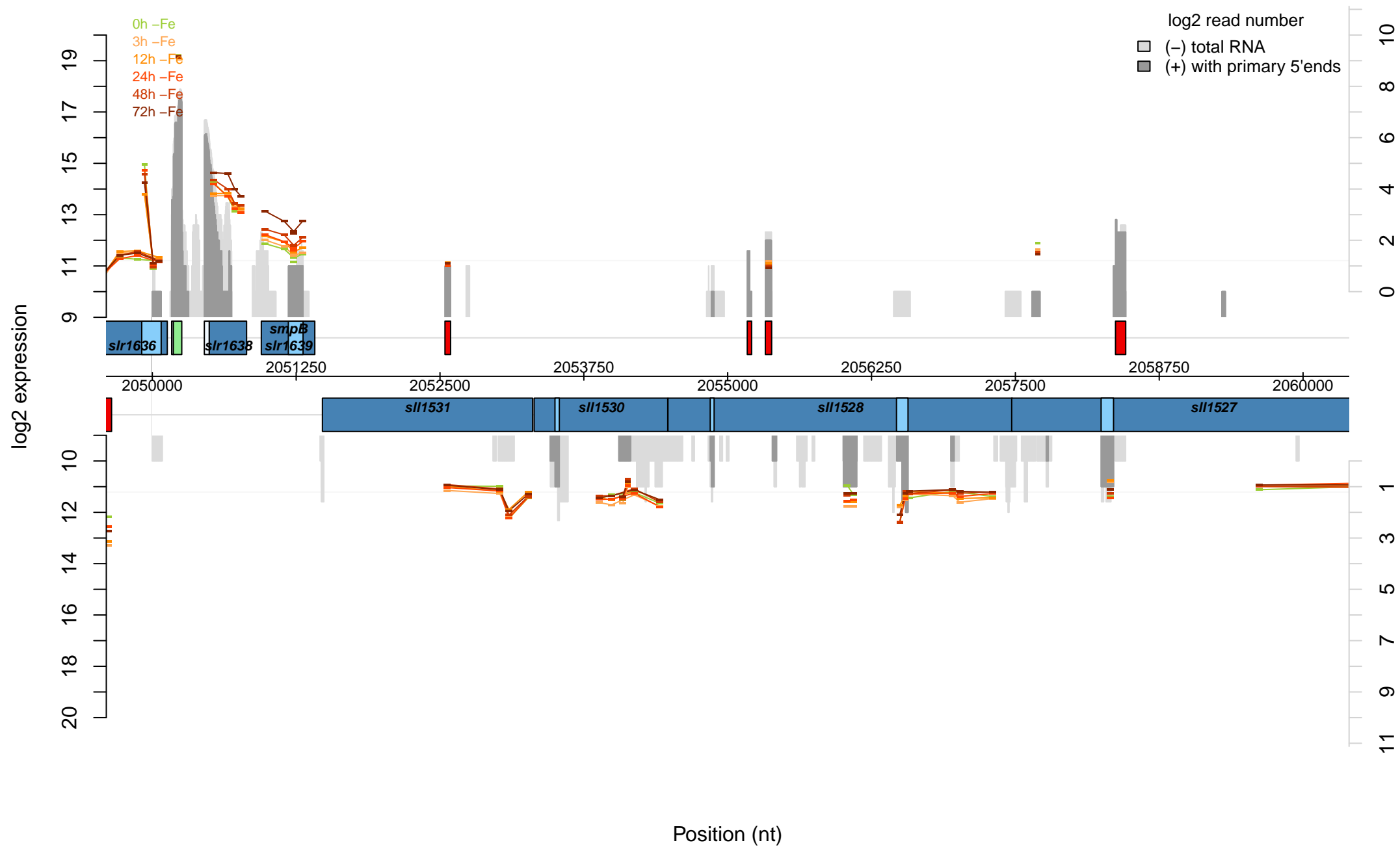

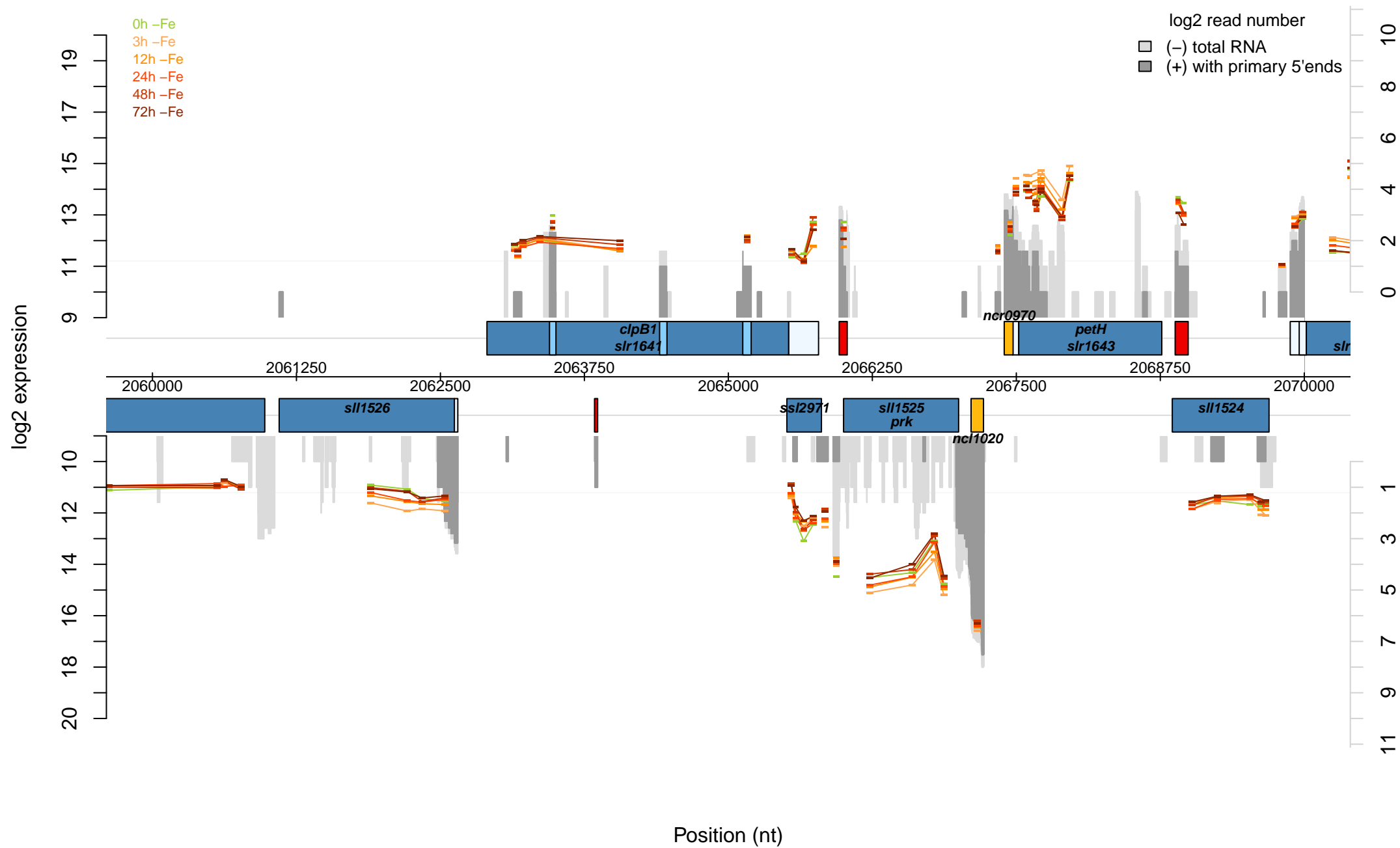

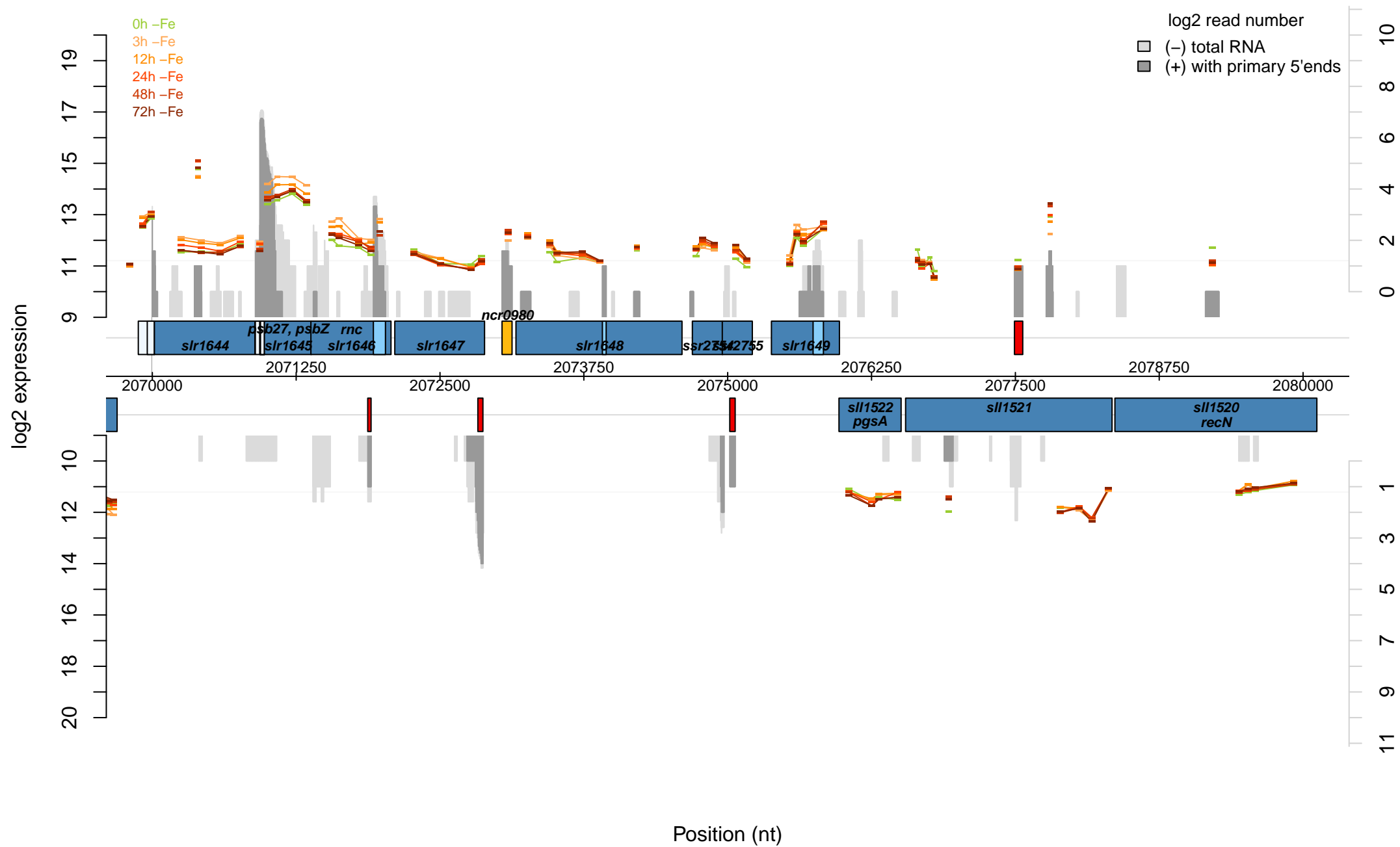

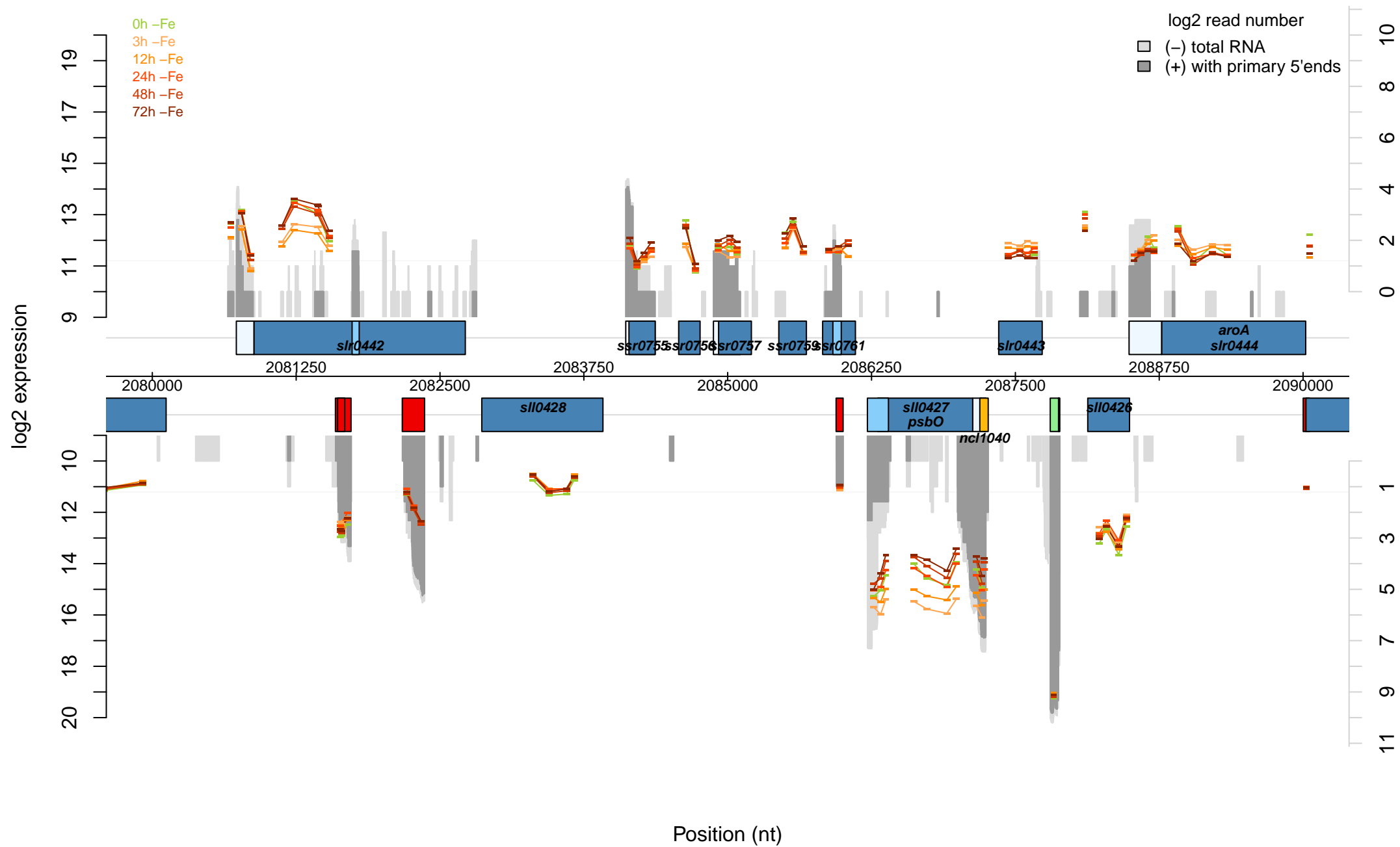

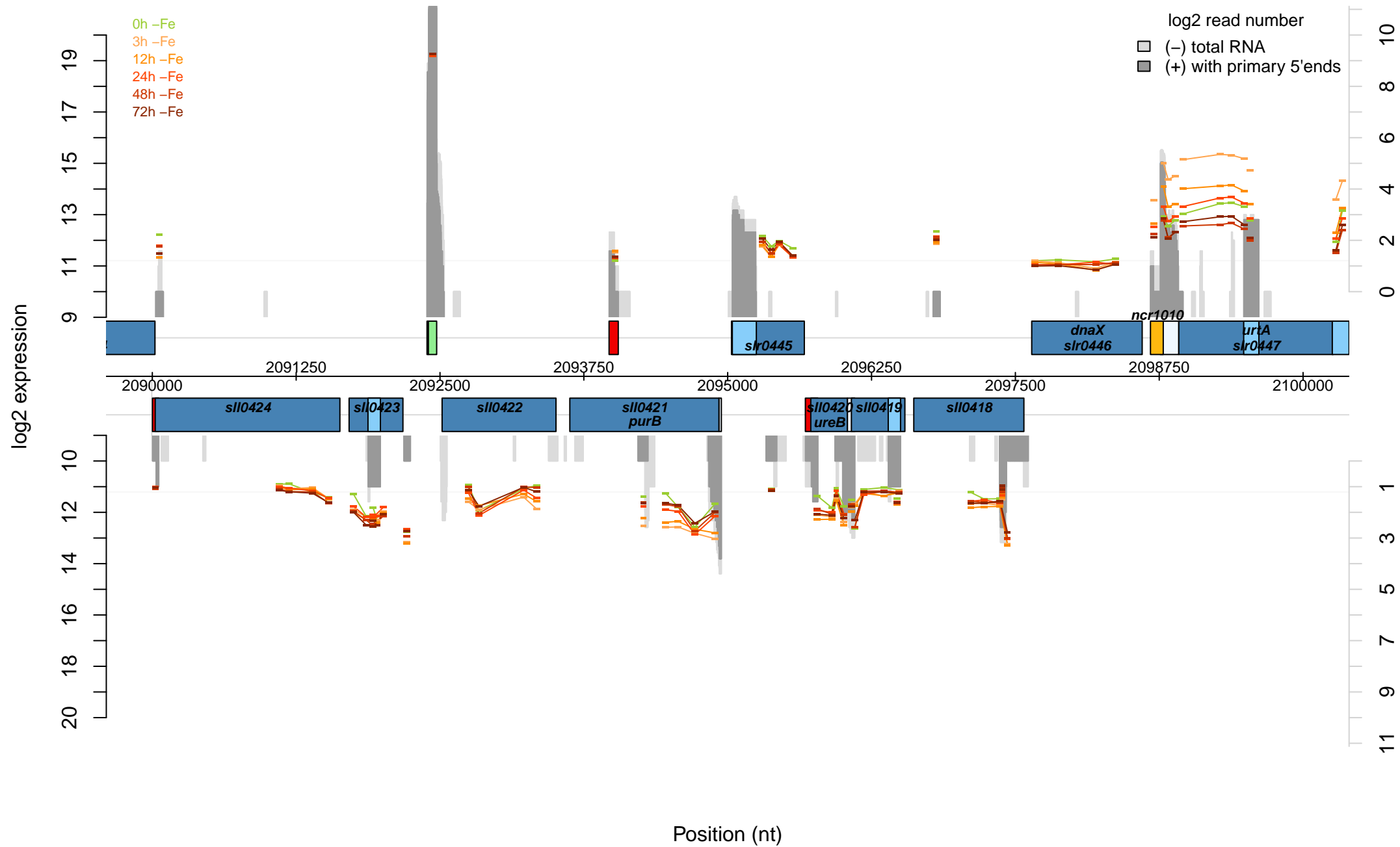

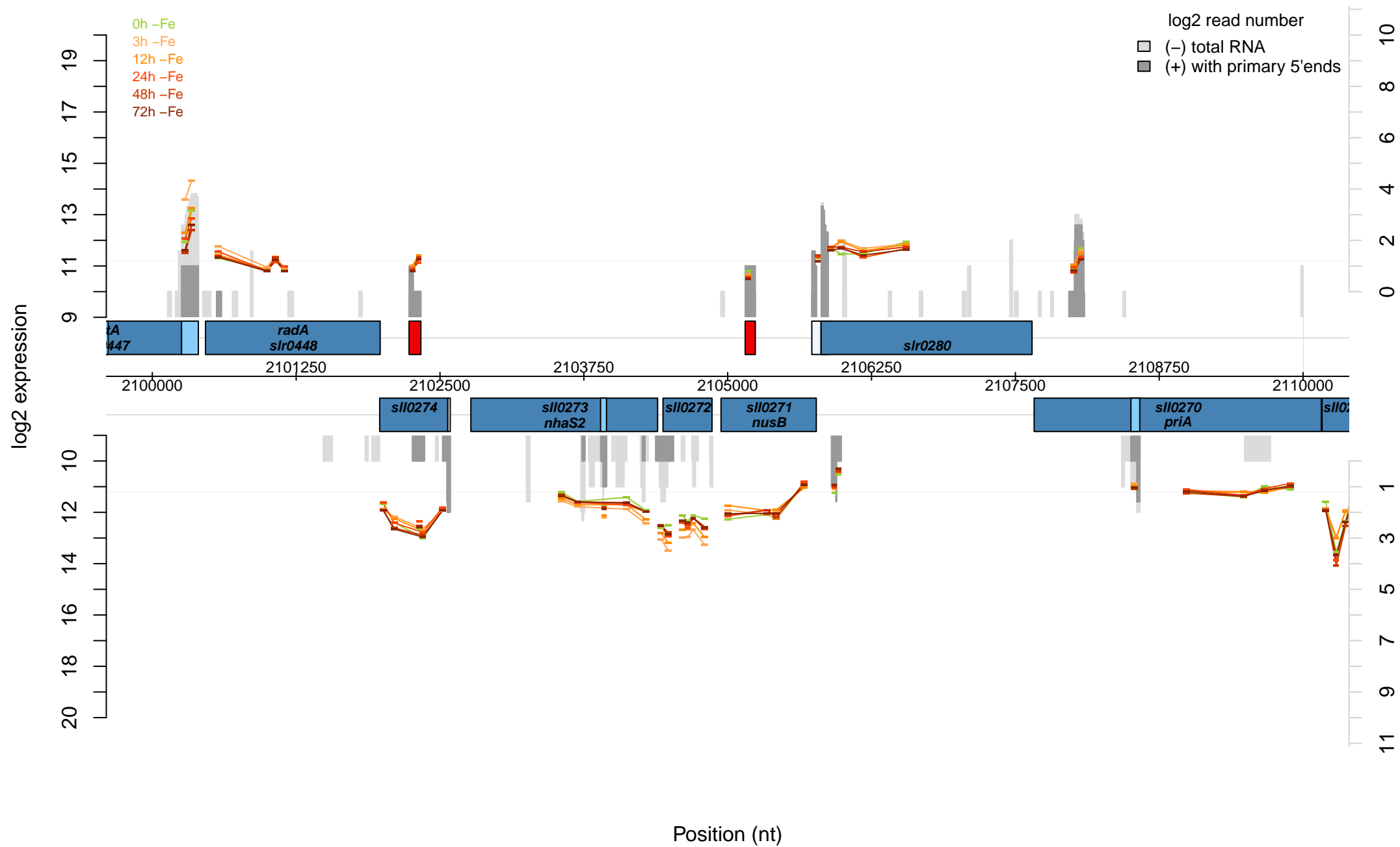

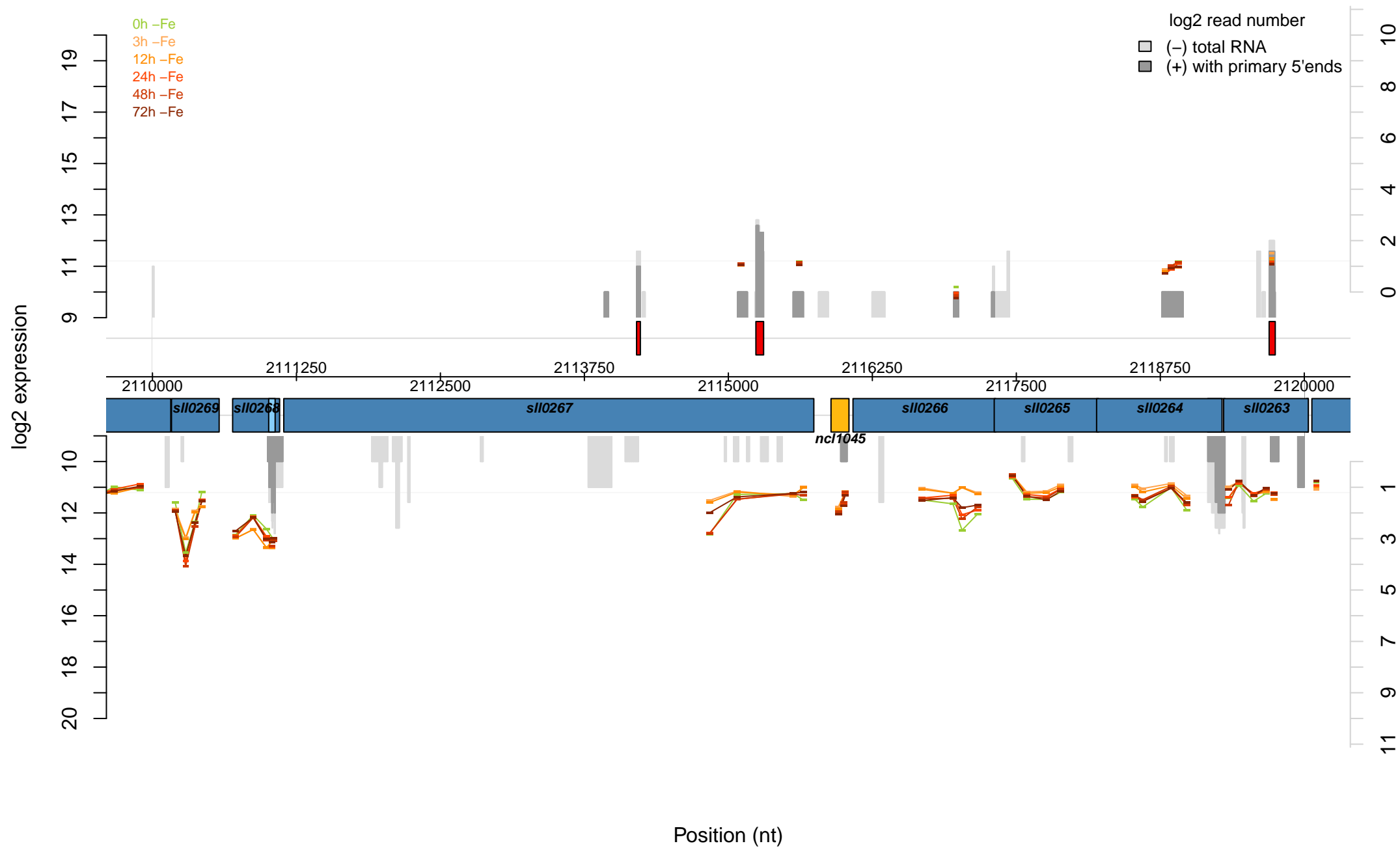

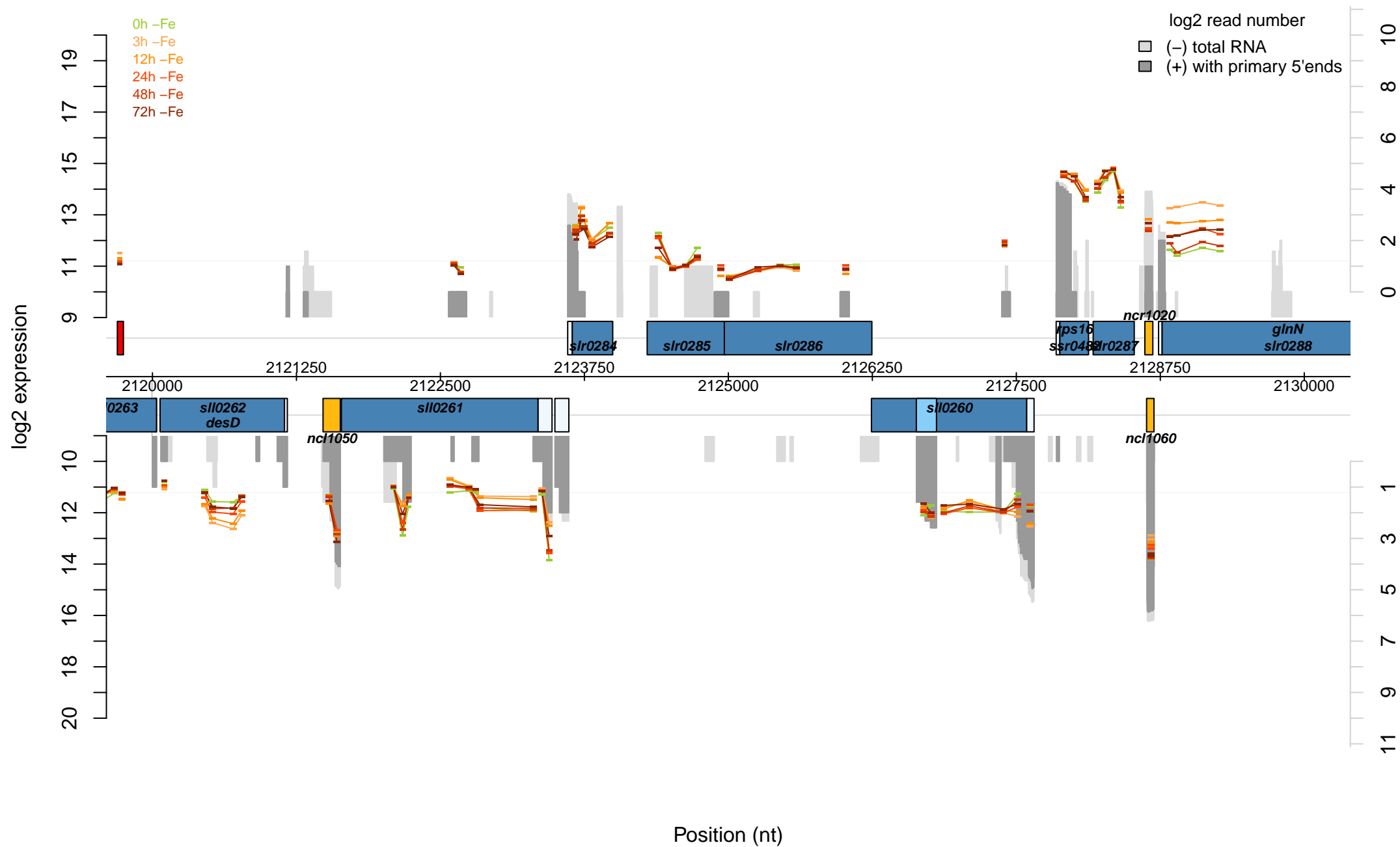

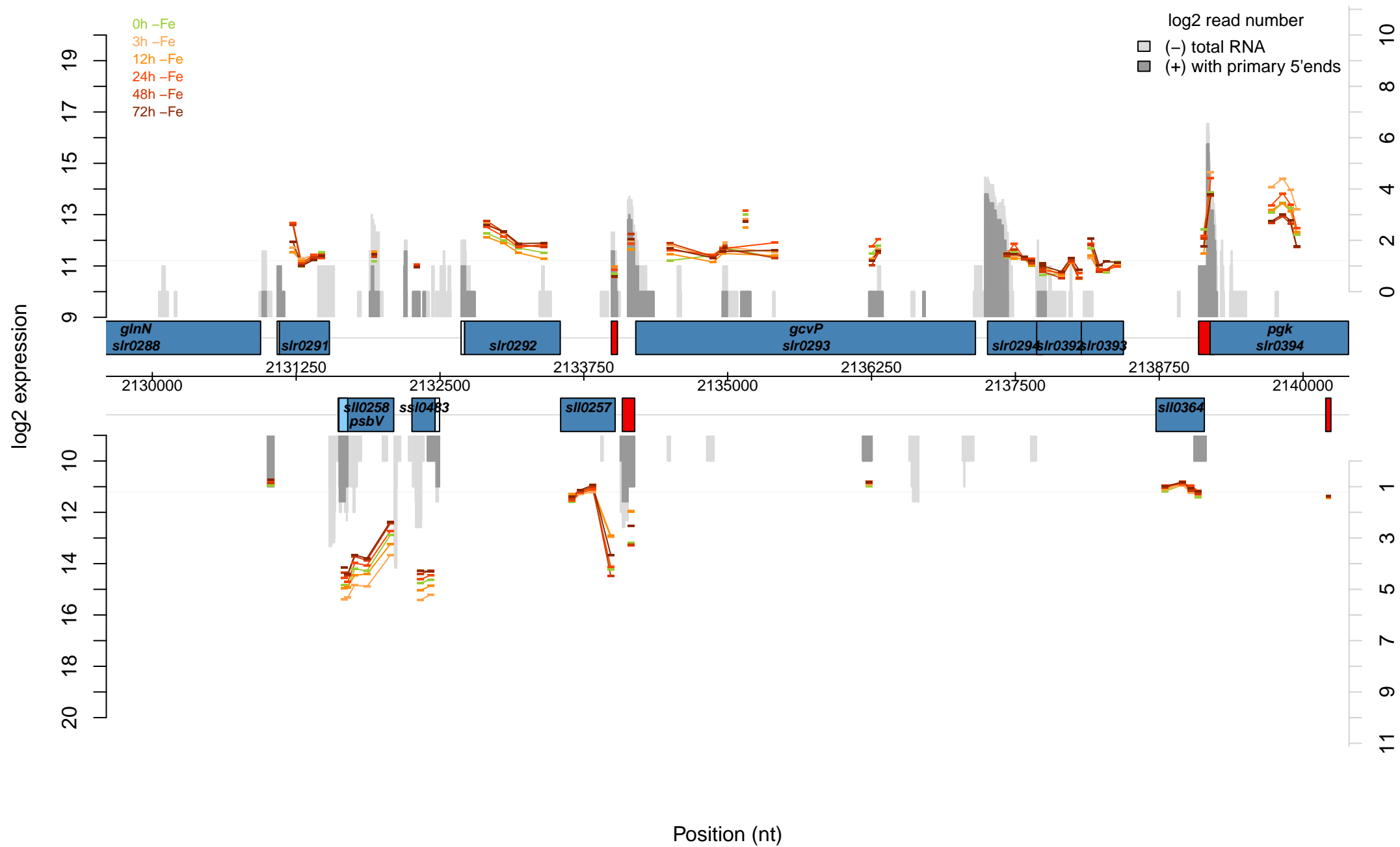

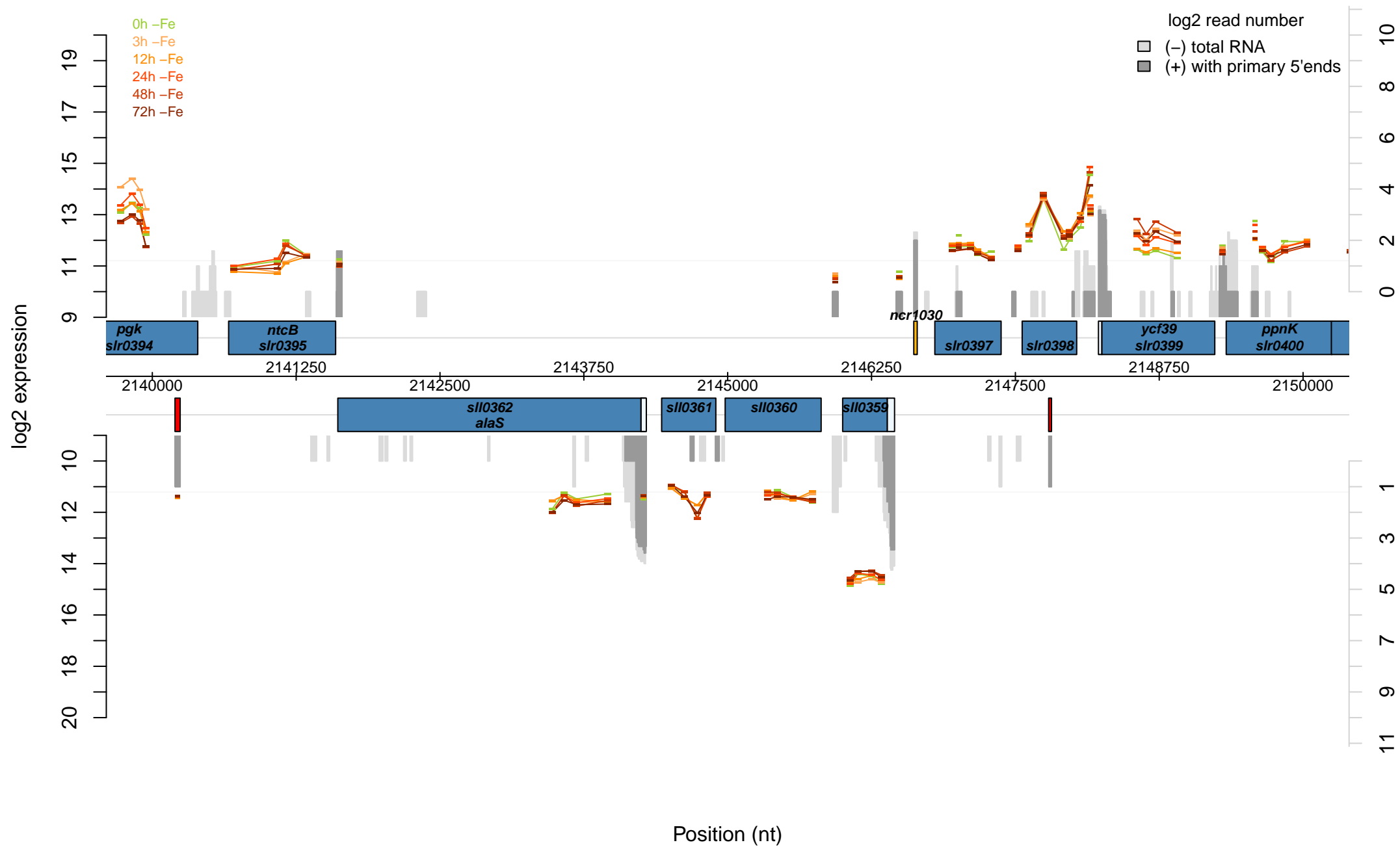

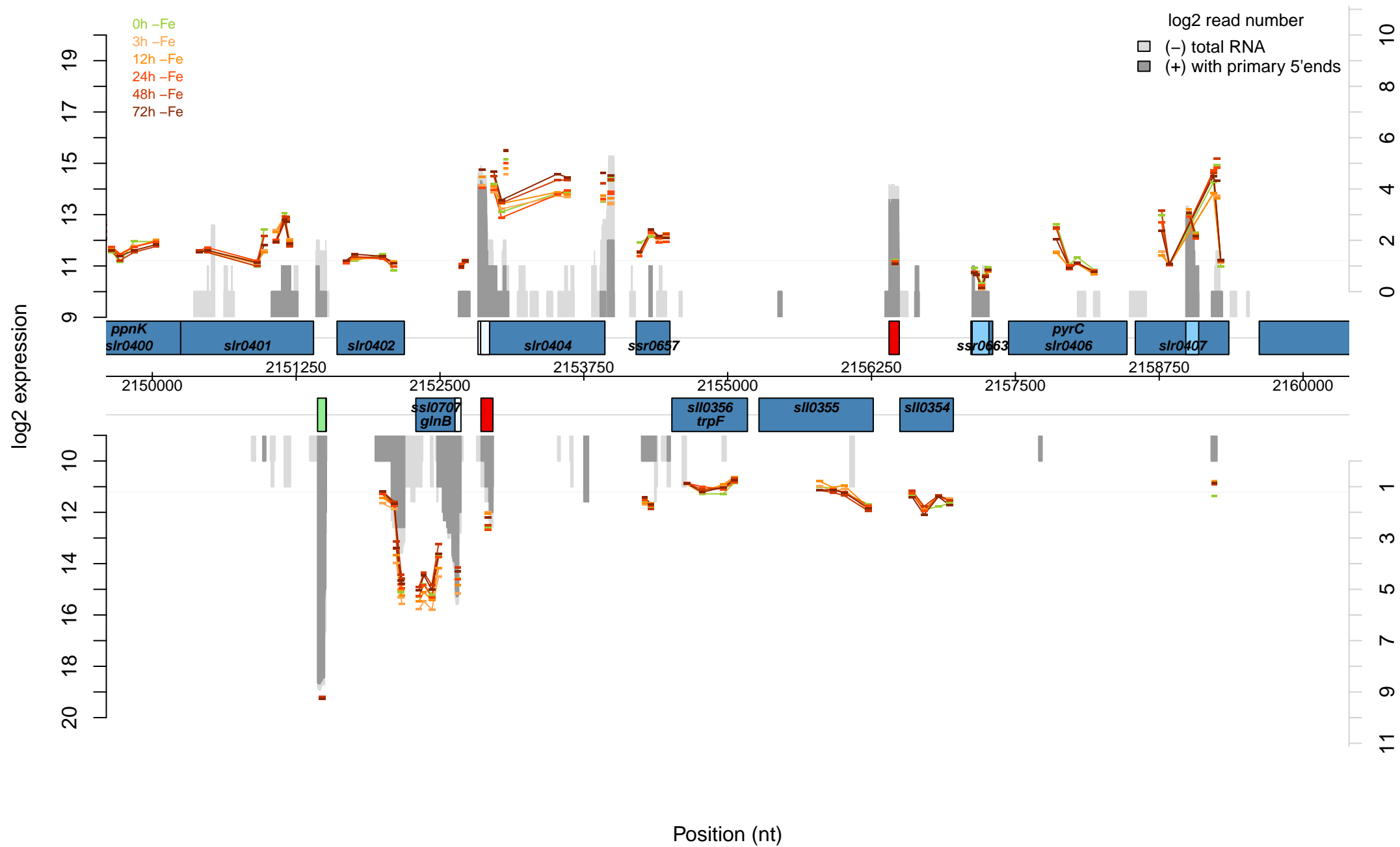

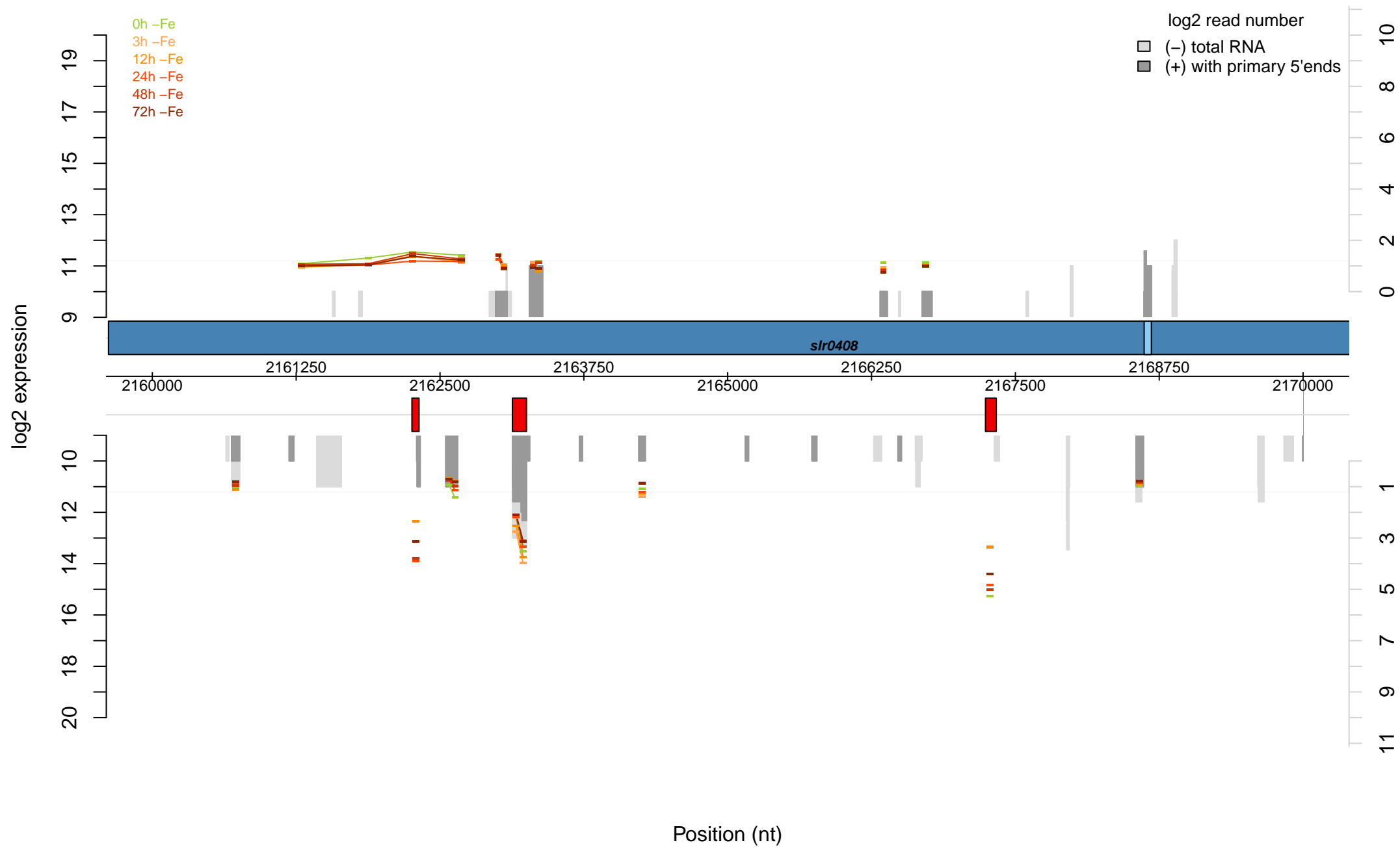

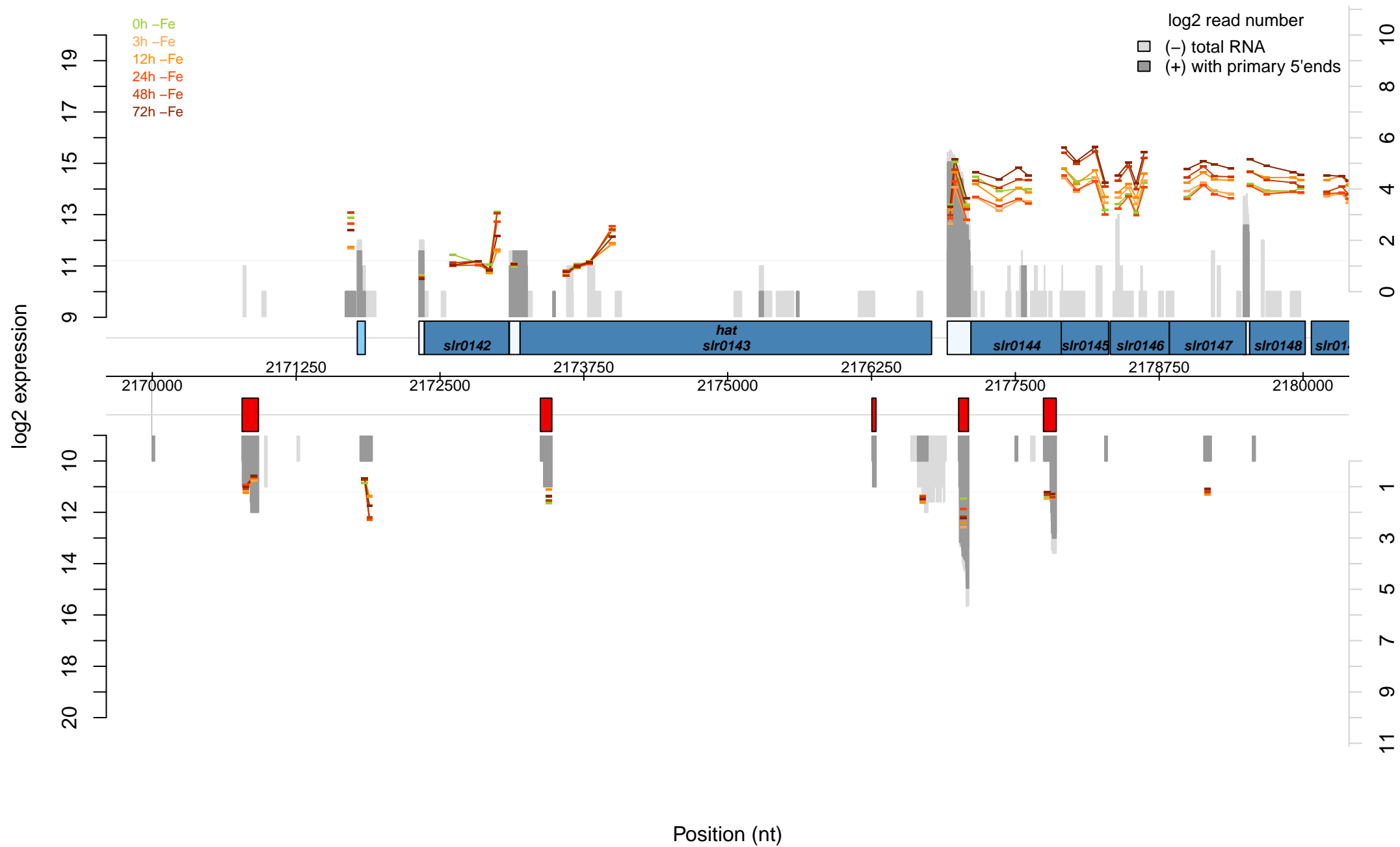

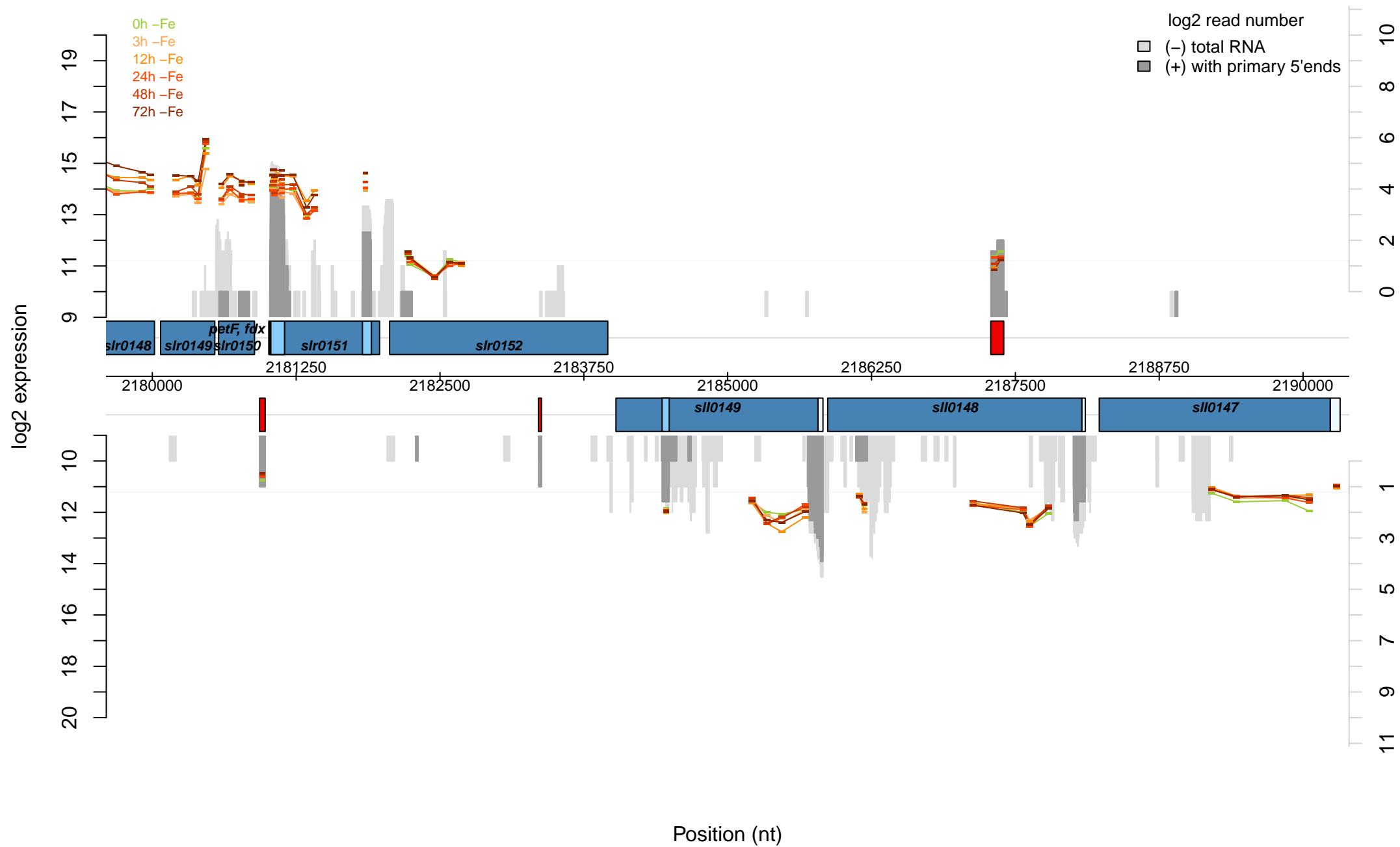

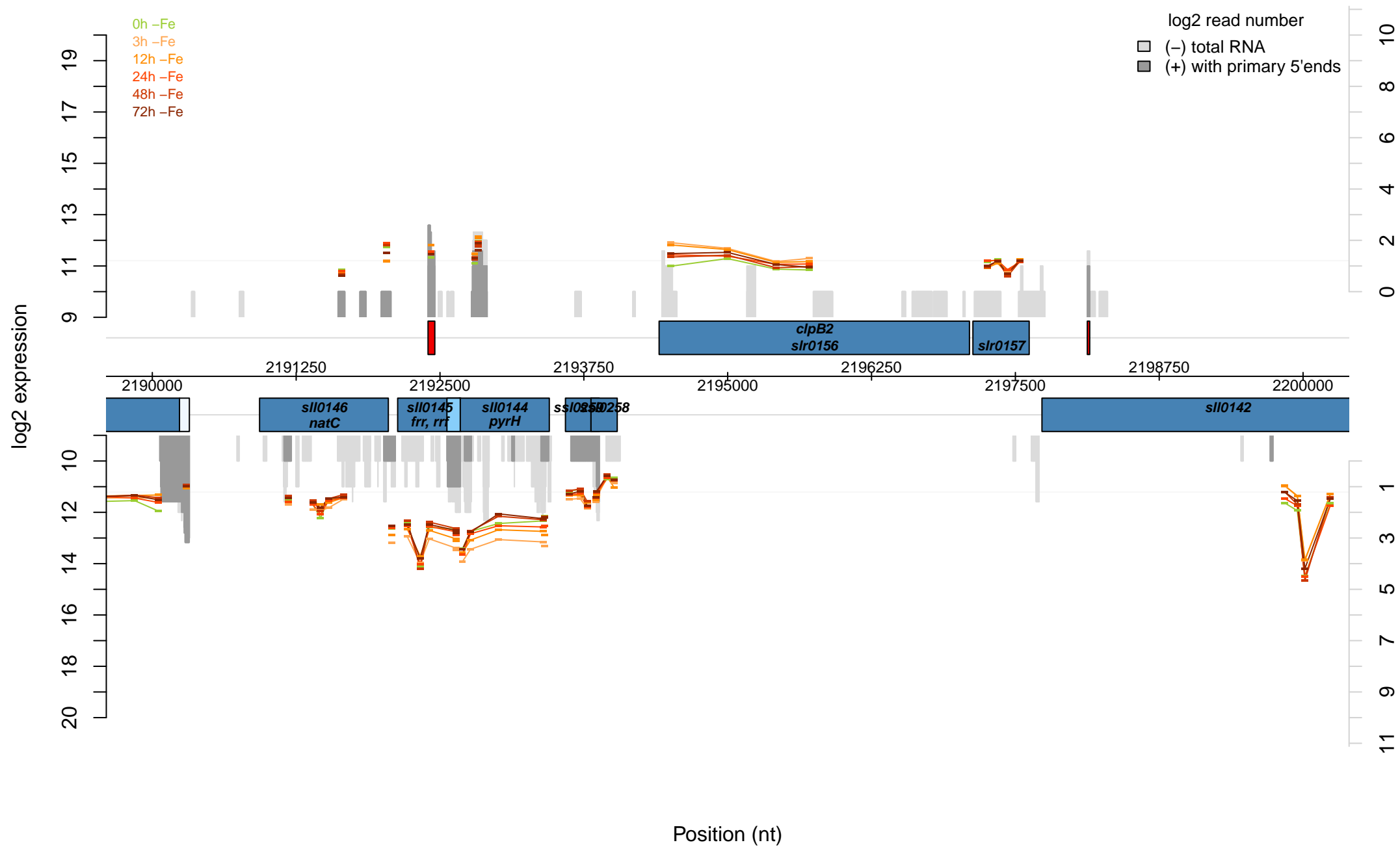

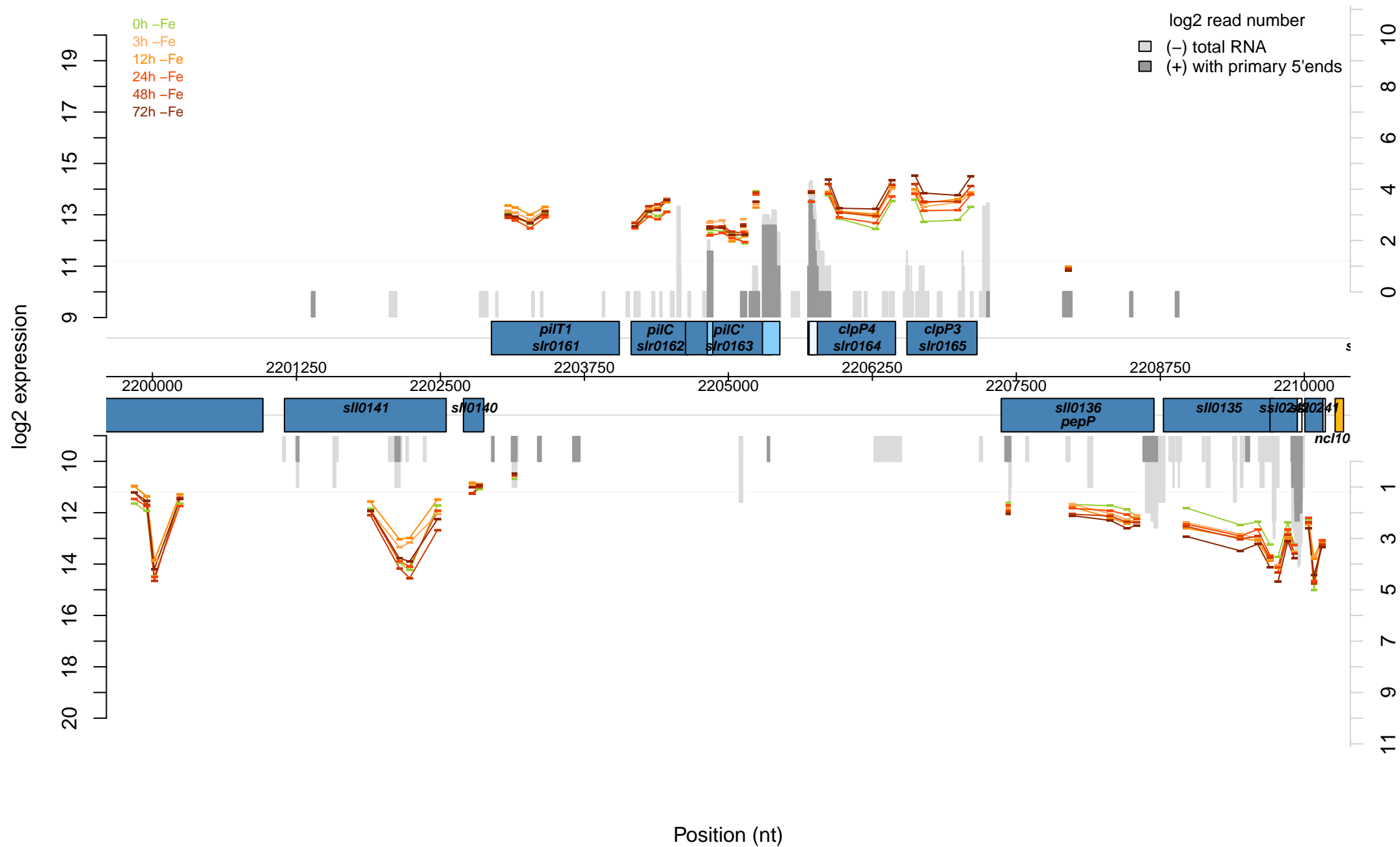

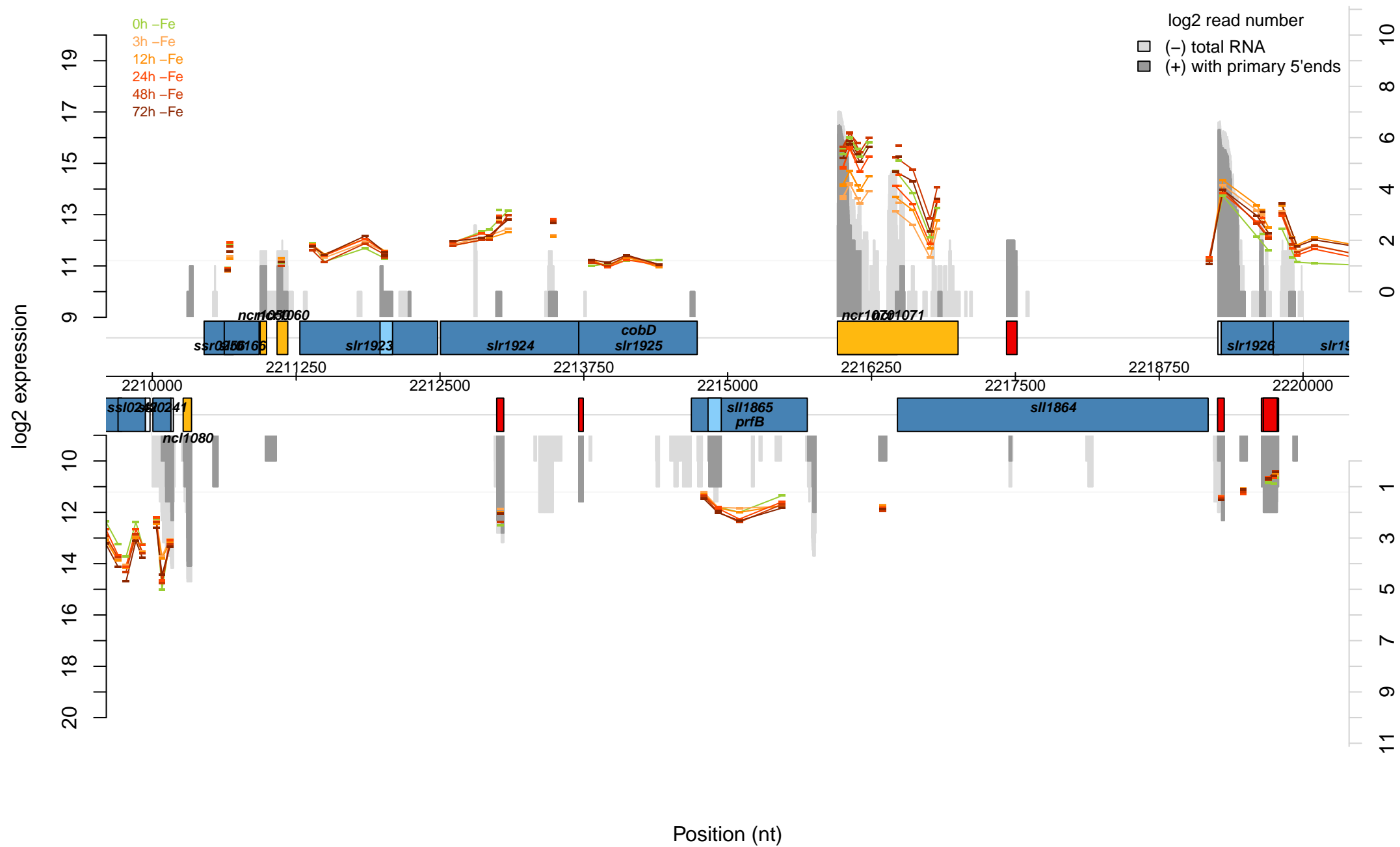

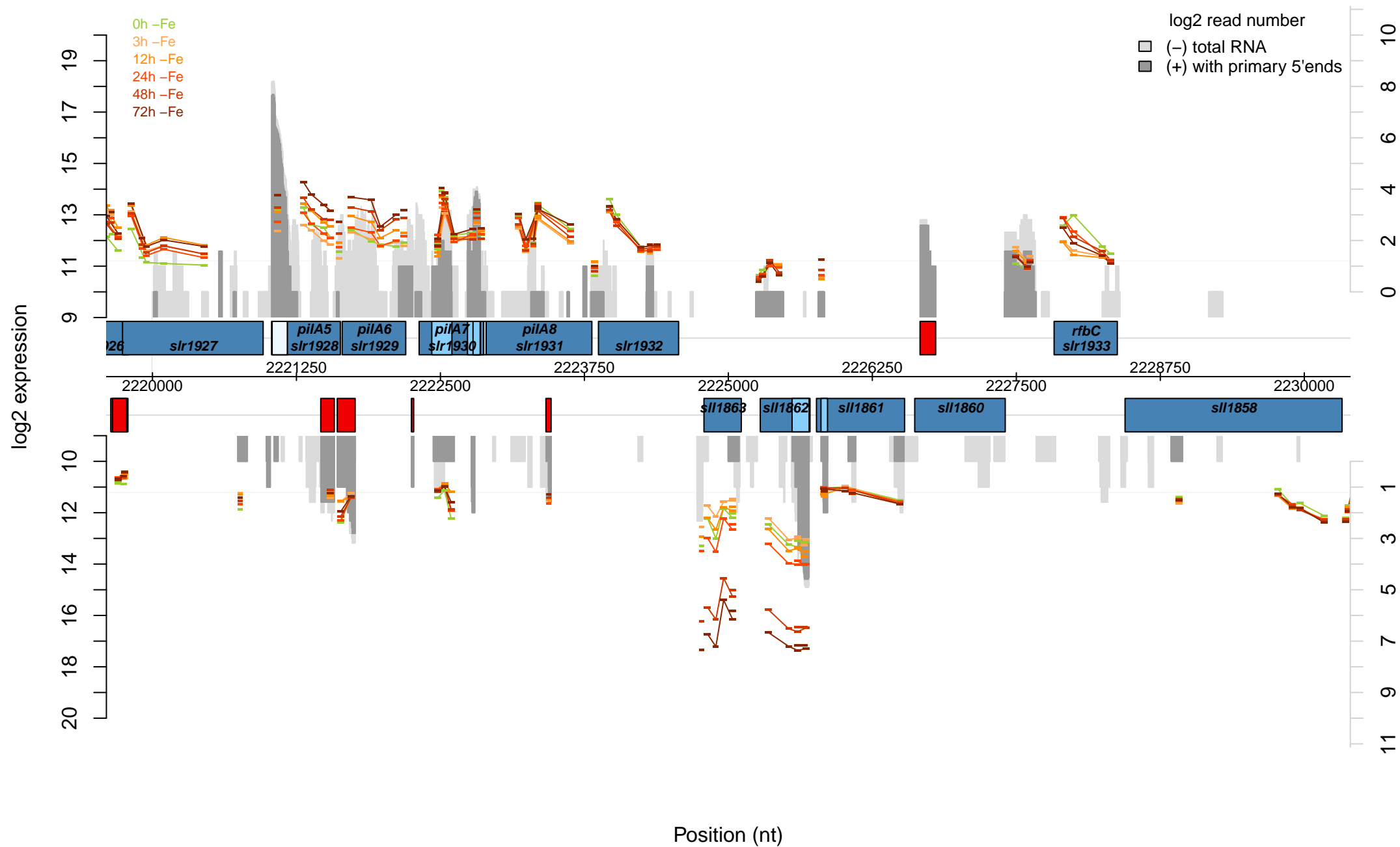

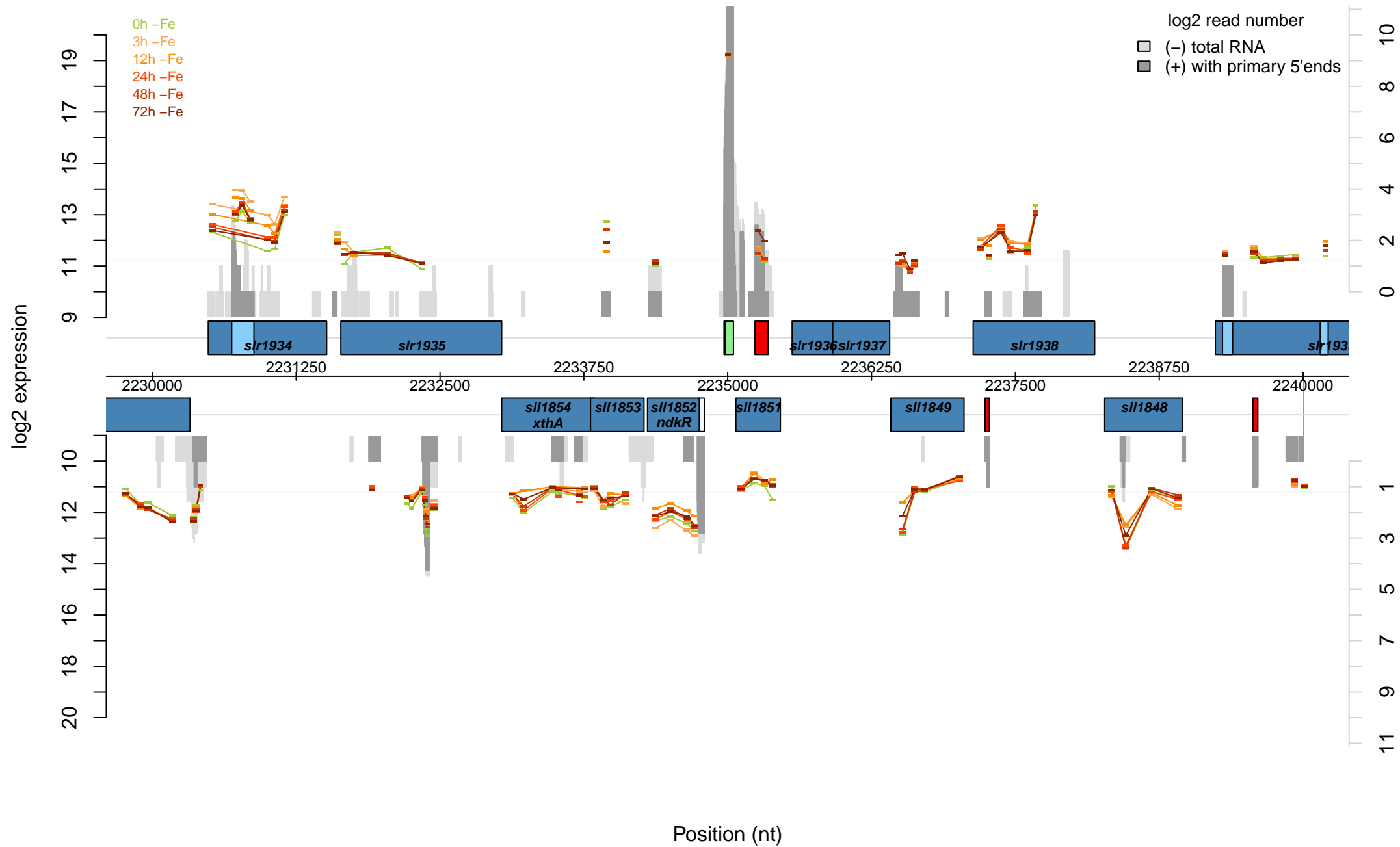

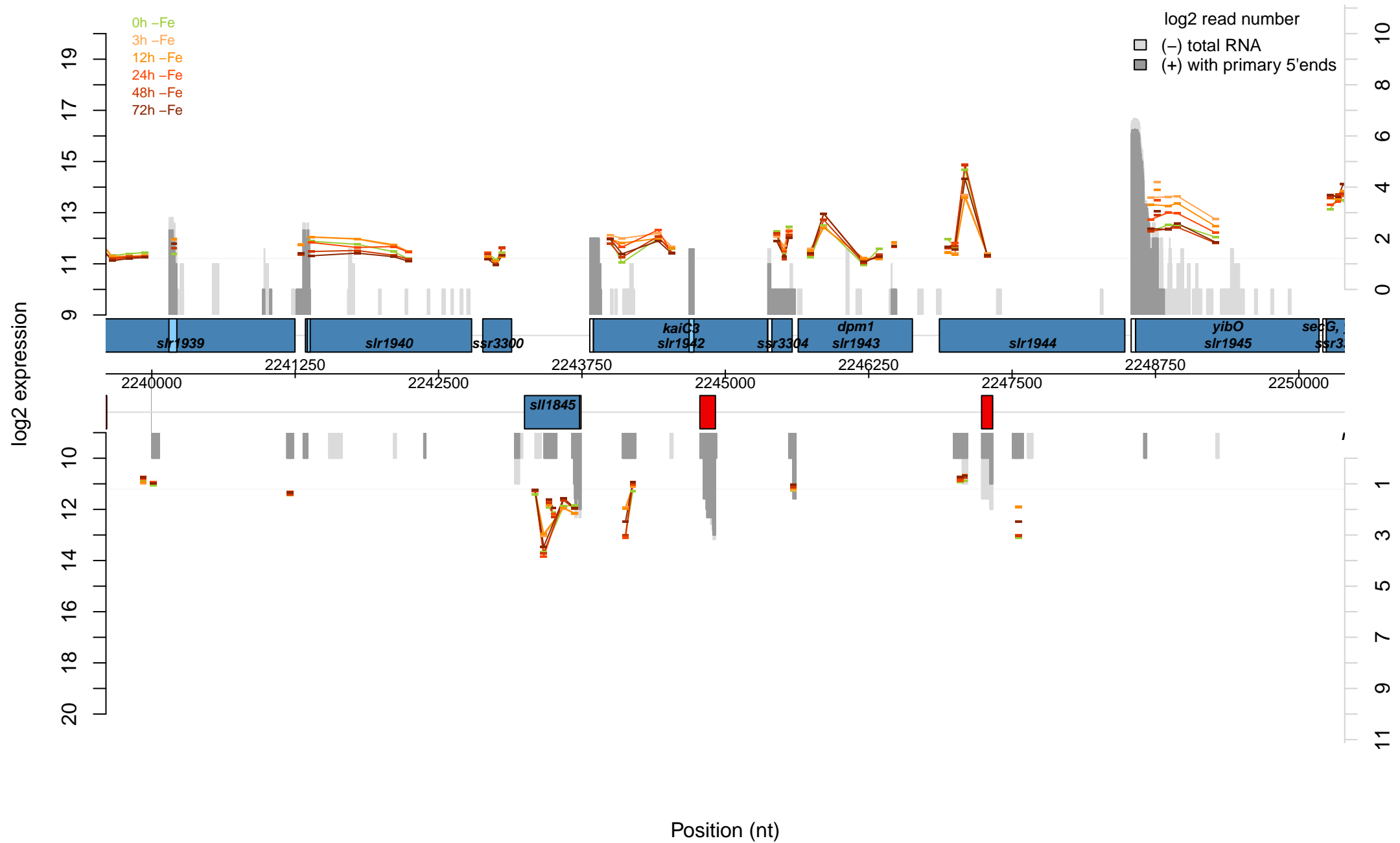

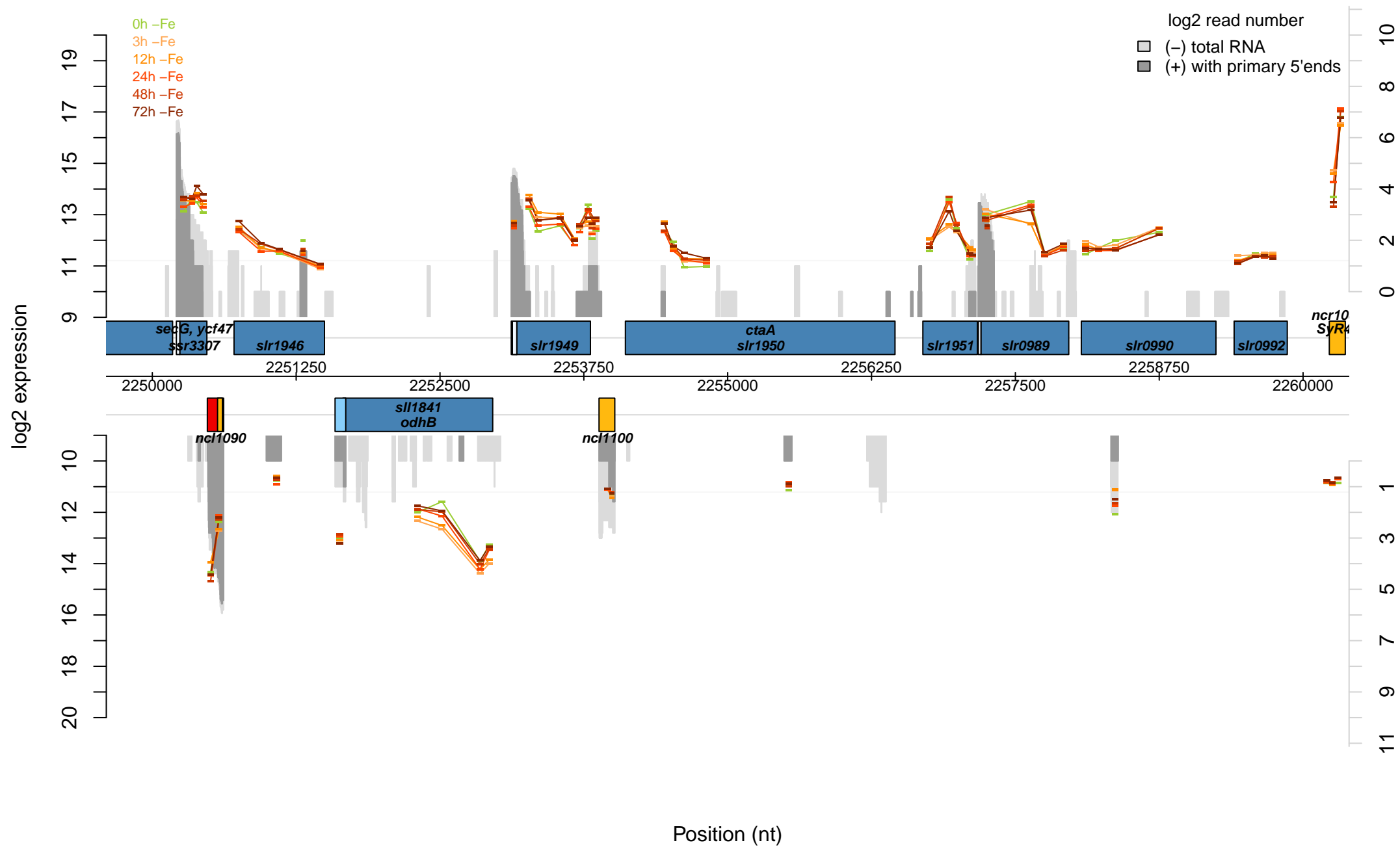

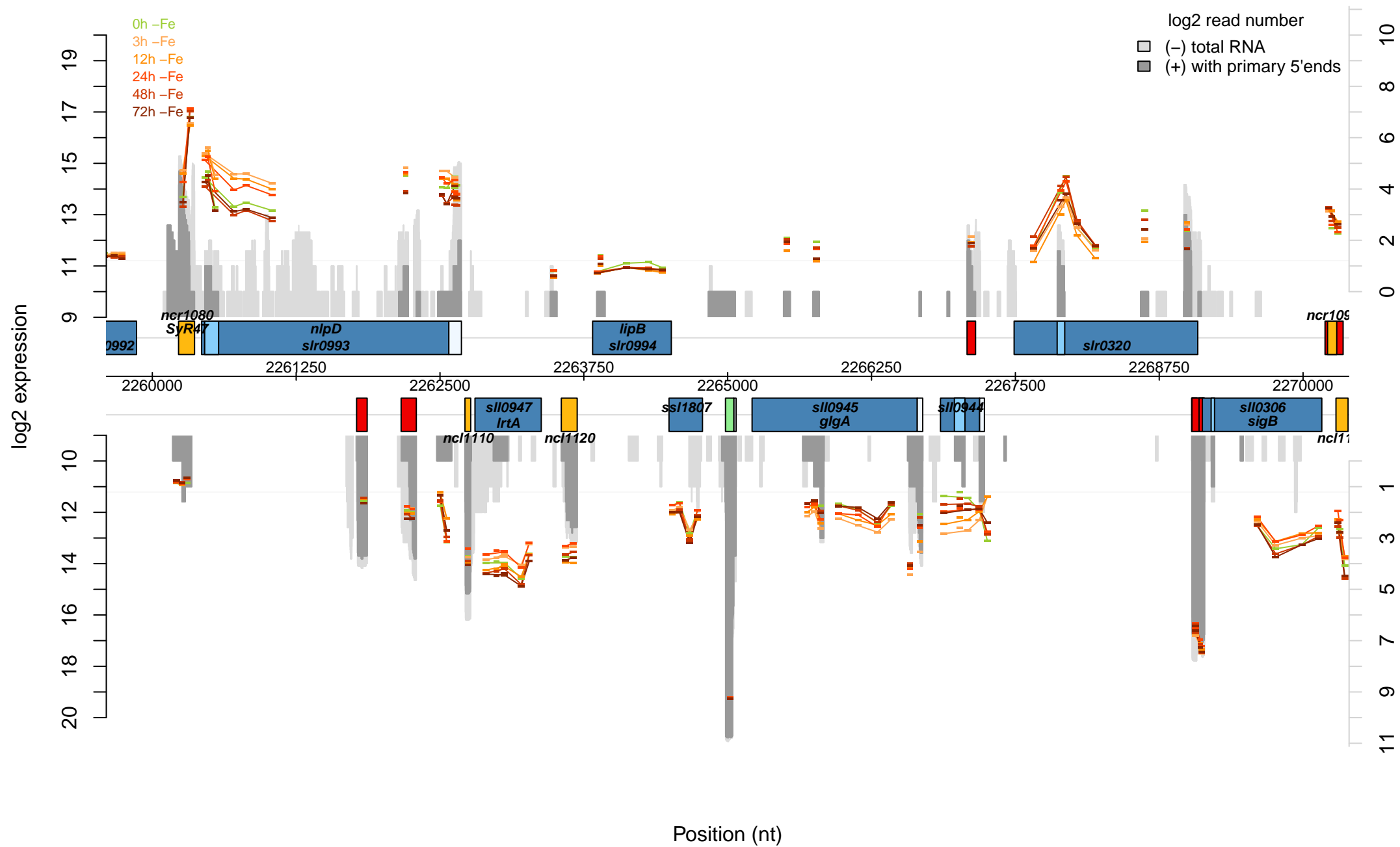

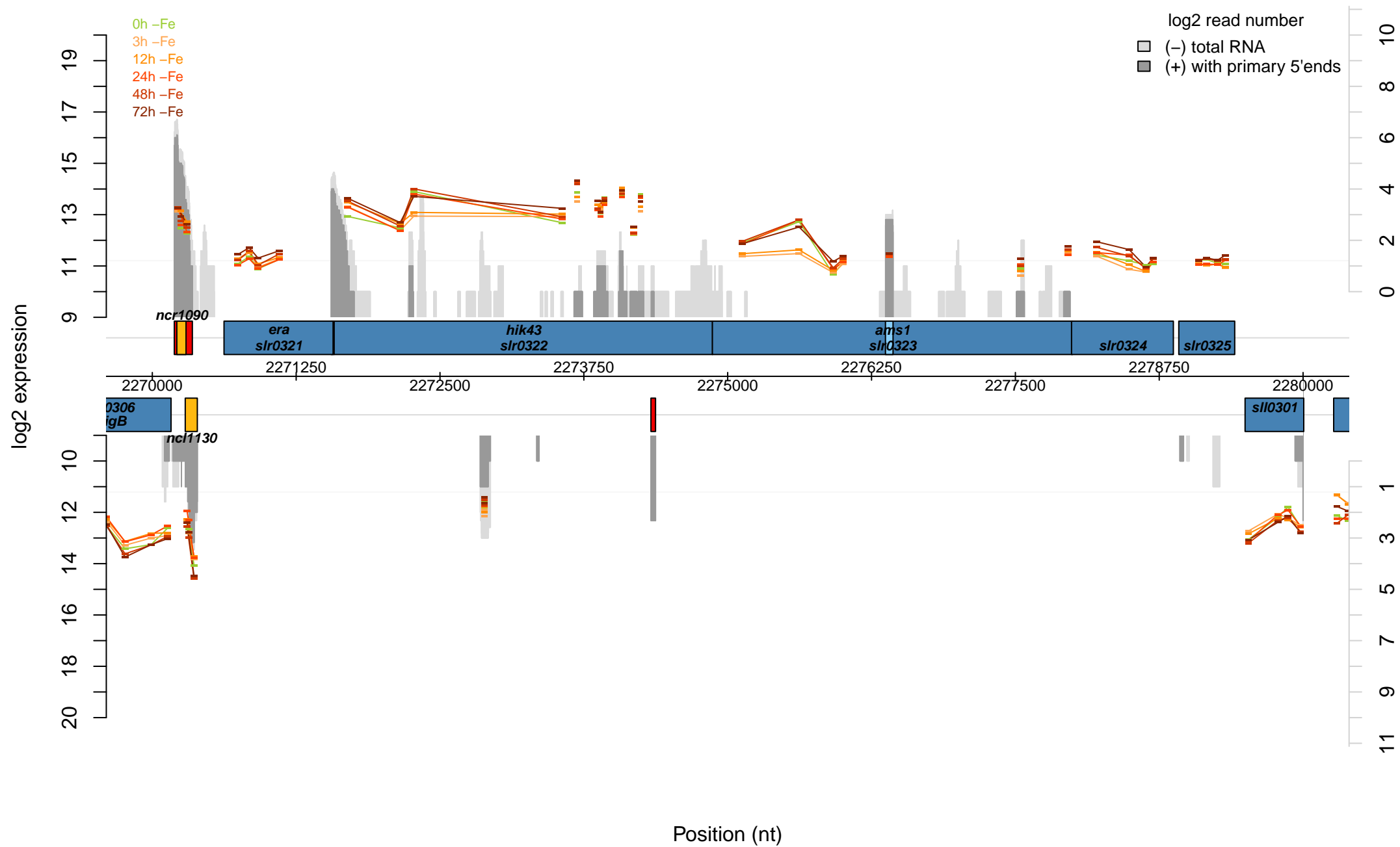

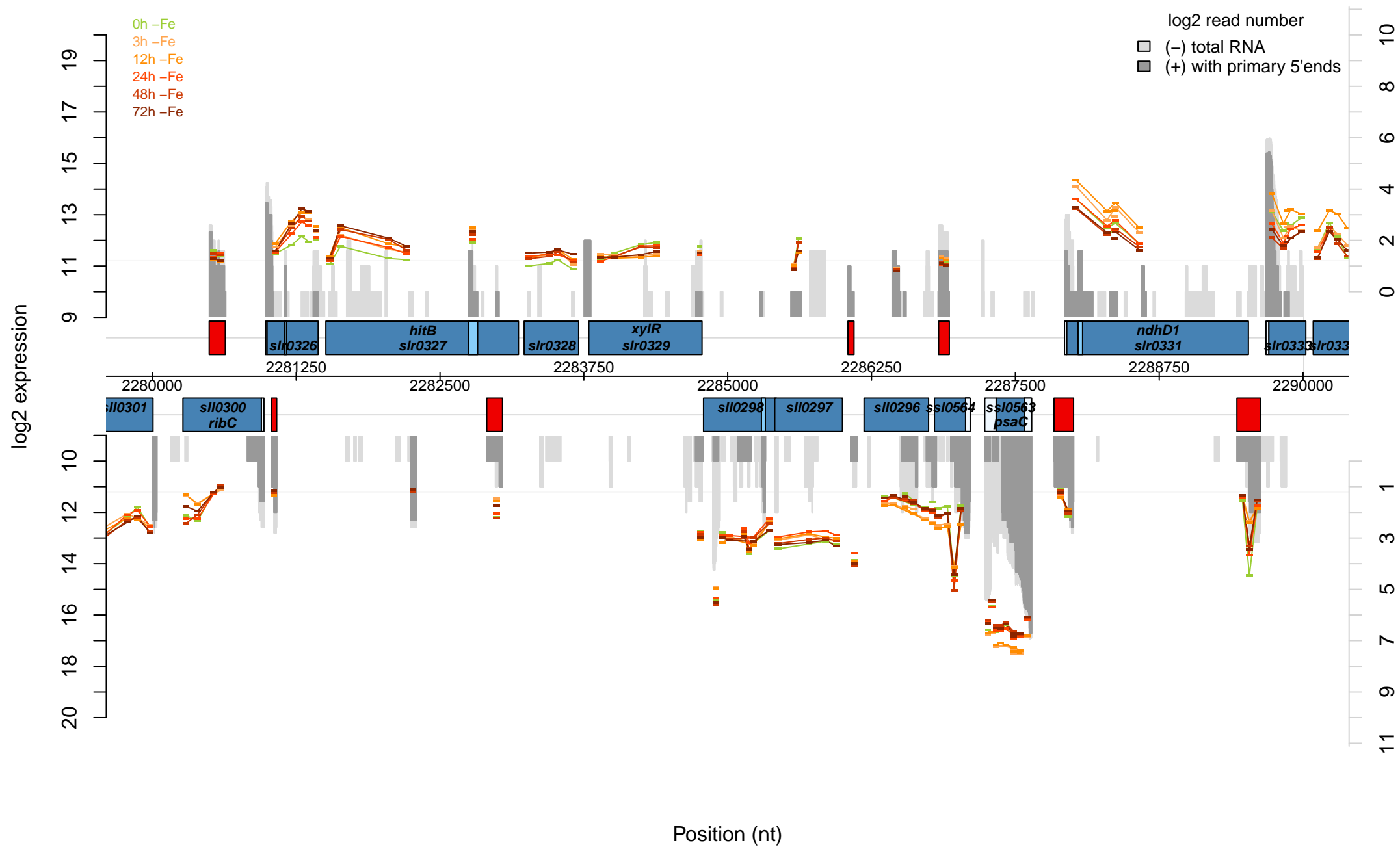

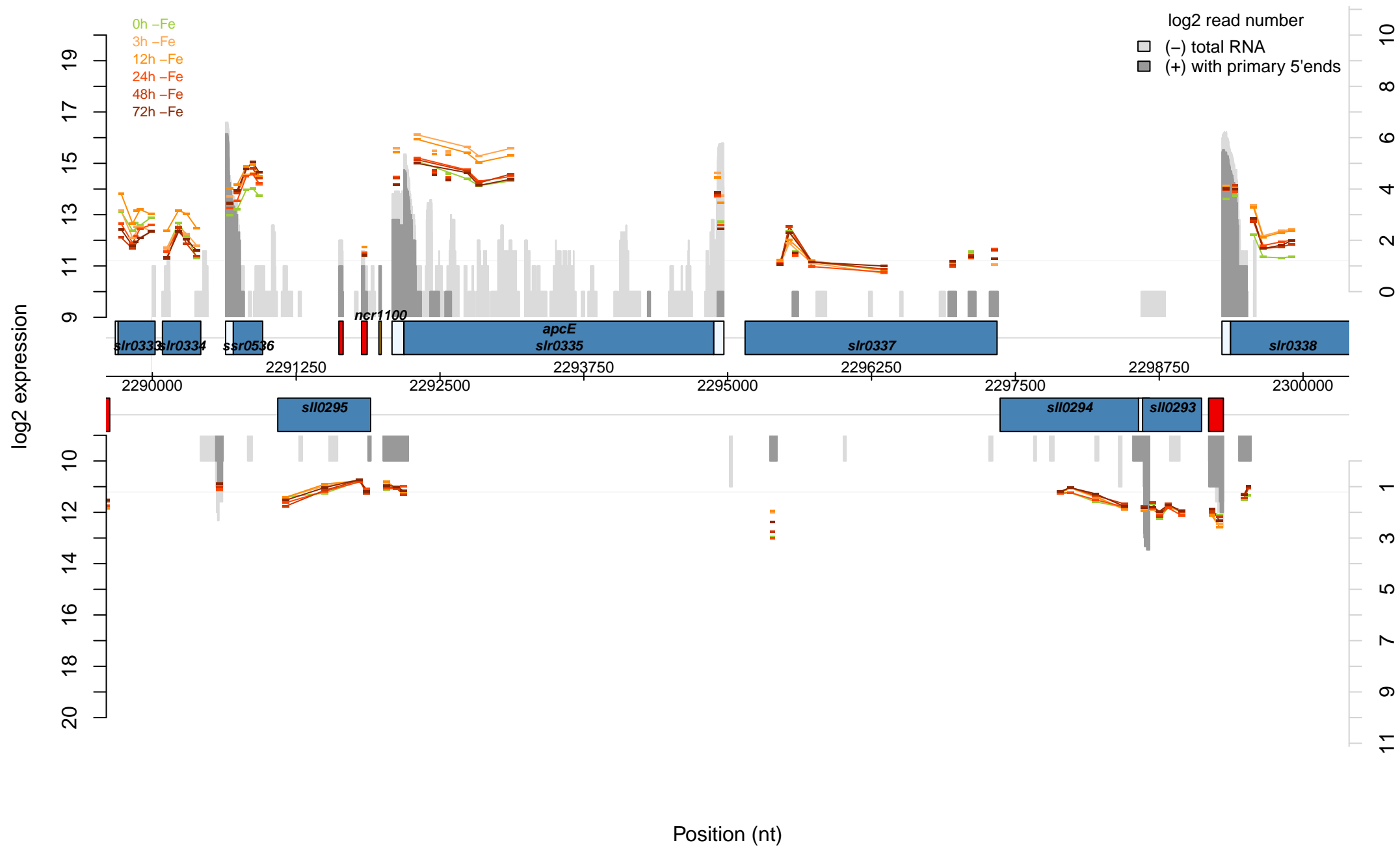

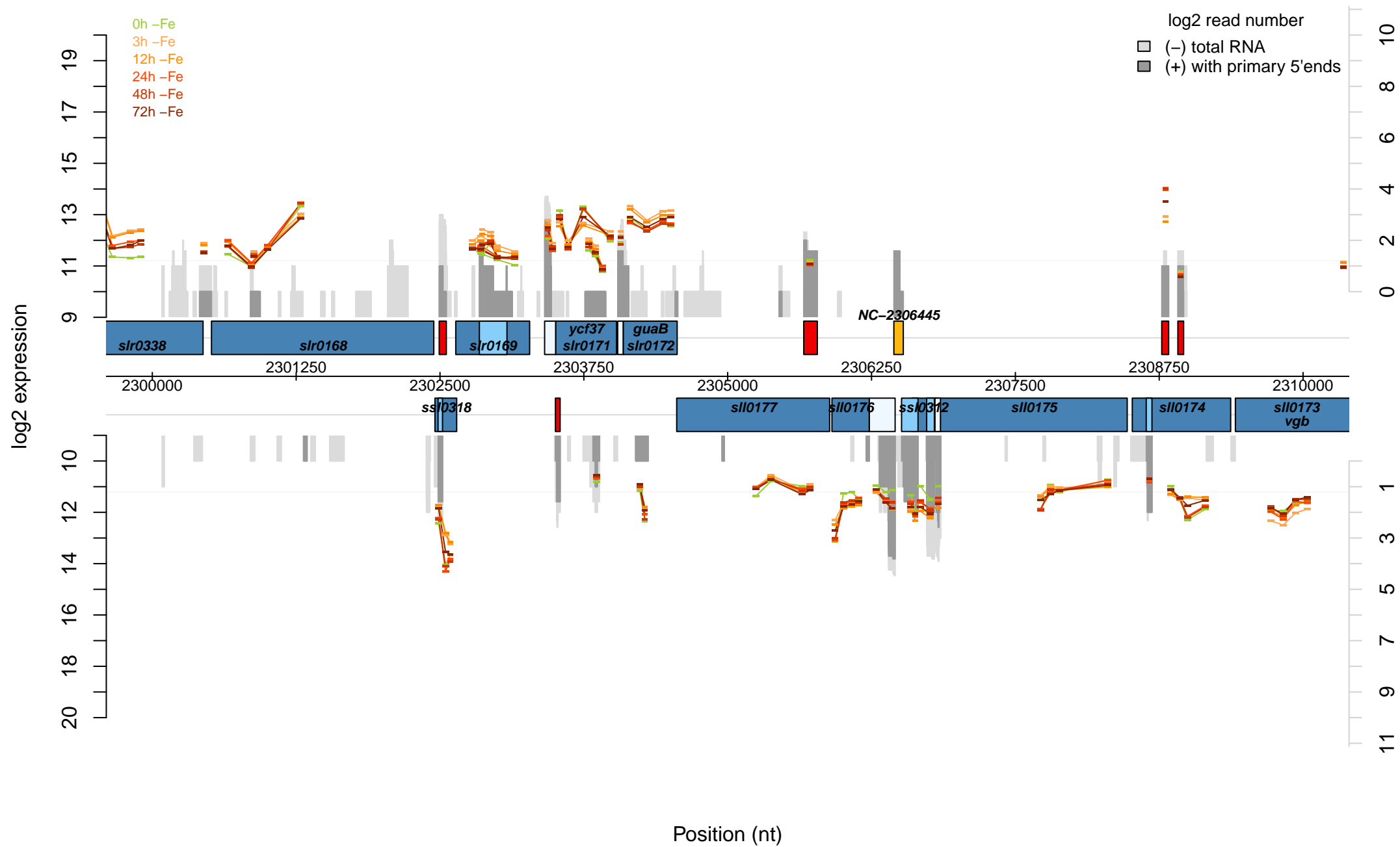

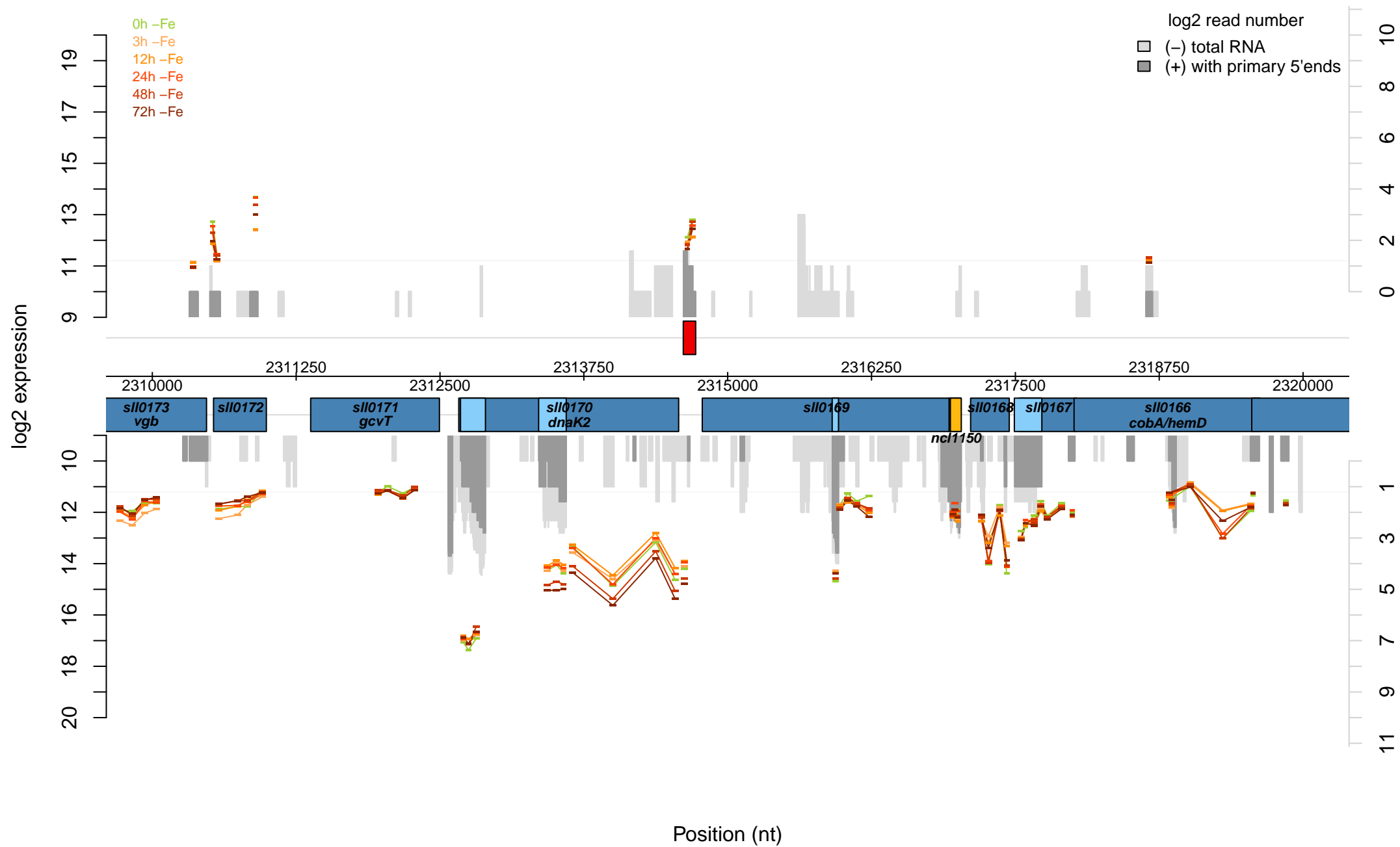

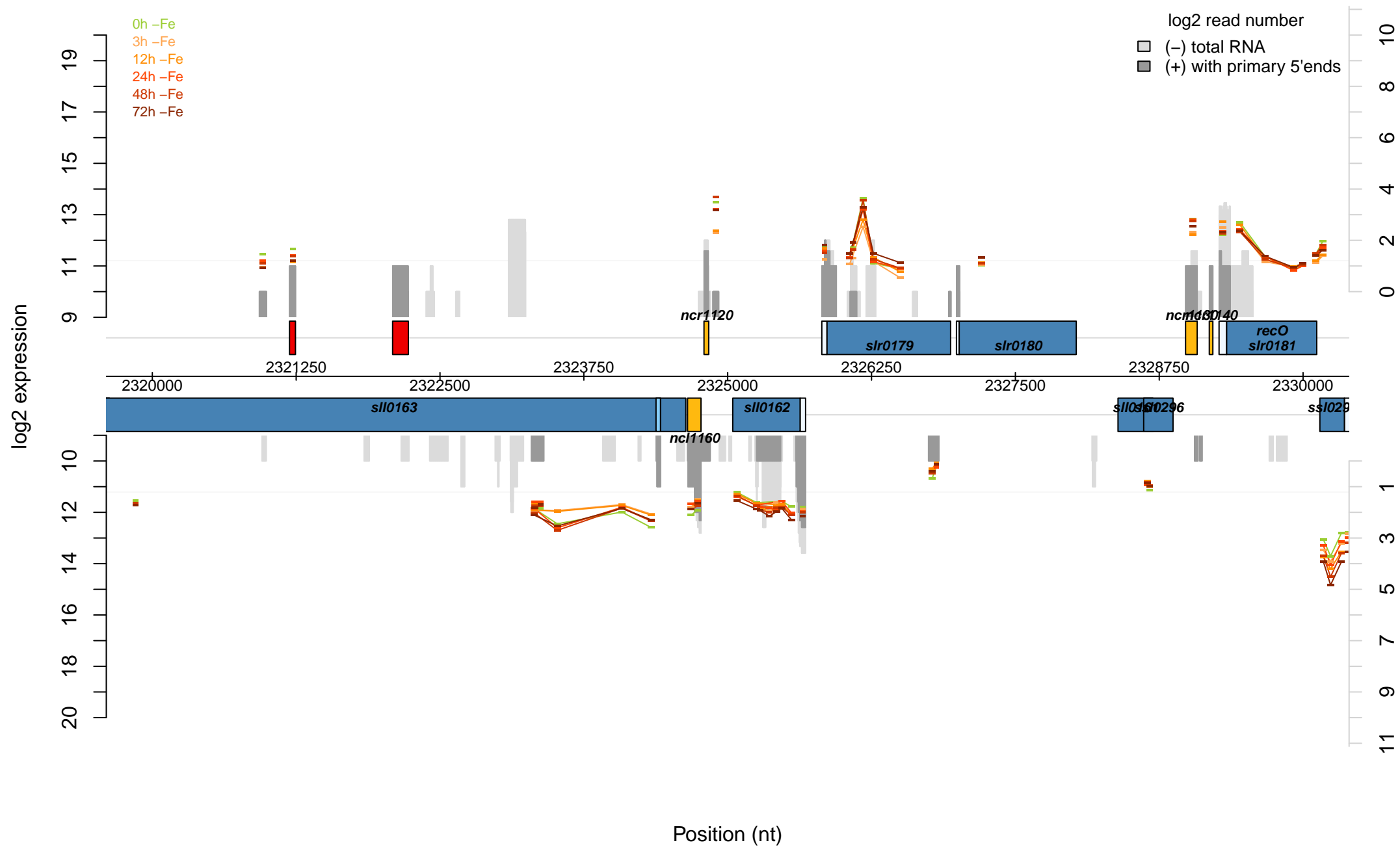

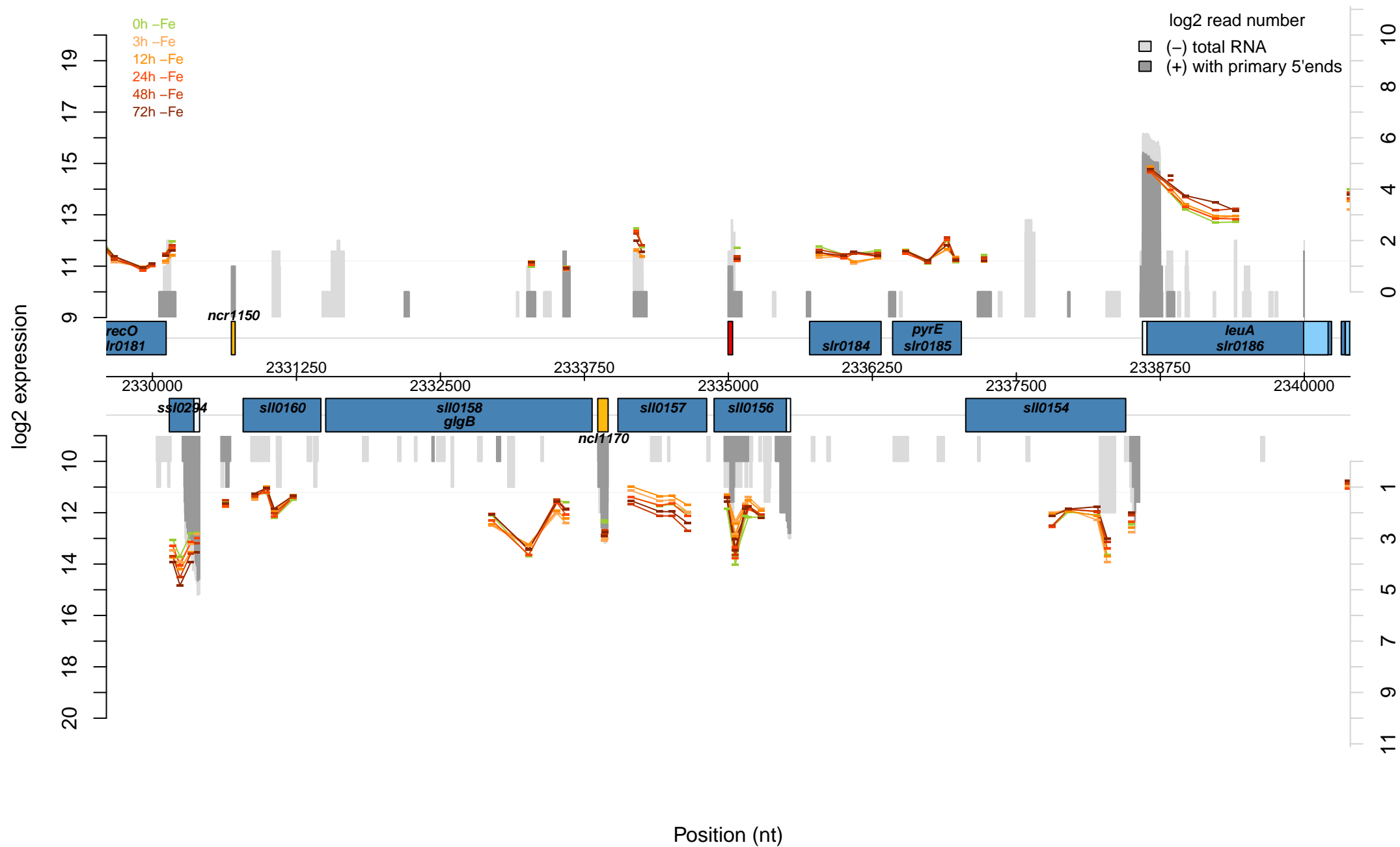

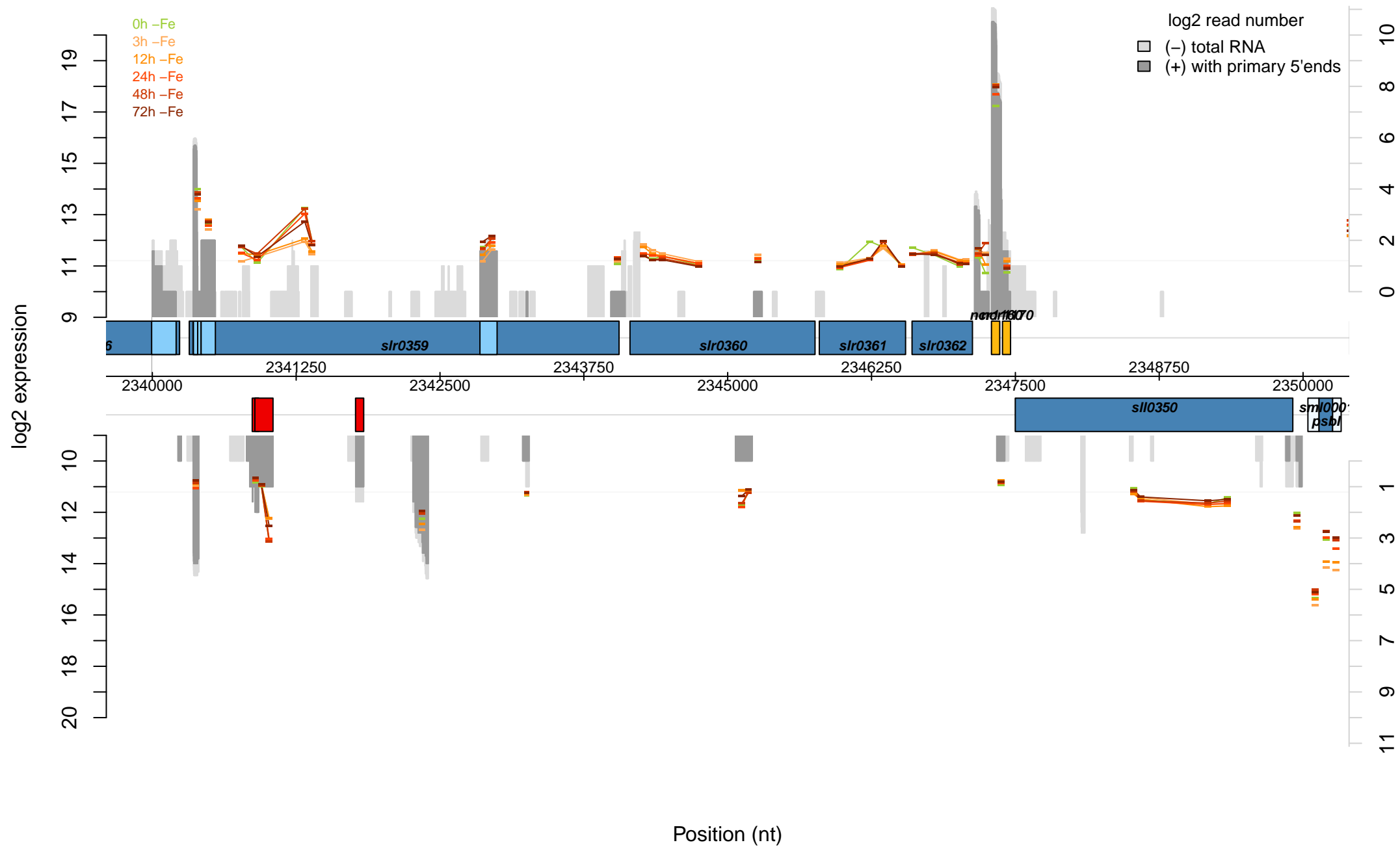

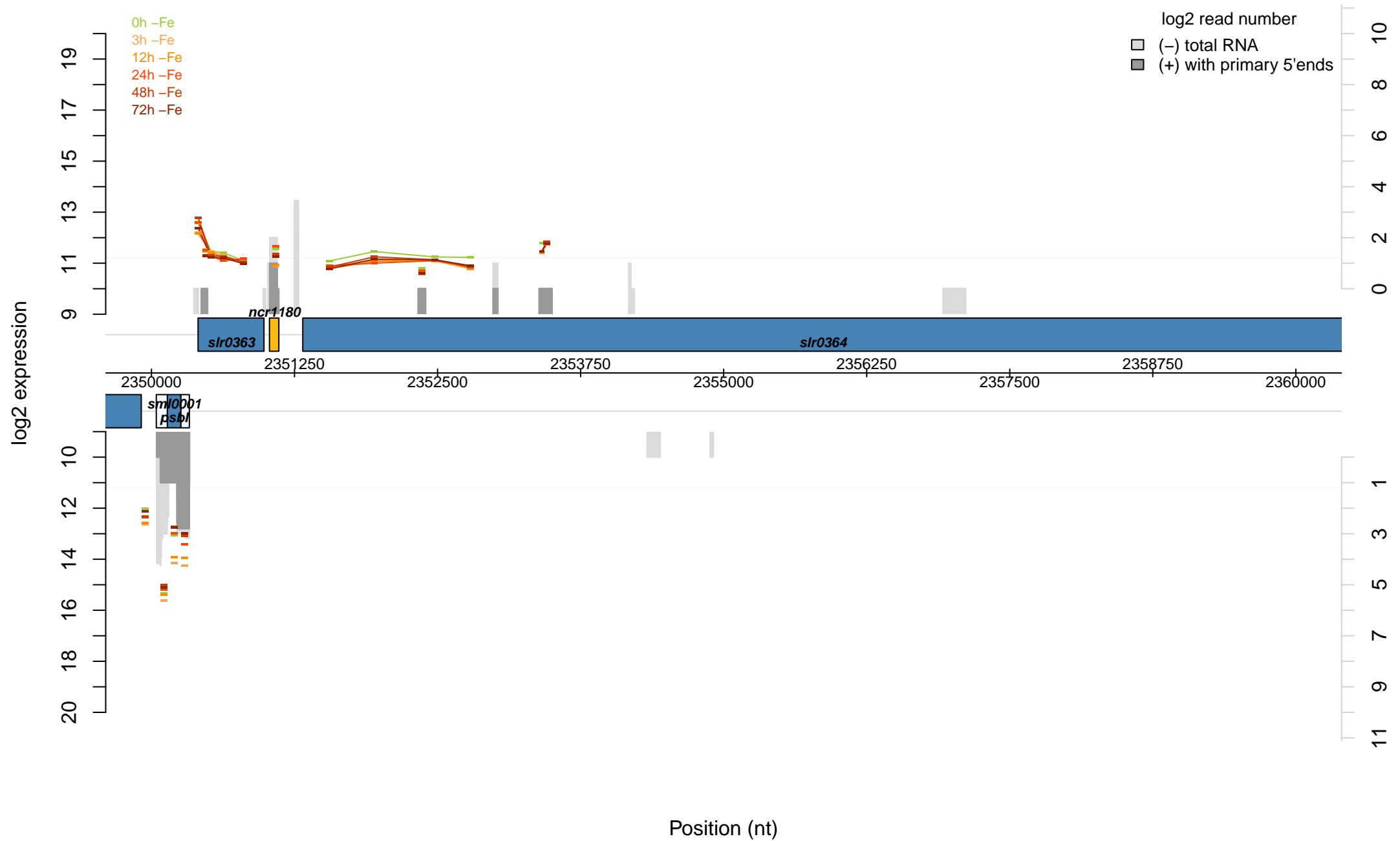

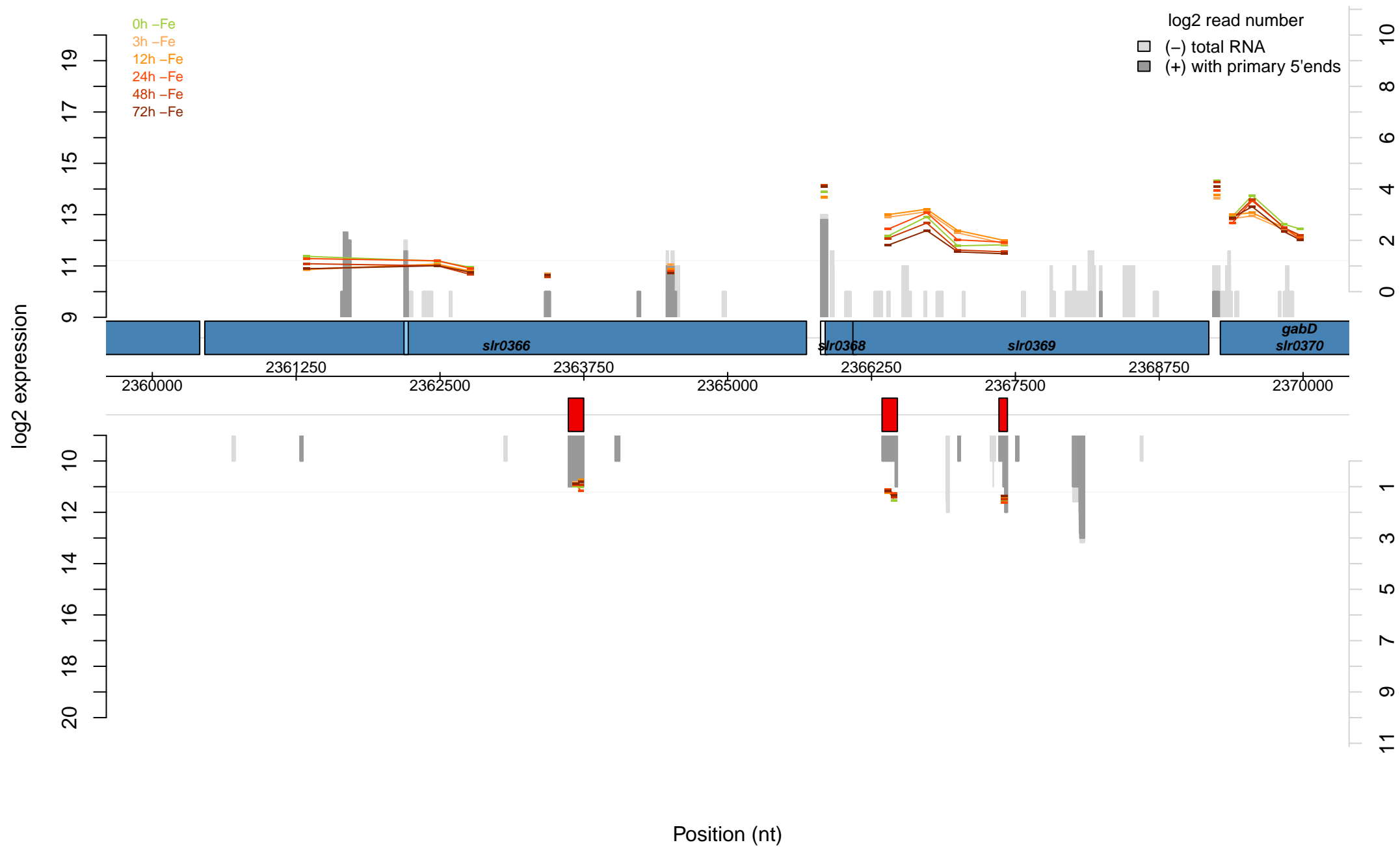

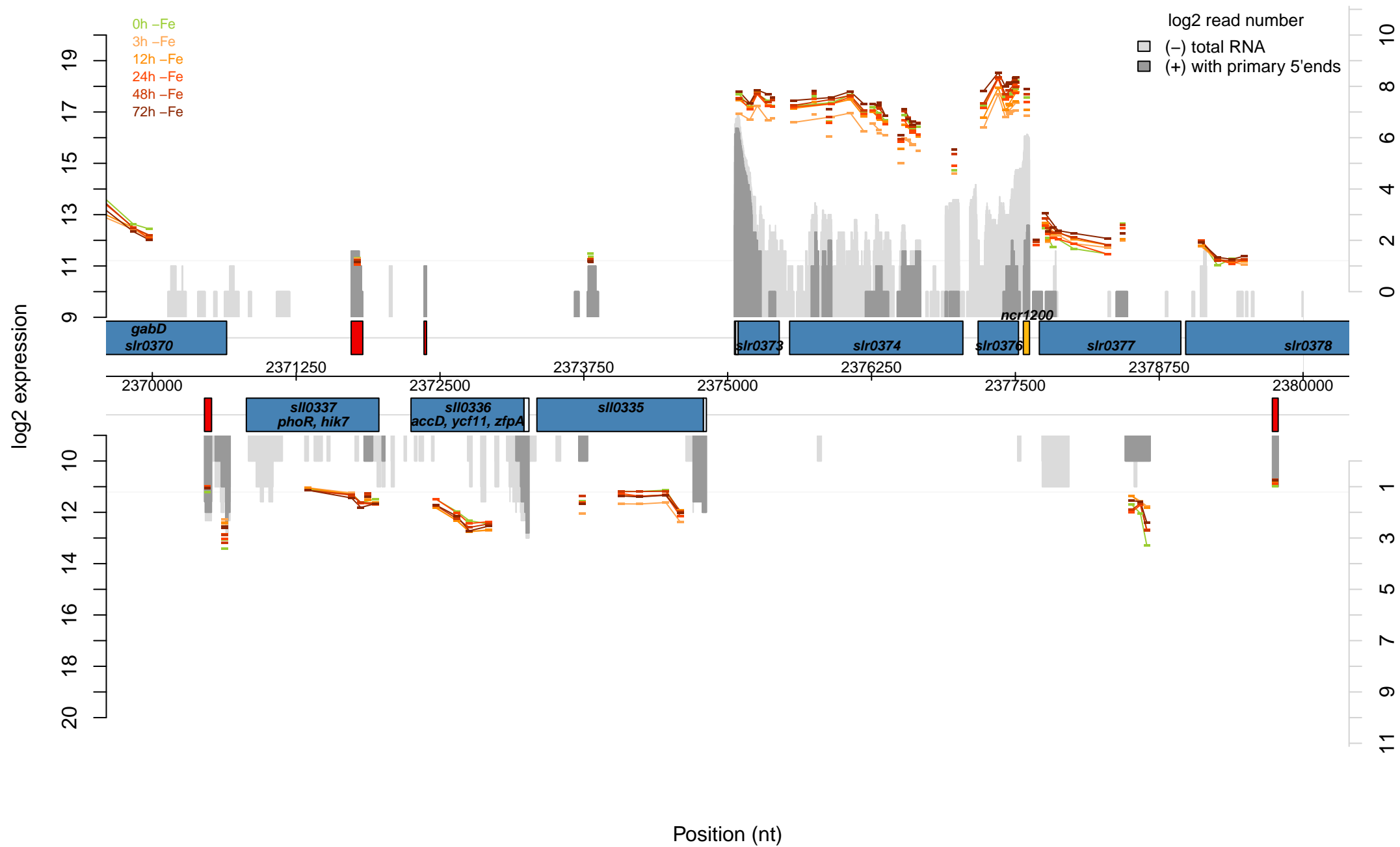

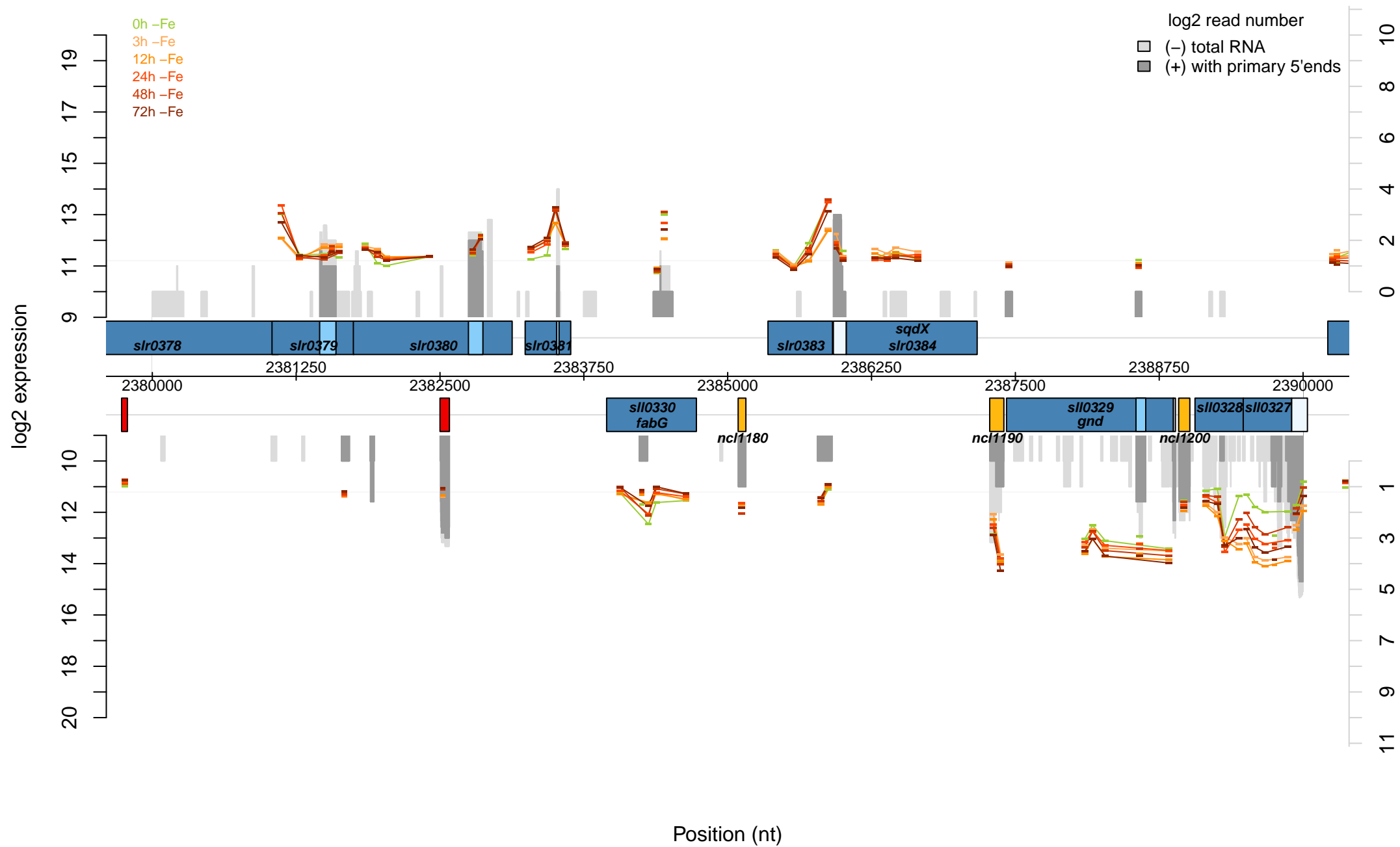

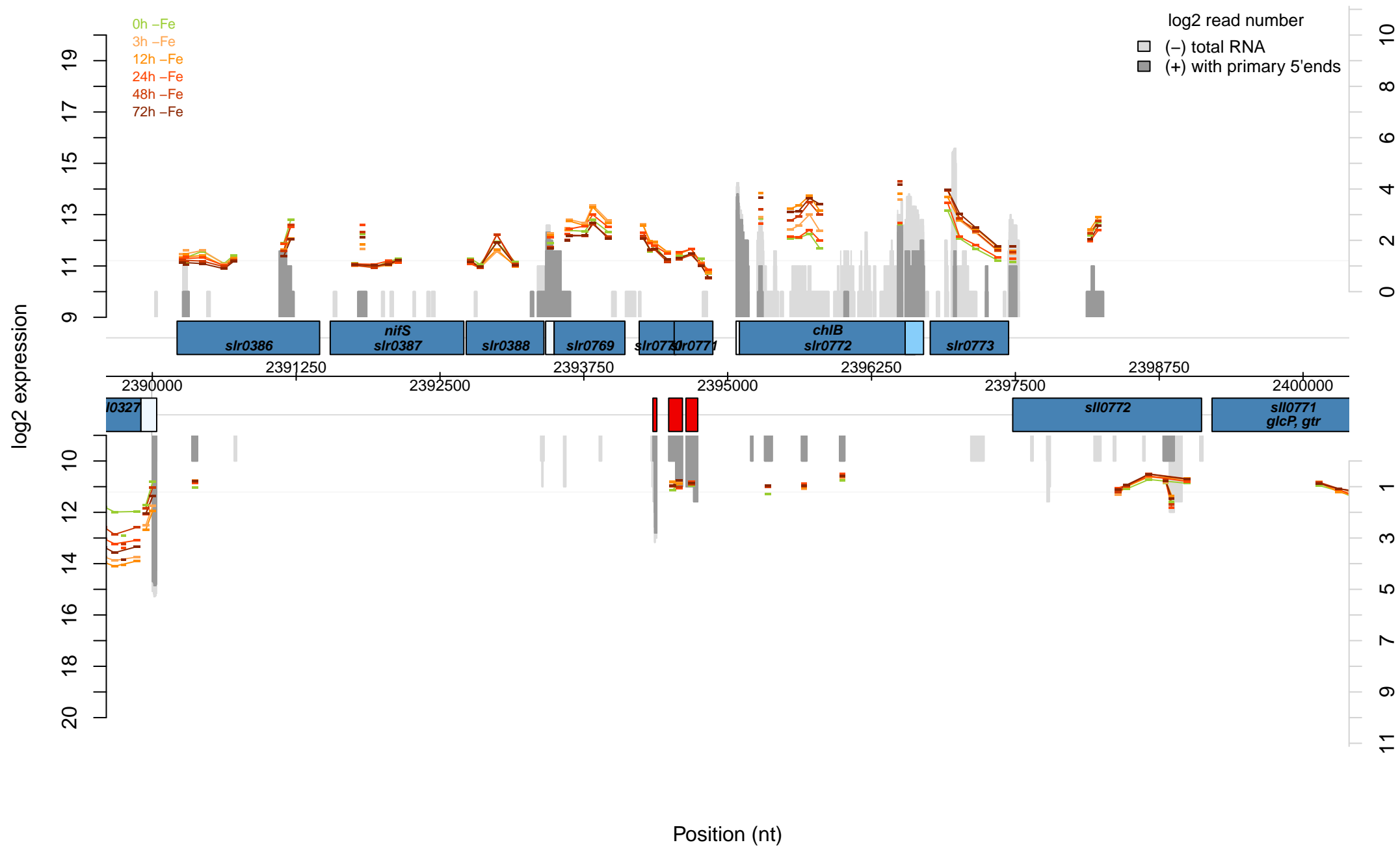

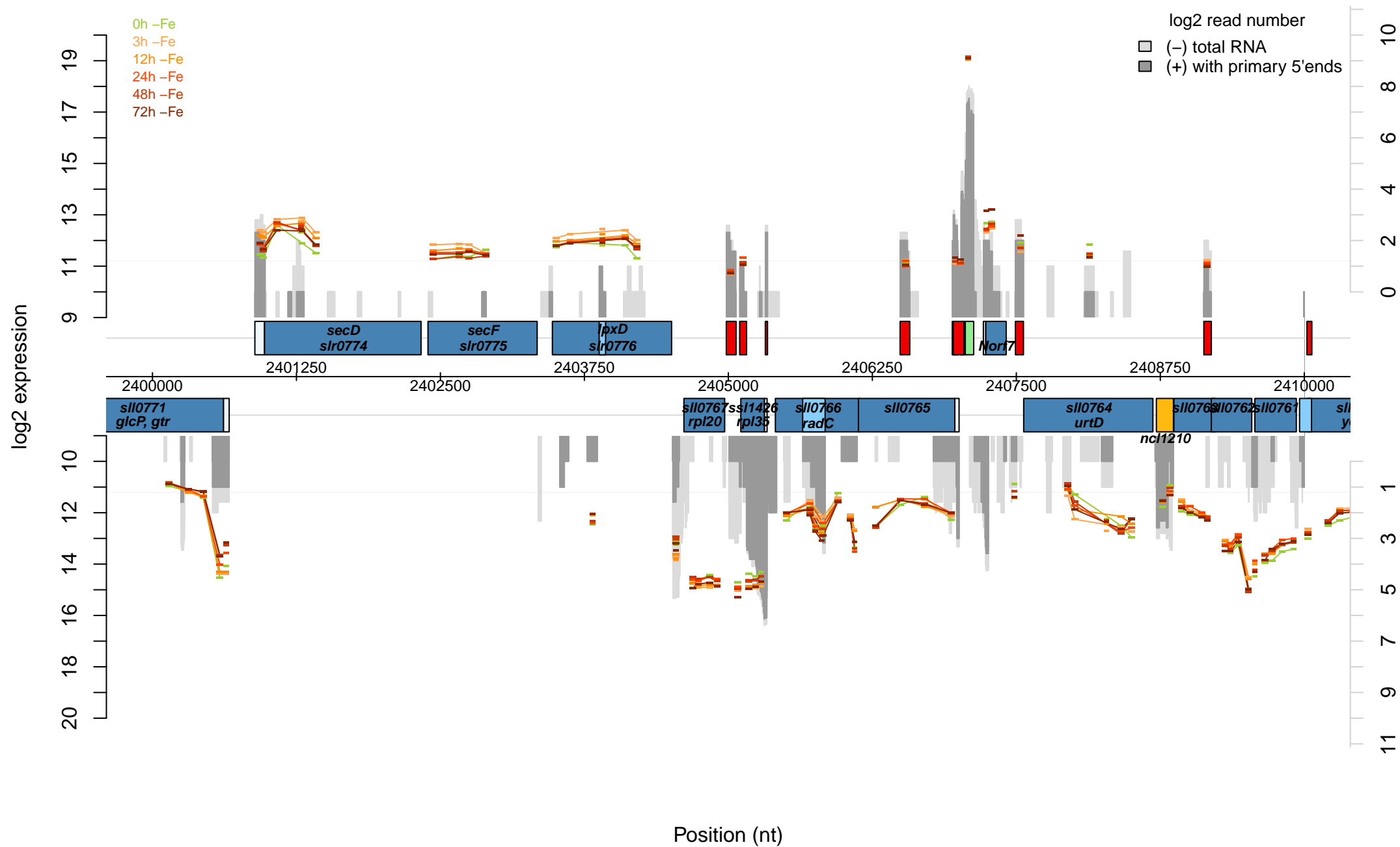

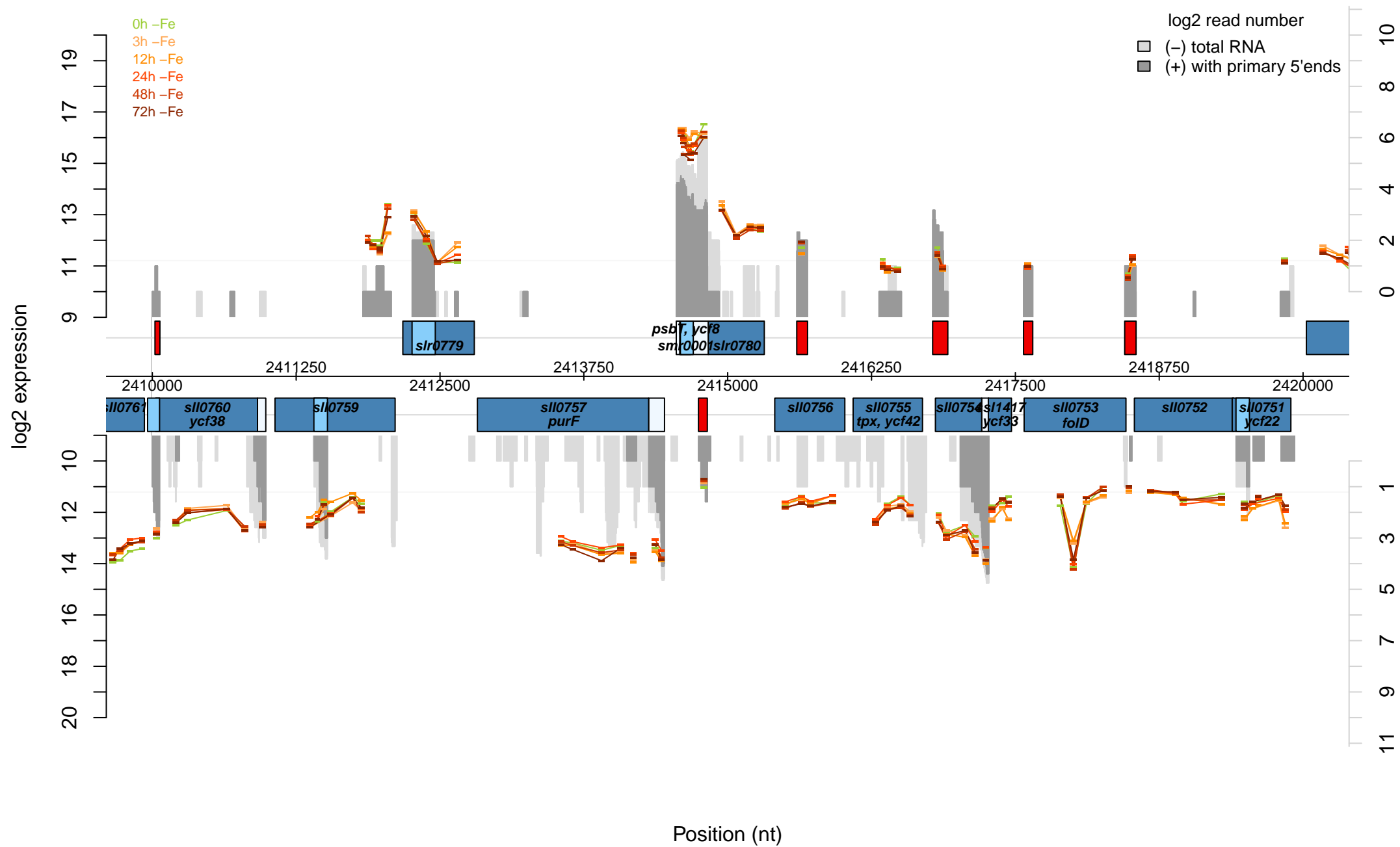

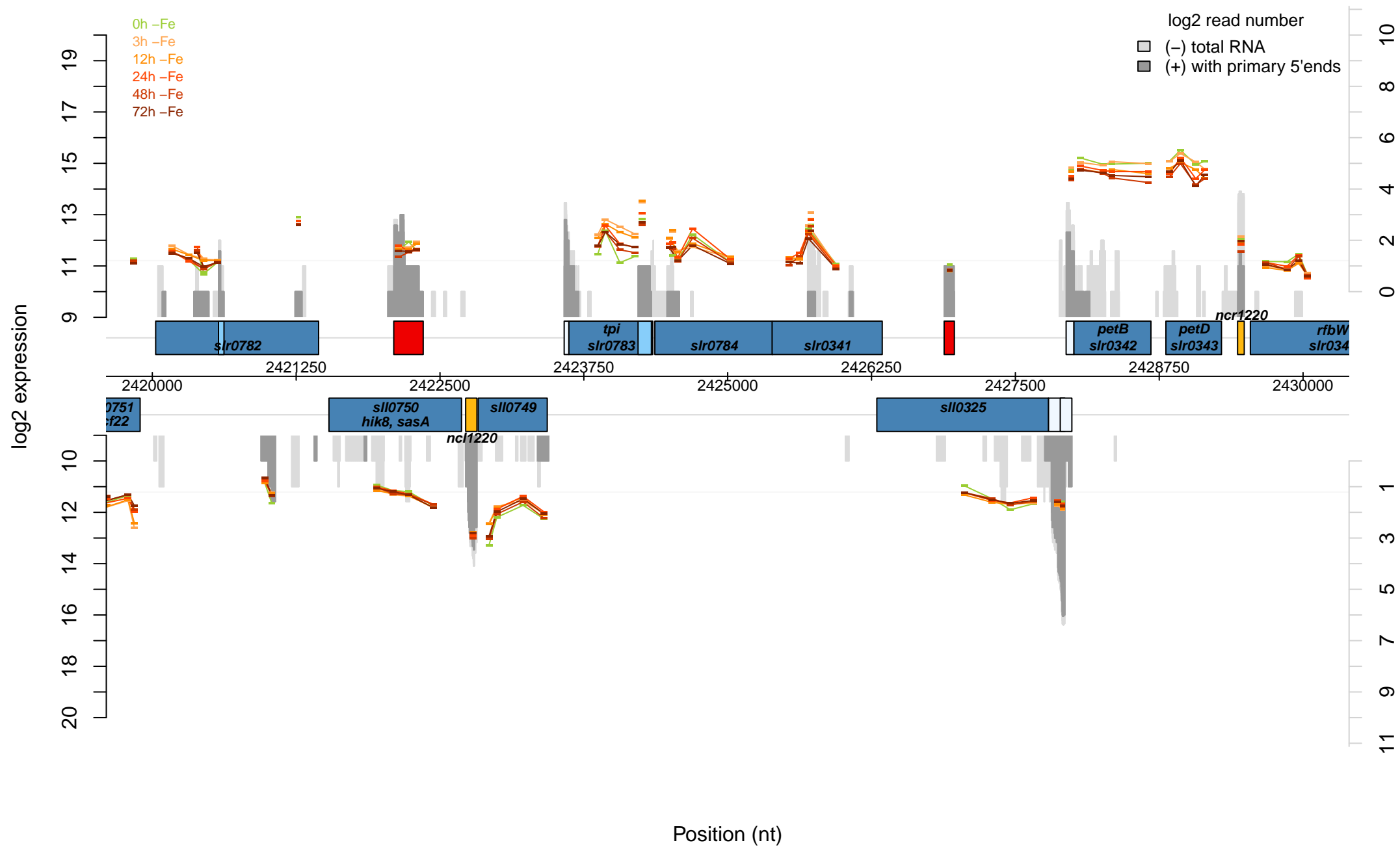

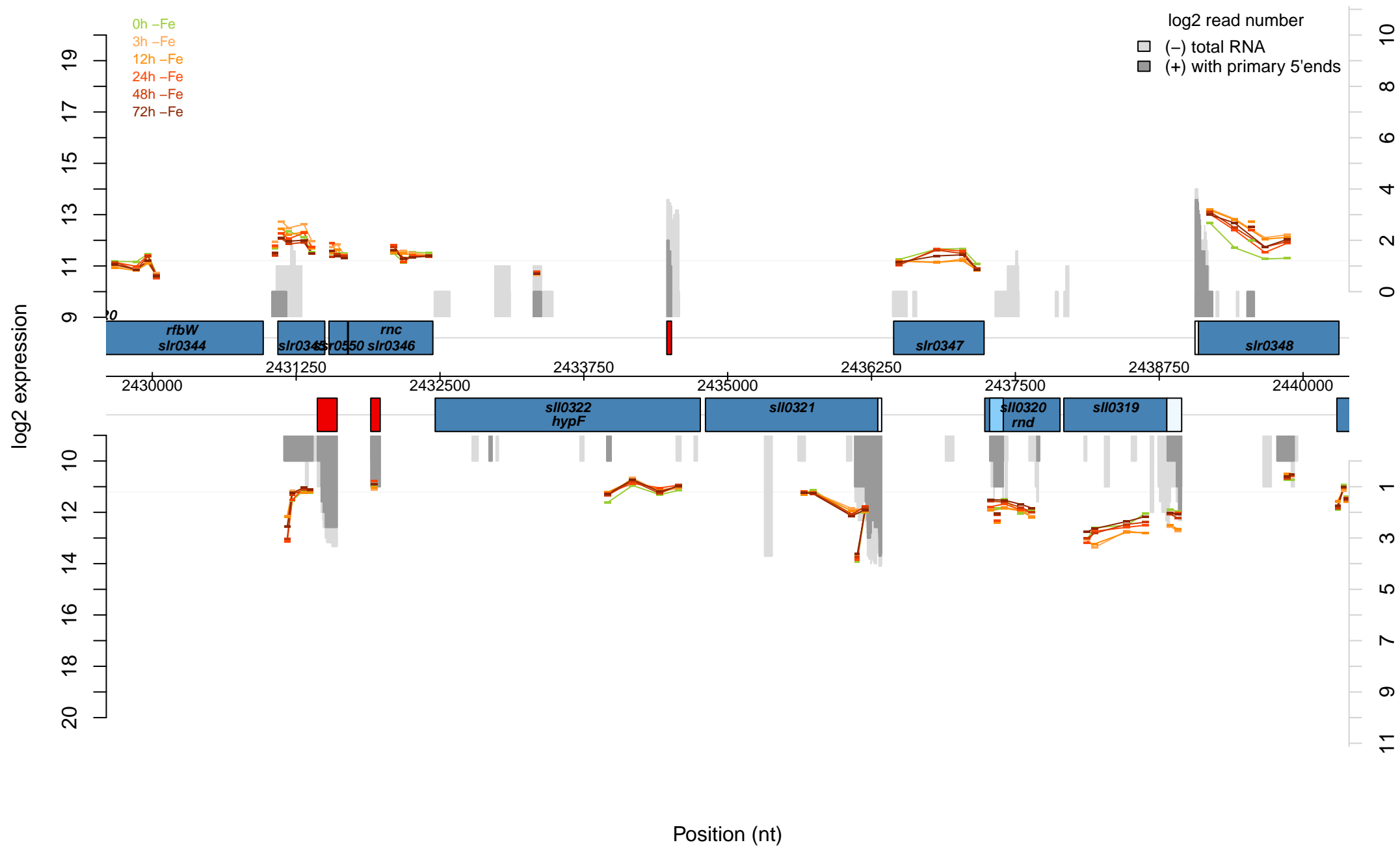

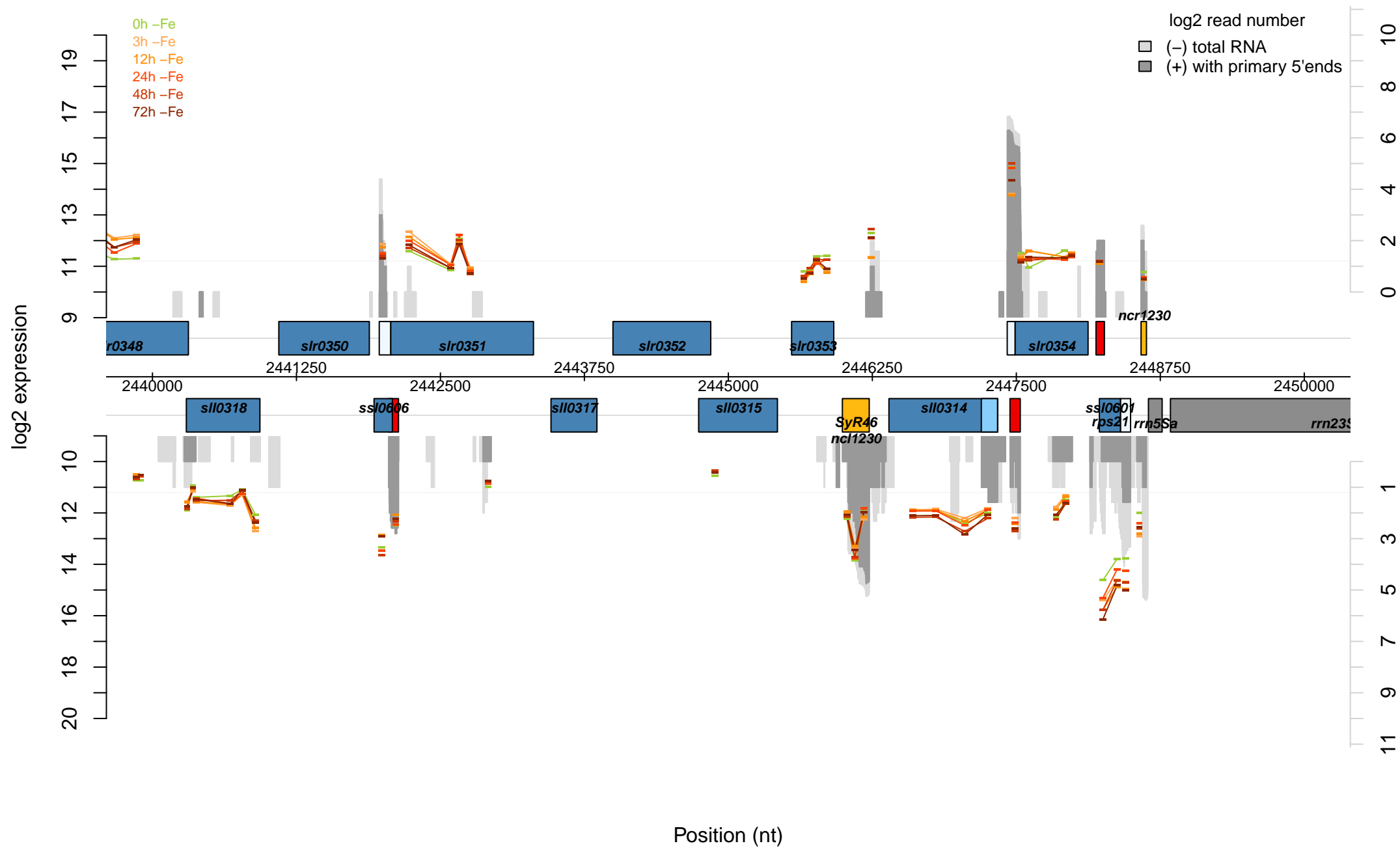

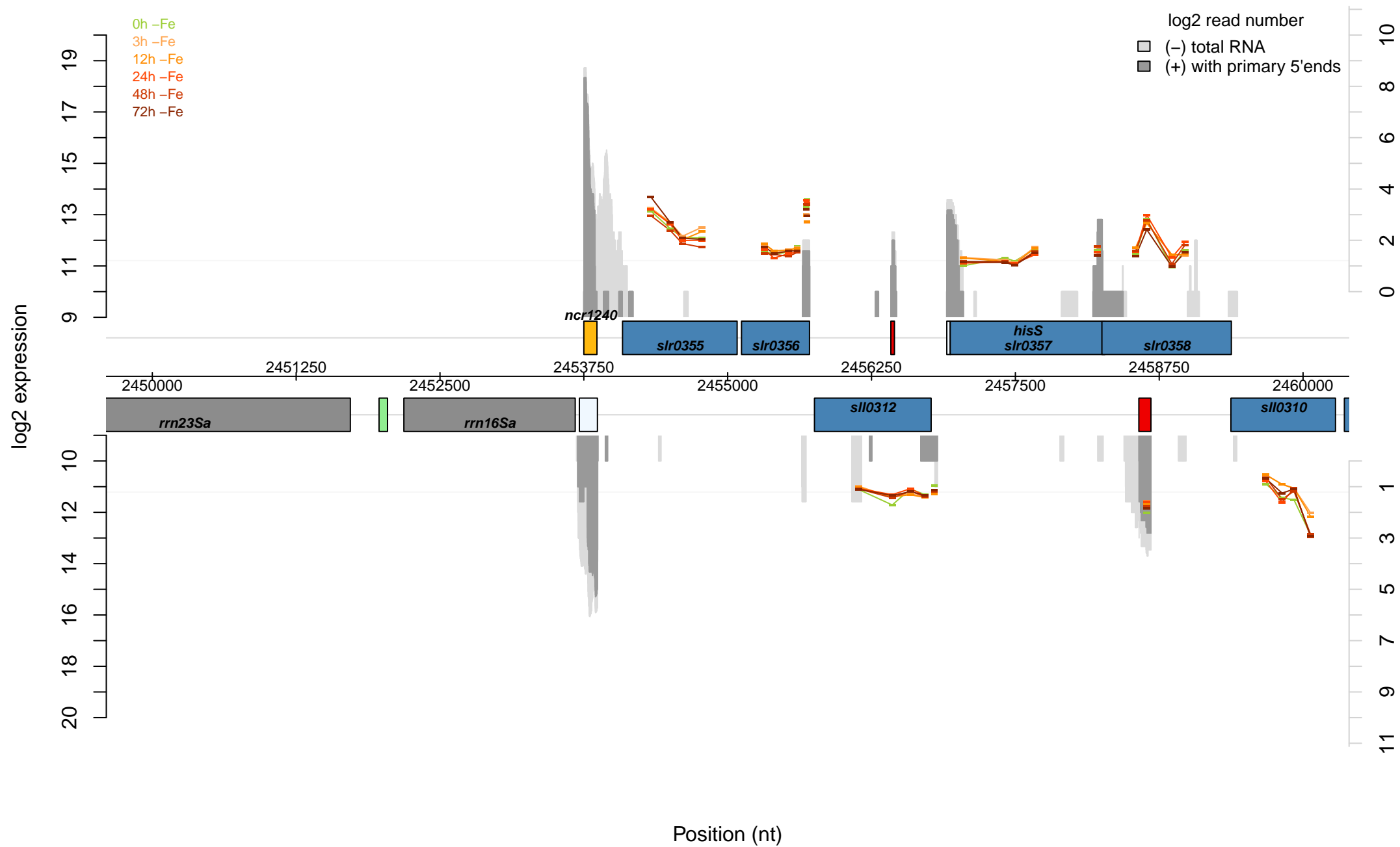

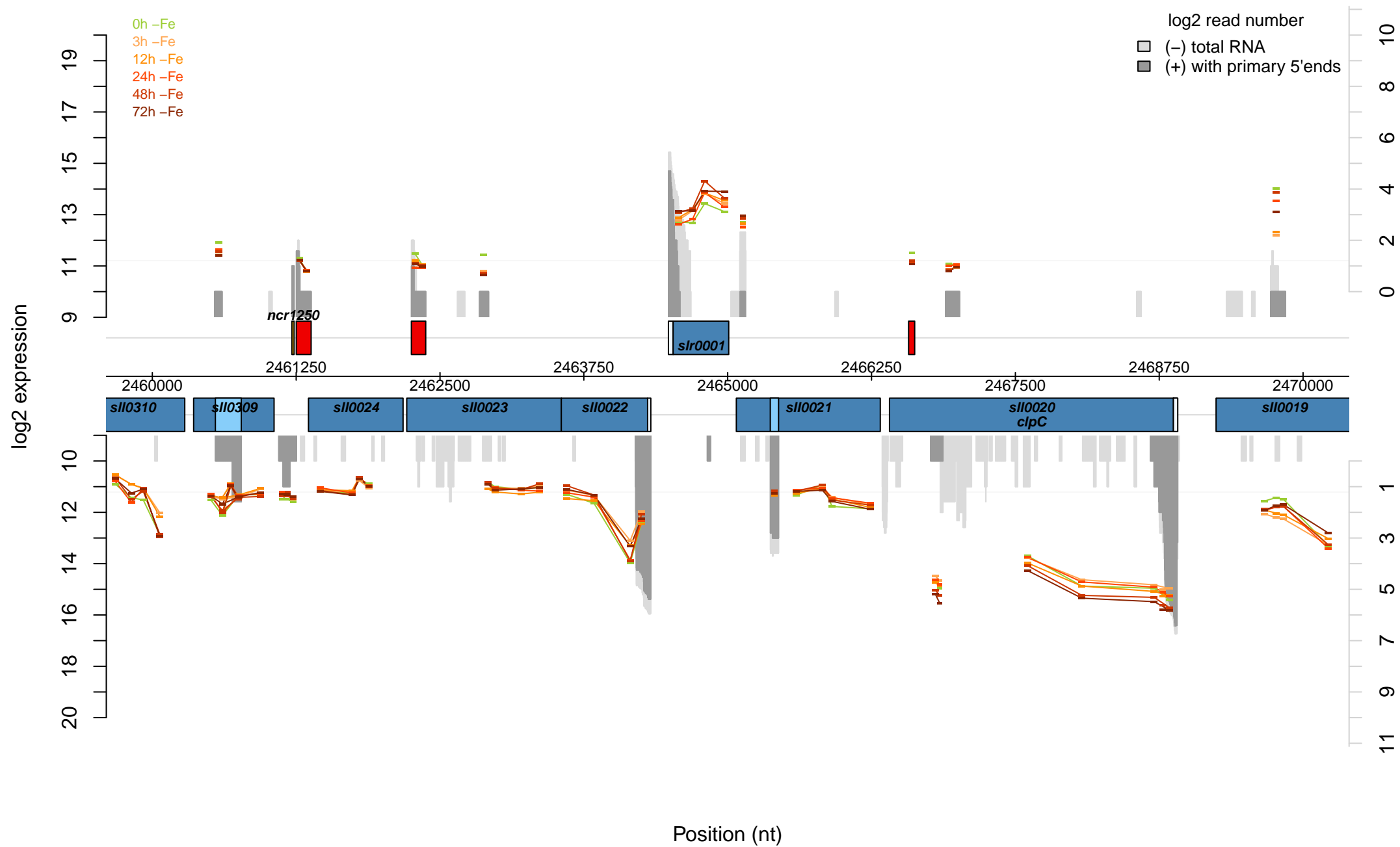

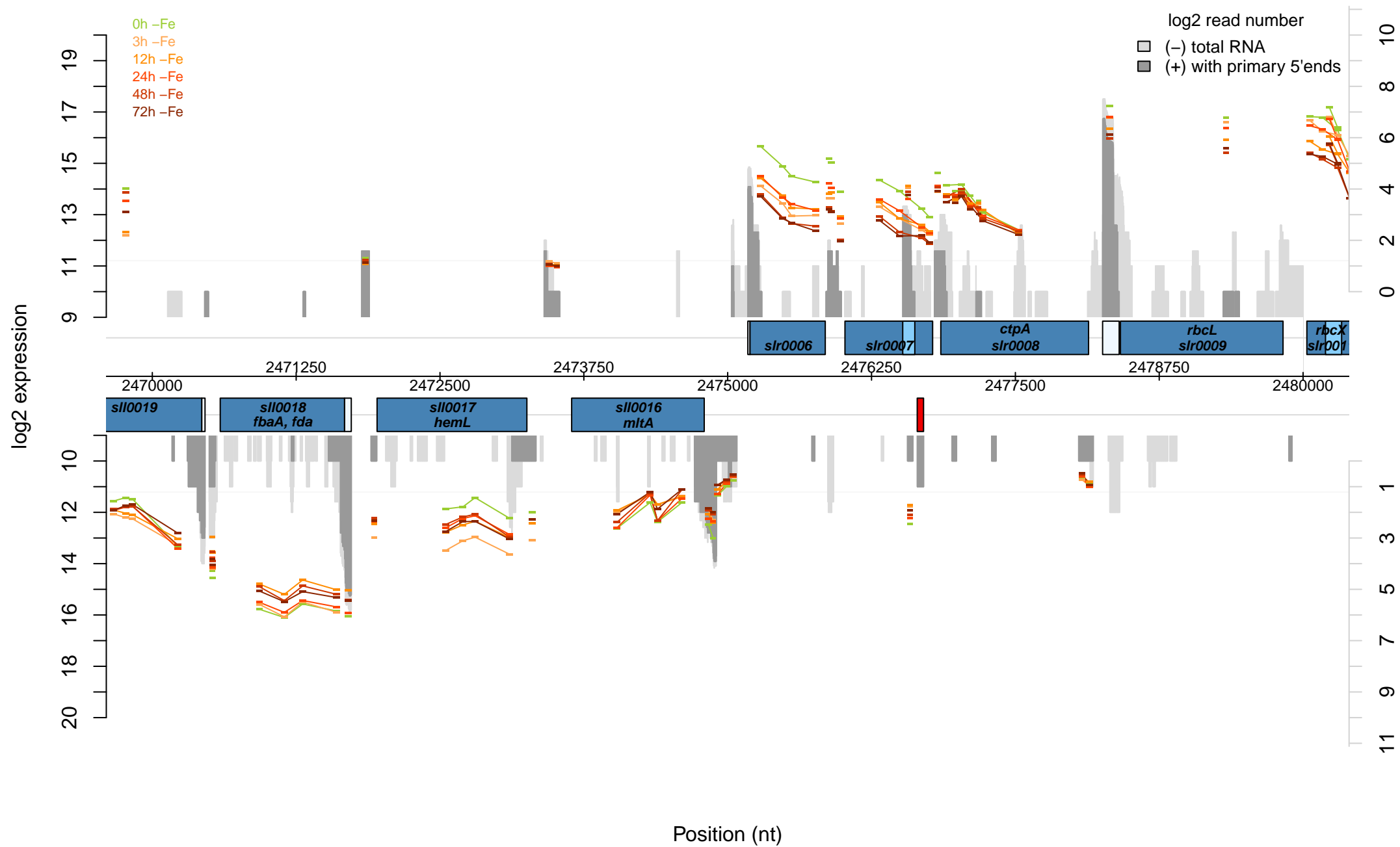



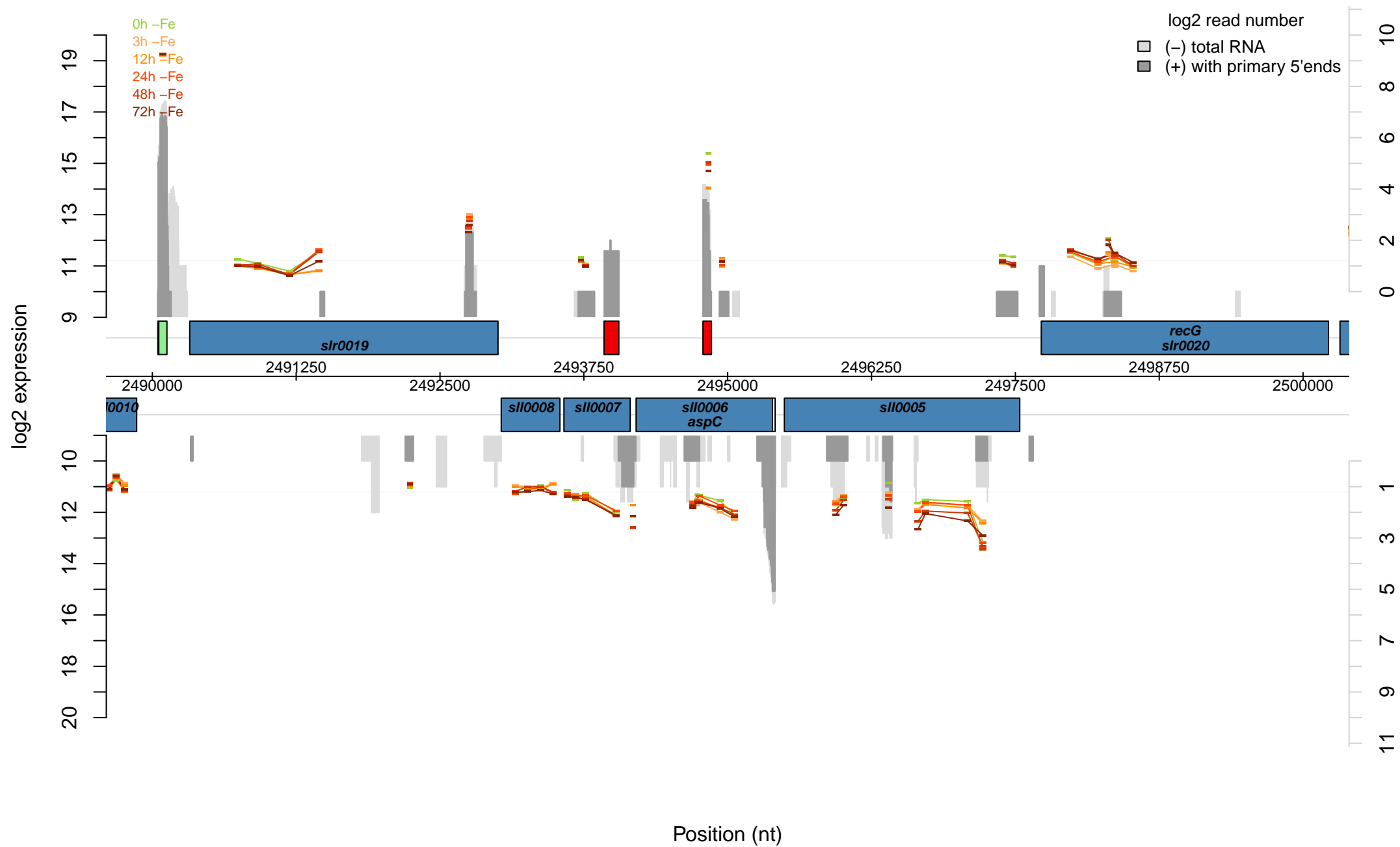

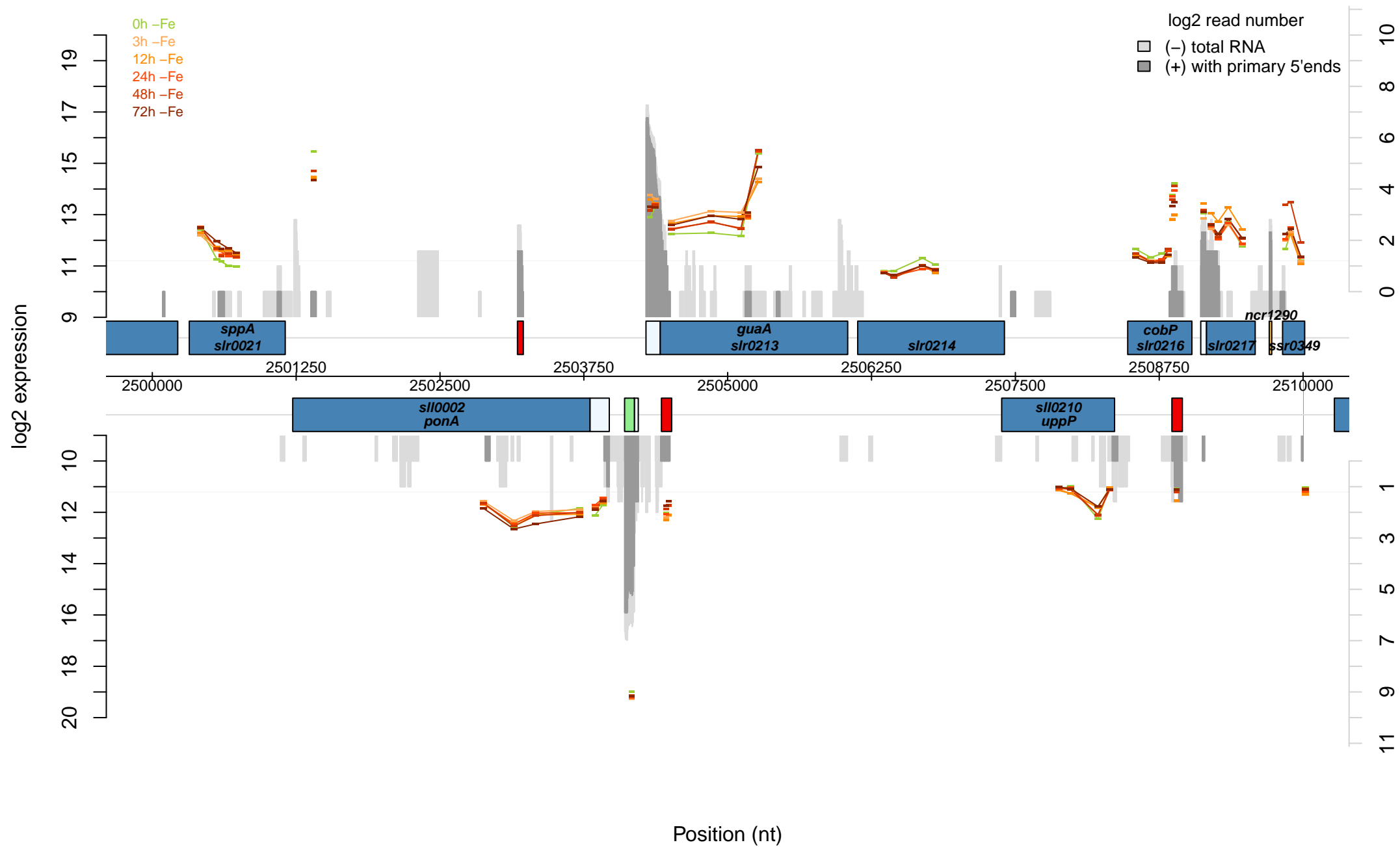

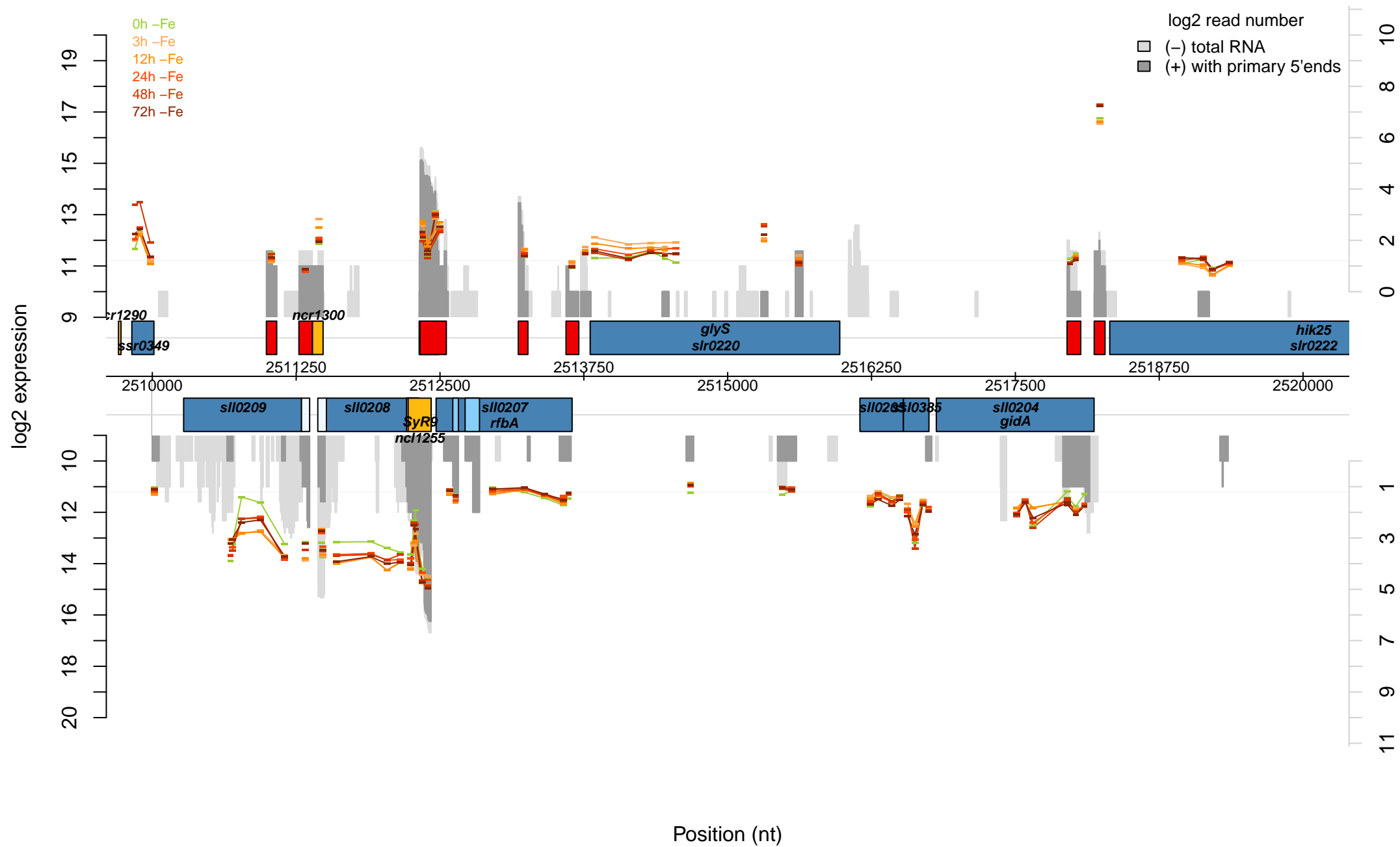

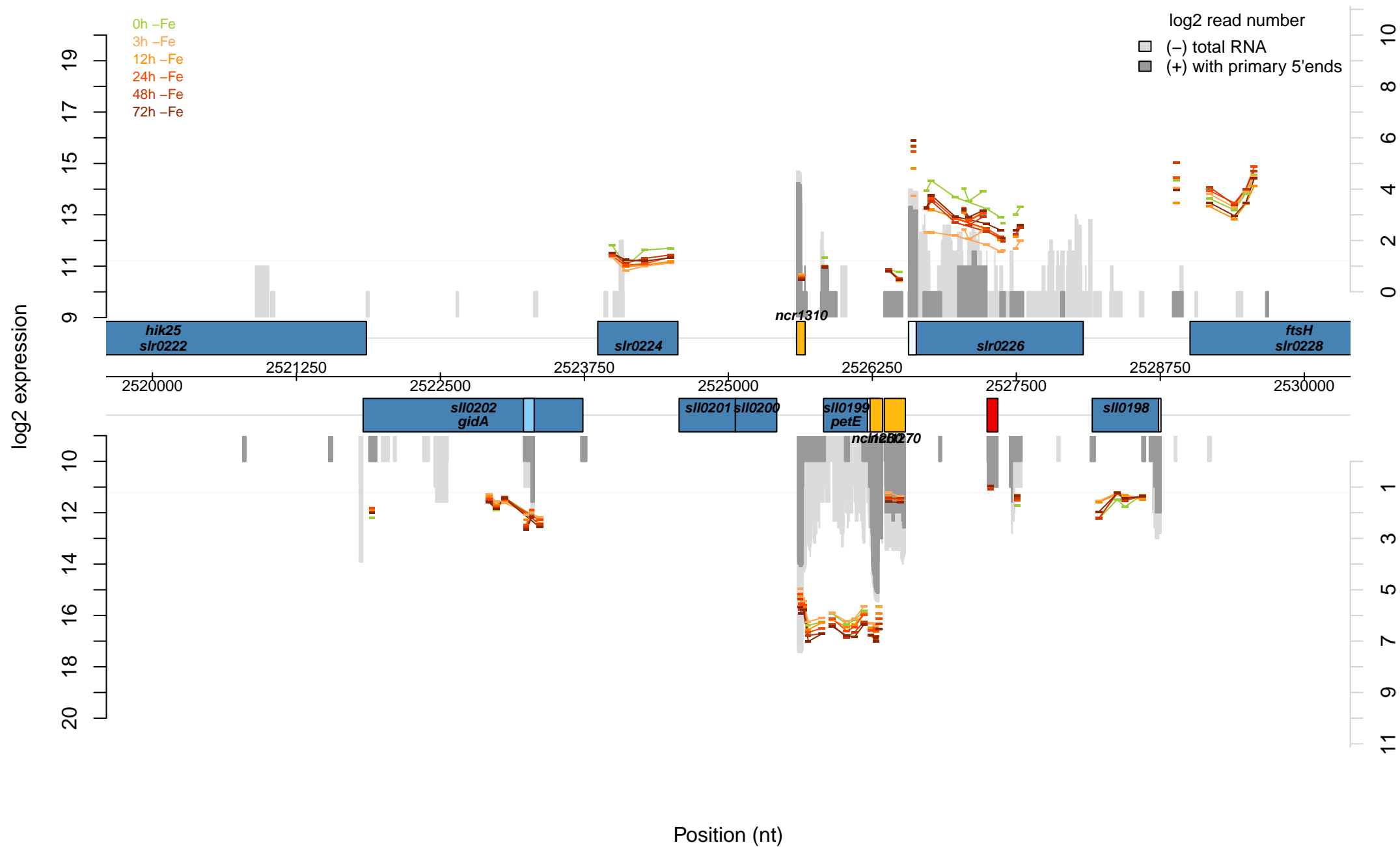

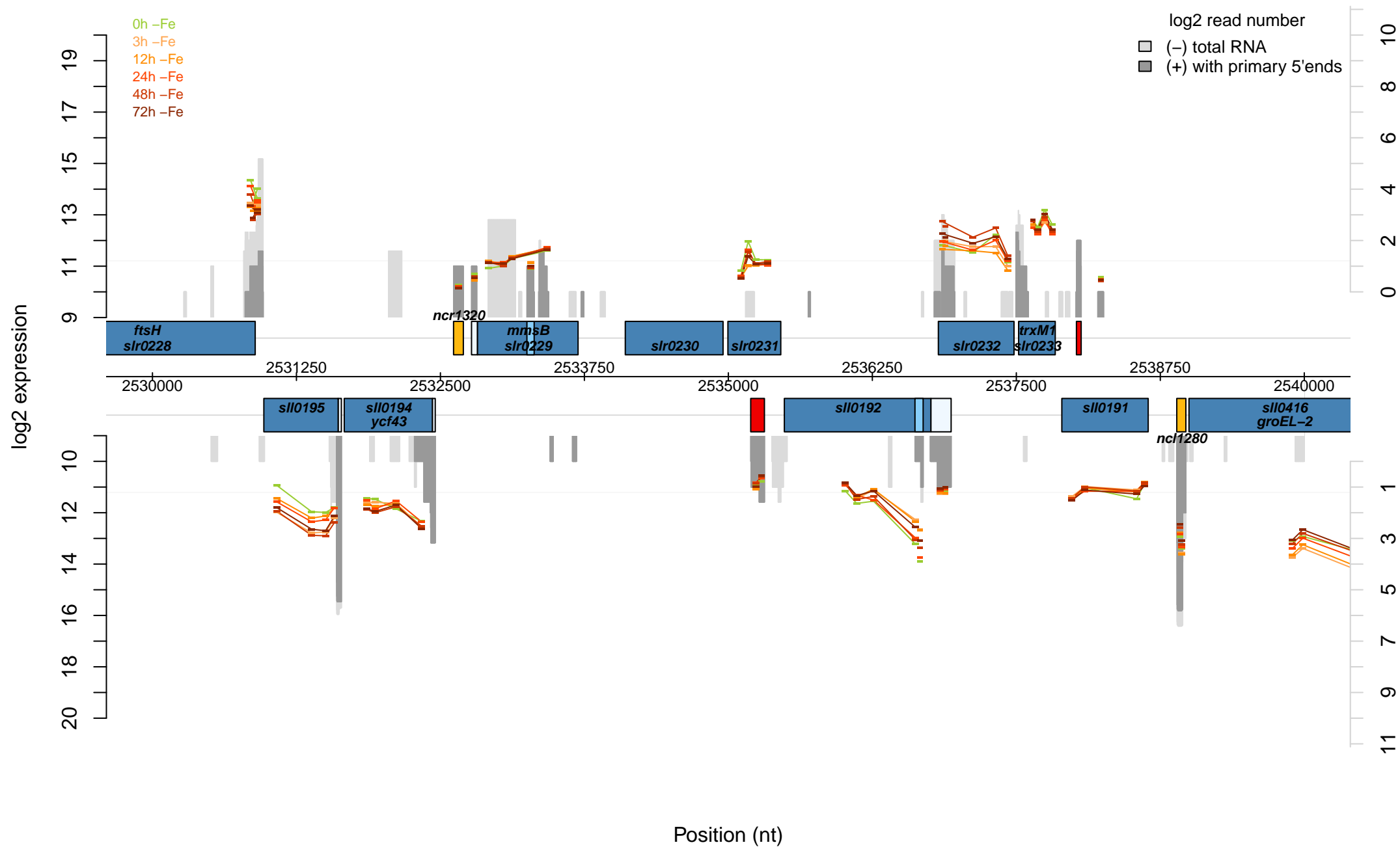

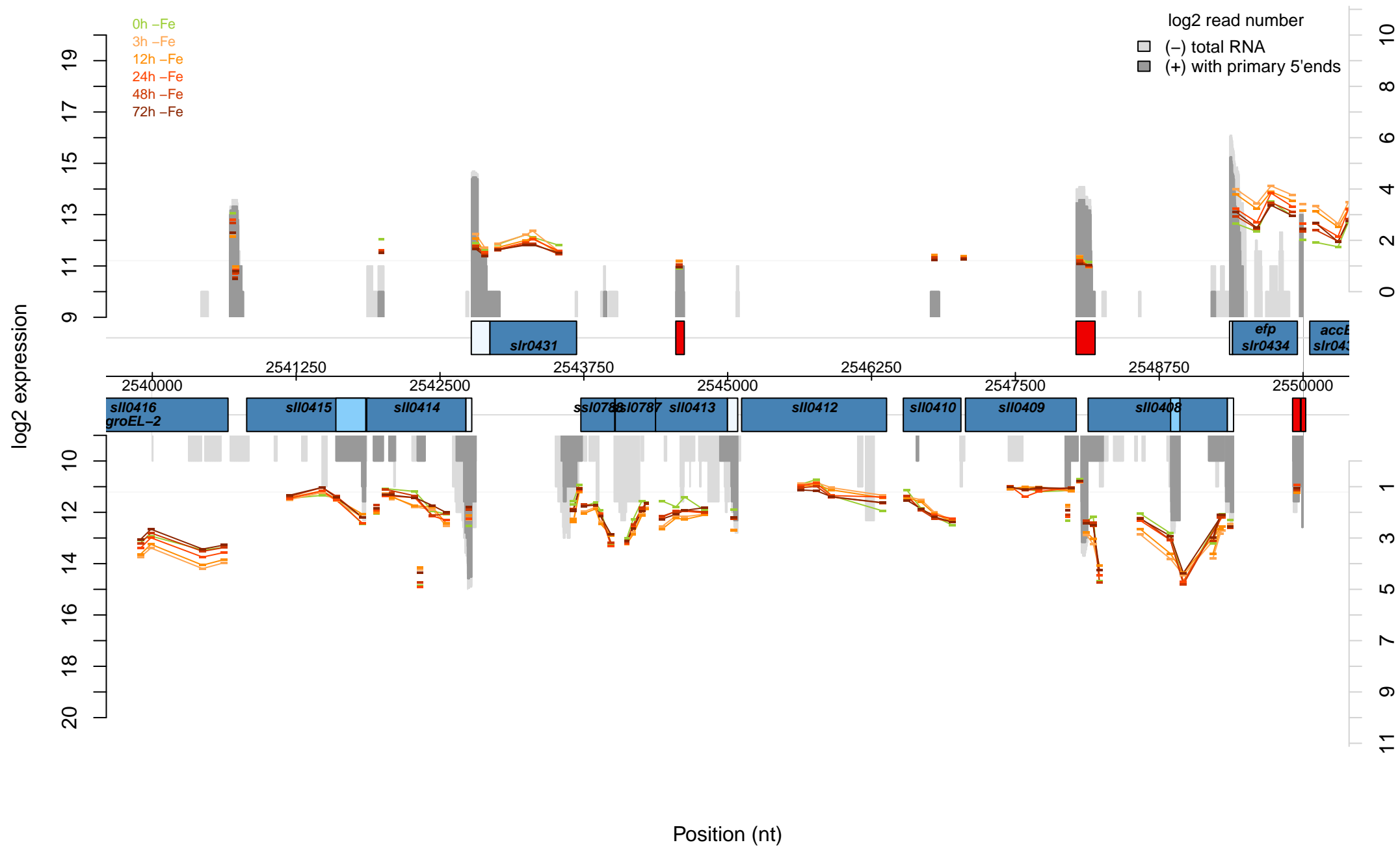

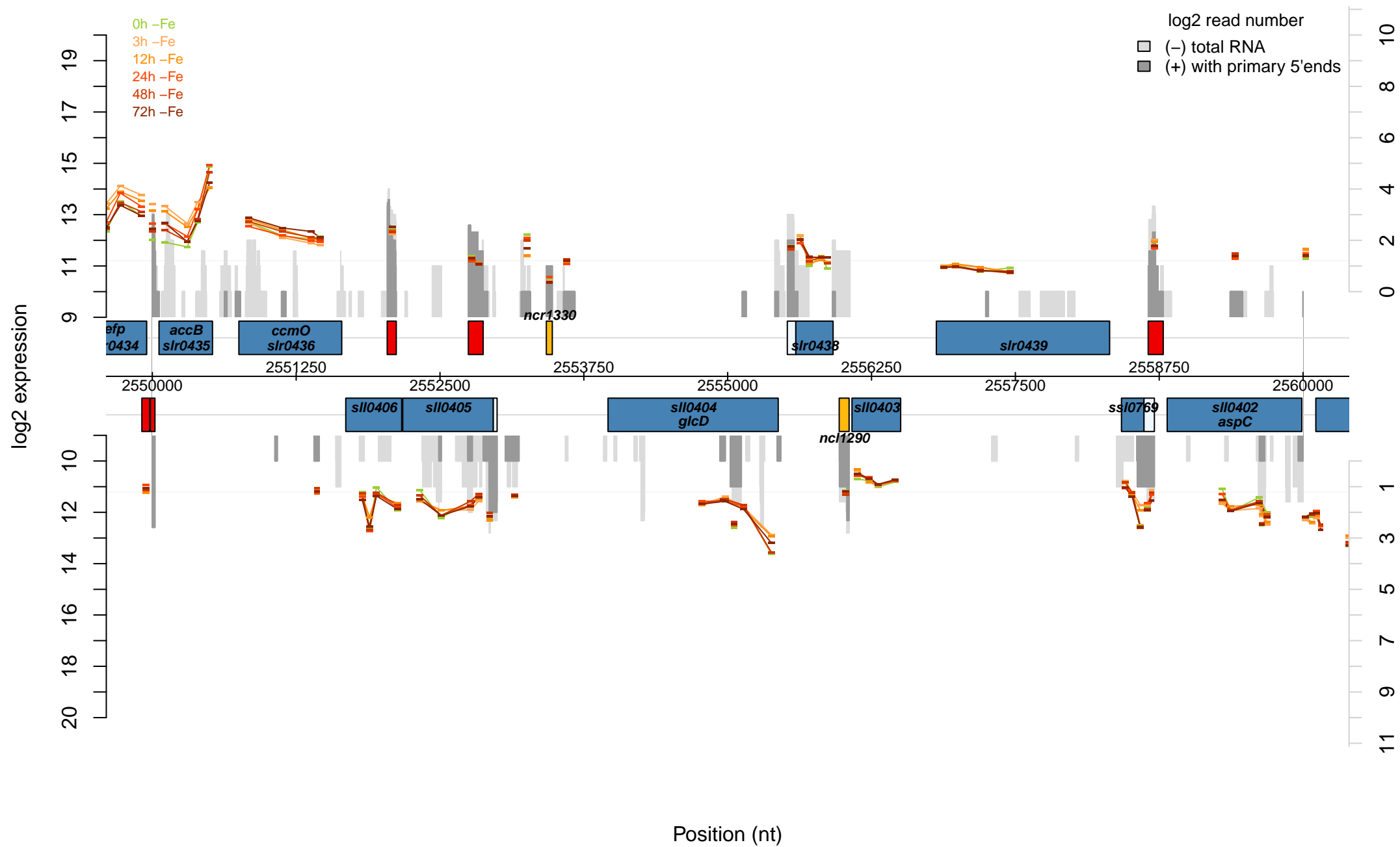

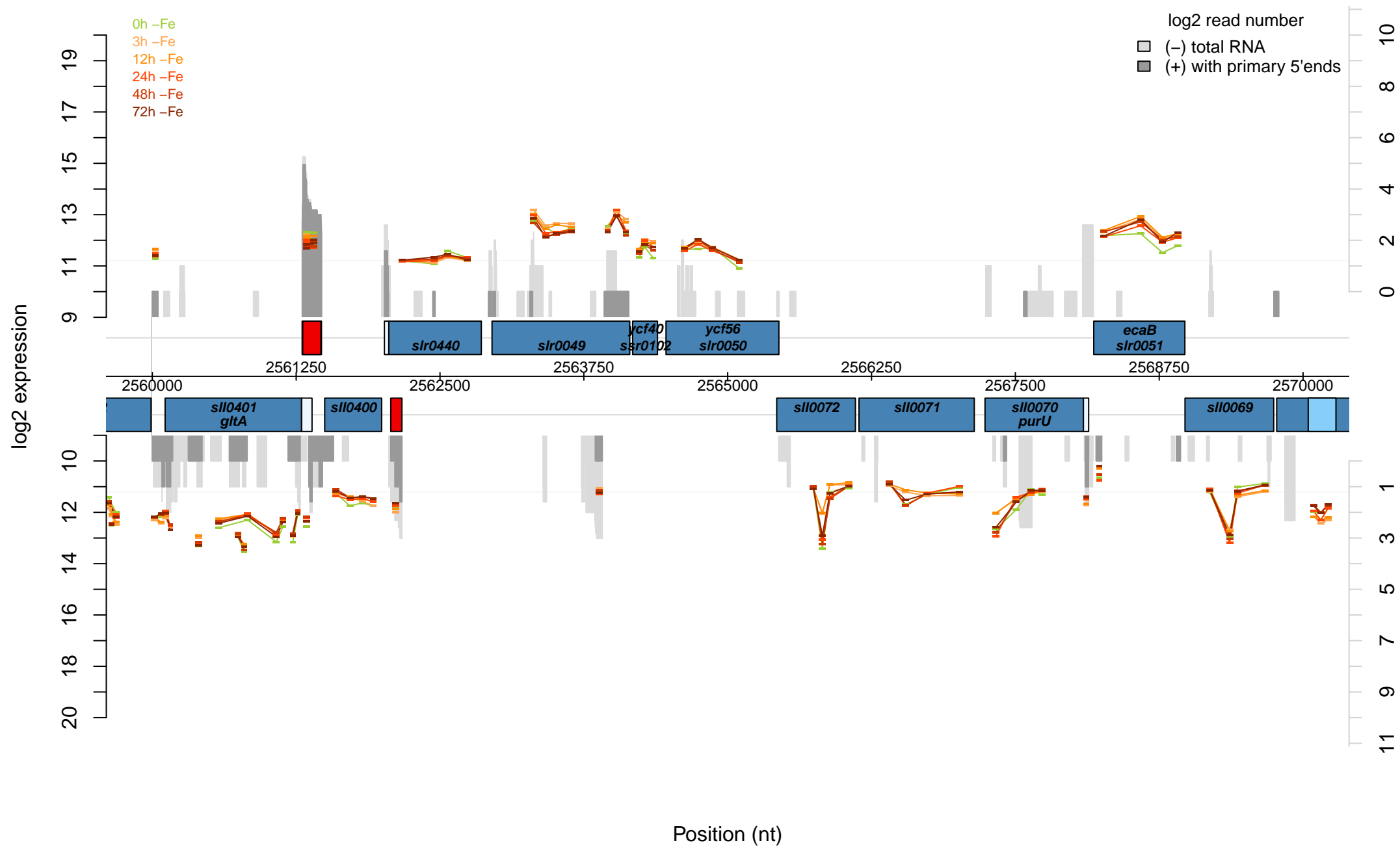

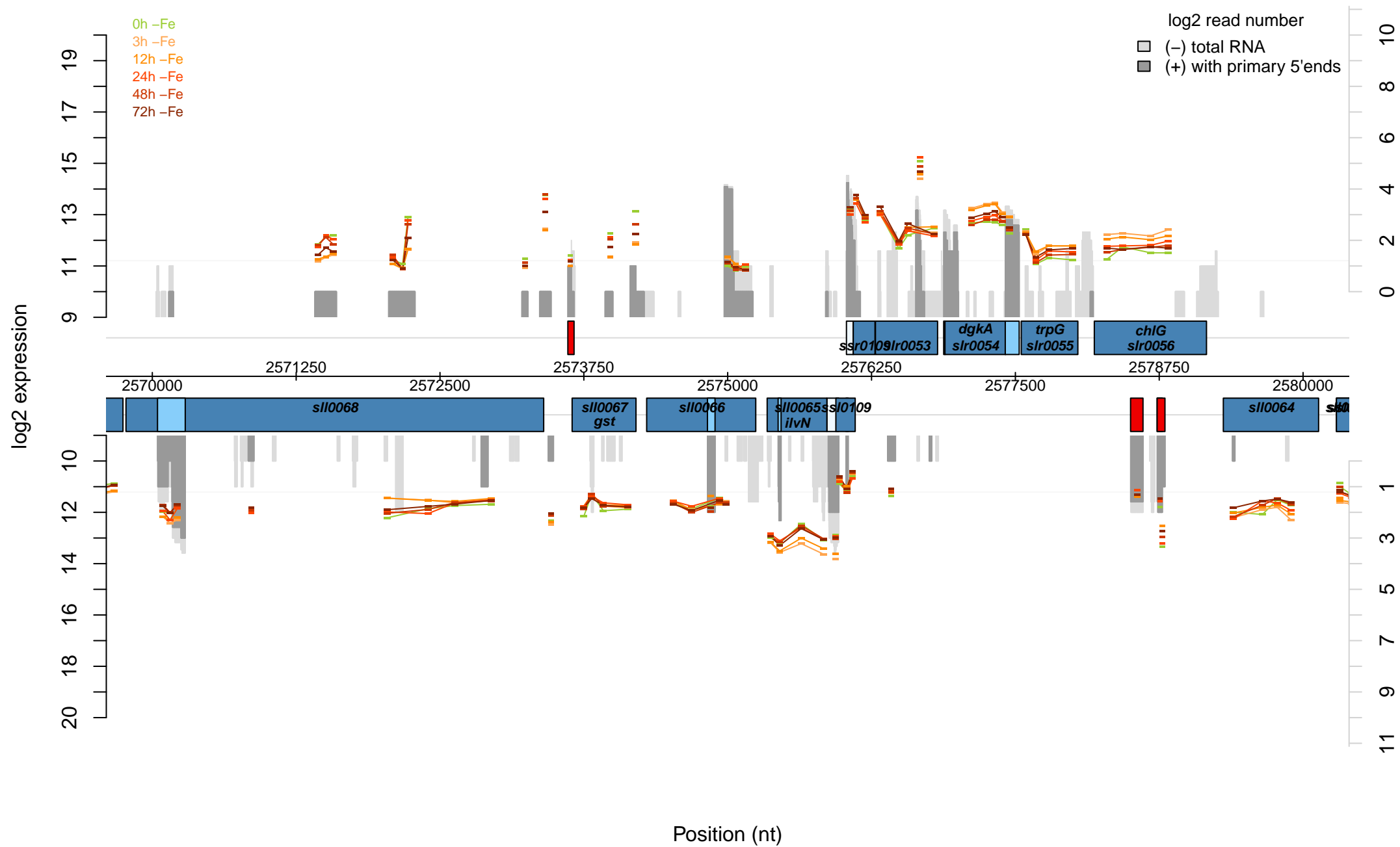

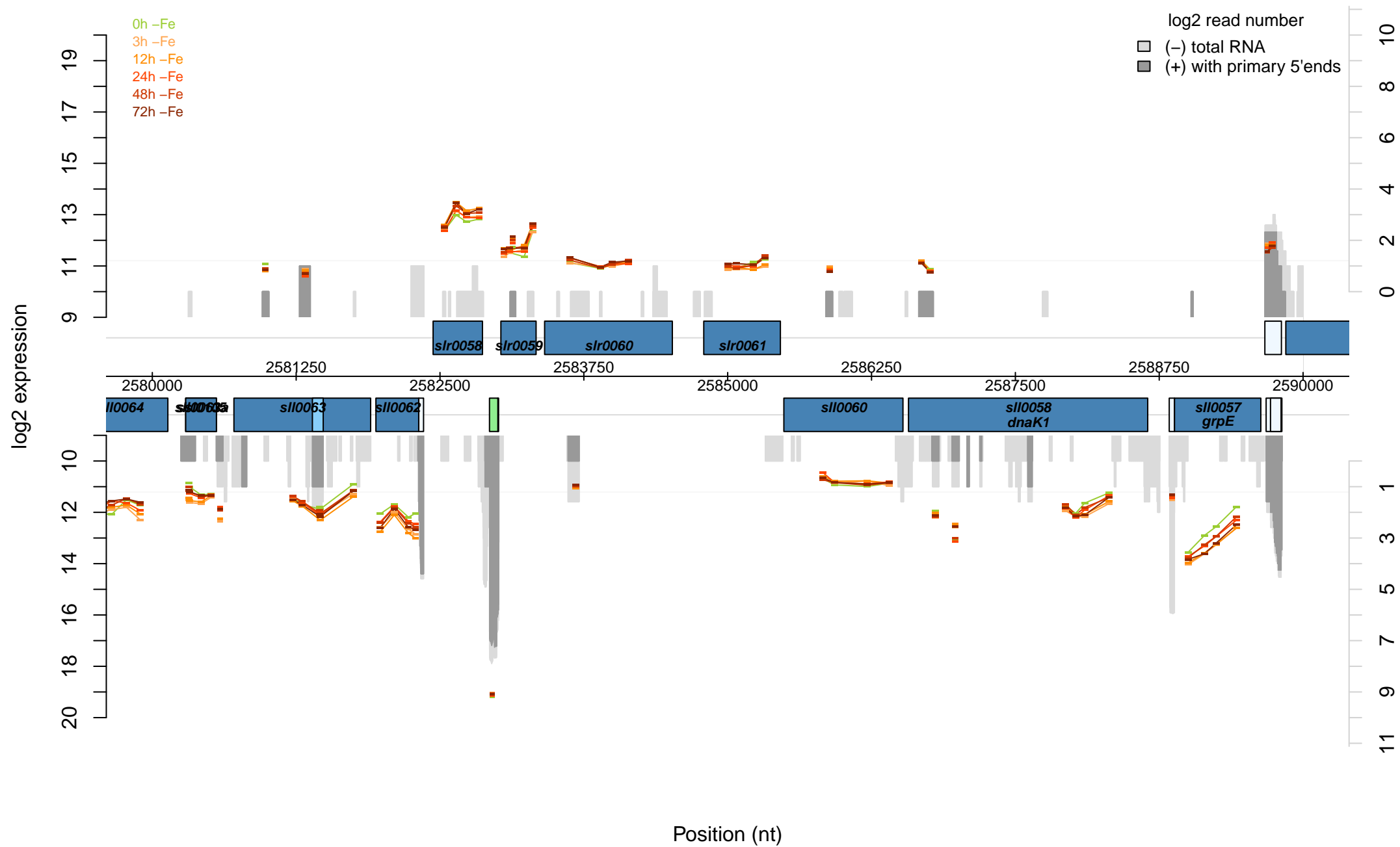

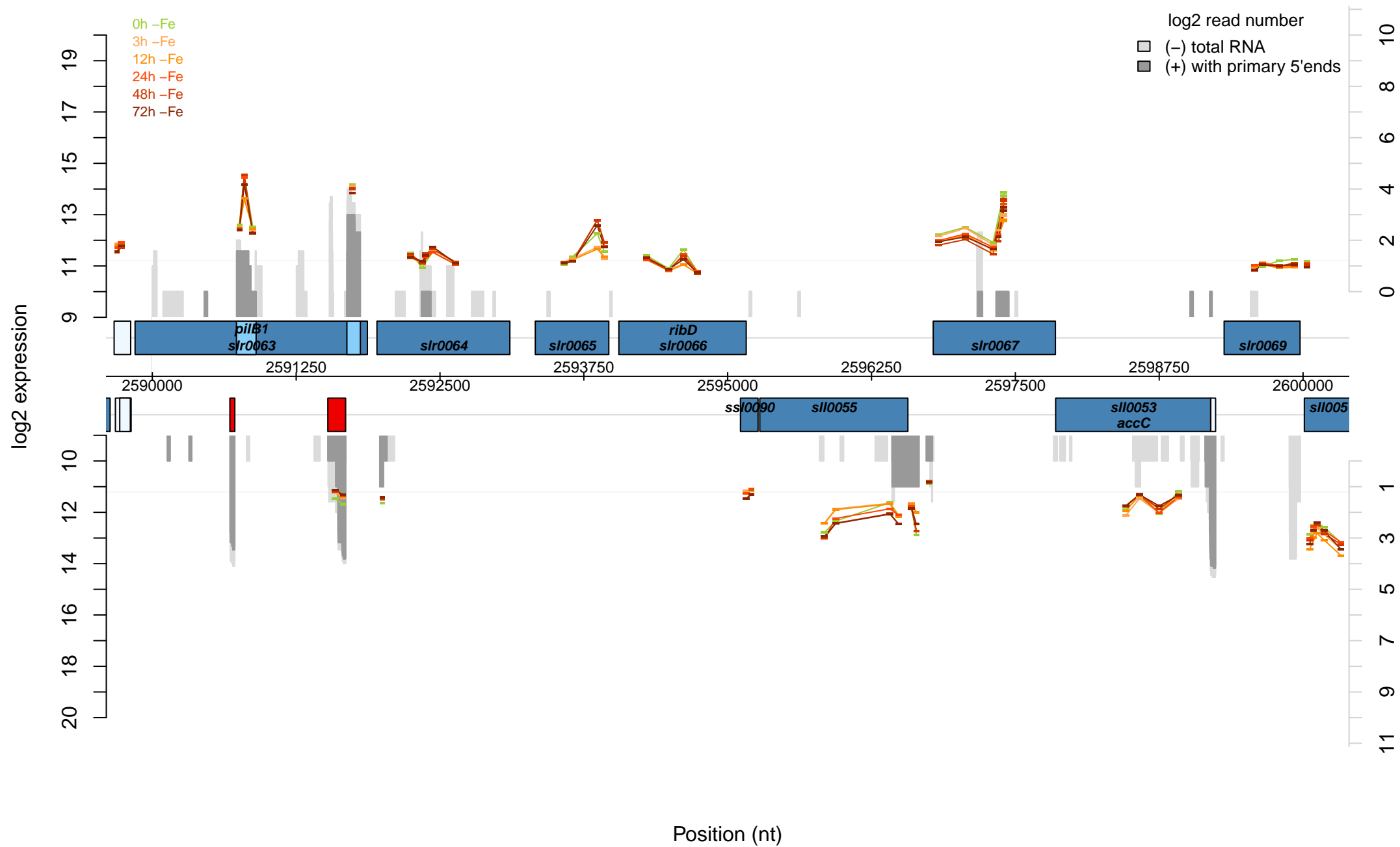

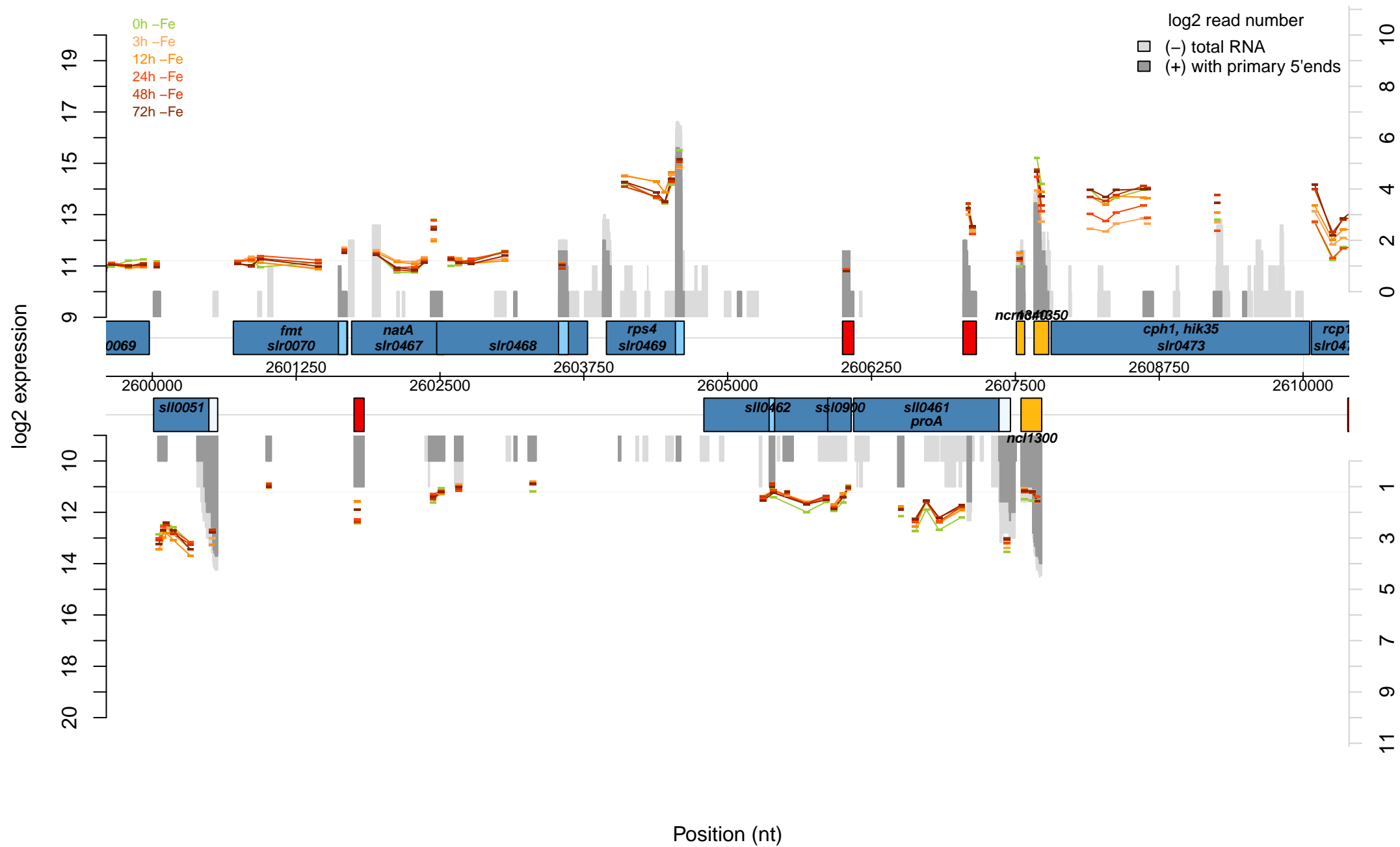

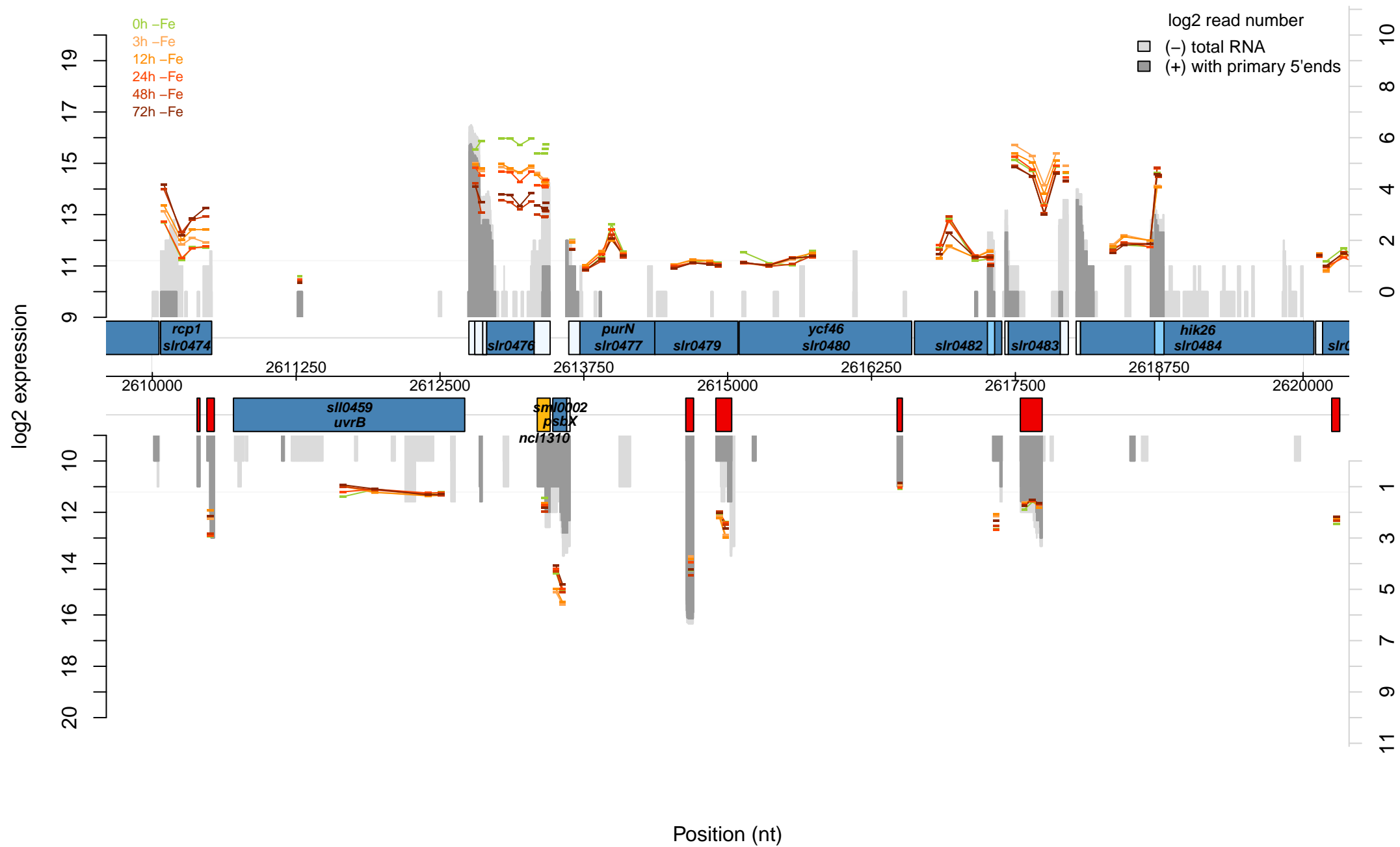

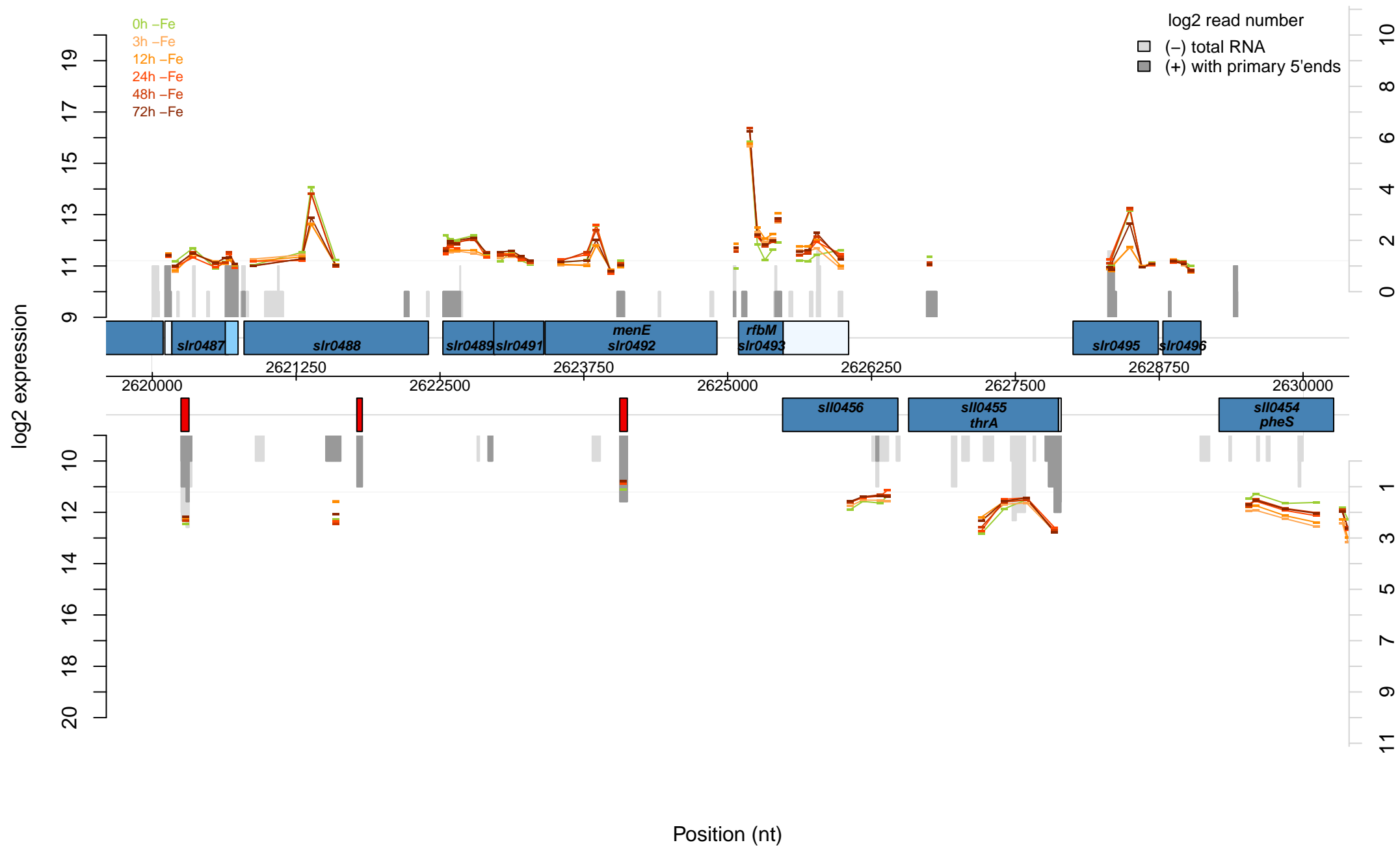

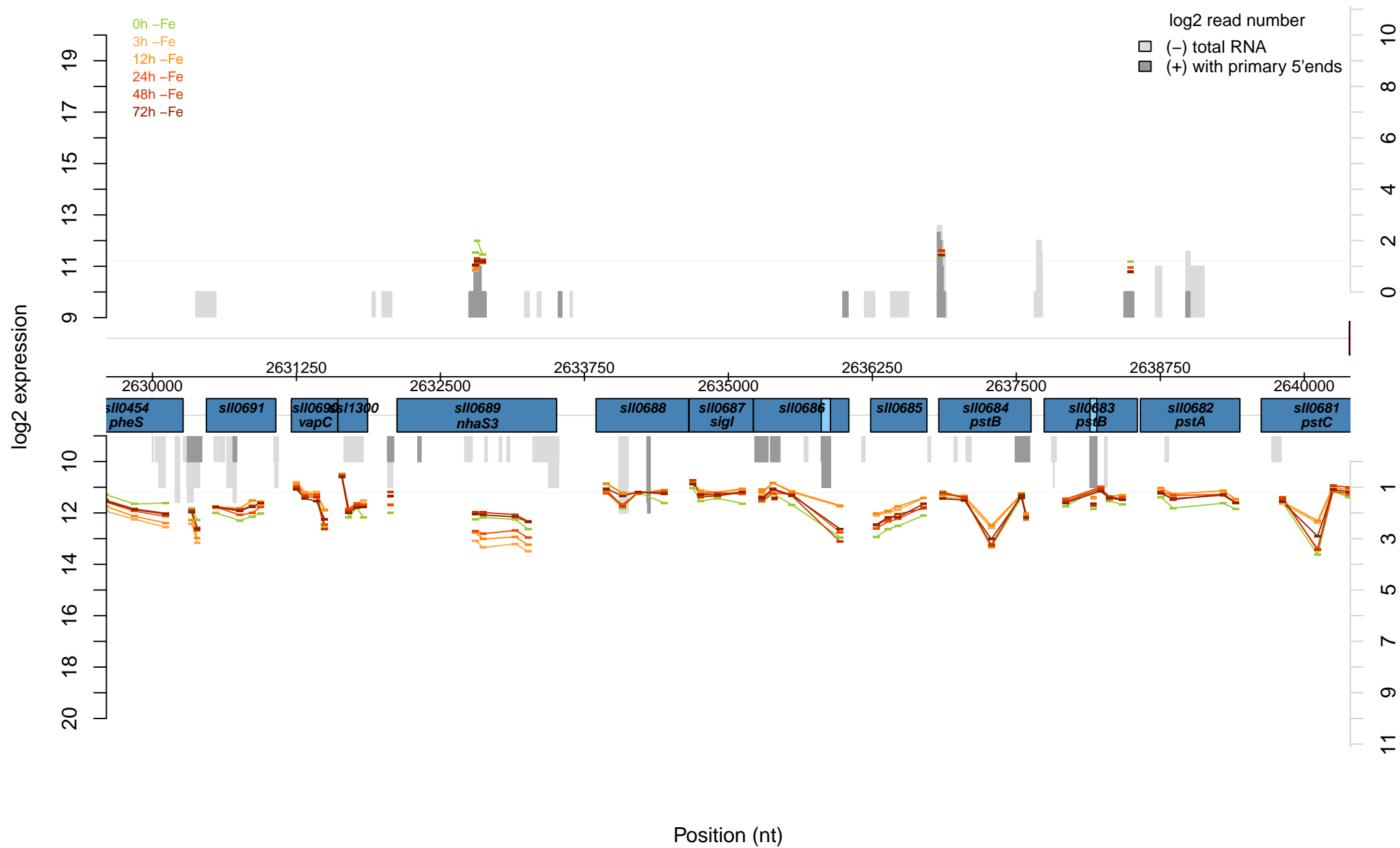

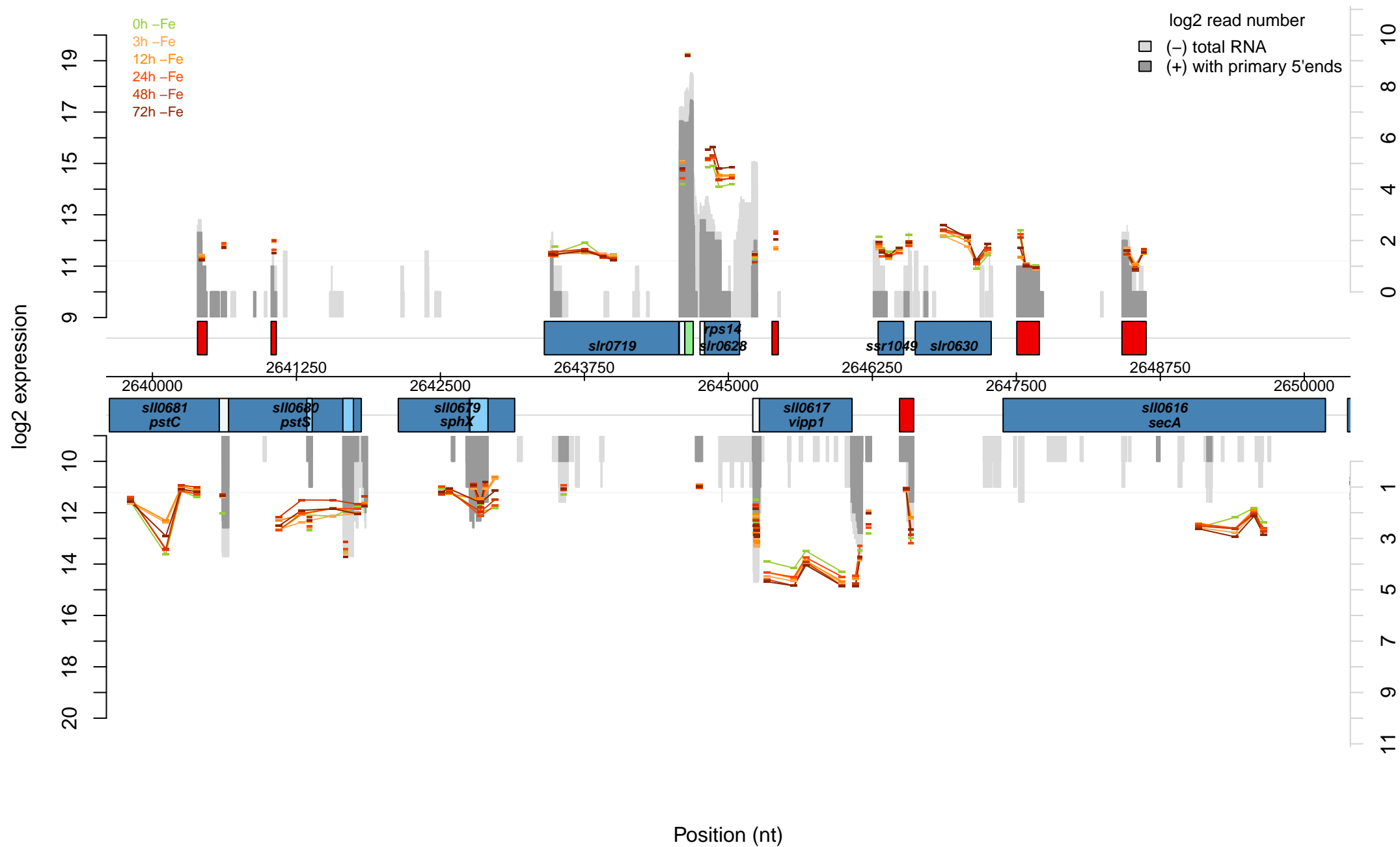

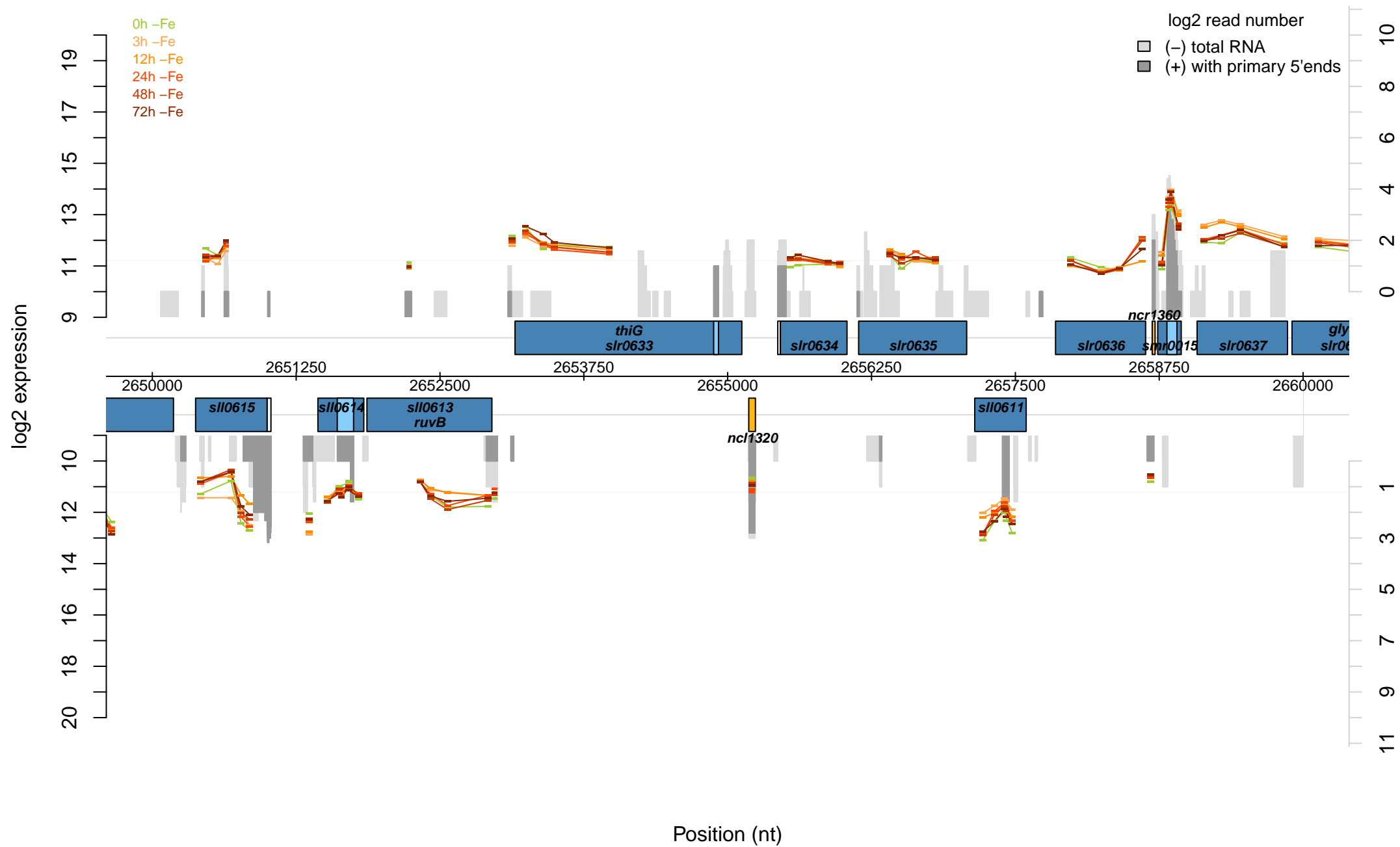

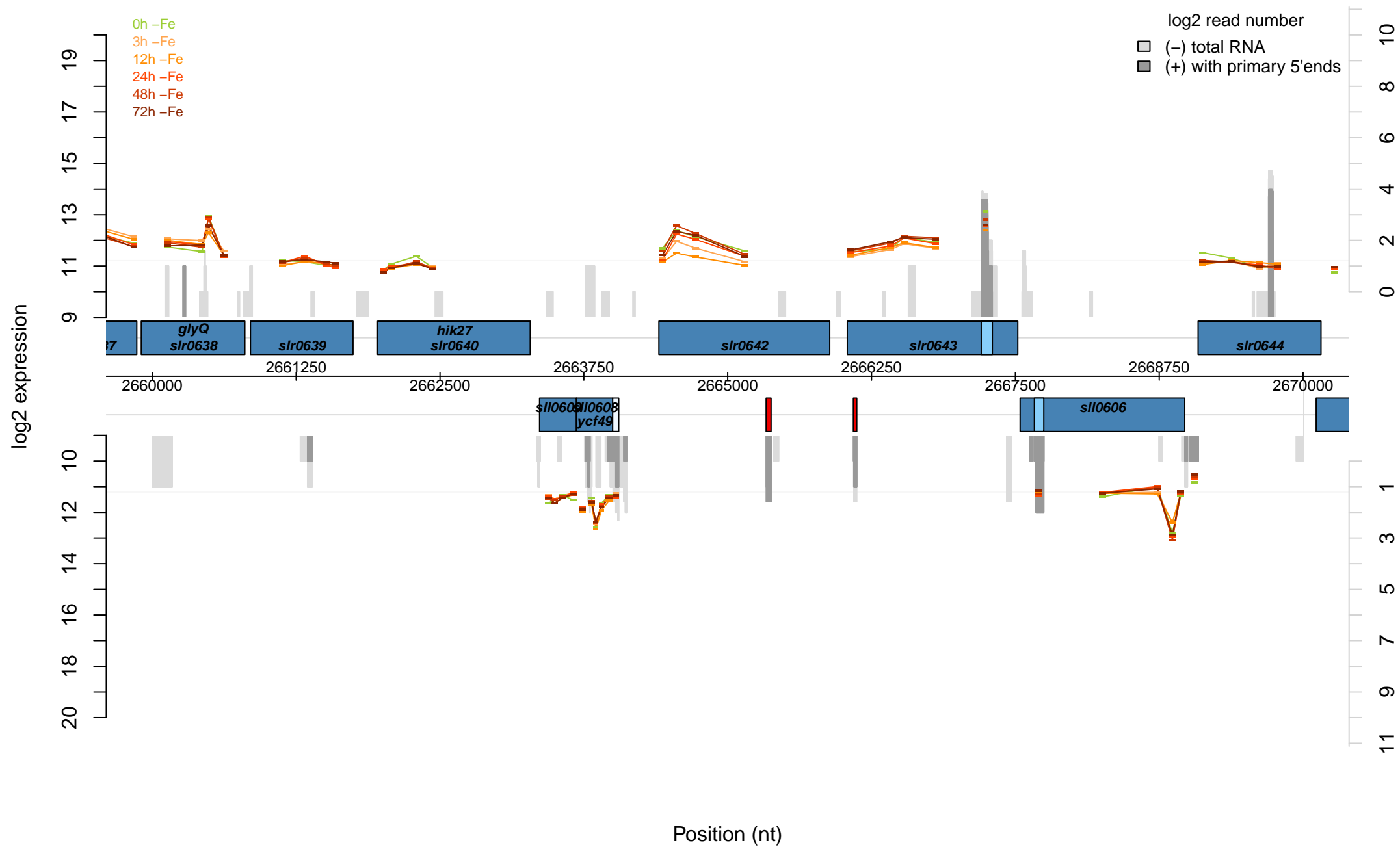

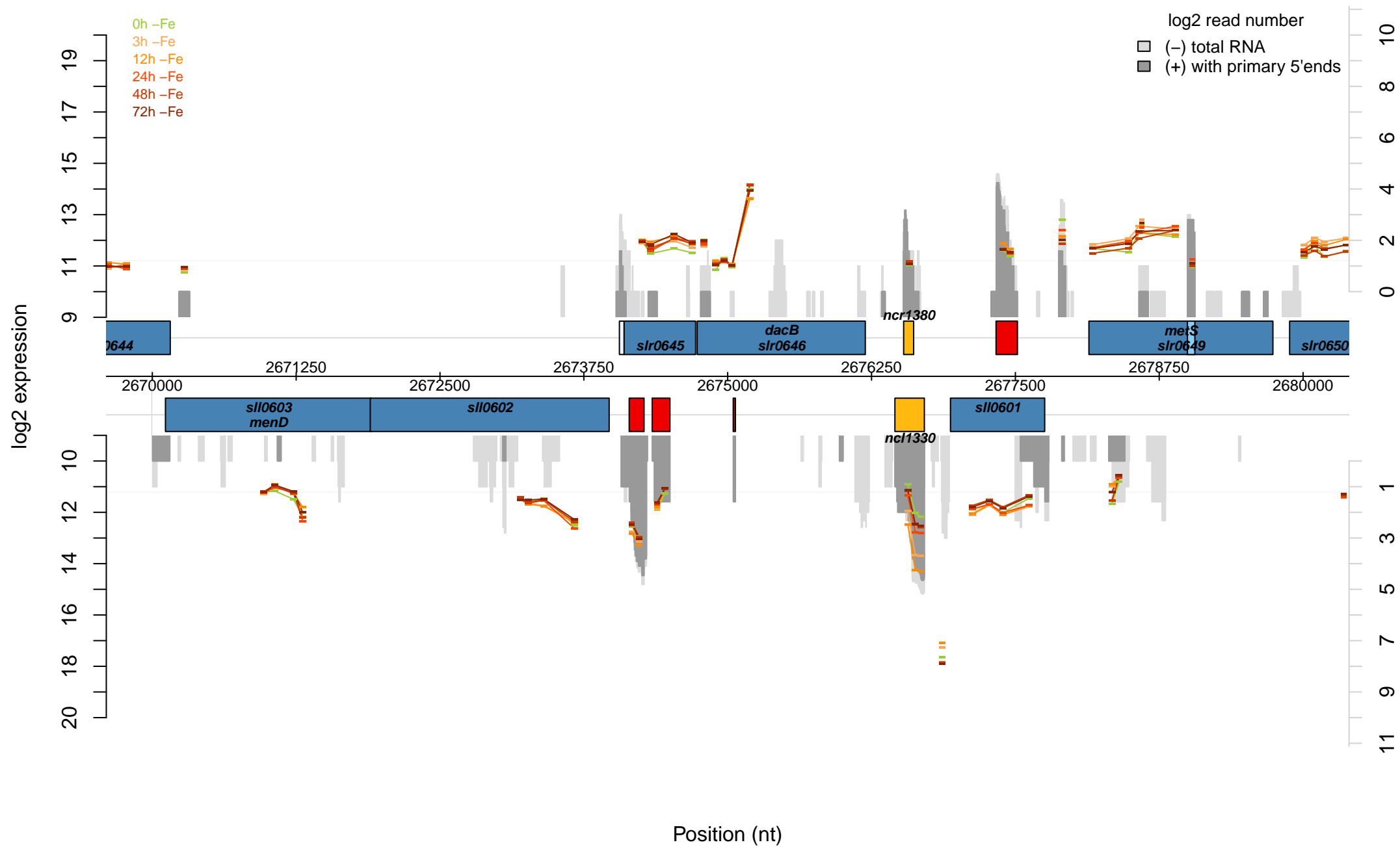

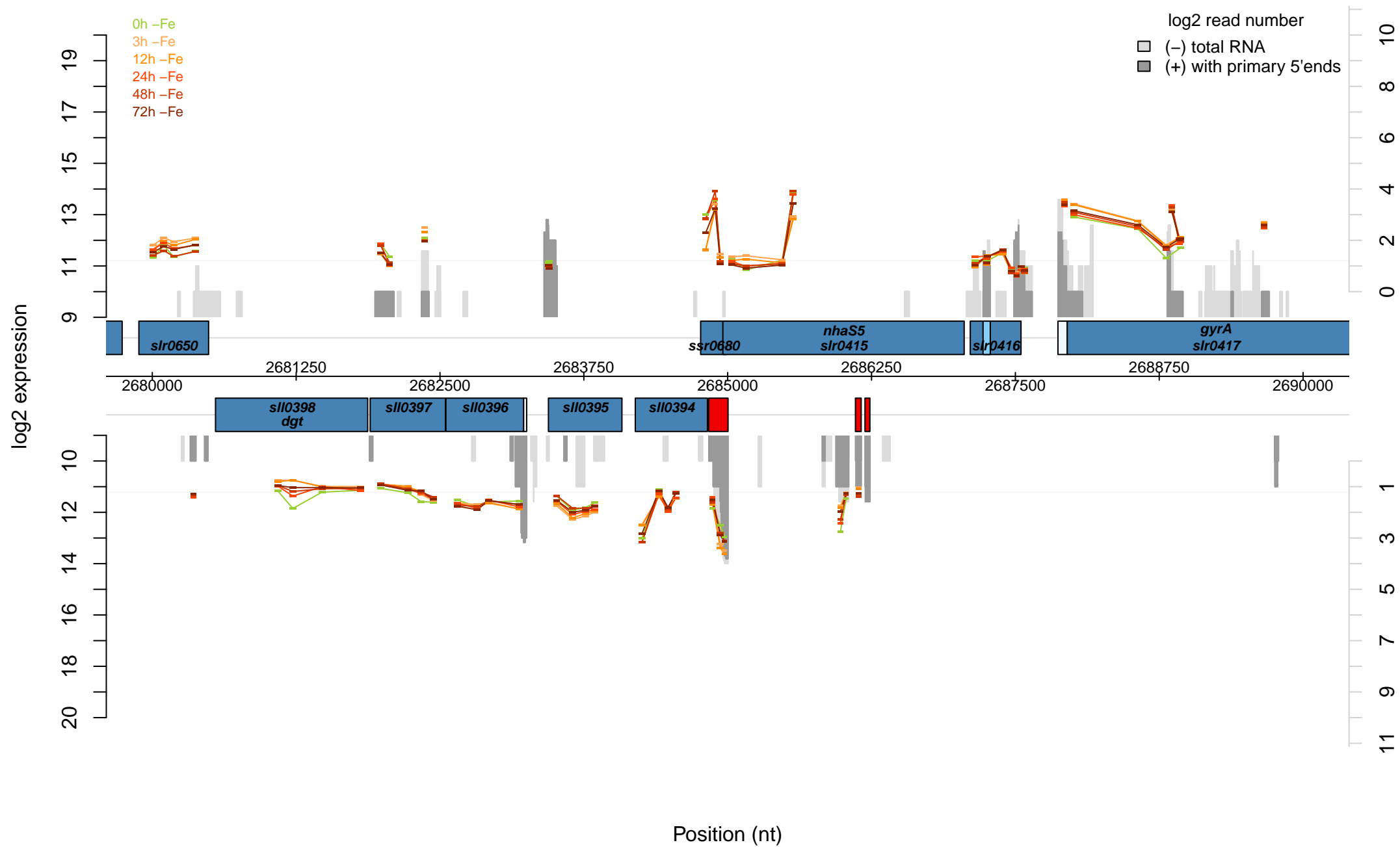

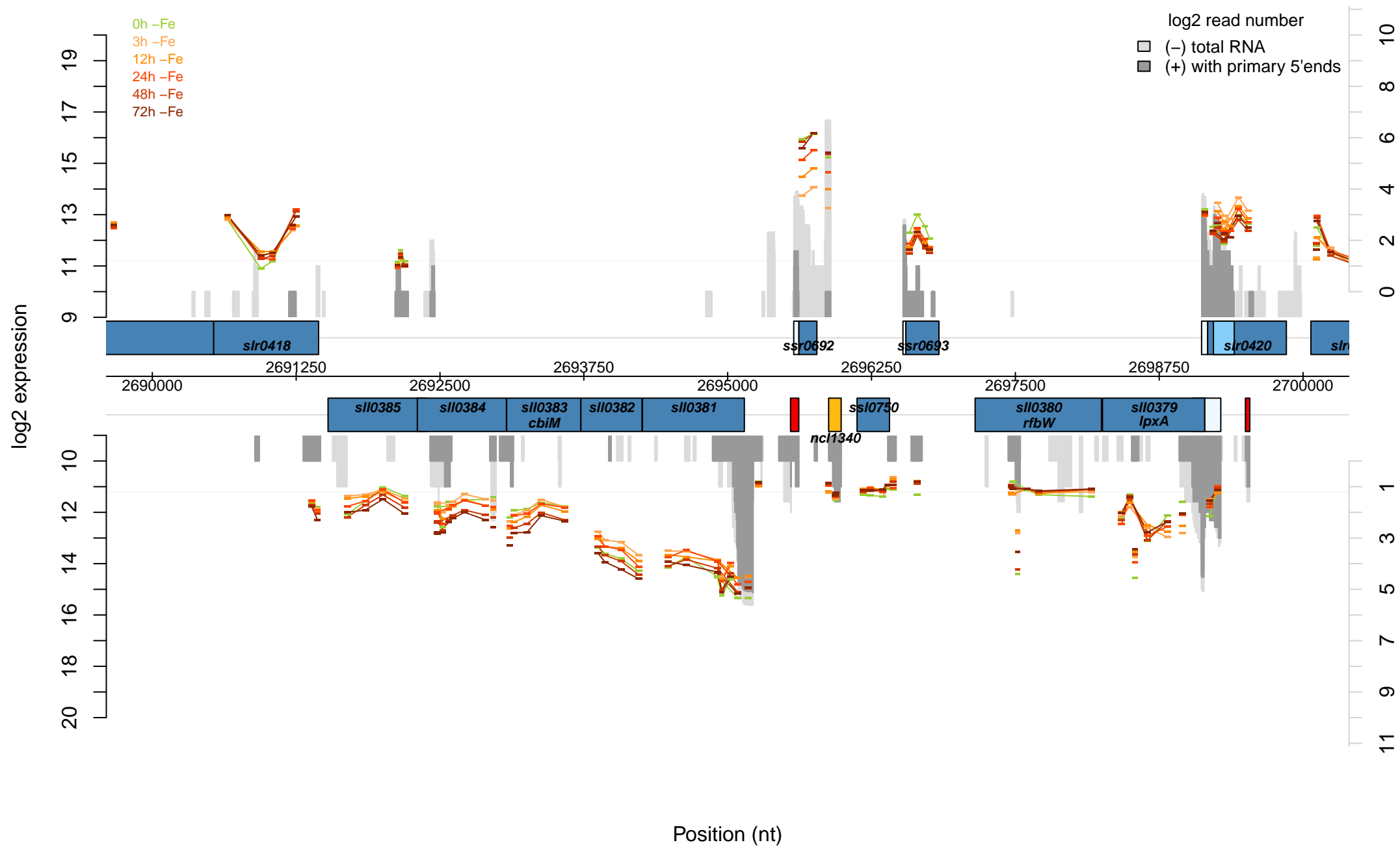

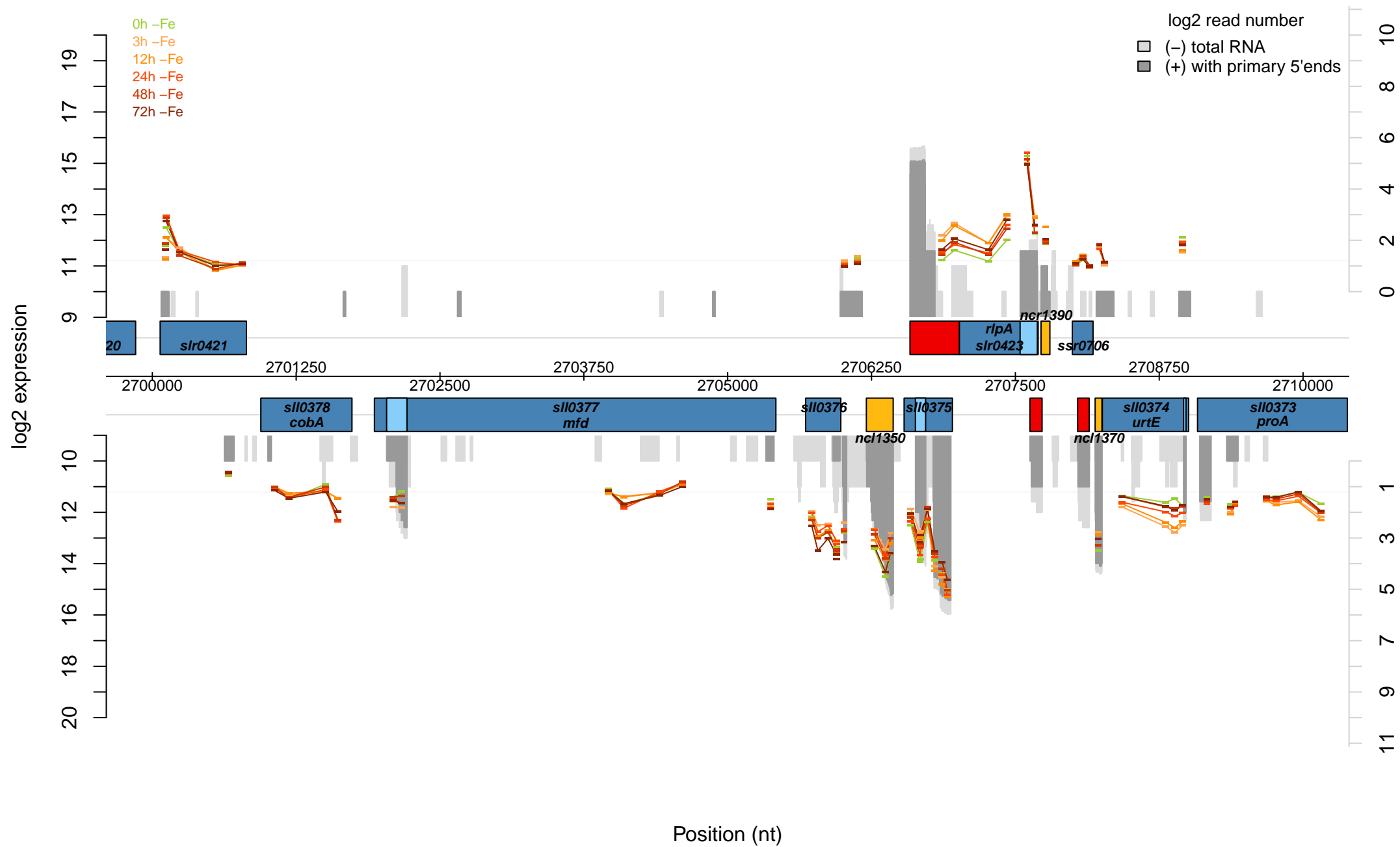

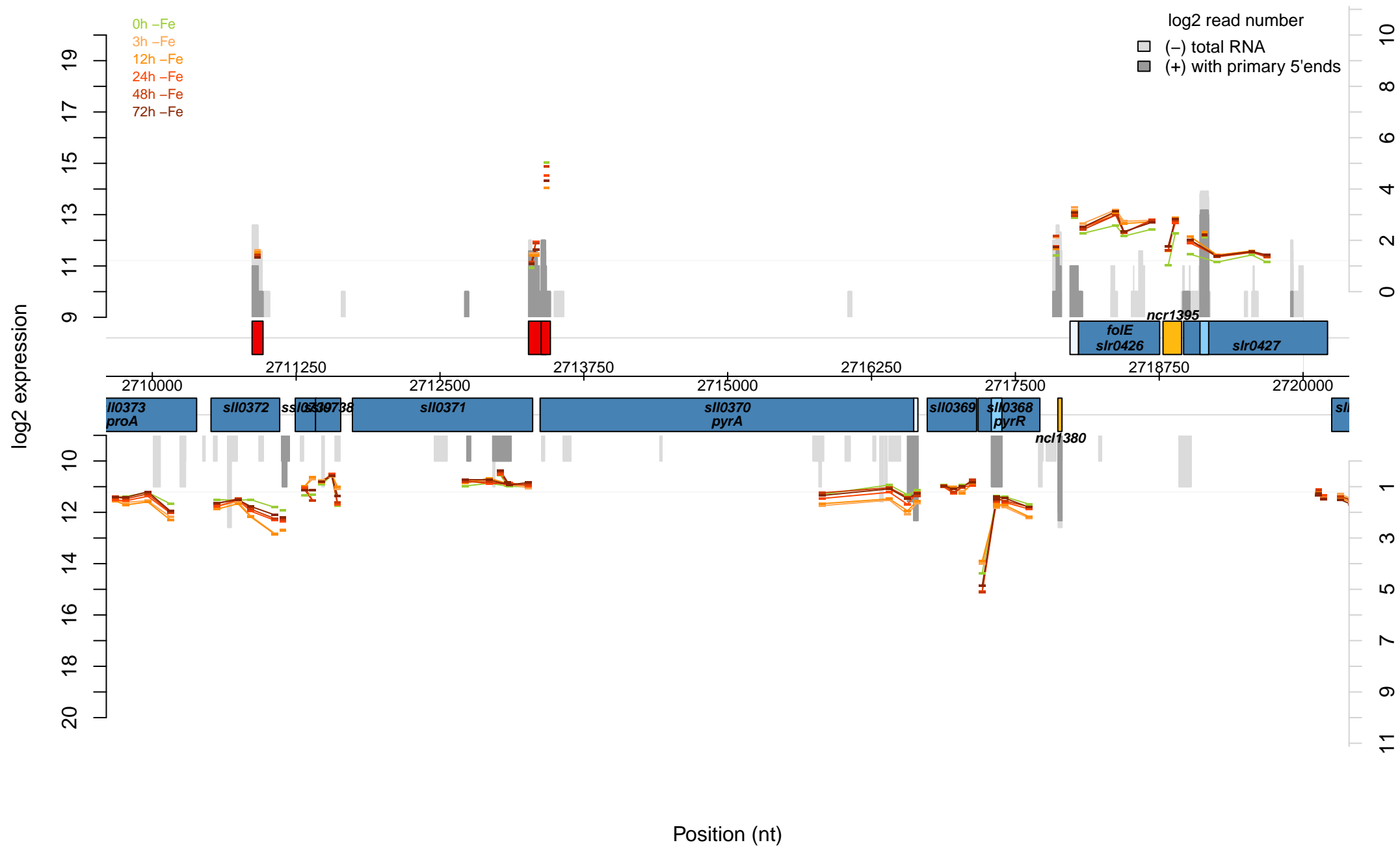

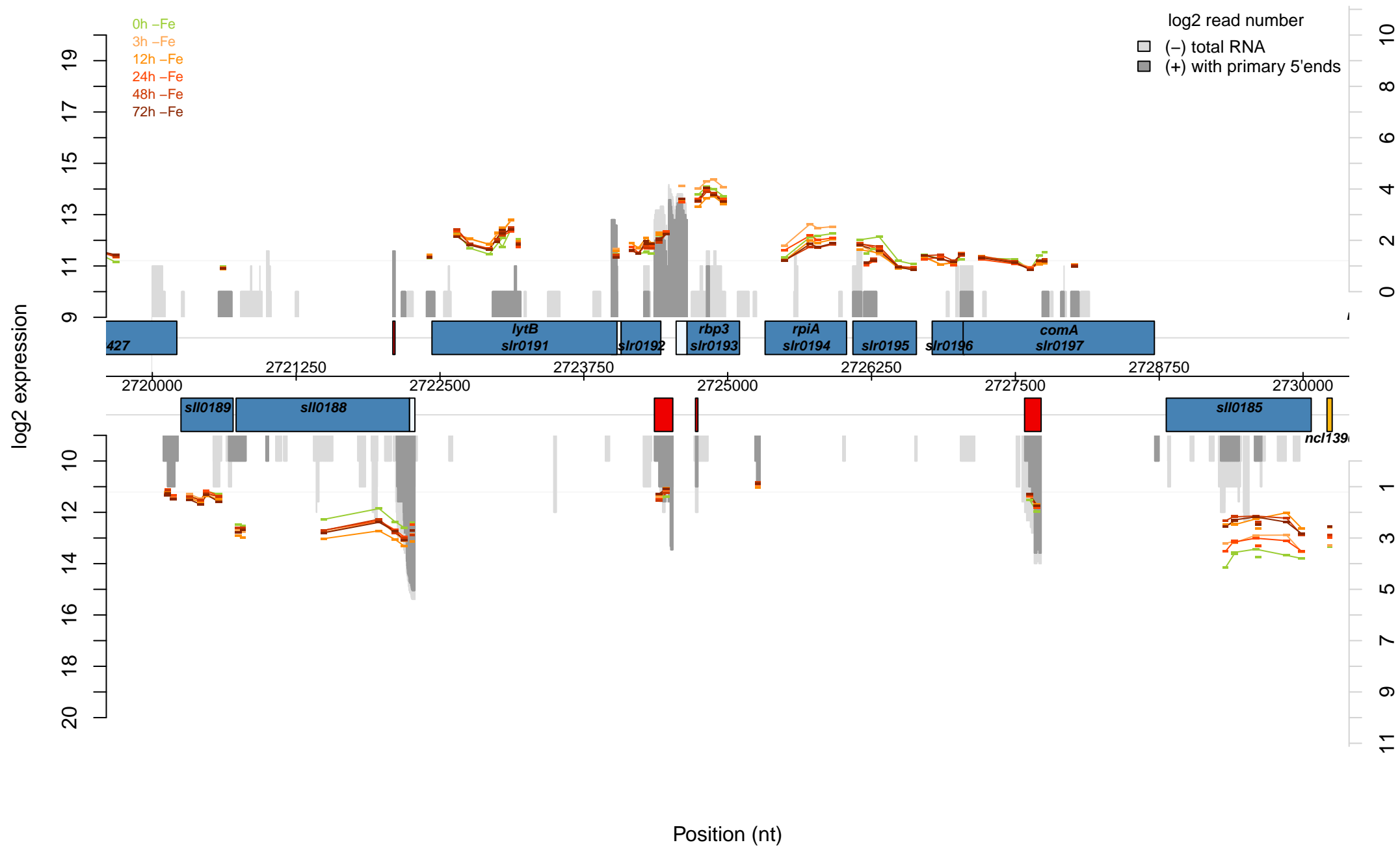

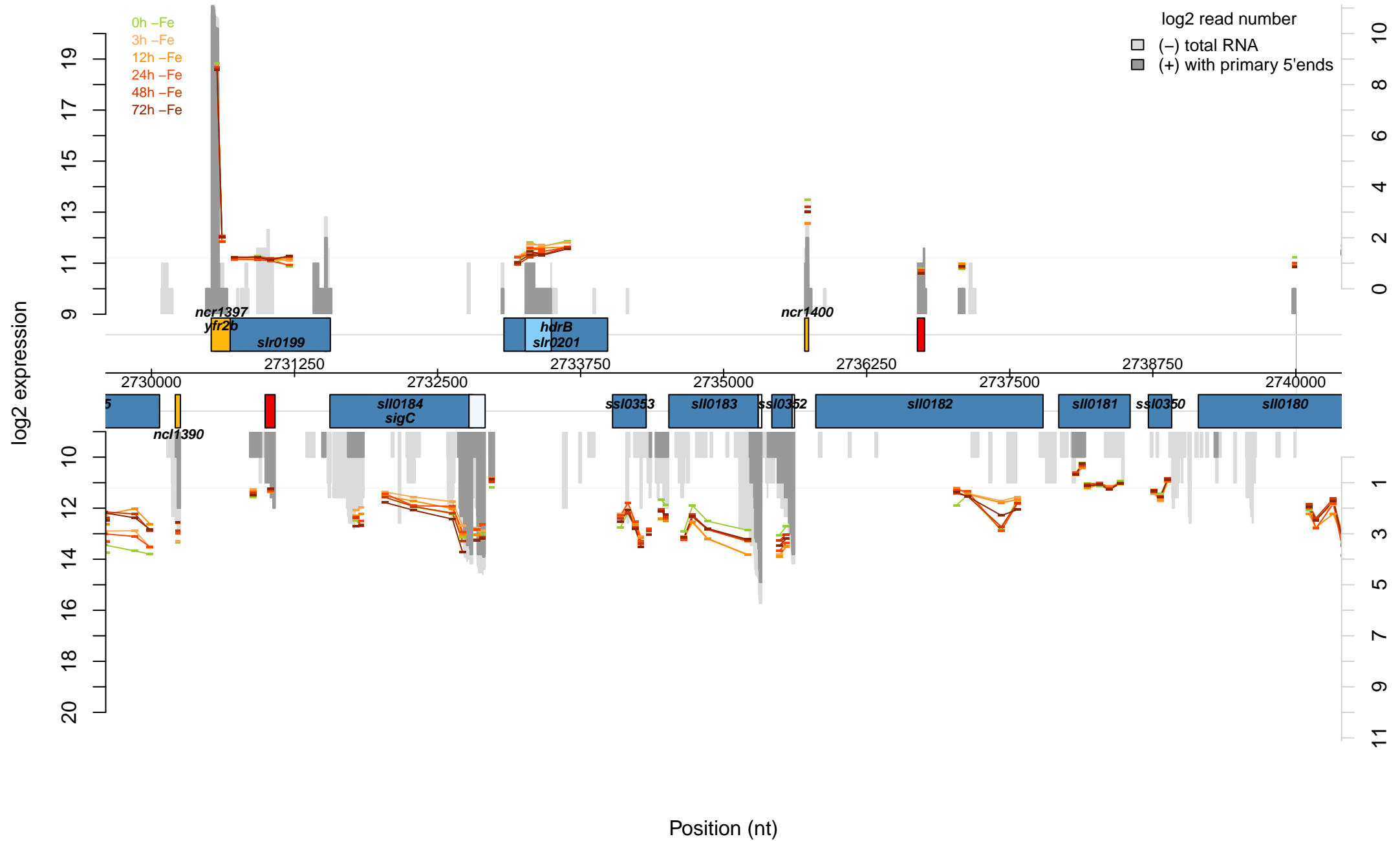

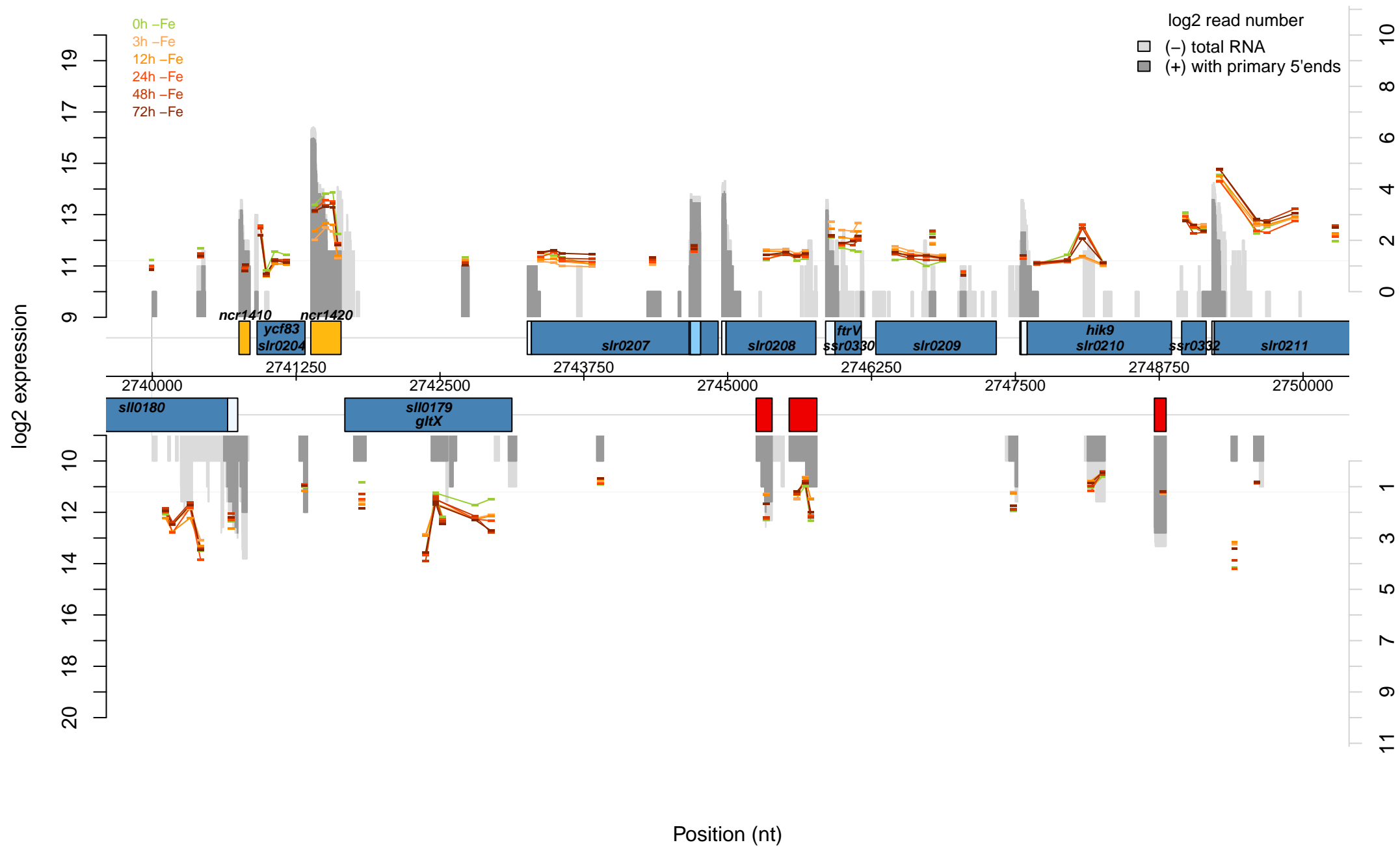

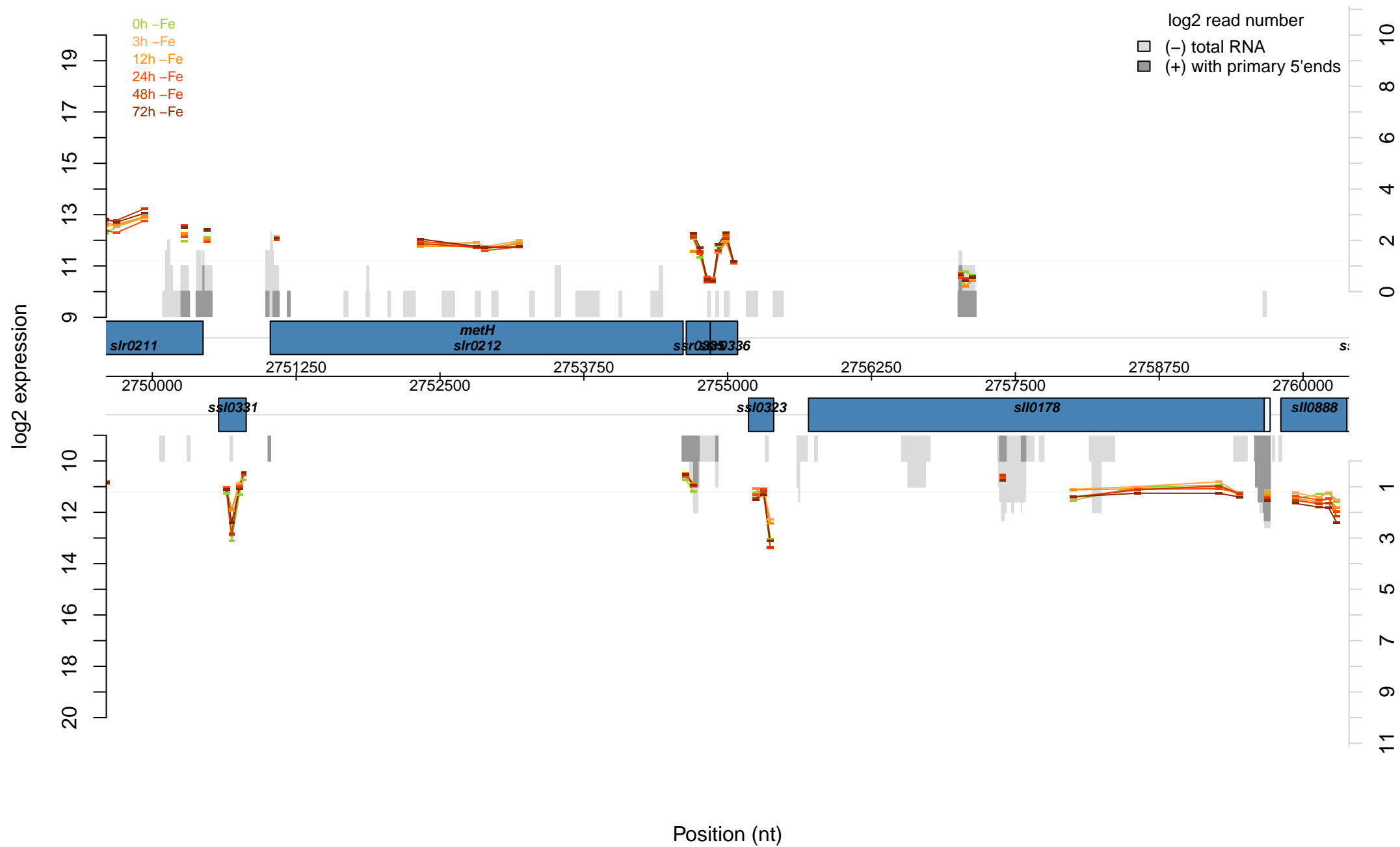

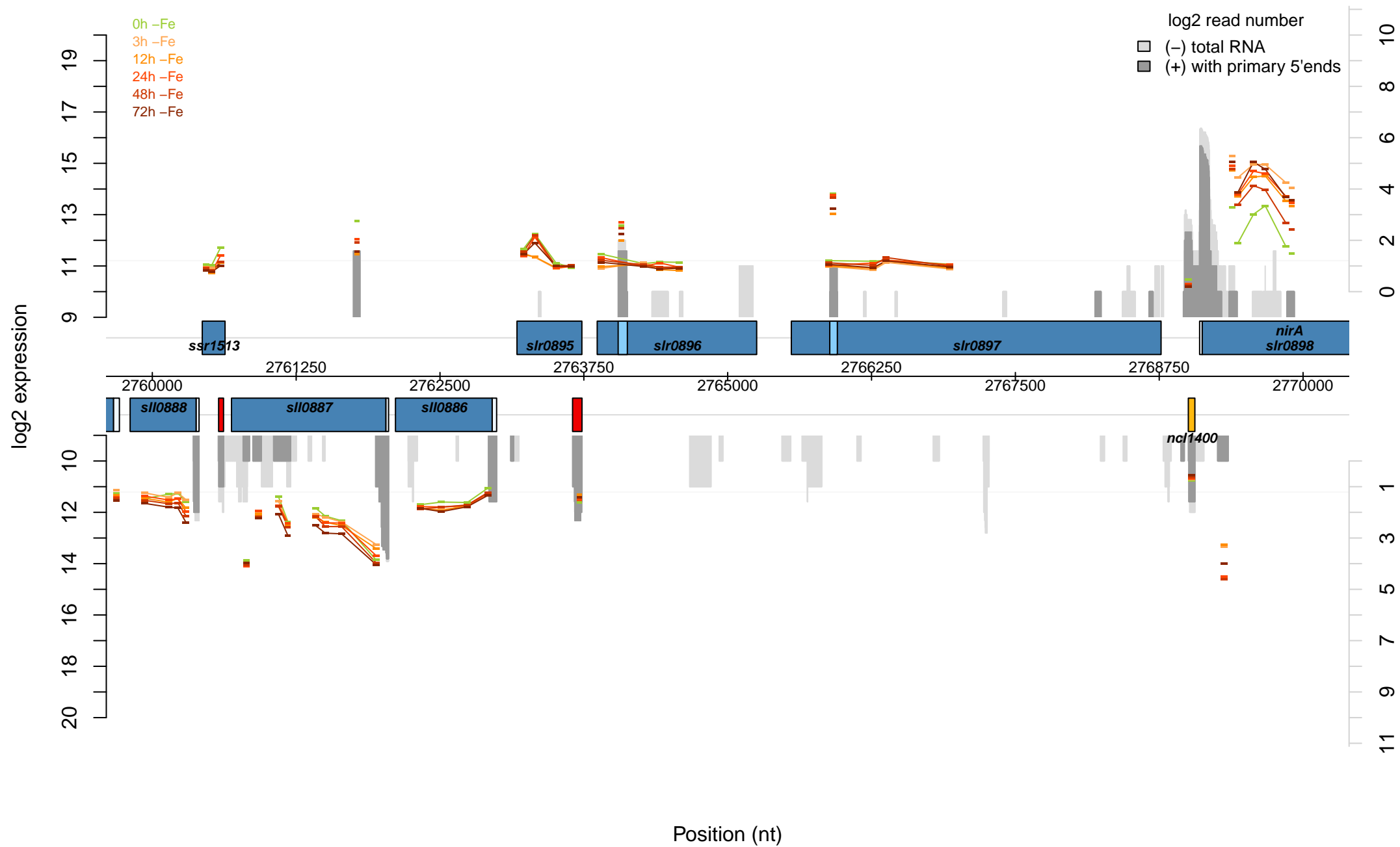

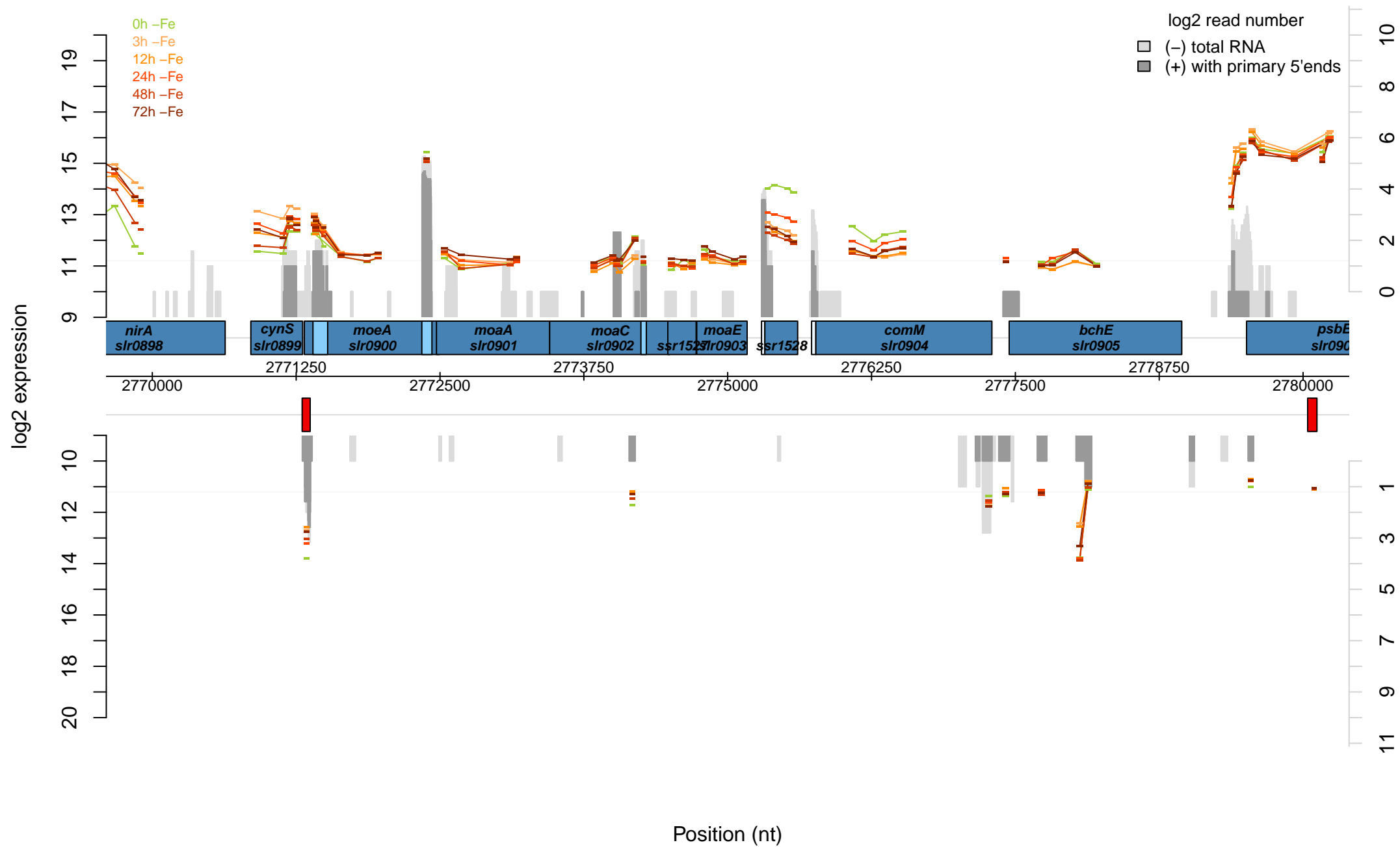

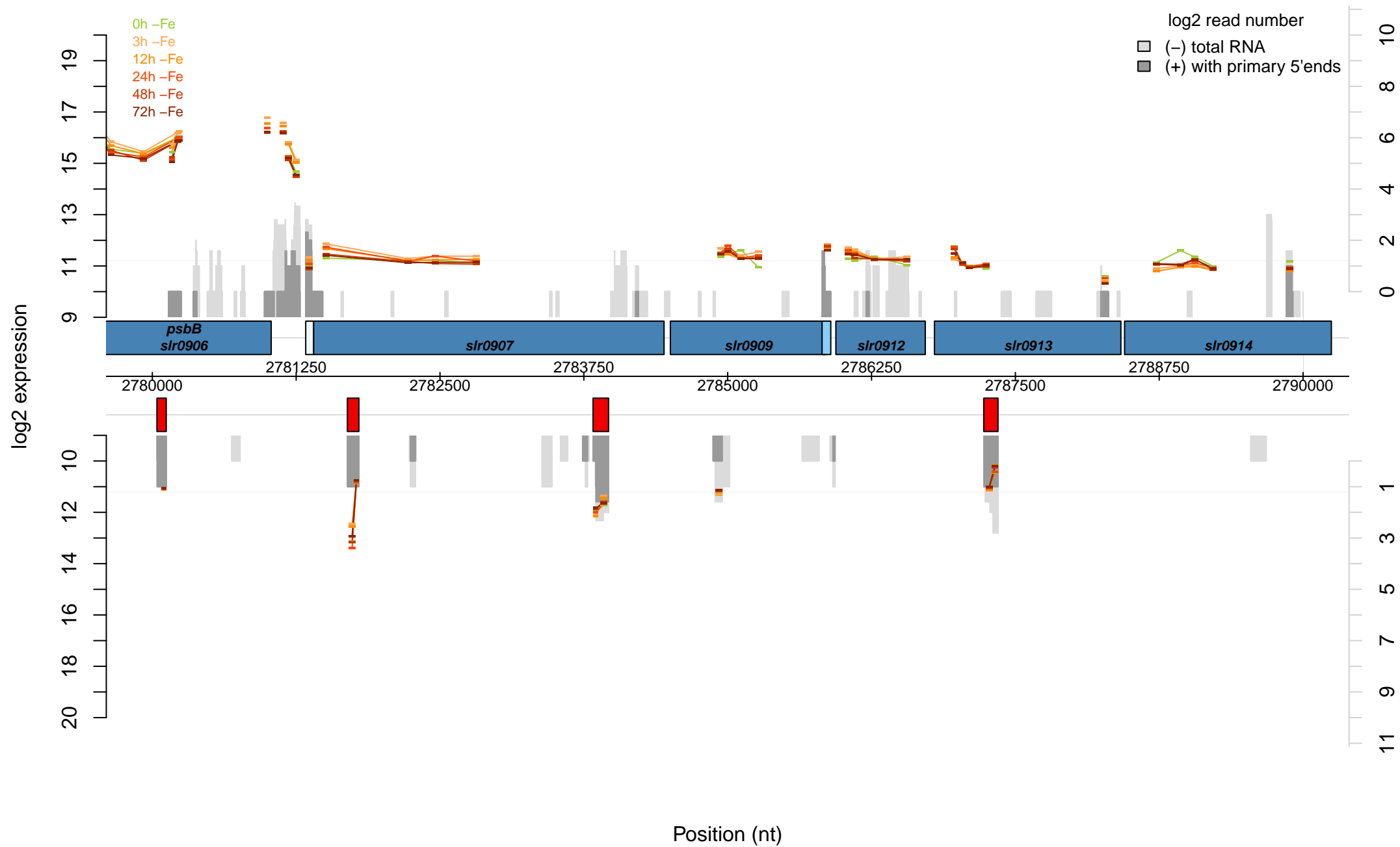

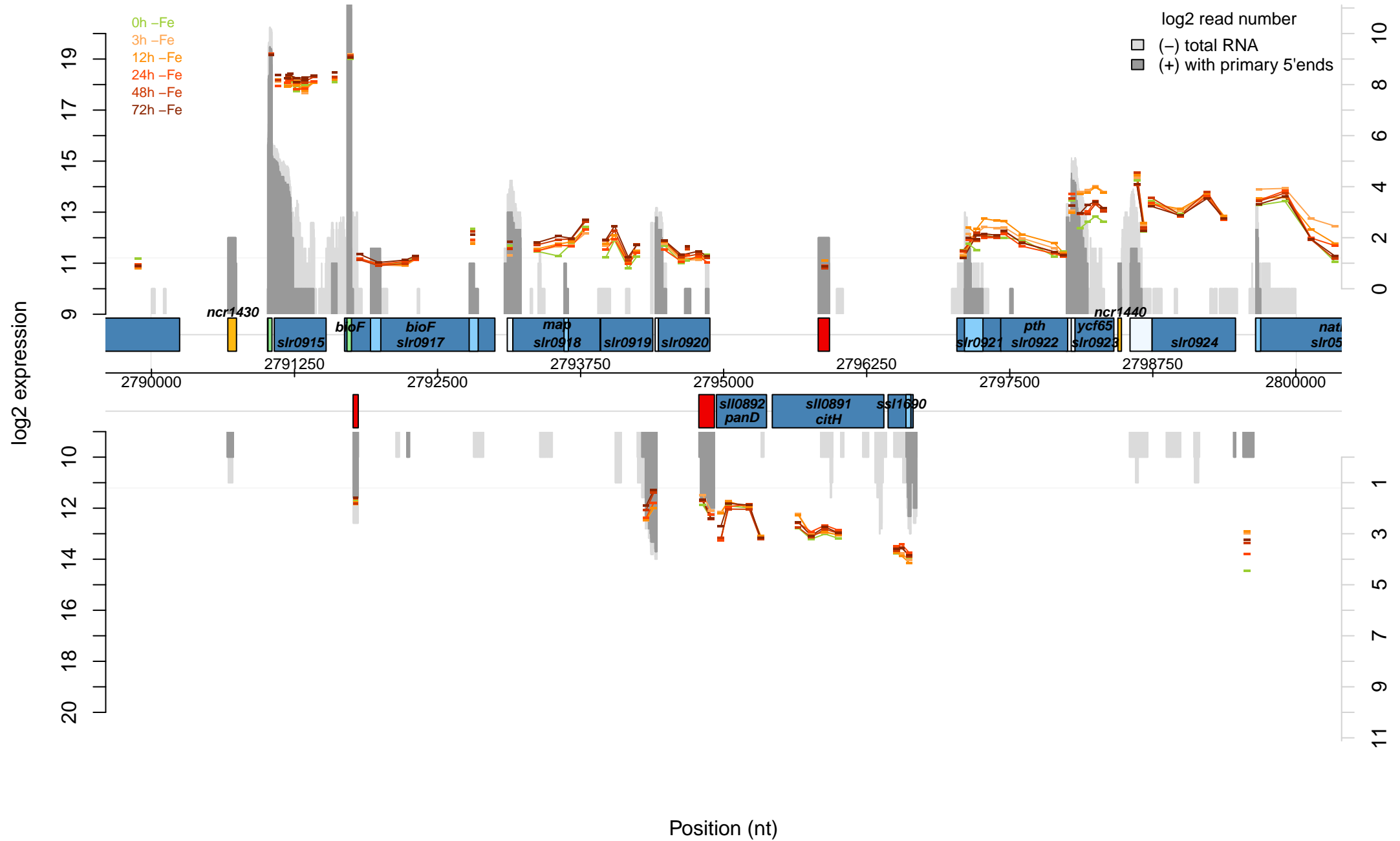

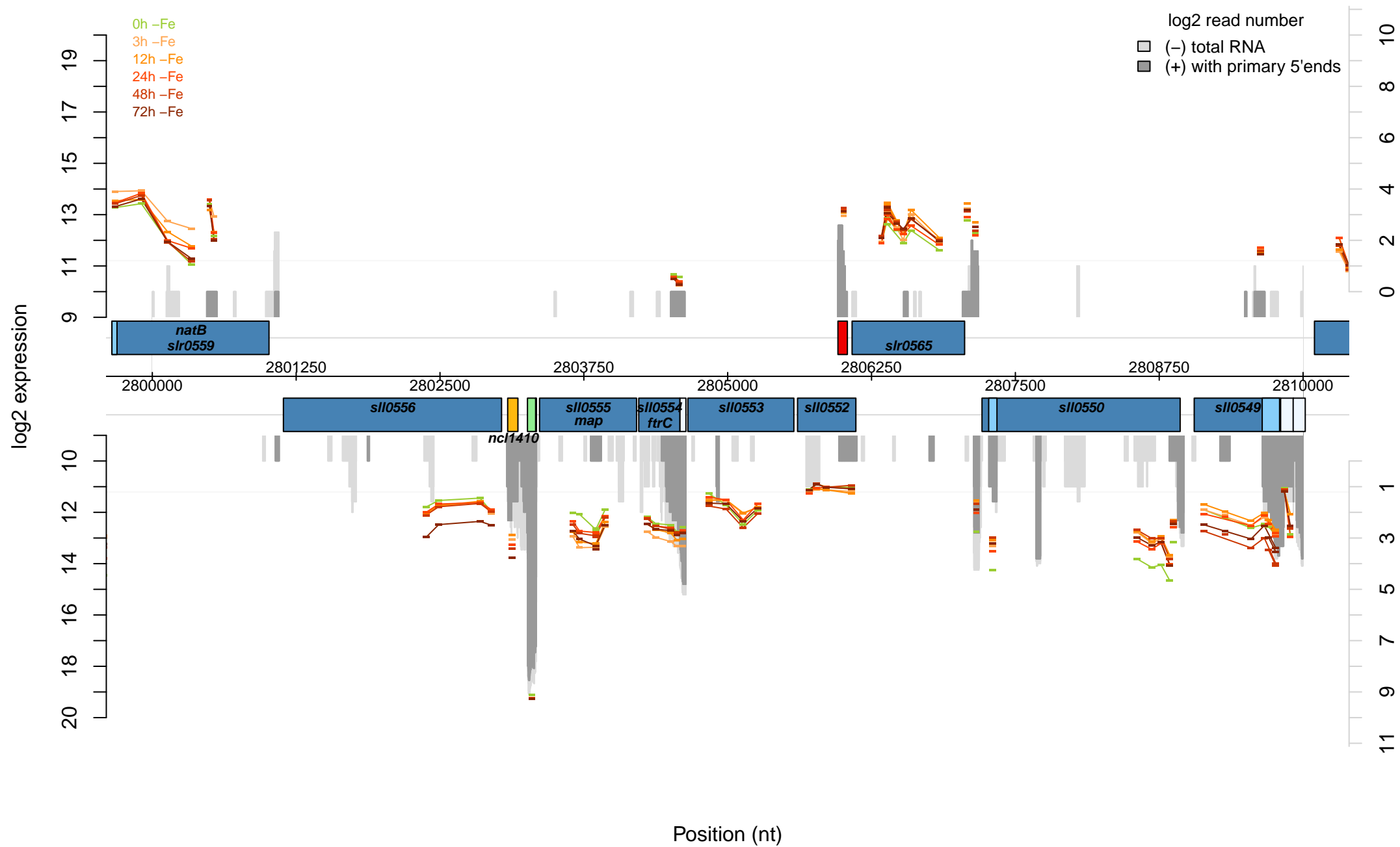

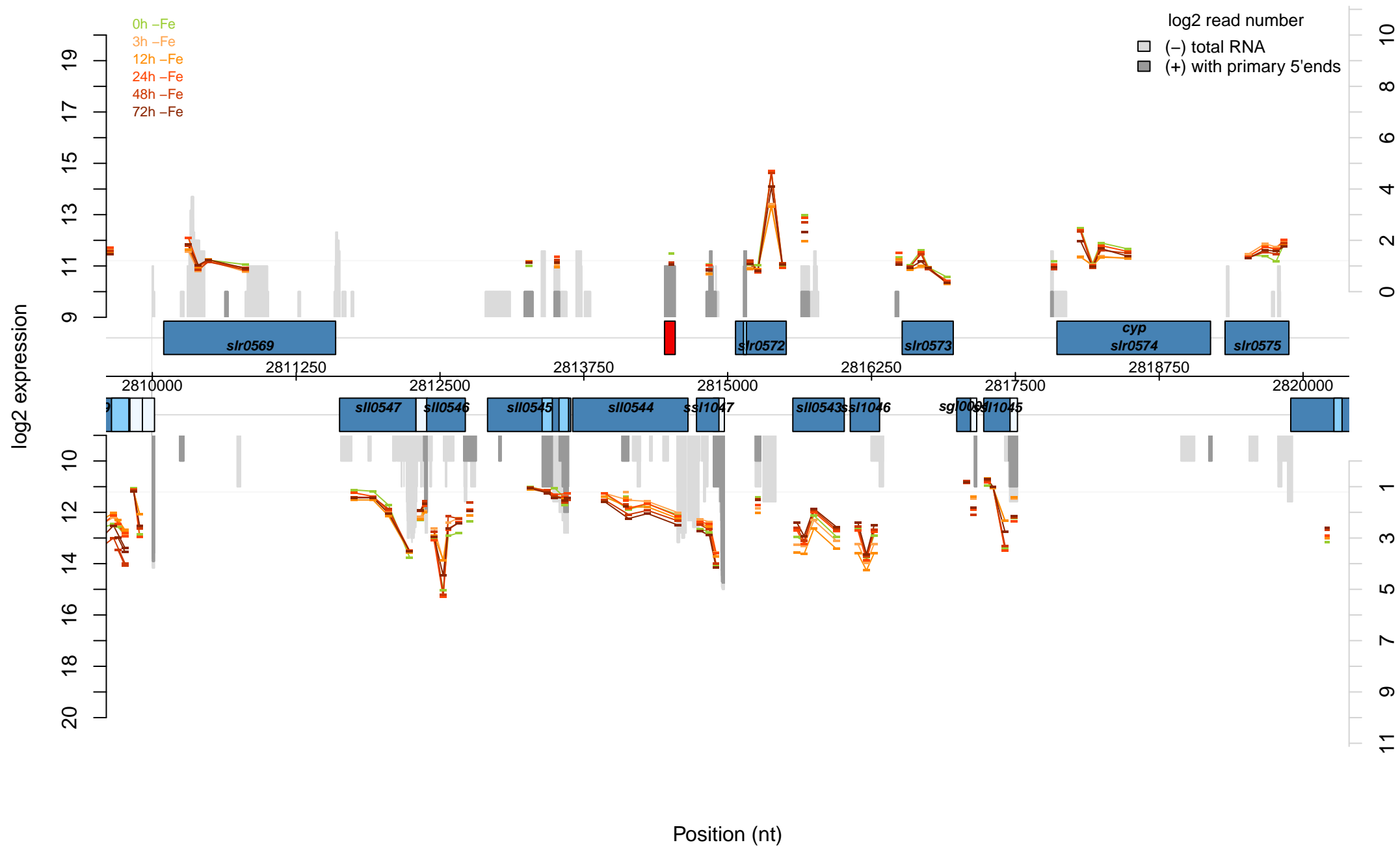

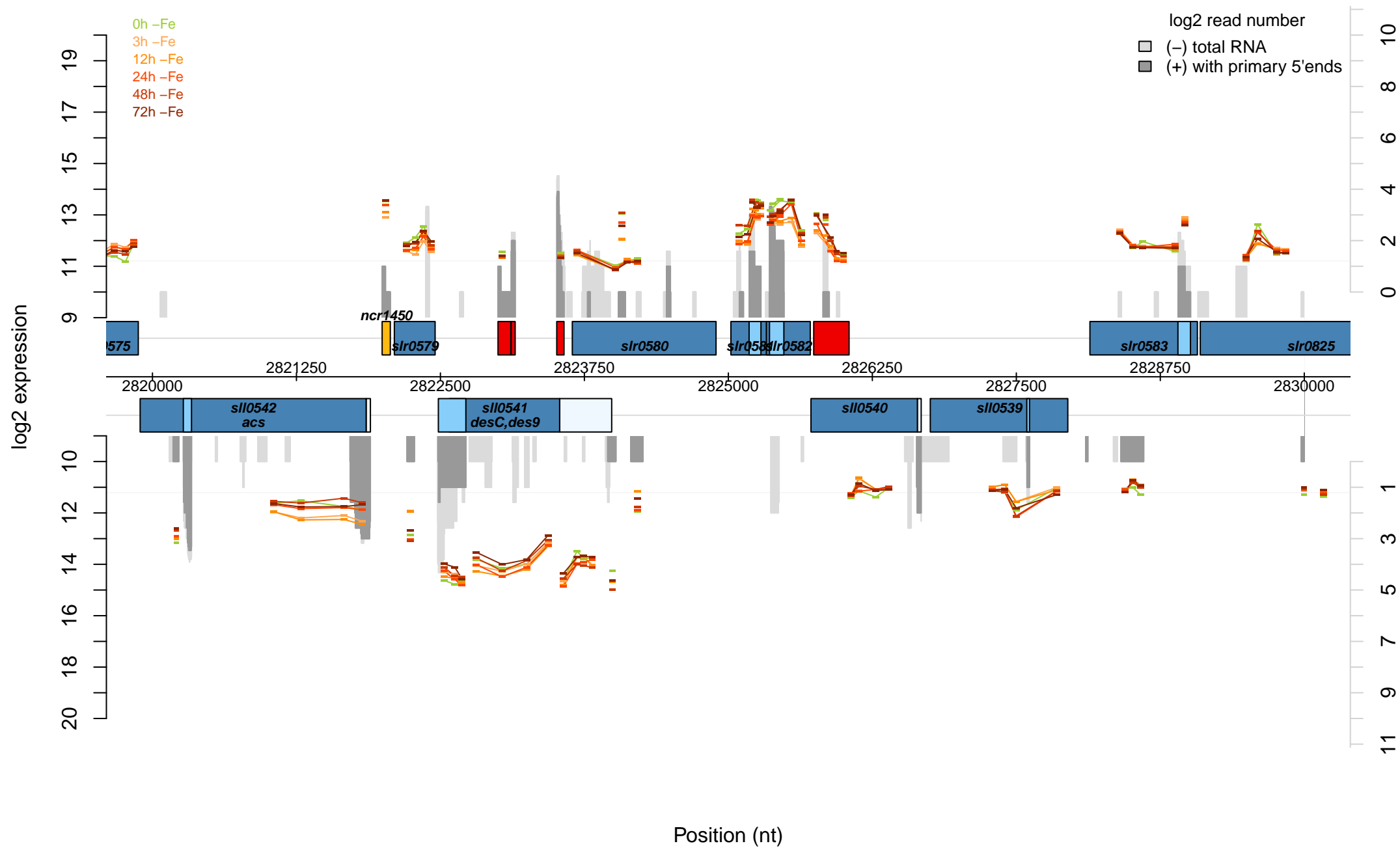

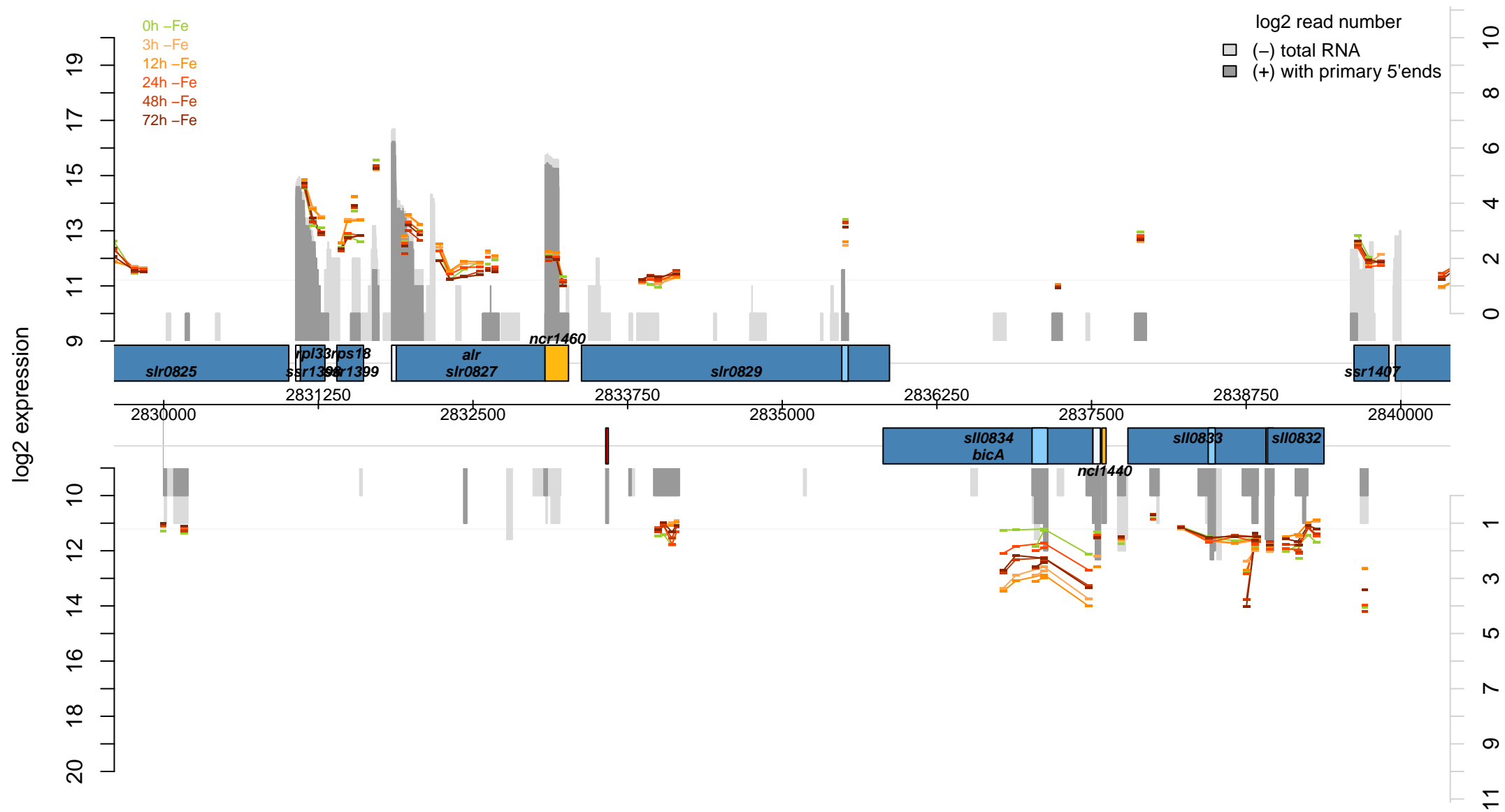

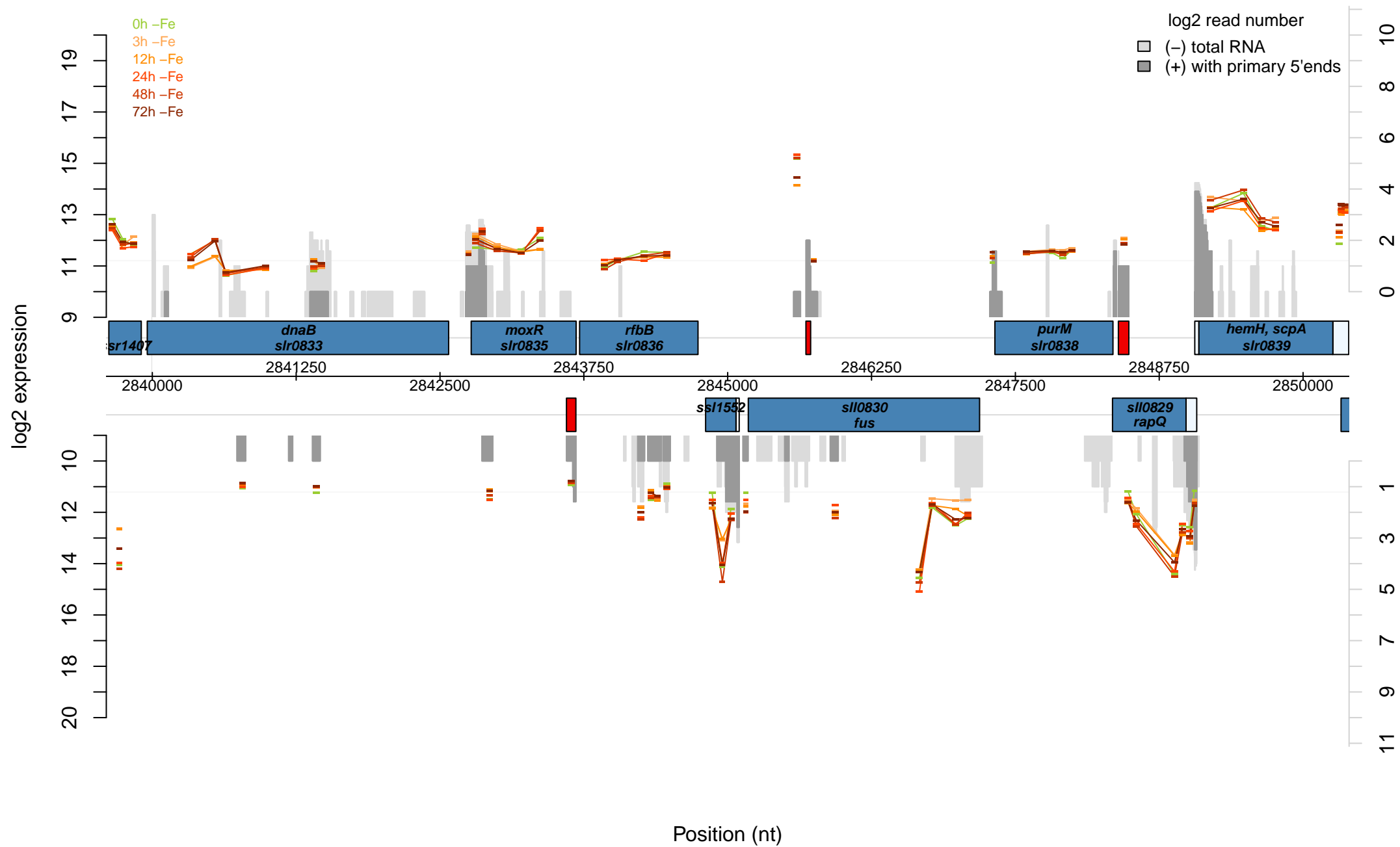

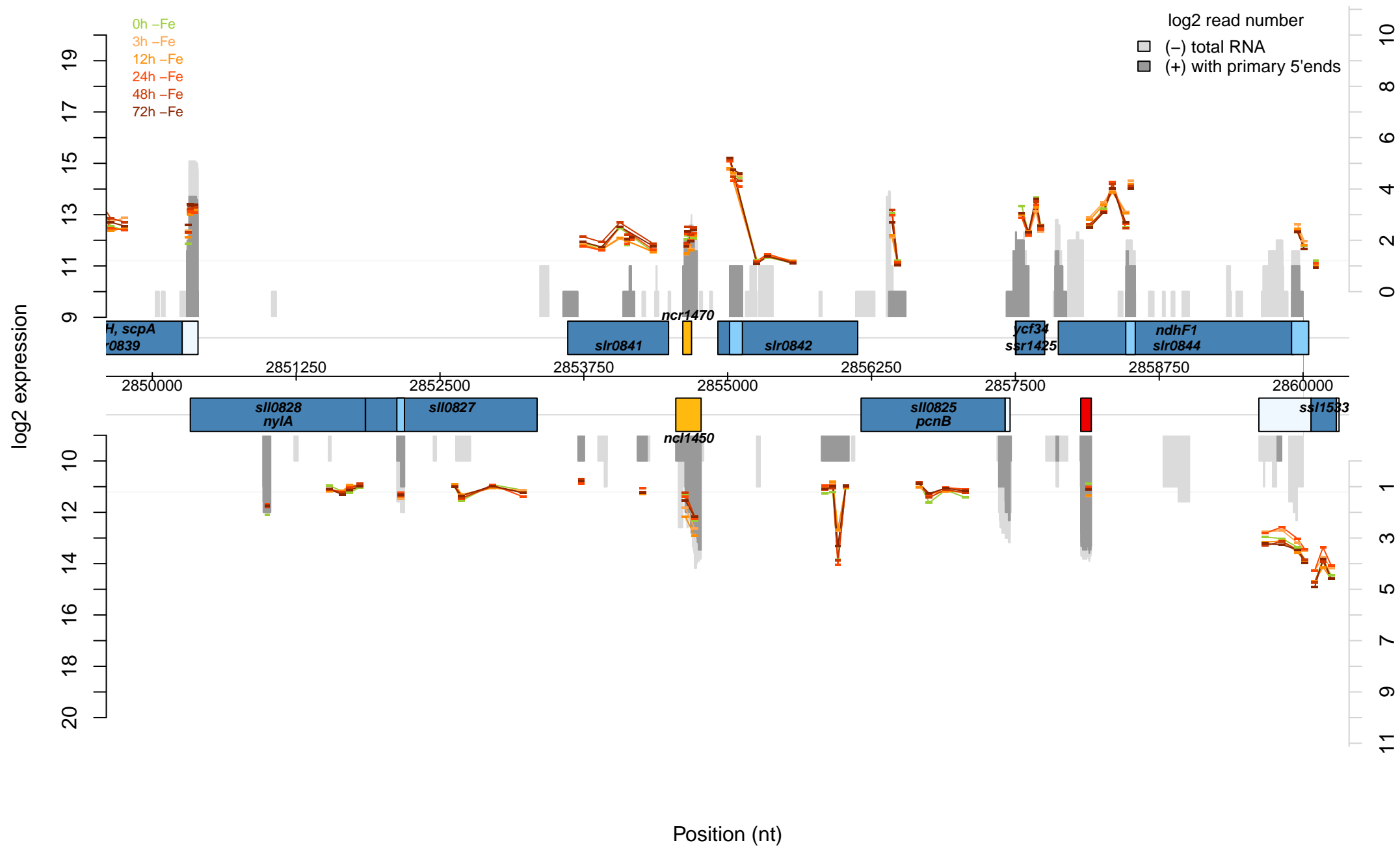

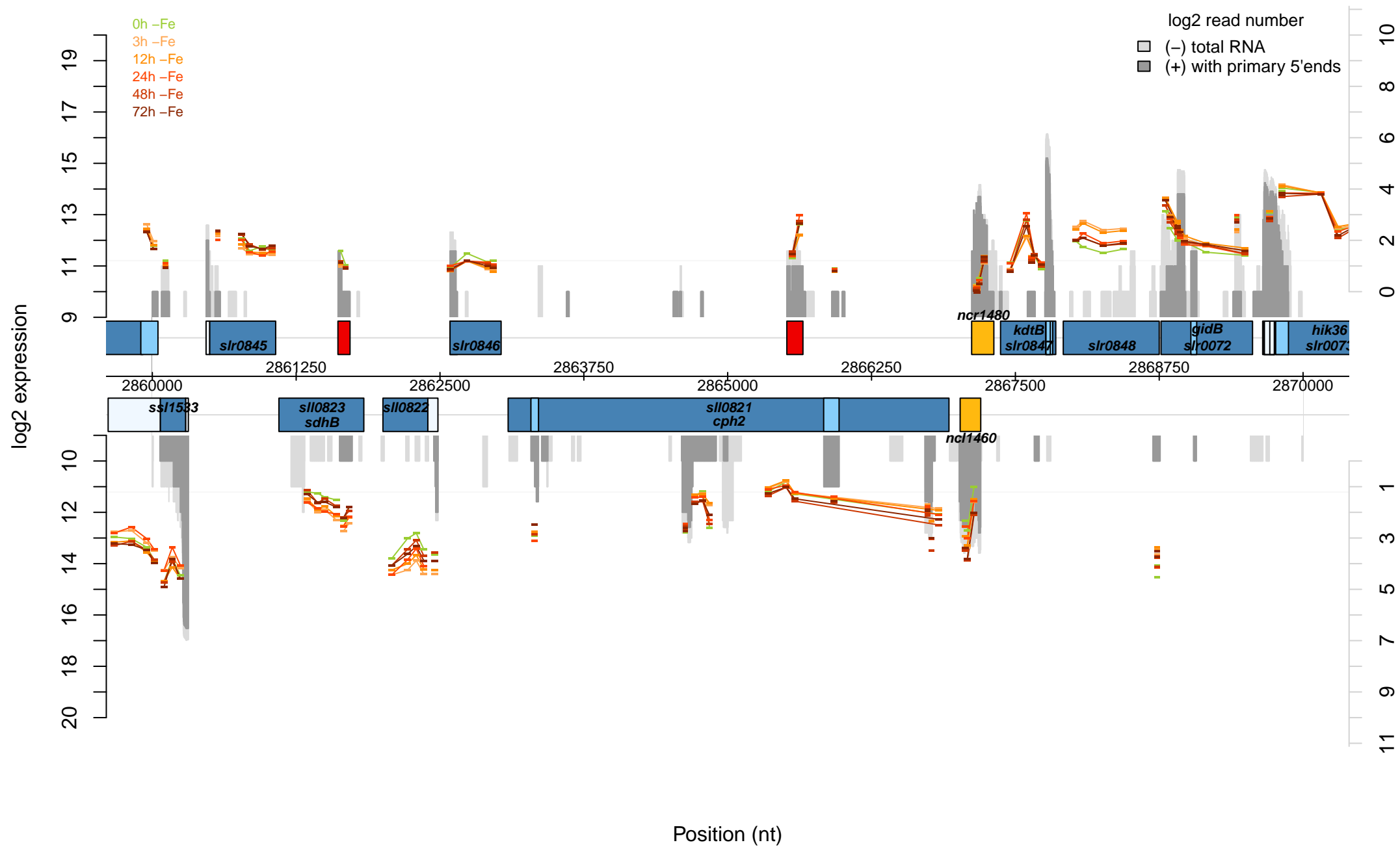

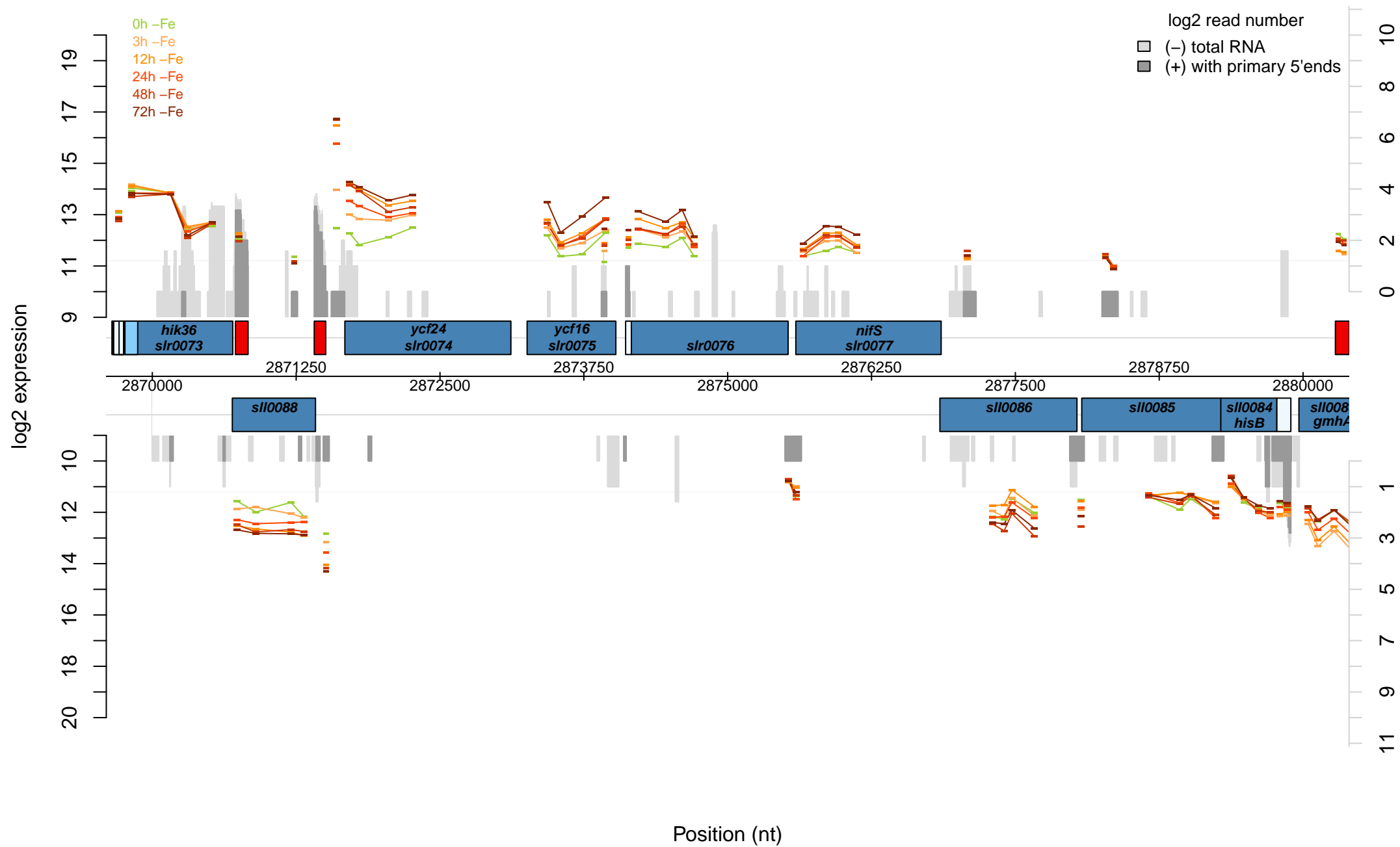

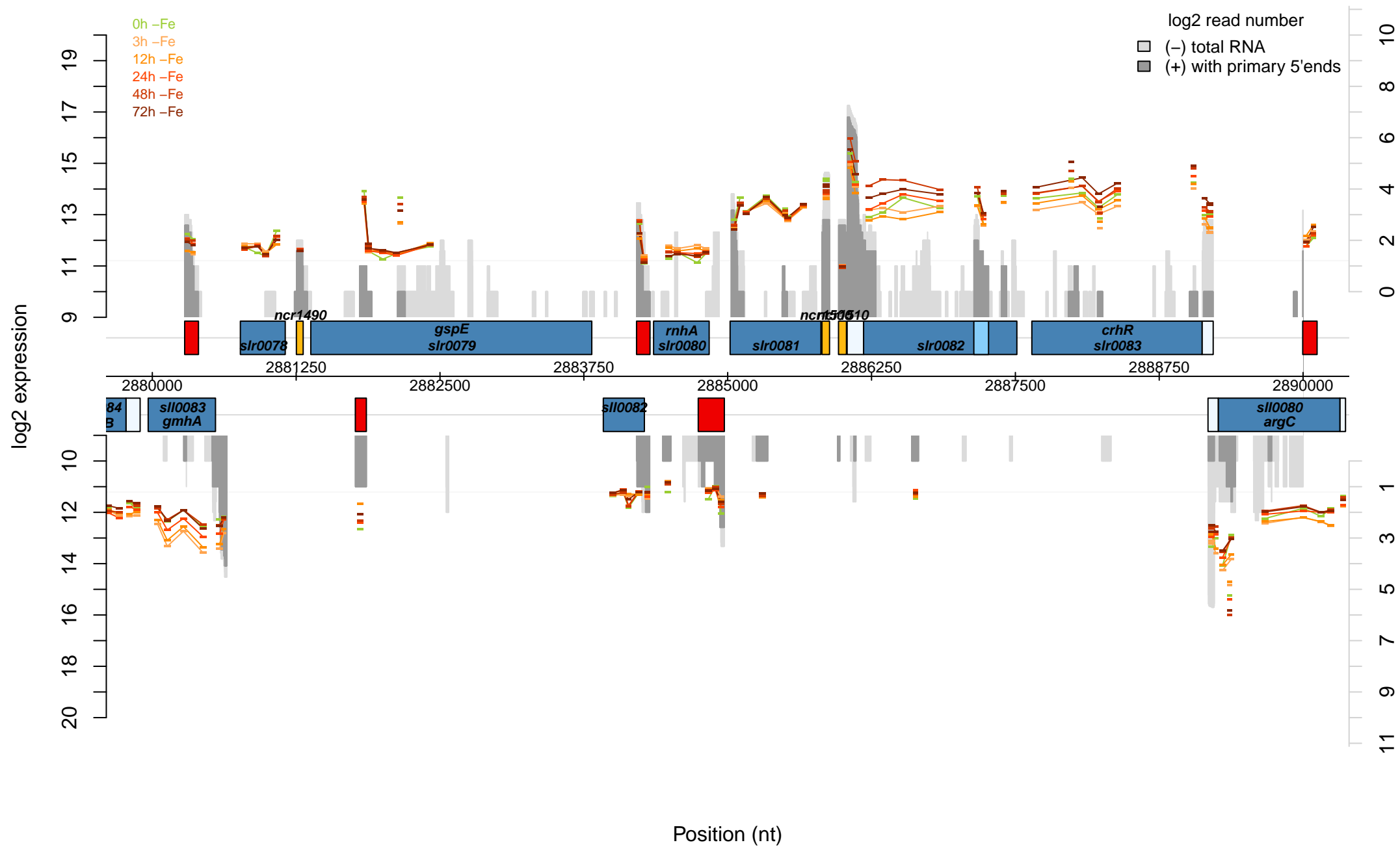

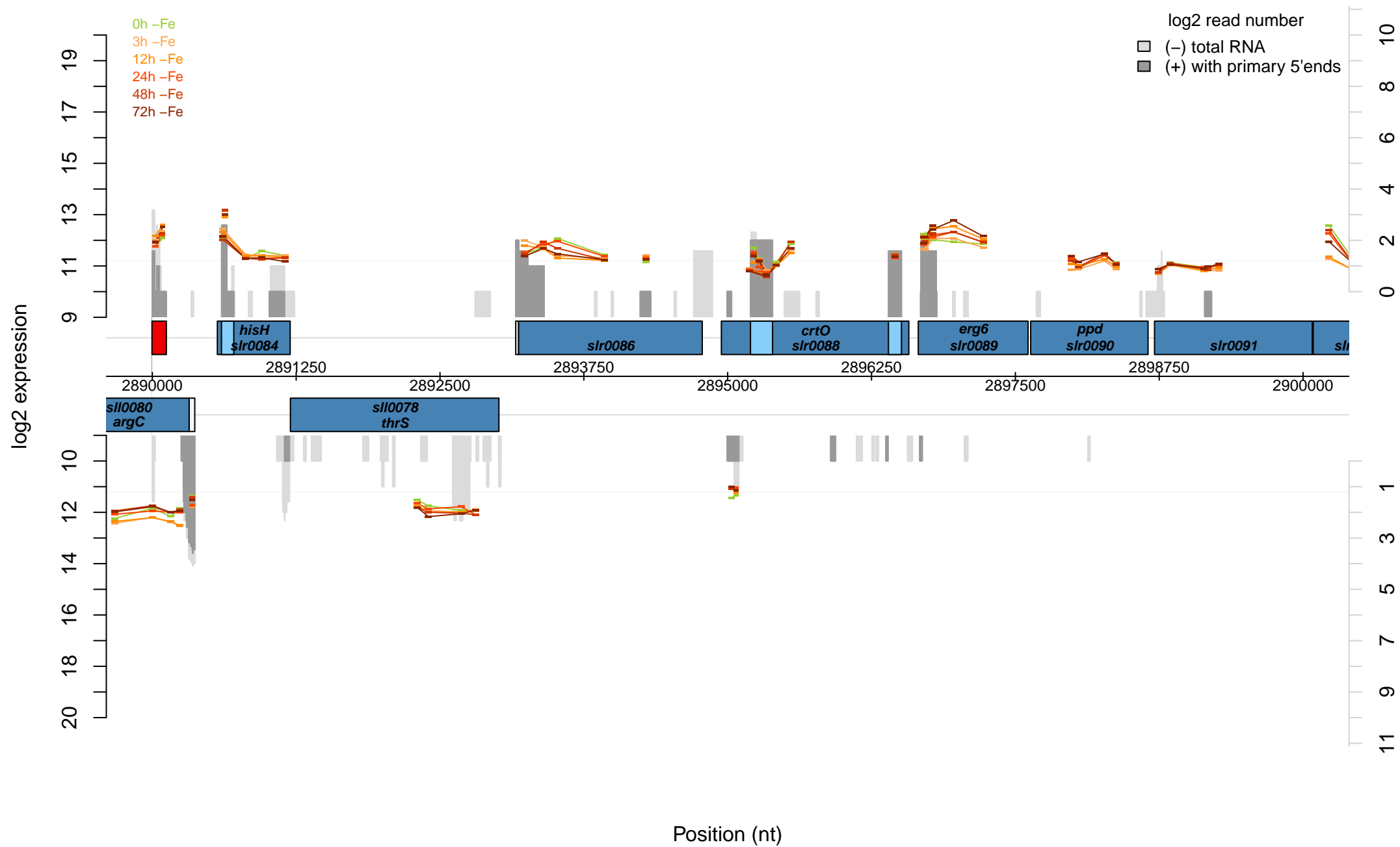

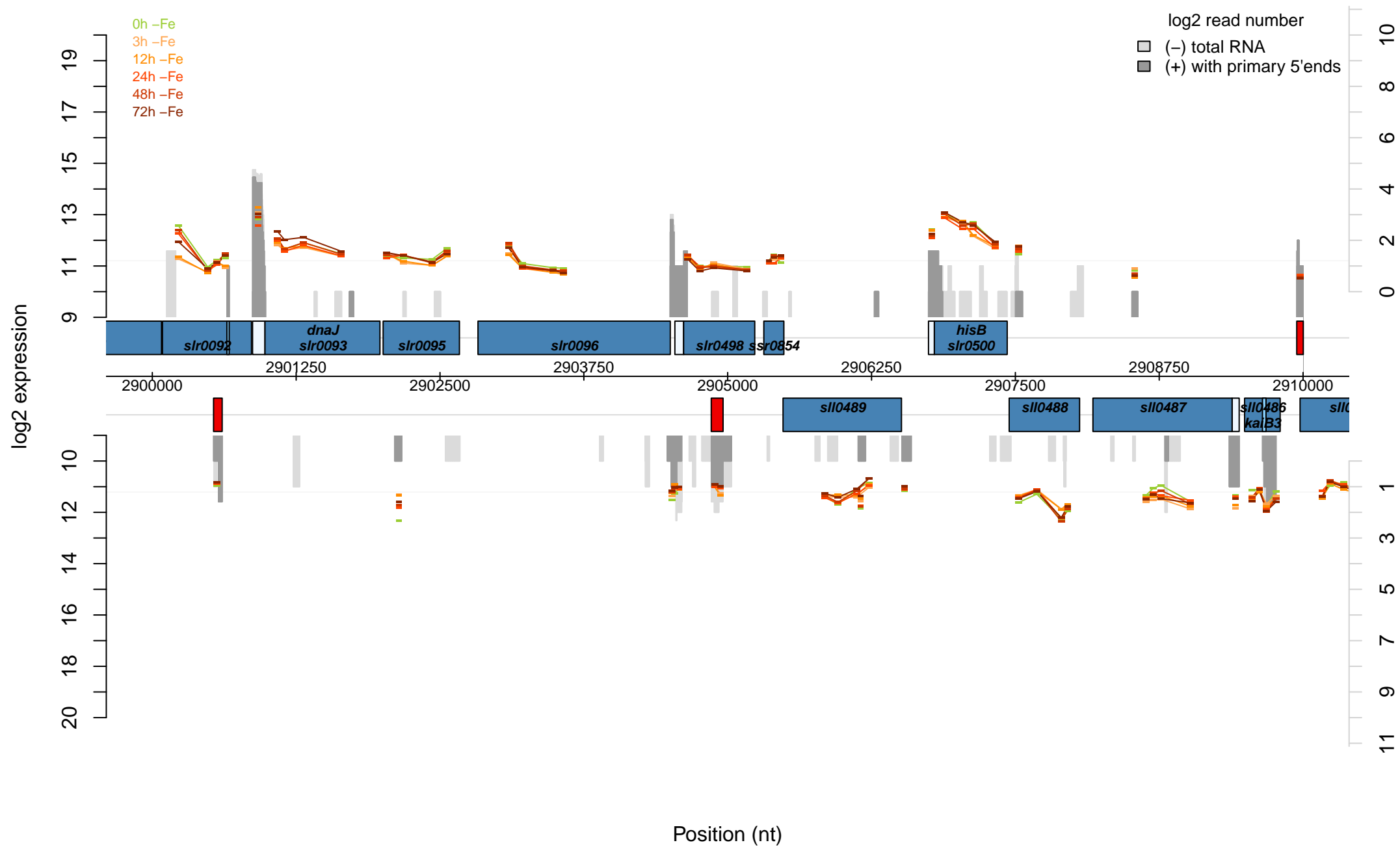

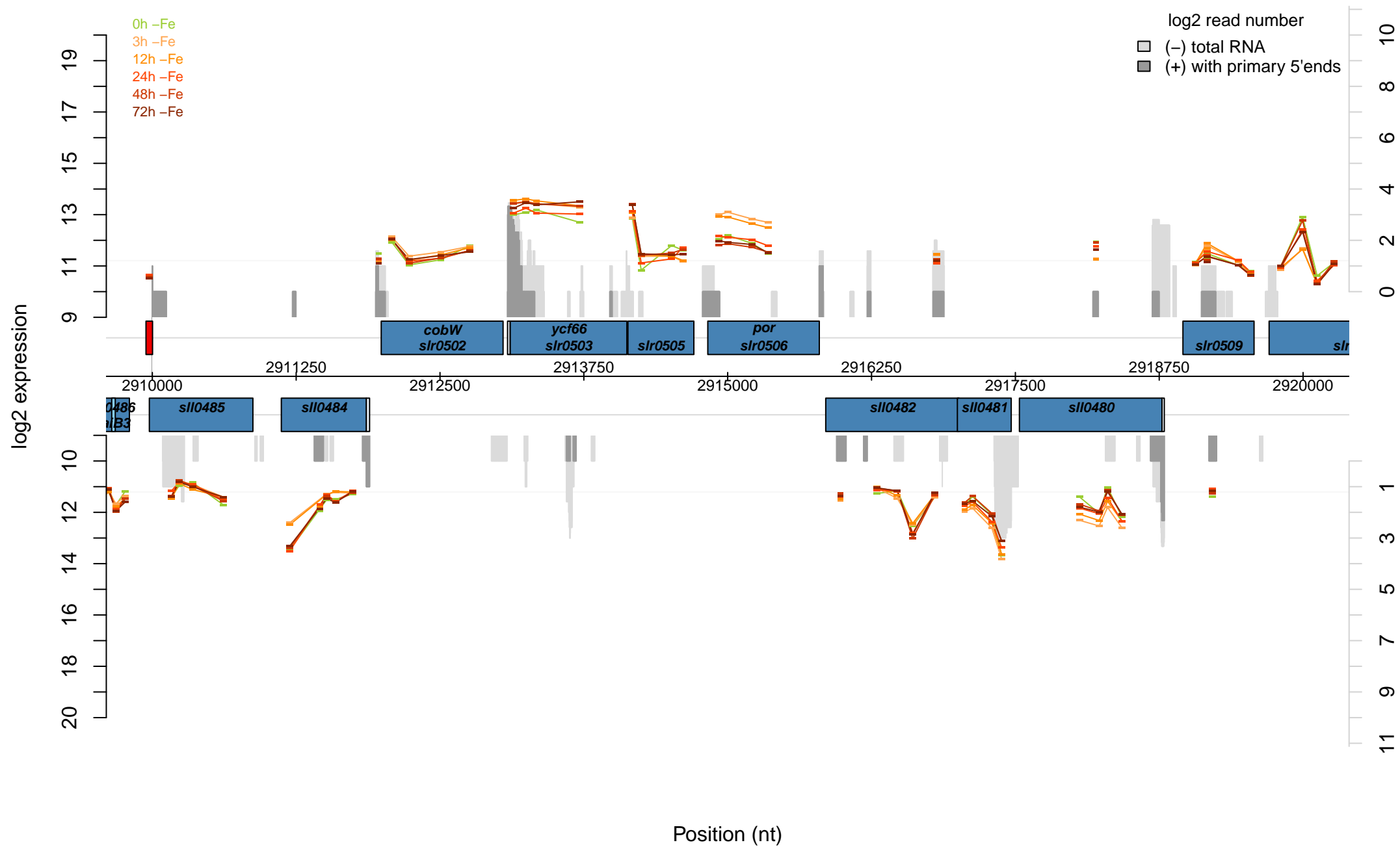

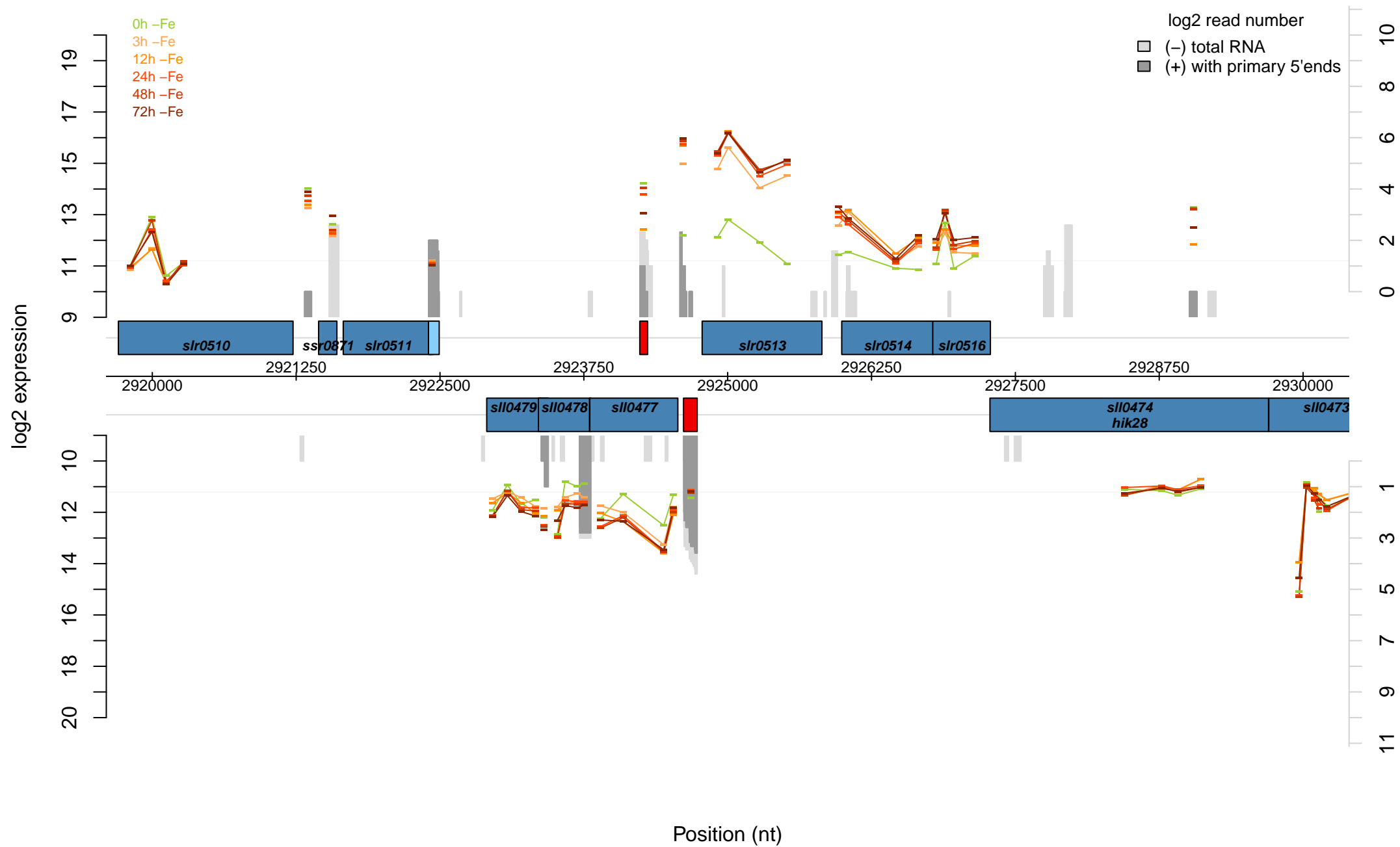

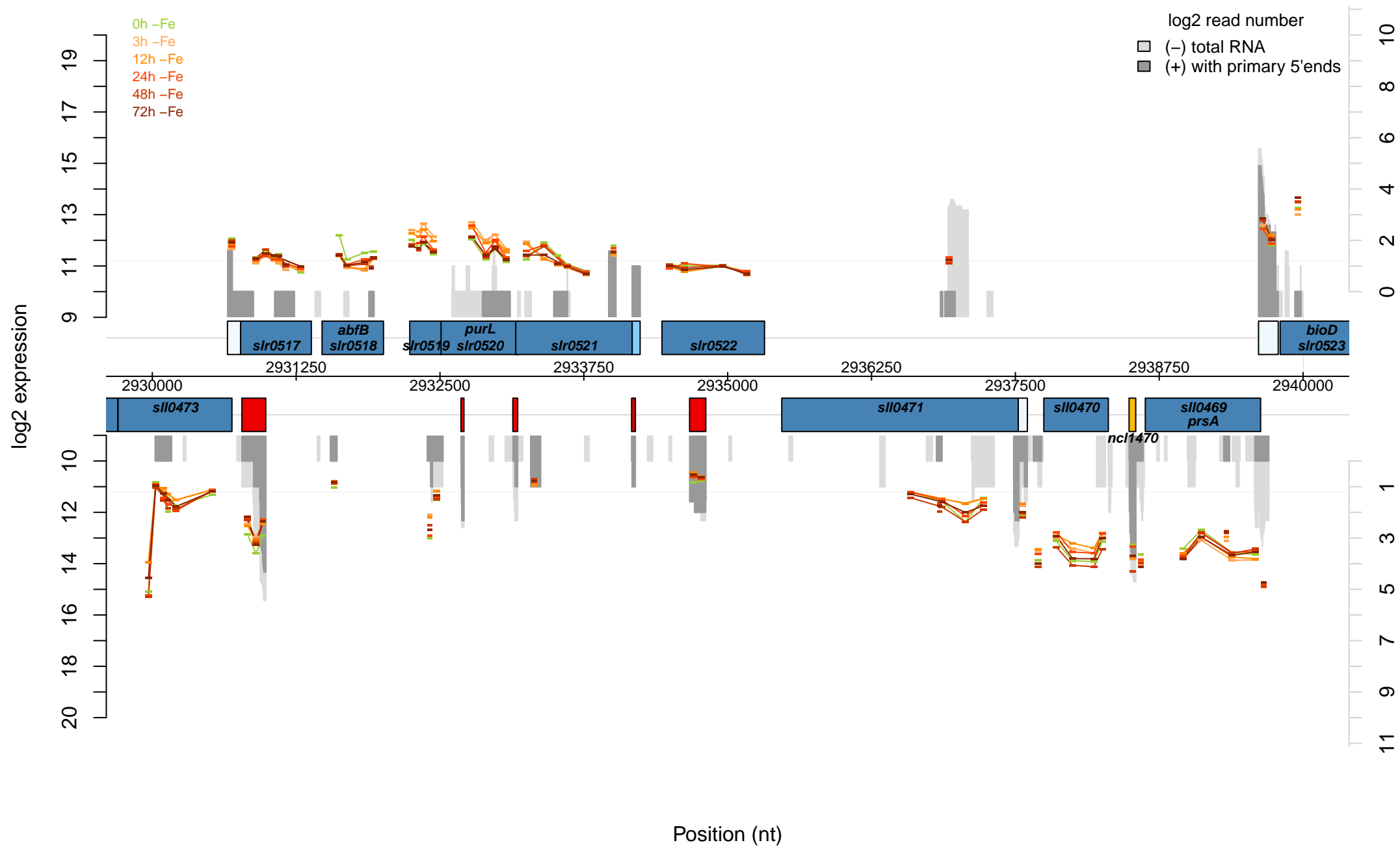

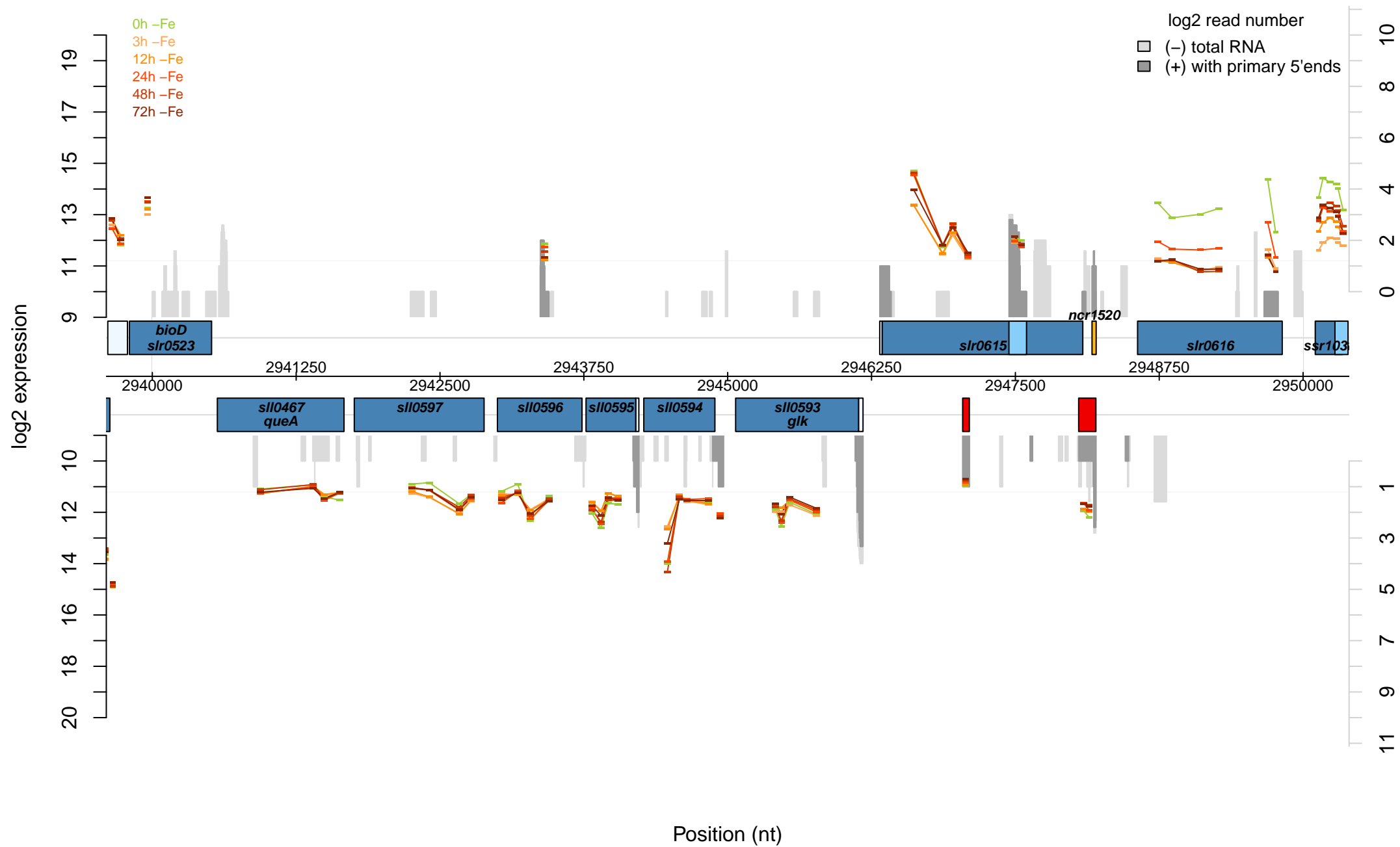

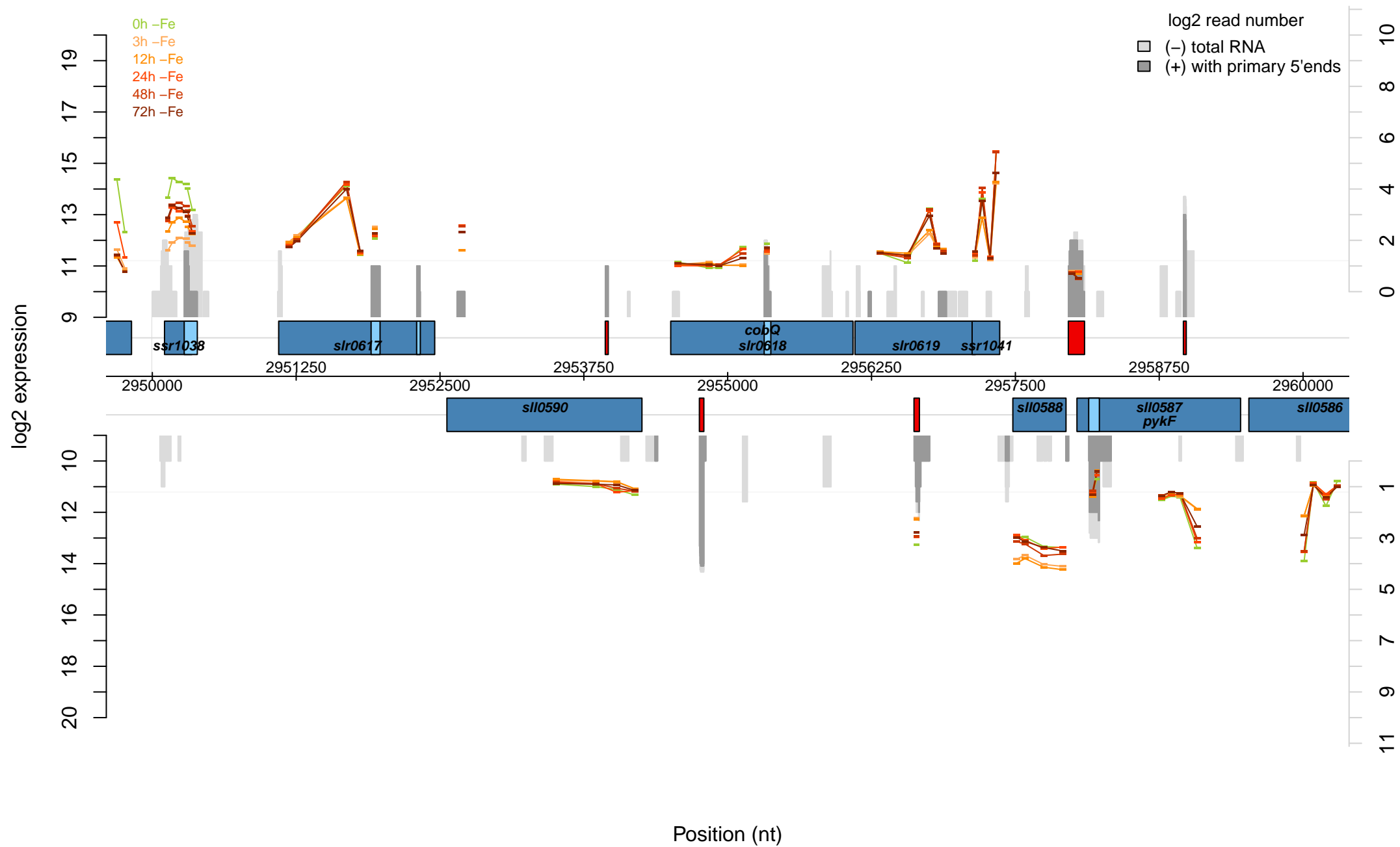

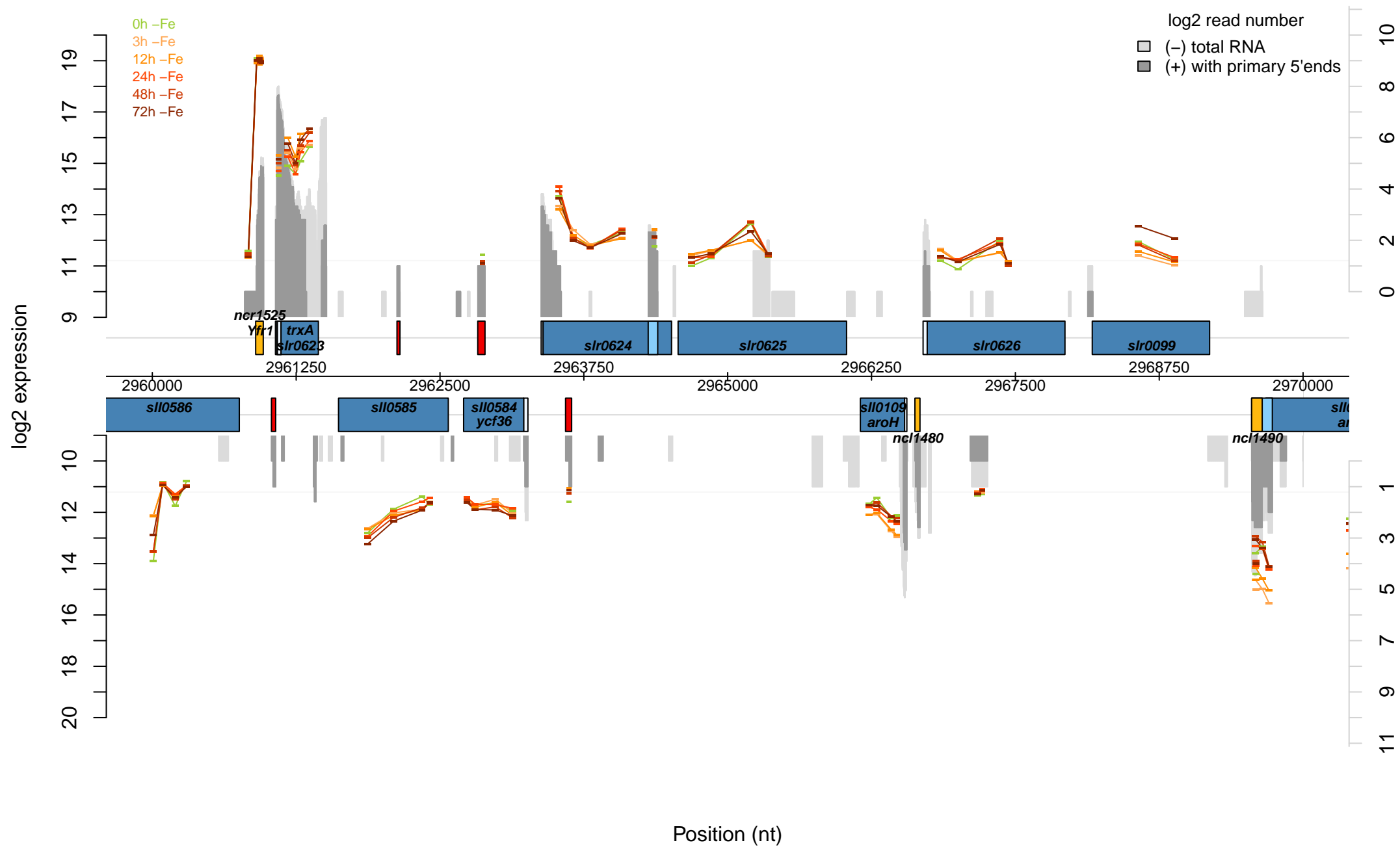

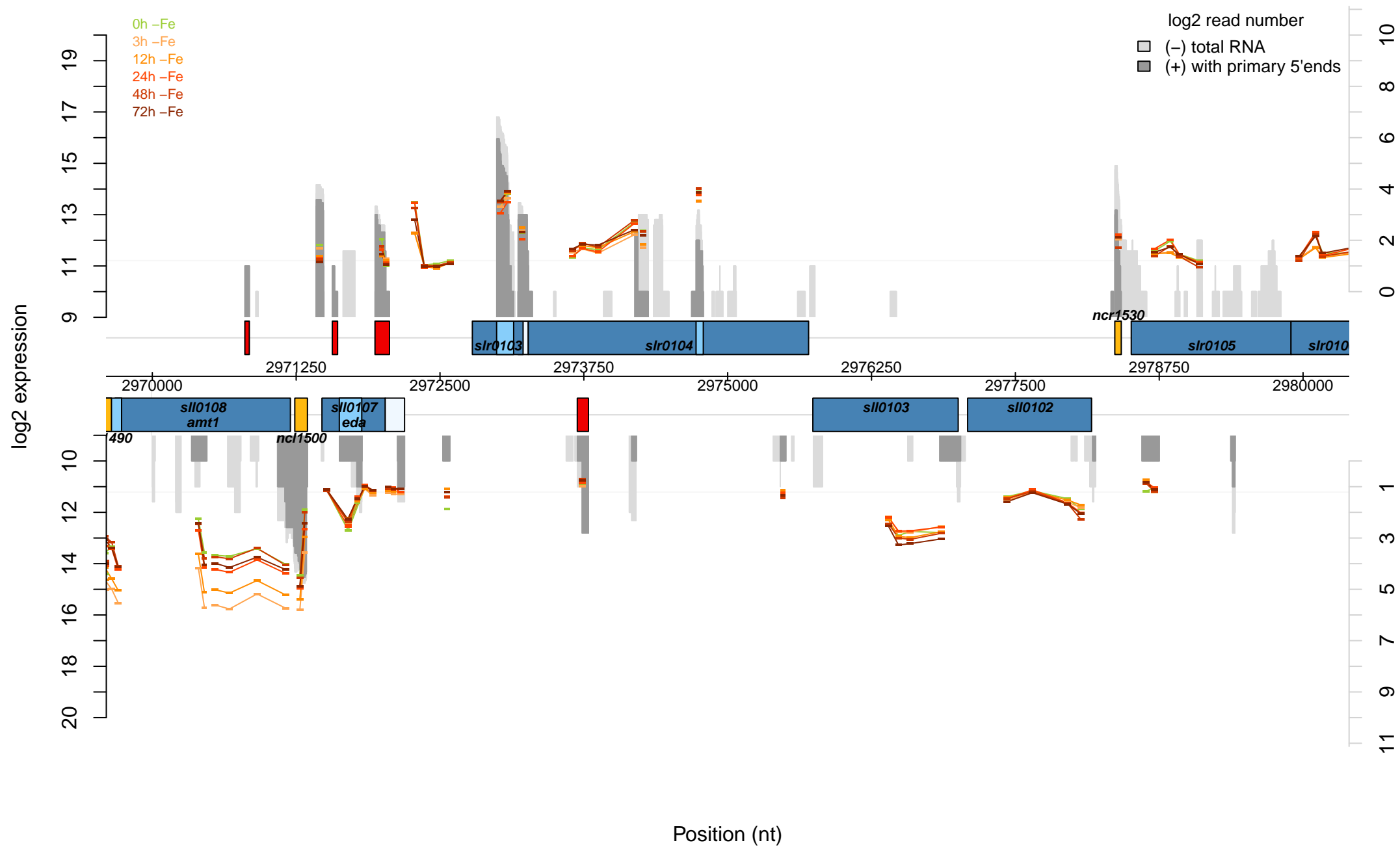

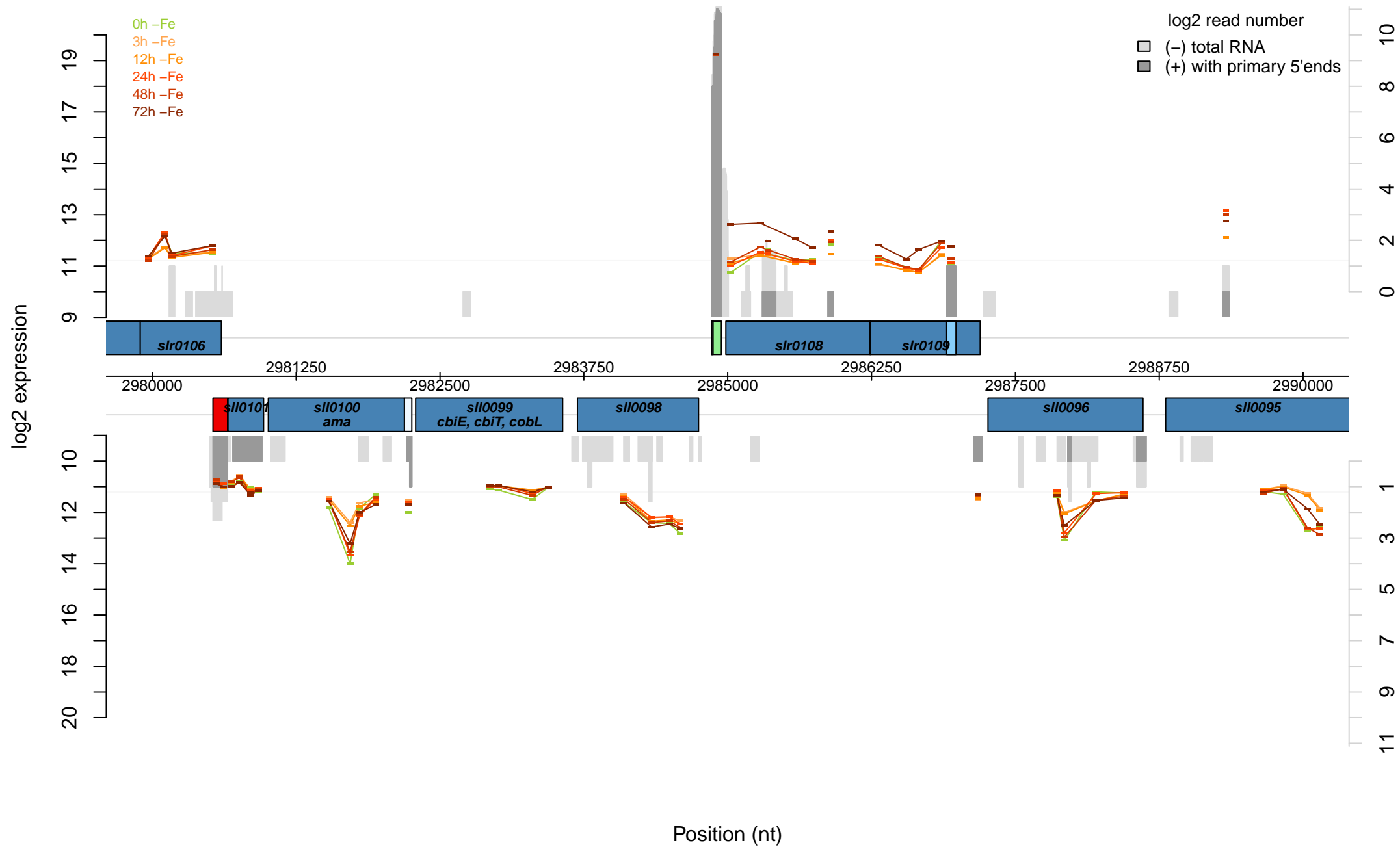

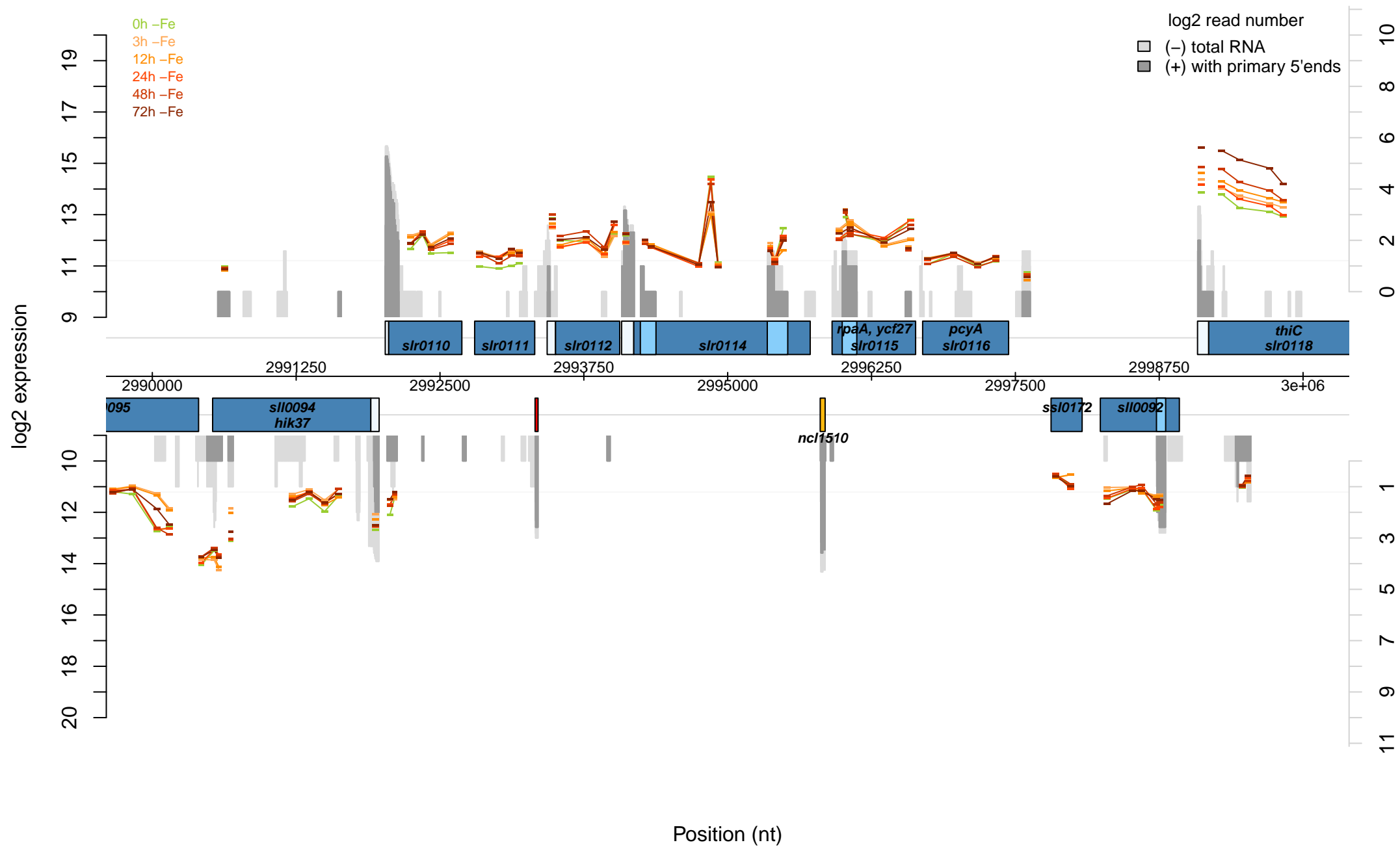

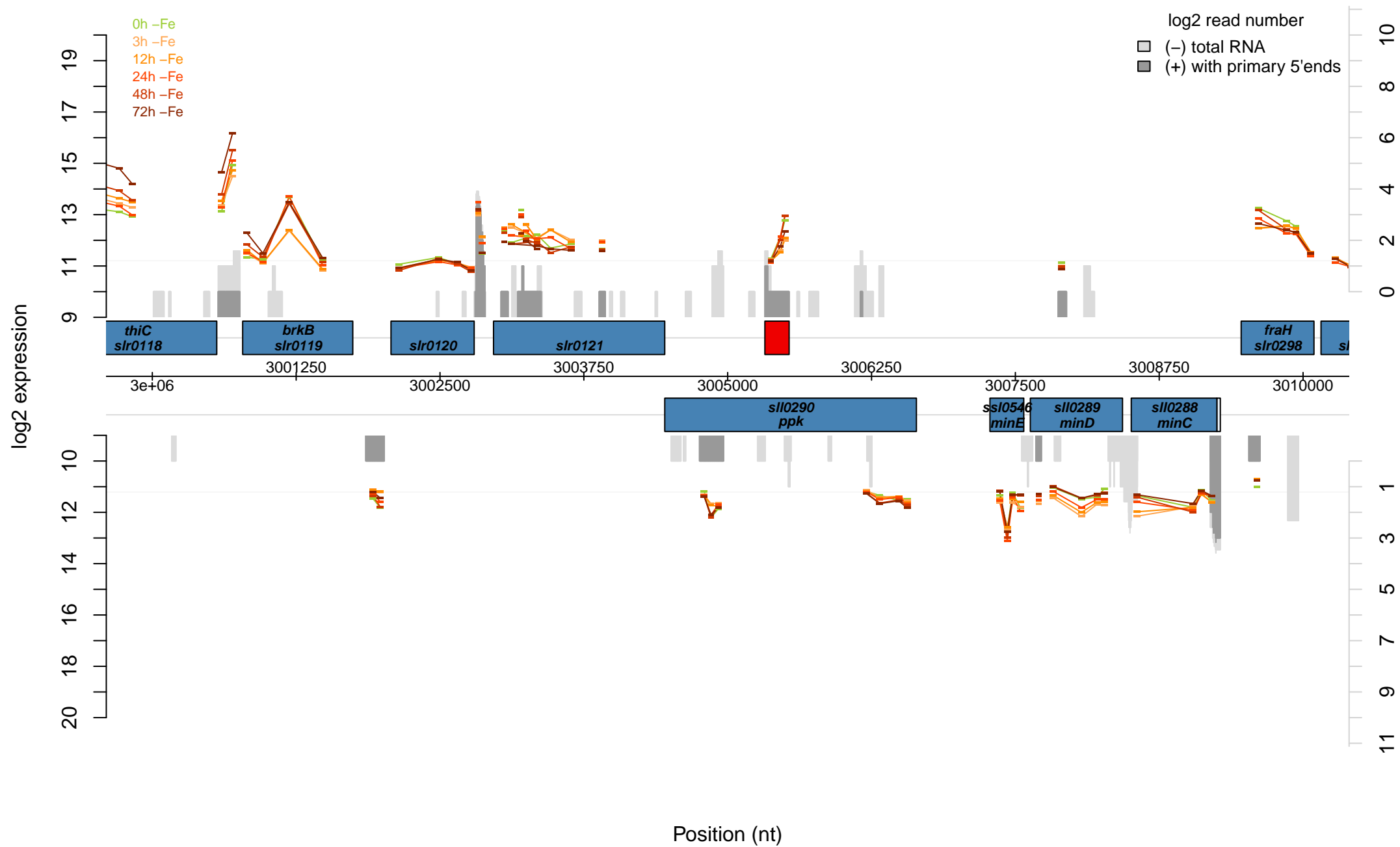

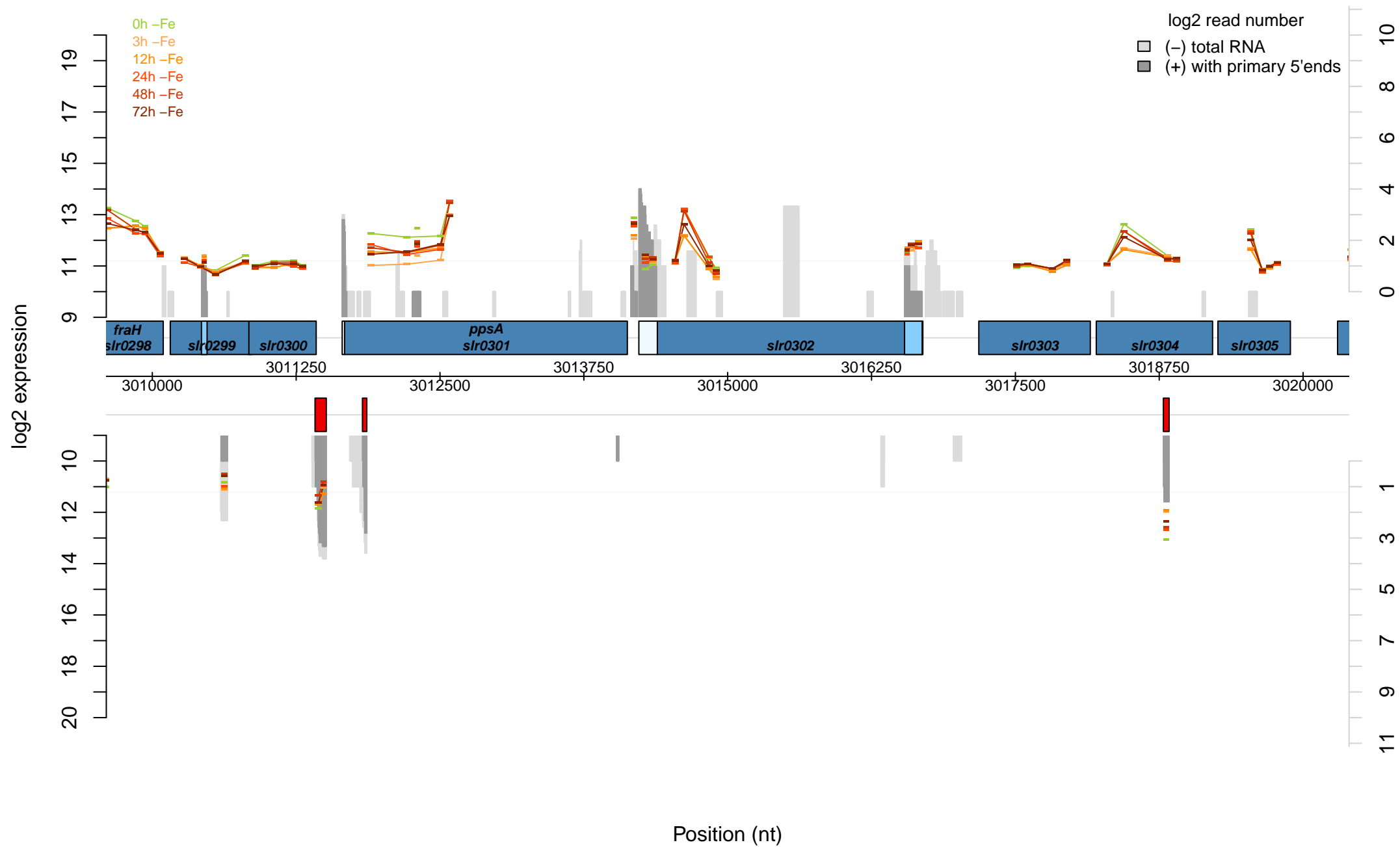

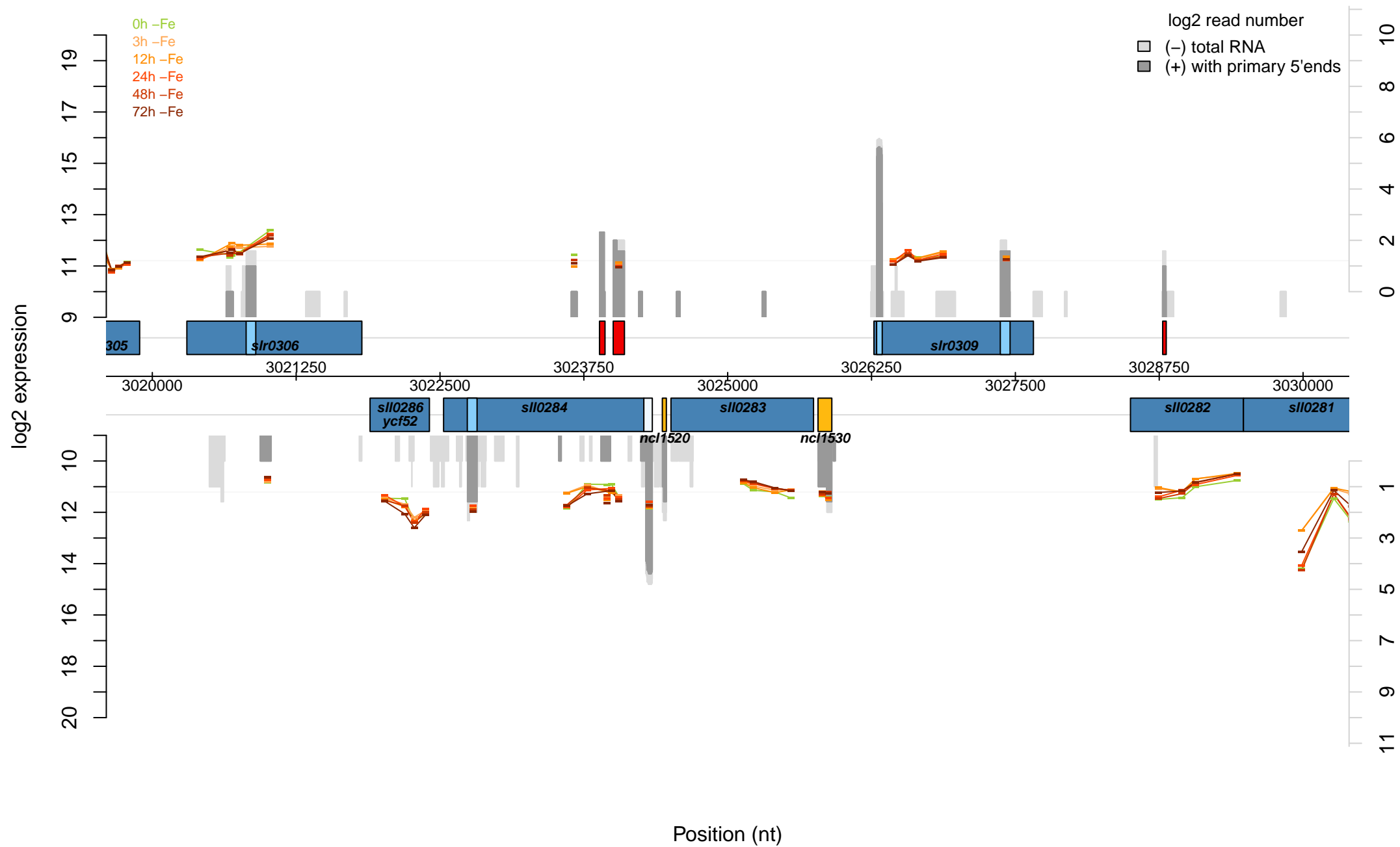

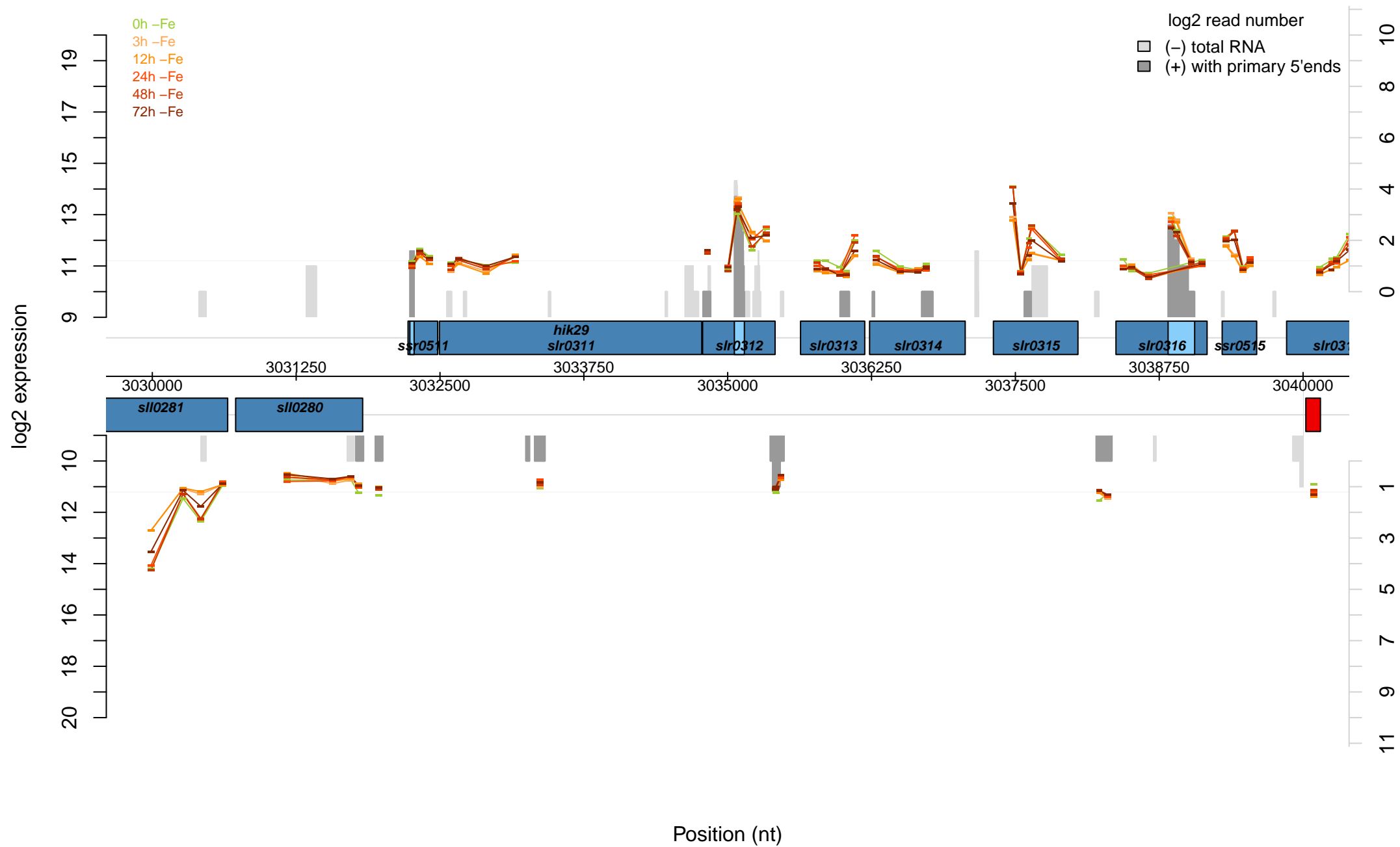

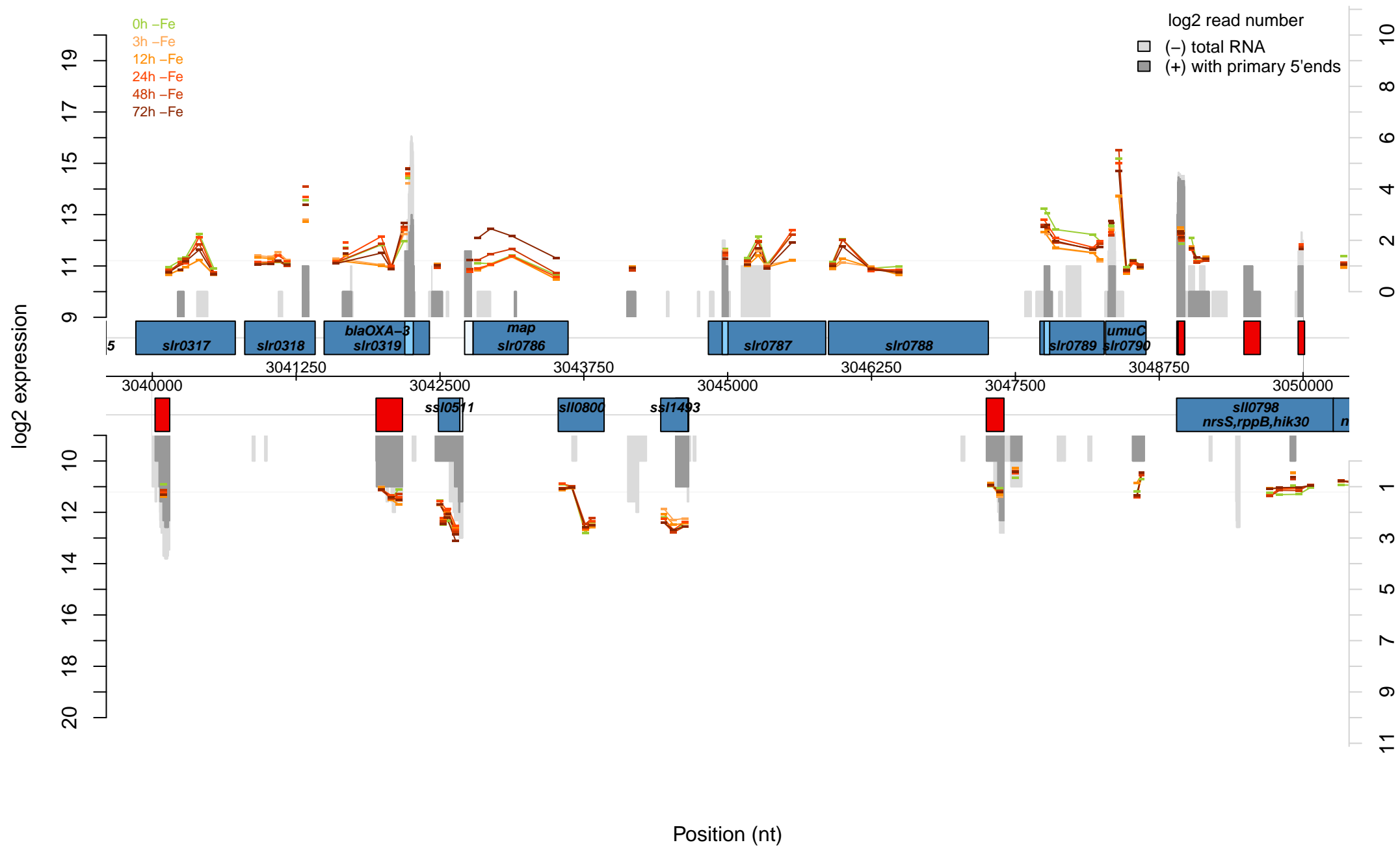

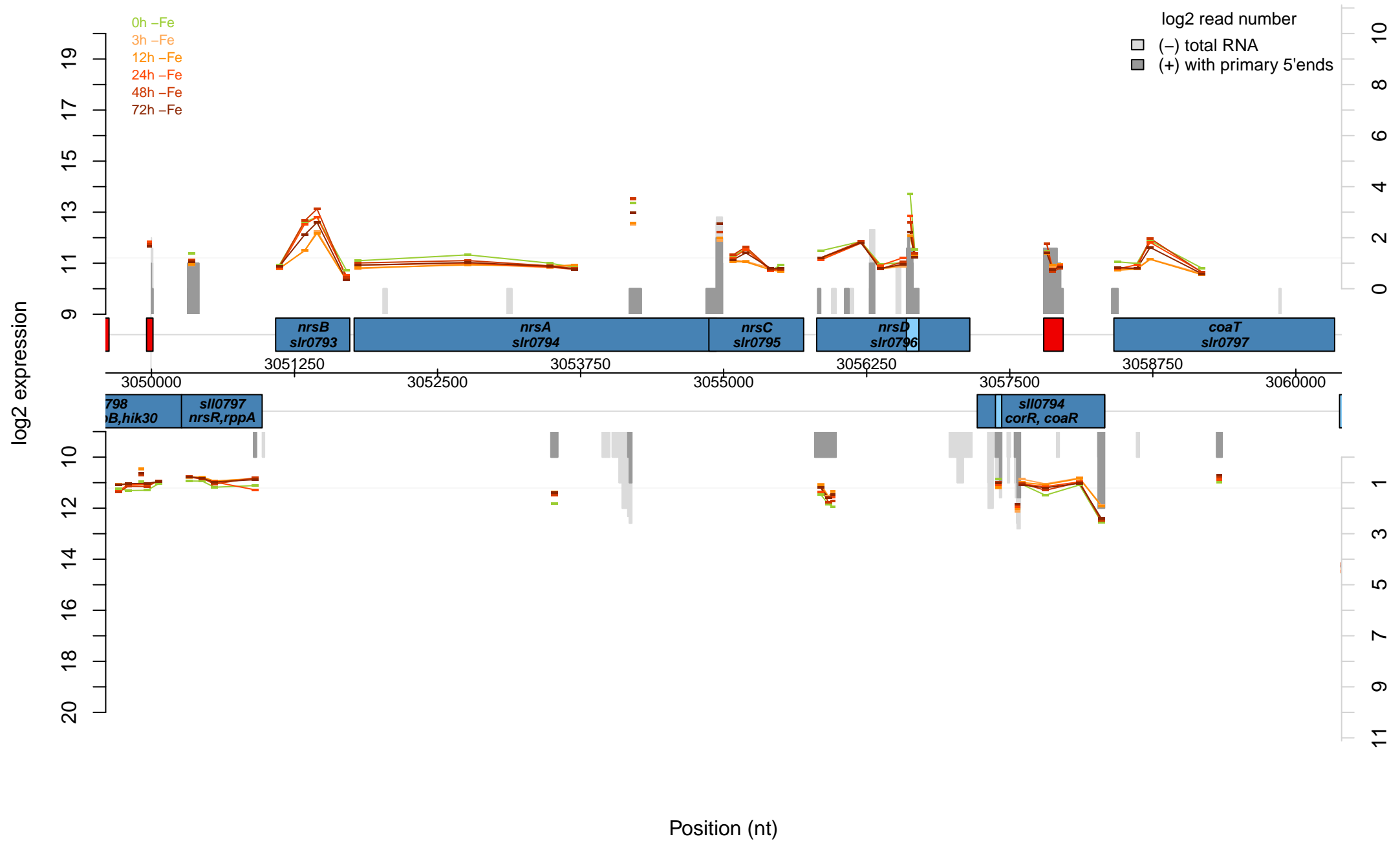

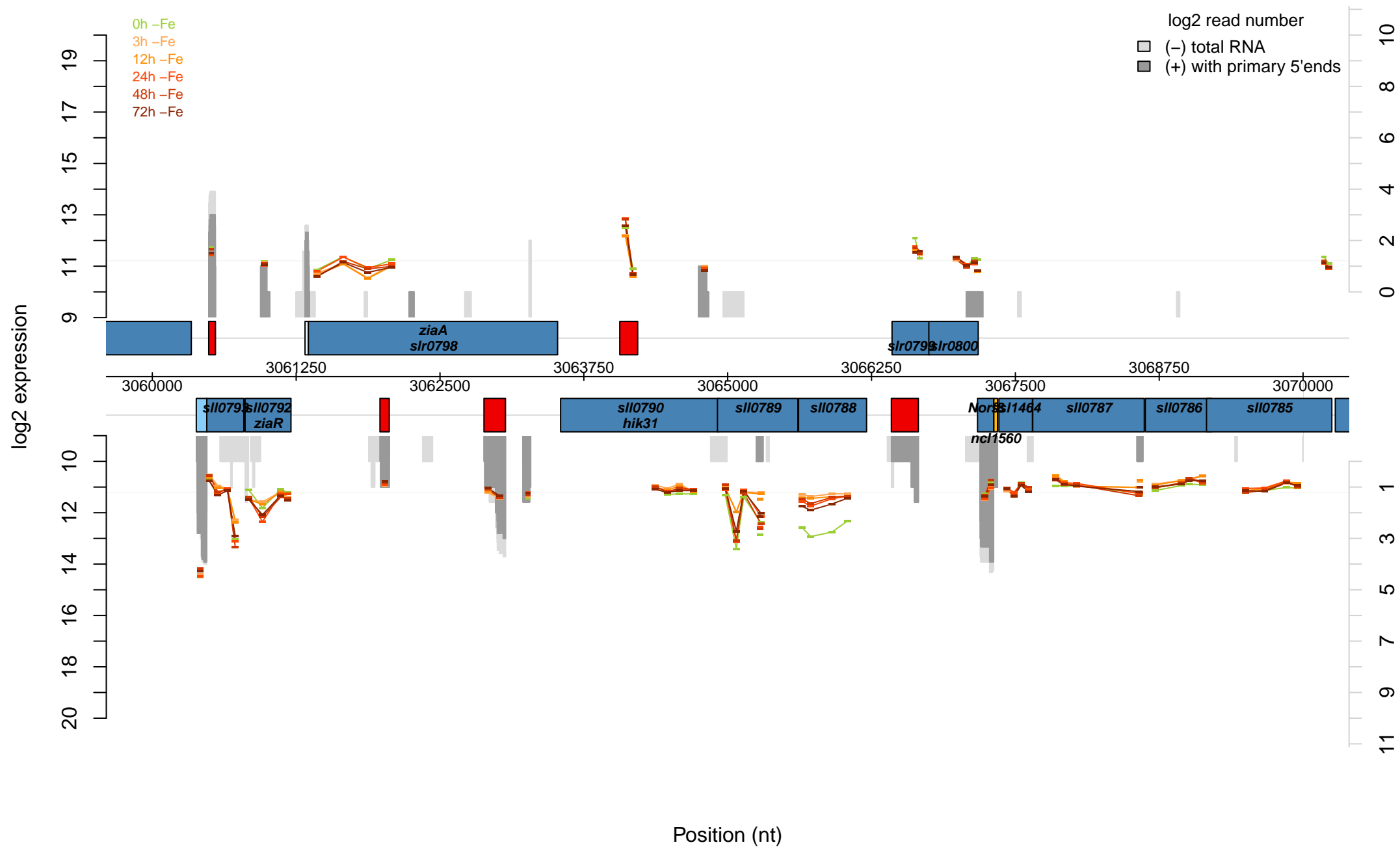

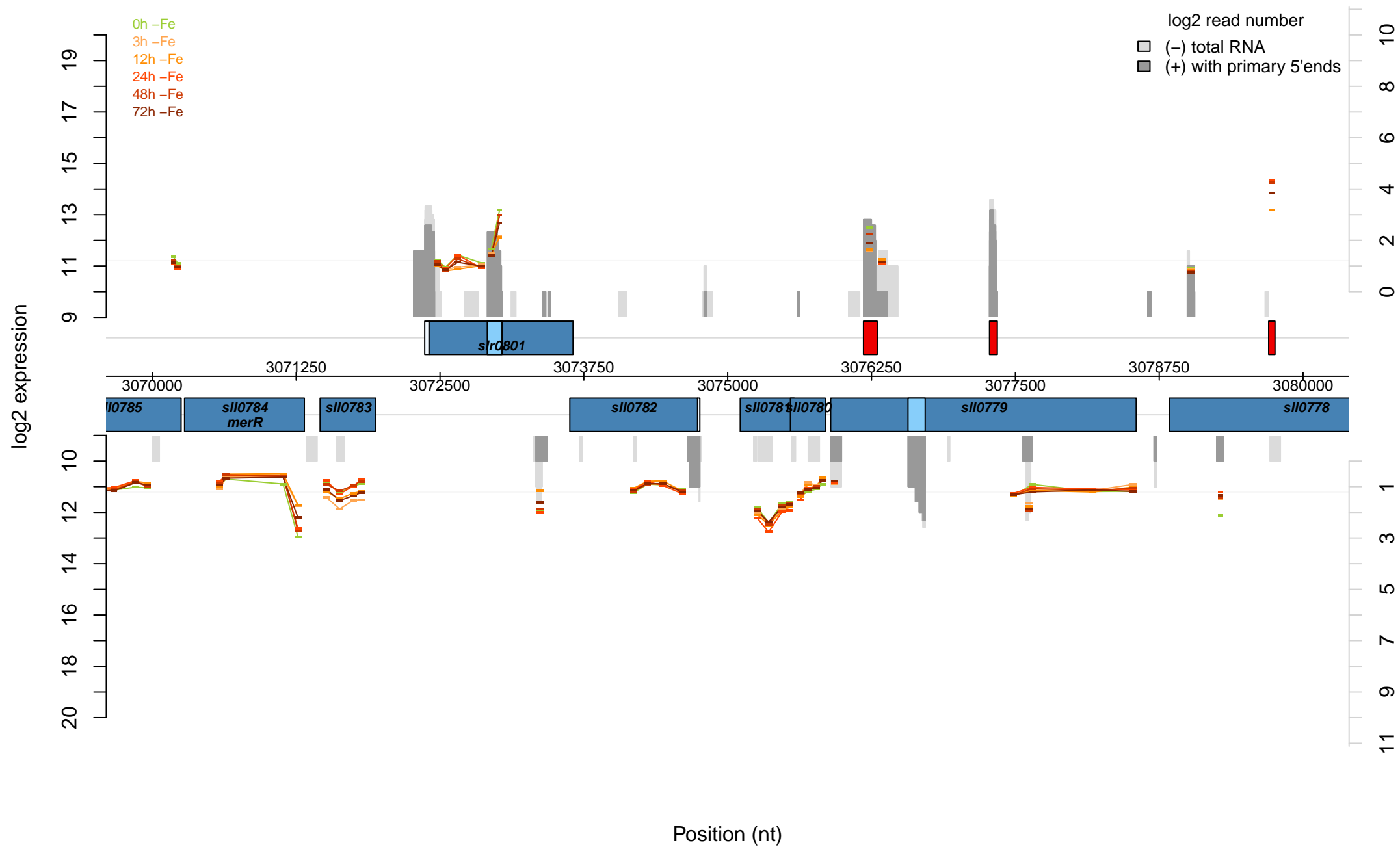

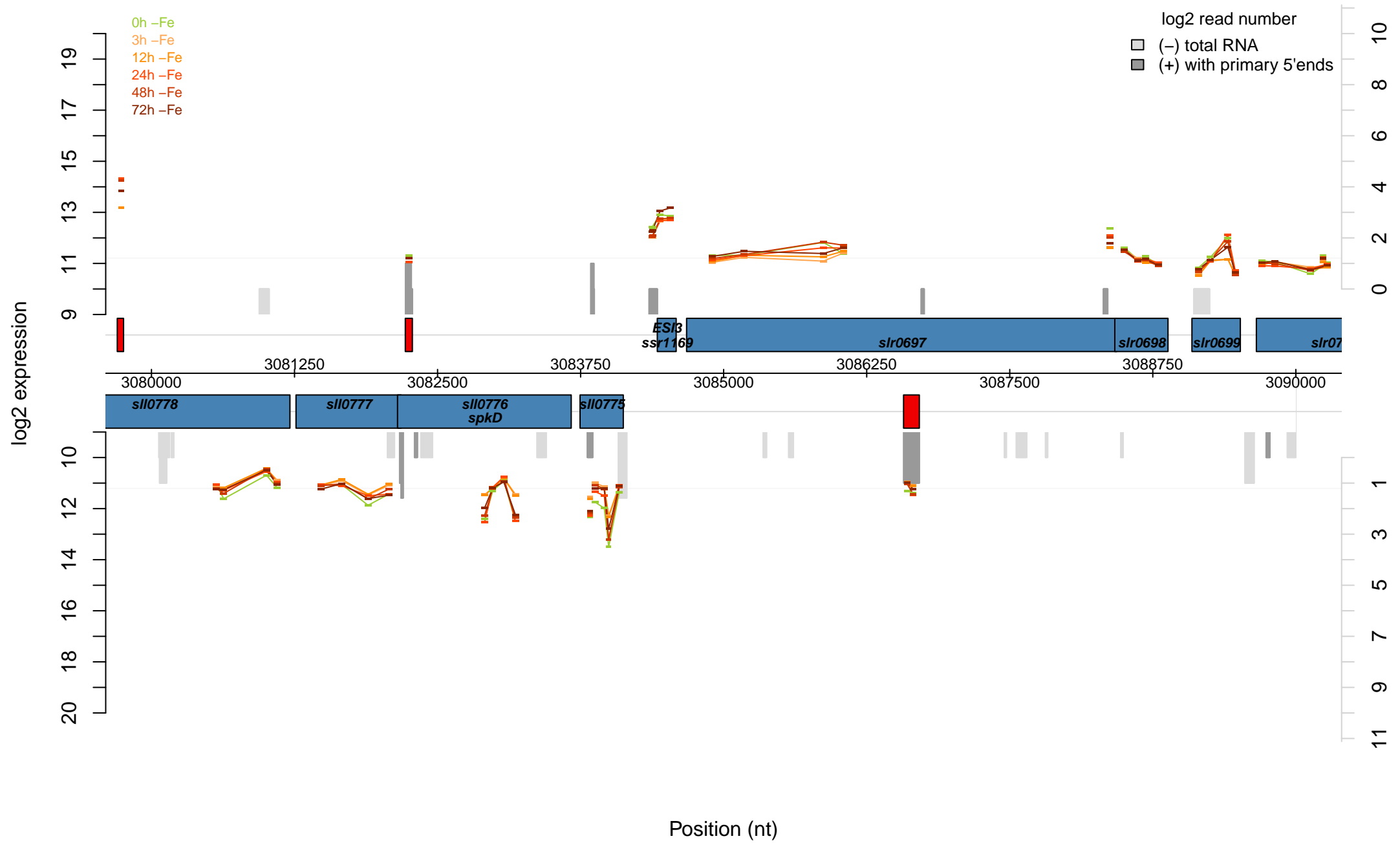

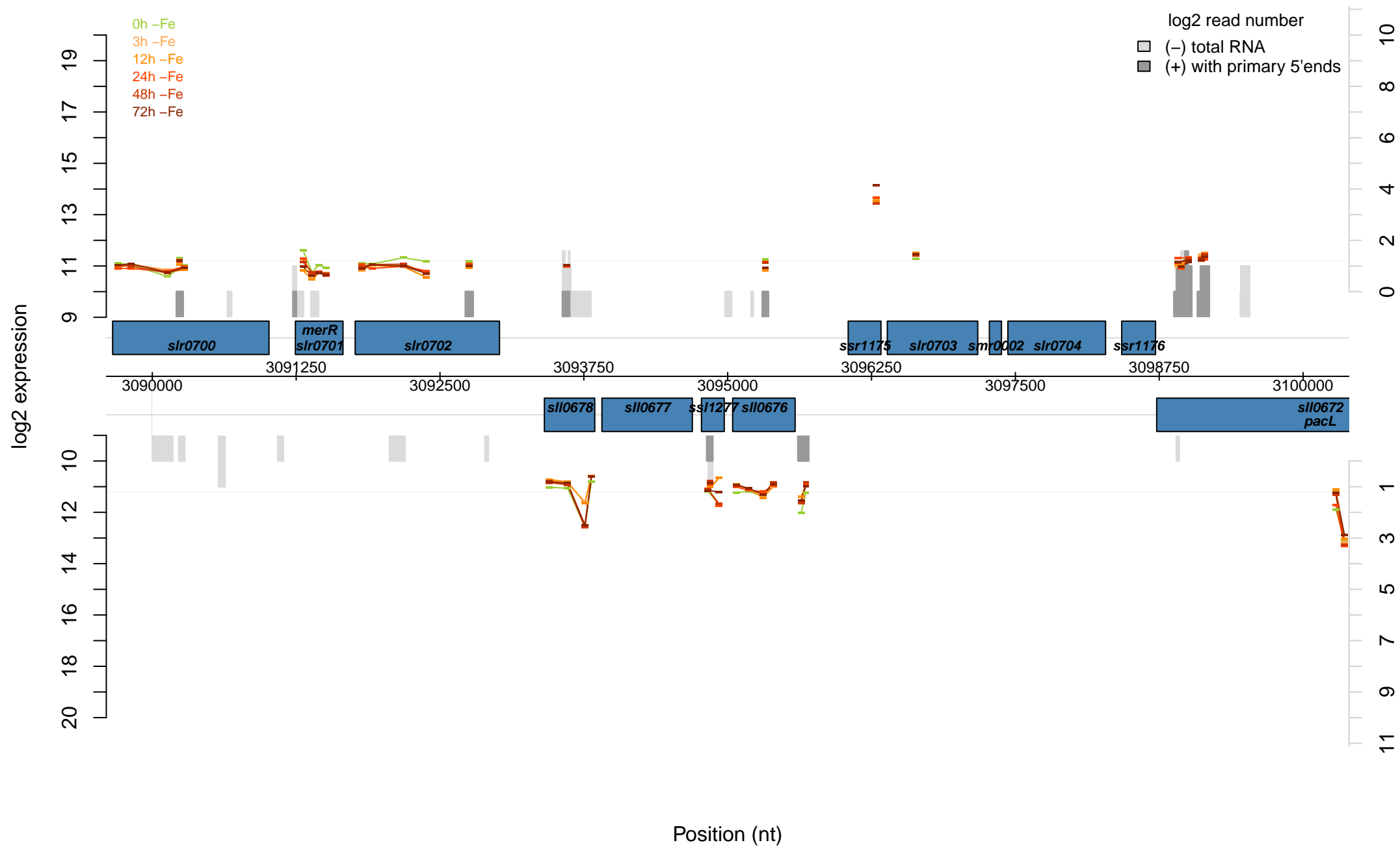

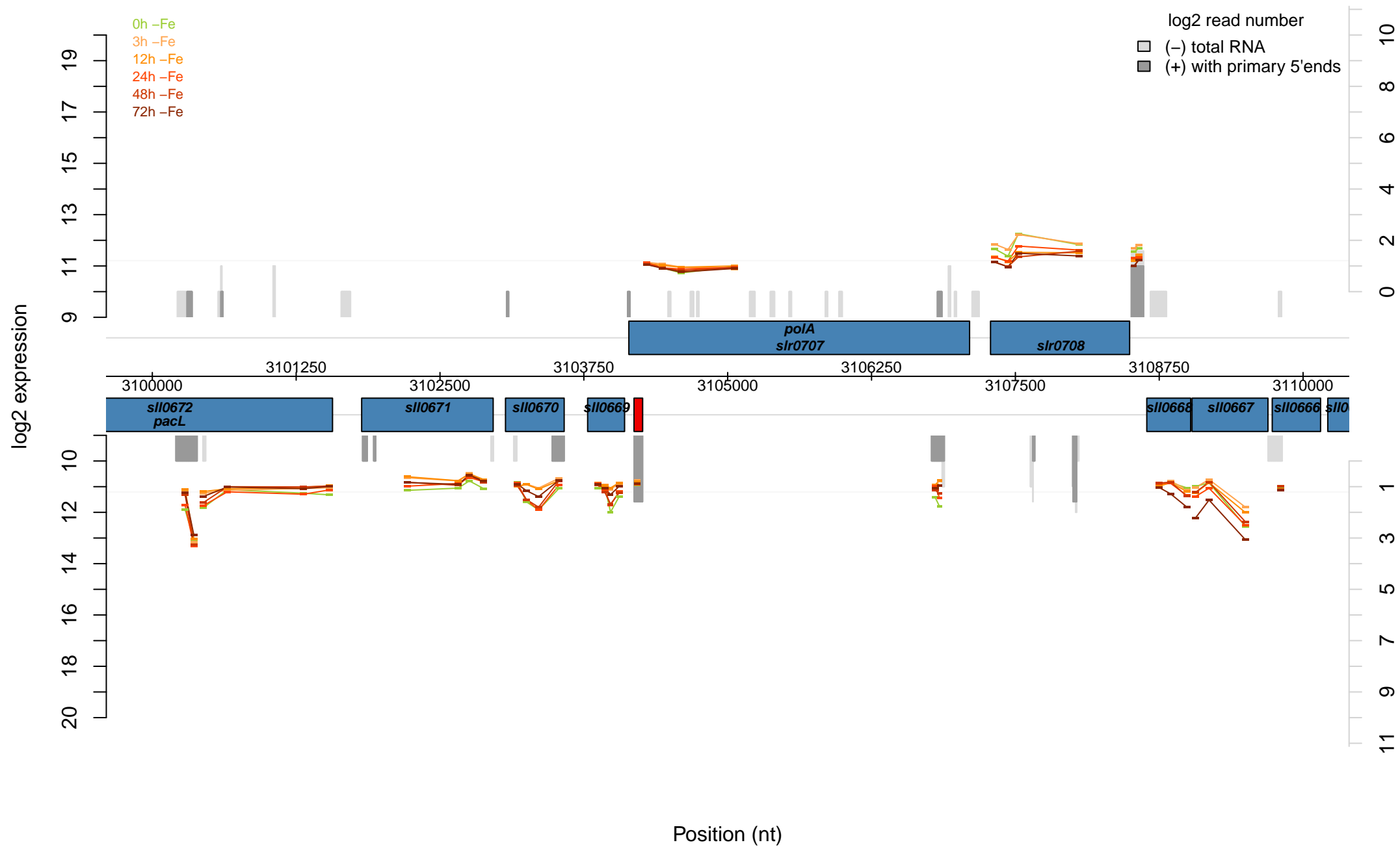

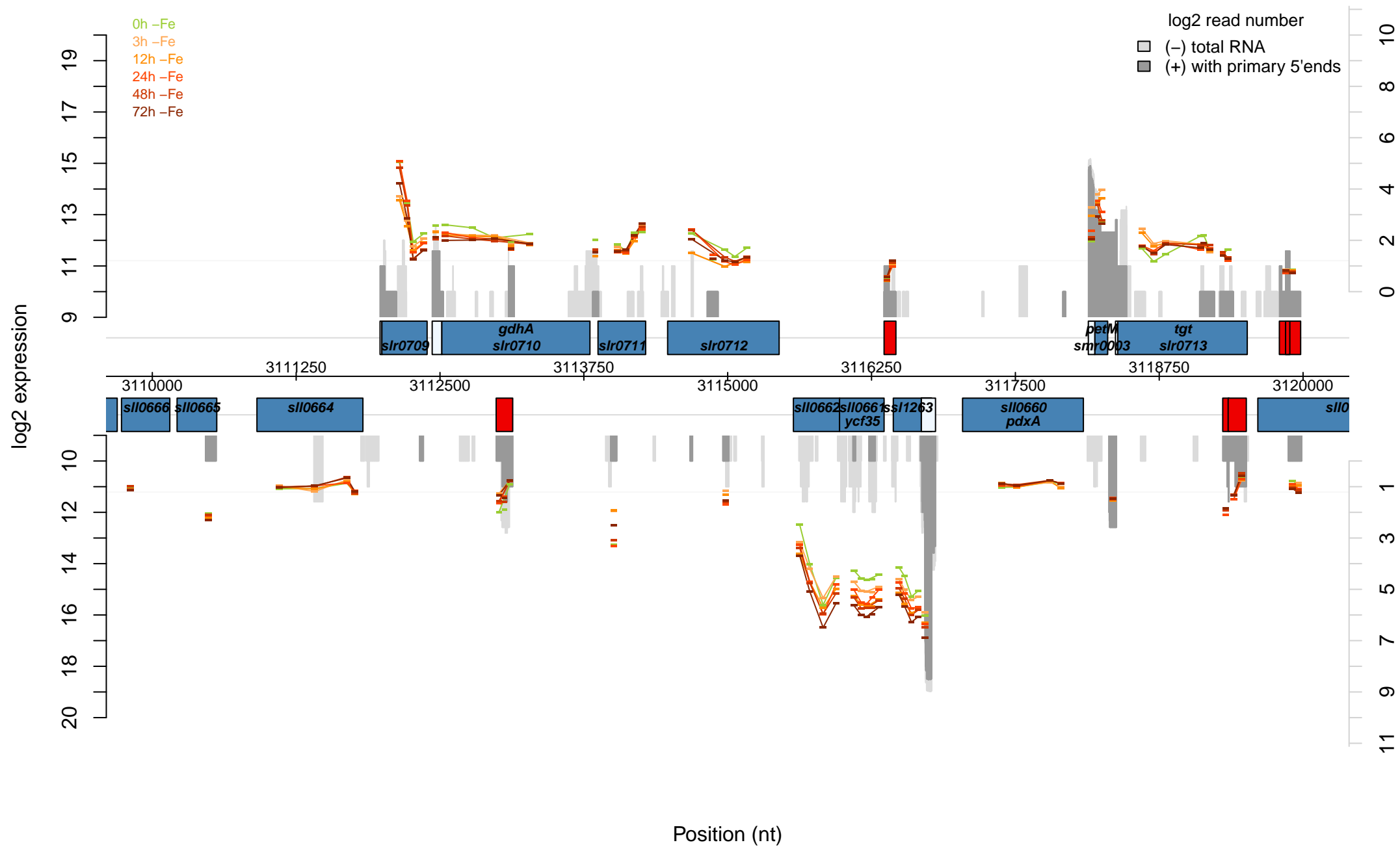

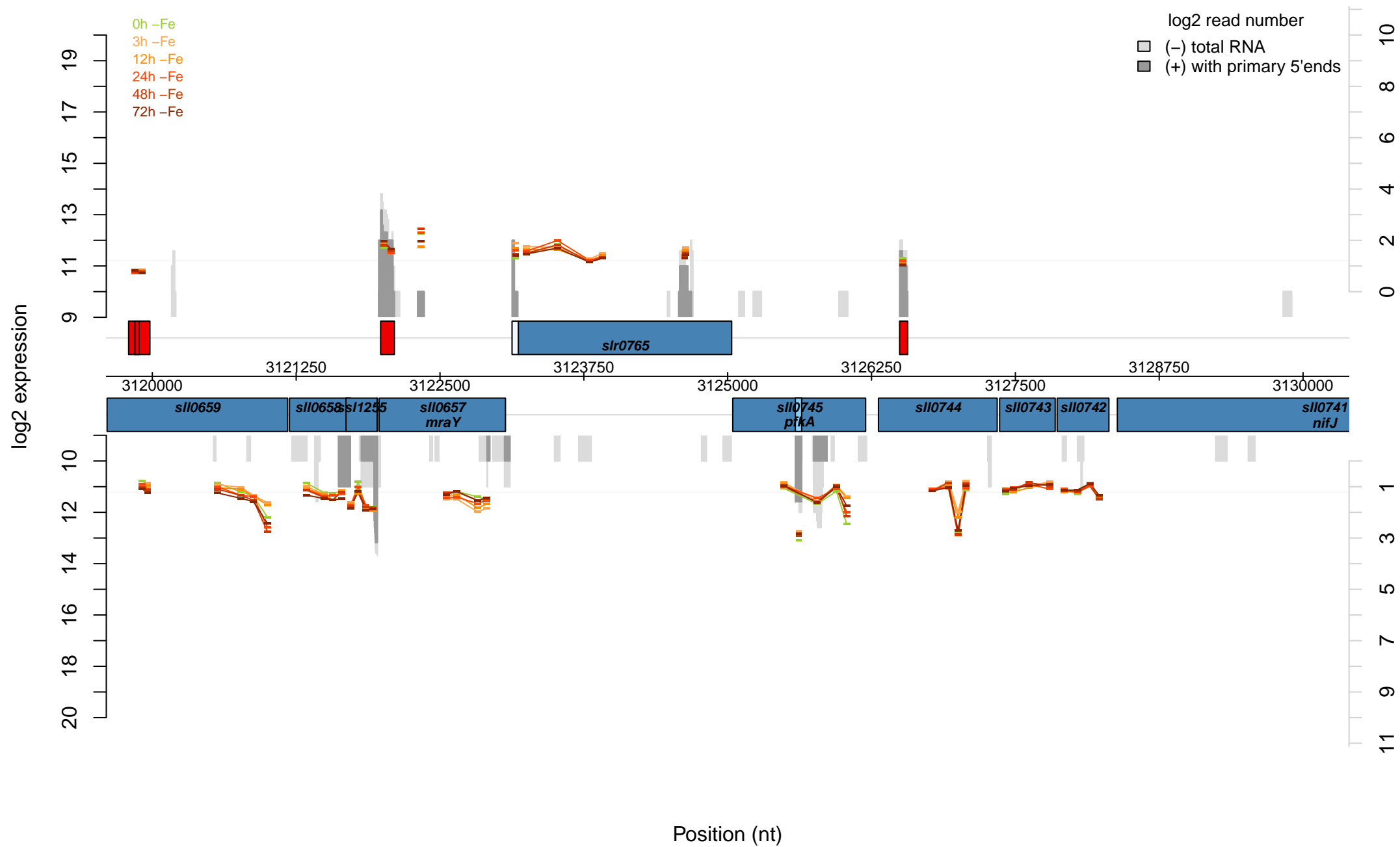

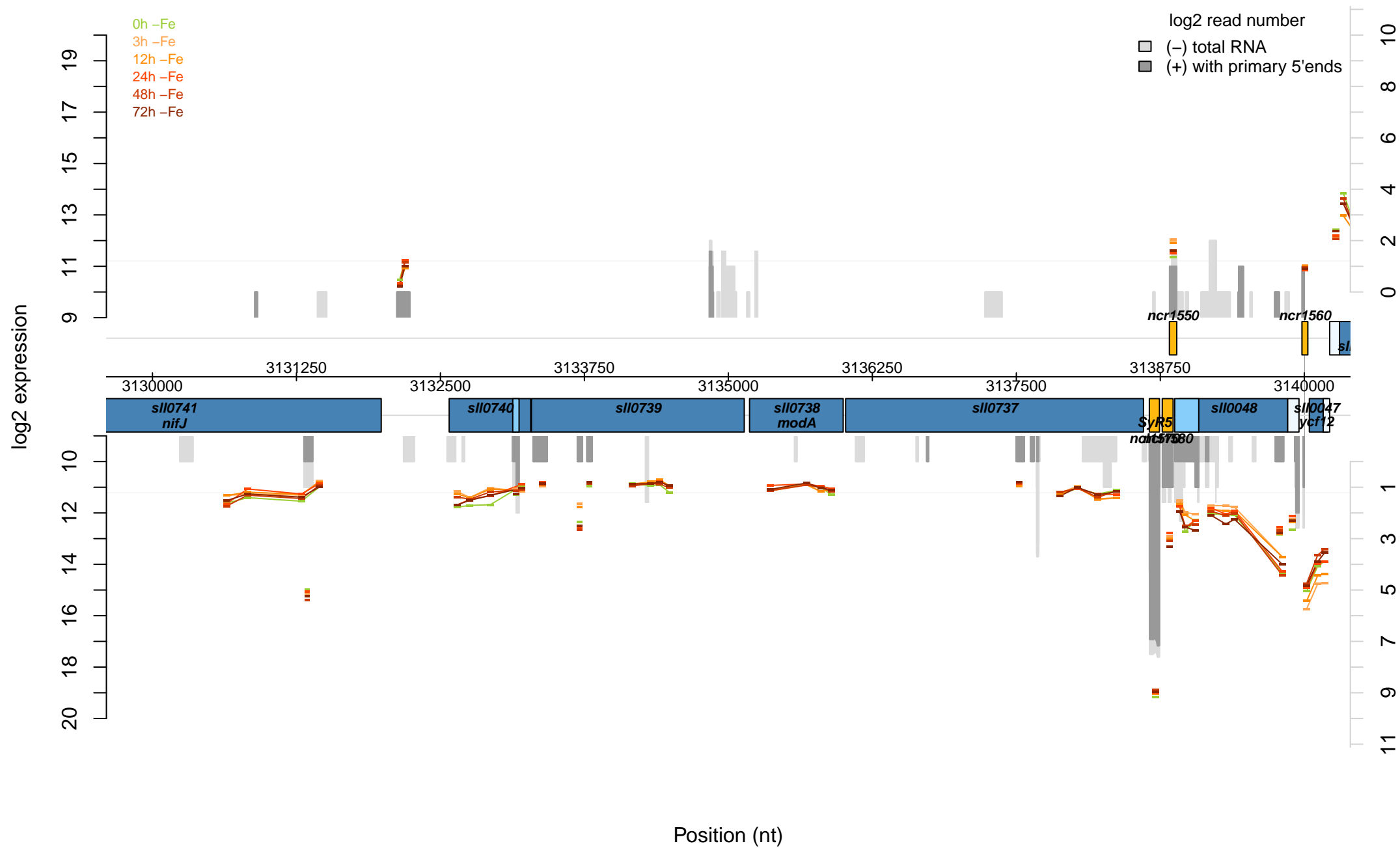

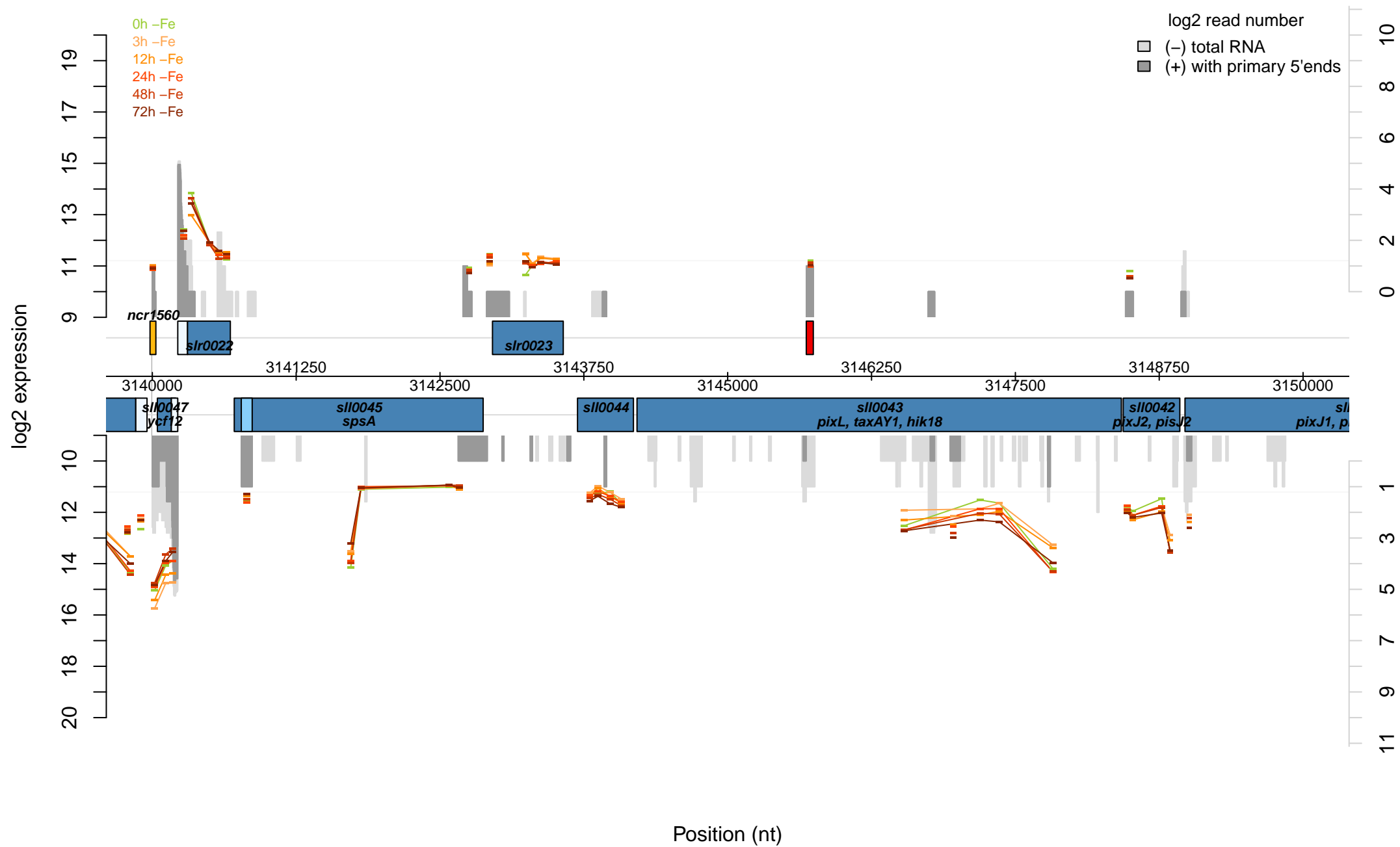

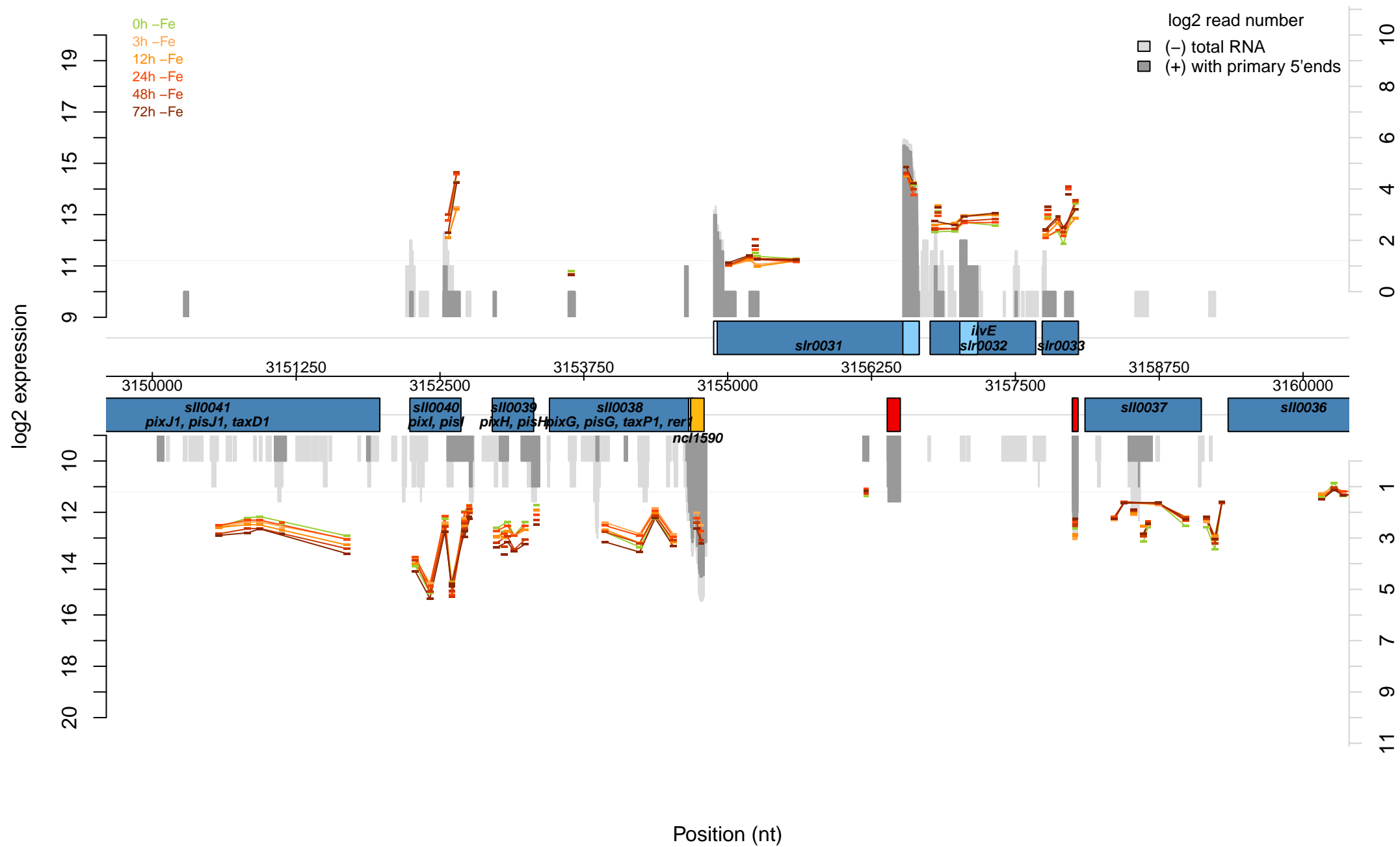

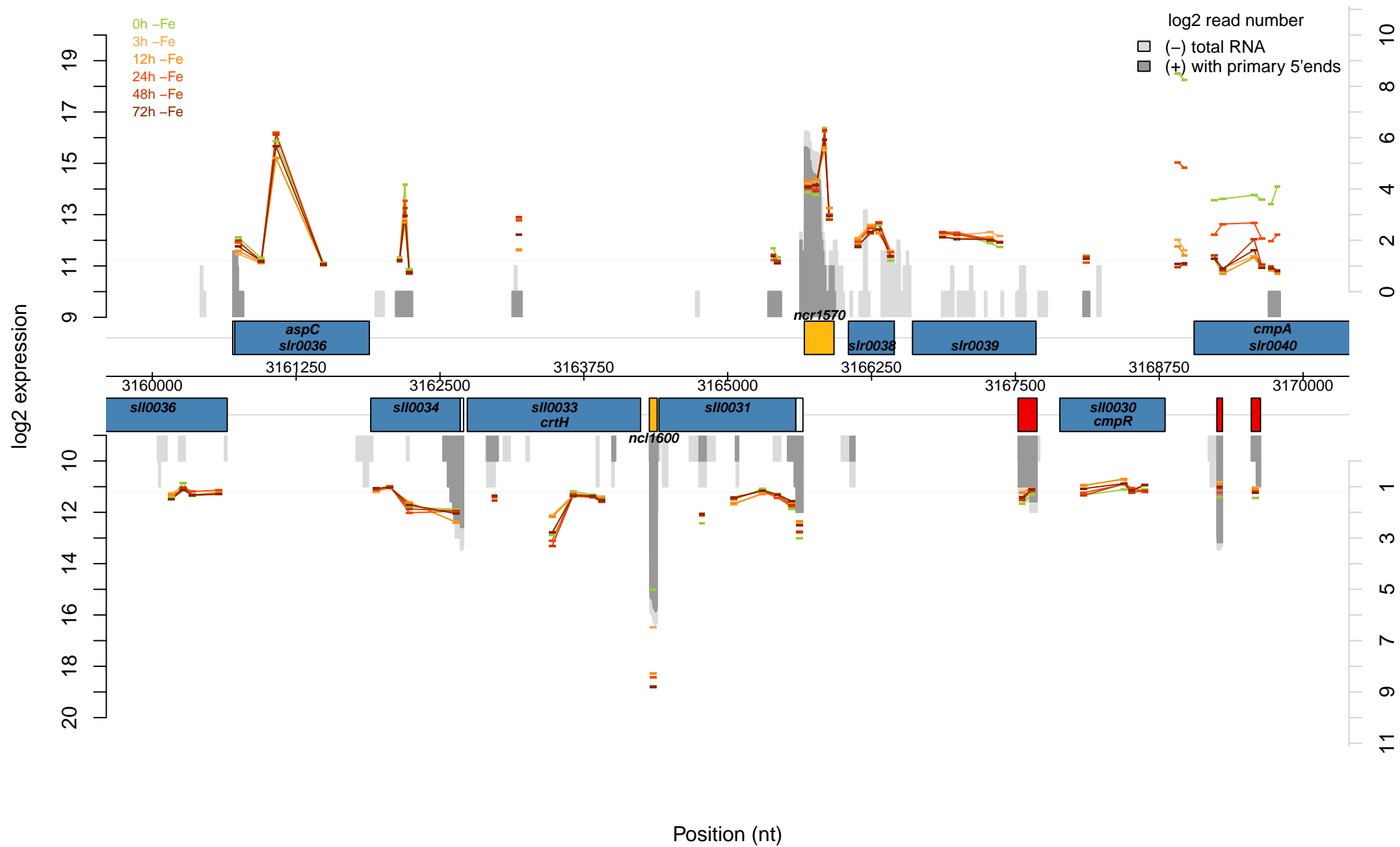

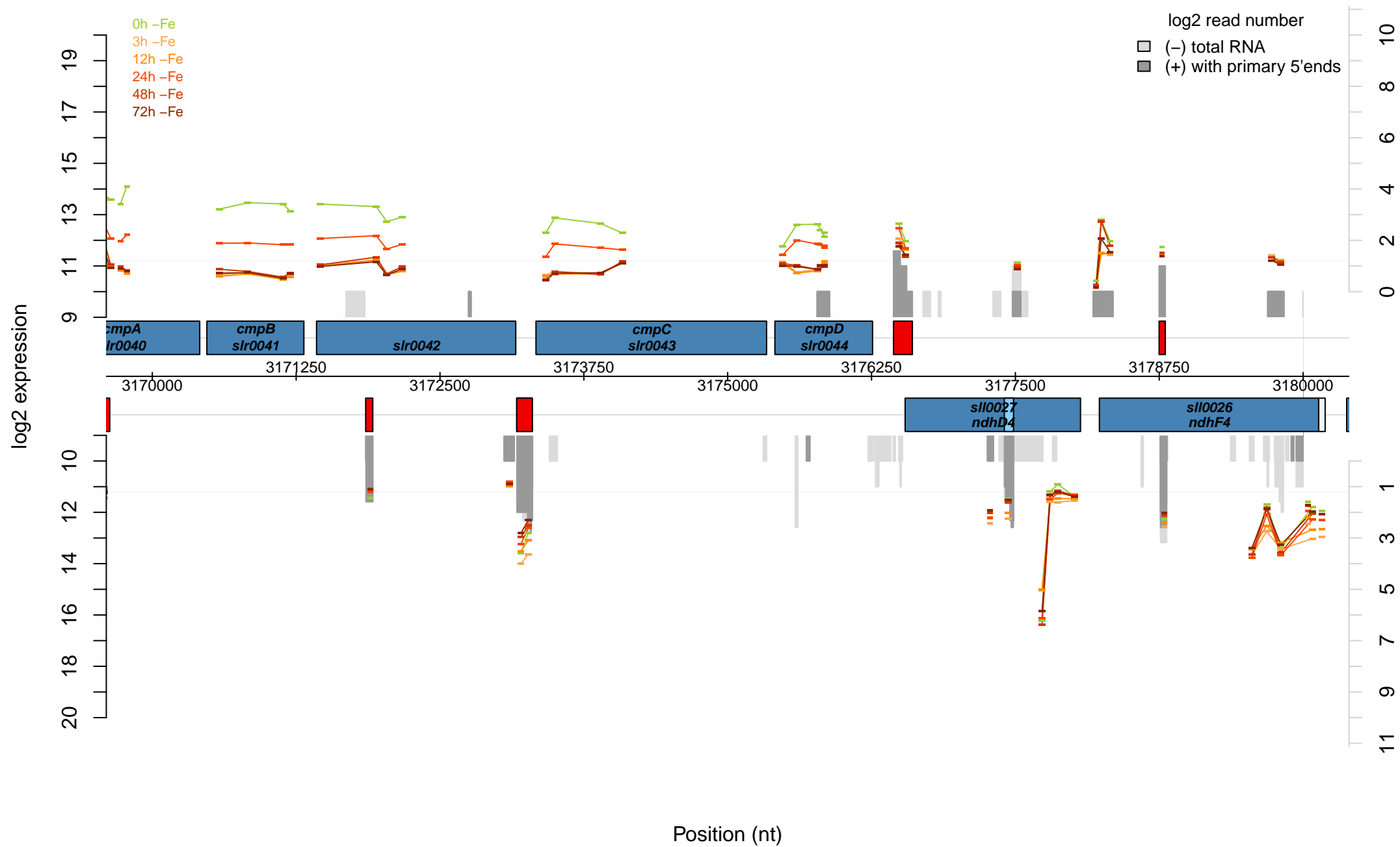

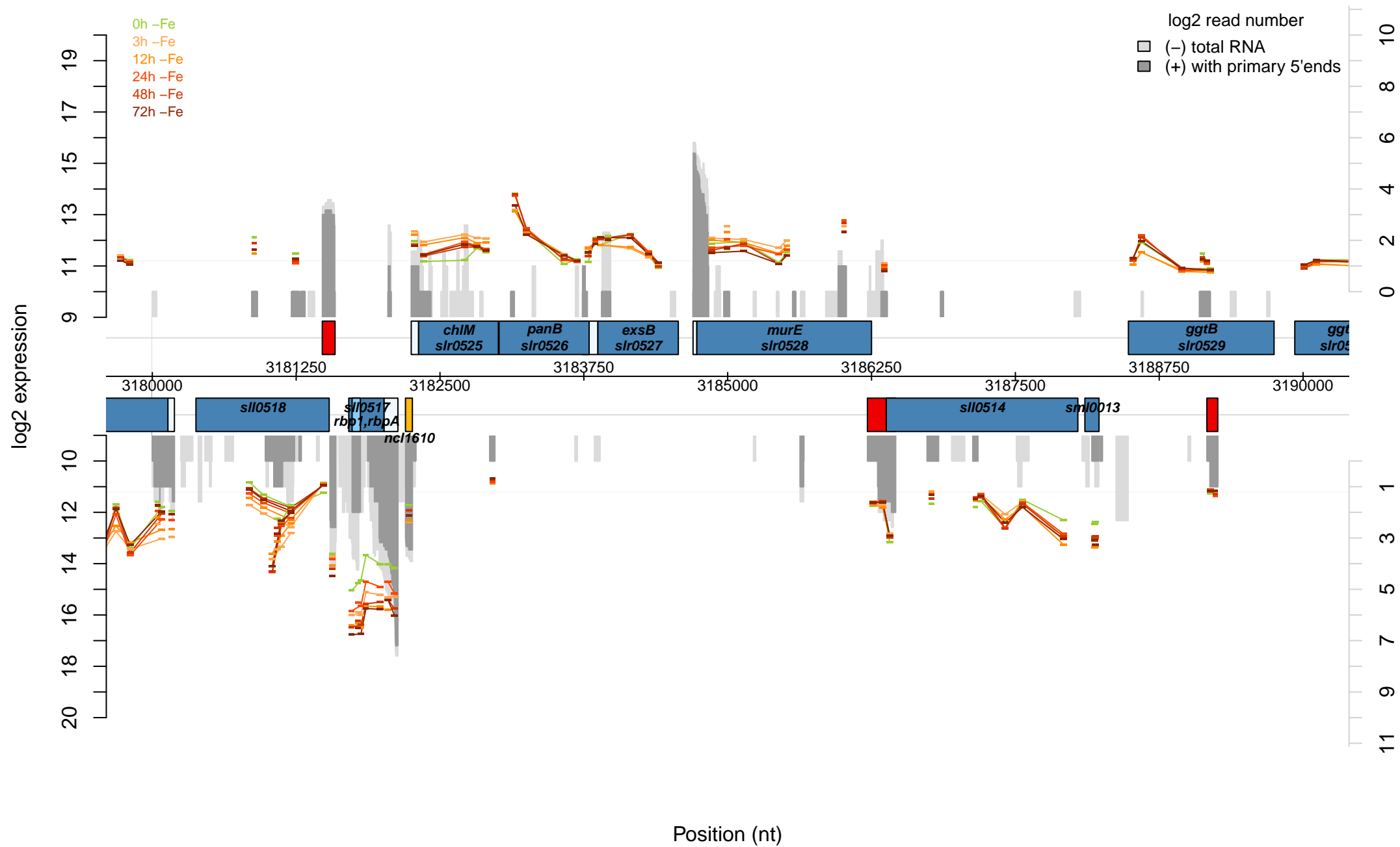

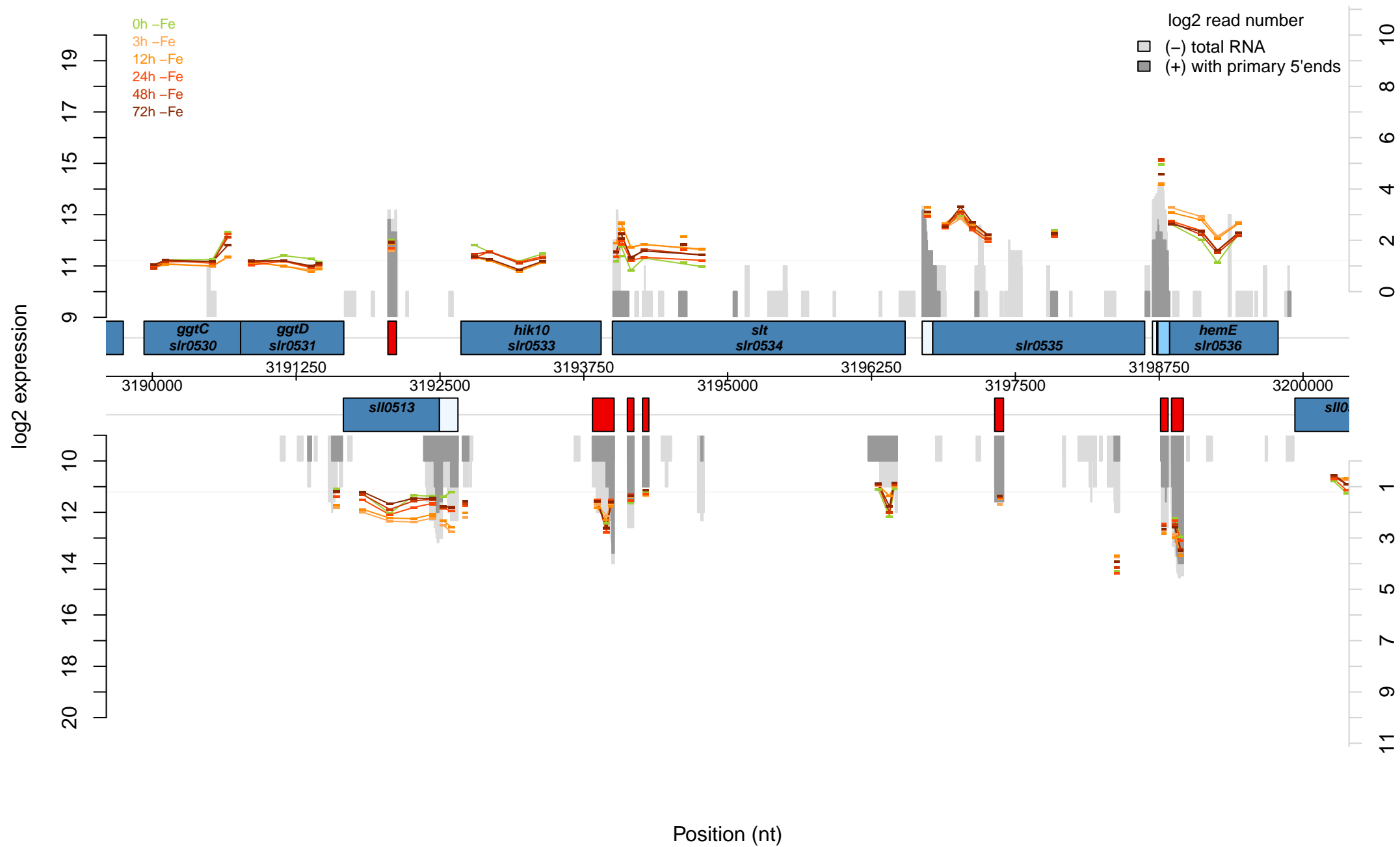

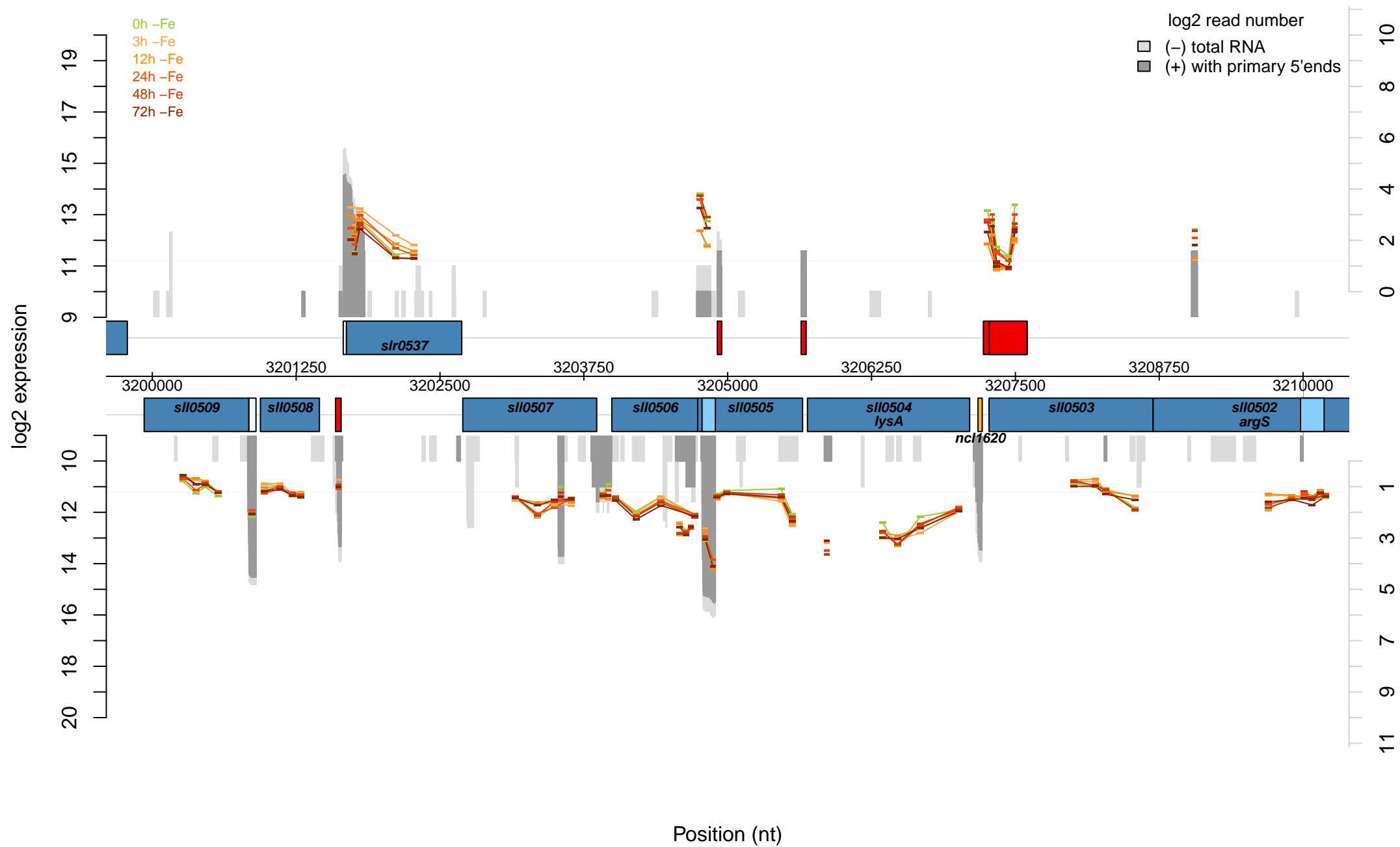

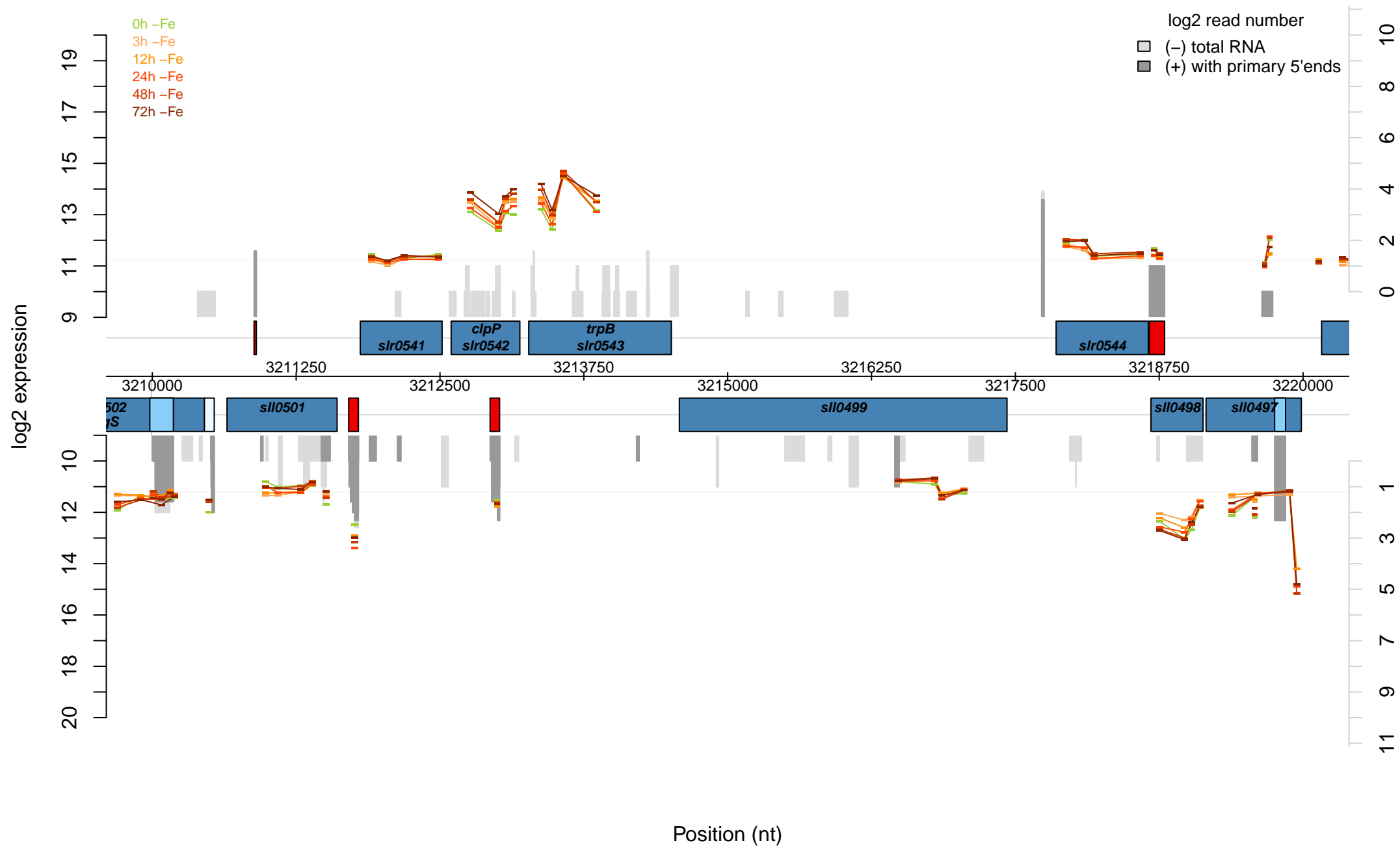

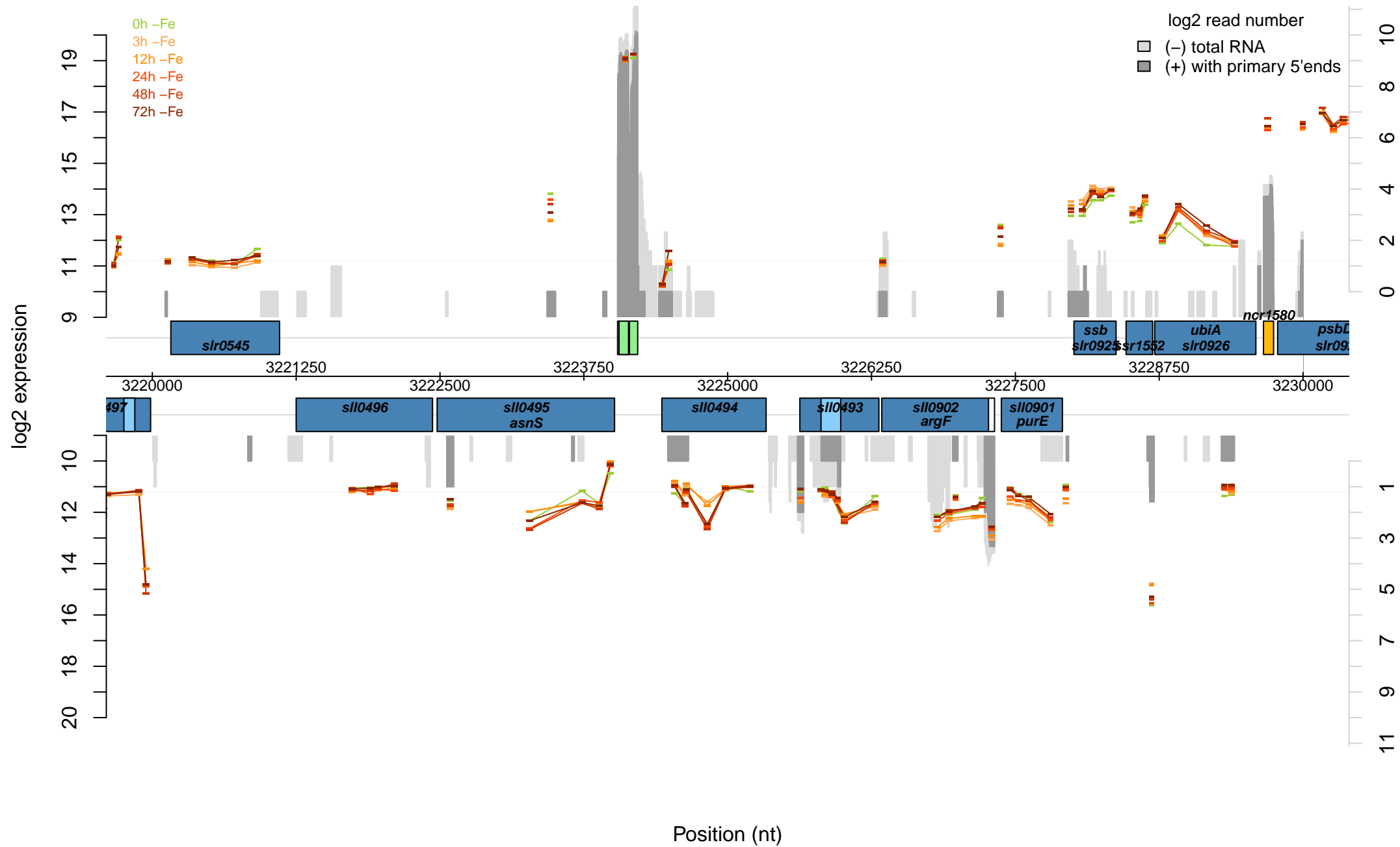

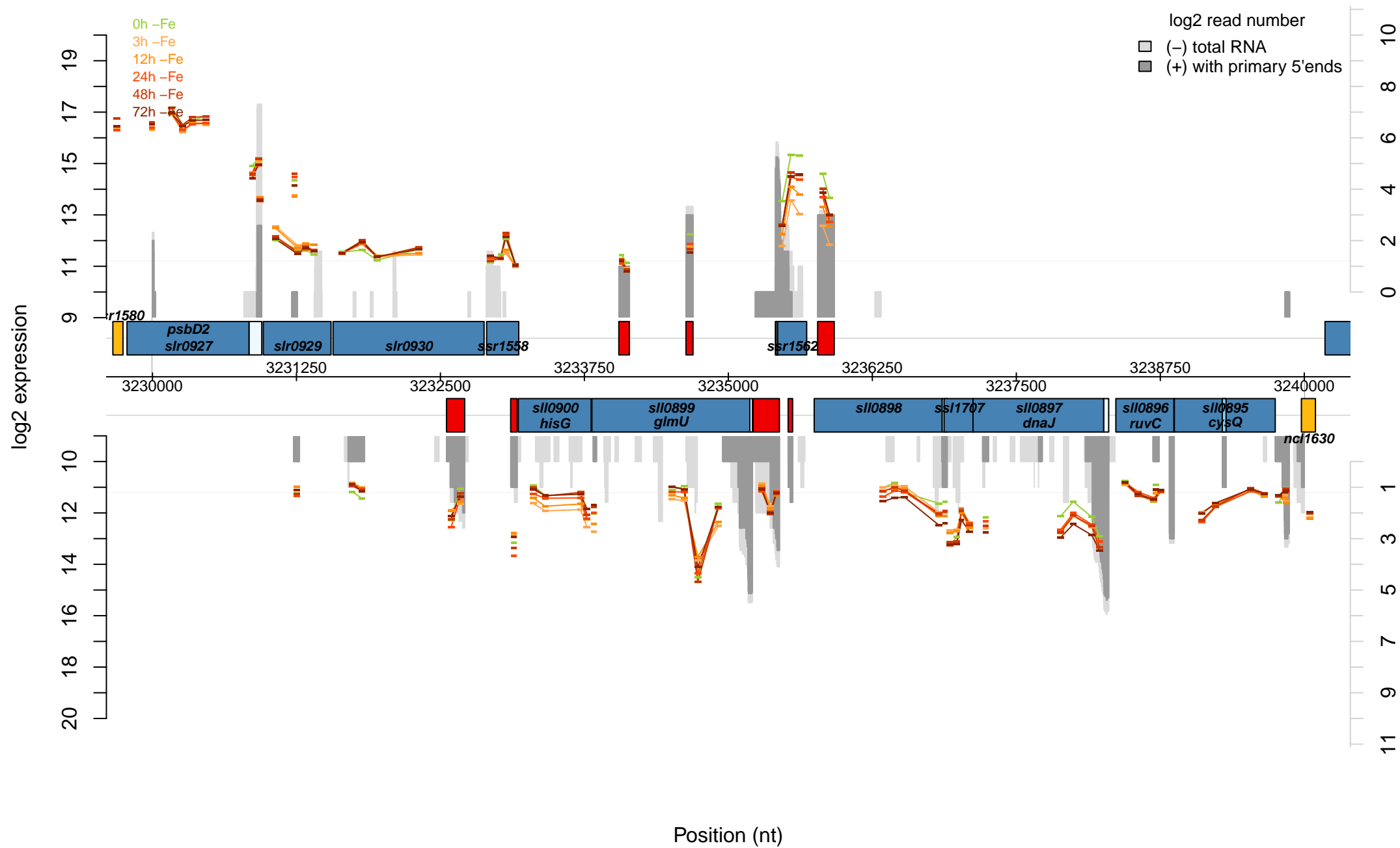

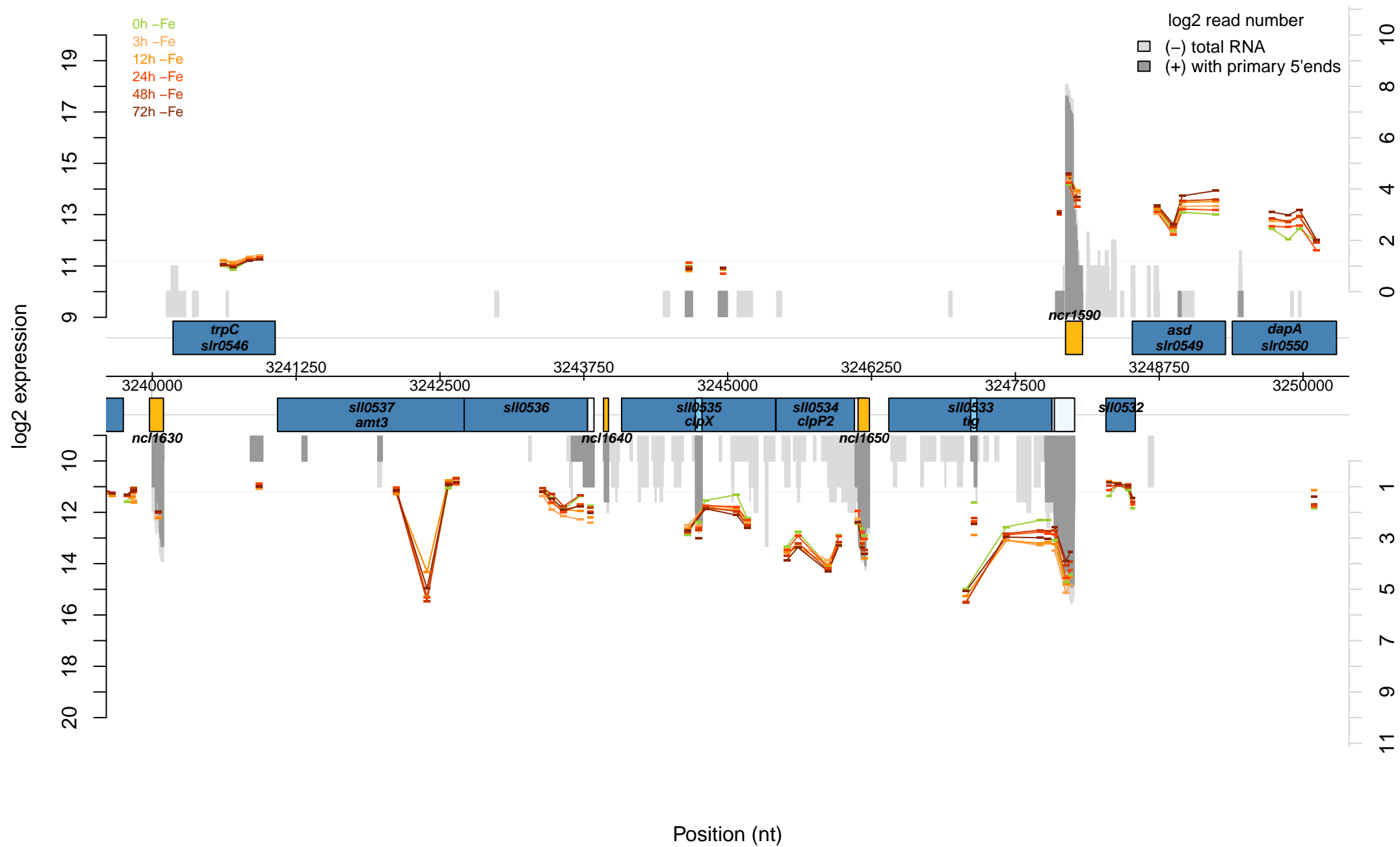

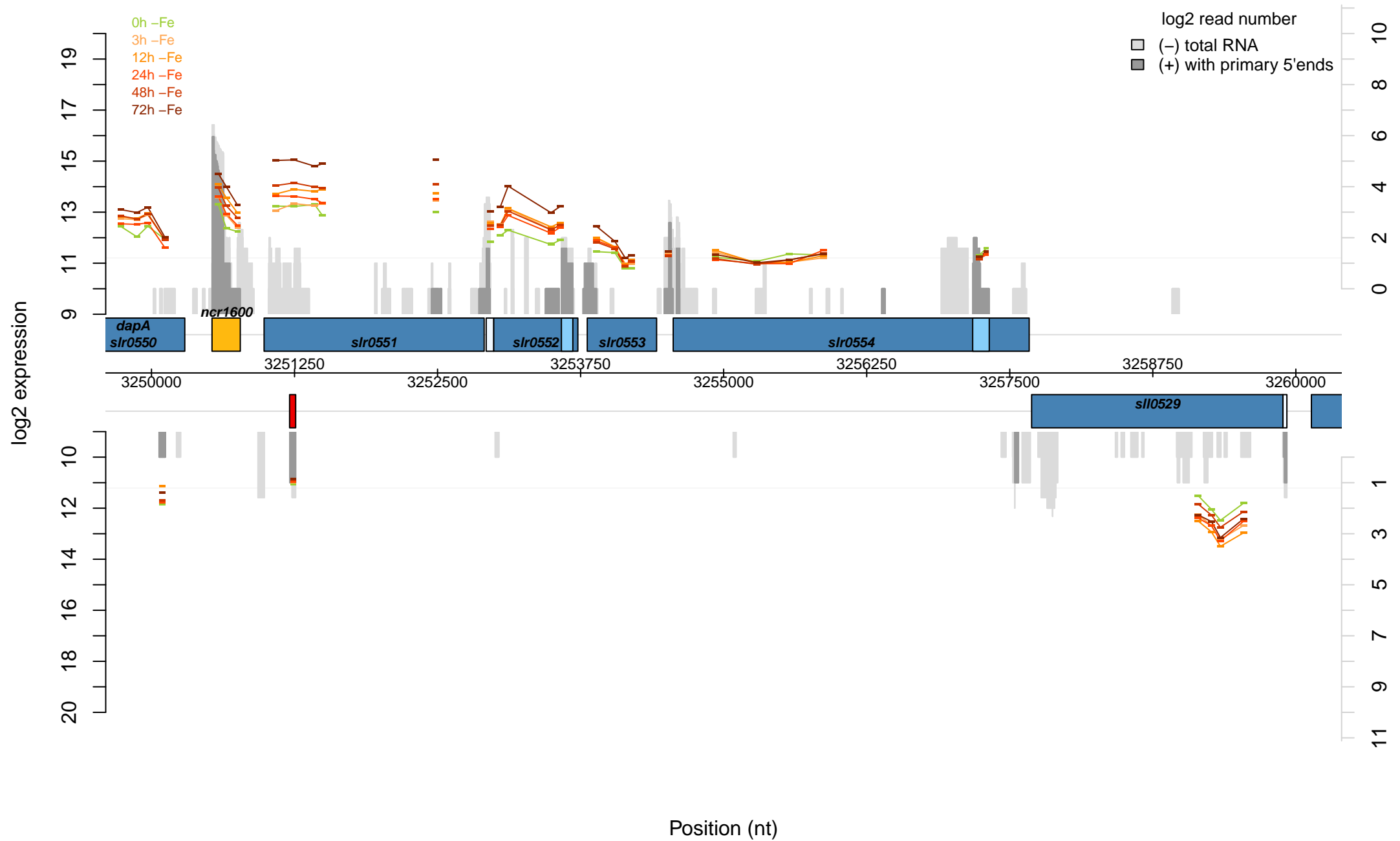

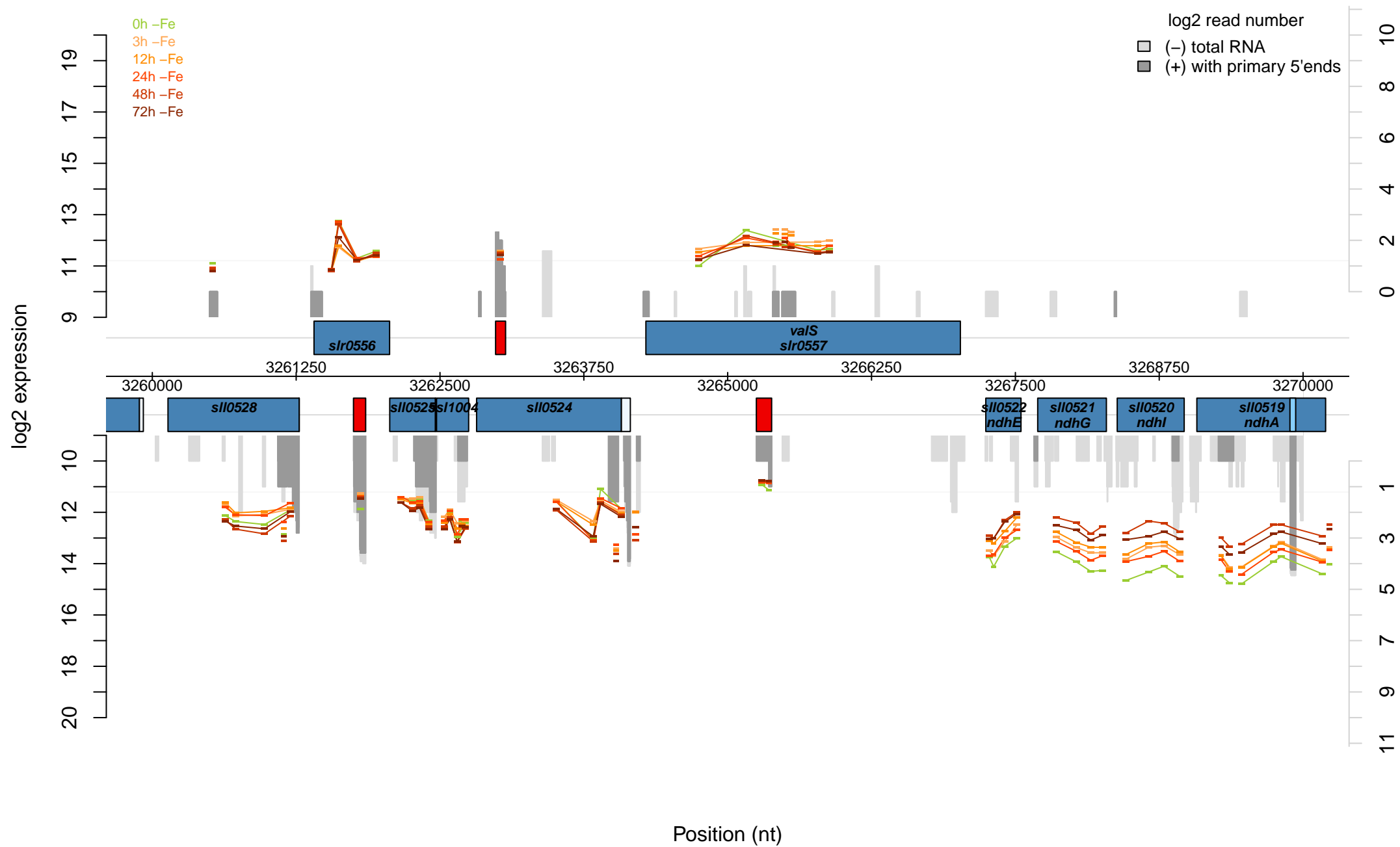

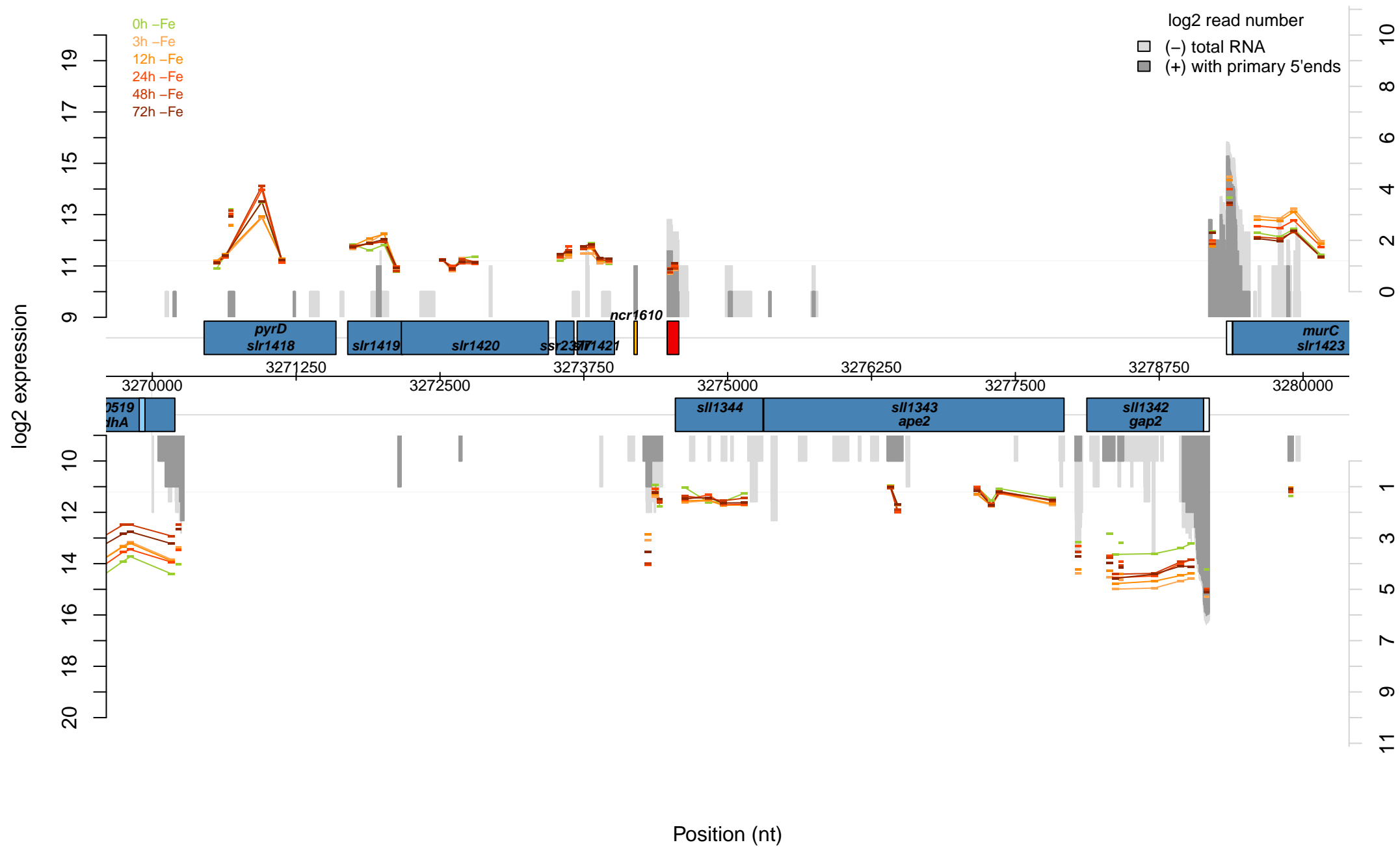

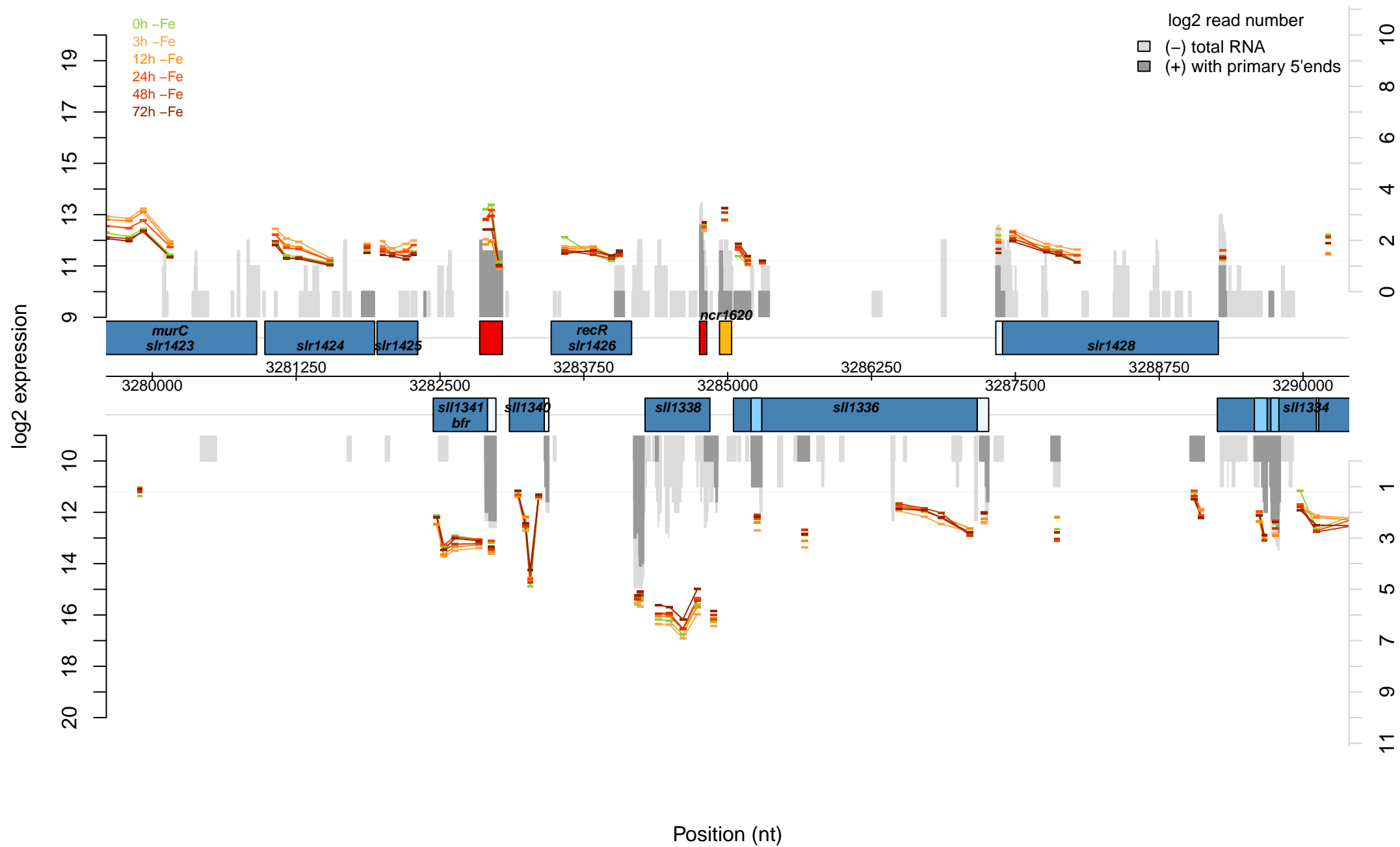

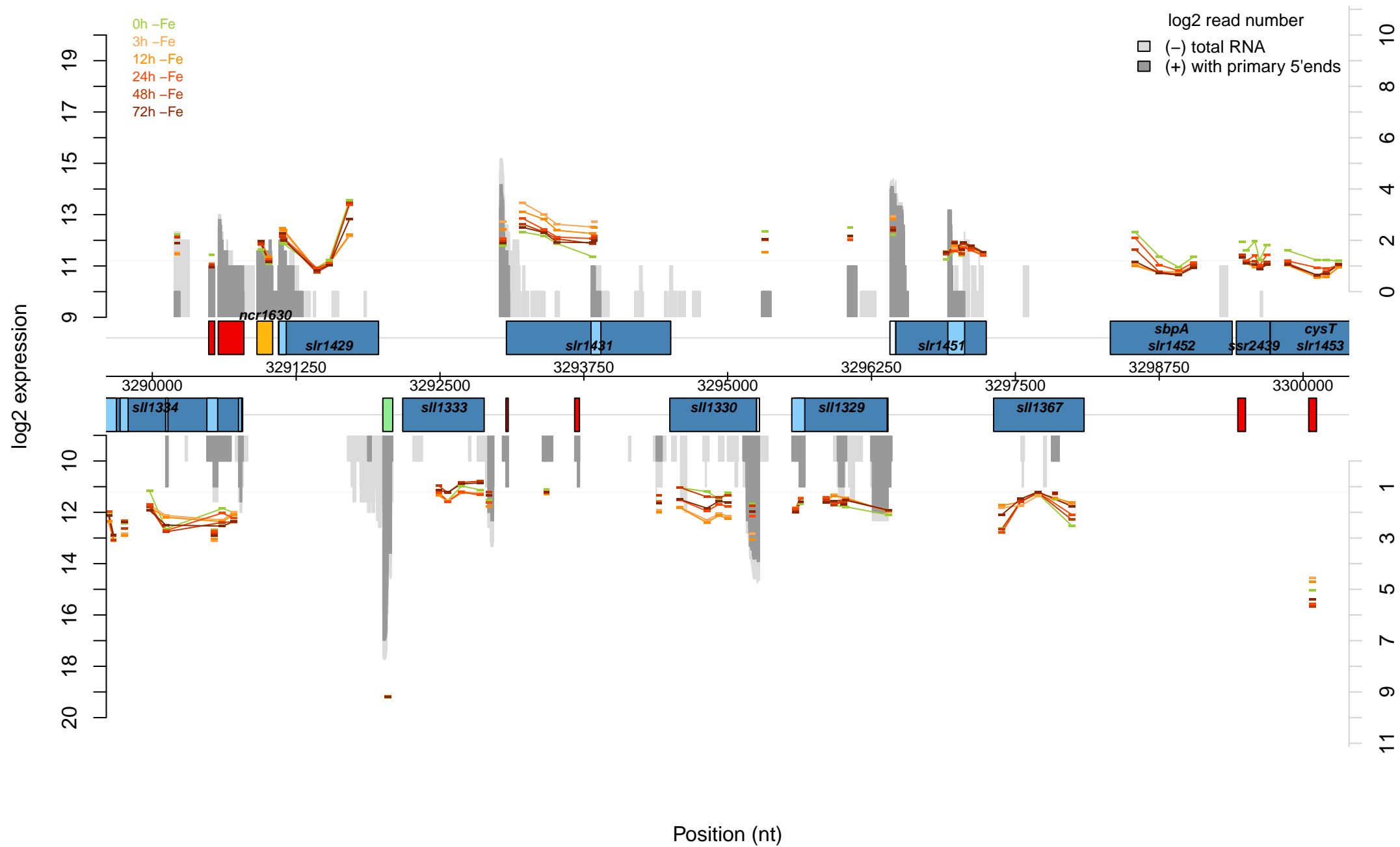

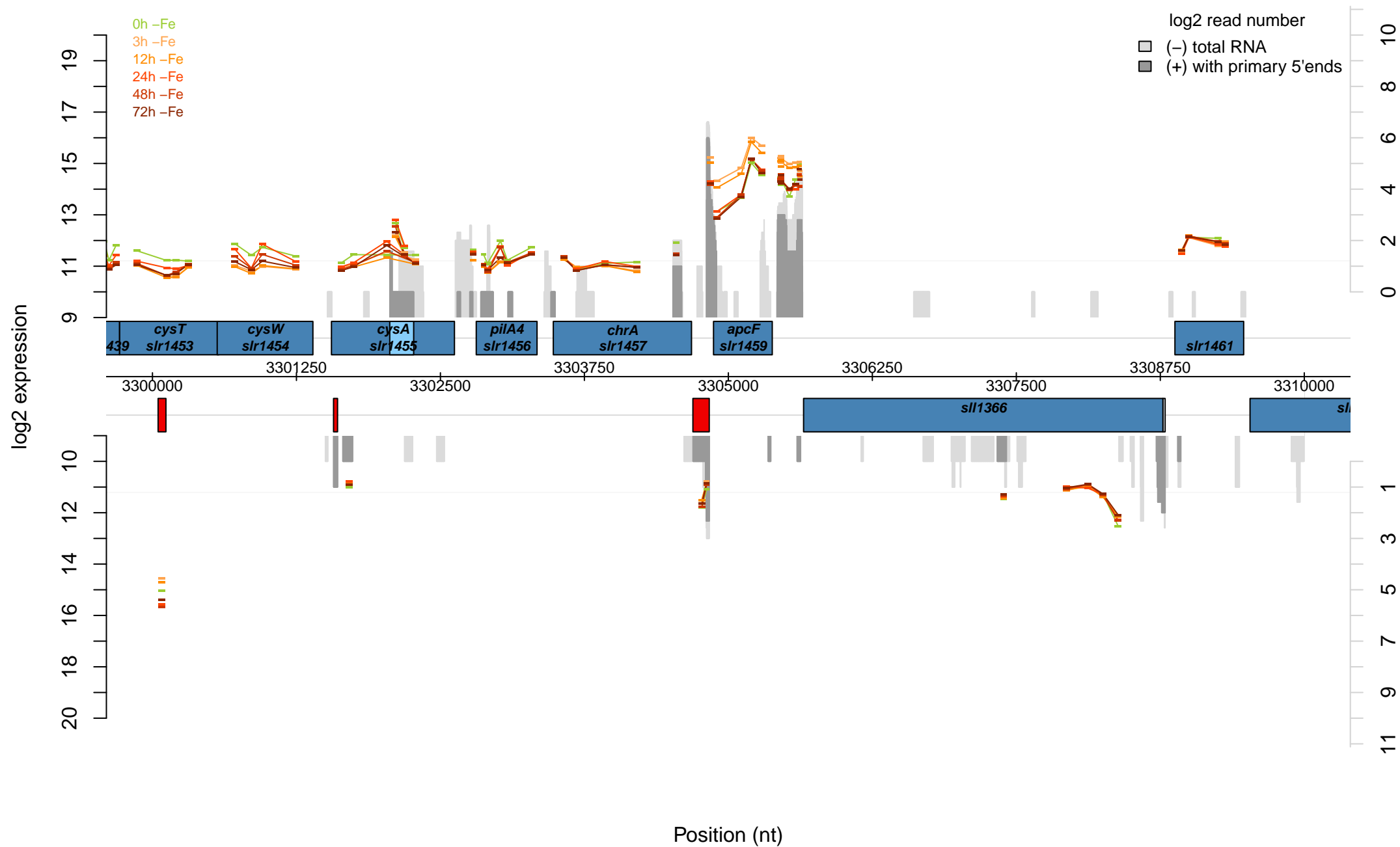

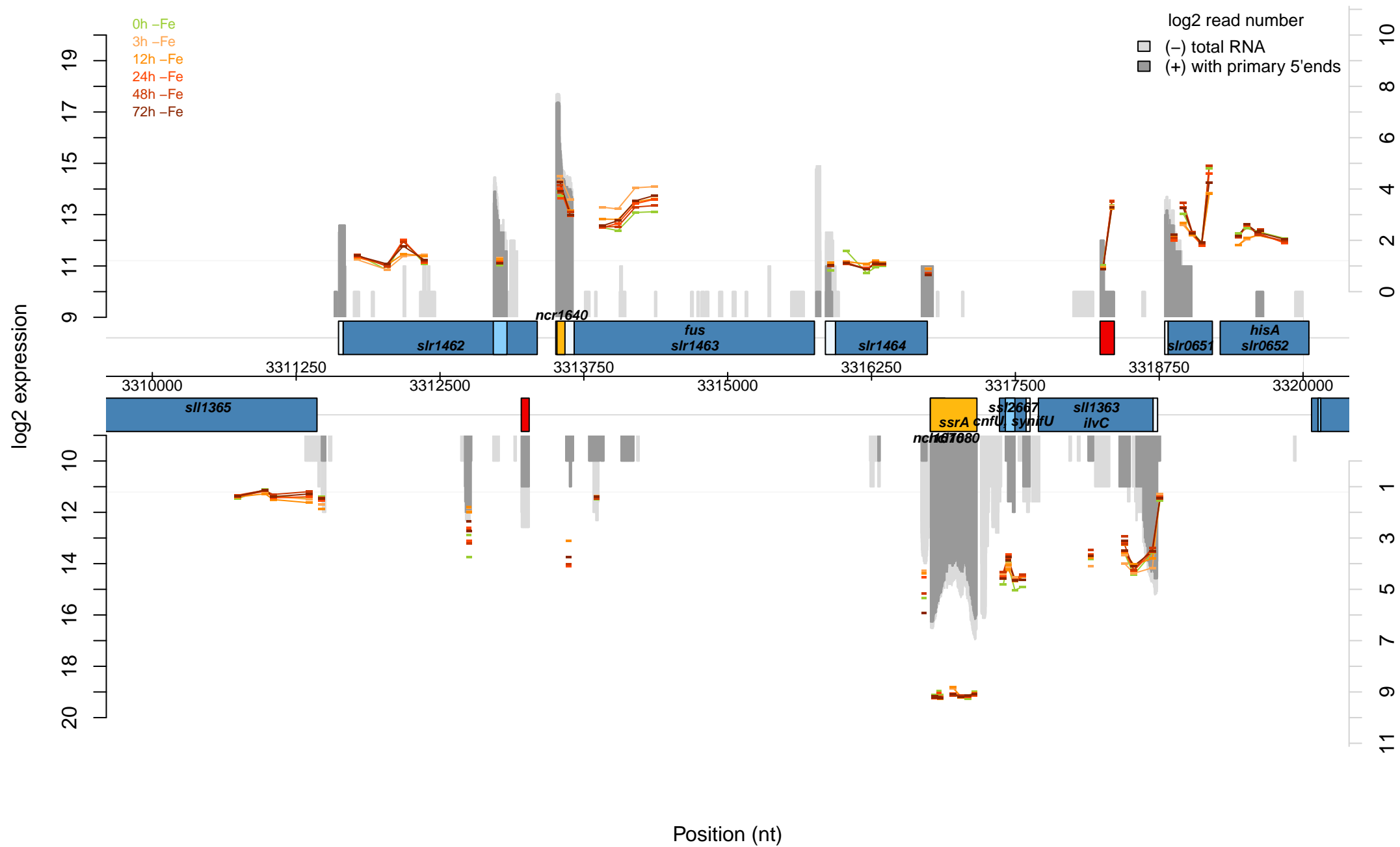

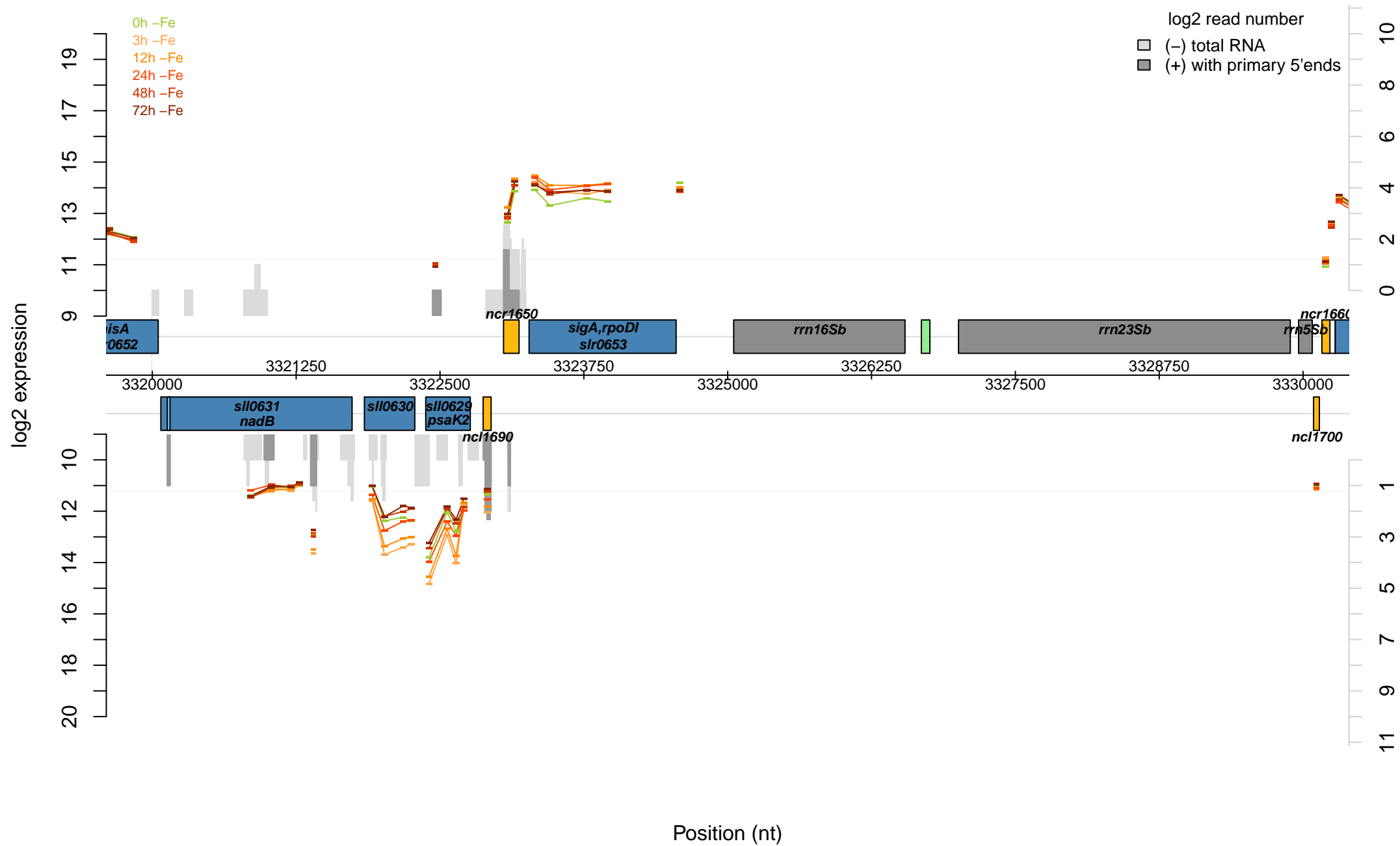

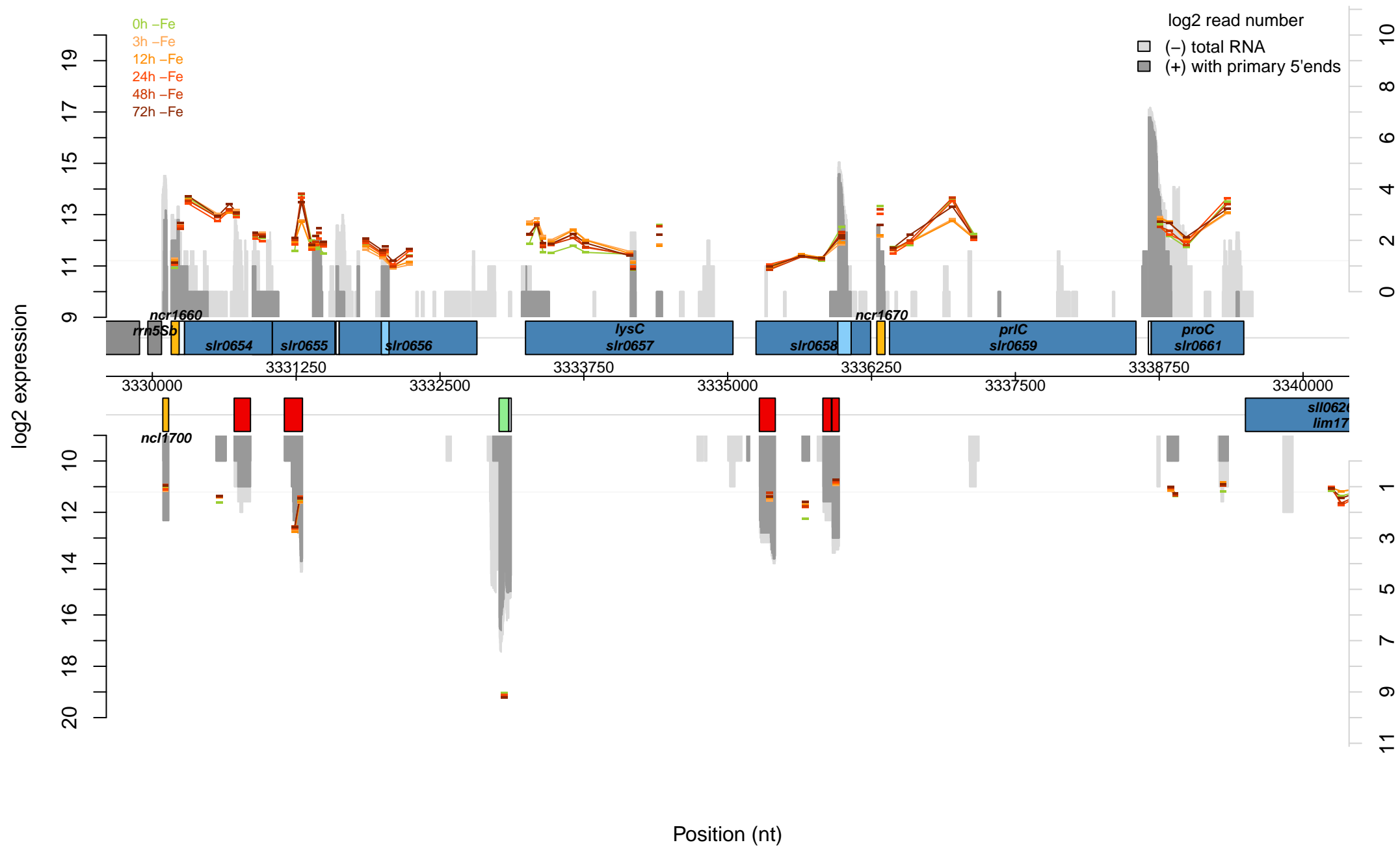

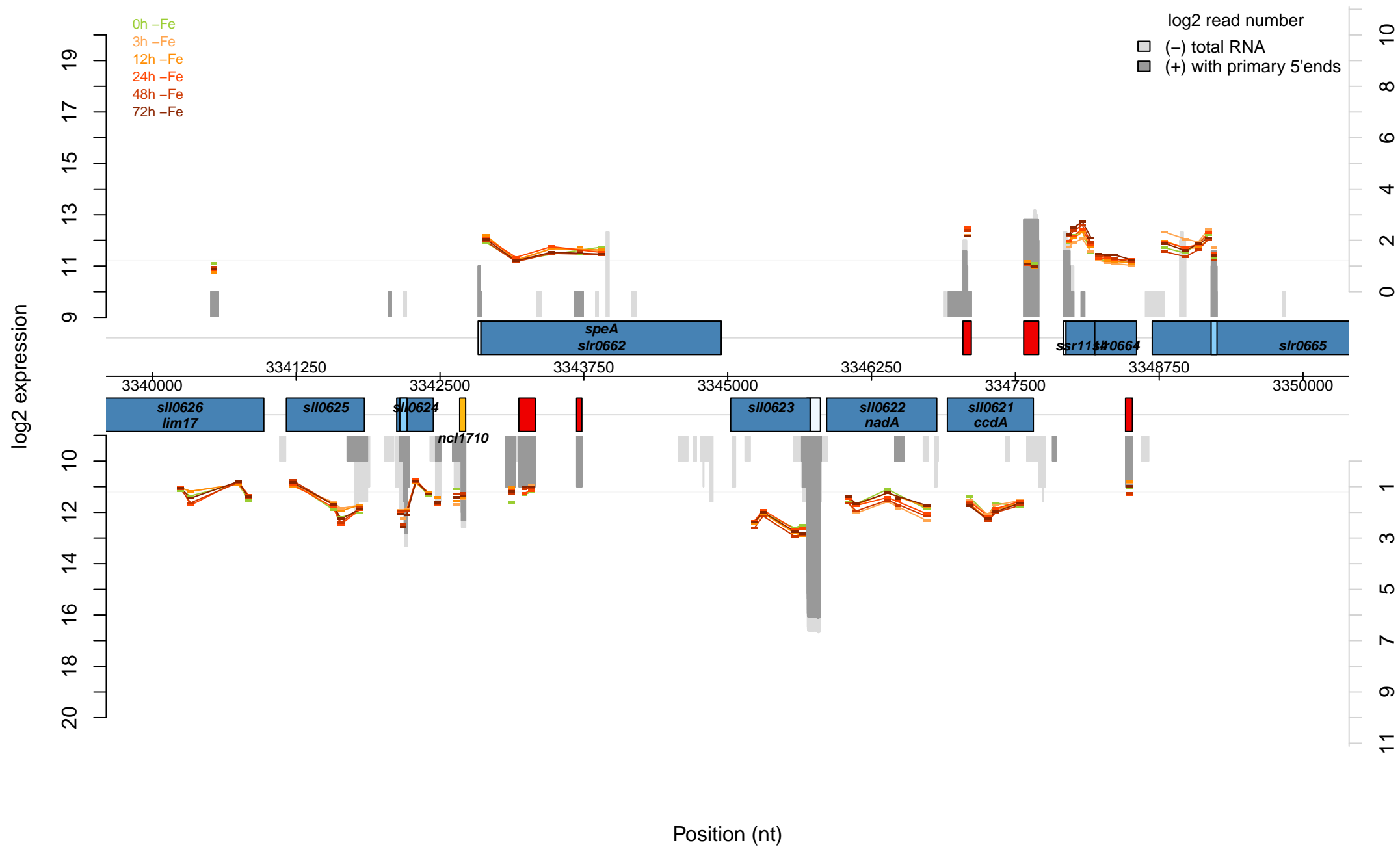

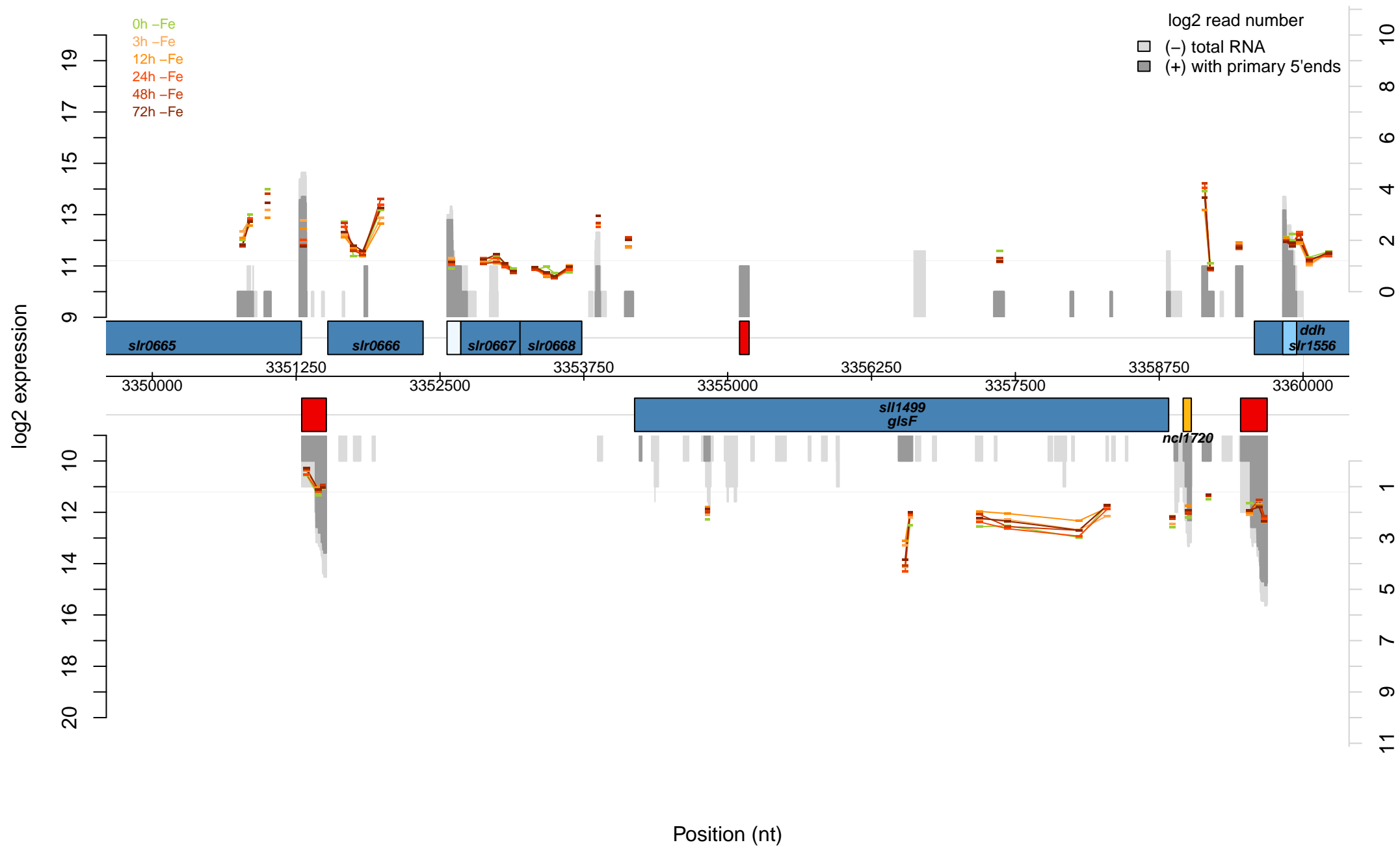

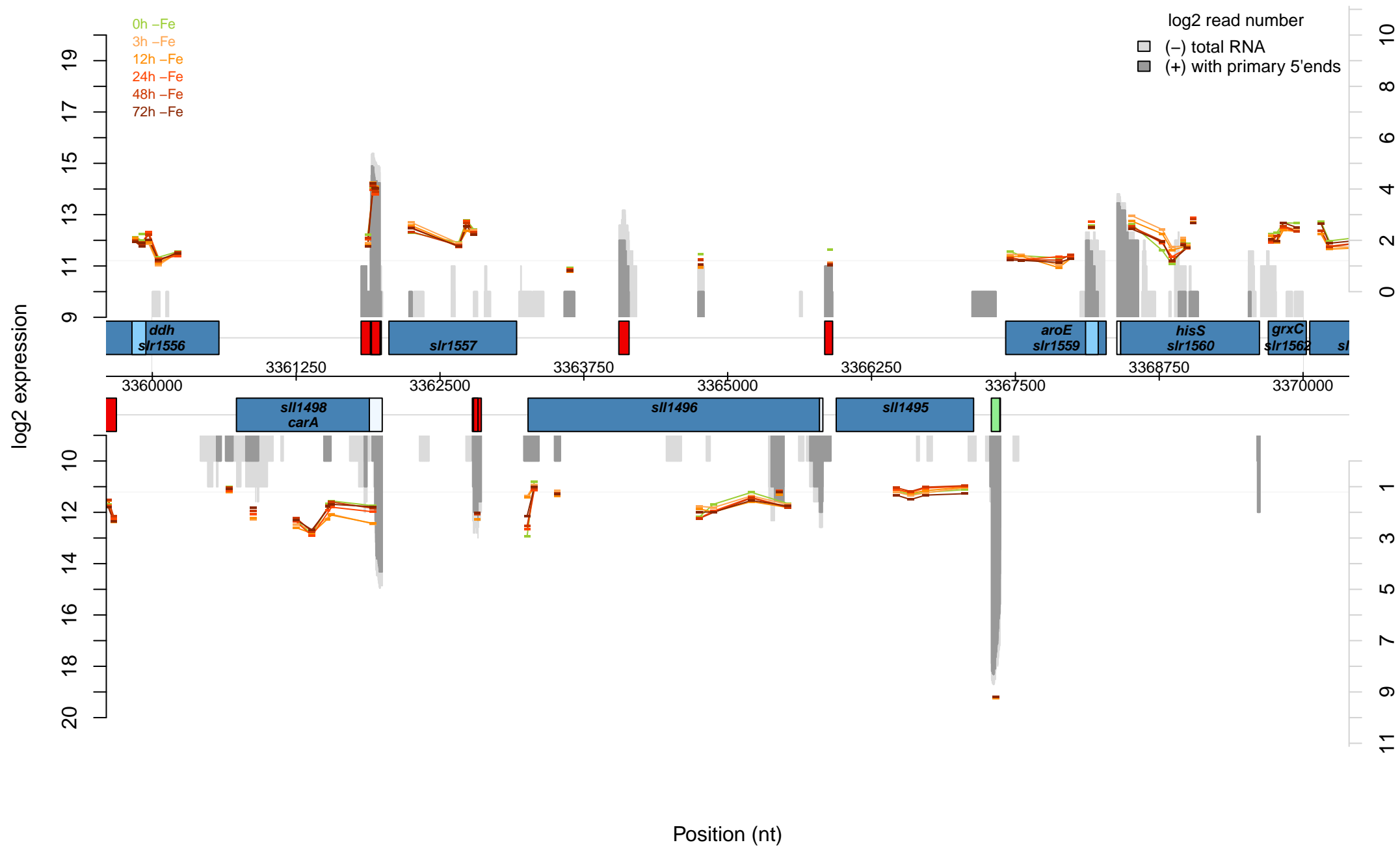

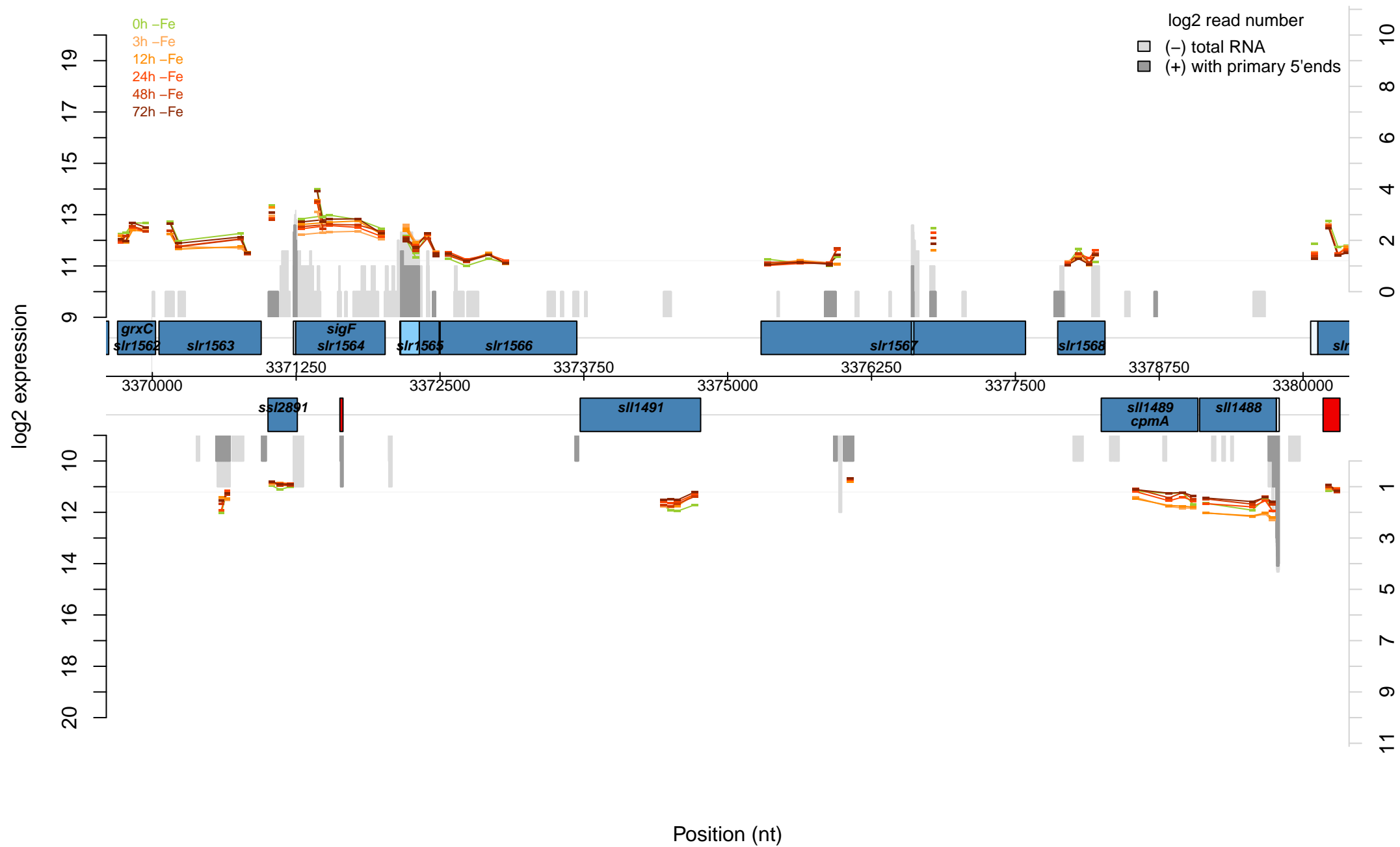

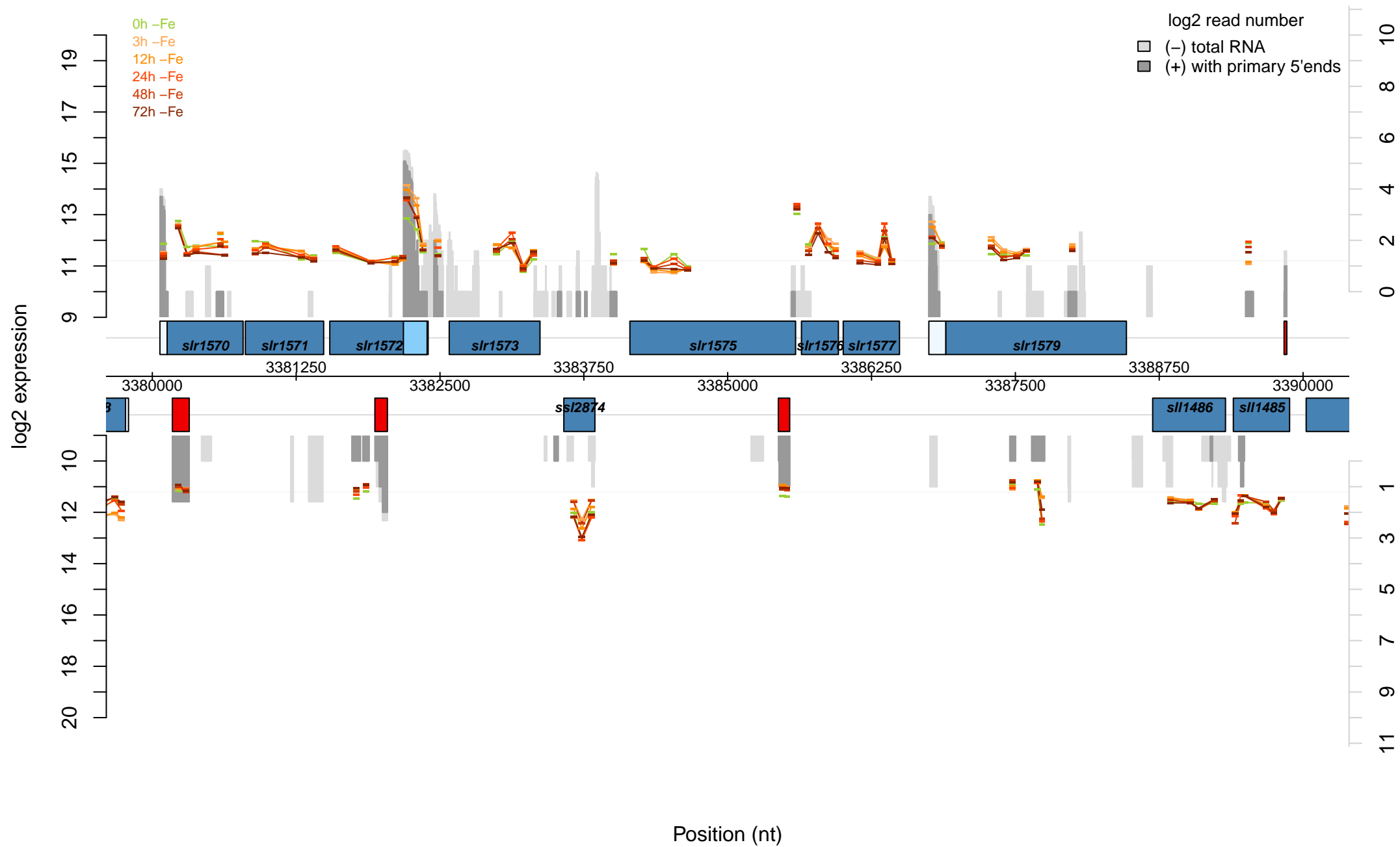

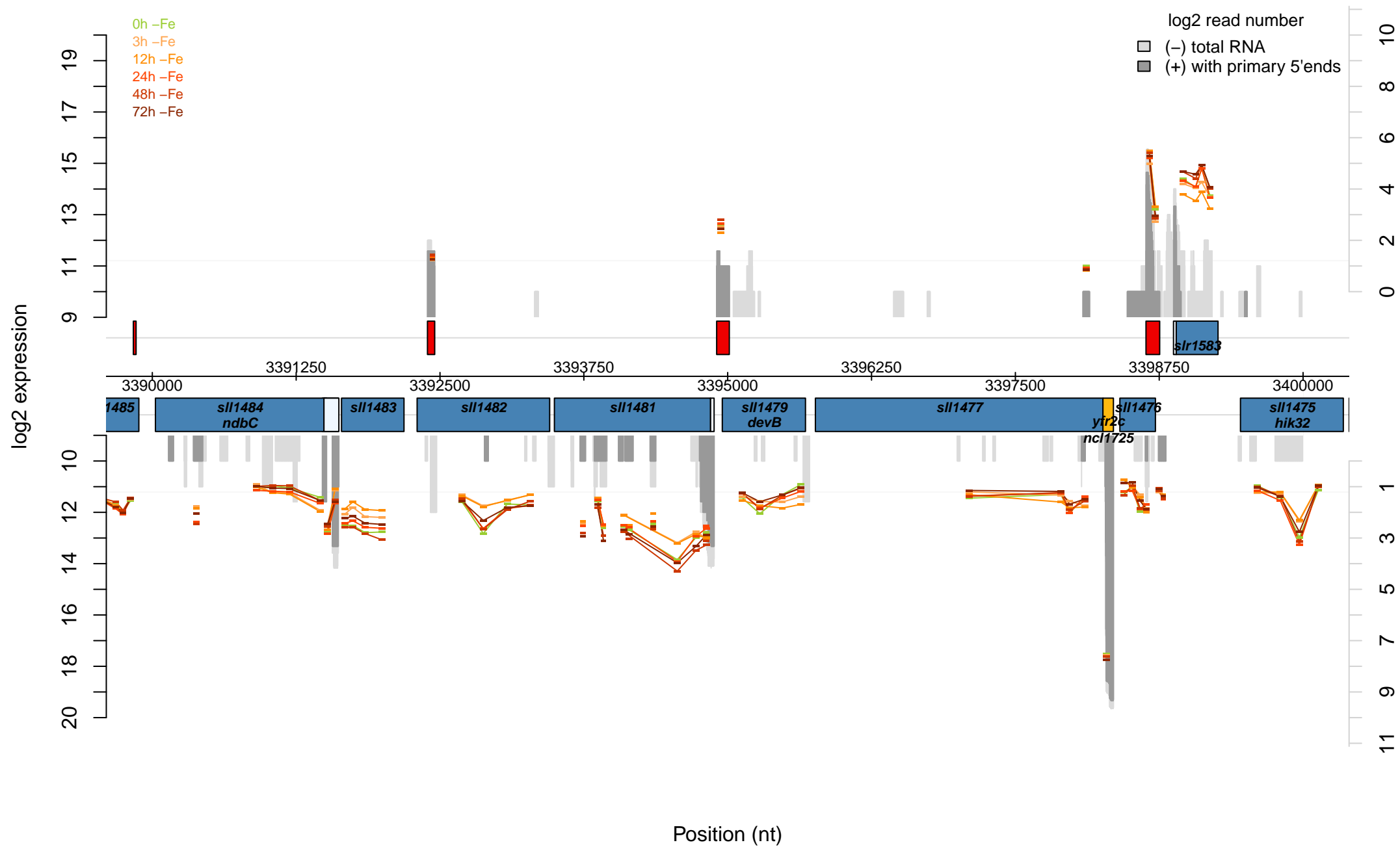

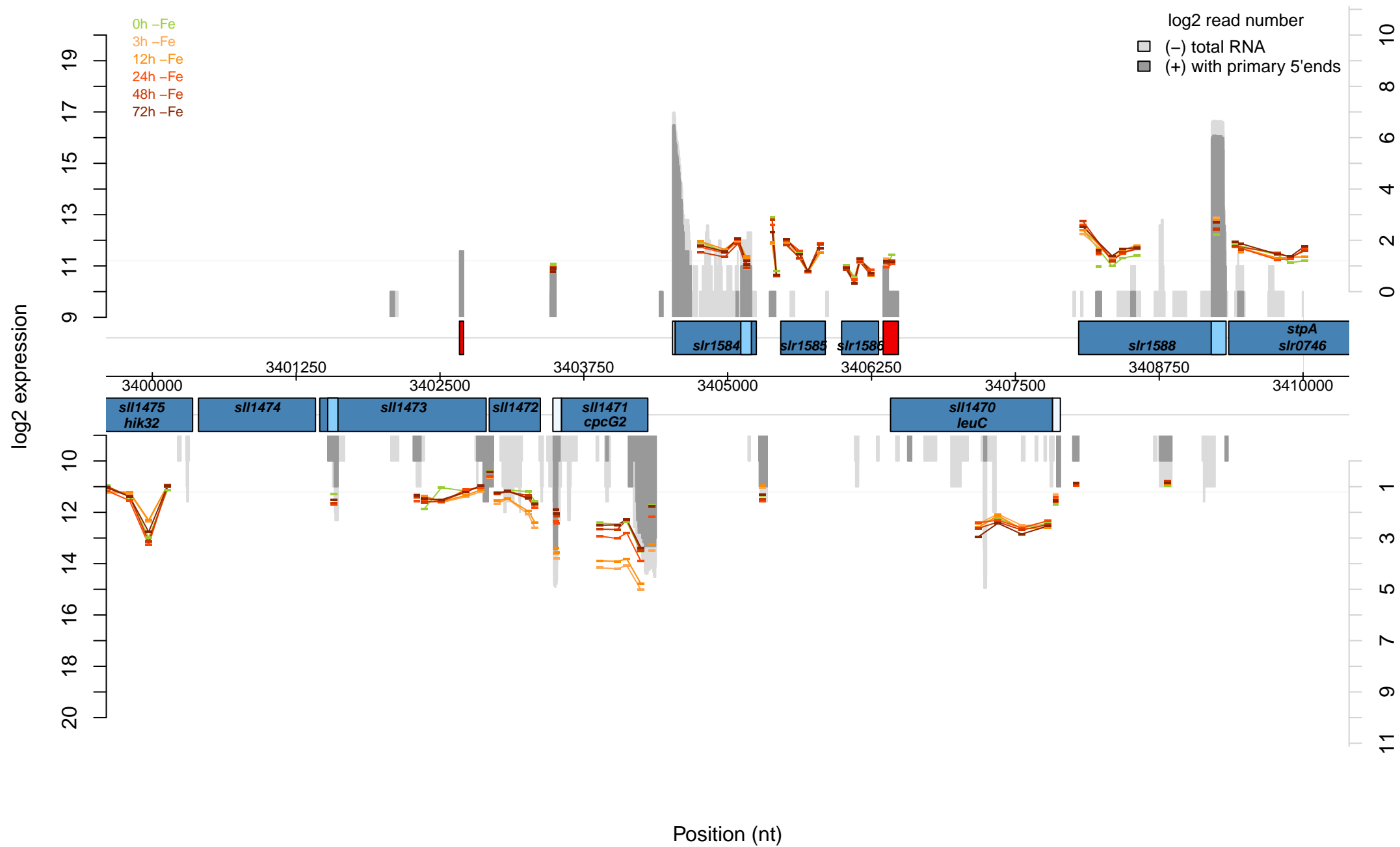

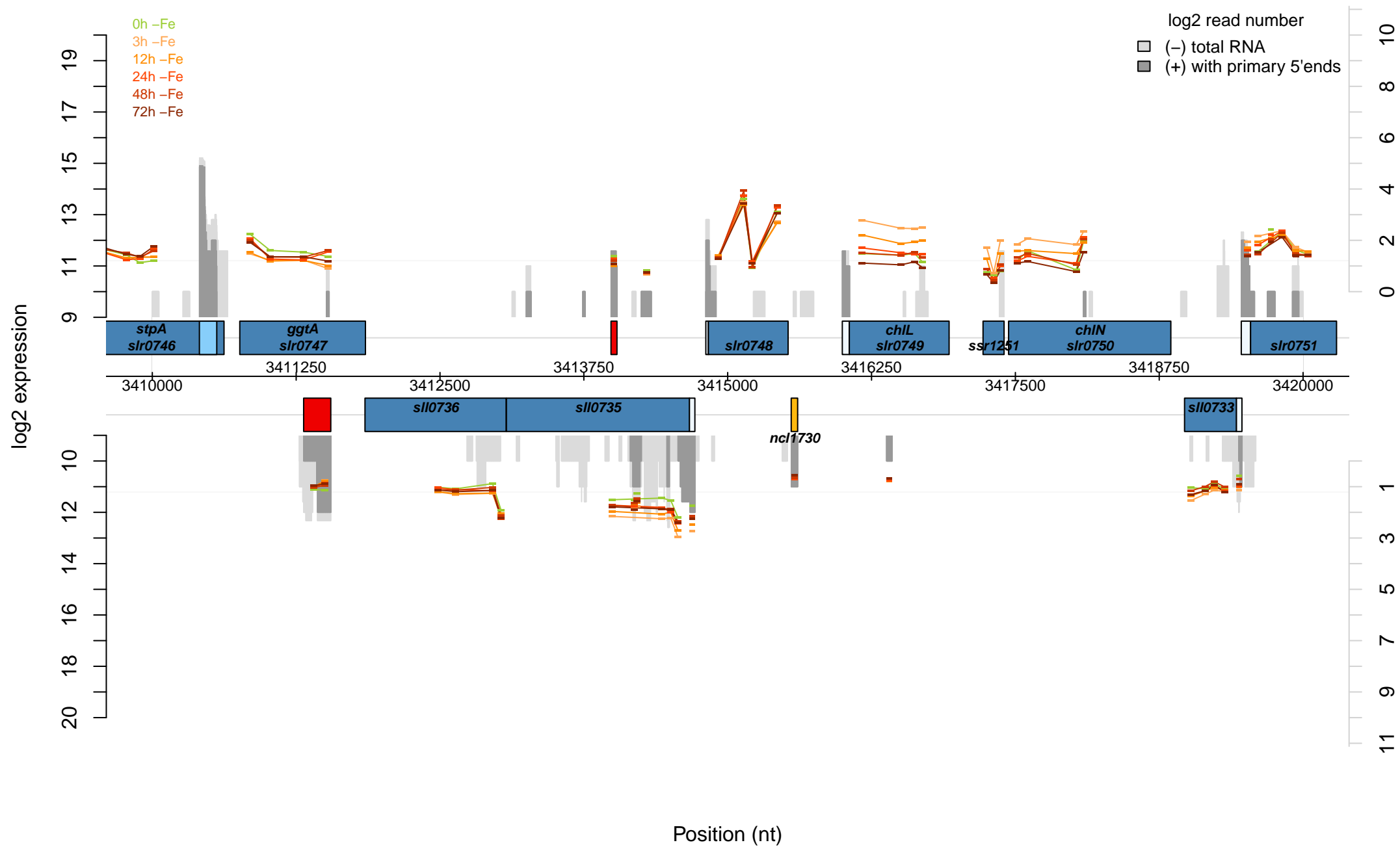

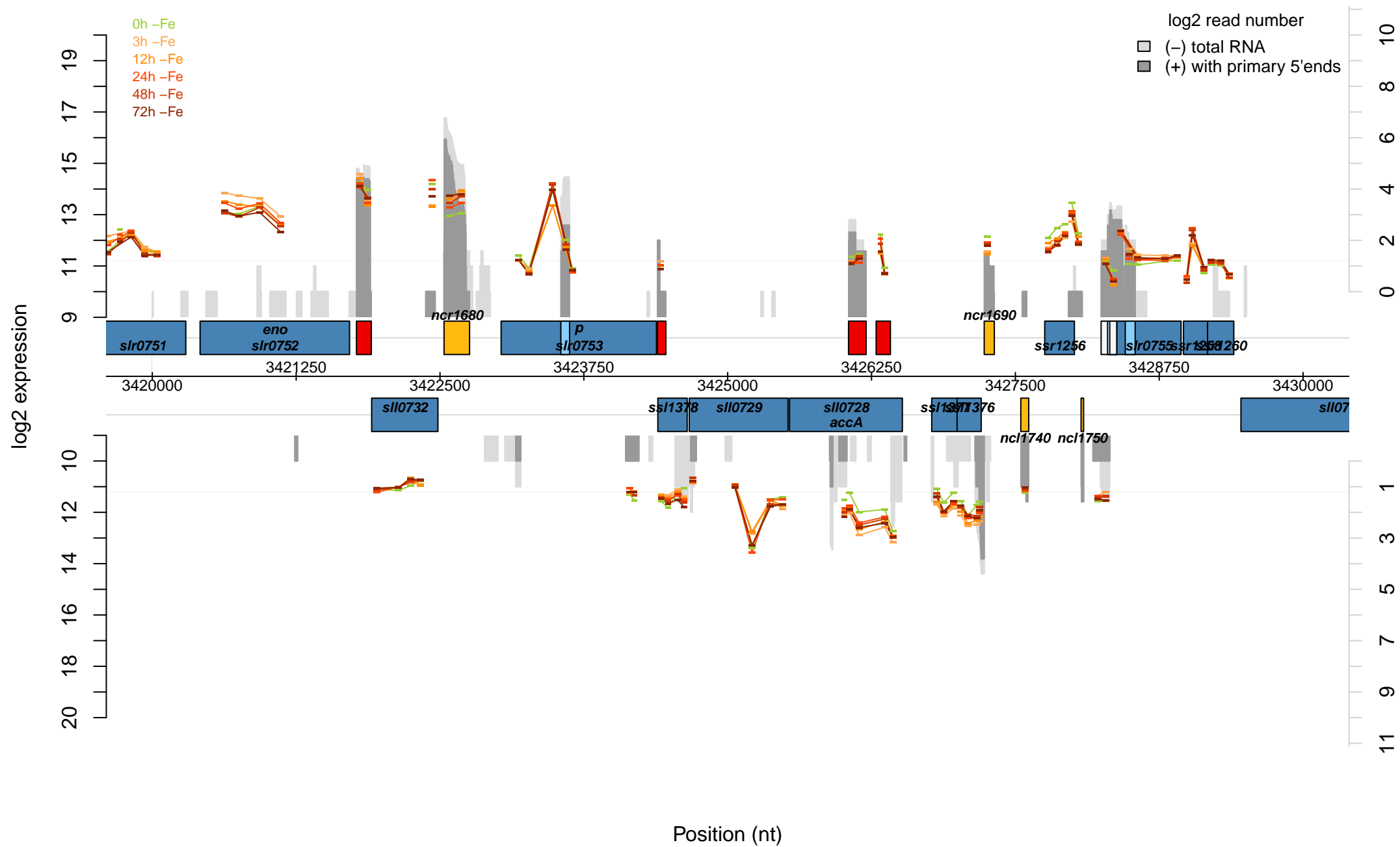

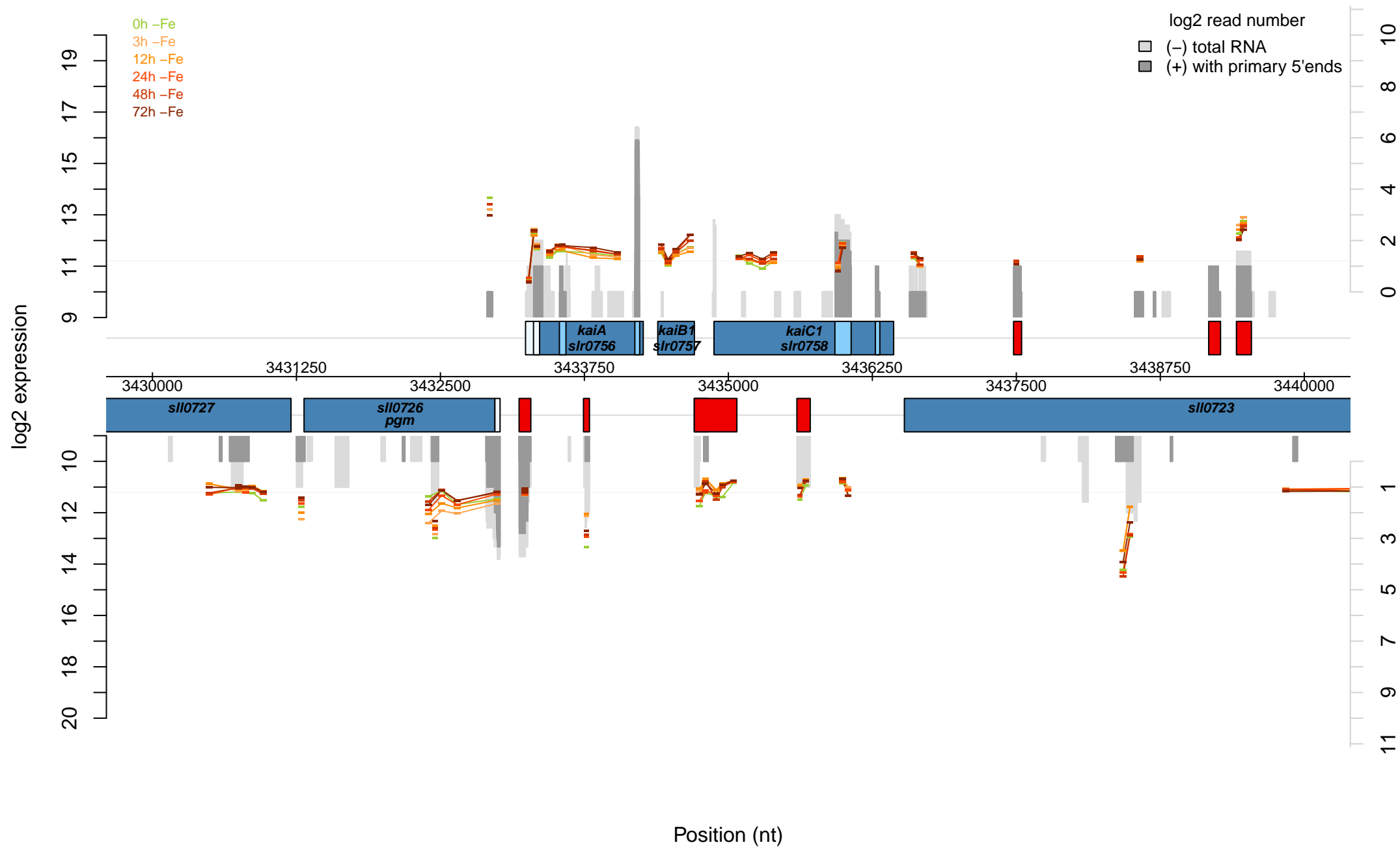

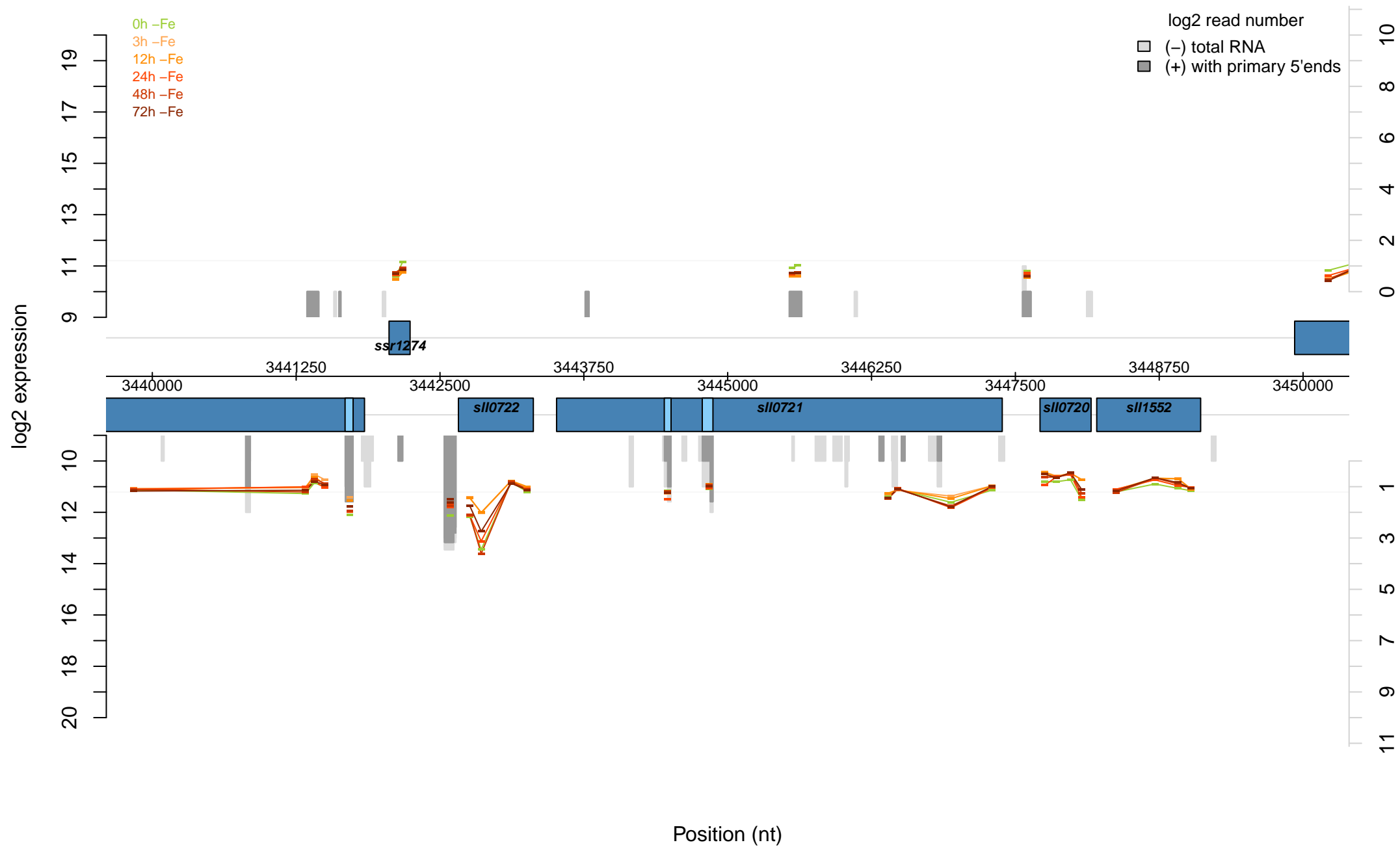

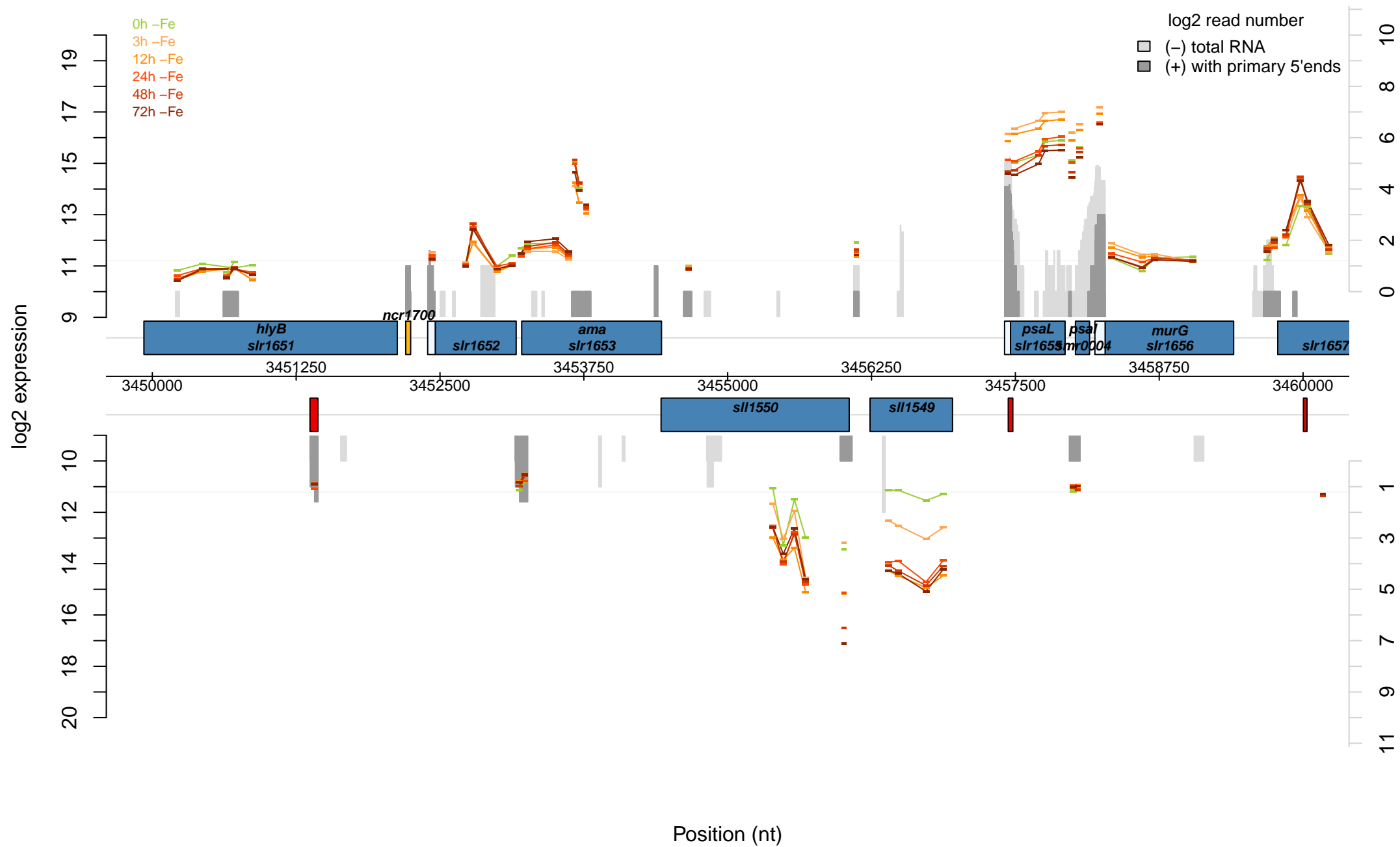

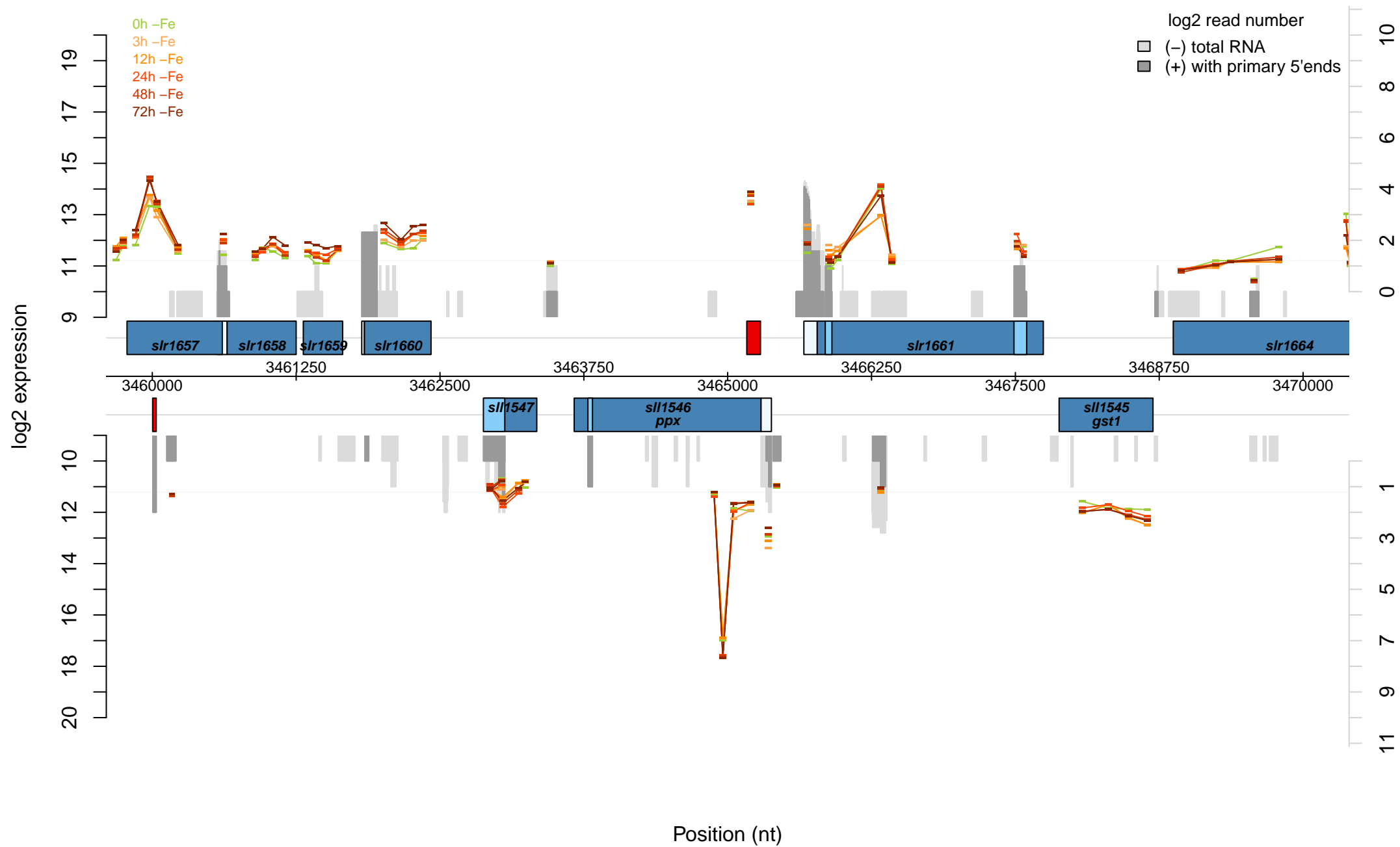

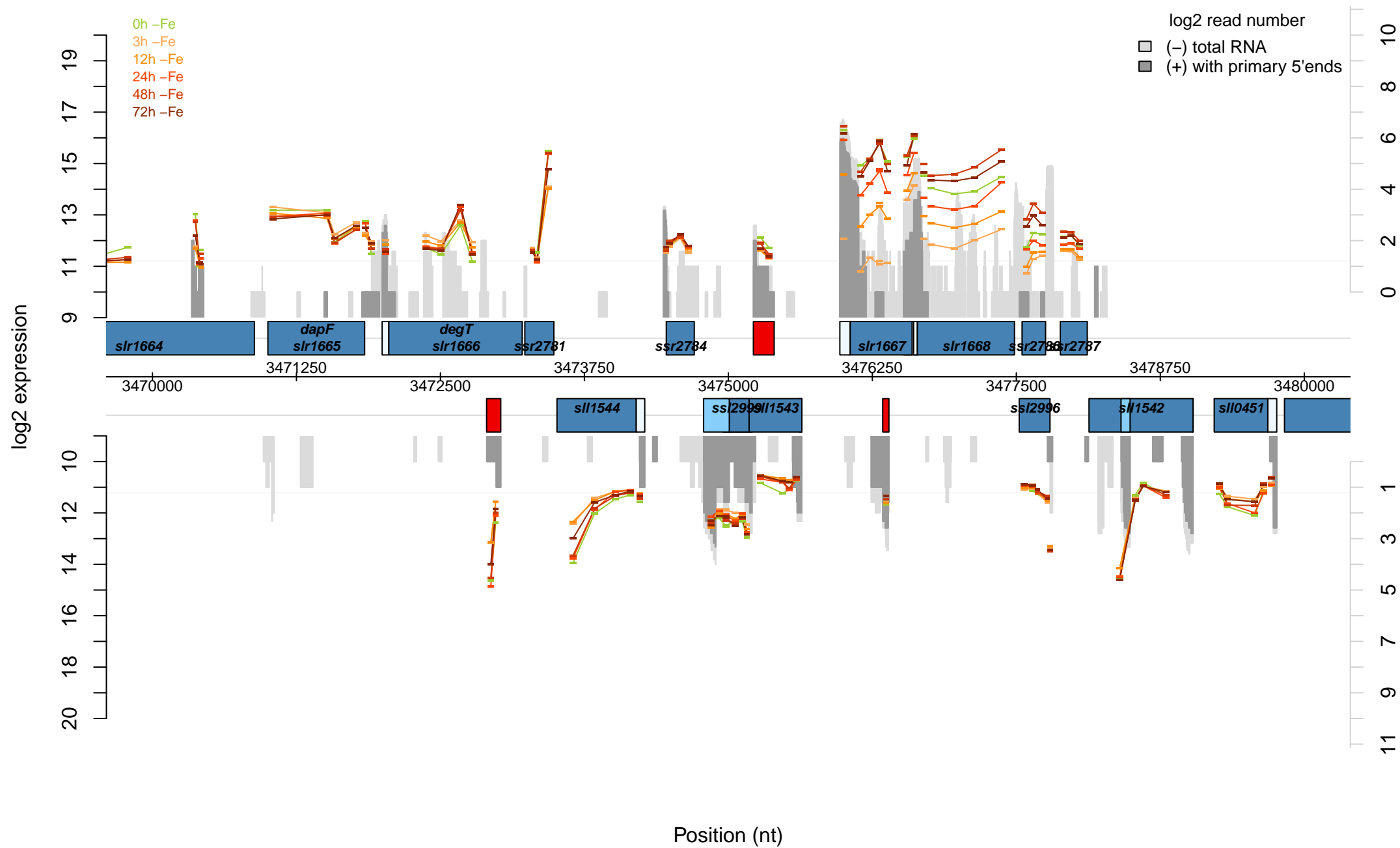

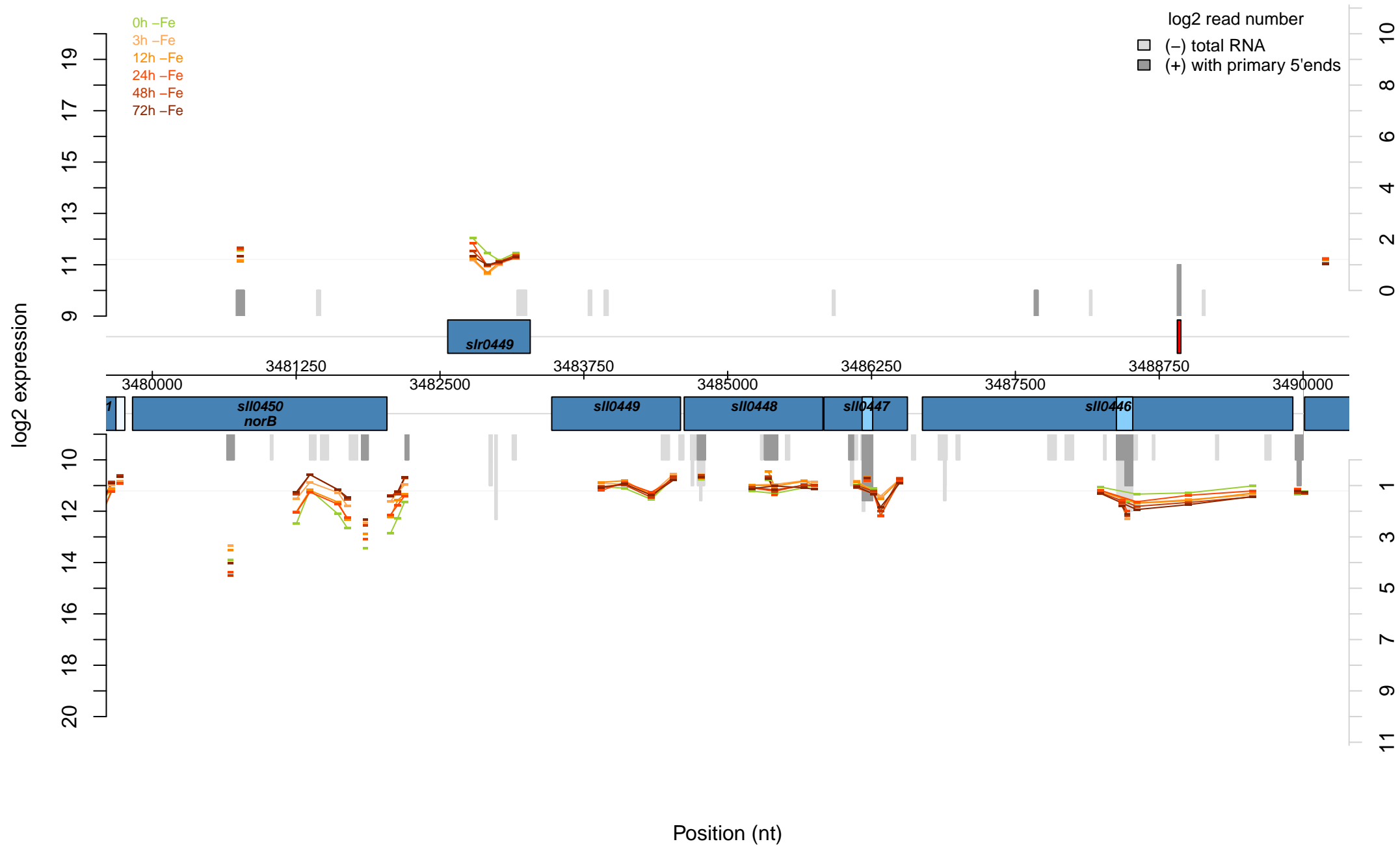

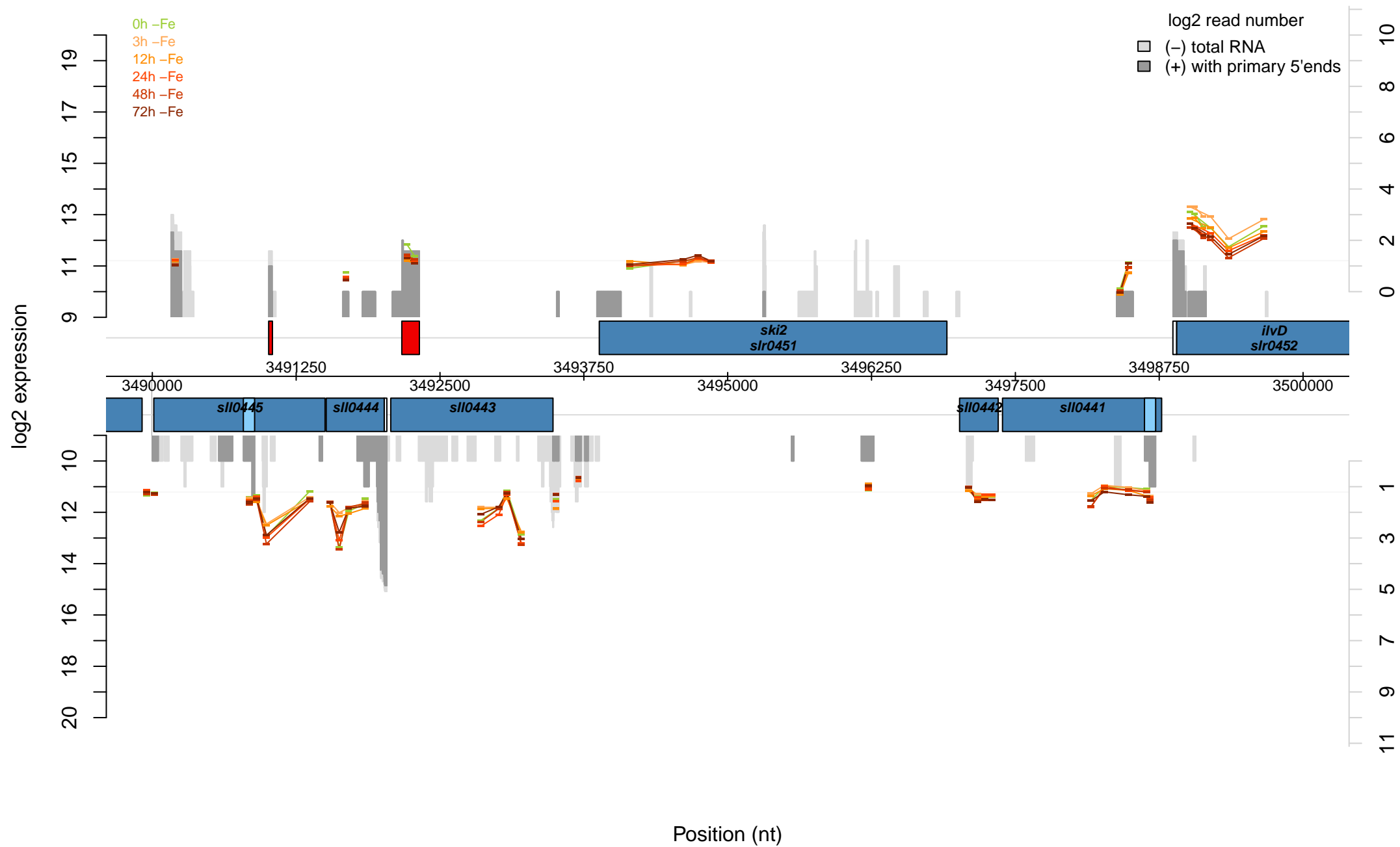

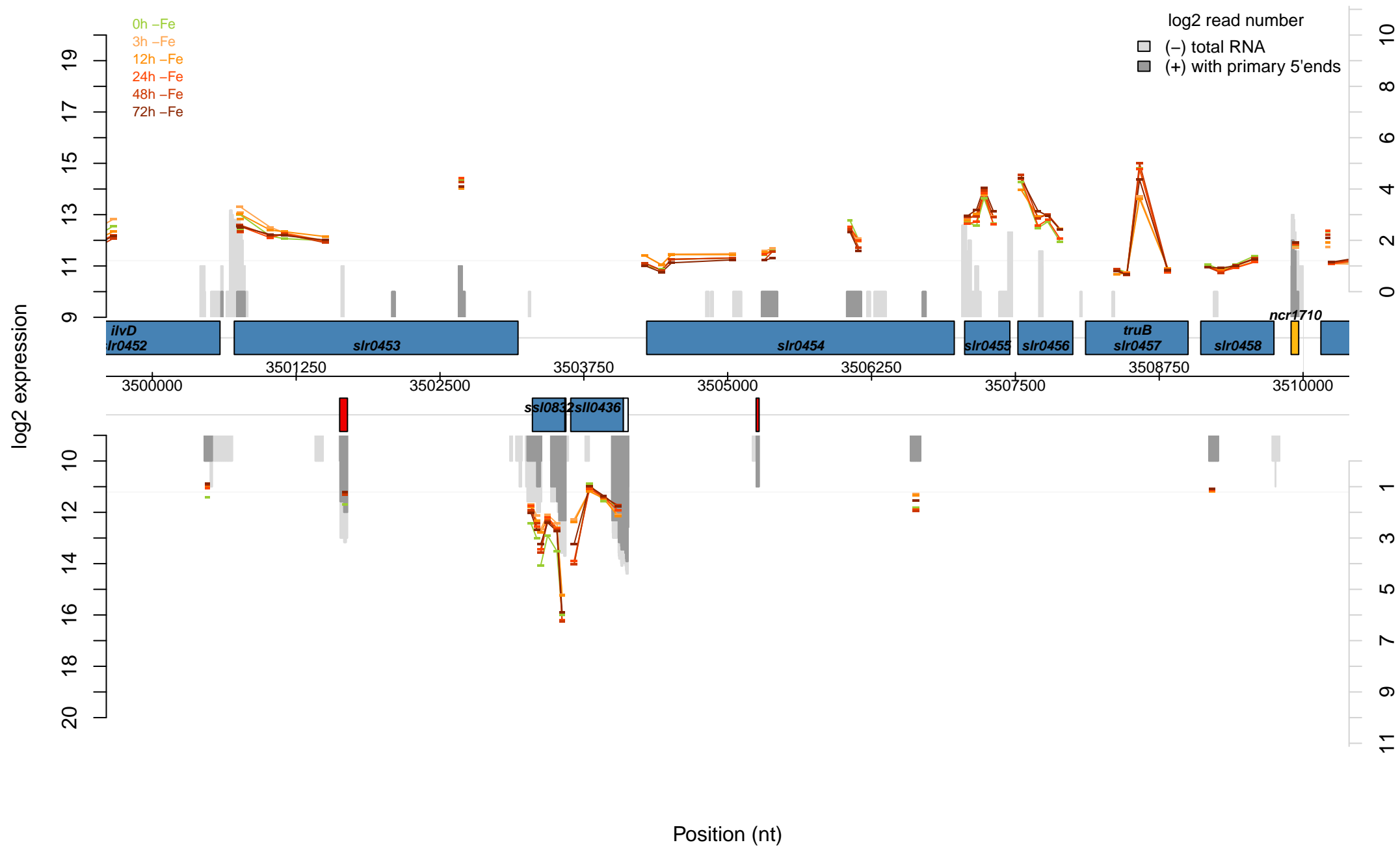

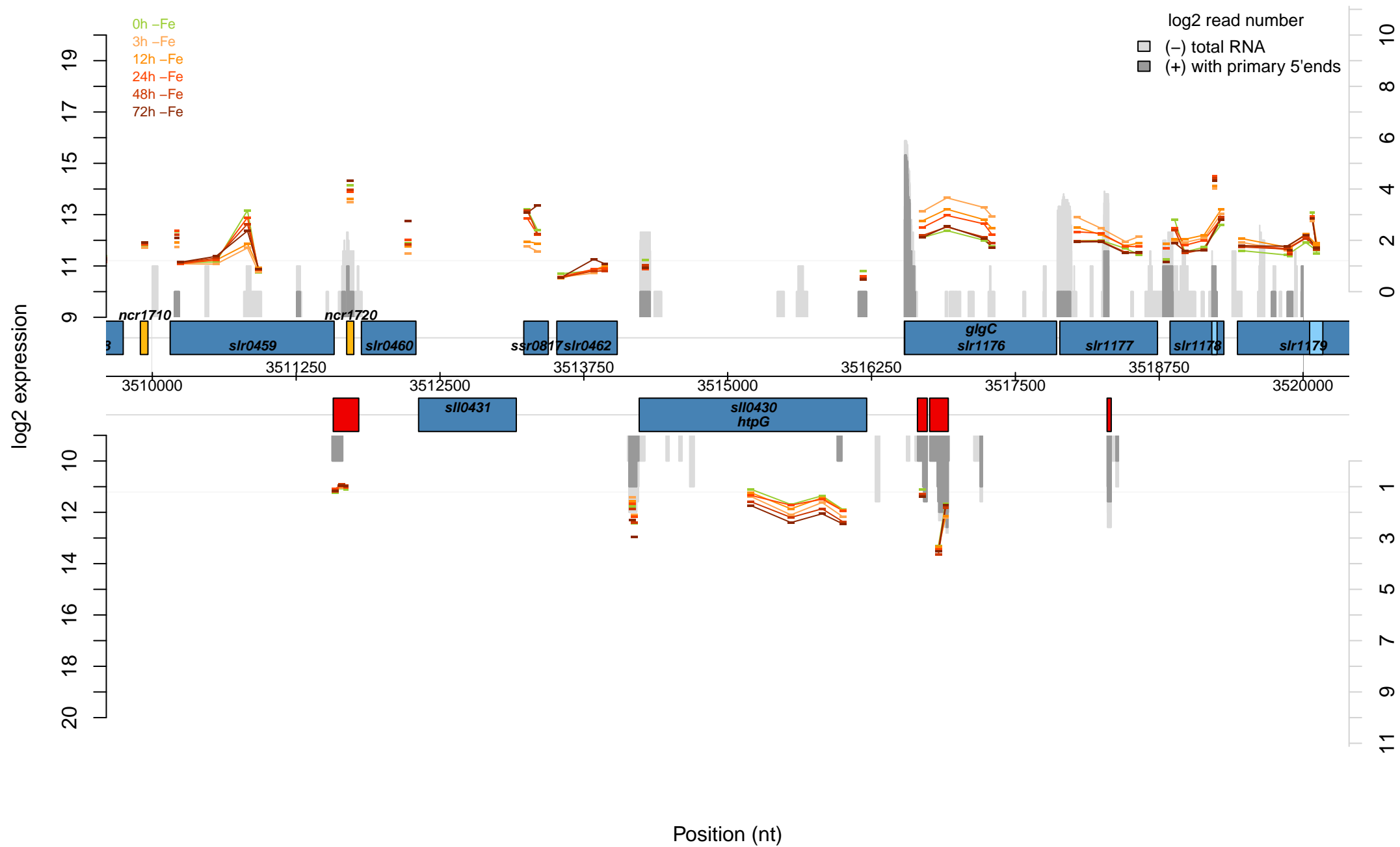

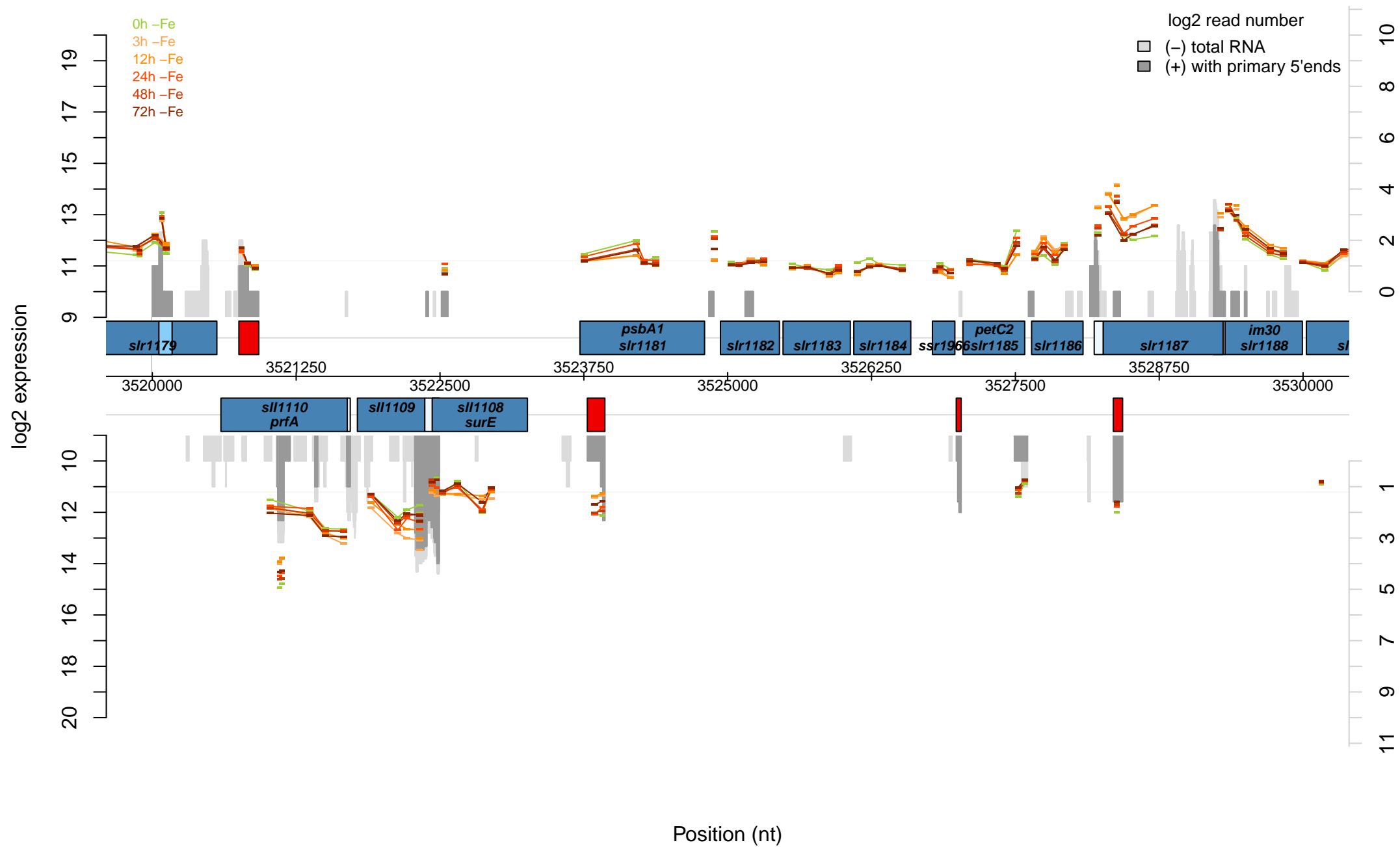



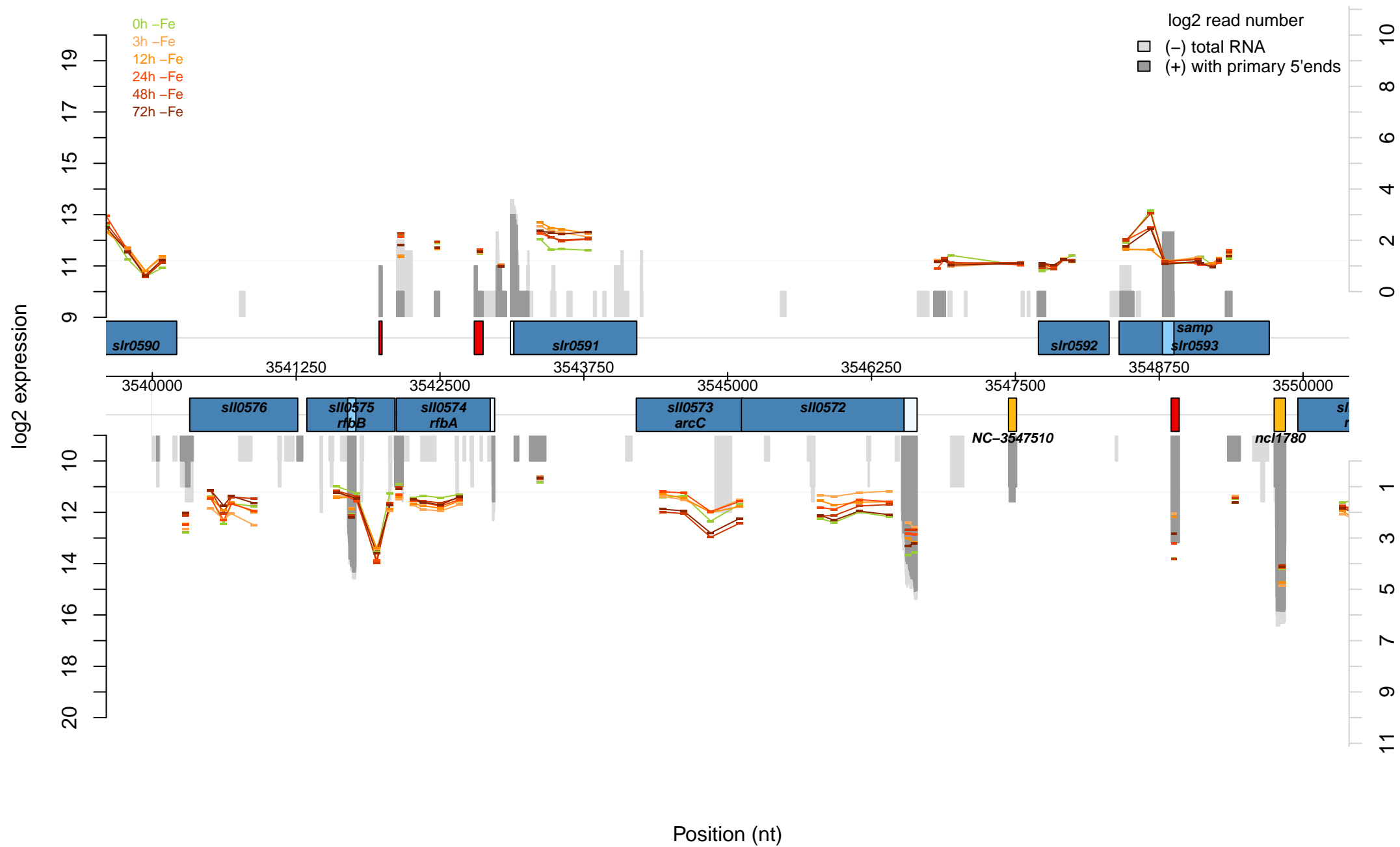

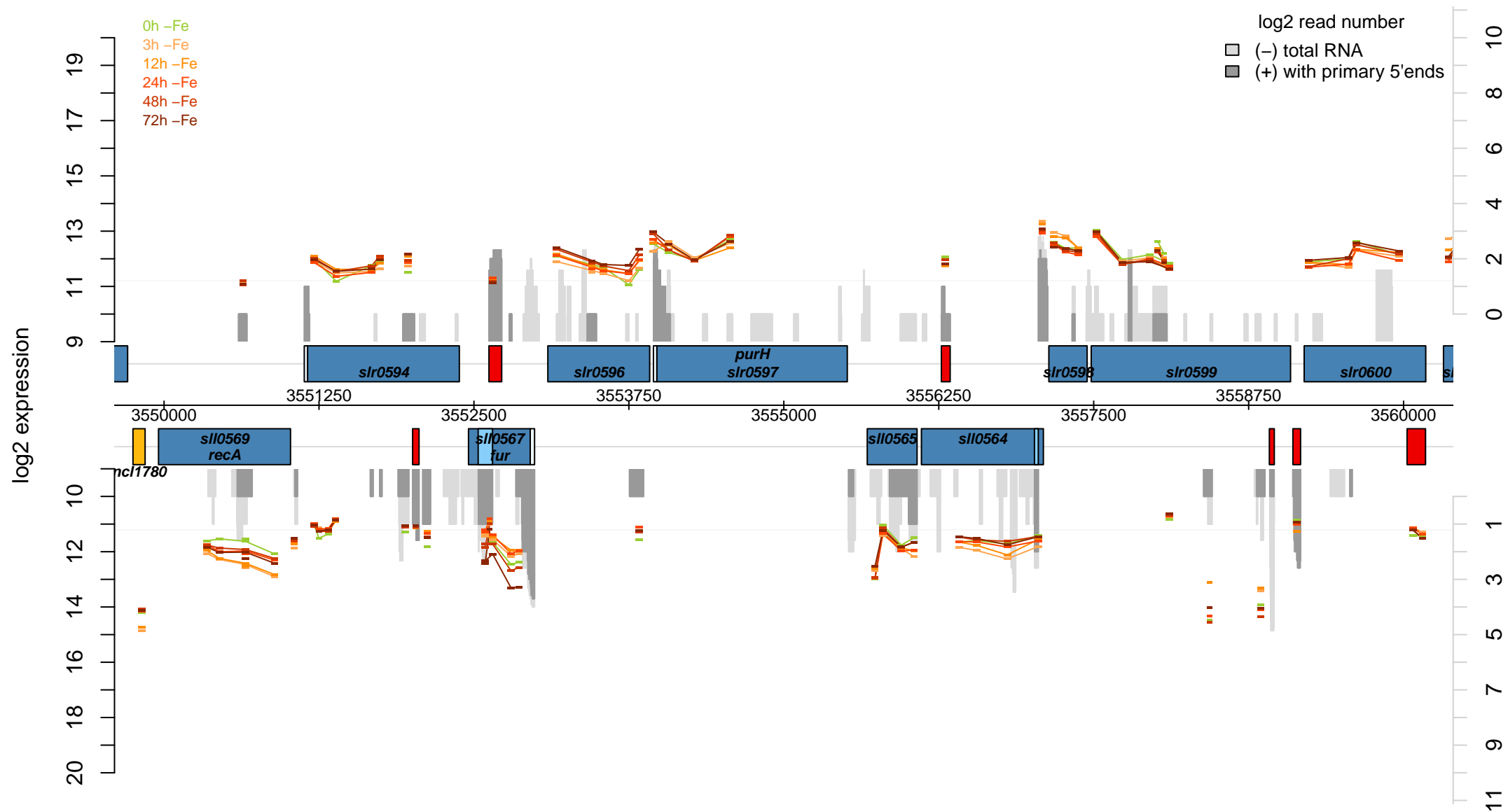

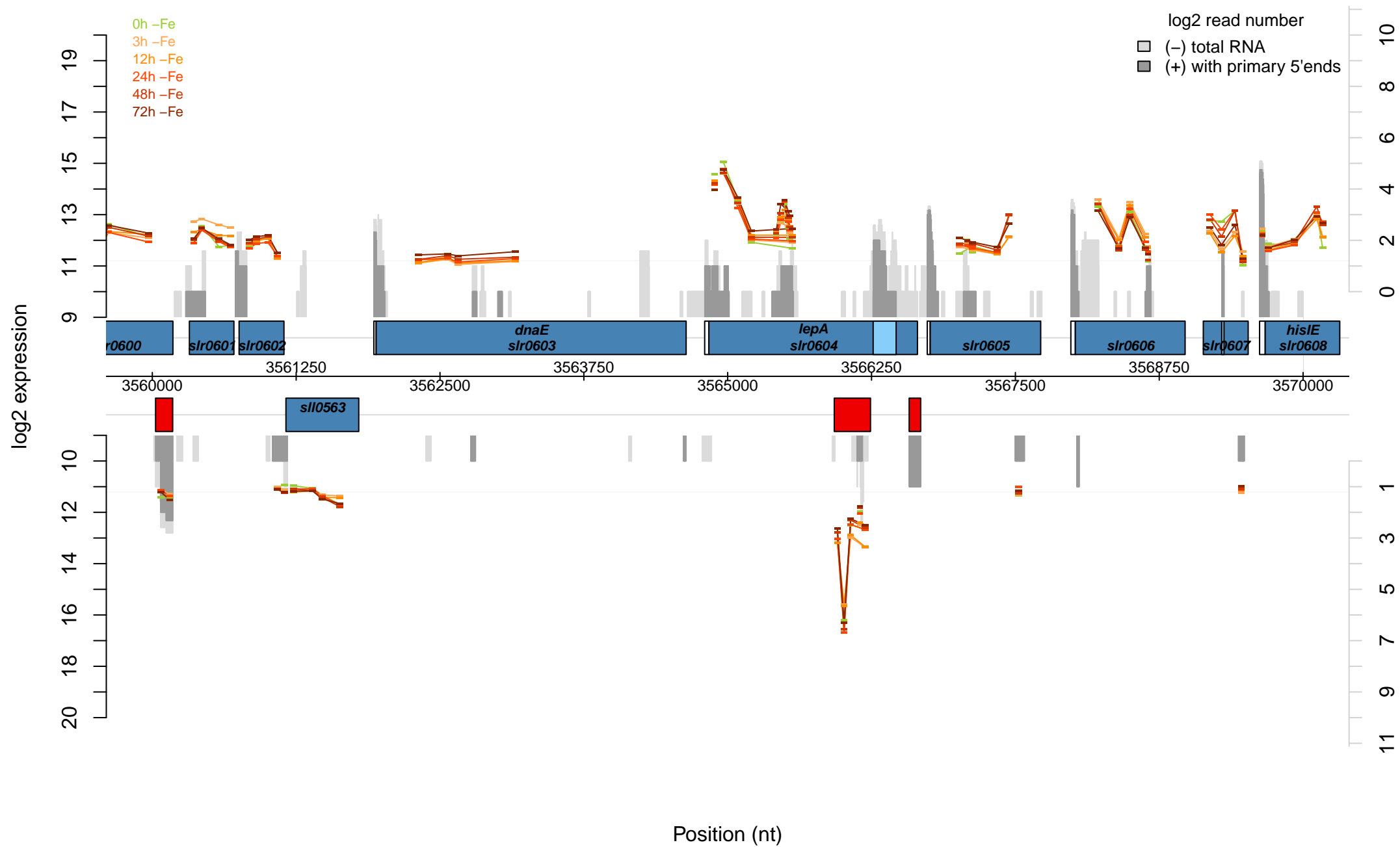

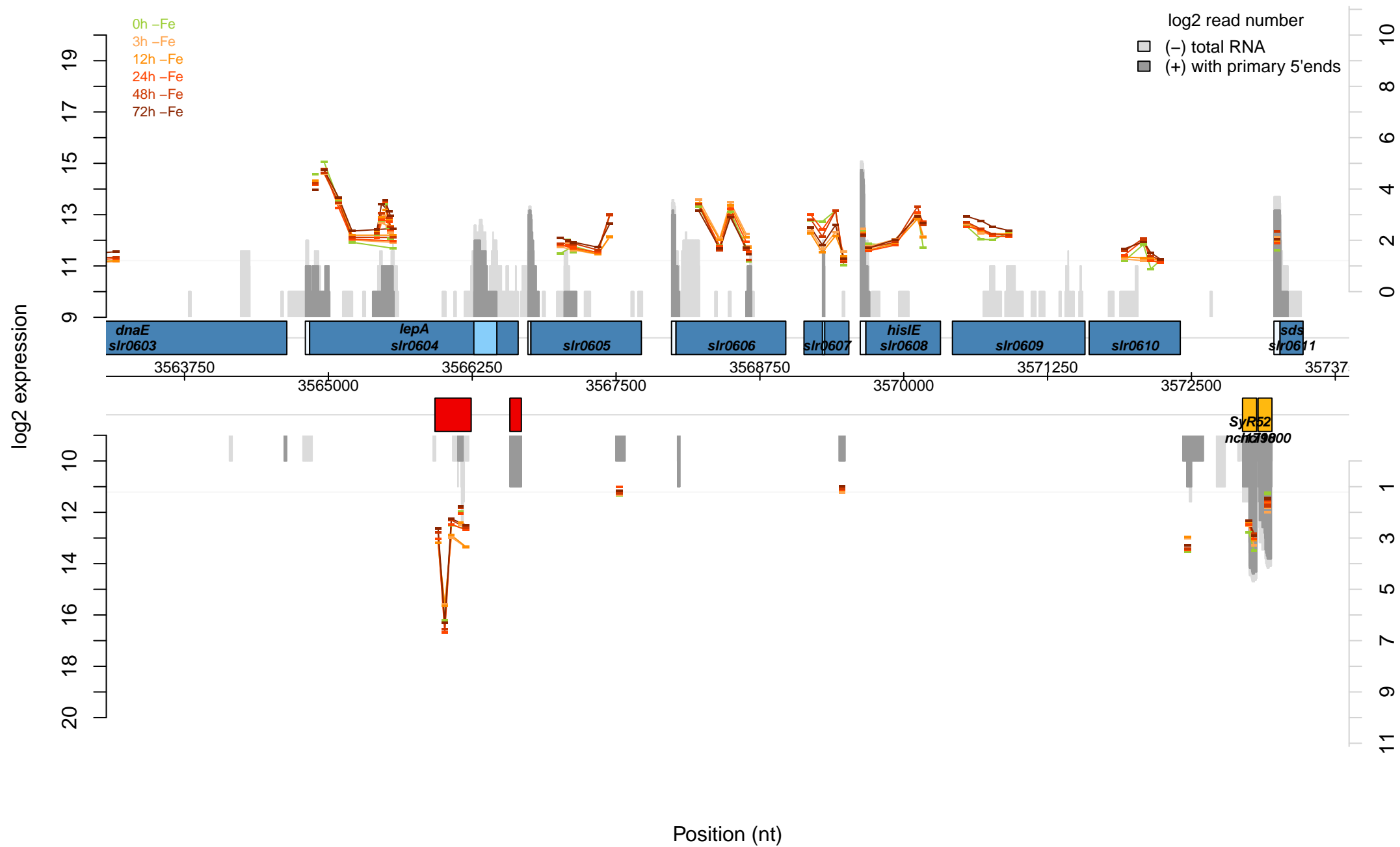

Supplement: Supporting Information [file supp_2.12.1475_FigureS4.pdf]
