# Supplementary material for: Iron Deprivation in Synechocystis: Inference of Pathways, Non-coding RNAs, and Regulatory Elements from Comprehensive Expression Profiling
Source: G3 (Bethesda). 2012 Dec 1;2(12):1475–95. doi: 10.1534/g3.112.003863 (PMC3516471; doi:10.1534/g3.112.003863)
Supplement: Supporting Information [file supp_2.12.1475_FigureS5.pdf]

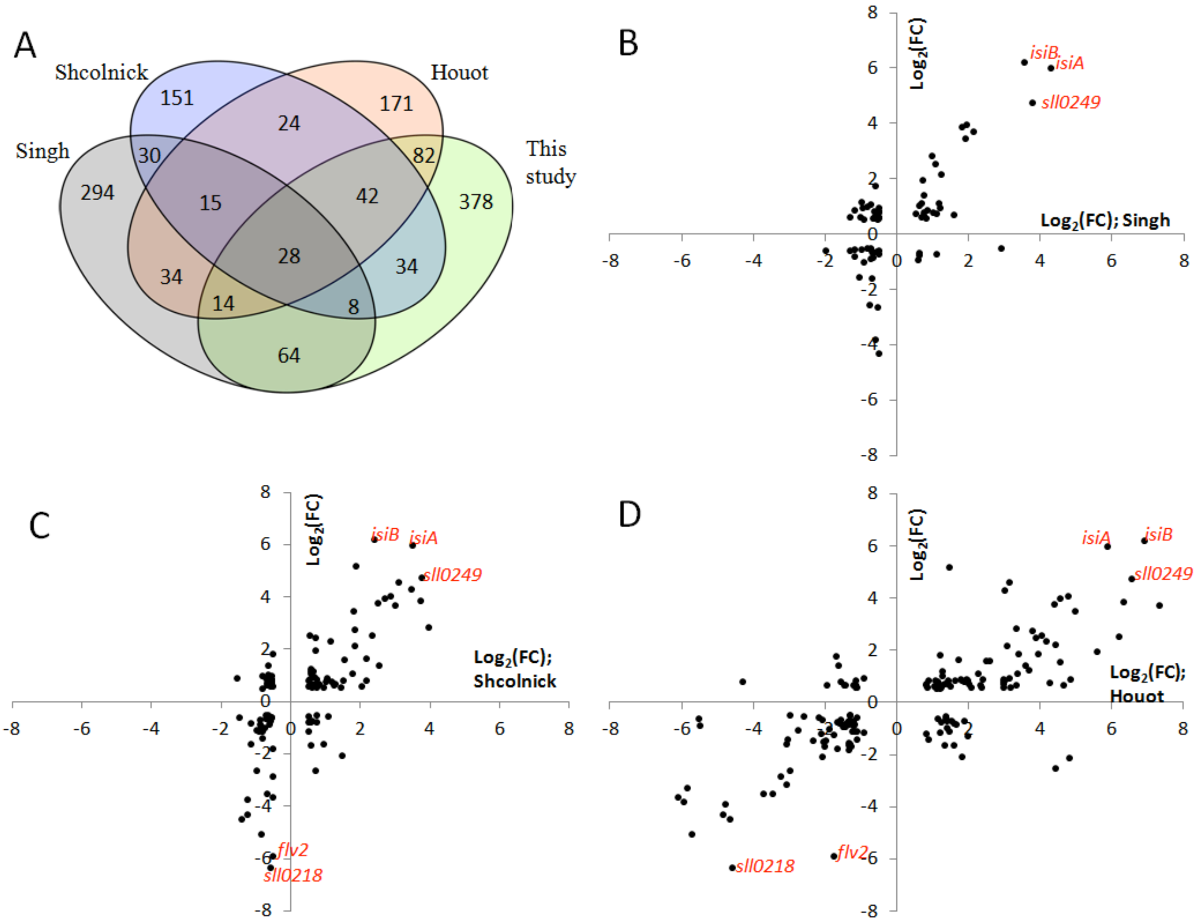

Figure S5 Comparision of microarray experiments. (A) Venn diagram that display the number of protein-coding genes differentially expressed (absolute log2(FC) ≥ 0.5, p value < 0.05) in the compared experiments. Numbers in the overlapping areas indicate genes that exhibited differential expression in either two, three or four of the studies compared. (B-D) The expression changes between samples grown in iron repleted and depleted conditions obtained in each of the compared experiments were plotted in the form of log2(FC) against the median log2(FC) obtained in our analysis. Key genes in the iron stress response, such as isiA and isiB, and genes encoding the flavodiiron protein (flv2) and the cotranscribed sll0218, which undergo down regulation upon DFB addition, are indicated in red.
