# Supplementary material for: Iron Deprivation in Synechocystis: Inference of Pathways, Non-coding RNAs, and Regulatory Elements from Comprehensive Expression Profiling
Source: G3 (Bethesda). 2012 Dec 1;2(12):1475–95. doi: 10.1534/g3.112.003863 (PMC3516471; doi:10.1534/g3.112.003863)
Supplement: Supporting Information [file supp_2.12.1475_FigureS6.pdf]

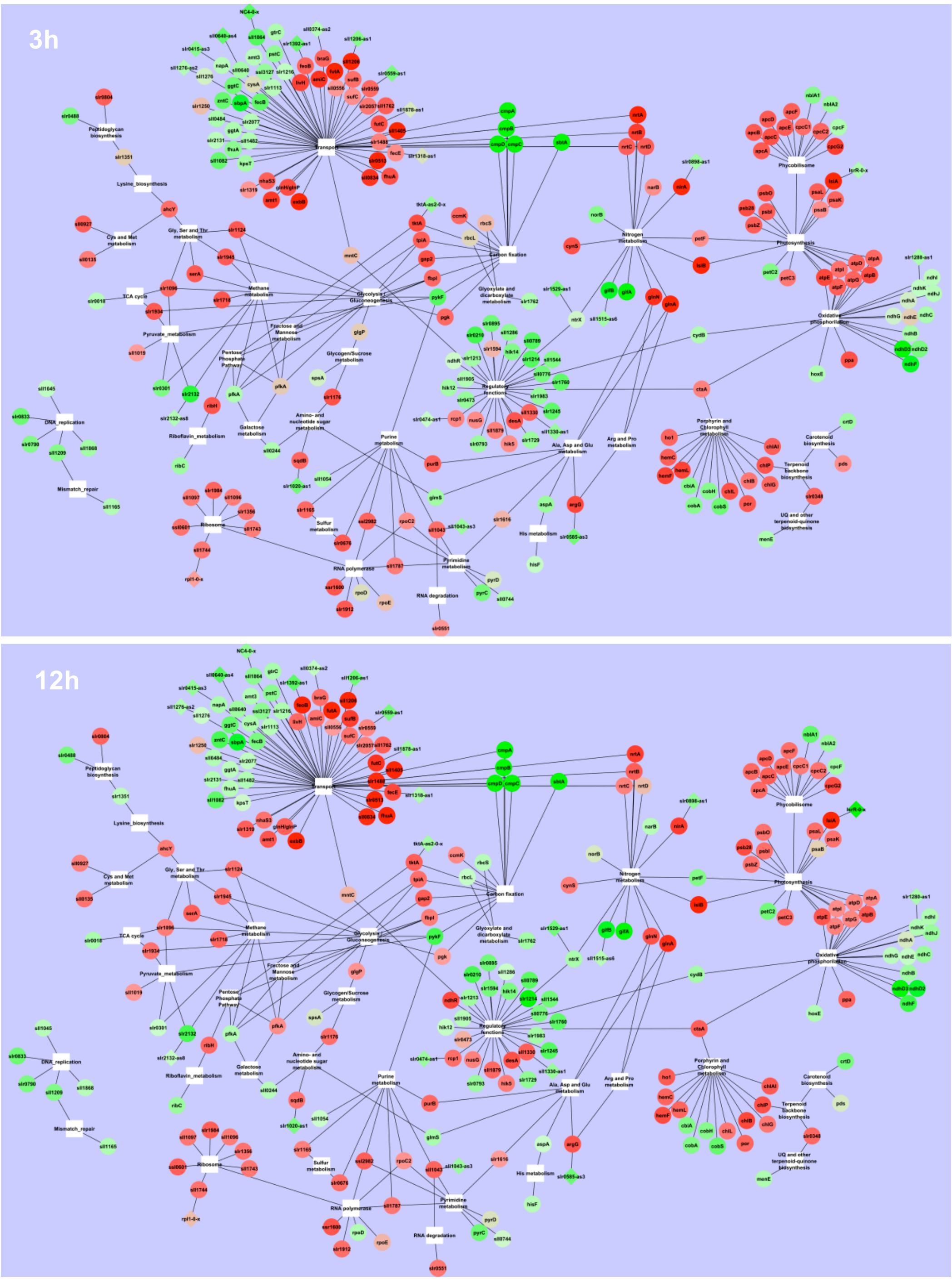
3h
12h

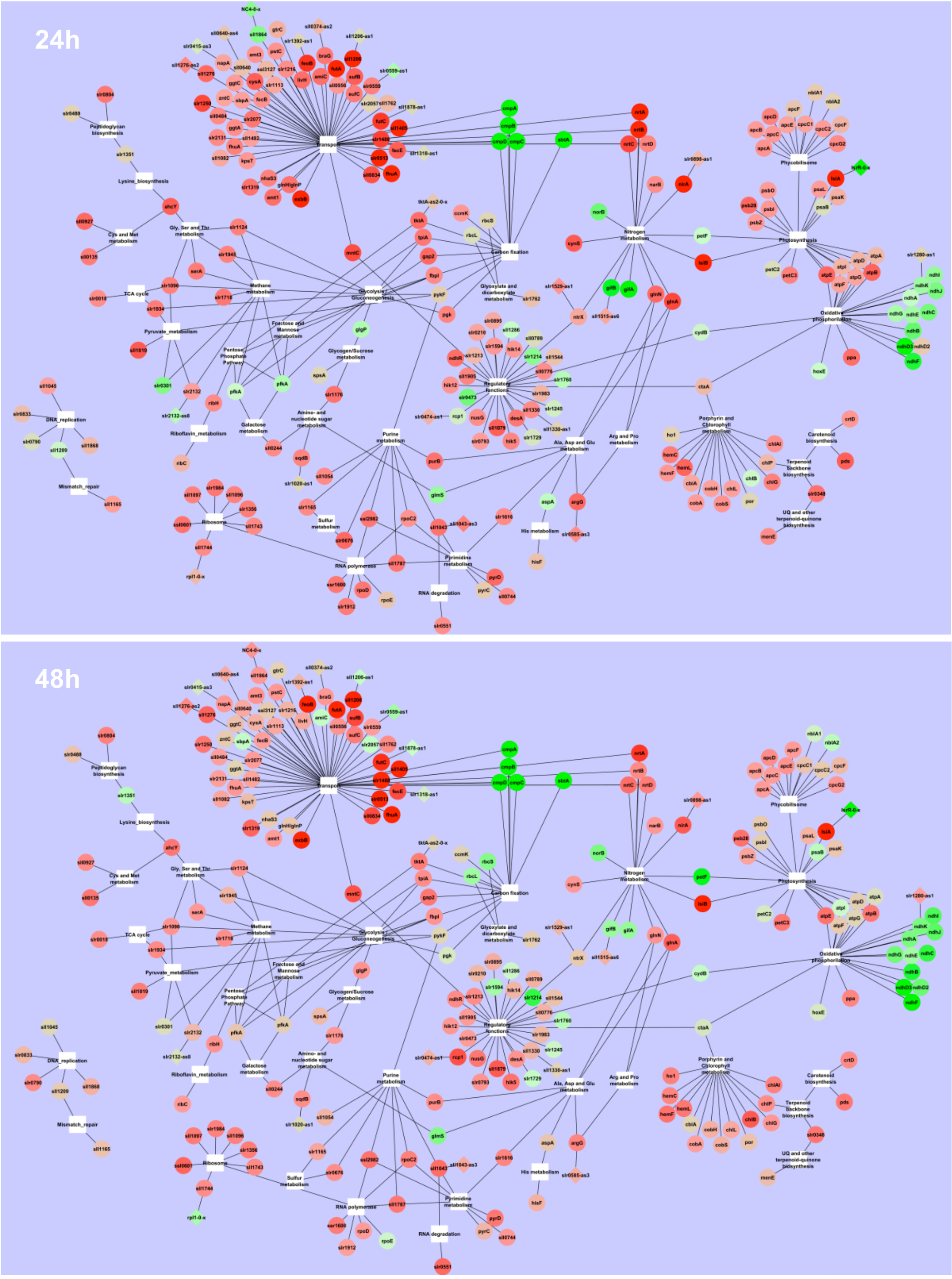
24h
48h

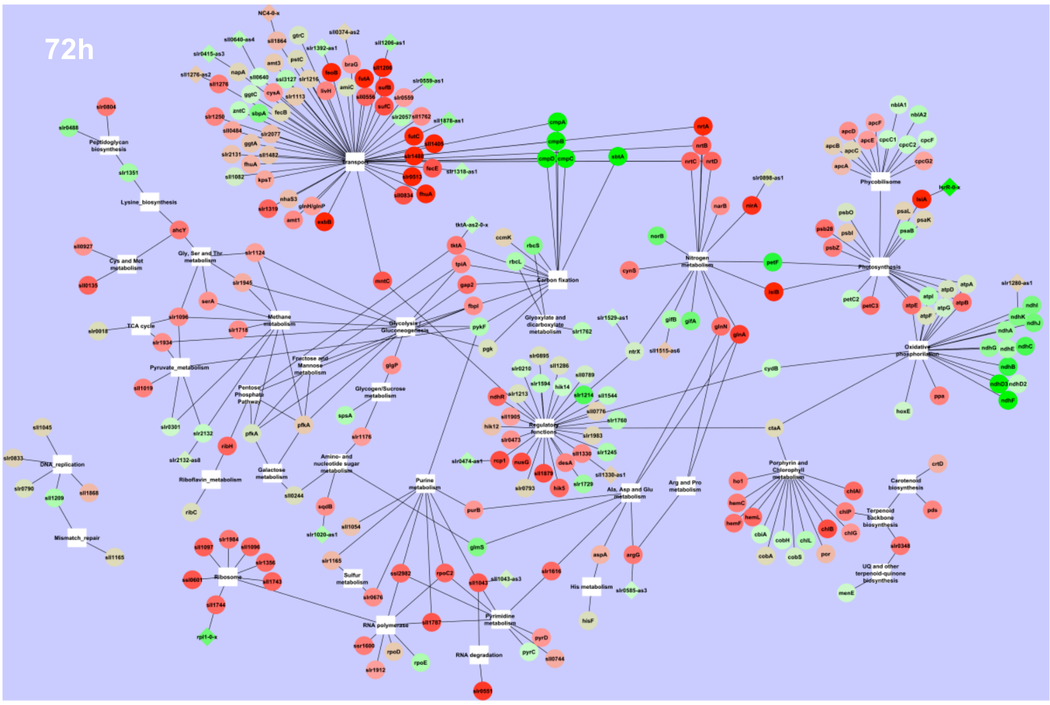

Figure S6 Functional networks based on KEGG pathways. Protein-coding genes (represented by circular nodes), which were differentially expressed under iron limiting conditions, are linked to their corresponding KEGG pathway (square nodes). Furthermore, differentially expressed asRNAs (diamond nodes) are linked to their complementary protein-coding genes. Genes with functional roles in more than one pathway are linked to all of them (such as glnA and glnN, which are key elements of three KEGG pathways “Nitrogen metabolism”, “Arginine and proline metabolism” and “Alanine, aspartate, and glutamate metabolism”). The magnitude of expression changes at different time points are color-coded with shades of red indicating induction and shades of green indicating repression.
