# Supplementary material for: Iron Deprivation in Synechocystis: Inference of Pathways, Non-coding RNAs, and Regulatory Elements from Comprehensive Expression Profiling
Source: G3 (Bethesda). 2012 Dec 1;2(12):1475–95. doi: 10.1534/g3.112.003863 (PMC3516471; doi:10.1534/g3.112.003863)
Supplement: Supporting Information [file supp_2.12.1475_FileS1.pdf]

File S1 – Extended Methods

1. Estimation of optimal parameters for soft clustering

The parameters for the fuzzy c-means (FCM) algorithm (i.e., the number of clusters *c* and the fuzzifier *m*) were obtained following the approach proposed by Schwämmle and Jensen (2010). An optimal value of 2.02 for the fuzzifier *m* was estimated from the number of clustered genes (N= 3218) and the number of time points (D=5) using their heuristically derived formula. This setting of parameter *m* ensures that no clusters are found for randomized data with the same number of genes and time points.

The minimum centroid distance (D_{min}) was calculated for repeated clustering, with c ranging from 2 to 20 (Figure S1). In general, D_{min} decreases monotonically. For values larger than c=4, however, D_{min} decreases notably less, indicting *c*=4 as an optimal solution (Schwämmle and Jensen 2010). The complete set of expressed genes resulting from filtering was utilized for this calculation.

2. Previous *Synechocystis* microarray studies included in meta-analysis

Microarray data obtained in our study were compared to three previously published microarray experiments that monitored the transcriptional response in *Synechocystis* to perturbations of extra-cellular iron concentration (Singh, McIntyre et al. 2003; Shcolnick, Summerfield et al. 2009; Houot, Floutier et al. 2007).

Singh et al. measured the gene expression during the recovery from stress produced by lack of iron. Firstly, cells were grown for at least 6 days in iron-free media at 30°C with a light intensity of 20-30 µmol photons m−2•s−1. Subsequently, iron was added directly to the media and samples were taken at 0, 3, 12 and 24 hours after reconstitution.

Houot et al. induced iron starvation in *Synechocystis* cells by growing them in media containing 1 or 2 mM of ferric ammonium citrate for 2 days at 30°C with a light intensity around 30 µmol photons m−2•s−1. Cells were washed and re-suspended in iron-free media for another 2 days before RNA was extracted. Houot et al. also measured gene expression in cells grown under iron excess. Here, cells were exposed to an iron concentration 1000 times higher (17mM) than that of standard BG11 (17 µM). Samples were taken 4 and 6 hours after the addition of the extra iron.

Shcolnick et al. extracted RNA from iron starved cells using desferrioxamine B (DFB) as a chelator (Shcolnick, Shaked et al. 2007). The cells were grown at 30°C with a light intensity of 60 µmol photons m−2•s−1 in the modified BG11 media, YBG11, in the presence of 50 µM DFB.

Besides the differences introduced due to experimental set-up and sampling time, experiments also differed in the microarrays used: both Singh et al. and Shcolnick et al. applied the platform described by Postier et al. (Postier, Wang et al. 2003), which is based on a two stage polymerase chain reaction (PCR) method to create probes against 3165 genes in the *Synechocystis* chromosome; whereas Houot et al. used the commercially available CyanoCHIP (http://www.takara-bio.com), which employs PCR fragments of C-terminal coding regions from 3079 genes in the Synechocystis chromosome as probes.

As result, the four independent studies partially differed in the set of genes detected as differentially expressed. Such divergence is a common feature when comparing gene expression data sets derived from different platforms. More importantly, however, the gene sets derived from the four studies show significant overlap. The results from our study are most similar to those by Houot et al. with 165 shared genes (40.5 % of differentially expressed genes in Houot et al.) (Figure S5). Less overlap was detected with the studies of Shcolnick et al. (112 genes; 33.7 %) and Singh et al. (72 genes; 23.8 %). In general, the measured expression levels tend to be concurrent, as shown by the calculated Pearson's correlation coefficients of $\log_2$FC for differentially expressed genes between our and previous studies. The highest correlation was obtained for the study by Houot et al. (R2 = 0.55) (Figure 4D), followed by Shcolnick et al. (R2 = 0.47) (Figure 4C) and Singh et al. (R2 = 0.46) (Figure 4B). Based on the corresponding contingency tables for differential expression, the observed

similarity with previous studies was highly significant (Houot et al.: $p = 2.2x10^{-16}$, Shcolnick et al.: $p = 4.0x10^{-12}$, Singh et al.: $p = 0.02$).
