# Supplementary material for: Iron Deprivation in Synechocystis: Inference of Pathways, Non-coding RNAs, and Regulatory Elements from Comprehensive Expression Profiling
Source: G3 (Bethesda). 2012 Dec 1;2(12):1475–95. doi: 10.1534/g3.112.003863 (PMC3516471; doi:10.1534/g3.112.003863)
Supplement: Supporting Information [file supp_2.12.1475_TableS1.pdf]

Table S1 List of primers used to generate single-stranded RNA probes to test gene expression under iron limiting conditions.

Region targeted	Primer name	Sequence
5’UTR-isiA	5´UTR-isiA-fw	5’-TAATACGACTCACTATAGG GCACAGAATTGCCTCCTTAATTGA-3’
	5`UTR-isiA-rev	5’-ATCAGTGGTTTGAGCTTAGTCC-3’
NC-181	NC-181-T7-fw	5’-TAATACGACTCACTATAGG GCAAAAAGTTAACAACGGACACG-3’
	NC-181-rev	5’-AGTGTTCTCTTCTCAAGGATTCAG-3’
NC-1321	NC-1321-T7-fw	5’-TAATACGACTCACTATAGG GCAAGGTAGATTGGCAGTGGG-3’
	NC-1321-rev	5’-CATGGTCAACAACAGGAGAAGG-3’
NC-350	NC-350-T7-fw	5’-TAATACGACTCACTATAGG GGAGCCGGTGGGGAGAG-3’
	NC-350-rev	5´-CGAAGTAAGTTTTTCGATTCCGTC-3’
rnpB	rnpB-T7-fw	5’-TAATACGACTCACTATAGG CCTTTTCAGTGGTCAGTTACC-3’
	rnpB-rev	5’-AGGCCAAACTTGCTGGGTAAC-3’
