# Supplementary material for: Iron Deprivation in Synechocystis: Inference of Pathways, Non-coding RNAs, and Regulatory Elements from Comprehensive Expression Profiling
Source: G3 (Bethesda). 2012 Dec 1;2(12):1475–95. doi: 10.1534/g3.112.003863 (PMC3516471; doi:10.1534/g3.112.003863)
Supplement: Supporting Information [file supp_2.12.1475_TableS5.pdf]

Table S5 Genes detected as differentially expressed in all iron-stress studies compared in this article.

Gene ID	NAME	Houot et al.				Sch	Singh et al.			This study				
		+Fe 4h	+Fe 6h	-Fe A	-Fe B	SHC	3h	12h	24h	3 h	12 h	24 h	48 h	72 h
slr0452	dihydroxyacid dehydratase	0.55	1.31	-0.58	-0.27	-0.66	-0.72	-0.95	-0.55	0.37	-0.20	-0.54	-0.68	-0.50
slr0899	cyanate lyase	-1.73	-3.32	2.49	2.41	0.77	-0.08	-0.56	-0.93	1.46	0.62	1.26	0.12	0.65
sll0247	iron-stress chlorophyll-binding protein, isiA	-3.17	-5.89	5.45	3.49	3.51	3.38	4.27	4.47	2.52	5.99	5.53	6.14	6.15
sll0248	flavodoxin, IsiB	-3.81	-6.91	6.49	4.19	2.40	3.83	3.53	3.19	2.83	6.21	5.44	6.66	6.86
sll1408	transcriptional regulator	-1.62	-4.26	1.91	1.40	1.16	1.02	0.99	0.83	-0.51	0.76	0.17	0.87	0.78
slr1214	two-component response regulator PatA subfamily	-0.14	-1.81	1.03	2.08	1.47	0.71	-0.10	-0.19	-2.03	-3.40	-1.46	-2.31	-2.07
slr0955	probable tRNA/rRNA methyltransferase	0.3	0.6	-0.40	-1.06	-0.55	-0.39	0.03	0.97	0.72	0.96	0.81	0.86	0.66
sll1406	ferrichrome-iron receptor	-3.26	-6.19	4.48	3.05	2.34	1.08	1.06	1.20	1.54	3.51	2.43	2.75	2.52
sll1878	iron(III)-transport ATP-binding protein	-1.54	-3.07	1.27	1.40	1.85	1.23	1.23	1.20	1.53	2.15	1.98	2.29	2.60
slr0447	ABC-type urea transport system substrate-binding protein	-1.43	-3.01	1.63	0.86	-0.66	-0.36	-1.18	-1.50	2.15	0.84	0.90	-0.87	-0.57
slr0513	iron transport system substrate-binding protein	-1.85	-4.55	3.30	2.67	2.71	1.92	2.09	1.93	3.32	4.00	3.64	4.00	3.96
slr1318	iron(III) dicitrate transport system ATP-binding protein	-1.91	-3.59	1.08	1.11	2.54	0.82	0.64	0.73	0.32	1.70	1.18	1.55	1.39
sll1407	probable methyltransferase	-4.31	-5.58	3.80	2.81	0.73	0.70	0.77	0.55	0.53	2.35	1.46	2.10	1.95
sll0249	hypothetical protein	-7.13	-6.55	4.45	2.60	3.75	3.60	3.78	3.82	1.39	4.74	3.64	5.34	5.33
sll0451	hypothetical protein	0.37	-1.51	-0.51	-0.18	-0.62	-0.41	-0.53	-0.60	-1.20	-0.96	-0.20	-0.49	-0.73
sll0529	hypothetical protein	-0.17	1.15	-0.24	0.32	0.53	-0.53	-0.38	-0.79	0.98	1.30	0.79	0.36	0.81
sll0662	4Fe-4S type iron-sulfur protein	-0.68	-1.42	0.55	0.84	0.80	1.57	1.94	1.54	0.18	0.68	0.35	0.72	1.14
sll1549	salt-enhanced periplasmic protein	-3.7	-7.33	4.88	3.57	3.00	2.33	2.11	2.14	1.74	3.93	3.12	3.70	3.87
sll1734	protein involved in	-0.63	3.48	0.21	0.21	-0.68	-0.37	-0.41	-0.56	-3.20	-4.05	-2.99	-3.62	-3.52

slr1512	CO_2 uptake sodium-dependent bicarbonate transporter	-0.26	4.84	3.45	-0.38	-1.25	-0.53	-0.33	-0.58	-3.67	-4.30	-3.46	-5.54	-5.28
ssl0461	hypothetical protein	-0.21	-3.34	1.03	-0.09	3.96	1.16	0.96	0.74	0.89	2.83	1.87	3.37	3.42
ssl1263	hypothetical protein	-0.25	-1.62	0.16	1.03	0.74	0.81	0.86	0.96	0.36	0.92	0.41	0.85	1.13
sll1862	unknown protein	-0.32	-2.98	-4.27	-0.86	2.18	0.31	0.73	1.10	-0.28	0.24	0.80	3.58	4.36
sll1863	unknown protein	-0.27	-2.97	-2.66	-0.83	2.03	0.75	0.82	0.80	-0.92	-0.26	0.59	3.53	4.51
slr0006	unknown protein	0.37	3.07	-0.56	-0.25	-1.15	-0.70	-0.36	-0.86	-1.61	-1.28	-1.43	-2.07	-2.15
slr1484	unknown protein	-2.82	-4.99	2.17	2.23	1.80	1.91	2.37	1.91	1.73	3.60	2.89	3.47	3.77
slr1485	putative phosphatidylinositol phosphate kinase	-2.92	-6.31	3.19	3.38	3.74	1.82	1.85	1.10	2.09	3.92	3.46	3.86	4.47
slr1544	LilA, light-harvesting-like (Lil) protein A	-0.24	1.21	-0.84	0.42	0.61	0.86	1.10	1.08	-1.19	-1.76	0.97	-0.05	-0.73
