# Supplementary material for: Iron Deprivation in Synechocystis: Inference of Pathways, Non-coding RNAs, and Regulatory Elements from Comprehensive Expression Profiling
Source: G3 (Bethesda). 2012 Dec 1;2(12):1475–95. doi: 10.1534/g3.112.003863 (PMC3516471; doi:10.1534/g3.112.003863)
Supplement: Supporting Information [file supp_2.12.1475_TableS6.pdf]

Table S6 Antisense RNAs differentially expressed. asRNAs are ordered and colored similarly to the two clusters defined in figure 4B (cluster I, red shade; cluster II, blue shade).

Gene ID	Description	3 hours	12 hours	24 hours	48 hours	72 hours	*q*-value
accA-0-x	sll0728-as	-1.69	-1.57	-0.27	-0.57	-1.21	$2.10 \cdot 10^{-5}$
cysT-0-x	slr1453-as	-0.26	-0.12	1.41	0.88	0.62	$4.79 \cdot 10^{-5}$
fbp-0-x	sll1636-as	-2.73	-2.63	-1.88	-1.60	-1.63	$1.29 \cdot 10^{-5}$
hik31-0-x	sll0790-as	-0.28	-0.33	1.26	0.51	0.22	$4.78 \cdot 10^{-6}$
kaiA-as1-0-x	slr0756-as	-1.57	-1.66	-0.55	-0.59	-0.79	$8.16 \cdot 10^{-6}$
sll0006-as1	asRNA	-1.49	-1.46	-0.82	-0.39	-0.76	$7.03 \cdot 10^{-8}$
sll0019-as2	asRNA	-2.17	-2.00	-0.75	-0.18	-1.04	$3.44 \cdot 10^{-4}$
sll0026-as3	asRNA	-1.63	-1.63	-0.15	-0.12	-0.85	$1.13 \cdot 10^{-3}$
sll0033-as1	asRNA	-1.85	-1.85	-0.31	0.06	-0.82	$1.71 \cdot 10^{-7}$
sll0034-as1	asRNA	-1.53	-1.71	-1.01	-1.01	-1.37	$1.43 \cdot 10^{-5}$
sll0040-as2	asRNA	-0.97	-1.04	0.29	0.34	-0.32	$2.23 \cdot 10^{-5}$
sll0067-as2	asRNA	-1.98	-2.00	-0.21	-0.46	-1.00	$6.41 \cdot 10^{-7}$
sll0067-as3	asRNA	-1.61	-1.77	-0.84	-0.60	-1.10	$2.07 \cdot 10^{-5}$
sll0068-as3	asRNA	-1.44	-1.37	0.21	-0.14	-0.72	$1.08 \cdot 10^{-4}$
sll0068-as4	asRNA	-1.87	-1.88	-0.21	-0.32	-1.06	$1.06 \cdot 10^{-4}$
sll0068-as6	asRNA	-1.64	-1.73	-0.06	0.01	-0.80	$1.66 \cdot 10^{-7}$
sll0083-as1	asRNA	-1.13	-1.03	-0.13	-0.20	-0.42	$1.37 \cdot 10^{-6}$
sll0095-as1	asRNA	-1.34	-1.28	0.19	-0.17	-0.46	$2.31 \cdot 10^{-6}$
sll0157-as1	asRNA	-1.23	-1.29	-0.17	-0.26	-0.66	$1.25 \cdot 10^{-3}$
sll0172-as1	asRNA	-1.14	-1.20	-0.20	-0.56	-1.03	$2.09 \cdot 10^{-7}$
sll0172-as2	asRNA	-1.54	-1.49	0.43	-0.33	-0.77	$1.11 \cdot 10^{-6}$
sll0174-as1	asRNA	-1.28	-1.63	-0.10	-0.01	-0.58	$9.62 \cdot 10^{-5}$
sll0204-as2	asRNA	-0.20	-0.12	1.01	0.55	0.47	$7.31 \cdot 10^{-5}$
sll0225-as1	asRNA	-1.23	-1.13	-0.03	-0.03	-0.62	$5.30 \cdot 10^{-7}$
sll0226-as1	asRNA	-1.59	-1.45	0.29	-0.15	-0.87	$1.56 \cdot 10^{-6}$
sll0227-as1	asRNA	-1.51	-1.44	-0.15	-0.02	-0.71	$7.51 \cdot 10^{-4}$
sll0230-as1	asRNA	-1.25	-0.97	0.50	-0.15	-0.49	$8.22 \cdot 10^{-7}$
sll0238-as1	asRNA	-1.20	-1.19	-0.16	-0.33	-0.89	$4.33 \cdot 10^{-6}$
sll0297-as1	asRNA	-1.01	-1.07	0.02	-0.24	-0.77	$1.30 \cdot 10^{-6}$
sll0330-as2	asRNA	-1.26	-1.21	-0.73	0.14	-0.74	$1.68 \cdot 10^{-4}$
sll0370-as1	asRNA	-1.03	-1.05	-0.57	-0.15	-0.77	$2.34 \cdot 10^{-4}$
sll0374-as2	asRNA	-1.05	-0.89	-0.19	-0.41	-0.45	$2.11 \cdot 10^{-4}$
sll0416-as2	asRNA	-1.15	-1.20	-0.32	-0.45	-0.96	$6.58 \cdot 10^{-6}$
sll0424-as1	asRNA	-1.13	-1.65	-0.82	-0.54	-1.12	$5.41 \cdot 10^{-6}$
sll0474-as1	asRNA	-1.94	-1.93	-0.24	-0.03	-0.96	$6.21 \cdot 10^{-5}$
sll0477-as1	asRNA	-2.04	-2.07	-0.90	-0.20	-1.30	$5.12 \cdot 10^{-5}$
sll0480-as1	asRNA	-2.32	-2.17	-0.96	-0.28	-1.12	$7.00 \cdot 10^{-5}$
sll0495-as1	asRNA	-1.15	-1.21	0.00	-0.47	-0.81	$3.58 \cdot 10^{-7}$
sll0502-as1	asRNA	-2.06	-2.02	-0.27	-0.05	-0.86	$1.74 \cdot 10^{-6}$
sll0503-as1	asRNA	-1.76	-1.67	-0.63	-0.69	-1.10	$1.97 \cdot 10^{-5}$
sll0506-as1	asRNA	-1.49	-1.56	-0.37	0.08	-0.48	$5.80 \cdot 10^{-6}$
sll0518-as1	asRNA	-0.82	-1.25	-0.25	-0.35	-0.76	$4.61 \cdot 10^{-5}$
sll0540-as1	asRNA	-1.08	-0.83	-0.34	0.10	0.21	$4.54 \cdot 10^{-6}$
sll0543-as1	asRNA	-1.33	-1.32	0.13	-0.31	-0.82	$3.64 \cdot 10^{-7}$
sll0574-as1	asRNA	-1.33	-1.51	-0.11	0.23	-0.48	$4.91 \cdot 10^{-5}$
sll0616-as1	asRNA	-1.90	-1.90	-0.02	-0.37	-1.02	$4.36 \cdot 10^{-8}$
sll0638-as1	asRNA	-1.19	-1.10	0.57	0.33	-0.33	$1.63 \cdot 10^{-3}$
sll0640-as4	asRNA	-1.91	-1.99	-0.32	-0.09	-0.98	$4.21 \cdot 10^{-5}$
sll0732-as2	asRNA	-0.96	-1.01	0.53	-0.25	-0.57	$2.60 \cdot 10^{-4}$
sll0759-as2	asRNA	-1.11	-0.89	0.00	-0.33	-0.48	$3.72 \cdot 10^{-6}$
sll0778-0-x	asRNA	-1.19	-1.20	0.46	-0.02	-0.46	$5.97 \cdot 10^{-6}$
sll0779-as1	asRNA	-1.37	-1.28	-0.58	-0.34	-0.84	$2.98 \cdot 10^{-3}$
sll0807-as1	asRNA	-1.52	-1.38	-0.47	-0.35	-0.53	$3.30 \cdot 10^{-6}$
sll0814-as2	asRNA	-0.20	-0.14	1.02	0.49	0.47	$1.28 \cdot 10^{-5}$
sll0825-as1	asRNA	-1.22	-1.10	-0.15	0.15	-0.46	$3.28 \cdot 10^{-7}$
sll0830-as1	asRNA	-1.14	-1.13	0.47	0.01	-0.82	$1.53 \cdot 10^{-7}$
sll0843-as1	asRNA	-2.21	-2.29	-0.64	-0.34	-1.07	$2.39 \cdot 10^{-6}$
sll0887-as1	asRNA	-1.85	-2.16	-1.28	-1.15	-1.69	$3.36 \cdot 10^{-7}$
sll0901-as1	asRNA	-1.02	-1.16	0.20	-0.16	-0.61	$1.93 \cdot 10^{-6}$
sll0913-as1	asRNA	-1.09	-0.95	-0.16	-0.47	-0.66	$1.86 \cdot 10^{-6}$

sll0920-as1	asRNA	-1.90	-1.90	-0.63	-0.53	-1.14	$1.64·10^{-6}$
sll0926-1-x	asRNA	-1.08	-0.77	0.02	-0.02	-0.34	$1.00·10^{-4}$
sll0931-as2	asRNA	-1.47	-1.53	-0.26	-0.36	-0.95	$2.07·10^{-5}$
sll0992-as1	asRNA	-2.06	-2.17	-1.04	-0.41	-1.04	$3.29·10^{-8}$
sll0992-as2	asRNA	-1.25	-1.26	0.34	0.12	-0.25	$1.43·10^{-4}$
sll1023-as2	asRNA	-1.11	-1.03	0.33	0.36	-0.23	$3.50·10^{-6}$
sll1043-as3	asRNA	-1.31	-1.30	0.54	-0.06	-0.70	$4.18·10^{-7}$
sll1049-0-x	asRNA	-1.36	-1.30	0.19	-0.05	-0.51	$8.54·10^{-6}$
sll1060-as1	asRNA	-1.46	-1.47	0.09	-0.06	-0.51	$1.57·10^{-6}$
sll1119-as4	asRNA	-1.23	-1.36	0.17	-0.19	-0.63	$7.02·10^{-7}$
sll1123-as1	asRNA	-0.72	-0.87	-0.20	-0.46	-1.07	$3.91·10^{-4}$
sll1130-as1	asRNA	-1.15	-1.03	-0.27	-0.06	-0.72	$8.91·10^{-4}$
sll1131-as1	asRNA	-0.96	-1.10	-0.38	-0.24	-0.74	$5.93·10^{-7}$
sll1172-as1	asRNA	-1.26	-1.24	-0.65	-0.85	-1.28	$4.18·10^{-7}$
sll1178-as2	asRNA	-1.78	-1.67	-0.60	-0.39	-0.89	$2.63·10^{-5}$
sll1200-as1	asRNA	-1.38	-1.40	-0.62	-0.62	-0.49	$8.17·10^{-7}$
sll1200-as2	asRNA	-1.20	-1.12	-0.34	-0.67	-0.36	$5.43·10^{-5}$
sll1204-as1	asRNA	-1.51	-1.35	-0.05	-0.08	-0.66	$1.17·10^{-4}$
sll1206-as1	asRNA	-1.52	-1.76	-0.53	-0.86	-1.21	$1.30·10^{-5}$
sll1275-as1	asRNA	-2.09	-1.68	-0.66	-0.73	-1.08	$3.58·10^{-4}$
sll1276-as2	asRNA	-1.04	-1.08	0.43	0.38	-0.34	$1.21·10^{-5}$
sll1283-as2	asRNA	-1.89	-1.74	-0.32	-0.59	-1.09	$6.69·10^{-8}$
sll1285-as1	asRNA	-1.56	-1.66	0.10	-0.20	-0.65	$1.73·10^{-7}$
sll1293-as1	asRNA	-1.21	-0.95	-0.32	-0.26	-0.54	$1.43·10^{-6}$
sll1293-as2	asRNA	-1.17	-1.09	-0.20	0.01	-0.51	$2.05·10^{-4}$
sll1296-as3	asRNA	-1.56	-1.56	-0.36	-0.40	-0.77	$1.04·10^{-6}$
sll1330-as1	asRNA	-1.34	-1.35	-0.41	-0.42	-0.40	$1.09·10^{-4}$
sll1334-as3	asRNA	-1.61	-1.88	-0.10	-0.38	-0.87	$3.65·10^{-7}$
sll1341-as1	asRNA	-1.62	-1.81	-0.46	-0.49	-1.06	$1.69·10^{-5}$
sll1354-as3	asRNA	-1.32	-1.23	-0.25	-0.26	-0.90	$7.28·10^{-4}$
sll1378-as2	asRNA	-1.85	-1.93	-0.78	-0.30	-1.07	$6.63·10^{-8}$
sll1386-as1	asRNA	-1.69	-1.74	-0.19	-0.25	-0.98	$7.90·10^{-8}$
sll1432-as1	asRNA	-0.83	-1.00	-0.25	-0.47	-0.67	$9.69·10^{-5}$
sll1434-as1	asRNA	-2.02	-2.03	-0.17	-0.26	-0.82	$5.94·10^{-7}$
sll1459-as1	asRNA	-1.21	-1.23	-0.47	-0.15	-0.70	$7.12·10^{-6}$
sll1486-as1	asRNA	-2.18	-2.06	-0.11	-0.46	-0.98	$1.45·10^{-6}$
sll1500-as1	asRNA	-1.43	-1.48	0.41	0.31	-0.26	$2.61·10^{-5}$
sll1515-as6	asRNA	-1.19	-1.02	-0.07	-0.14	-0.31	$3.89·10^{-3}$
sll1525-as1	asRNA	-1.37	-1.37	-0.64	-0.29	-0.88	$1.68·10^{-4}$
sll1538-as1	asRNA	-1.17	-1.18	-0.29	-0.50	-0.81	$1.88·10^{-5}$
sll1601-as1	asRNA	-0.16	-0.77	-0.39	-0.54	-1.01	$3.78·10^{-4}$
sll1612-as1	asRNA	-2.23	-1.92	-0.75	-0.52	-1.25	$4.41·10^{-4}$
sll1635-as1	asRNA	-1.87	-1.90	-0.52	0.00	-0.82	$7.81·10^{-3}$
sll1641-as1	asRNA	-1.70	-1.78	0.16	-0.23	-0.76	$2.84·10^{-6}$
sll1677-as1	asRNA	-1.69	-1.65	0.36	0.05	-0.59	$3.72·10^{-6}$
sll1685-as3	asRNA	-1.17	-1.06	-0.65	-0.29	-0.58	0.01
sll1722-as1	asRNA	-1.78	-1.80	-0.21	-0.09	-0.88	$1.87·10^{-6}$
sll1724-as1	asRNA	-1.60	-1.58	-0.26	-0.23	-0.76	$3.26·10^{-5}$
sll1750-as4	asRNA	-0.98	-1.05	-0.36	-0.29	-0.78	$1.84·10^{-4}$
sll1800-as3	asRNA	-0.75	-0.94	-0.40	-0.55	-1.01	$1.24·10^{-5}$
sll1853-as1	asRNA	-1.79	-1.88	-0.59	-0.42	-1.12	$2.68·10^{-8}$
sll1866-as1	asRNA	-1.61	-1.71	-1.22	-1.28	-1.25	$8.47·10^{-7}$
sll1870-as1	asRNA	-1.02	-1.20	0.41	0.16	-0.04	$5.93·10^{-5}$
sll1878-as1	asRNA	-0.62	-1.25	-0.51	-0.81	-1.31	$1.45·10^{-4}$
sll1878-as2	asRNA	-0.90	-1.10	0.66	0.73	-0.07	$1.37·10^{-4}$
sll1886-as1	asRNA	-1.16	-1.12	-0.34	-0.12	-0.59	$1.53·10^{-4}$
sll1906-as1	asRNA	0.07	-0.01	1.17	0.36	0.20	$1.76·10^{-6}$
sll1927-as1	asRNA	-1.43	-1.54	0.35	-0.16	-0.76	$4.77·10^{-6}$
sll1987-as1	asRNA	-1.68	-1.67	-0.44	-0.44	-0.98	$2.81·10^{-6}$
sll2002-as1	asRNA	-0.81	-1.01	0.83	0.93	0.06	$3.25·10^{-3}$
sll2003-as3	asRNA	-1.01	-1.04	0.18	-0.10	-0.59	$4.52·10^{-4}$
sll2008-as1	asRNA	-1.03	-1.08	0.29	0.06	-0.48	$2.12·10^{-6}$
sll7029-as1	asRNA	-1.38	-1.52	-0.56	-0.85	-1.13	$2.42·10^{-6}$
sll7063-as1	asRNA	-1.09	-1.13	-0.14	-0.35	-0.88	$2.57·10^{-7}$
sll7077-as1	asRNA	-1.70	-1.88	-0.01	-0.28	-0.95	$4.66·10^{-6}$

slr0007-as1	asRNA	-1.01	-1.11	-0.35	-0.48	-0.76	$5.60 \cdot 10^{-7}$
slr0079-as1	asRNA	-1.45	-1.42	-0.25	-0.40	-0.77	$5.00 \cdot 10^{-6}$
slr0080-as2	asRNA	-1.34	-0.98	-0.57	-0.55	-0.65	$4.94 \cdot 10^{-6}$
slr0208-as1	asRNA	-1.86	-1.91	0.02	-0.18	-0.91	$2.41 \cdot 10^{-6}$
slr0208-as2	asRNA	-1.16	-1.29	-0.43	-0.26	-0.58	$3.94 \cdot 10^{-6}$
slr0211-as2	asRNA	-1.01	-1.09	0.37	-0.33	-0.80	$6.57 \cdot 10^{-7}$
slr0244-as3	asRNA	-0.97	-1.02	-0.53	-0.68	-0.74	$6.89 \cdot 10^{-3}$
slr0252-as1	asRNA	-1.80	-1.87	-0.98	-0.90	-1.29	$2.93 \cdot 10^{-3}$
slr0293-as1	asRNA	-1.68	-1.63	0.08	0.07	-0.81	$1.21 \cdot 10^{-4}$
slr0304-as1	asRNA	-1.46	-1.53	-0.62	-0.60	-0.90	$4.25 \cdot 10^{-6}$
slr0327-as1	asRNA	-1.32	-1.16	-0.03	0.03	-0.71	$1.56 \cdot 10^{-5}$
slr0331-as2	asRNA	-1.21	-1.14	-0.69	-0.79	-0.78	$2.50 \cdot 10^{-4}$
slr0337-as1	asRNA	-1.25	-1.35	0.36	-0.24	-0.74	$2.38 \cdot 10^{-7}$
slr0345-as1	asRNA	-1.14	-1.11	0.43	-0.01	-0.60	$3.05 \cdot 10^{-6}$
slr0359-as2	asRNA	-1.09	-1.11	0.07	0.03	-0.72	$6.65 \cdot 10^{-4}$
slr0370-as2	asRNA	-1.00	-0.84	-0.24	-0.23	-0.63	$3.78 \cdot 10^{-6}$
slr0370-as3	asRNA	-1.40	-1.19	-0.69	-0.27	-0.96	$1.46 \cdot 10^{-4}$
slr0377-as1	asRNA	-1.48	-1.37	-0.72	-0.63	-0.91	$2.83 \cdot 10^{-6}$
slr0408-0-x	asRNA	-2.13	-2.11	-0.65	-0.28	-0.95	$5.51 \cdot 10^{-6}$
slr0408-1-x	asRNA	-1.84	-1.80	0.19	-0.05	-0.82	$6.77 \cdot 10^{-4}$
slr0408-as11	asRNA	-1.67	-1.77	0.22	-0.14	-0.83	$1.10 \cdot 10^{-7}$
slr0415-as3	asRNA	-1.49	-1.35	-0.63	-0.62	-1.07	$5.37 \cdot 10^{-7}$
slr0467-as1	asRNA	-1.31	-1.39	-0.22	-0.07	-0.77	$6.84 \cdot 10^{-6}$
slr0474-as1	asRNA	-0.85	-1.32	-0.13	-0.12	-1.00	$5.24 \cdot 10^{-6}$
slr0488-as1	asRNA	-1.21	-1.14	0.38	0.25	-0.27	$3.16 \cdot 10^{-4}$
slr0519-as1	asRNA	-1.14	-0.98	0.28	-0.60	-0.39	$9.03 \cdot 10^{-5}$
slr0534-as5	asRNA	-1.80	-1.90	-0.52	-0.48	-0.83	$2.59 \cdot 10^{-7}$
slr0541-as1	asRNA	0.62	0.54	1.69	0.80	0.59	$8.98 \cdot 10^{-6}$
slr0559-as1	asRNA	-1.62	-1.70	-1.09	-1.18	-1.33	$2.12 \cdot 10^{-6}$
slr0579-as1	asRNA	-1.25	-1.23	0.60	0.27	-0.24	$9.94 \cdot 10^{-7}$
slr0585-as3	asRNA	-1.71	-1.76	0.34	-0.10	-0.78	$1.11 \cdot 10^{-5}$
slr0593-as1	asRNA	-2.09	-1.95	-0.94	0.04	-1.09	$9.62 \cdot 10^{-4}$
slr0599-as1	asRNA	-1.48	-1.52	-0.02	0.10	-0.50	$3.27 \cdot 10^{-5}$
slr0619-as1	asRNA	-1.29	-1.24	-0.46	-0.42	-0.59	$1.08 \cdot 10^{-5}$
slr0711-as1	asRNA	-1.66	-1.74	0.26	-0.23	-0.91	$1.29 \cdot 10^{-6}$
slr0727-as1	asRNA	-1.38	-1.50	-0.05	0.17	-0.62	$2.60 \cdot 10^{-4}$
slr0822-as2	asRNA	-1.38	-1.16	-0.17	-0.56	-1.01	$3.89 \cdot 10^{-4}$
slr0842-as1	asRNA	-1.31	-1.33	0.54	0.07	-0.57	$3.48 \cdot 10^{-5}$
slr0848-0-x	asRNA	-0.99	-1.13	-0.30	-0.46	-0.87	$1.11 \cdot 10^{-5}$
slr0872-as1	asRNA	-1.21	-1.24	-0.27	-0.41	-0.90	$1.72 \cdot 10^{-4}$
slr0872-as2	asRNA	-1.04	-1.03	0.19	0.07	-0.28	$1.10 \cdot 10^{-5}$
slr0898-as1	asRNA	-1.28	-1.40	-0.23	0.12	-0.57	$6.49 \cdot 10^{-6}$
slr0900-as1	asRNA	-1.28	-1.43	-0.96	-0.88	-1.22	$1.34 \cdot 10^{-6}$
slr0905-as3	asRNA	-1.60	-1.45	0.32	0.09	-0.54	$9.04 \cdot 10^{-7}$
slr0936-as2	asRNA	-1.84	-1.94	-0.24	-0.25	-1.02	$1.87 \cdot 10^{-6}$
slr0942-as1	asRNA	-0.99	-1.11	-0.72	0.01	-0.71	0.01
slr0993-as4	asRNA	-0.99	-1.04	0.30	-0.12	-0.43	$3.29 \cdot 10^{-7}$
slr1020-as1	asRNA	-1.75	-1.64	-0.46	-0.51	-1.08	$1.67 \cdot 10^{-4}$
slr1022-as1	asRNA	-1.57	-1.59	-0.22	-0.11	-0.83	$6.35 \cdot 10^{-8}$
slr1028-as15	asRNA	-1.03	-1.07	-0.37	-0.38	-0.77	$4.55 \cdot 10^{-5}$
slr1039-as2	asRNA	-2.66	-2.41	-0.57	-0.07	-0.92	$2.17 \cdot 10^{-5}$
slr1050-as1	asRNA	-1.47	-1.48	-0.44	-0.22	-0.79	$7.52 \cdot 10^{-6}$
slr1051-as2	asRNA	-1.47	-1.41	-0.50	-0.36	-0.83	$5.54 \cdot 10^{-5}$
slr1101-as1	asRNA	-1.92	-1.96	-0.42	-0.50	-0.94	$2.91 \cdot 10^{-5}$
slr1102-0-x	asRNA	-1.17	-1.06	-0.49	-0.37	-0.61	$6.75 \cdot 10^{-6}$
slr1103-as4	asRNA	-1.50	-1.54	-0.11	-0.36	-1.08	$1.21 \cdot 10^{-5}$
slr1104-as3	asRNA	-1.20	-1.33	0.33	0.03	-0.48	$4.82 \cdot 10^{-6}$
slr1123-as1	asRNA	-1.57	-1.64	-0.16	-0.10	-0.67	$8.61 \cdot 10^{-8}$
slr1161-as1	asRNA	-1.72	-2.38	-0.32	-0.05	-0.76	$1.71 \cdot 10^{-7}$
slr1174-as1	asRNA	-1.14	-1.21	0.28	-0.22	-0.94	$1.07 \cdot 10^{-6}$
slr1181-as1	asRNA	-1.78	-1.90	-0.06	-0.36	-1.00	$2.65 \cdot 10^{-6}$
slr1207-0-x	asRNA	-1.28	-1.30	0.61	0.27	-0.36	$6.51 \cdot 10^{-7}$
slr1207-as2	asRNA	-0.49	-0.52	1.12	0.61	0.12	$6.10 \cdot 10^{-4}$
slr1219-as2	asRNA	-1.18	-1.19	-0.22	0.00	-0.63	$9.39 \cdot 10^{-6}$
slr1228-as1	asRNA	-2.32	-2.18	0.14	-0.30	-0.94	$3.08 \cdot 10^{-7}$

slr1229-as1	asRNA	-0.14	-0.23	1.43	0.86	0.62	$1.34 \cdot 10^{-4}$
slr1254-as1	asRNA	-1.16	-1.07	0.28	-0.04	-0.51	$1.38 \cdot 10^{-5}$
slr1272-as1	asRNA	-2.95	-2.82	-1.03	-0.38	-1.43	$4.22 \cdot 10^{-4}$
slr1280-as1	asRNA	-1.16	-1.17	-0.41	0.02	-0.41	$1.71 \cdot 10^{-5}$
slr1293-as1	asRNA	-1.21	-1.27	-0.40	-0.22	-0.70	$8.39 \cdot 10^{-6}$
slr1305-as1	asRNA	-2.29	-2.15	-0.69	-0.27	-1.44	$4.14 \cdot 10^{-6}$
slr1318-as1	asRNA	-0.57	-1.11	-0.52	-0.67	-0.94	$8.30 \cdot 10^{-6}$
slr1324-as3	asRNA	-1.19	-1.15	-0.53	-0.59	-1.04	$8.19 \cdot 10^{-5}$
slr1347-as1	asRNA	-1.02	-1.03	0.15	-0.03	-0.53	$1.77 \cdot 10^{-6}$
slr1367-as1	asRNA	-1.05	-1.21	0.05	0.11	-0.32	$3.27 \cdot 10^{-4}$
slr1383-as1	asRNA	-1.64	-1.55	-0.17	-0.23	-0.81	$8.74 \cdot 10^{-5}$
slr1392-as1	asRNA	-1.82	-1.96	-0.46	-0.17	-0.95	$4.24 \cdot 10^{-5}$
slr1403-as6	asRNA	-1.55	-1.52	-0.62	-0.01	-0.78	$1.06 \cdot 10^{-4}$
slr1403-as8	asRNA	-1.16	-1.17	-0.72	-0.09	-0.67	$1.19 \cdot 10^{-6}$
slr1428-as1	asRNA	-0.66	-0.63	1.06	0.45	0.12	$8.21 \cdot 10^{-6}$
slr1435-as1	asRNA	-1.23	-1.30	0.46	-0.16	-0.41	$4.90 \cdot 10^{-5}$
slr1462-as1	asRNA	-2.20	-2.18	-0.99	-0.61	-1.19	$1.97 \cdot 10^{-7}$
slr1462-as2	asRNA	-1.36	-1.38	-0.43	-0.08	-0.54	$7.26 \cdot 10^{-6}$
slr1489-as1	asRNA	-1.84	-1.61	-0.79	-0.51	-0.84	$5.99 \cdot 10^{-6}$
slr1512-as1	asRNA	-1.41	-1.51	0.10	0.01	-0.74	$3.60 \cdot 10^{-7}$
slr1529-as1	asRNA	-1.73	-1.73	0.22	-0.18	-0.89	$1.16 \cdot 10^{-4}$
slr1535-as1	asRNA	-1.00	-1.10	-0.12	-0.39	-0.70	$1.15 \cdot 10^{-5}$
slr1550-as1	asRNA	-1.05	-1.09	-0.12	-0.16	-0.46	$6.82 \cdot 10^{-5}$
slr1579-as1	asRNA	-1.73	-1.95	0.05	-0.32	-0.84	$1.09 \cdot 10^{-6}$
slr1591-as1	asRNA	-2.26	-2.52	-1.88	-2.04	-1.82	$4.24 \cdot 10^{-6}$
slr1609-as2	asRNA	-1.14	-1.21	-0.18	-0.06	-0.71	$6.52 \cdot 10^{-7}$
slr1666-as1	asRNA	-1.45	-1.51	0.10	-0.32	-0.73	$4.50 \cdot 10^{-6}$
slr1673-as2	asRNA	-1.51	-1.38	-0.41	-0.49	-0.95	$8.02 \cdot 10^{-6}$
slr1676-as1	asRNA	-1.05	-1.02	-0.26	-0.33	-0.83	$3.82 \cdot 10^{-4}$
slr1679-as1	asRNA	-1.48	-1.67	-0.46	-0.10	-0.41	$1.18 \cdot 10^{-3}$
slr1691-as2	asRNA	-1.08	-0.94	-0.51	-0.18	-0.94	$3.00 \cdot 10^{-5}$
slr1704-as2	asRNA	-2.29	-2.27	-0.90	-0.29	-1.29	$1.87 \cdot 10^{-6}$
slr1727-as1	asRNA	-1.84	-1.89	-0.10	0.23	-0.92	$6.52 \cdot 10^{-3}$
slr1753-as2	asRNA	-1.11	-1.80	0.22	0.23	-0.48	$3.88 \cdot 10^{-6}$
slr1772-as1	asRNA	0.02	0.13	1.15	0.74	0.54	$8.67 \cdot 10^{-4}$
slr1777-as2	asRNA	-1.90	-2.07	-0.28	-0.47	-1.14	$8.45 \cdot 10^{-7}$
slr1780-as2	asRNA	-1.79	-1.65	-0.49	-0.45	-0.81	$3.43 \cdot 10^{-7}$
slr1839-as1	asRNA	-1.42	-1.43	0.02	-0.22	-0.64	$3.08 \cdot 10^{-7}$
slr1864-as1	asRNA	-1.49	-1.53	0.20	0.11	-0.63	$2.53 \cdot 10^{-5}$
slr1866-as1	asRNA	-1.34	-1.30	0.52	-0.06	-0.29	$2.25 \cdot 10^{-6}$
slr1876-as2	asRNA	-0.99	-1.09	-0.61	-0.46	-0.39	$3.87 \cdot 10^{-7}$
slr1876-as5	asRNA	-0.86	-1.01	-0.55	-0.41	-0.43	$1.77 \cdot 10^{-6}$
slr1908-as1	asRNA	-1.44	-1.26	0.28	-0.24	-0.69	$1.01 \cdot 10^{-6}$
slr1929-as1	asRNA	-1.31	-1.36	0.22	-0.31	-0.60	$3.16 \cdot 10^{-7}$
slr1942-as1	asRNA	-1.48	-1.37	0.26	-0.02	-0.68	$1.28 \cdot 10^{-5}$
slr1944-as2	asRNA	-1.57	-1.69	-0.35	-0.10	-0.81	$3.45 \cdot 10^{-5}$
slr1968-as1	asRNA	-2.46	-2.61	-1.84	-1.76	-1.85	$1.71 \cdot 10^{-5}$
slr1968-as3	asRNA	-1.21	-1.07	-0.06	-0.17	-0.11	$4.35 \cdot 10^{-6}$
slr2009-as1	asRNA	-1.10	-1.04	-0.06	-0.22	-0.75	$7.73 \cdot 10^{-6}$
slr2023-as1	asRNA	-1.48	-1.48	0.48	-0.01	-0.49	$3.13 \cdot 10^{-5}$
slr2046-as5	asRNA	-1.79	-1.25	-0.03	-0.45	-0.81	$9.26 \cdot 10^{-6}$
slr2046-as6	asRNA	-1.35	-1.31	-0.32	-0.31	-0.76	$3.59 \cdot 10^{-3}$
slr2076-as2	asRNA	-0.76	-1.07	-0.35	-0.42	-0.67	$1.62 \cdot 10^{-3}$
slr2141-as1	asRNA	-1.49	-1.41	-0.83	-0.25	-0.96	$3.39 \cdot 10^{-4}$
spoIID-0-x	sll1283-as	-2.02	-1.93	-0.67	-0.22	-0.91	$6.42 \cdot 10^{-8}$
ssl0294-as1	asRNA	-1.11	-1.00	-0.33	-0.25	-0.53	$7.66 \cdot 10^{-4}$
ssl0426-as2	asRNA	-0.66	-0.59	1.08	0.52	-0.25	$1.43 \cdot 10^{-4}$
ssl3177-as1	asRNA	-1.09	-1.04	-0.01	0.02	-0.49	$2.58 \cdot 10^{-5}$
ssl3436-as1	asRNA	-1.10	-1.22	0.08	0.39	-0.16	0.04
ssr1375-as1	asRNA	-0.96	-1.23	-0.42	-0.43	-0.53	$5.63 \cdot 10^{-6}$
ssr1407-as1	asRNA	-1.62	-1.65	-0.41	0.18	-0.75	$1.45 \cdot 10^{-5}$
ssr1558-as1	asRNA	-0.24	-0.35	1.05	0.31	-0.20	$1.19 \cdot 10^{-4}$
ssr1604-as1	asRNA	-1.11	-0.97	0.16	-0.06	-0.50	$6.10 \cdot 10^{-5}$
ssr2318-as1	asRNA	-1.17	-1.07	-0.16	0.02	-0.79	$8.53 \cdot 10^{-6}$
tktA-as2-0-x	asRNA	-1.36	-1.21	-0.33	-0.43	-0.89	$5.16 \cdot 10^{-5}$

6803t13-0-x	slr1740-as	0.51	0.48	-0.03	0.48	1.77	$1.53\cdot10^{-7}$
IsrR-0-x	sll0247-as	-1.09	-7.43	-6.56	-7.25	-7.55	$1.13\cdot10^{-10}$
NC4-0-x	sll1864-as	-1.97	-1.39	-1.46	0.17	-0.20	$1.38\cdot10^{-5}$
NC5-0-x	sll1864-as	-1.43	-0.79	-1.09	0.87	0.31	$6.51\cdot10^{-5}$
rpl1-0-x	sll1744-as	0.02	-0.24	-0.21	-1.17	-1.78	$1.90\cdot10^{-7}$
rpl1-1-x	sll1744-as	0.07	-0.16	-0.06	-1.09	-1.62	$4.20\cdot10^{-7}$
sll0002-as1	asRNA	-1.05	-1.10	-1.18	-0.84	-1.22	$1.86\cdot10^{-3}$
sll0027-as1	asRNA	-0.65	-0.82	-0.73	-1.02	-1.08	$1.49\cdot10^{-5}$
sll0107-as1	asRNA	-0.31	-1.12	-0.99	-0.95	-1.27	$3.68\cdot10^{-7}$
sll0217-as2	asRNA	0.98	1.17	0.86	1.06	1.29	$4.44\cdot10^{-7}$
sll0247-as2	asRNA	-1.06	-7.04	-5.93	-6.61	-6.89	$1.98\cdot10^{-10}$
sll0270-as1	asRNA	-0.34	-0.57	-1.02	-0.80	-1.05	$1.89\cdot10^{-5}$
sll0456-as1	asRNA	1.05	1.47	1.69	1.08	1.19	$3.70\cdot10^{-4}$
sll0477-as2	asRNA	3.18	3.96	3.78	4.12	4.27	$5.44\cdot10^{-8}$
sll0898-as1	asRNA	-2.31	-1.35	-1.70	-0.71	-0.79	$9.93\cdot10^{-8}$
sll1098-as1	asRNA	0.12	0.18	0.01	-0.84	-1.14	$1.43\cdot10^{-6}$
sll1198-as1	asRNA	2.80	3.97	3.82	4.34	4.54	$2.10\cdot10^{-9}$
sll1289-as2	asRNA	1.10	0.95	0.68	0.34	0.07	$1.48\cdot10^{-7}$
sll1319-as1	asRNA	-1.19	-0.84	-1.21	-0.72	-0.09	$2.45\cdot10^{-7}$
sll1321-as1	asRNA	-1.92	-1.39	-1.27	-0.44	-0.34	$6.42\cdot10^{-7}$
sll1374-as3	asRNA	-1.11	-1.37	-1.19	-0.85	-1.05	$1.33\cdot10^{-4}$
sll1658-as1	asRNA	1.01	0.78	0.83	0.29	0.26	$2.32\cdot10^{-3}$
sll1723-as1	asRNA	1.01	0.64	0.25	-0.23	-0.17	$1.58\cdot10^{-6}$
sll1851-as1	asRNA	0.28	0.19	-0.14	-0.03	1.26	$2.32\cdot10^{-5}$
sll1860-as1	asRNA	0.97	1.09	0.54	0.37	0.60	$1.93\cdot10^{-6}$
sll1864-as1	asRNA	-1.82	-1.08	-1.55	0.60	0.15	$9.12\cdot10^{-5}$
sll1867-as1	asRNA	-0.42	0.28	-1.77	-1.16	-0.65	$1.04\cdot10^{-4}$
sll1900-as1	asRNA	-0.07	0.69	-0.59	1.10	1.03	$5.14\cdot10^{-6}$
sll1939-as1	asRNA	-0.20	-0.05	-0.41	-1.03	-1.33	$2.25\cdot10^{-7}$
slr0015-as1	asRNA	0.78	-0.92	-0.55	-2.34	-1.71	$5.54\cdot10^{-9}$
slr0144-as1	asRNA	1.50	1.29	0.90	0.96	1.02	$9.61\cdot10^{-5}$
slr0300-as1	asRNA	-1.11	-0.30	-0.92	-0.84	-0.45	$1.63\cdot10^{-5}$
slr0431-as3	asRNA	0.97	1.06	0.44	0.39	0.36	$6.00\cdot10^{-7}$
slr1053-as2	asRNA	1.08	0.31	-0.97	-1.11	-1.13	$2.22\cdot10^{-6}$
slr1053-as3	asRNA	0.44	-0.28	-1.23	-1.15	-1.34	$2.78\cdot10^{-8}$
slr1053-as4	asRNA	0.45	-0.28	-1.33	-1.26	-1.40	$1.20\cdot10^{-8}$
slr1118-as1	asRNA	-1.11	-0.82	-0.95	-0.41	-0.26	$4.81\cdot10^{-4}$
slr1464-as2	asRNA	-1.16	-1.06	-1.34	-0.19	0.63	$5.09\cdot10^{-7}$
slr1467-as1	asRNA	0.81	0.79	1.57	0.58	0.89	$7.38\cdot10^{-5}$
slr1636-as1	asRNA	1.39	1.19	0.68	0.72	0.73	$1.24\cdot10^{-3}$
slr1740-as1	asRNA	0.24	0.25	-0.18	0.18	1.09	$2.03\cdot10^{-5}$
slr1776-as2	asRNA	-1.19	-1.46	-1.46	-1.12	-1.25	$9.92\cdot10^{-7}$
slr1878-as1	asRNA	0.99	1.07	0.60	0.55	0.72	$2.27\cdot10^{-6}$
slr1900-as2	asRNA	1.64	1.24	0.50	0.47	0.09	$2.64\cdot10^{-6}$
slr1919-as1	asRNA	1.42	1.32	0.96	0.94	0.90	$2.92\cdot10^{-3}$
slr1935-as4	asRNA	-1.05	-0.94	-0.92	-0.55	-0.66	$2.79\cdot10^{-6}$
slr1964-0-x	asRNA	1.20	1.26	0.94	1.23	1.11	$2.90\cdot10^{-6}$
slr2026-as2	asRNA	1.02	0.74	0.35	-0.23	-0.20	$7.68\cdot10^{-7}$
slr2042-as1	asRNA	-0.12	-0.45	-0.42	-0.75	-1.05	$9.27\cdot10^{-6}$
slr2132-as8	asRNA	-1.04	-0.84	-1.02	-0.56	-0.71	$1.70\cdot10^{-4}$
slr2143-as1	asRNA	1.08	0.92	0.54	0.15	-0.20	$9.97\cdot10^{-7}$
ssl3382-as2	asRNA	0.55	1.01	0.72	0.78	1.20	$3.27\cdot10^{-3}$
