# Supplementary material for: Iron Deprivation in Synechocystis: Inference of Pathways, Non-coding RNAs, and Regulatory Elements from Comprehensive Expression Profiling
Source: G3 (Bethesda). 2012 Dec 1;2(12):1475–95. doi: 10.1534/g3.112.003863 (PMC3516471; doi:10.1534/g3.112.003863)
Supplement: Supporting Information [file supp_2.12.1475_TableS7.pdf]

Table S7 Small RNAs differentially expressed. sRNAs are divided and colored like the three clusters defined in figure 4C (top cluster I; cluster II, light green; bottom cluster III).

Gene ID	Description	3 hours	12 hours	24 hours	48 hours	72 hours	*q*-value
NC-1082	sRNA	1.42	1.34	0.74	0.32	-0.01	$5.04 \cdot 10^{-6}$
NC-1096	sRNA	1.15	1.21	0.88	0.76	0.88	$6.71 \cdot 10^{-4}$
NC-1171	sRNA	-1.59	-1.74	-2.13	-2.88	-2.48	$3.04 \cdot 10^{-9}$
NC-1188	sRNA	-1.67	-1.46	-1.16	-2.07	-2.15	$1.85 \cdot 10^{-7}$
NC-119	sRNA	1.37	1.11	0.73	0.93	0.73	$1.84 \cdot 10^{-7}$
NC-1190	sRNA	-1.52	-1.29	-1.12	-2.09	-2.10	$5.86 \cdot 10^{-7}$
NC-1248	sRNA	1.93	2.11	0.78	-0.82	0.29	$1.40 \cdot 10^{-4}$
NC-1261	sRNA	1.01	0.75	0.74	0.22	0.41	$1.01 \cdot 10^{-5}$
NC-1399	sRNA	1.25	1.04	0.20	0.14	-0.30	$5.65 \cdot 10^{-6}$
NC-1410	sRNA	-2.67	-1.80	-2.13	-3.34	-2.98	$6.89 \cdot 10^{-6}$
NC-1413	sRNA	-2.58	-1.43	-2.22	-3.33	-2.86	$6.01 \cdot 10^{-6}$
NC-1414	sRNA	1.19	1.29	0.38	0.07	0.17	$5.09 \cdot 10^{-5}$
NC-1435	sRNA	-0.85	-0.86	-0.75	-1.27	-1.02	$9.07 \cdot 10^{-5}$
NC-1442	sRNA	1.22	1.08	0.94	0.82	0.80	$3.01 \cdot 10^{-5}$
NC-1481	sRNA	-1.43	-1.55	-2.27	-2.89	-2.64	$1.21 \cdot 10^{-8}$
NC-156	sRNA	0.97	0.98	-0.39	-0.79	-1.06	$7.15 \cdot 10^{-7}$
NC-1606	sRNA	0.08	-0.97	-1.09	-1.08	-1.15	$3.80 \cdot 10^{-6}$
NC-1637	sRNA	0.21	-0.59	-0.36	-1.14	-1.14	$1.24 \cdot 10^{-6}$
NC-1673	sRNA	-3.17	-4.12	-3.07	-4.60	-4.17	$1.07 \cdot 10^{-10}$
NC-1690	sRNA	1.10	1.01	0.69	0.35	0.39	$9.27 \cdot 10^{-4}$
NC-17	sRNA	-0.11	-0.63	-1.16	-1.07	-1.07	$2.04 \cdot 10^{-7}$
NC-172	sRNA	-0.66	-0.12	-0.85	-1.23	-0.79	$8.79 \cdot 10^{-6}$
NC-176	sRNA	-0.59	0.00	-1.11	-1.27	-0.84	$3.01 \cdot 10^{-6}$
NC-196	sRNA	3.65	2.94	3.04	1.55	2.95	$1.32 \cdot 10^{-7}$
NC-271	sRNA	1.21	0.82	0.47	0.13	0.03	$9.28 \cdot 10^{-6}$
NC-285	sRNA	1.03	0.83	0.57	0.16	0.36	$1.80 \cdot 10^{-3}$
NC-3	sRNA	-0.43	-0.56	-0.97	-1.04	-0.93	$5.01 \cdot 10^{-7}$
NC-318	sRNA	1.78	1.21	1.36	0.12	0.61	$5.03 \cdot 10^{-6}$
NC-334	sRNA	1.80	2.49	0.81	0.47	0.38	$3.18 \cdot 10^{-8}$
NC-349	sRNA	1.29	0.84	0.08	-0.28	-0.45	$2.32 \cdot 10^{-7}$
NC-380	sRNA	1.86	1.62	1.57	0.68	0.91	$1.19 \cdot 10^{-6}$
NC-392	sRNA	-1.44	-1.53	-2.00	-2.54	-2.21	$6.71 \cdot 10^{-7}$
NC-407	sRNA	0.99	0.80	1.11	0.71	0.78	$3.29 \cdot 10^{-4}$
NC-430	sRNA	0.99	1.34	0.61	0.64	0.64	$3.47 \cdot 10^{-6}$
NC-431	sRNA	1.17	1.73	1.18	1.01	1.20	$1.12 \cdot 10^{-6}$
NC-445	sRNA	1.51	1.30	1.04	0.92	0.67	$3.32 \cdot 10^{-7}$
NC-492	sRNA	1.36	1.19	0.40	0.40	0.60	$3.38 \cdot 10^{-7}$
NC-520	sRNA	-0.50	-0.95	-1.32	-0.87	-0.97	$7.75 \cdot 10^{-7}$
NC-711	sRNA	2.12	1.86	0.73	0.12	0.00	$1.21 \cdot 10^{-6}$
NC-717	sRNA	1.17	0.86	-0.24	-0.37	-0.37	$2.22 \cdot 10^{-5}$
NC-83	sRNA	1.64	0.56	0.49	-0.02	-0.18	$7.73 \cdot 10^{-6}$
NC-841	sRNA	-1.14	-1.15	-1.49	-1.46	-1.42	$3.19 \cdot 10^{-5}$
NC-954	sRNA	-0.22	-0.75	-0.57	-1.38	-1.21	$8.41 \cdot 10^{-8}$
NC-995	sRNA	1.77	1.07	1.70	0.96	0.74	$7.37 \cdot 10^{-5}$
SyR2-0-x	sRNA	1.79	1.52	0.16	-0.09	-0.35	$4.92 \cdot 10^{-9}$
NC-108	sRNA	-1.47	-1.42	-0.56	-0.15	-0.89	$1.93 \cdot 10^{-5}$
NC1-0-x	sRNA	0.12	0.00	1.00	0.18	-0.13	$3.72 \cdot 10^{-4}$
NC-1103	sRNA	-0.59	-0.58	-0.54	-0.71	-1.70	$1.35 \cdot 10^{-6}$
NC-1134	sRNA	-1.30	-1.85	-0.81	-0.37	-0.68	$1.09 \cdot 10^{-6}$
NC-1136	sRNA	-1.29	-1.83	-0.66	-0.35	-0.44	$5.93 \cdot 10^{-7}$
NC-1148	sRNA	-1.48	-1.49	-0.28	-0.34	-0.72	$2.33 \cdot 10^{-5}$
NC-1331	sRNA	-1.21	-1.23	-0.06	-0.43	-0.92	$7.90 \cdot 10^{-8}$
NC-1484	sRNA	-1.20	-1.10	-0.32	-0.45	-1.05	$2.20 \cdot 10^{-6}$
NC-1491	sRNA	-1.86	-1.83	-0.37	-0.01	-1.07	$5.10 \cdot 10^{-7}$
NC-1499	sRNA	-1.44	-1.59	-1.29	-1.57	-1.85	$2.73 \cdot 10^{-7}$
NC-163	sRNA	-1.07	-1.33	-0.42	-0.41	-0.57	$6.28 \cdot 10^{-5}$
NC-1687	sRNA	-1.07	-0.89	-0.52	-0.23	-0.30	$2.04 \cdot 10^{-6}$
NC-1703	sRNA	-1.07	-1.33	0.00	-0.03	-0.57	$1.86 \cdot 10^{-4}$
NC-1706	sRNA	1.24	0.53	1.39	0.92	0.54	$6.68 \cdot 10^{-4}$
NC-1722	sRNA	0.88	0.16	1.31	0.97	0.34	$8.79 \cdot 10^{-6}$

NC-1731	sRNA	-1.45	-1.34	-0.37	0.23	-0.37	$3.01 \cdot 10^{-6}$
NC-1737	sRNA	-2.22	-2.22	-0.86	-0.63	-1.25	$3.30 \cdot 10^{-6}$
NC-207	sRNA	-0.23	-0.17	1.03	0.56	0.62	$5.46 \cdot 10^{-4}$
NC2-0-x	sRNA	0.65	0.66	1.09	0.66	0.81	$2.08 \cdot 10^{-5}$
NC-211	sRNA	-1.07	-1.34	-0.52	0.04	-0.57	$2.60 \cdot 10^{-3}$
NC-232	sRNA	-0.73	-1.16	-0.04	0.17	0.04	$2.06 \cdot 10^{-6}$
NC-244	sRNA	0.01	-0.62	1.55	0.88	0.37	$6.99 \cdot 10^{-5}$
NC-247	sRNA	-0.61	-1.17	-0.19	-0.03	-0.12	$8.11 \cdot 10^{-7}$
NC-265	sRNA	0.27	0.48	1.85	1.40	1.52	$2.49 \cdot 10^{-3}$
NC-269	sRNA	-1.21	-0.54	0.01	0.42	0.13	$3.64 \cdot 10^{-3}$
NC-272	sRNA	-1.27	-1.12	-0.56	-0.17	-0.15	$1.32 \cdot 10^{-6}$
NC-282	sRNA	-1.98	-2.27	-1.08	-1.20	-1.84	$1.92 \cdot 10^{-6}$
NC-288	sRNA	-1.65	-1.79	-0.18	0.22	-0.22	$2.03 \cdot 10^{-6}$
NC-29	sRNA	-0.69	-1.10	0.57	-0.65	-1.03	$5.19 \cdot 10^{-6}$
NC-296	sRNA	-1.39	-1.18	-0.97	-0.82	-0.82	$6.04 \cdot 10^{-6}$
NC-298	sRNA	-1.72	-1.42	-0.46	-0.46	-0.52	$9.97 \cdot 10^{-7}$
NC-309	sRNA	0.49	0.09	0.53	1.67	0.87	$8.52 \cdot 10^{-5}$
NC-347	sRNA	-1.95	-1.94	-0.86	-0.80	-1.38	$1.71 \cdot 10^{-7}$
NC-41	sRNA	-0.94	-1.22	0.07	-0.20	0.05	$1.29 \cdot 10^{-6}$
NC-421	sRNA	-1.08	-1.11	-0.59	-0.32	-0.56	$2.62 \cdot 10^{-6}$
NC-425	sRNA	-1.08	-1.55	-0.58	-0.17	-0.30	$4.26 \cdot 10^{-8}$
NC-433	sRNA	-1.93	-1.93	0.00	-0.23	-0.61	$1.96 \cdot 10^{-7}$
NC-449	sRNA	0.68	0.55	0.99	1.37	1.30	$3.41 \cdot 10^{-6}$
NC-516	sRNA	-0.71	-0.83	-0.71	-0.77	-1.01	$3.85 \cdot 10^{-4}$
NC-667	sRNA	0.02	0.11	1.22	0.20	0.21	$1.31 \cdot 10^{-5}$
NC-681	sRNA	-0.32	-0.96	1.06	0.77	-0.40	$5.08 \cdot 10^{-5}$
NC-693	sRNA	-1.10	-1.11	0.60	0.13	-0.24	$3.66 \cdot 10^{-4}$
NC-764	sRNA	-0.69	-1.08	-0.64	-0.27	-0.24	$1.74 \cdot 10^{-5}$
NC-766	sRNA	-1.05	-1.05	-0.32	-0.02	-0.61	$1.13 \cdot 10^{-5}$
NC-882	sRNA	-2.41	-2.60	-1.55	-1.61	-1.69	$5.16 \cdot 10^{-7}$
NC-895	sRNA	-0.59	-1.41	-0.87	-1.36	-1.13	$1.40 \cdot 10^{-6}$
NC-980	sRNA	-0.82	-1.49	-0.54	-0.80	-0.50	$2.42 \cdot 10^{-6}$
NC-981	sRNA	-0.39	-1.11	-0.44	-0.75	-0.57	$1.84 \cdot 10^{-5}$
ncRNA 983563:983626	sRNA	-0.69	-1.09	0.59	-0.67	-1.04	$4.90 \cdot 10^{-6}$
SyR4-0-x	sRNA	-1.61	-1.44	-1.03	-1.29	-1.27	$9.53 \cdot 10^{-5}$
NC-1036	sRNA	1.94	1.75	1.64	1.72	3.82	$7.75 \cdot 10^{-7}$
NC-110	sRNA	-2.05	-1.83	-1.97	-1.62	-1.77	$9.02 \cdot 10^{-7}$
NC-111	sRNA	0.81	1.03	0.30	0.69	0.81	$4.79 \cdot 10^{-7}$
NC-1208	sRNA	-0.10	0.12	0.22	0.68	1.49	$1.66 \cdot 10^{-7}$
NC-1321	sRNA	0.40	1.36	0.68	1.54	1.67	$5.32 \cdot 10^{-8}$
NC-137	sRNA	-1.51	-0.41	-2.02	-0.71	-0.55	$1.77 \cdot 10^{-4}$
NC-1371	sRNA	-1.72	-1.90	-1.64	-1.50	-1.18	$5.96 \cdot 10^{-6}$
NC-1382	sRNA	0.83	1.00	-0.29	0.59	0.72	$3.05 \cdot 10^{-6}$
NC-142	sRNA	-0.41	0.26	-1.43	-0.13	0.09	$2.93 \cdot 10^{-5}$
NC-1453	sRNA	-1.14	-0.89	-0.75	-0.08	0.11	$8.07 \cdot 10^{-7}$
NC-1567	sRNA	-3.25	-1.65	-2.22	-3.05	-2.37	$1.79 \cdot 10^{-5}$
NC-1619	sRNA	-0.48	-0.07	-1.06	-0.63	-0.28	$2.66 \cdot 10^{-3}$
NC-1630	sRNA	0.24	0.53	-0.17	-0.17	1.15	$1.52 \cdot 10^{-6}$
NC-168	sRNA	-1.00	-0.52	-2.15	-0.70	-0.10	$2.40 \cdot 10^{-4}$
NC-181	sRNA	1.54	3.32	3.34	3.75	3.77	$3.85 \cdot 10^{-8}$
NC-233	sRNA	-0.67	0.37	-1.02	-0.68	0.00	$1.83 \cdot 10^{-4}$
NC-253	sRNA	-1.37	-1.21	-1.24	-1.09	-0.99	$7.48 \cdot 10^{-5}$
NC-316	sRNA	0.64	0.61	0.38	1.14	0.51	$4.39 \cdot 10^{-4}$
NC-319	sRNA	-0.74	1.68	0.94	0.22	0.85	$6.46 \cdot 10^{-6}$
NC-324	sRNA	-1.23	0.62	0.61	0.22	0.39	$6.50 \cdot 10^{-4}$
NC-330	sRNA	-0.82	-0.18	-1.09	-0.54	-0.67	$1.49 \cdot 10^{-3}$
NC-350	sRNA	0.38	1.07	0.51	0.84	1.52	$8.03 \cdot 10^{-8}$
NC-361	sRNA	-0.18	0.35	-1.53	-0.08	0.10	$3.11 \cdot 10^{-5}$
NC-404	sRNA	-0.22	0.33	-1.66	-0.02	-0.04	$3.47 \cdot 10^{-5}$
NC-436	sRNA	0.75	0.76	0.75	1.38	1.30	$4.85 \cdot 10^{-5}$
NC-52	sRNA	-1.40	-1.24	-1.13	-0.56	-0.73	$1.25 \cdot 10^{-6}$
NC-688	sRNA	-0.14	0.39	-0.02	0.55	1.01	$1.50 \cdot 10^{-3}$
NC-833	sRNA	-2.12	-1.31	-1.25	0.19	0.23	$4.38 \cdot 10^{-7}$
NC-870	sRNA	0.76	1.15	0.86	0.69	1.09	$8.96 \cdot 10^{-6}$

NC-91	sRNA	-1.11	-0.63	-0.86	-0.51	-0.50	$1.33{\cdot}10^{-4}$
