# Supplementary material for: Iron Deprivation in Synechocystis: Inference of Pathways, Non-coding RNAs, and Regulatory Elements from Comprehensive Expression Profiling
Source: G3 (Bethesda). 2012 Dec 1;2(12):1475–95. doi: 10.1534/g3.112.003863 (PMC3516471; doi:10.1534/g3.112.003863)
Supplement: Supporting Information [file supp_2.12.1475_TableS8.pdf]

Table S8 Predicted targets for the sRNAs induced during iron starvation, i.e. NC-181, NC-1321, NC-265, and NC-350 (*r*: spearman correlation coefficient)

NC-181 Targets	Description	Energy score	*r*	3 h	12 h	24 h	48 h	72 h	*q*-value
slr1920	unknown protein	-10.19	-0.98	1.50	0.80	0.92	0.63	0.73	$1.74 \cdot 10^{-6}$
sll0017	glutamate-1-semialdehyde aminomutase	-11.52	-0.96	1.83	1.07	1.12	0.76	1.04	$3.53 \cdot 10^{-6}$
ssr0330	ferredoxin-thioredoxin reductase	-12.00	-0.95	1.12	0.71	0.62	0.32	0.46	$2.18 \cdot 10^{-6}$
slr1516	superoxide dismutase	-11.42	-0.94	-0.27	-1.42	-1.06	-1.88	-1.58	$8.29 \cdot 10^{-8}$
sll1029	carbon dioxide concentrating mechanism protein	-14.12	-0.93	1.05	0.36	0.10	-0.43	-0.42	$2.70 \cdot 10^{-7}$
slr0708	periplasmic protein, function unknown	-11.29	-0.92	0.06	-1.03	-1.36	-1.03	-1.11	$2.24 \cdot 10^{-6}$
sll1323	ATP synthase subunit b' of CF(0)	-10.48	-0.91	1.16	0.47	-0.03	-0.45	-0.71	$6.72 \cdot 10^{-8}$
ssl0020	ferredoxin I	-20.56	-0.90	0.33	-1.57	-0.82	-2.72	-2.40	$9.52 \cdot 10^{-9}$
slr1165	sulfate adenylyltransferase	-13.33	-0.90	1.04	0.56	0.38	-0.03	-0.20	$2.68 \cdot 10^{-6}$
sll1326	ATP synthase alpha chain	-18.82	-0.90	1.05	0.46	-0.20	-0.54	-0.64	$1.39 \cdot 10^{-7}$
sll1119	hypothetical protein	-13.29	-0.89	1.50	0.84	0.62	0.11	0.68	$3.64 \cdot 10^{-7}$
slr1200	urea transport system permease protein	-14.39	-0.89	1.79	1.01	0.93	-0.07	0.47	$9.96 \cdot 10^{-8}$
slr0447	ABC-type urea transport system substrate-binding protein	-10.84	-0.88	2.15	0.84	0.90	-0.87	-0.57	$9.16 \cdot 10^{-8}$
slr1281	NADH dehydrogenase subunit I	-10.82	-0.88	-1.01	-1.32	-1.62	-1.96	-1.73	$1.20 \cdot 10^{-7}$
sll0108	ammonium/methylammonium permease	-14.15	-0.87	2.01	1.41	0.89	0.05	0.36	$7.15 \cdot 10^{-8}$
slr1329	ATP synthase beta subunit	-11.50	-0.86	1.61	1.33	1.14	0.70	0.82	$2.46 \cdot 10^{-5}$
sll1069	3-oxoacyl-[acyl-carrier-protein] synthase II	-11.35	-0.86	1.19	0.96	0.70	0.50	0.34	$4.15 \cdot 10^{-7}$
slr1166	UDP-glucose:tetrahydrobiopterin glucosyltransferase	-10.62	-0.86	1.01	0.45	0.55	0.36	-0.14	$6.82 \cdot 10^{-5}$
slr1431	hypothetical protein	-12.04	-0.84	1.24	0.94	0.89	0.38	0.30	$1.37 \cdot 10^{-6}$
slr0434	elongation factor P	-10.02	-0.83	1.12	0.90	0.64	0.18	0.15	$8.13 \cdot 10^{-7}$
slr1945	2,3-bisphosphoglycerate-independent phosphoglycerate mutase	-13.87	-0.82	1.26	0.93	0.60	-0.19	-0.09	$3.76 \cdot 10^{-5}$
slr1280	NADH dehydrogenase subunit	-25.97	-0.81	-0.90	-1.03	-1.27	-1.64	-1.54	$2.35 \cdot 10^{-5}$
slr1986	allophycocyanin beta subunit	-11.93	-0.81	1.31	1.01	0.07	0.02	-0.37	$3.19 \cdot 10^{-7}$
ssr3383	phycobilisome small core linker polypeptide	-10.86	-0.80	1.53	1.21	0.15	0.03	-0.45	$2.09 \cdot 10^{-7}$
sll0421	adenylosuccinate lyase	-10.50	-0.80	1.24	0.98	0.83	0.27	0.12	$3.72 \cdot 10^{-7}$
ssr0390	photosystem I reaction center subunit X	-10.86	-0.80	1.08	0.85	-0.12	-0.30	-0.44	$4.44 \cdot 10^{-8}$
sll0630	unknown protein	-10.47	-0.80	1.14	0.85	0.27	-0.46	-0.60	$1.58 \cdot 10^{-7}$
sll0374	urea transport system ATP-binding	-11.58	-0.79	1.11	0.90	0.75	0.10	0.09	$7.28 \cdot 10^{-7}$

	protein								
slr1853	carboxymuconolactone decarboxylase	-11.96	-0.78	1.12	0.89	-0.36	-0.38	-0.68	$2.53{\cdot}10^{-8}$
slr1756	glutamate--ammonia ligase	-11.53	-0.78	2.53	2.20	1.73	1.19	1.85	$6.69{\cdot}10^{-8}$
slr1279	NADH dehydrogenase subunit 3	-13.60	-0.76	-1.17	-1.29	-1.67	-2.29	-2.14	$2.16{\cdot}10^{-5}$
sll1942	unknown protein	-14.01	-0.75	1.22	1.09	0.98	0.47	0.39	$1.83{\cdot}10^{-5}$
slr1351	UDP-N-acetylmuramoylalanyl-D-glutamyl-2 6-diaminopimelate--D-alanyl-D-alanine ligase	-11.68	-0.74	-0.37	-0.94	-0.44	-0.99	-1.23	$1.02{\cdot}10^{-5}$
slr0338	probable oxidoreductase	-14.22	-0.74	1.30	1.21	1.04	0.50	0.60	$5.10{\cdot}10^{-7}$
sll1398	photosystem II reaction center 13 kDa protein	-10.31	-0.73	1.57	1.56	1.04	0.86	0.92	$1.22{\cdot}10^{-6}$
sll1281	photosystem II PsbZ protein	-12.93	-0.73	1.25	0.86	0.07	-0.05	0.66	$1.41{\cdot}10^{-7}$
slr0993	putative peptidase, lipoprotein NlpD	-16.05	-0.72	1.21	1.02	0.83	-0.38	-0.26	$3.73{\cdot}10^{-5}$
slr0423	hypothetical protein	-12.88	-0.72	1.15	1.07	0.85	0.28	0.59	$4.81{\cdot}10^{-7}$
ssr1600	similar to anti-sigma f factor antagonist	-13.62	-0.70	1.38	1.41	0.78	0.67	0.63	$9.18{\cdot}10^{-8}$
slr1187	unknown protein	-12.09	-0.70	1.00	1.02	0.62	0.13	0.04	$2.14{\cdot}10^{-6}$
slr1437	unknown protein	-12.40	-0.67	-0.34	-0.55	-1.76	-1.43	-1.13	$2.86{\cdot}10^{-4}$
sll1185	coproporphyrinogen III oxidase, aerobic (oxygen-dependent)	-11.60	-0.61	1.76	1.59	0.09	0.85	0.81	$4.21{\cdot}10^{-8}$
sll1242	hypothetical protein	-12.74	-0.56	1.29	1.46	0.92	0.72	0.89	$1.03{\cdot}10^{-5}$
slr1545	RNA polymerase ECF-type (group 3) sigma-E factor	-11.73	-0.54	-0.29	-0.19	-0.32	-0.73	-1.10	$9.86{\cdot}10^{-7}$
ssl2982	probable DNA-directed RNA polymerase omega subunit	-10.35	-0.50	1.14	1.17	0.74	0.80	1.02	$1.21{\cdot}10^{-5}$

NC-1321 Targets	Description	Energy score	*r*	3 h	12 h	24 h	48 h	72 h	*q*-value
slr0923	hypothetical protein YCF65	-11.04	-0.53	1.46	1.40	0.79	0.55	0.70	$2.84{\cdot}10^{-7}$
slr1887	porphobilinogen deaminase	-12.39	-0.57	1.23	1.07	0.64	0.46	0.61	$2.50{\cdot}10^{-5}$
sll0615	hypothetical protein	-10.50	-0.60	-0.36	-1.83	-0.53	-0.74	-1.02	$2.03{\cdot}10^{-6}$
ssl0410	unknown protein	-10.04	-0.62	-0.46	-0.57	-0.86	-1.14	-0.93	$2.64{\cdot}10^{-5}$
slr1908	probable porin	-11.79	-0.63	1.13	1.21	0.89	0.64	0.34	$2.65{\cdot}10^{-7}$
slr1364	biotin synthetase	-10.67	-0.83	1.09	0.82	0.73	0.41	0.50	$5.53{\cdot}10^{-5}$

NC-265 Targets	Description	Energy score	*r*	3 h	12 h	24 h	48 h	72 h	*q*-value
slr2002	cyanophycin synthetase	-10.48	-0.51	1.46	1.02	1.00	0.43	1.10	$2.65{\cdot}10^{-7}$
slr1545	RNA polymerase ECF-type (group 3) sigma-E factor	-11.45	-0.53	-0.29	-0.19	-0.32	-0.73	-1.10	$9.86{\cdot}10^{-7}$
sll1654	hypothetical protein	-14.39	-0.55	-0.46	-0.22	-1.14	-0.36	-0.30	$4.48{\cdot}10^{-3}$
ssr1155	hypothetical protein	-12.68	-0.58	-0.69	-0.19	-0.83	-1.17	-0.70	$1.06{\cdot}10^{-6}$

slr1166	UDP-glucose:tetrahydrobiopterin glucosyltransferase	-10.63	-0.59	1.01	0.45	0.55	0.36	-0.14	$6.82 \cdot 10^{-5}$
slr0993	putative peptidase	-10.66	-0.61	1.21	1.02	0.83	-0.38	-0.26	$3.73 \cdot 10^{-5}$
slr0559	periplasmic binding protein of ABC transporter for natural amino acids	-11.17	-0.62	1.05	0.47	0.57	0.04	0.07	$6.07 \cdot 10^{-6}$
sll1583	unknown protein	-10.76	-0.66	0.53	1.02	-0.28	0.54	0.64	$1.78 \cdot 10^{-3}$
slr0338	probable oxidoreductase	-10.77	-0.67	1.30	1.21	1.04	0.50	0.60	$5.10 \cdot 10^{-7}$
sll1270	Bgt permease for basic amino acids and glutamine BgtB	-11.60	-0.70	1.62	1.24	0.86	-0.30	0.02	$1.10 \cdot 10^{-7}$
slr1364	biotin synthetase	-10.07	-0.73	1.09	0.82	0.73	0.41	0.50	$5.53 \cdot 10^{-5}$
slr1165	sulfate adenylyltransferase	-10.14	-0.76	1.04	0.56	0.38	-0.03	-0.20	$2.68 \cdot 10^{-6}$
slr1622	soluble inorganic pyrophosphatase	-12.83	-0.76	1.55	1.15	0.95	0.46	0.60	$4.94 \cdot 10^{-7}$
sll1029	carbon dioxide concentrating mechanism protein	-13.01	-0.78	1.05	0.36	0.10	-0.43	-0.42	$2.70 \cdot 10^{-7}$
sll1342	NAD(P)-dependent glyceraldehyde-3-phosphate dehydrogenase	-17.09	-0.81	1.45	1.22	1.02	0.76	0.92	$1.68 \cdot 10^{-5}$
sll1898	hypothetical protein	-10.35	-0.82	0.52	1.20	-0.21	-0.59	-0.48	$3.90 \cdot 10^{-8}$
sll1069	3-oxoacyl-[acyl-carrier-protein] synthase II	-10.69	-0.83	1.19	0.96	0.70	0.50	0.34	$4.15 \cdot 10^{-7}$
slr1847	hypothetical protein	-10.61	-0.85	1.37	1.40	0.91	0.61	0.67	$2.94 \cdot 10^{-7}$
slr1708	probable peptidase	-11.79	-0.86	1.23	1.20	0.64	0.38	0.22	$8.49 \cdot 10^{-6}$
slr0348	hypothetical protein	-11.55	-0.86	1.09	1.02	0.85	0.72	0.78	$3.82 \cdot 10^{-7}$
ssl2982	probable DNA-directed RNA polymerase omega subunit	-15.71	-0.87	1.14	1.17	0.74	0.80	1.02	$1.21 \cdot 10^{-5}$
sll1326	ATP synthase alpha chain	-11.26	-0.88	1.05	0.46	-0.20	-0.54	-0.64	$1.39 \cdot 10^{-7}$
slr1020	sulfolipid biosynthesis protein	-11.94	-0.88	1.09	0.81	0.48	0.30	0.29	$9.50 \cdot 10^{-6}$
sll1398	photosystem II reaction center 13 kDa protein	-17.30	-0.91	1.57	1.56	1.04	0.86	0.92	$1.22 \cdot 10^{-6}$
slr1887	porphobilinogen deaminase (hydroxymethylbilane synthase, preuroporphyrinogen synthase)	-20.08	-0.91	1.23	1.07	0.64	0.46	0.61	$2.50 \cdot 10^{-5}$
ssl1498	hypothetical protein	-10.87	-0.93	1.07	0.81	0.03	-0.16	-0.23	$3.30 \cdot 10^{-7}$
ssr1600	similar to anti-sigma f factor antagonist	-11.31	-0.93	1.38	1.41	0.78	0.67	0.63	$9.18 \cdot 10^{-8}$
sll1091	geranylgeranyl hydrogenase	-10.44	-0.95	1.39	1.51	-0.23	0.28	0.59	$3.29 \cdot 10^{-8}$
slr0335	phycobilisome core-membrane linker polypeptide	-10.80	-0.99	1.26	1.01	-0.02	0.20	0.05	$3.25 \cdot 10^{-7}$

**NC-350 Targets**	**Description**	**Energy score**	***r***	**3 h**	**12 h**	**24 h**	**48 h**	**72 h**	***q***
slr1166	UDP-glucose:tetrahydrobiopterin glucosyltransferase	-11.28	-0.91	1.01	0.45	0.55	0.36	-0.14	$6.82 \cdot 10^{-5}$

slr1215	hypothetical protein	-11.63	-0.80	-0.78	-1.44	-0.73	-1.16	-1.24	$6.12 \cdot 10^{-7}$
slr1835	P700 apoprotein subunit Ib	-10.43	-0.75	0.34	-0.41	-0.54	-0.96	-1.16	$4.26 \cdot 10^{-8}$
slr0559	periplasmic binding protein of ABC transporter for natural amino acids	-11.36	-0.75	1.05	0.47	0.57	0.04	0.07	$6.07 \cdot 10^{-6}$
slr0676	adenylylsulfate kinase	-10.15	-0.74	1.26	0.97	0.98	0.33	0.26	$8.03 \cdot 10^{-7}$
sll0421	adenylosuccinate lyase	-12.55	-0.71	1.24	0.98	0.83	0.27	0.12	$3.72 \cdot 10^{-7}$
slr0447	periplasmic protein, ABC-type urea transport system substrate-binding protein	-10.55	-0.69	2.15	0.84	0.90	-0.87	-0.57	$9.16 \cdot 10^{-8}$
sll0262	acyl-lipid desaturase (delta 6)	-15.39	-0.66	1.11	0.89	0.72	0.13	0.09	$4.87 \cdot 10^{-5}$
sll1069	3-oxoacyl-[acyl-carrier-protein] synthase II	-12.79	-0.66	1.19	0.96	0.70	0.50	0.34	$4.15 \cdot 10^{-7}$
sll1029	carbon dioxide concentrating mechanism protein CcmK	-10.12	-0.65	1.05	0.36	0.10	-0.43	-0.42	$2.70 \cdot 10^{-7}$
slr0879	glycine decarboxylase complex H-protein	-13.00	-0.63	1.21	0.90	0.72	0.40	0.41	$5.73 \cdot 10^{-7}$
sll0689	Na+/H+ antiporter	-15.07	-0.62	1.17	0.83	0.57	-0.34	-0.25	$2.21 \cdot 10^{-4}$
slr1096	dihydrolipoamide dehydrogenase	-10.42	-0.61	1.03	0.65	0.51	0.19	0.27	$6.28 \cdot 10^{-5}$
sll0630	unknown protein	-10.66	-0.60	1.14	0.85	0.27	-0.46	-0.60	$1.58 \cdot 10^{-7}$
slr1622	soluble inorganic pyrophosphatase	-11.70	-0.59	1.55	1.15	0.95	0.46	0.60	$4.94 \cdot 10^{-7}$
slr0007	probable sugar-phosphate nucleotidyltransferase	-23.42	-0.57	-1.07	-0.92	-0.97	-1.57	-1.62	$6.41 \cdot 10^{-7}$
sll1638	hypothetical protein	-13.64	-0.57	1.58	1.38	1.00	0.98	0.79	$1.03 \cdot 10^{-7}$
ssr3383	phycobilisome small core linker polypeptide	-11.36	-0.55	1.53	1.21	0.15	0.03	-0.45	$2.09 \cdot 10^{-7}$
slr1020	sulfolipid biosynthesis protein SqdB	-11.71	-0.54	1.09	0.81	0.48	0.30	0.29	$9.50 \cdot 10^{-6}$
sll0045	sucrose phosphate synthase	-10.09	-0.54	-0.69	-0.59	-0.45	-0.20	-1.04	$7.34 \cdot 10^{-3}$
slr1986	allophycocyanin beta subunit	-13.06	-0.53	1.31	1.01	0.07	0.02	-0.37	$3.19 \cdot 10^{-7}$
sll0108	ammonium/methylammonium permease	-20.54	-0.53	2.01	1.41	0.89	0.05	0.36	$7.15 \cdot 10^{-8}$
sll1452	nitrate/nitrite transport system ATP-binding protein	-11.66	-0.52	1.25	0.59	1.69	0.92	1.10	$8.61 \cdot 10^{-8}$
